# A 3D Bioprinted Cortical Organoid Platform for Modeling Human Brain Development

**DOI:** 10.1002/adhm.202401603

**Published:** 2024-06-08

**Authors:** Melissa A. Cadena, Anson Sing, Kylie Taylor, Linqi Jin, Liqun Ning, Mehdi Salar Amoli, Yamini Singh, Samantha N. Lanjewar, Martin L. Tomov, Vahid Serpooshan, Steven A. Sloan

**Affiliations:** ^1^ Department of Biomedical Engineering Emory University School of Medicine and Georgia Institute of Technology Atlanta GA 30322 USA; ^2^ Department of Human Genetics Emory University School of Medicine Atlanta GA 30322 USA; ^3^ Department of Mechanical Engineering Cleveland State University Cleveland OH 44115 USA; ^4^ Department of Pediatrics Emory University School of Medicine Atlanta GA 30322 USA; ^5^ Children's Healthcare of Atlanta Atlanta GA 30322 USA

**Keywords:** 3D bioprinting, brain organoids, extracellular matrix, induced pluripotent stem cells, vasculature

## Abstract

The ability to promote three‐dimensional (3D) self‐organization of induced pluripotent stem cells into complex tissue structures called organoids presents new opportunities for the field of developmental biology. Brain organoids have been used to investigate principles of neurodevelopment and neuropsychiatric disorders and serve as a drug screening and discovery platform. However, brain organoid cultures are currently limited by a lacking ability to precisely control their extracellular environment. Here, this work employs 3D bioprinting to generate a high‐throughput, tunable, and reproducible scaffold for controlling organoid development and patterning. Additionally, this approach supports the coculture of organoids and vascular cells in a custom architecture containing interconnected endothelialized channels. Printing fidelity and mechanical assessments confirm that fabricated scaffolds closely match intended design features and exhibit stiffness values reflective of the developing human brain. Using organoid growth, viability, cytoarchitecture, proliferation, and transcriptomic benchmarks, this work finds that organoids cultured within the bioprinted scaffold long‐term are healthy and have expected neuroectodermal differentiation. Lastly, this work confirms that the endothelial cells (ECs) in printed channel structures can migrate toward and infiltrate into the embedded organoids. This work demonstrates a tunable 3D culturing platform that can be used to create more complex and accurate models of human brain development and underlying diseases.

## Graphical abstract

Embedded 3D bioprinting with gelatin methacrylate bioink creates a highly tunable in vitro culturing scaffold for brain organoid development. Predefined architectural features support the incorporation of endothelial cells (ECs) and enables the investigation of organoid–endothelial cell interactions.

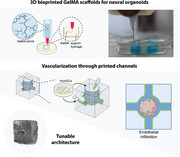

## 1 Introduction

Cortical organoids, derived from human embryonic stem cells (hESCs) or induced pluripotent stem cells (hiPSCs) provide a robust three‐dimensional (3D) in vitro model system to study human brain development and disease.^[^ ^1^, ^2^, ^3^, ^4^ ^]^ Importantly, cortical organoids contain multiple cell types present in the human brain, such as neural progenitors, neurons, and astrocytes,^[^ ^5^ ^]^ and recapitulate microarchitectural aspects of the fetal neocortex.^[^ ^6^, ^7^, ^8^ ^]^ However, organoid complexity remains limited by challenges in controlling the extracellular matrix (ECM) in which these tissues are grown. Meanwhile, in the fields of tissue engineering and regenerative medicine, rapid advances are enabling the creation of complex tissue geometries.^[^ ^6^, ^7^, ^8^ ^]^ These technologies offer the opportunity to increase the spatial control and environmental/compositional complexity of 3D organoid cultures.

Many improvements have led to more precise control of the differentiation, organization, and survival of cells within organoid cultures. These include slice cultures to improve neuronal layering and viability,^[^ ^9^ ^]^ fusing organoids patterned from different brain regions,^[^ ^10^, ^11^ ^]^ and integrating signaling centers to simulate extrinsic morphogen gradients.^[^ ^12^ ^]^ While these efforts have improved organoid complexity, the platform continues to lack two critical components of brain development—defined ECM characteristics and vascularization. The physical properties of the brain ECM directly influence neural progenitor proliferation,^[^ ^13^, ^14^ ^]^ differentiation,^[^ ^15^ ^]^ migration,^[^ ^16^, ^17^ ^]^ and circuit connectivity.^[^ ^16^, ^18^, ^19^ ^]^ Mutations in ECM‐related genes have been linked to neurodevelopmental disorders such as schizophrenia and autism spectrum disorder^[^ ^19^ ^]^ and changes in ECM composition are associated with neurodegenerative diseases including Alzheimer's disease and multiple sclerosis.^[^ ^20^ ^]^ Vasculature also plays a multifaceted role in the central nervous system, including blood–brain barrier (BBB) formation,^[^ ^21^ ^]^ regulating and maintaining neural stem cell niches,^[^ ^22^, ^23^ ^]^ serving as a migratory scaffold for neural progenitor cells, and secreting ECM proteins such as laminins and integrins that act as stem cell anchors.^[^ ^22^ ^]^ To improve the sophistication of 3D in vitro models of brain development, it is critical to engineer platforms with tunable ECM properties and vascular networks.

Several strategies have been employed to incorporate ECM into organoid cultures, including embedding organoids in Matrigel,^[^ ^1^, ^24^, ^25^, ^26^, ^27^ ^]^ culturing organoids with decellularized brain tissues,^[^ ^28^, ^29^ ^]^ or using microfluidic devices containing ECM components.^[^ ^30^ ^]^ These approaches are generally difficult to scale and/or contain animal‐based biologicals that vary across batches. Vascular incorporation into organoids is another arena that has made considerable progress in recent years. Organoids can be implanted into a surrogate murine environment,^[^ ^31^, ^32^ ^]^ aggregated with endothelial cells (ECs),^[^ ^33^, ^34^ ^]^ or genetically engineered to overexpress vascular transcription factors.^[^ ^6^, ^35^ ^]^ In light of these advances, what remains a key ambition in the organoid field is to incorporate precisely engineered and reproducible vasculature with the capability of perfusion via physiologically relevant flow regimens.

Vascularization strategies for tissue engineered constructs include the addition of angiogenic factors,^[^ ^36^ ^]^ coculturing ECs with other cell types,^[^ ^37^ ^]^ and using scaffold‐based approaches.^[^ ^38^ ^]^ 3D bioprinting, a type of scaffold‐based approach, is an additive biomanufacturing technique that fabricates 3D structures with high spatial control. Biomaterials, often referred to as bioinks, are printed in a precise layer‐by‐layer format, and can encapsulate other materials, molecules, and/or cells to help mimic the native tissue environment.^[^ ^39^ ^]^ In the last decade, 3D bioprinting has been employed to incorporate vasculature into various engineered tissue constructs.^[^ ^40^, ^41^, ^42^ ^]^

An important parameter to consider when employing 3D bioprinting is selecting materials that recapitulate the native ECM of the desired tissue.^[^ ^43^ ^]^ The developing brain ECM is primarily comprised of glycosaminoglycans (hyaluronan, chondroitin sulfate, and heparin sulfate), proteoglycans, glycoproteins (laminins), and low amounts of fibrous proteins like collagen.^[^ ^44^, ^45^ ^]^ Furthermore, the brain microenvironment contains perineuronal nets—condensed chondroitin sulfate proteoglycan ECM structures that surround neuronal cell bodies and their processes.^[^ ^46^ ^]^ Previously reported biomaterials used in neural tissue engineering applications include alginate,^[^ ^47^, ^48^, ^49^, ^50^ ^]^ agarose,^[^ ^50^, ^51^, ^52^ ^]^ fibrinogen,^[^ ^49^, ^53^, ^54^ ^]^ collagen,^[^ ^51^, ^55^, ^56^ ^]^ gelatin,^[^ ^52^, ^57^, ^58^, ^59^ ^]^ and gelatin methacrylate (GelMA).^[^ ^48^, ^59^, ^60^, ^61^, ^62^ ^]^ GelMA is a particularly attractive option for 3D neural tissue engineering due to its inexpensive and facile fabrication process,^[^ ^63^ ^]^ its ability to support neuronal cultures,^[^ ^59^, ^60^ ^]^ its tunable mechanical properties,^[^ ^48^, ^64^ ^]^ and the ability to incorporate other materials or cells to create a more biomimetic environment.^[^ ^61^, ^65^, ^66^ ^]^ Currently, however, most 3D bioprinting applications involve directly printing bioinks laden with single‐cell suspensions, which require a high cell density and hinders the formation of 3D tissue architecture.^[^ ^43^ ^]^ Integrating the spatial control of 3D bioprinting and the scalability and biological relevance of cortical organoids offers the potential to generate a tunable, robust model of brain development.

Here, we report a 3D bioprinted GelMA microchanneled scaffold to be used as a platform for tunable organoid culture. We characterize the bioprinting fidelity and mechanical properties of the microchanneled scaffolds to ensure precision and reproducibility. We confirm the ability of bioprinted GelMA scaffolds to support long‐term culture of human cortical organoids (hCOs) without hindering neuroectodermal differentiation. Finally, we leverage the precisely engineered scaffold architecture to incorporate vasculature into developing organoid cultures. The ability of human umbilical vein endothelial cells (HUVECs) to vascularize the microchannels of bioprinted scaffolds and subsequently infiltrate encapsulated organoid is examined. Altogether, we validate that the 3D bioprinted GelMA scaffold is a highly robust, reproducible, and tunable in vitro platform that can increase the patterning capabilities of organoid cultures and address some of the key biological questions related to the human brain development.

## 2 Results

**Figure 1:**
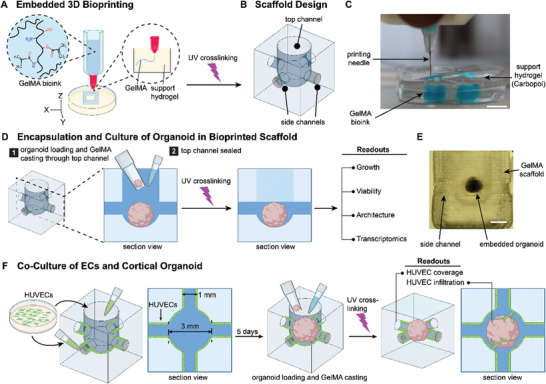
Schematic illustration of the study workflow. A) Three‐dimensional (3D) embedded bioprinting process of gelatin methacrylate (GelMA) into a support hydrogel. B) GelMA scaffold design consisting of a network of interconnected channels on the lateral side to incorporate vasculature and a top channel to manually load organoids into the scaffold. C) 3D embedded bioprinting, where GelMA (blue) is extruded from the printing needle into Carbopol (clear), the support hydrogel. Scale bar, 5 mm. D) Workflow to encapsulate and culture organoids in the bioprinted scaffold. 1) Organoids are manually loaded through the top channel, followed by GelMA casting and UV cross‐linking. 2) Following UV cross‐linking, the top channel is sealed, and organoids can be cultured. Readouts include assessing growth, viability, architecture, and transcriptomics. E) Bioprinted GelMA scaffold containing an encapsulated organoid, immediately after loading. Scale bar, 1 mm. F) Workflow to coculture endothelial cells (ECs) and cortical organoids. Human umbilical vein endothelial cells (HUVECs) are manually seeded through the top and side channels to achieve uniform endothelialization. Following 5 days of HUVEC culture, organoids are loaded through the top channel, GelMA is cast and UV cross‐linked, thus beginning the EC‐organoid coculture. Readouts include HUVEC coverage of the side channels and HUVEC infiltration into the organoid. Schematics created with BioRender.com.

We used embedded 3D bioprinting to generate our scaffolds because, as compared to direct (air) bioprinting, the embedded approach results in higher fidelity, particularly for scaffolds with complex geometries and relatively low stiffness (**Figure** **1**A).^[^ ^67^, ^68^, ^69^ ^]^ By printing within a support hydrogel, delicate architectural features exhibit improved accuracy since they are supported by the viscosity of the bath material. We selected 0.5% Carbopol as a support bath material due to its adequate shear thinning, biocompatibility, and printing fidelity properties.^[^ ^69^ ^]^ We selected a GelMA bioink at a concentration of 10% (w/v) based on its prior applications in neural tissue printing^[^ ^42^, ^70^ ^]^ and tunable mechanical properties at this concentration that could best reflect the developing nervous system ECM.^[^ ^64^ ^]^ Our scaffold design included a top channel for organoid loading and four interconnected side channels intended to introduce vascularization (Figure 1B and Movie S1, Supporting Information). We designed a top channel that was 3 mm in diameter to provide flexibility for the sizes (and ages) of loaded organoids, and side channels that were 1 mm in diameter to provide future access to perfusion systems. The scaffolds were designed so that organoids can be manually loaded through the top channel after printing the initial construct. Organoid loading is followed by GelMA casting and UV cross‐linking (320–365 nm) to seal the organoid in the center of the scaffold (Figure 1D,E). Importantly, the incorporation of side channels within this scaffold design affords the ability to coculture vascular cells with organoids by seeding a cell type of choice into the microchannels prior to organoid loading (Figure 1F). Ultimately, this tunable platform allowed us to investigate the role the microenvironment plays on cortical organoids and study organoid–endothelial interactions.

All GelMA used for bioprinting was fabricated in‐house. To ensure the consistency and reproducibility of our fabrication process, we performed a strict quality control procedure on randomly selected batches of GelMA that were synthesized in the lab. We used ^1^H NMR across randomly selected batches and found reproducible chemical signatures, including the presence of the acrylic proton of methacryloyl groups, methyl proton of the methacryloyl group, and reduction of the unmodified lysine methylene in gelatin control samples (Figure S1 and Equation 1, Supporting Information). These data suggest an average methyacryloyl substitution degree of 84.47% (SD = 0.75%). This uniform substitution degree, along with the precise reproducibility of the NMR peaks across batches, demonstrates consistency in the chemical structure of our fabricated GelMA. Next, we examined the GelMA batch consistency and reproducibility by reconstituting, casting, and cross‐linking randomly selected batches of synthesized GelMA at two distinct photo cross‐linking conditions (2.5 mW cm^−2^ or 20 mW cm^−2^ for 2 min). We then tested the elastic moduli of each condition to measure variability across all six batches (Table S1, Supporting Information, Equations (2), (3), (4), (5)).

**Figure 2:**
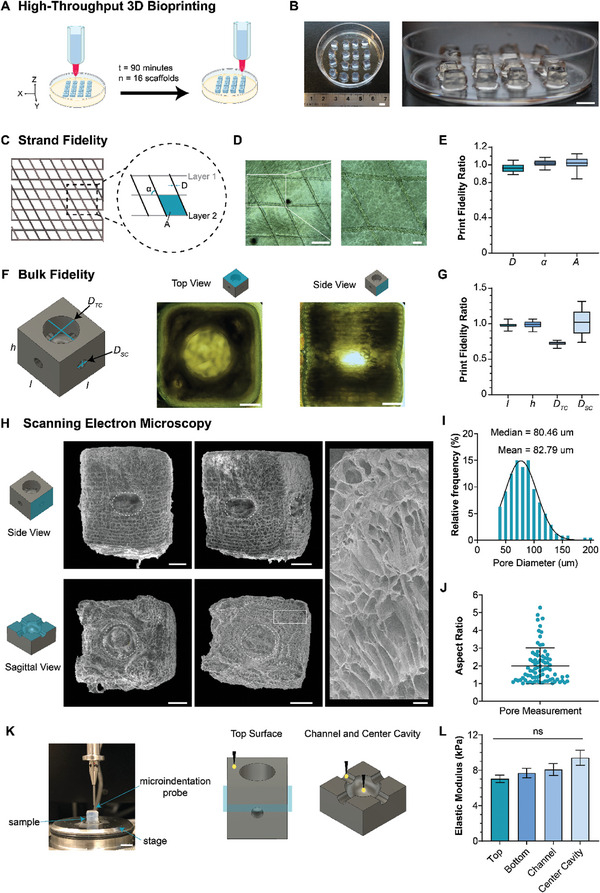
Characterization of printing fidelity, porosity, and mechanical properties of bioprinted scaffolds. A) Schematic depicting the high throughput capabilities of embedded 3D bioprinting, where 16 scaffolds can be printed in 90 min. B) Cross‐linked gelatin methacrylate (GelMA) scaffolds, following high throughput Three‐dimensional (3D) bioprinting (*n =* 16 scaffolds). Scale bar, 5 mm. C) Designed and D) printed two‐layer model used to assess 2D bioprinting fidelity. Scale bars, 1 mm (left) and 250 µm (right). E) Printing fidelity characterization of the strand diameter (*D*), interstrand angle (*α*), and interstrand area (*A*) for two‐layer model (*n =* 9 prints, 27 measurements). The mean and standard deviations for the strand fidelity ratios are 0.96 ± 0.05 (*D*), 1.02 ± 0.04 (*α*), and 1.02 ± 0.06 (*A*). F) Designed and printed GelMA scaffolds, highlighting the top channel (left) and side channel (right). Scale bar, 1 mm. G) Bulk fidelity characterization of the length (*l*), height (*h*), top channel diameter (*D*_TC_), and side channel diameter (*D*_SC_) of microchanneled GelMA scaffold (*n =* 12 scaffolds). The mean and standard deviations for the bulk fidelity ratios are 0.98 ± 0.03 (*l*), 0.99 ± 0.05 (*h*), 0.72 ± 0.03 (*D*_TC_), and 1.04 ± 0.18 (*D*_SC_). H) Scanning electron microscopy (SEM) of the microchanneled GelMA scaffolds. The side channels (top row), the sagittal view of the scaffold (bottom row), and magnified view of the middle of the scaffold (inset) are shown. Scale bar represents 1 mm for the low magnification images and 100 µm for the inset. I) Pore size quantification of GelMA microchanneled construct (*n =* 4 scaffolds, 60 measurements per scaffold). Gaussian distribution fit to the histogram of the pore size. Median = 80.46 µm, mean = 82.79 µm, SD = 27.57 µm. J) Quantification of the aspect ratio of the pores within the microchanneled scaffold (*n =* 4 scaffolds, 20 measurements/scaffold). Mean = 2.00, SD = 1.01. Data represents mean ± SD. K) Mechanical testing workflow where a microindentation probe is used to measure the elastic modulus of the top and bottom surfaces of the scaffold. Scaffolds are then cut in half and the elastic moduli of the side channels and central cavity are assessed. Scale bar, 5 mm. L) Elastic modulus of the top, bottom, side channel, and central cavity of cross‐linked GelMA scaffolds (*n =* 4 scaffolds). Elastic modulus values are 7.03 ± 0.42 kPa (top), 7.69 ± 0.53 kPa (bottom), 8.09 ± 0.67 kPa (side channels), and 9.42 ± 0.84 kPa (center cavity). All error bars represent ± standard error of the mean (SEM). No significant difference in elastic modulus of different areas of the microchanneled scaffold (one‐way analysis of variance (ANOVA) with Tukey's multiple comparisons test), *p* = 0.1757. Created in part with BioRender.com.

Having validated the GelMA fabrication protocol and optimized the GelMA and Carbopol printing parameters, we next assessed the fidelity, pore size, and mechanical properties of the bioprinted constructs. Using the embedded 3D bioprinting approach, we printed scaffolds at high throughput and reproducible scales (printing 16 scaffolds in 90 min) (**Figure** **2**A,B). We used strand diameter (*D*), strand angle (α), and interstrand area (*A*) of a two‐layer print to quantify printing fidelity (Figure 2C,D). Measured dimensions were normalized to the initial CAD model dimensions (Equations (6), (7), (8)). The ratios for strand diameter (0.96 ± 0.05 SD), strand angle (1.02 ± 0.04 SD), and interstrand area (1.02 ± 0.06 SD) were all close to the designed dimensions (approaching 1.00), indicating high 2D printing fidelity and minimal variation (Figure 2E). To assess the bulk (macroscale) fidelity of our microchanneled GelMA scaffolds, we measured scaffold length (*l*), height (*h*), top channel diameters (*D*_TC_), and side channel diameters (*D*_SC_) (Figure 2F, Equations (9), (10), (11), (12)). The ratios for scaffold length (0.98 ± 0.03) and height (0.99 ± 0.05) indicae that these printed features closely matched their intended design parameters (Figure 2G). The top channel diameter exhibited a slightly lower printing fidelity ratio at 0.72 ± 0.03; however, this did not hinder organoid loading since the top channel was designed with significant excess clearance. Measured side channel ratios of 1.04 ± 0.18 indicate that on average microchannels were printed at adequate levels of precision. We did observe higher variability in the fidelity of the side channel diameter, which may be a result of gravitational artifacts that cause the side channels to deform and become oblong. This variation was within the expected range of 3D bioprinting. Altogether, the printing fidelity measurements demonstrated that we can reproducibly and accurately print our microchanneled GelMA constructs.

To assess the ultra‐architecture of the microchanneled scaffolds and quantify pore size and homogeneity, we next performed scanning electron microscopy (SEM) of bioprinted scaffolds. Interestingly, we observed a radial alignment of pores toward the center of the scaffold, likely resulting from the concentric path of the printhead during the printing process (Figure 2H). SEM image analysis indicated a mean pore diameter of 82.79 ± 27.57 µm (SD) and median pore diameter of 80.46 µm (Figure 2I). This pore size range is sufficiently large to allow for oxygen exchange and cell migration.^[^ ^71^ ^]^ Aspect ratio measurements of the pore structure indicated an anisotropic pore shape (rectangles with aspect ratio of 2.00 ± 1.01, Figure 2J). These findings were consistent with prior reports demonstrating GelMA structures with a variety of pore shapes and sizes.^[^ ^72^ ^]^

We were next interested in understanding the mechanical properties of the cross‐linked microchanneled scaffold to characterize the microscale (niche) stiffness that the embedded organoids experience. Our goal was to generate stiffness values ranging between 1 and 10 kPa,^[^ ^73^, ^74^, ^75^ ^]^ as previously noted for the native neural tissues.^[^ ^43^ ^]^ Microindentation tests measured the elastic moduli for the top (7.03 ± 0.42 kPa) and bottom (7.69 ± 0.53 kPa) surfaces of the scaffold, as well as the side channels (8.09 ± 0.67 kPa) and center cavity (9.42 ± 0.84 kPa) where the organoid resides (Figure 2K,L, Equations (9), (10), (11), (12)). No significant differences were detected in the measured elastic moduli on various scaffold surfaces. Results were also consistent for various batches of GelMA bioink and bioprinting rounds.

**Figure 3:**
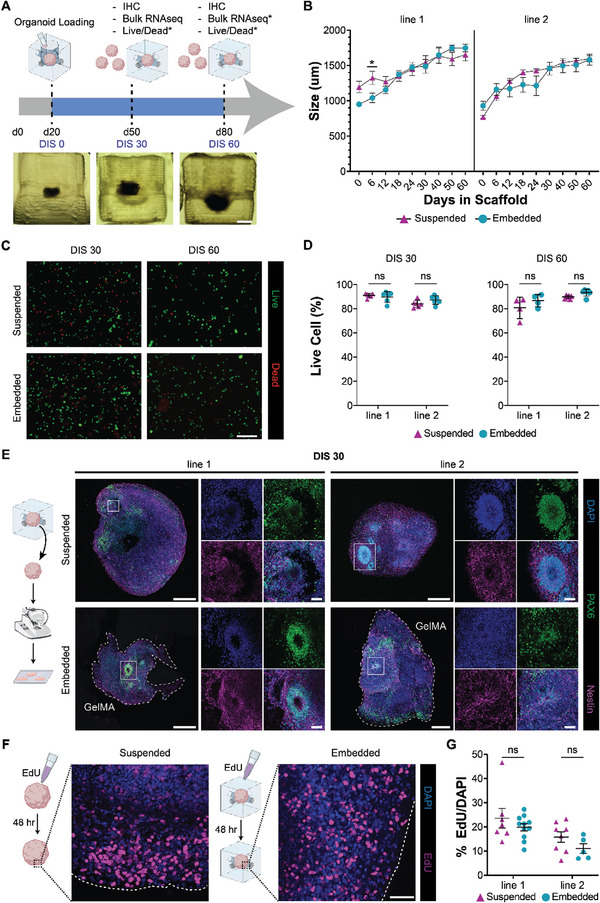
Assessment of the growth, viability, cytoarchitecture, and proliferation of embedded organoids compared to suspended controls. A) Schematic depicting study workflow. Day 20–25 organoids are manually embedded at a timepoint considered “days in scaffold” (DIS 0). Scaffolds are then cultured for 30 or 60 days. Asterisk (*) indicates that the assay was only performed on one differentiation per hiPSC line used, for a total of two lines. Scale bar, 1 mm. B) Quantification of organoid growth over 60 days of culture (*n* = 5–6 organoids per condition). Two‐way analysis of variance (ANOVA) performed with Tukey's multiple comparisons test reveals a significant size difference between suspended and embedded organoids only at DIS 6 (*p* = 0.044), validating that embedded organoids grow comparably to the suspended control. Error bars represent ± standard error of the mean (SEM). C) Live (calcein‐AM) and dead (ethidium homodimer‐1) quantification of dissociated suspended and embedded organoids at DIS 30 and 60. Scale bars, 100 µm. D) Quantification of the percentage of live cells at DIS 30 and DIS 60 across both cell lines. A total of *n* = 6 samples per condition were used for both cell lines at DIS 30. For DIS 60, *n* = 4 samples per condition used for line 1 and *n* = 6 samples per condition used for line 2. The percentage of live cell at DIS 30 are 89.74 ± 4.39% (line 1) and 87.31 ± 3.58% (line 2) for embedded samples, and 91.10 ± 1.79% (line 1) and 84.12 ± 3.06% (line 2) for suspended samples. The percentage of live cells at DIS 60 are at 86.70 ± 5.20% (line 1) and 93.37 ± 2.89% (line 2) for embedded samples, and 80.82 ± 8.23% (line 1) and 89.86 ± 1.35% (line 2) for suspended samples. Error bars represent ± SD. Two‐way ANOVA with Sidak's multiple comparisons test no significant difference in cell viability between suspended and embedded samples (DIS 30: line 1, *p* = 0.7370, line 2, *p* = 0.2131; DIS 60: line 1, *p* = 0.1918, line 2, *p* = 0.3935). E) IHC of suspended and embedded organoids reveals no difference in overall organoid cytoarchitecture. The stains are DAPI (blue), PAX6 (green), and Nestin (magenta). Scale bars are 200 and 50 µm (magnified images). F) Immunohistochemistry (IHC) on sections of suspended and embedded organoids exposed to a 48‐h pulse of EdU. Stains represent DAPI (blue) and EdU (magenta). Scale bar, 50 µm. G) Quantification of the percentage of EdU positive cells normalized to the number of DAPI cells. A total of *n =* 4 samples per condition per cell line were used. A two‐way ANOVA with Sidak's multiple comparisons test was used and demonstrates no significant difference in the percentage of EdU cells between the two culture conditions (line 1: *p* = 0.4784, line 2: *p* = 0.4163). Error bars represent ± SEM. ^*^ *p* < 0.05, ^**^ *p* < 0.01, ^***^ *p* < 0.001. Schematics created with BioRender.com.

Having characterized the micro‐ and macroscale features of the bioprinted scaffolds, we next evaluated the feasibility of culturing organoids embedded within the 3D GelMA constructs. We formed and cultured hCOs as previously described.^[^ ^3^ ^]^ We performed these experiments across three hiPSC lines, and five separate differentiations. We decided to begin organoid loading into scaffolds on day 20 of differentiation because this represents a timepoint at which organoids enter a rapid growth phase and are also highly sensitive to extrinsic cues for patterning and differentiation.^[^ ^76^, ^77^ ^]^ We loaded a single 20‐day hCO through the top channel of each bioprinted construct and positioned them to rest in the middle of the scaffold, on top of the central cavity. We then cast GelMA through the top channel and UV cross‐link to seal the organoid within the scaffold. Embedded organoids are not fully encased in GelMA, as the microchannels in the scaffold are not sealed. After organoid loading into scaffold, we designated this time point as “days in scaffold” (DIS) 0, and cultured for 30 or 60 days while we monitored the growth, viability, cytoarchitecture, and proliferation compared to suspended organoid controls from identical differentiations (**Figure** **3**A). Both suspended and embedded organoids were cultured statically. Organoids embedded in the scaffolds demonstrated no overall growth differences compared to suspended controls across cell lines 1 and 2 (*p* = 0.1844) (Figure 3B). This remained true for up to 60 days in culture (DIS 60), suggesting that the microchanneled scaffolds do not significantly hinder the physical growth of organoids.

To next assess the viability of embedded organoids, we performed a live/dead assay on single cell suspensions from suspended (control) and embedded organoids (Figure 3C). Across both hiPSC lines and at both DIS 30 and 60 time points, we observed no declines in cell viability for organoids cultured within the GelMA scaffolds. The live cells percentage of embedded organoids at DIS 30 was 89.74 ± 4.39% (line 1) and 87.31 ± 3.58% (line 2). The viability of suspended organoids at DIS 30 was 91.10 ± 1.79% (line 1) and 84.12 ± 3.06% (line 2). At DIS 60, the viability of embedded organoids remained consistent at 86.70 ± 5.20% (line 1) and 93.37 ± 2.89% (line 2). The live cell percentage of suspended organoids at DIS 60 was 80.82 ± 8.23% (line 1) and 89.86 ± 1.35% (line 2) at DIS 60. There are no significant differences between the suspended and embedded cell viability at each timepoint (Figure 3D).

hCOs have been cultured in a variety of matrices, with Matrigel among the most prevalent.^[^ ^1^, ^24^, ^25^, ^26^, ^27^ ^]^ Therefore, we next compared the behavior of organoids embedded in our GelMA scaffold versus traditional Matrigel droplets. In this scenario we did observe a small growth advantage in Matrigel conditions after 12 days with the Matrigel‐embedded hCOs reaching about 50% larger size (*p* < 0.04, *n* = 1 hiPSC line) (Figure S2A, Supporting Information). However, viability of hCOs in either matrix was unchanged (live cell percentages: GelMA 85.29 ± 3.99%, Matrigel 78.95 ± 6.27%, and suspended 83.12 ± 2.98%, p = 0.30) (Figure S2B, Supporting Information). One caveat of the live/dead viability assay is the possibility that dead or dying cells are lost during dissociation and thus provide a misleading interpretation of overall cell health. Therefore, we also performed TUNEL staining on sectioned suspended, Matrigel‐encapsulated, and GelMA embedded organoids to test rates of cell death endogenously. We find the percentage of TUNEL+ cells are as follows: 9.18 ± 2.71% for suspended, 13.63 ± 9.92% for embedded, and 7.65 ± 6.04% for Matrigel encapsulated hCOs. These numbers were consistent with the dissociated live/dead assay and revealed no significant differences in cell viability across all conditions (Figure S2C,D, Supporting Information). To further elucidate potential effects between GelMA and Matrigel embedding, we performed bulk RNA sequencing on DIS 30 organoids from both conditions and found only a small number of non‐specific changes (14 upregulated genes in embedded samples and 91 upregulated genes in Matrigel samples, Table S2, Supporting Information), mostly related to collagen gene regulation in the presence of Matrigel (Figure S2E, Supporting Information).

To investigate if the cytoarchitecture of embedded organoids was altered by culturing in the GelMA scaffold, we next sectioned and immunostained organoids for canonical differentiation markers. The overall cytoarchitecture of embedded organoids was highly reminiscent of typical suspended organoids, including the regular presence of neural rosettes (highlighted by PAX6 and NESTIN stains, Figure 3E). We hypothesized that one consequence of continued growth of organoids within the bioprinted scaffold could be stunted proliferation. Therefore, to test the degree of active cell division within embedded organoids, we added EdU to the culture media of suspended and embedded organoids for a 48‐h pulse at DIS 28 and then quantified the percentage of EdU positive progeny (Figure 3F). We observed no significant difference in overall EdU positivity (normalized to DAPI) in either cell line (*p* = 0.4784 for line 1 and *p* = 0.4163 for line 2, Figure 3G), suggesting that scaffold environment does not hinder organoid organization or proliferation.

**Figure 4:**
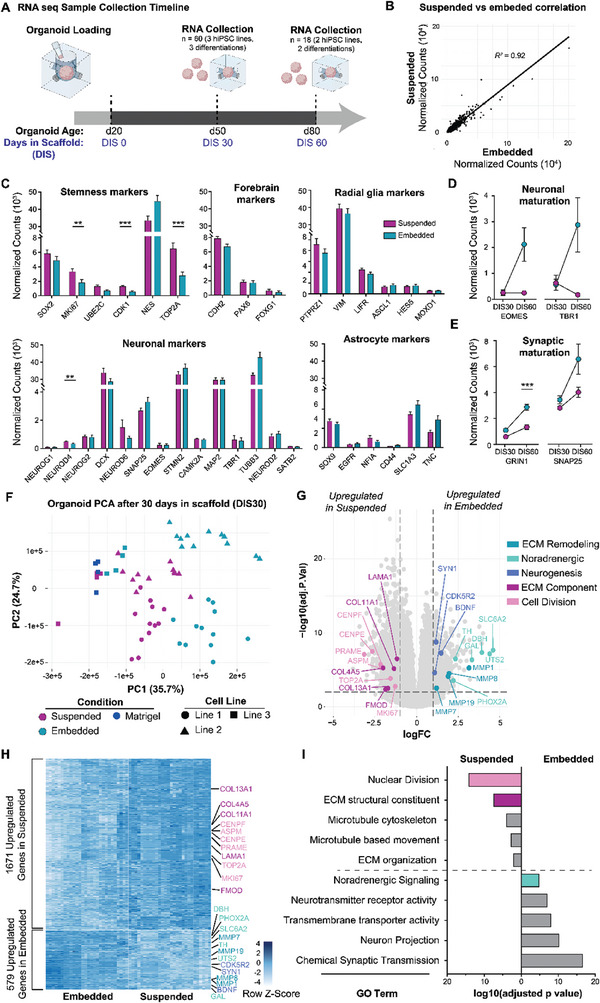
Transcriptomic assessment of embedded and suspended organoids at “days in scaffold” (DIS 30). A) Schematic depicting the RNA‐seq sample collection timeline. A total of *n =* 60 samples were sequenced at DIS 30 (*n =* 28 embedded samples*, n =* 28 suspended samples*, n =* 4 Matrigel embedded samples) and *n =* 9 suspended and *n =* 9 embedded samples were sequenced at DIS 60. DIS 30 samples include organoids from three different hiPSC lines and three different differentiations that are pooled together for analyses. DIS 60 samples include organoids from two different hiPSC lines that are pooled together for analyses. B) Normalized counts of suspended and embedded samples at DIS 30 reveals that there is a correlation in gene expression across the two culture conditions (*R* ^2^ = 0.92). C) Comparison of the expression (normalized counts) of different neuroectodermal related genes. ^*^ *p*_adjusted < 0.05, ^**^ *p*_adjusted < 0.01, ^***^ *p*_adjusted < 0.001. Significance determined by edgeR and Bonferroni correction. Error bars represent ± standard error of the mean (SEM). D) Normalized counts of neuronal maturation genes, EOMES and TBR1, and E) synaptic maturation genes, GRIN1 and SNAP25 from DIS 30 to DIS 60. GRIN1 is upregulated in embedded DIS 60 samples according to differential expression analysis (*p* < 0.001). Error bars represent ± SEM. F) Principal component analysis (PCA) reveals that the primary variation is a result of culture condition (PC1 = 35.7%), followed by cell line (PC2 = 24.7%). G) Volcano plot highlighting the upregulated genes in the suspended and embedded samples. Cutoffs are logFC ± 1 and adjusted *p* value < 0.01. H) Heatmap demonstrating the differentially expressed genes and highlighting genes involved in matrix remodeling, noradrenergic signaling, neurogenesis, extracellular matrix (ECM) components, and cell division. I) Gene ontology (GO) analysis using upregulated genes for suspended and embedded samples. Schematics created with BioRender.com.

To more holistically assess potential changes in organoid growth upon embedding, we next performed bulk RNA sequencing of organoids grown in suspension or within microchanneled scaffolds for 30 or 60 days (**Figures** **4**A and S3A, Supporting Information). Overall, we observed high transcriptional correlation between the suspended and embedded samples at both DIS 30 (*R* ^2^ = 0.92) (Figure 4B) and DIS 60 (*R* ^2^ = 0.92) (Figure S3B, Supporting Information), again highlighting the general health and differentiation trajectory that is maintained in this platform. To specifically investigate the degree of neuroectodermal differentiation, we curated a signature of canonical dorsal forebrain developmental genes and assessed their differential gene expression (DGE) between suspended and embedded samples. We observed nearly identical expression levels between the two culture conditions for markers of stemness (3 of 6), forebrain differentiation (3 of 3), radial glia identity (6 of 6), neuronal identity (13 of 14), and astrogenesis (6 of 6) (Figure 4C). This trend was also present at DIS 60 (Figure S3C, Supporting Information). Notably, we did observe that transcript abundances of MKI67, CDK1, and TOP2A, markers of proliferation, were significantly lower in embedded organoids at DIS 30, despite our previous observations about the overall organoid size and EdU proliferation metrics.

To ask whether neural maturation may be affected in the GelMA construct, we next selected several markers of neuronal maturation (EOMES and TBR1) and synaptic maturation (GRIN1 and SNAP25) to assess dynamic expression changes from DIS 30 to 60 (Figure 4D,E). For each of these genes we observed either similar increases in normalized counts across both suspended and embedded samples, or even higher expression in embedded samples (NMDA receptor, GRIN1 *p* < 0.001). It has also previously been reported that typical organoid culture conditions provoke an upregulation of cell stress markers.^[^ ^78^ ^]^ Bhaduri et al. identified that the ER stress genes ARCN1 and GORASP2 and the glycolysis gene PGK1 are particularly sensitive indicators of organoid stress as compared to primary fetal cells. We therefore used this curated “stress” list to assess expression changes in embedded and suspended organoid between DIS 30 to 60. We observed nearly identical expression levels across all three markers at each timepoints (Figure S4, Supporting Information).

Overall, we did observe several notable transcriptomic differences between suspended and embedded organoids, as highlighted by these groups separating clearly by principal component analysis (PCA) at DIS 30 and DIS 60 (Figure 4F, Figure S3D, Supporting Information). At DIS 30, we found a total of 2250 differentially expressed genes (1671 upregulated in suspended samples and 579 upregulated in embedded samples, Table S3, Supporting Information). These genes revealed a general upregulation of ECM remodeling, noradrenergic signaling, and neurogenesis related genes and downregulation of ECM component and cell division genes in the embedded samples (Figure 4G,H). Gene ontology (GO) analysis at DIS 30 further demonstrated an upregulation of neurotransmitter receptor activity, chemical synaptic signaling, and neuron projections and a downregulation of spindle organization, ECM organization, and microtubule cytoskeleton in embedded samples (Figure 4G, Table S4, Supporting Information).

At DIS 60, we found a total of 1281 differentially expressed genes (921 upregulated in suspended samples and 360 upregulated in embedded samples, Table S5, Supporting Information). These included the upregulation of ECM remodeling and neuronal projection genes and a downregulation in genes involved in cilium and microtubules in embedded samples. Genes involved in neurogenesis exhibited mixed patterns of up/downregulation in both suspended and embedded samples (Figure S3E,F, Supporting Information). At DIS 60, the GO analysis revealed an upregulation of synapse, chemical synaptic transmission, and monoatomic ion channel activity, and a downregulation of dynein binding, microtubule‐based movement, and cilium organization in embedded samples (Figure S3G and Table S6, Supporting Information).

The GO analysis revealed an upregulation of synaptic signaling related genes in embedded samples at DIS 30 and 60. A recent study reported that 3D cultures, such as cortical organoids, upregulate pathways related to chemical synaptic signaling as compared to 2D monocultures.^[^ ^79^ ^]^ To test whether scaffold‐embedded organoids were exacerbating this particular molecular signature, we projected this curated gene list of chemical synaptic signaling (68 genes) onto our differential expression plots between embedded and suspended organoids. We found a clear upregulation trend in scaffold embedded samples at both DIS 30 and DIS 60 (Figure S5A,B, Supporting Information). Altogether, the transcriptomic data indicated that the scaffold environment does not significantly alter neuroectodermal differentiation. Embedded organoids are capable of sensing, responding, and remodeling the GelMA matrix and embedded organoids actually upregulate synaptic machinery proteins.

Having demonstrated the capacity of the GelMA scaffolds to support long term culture of embedded organoids, we next pursued whether we could engraft ECs to form vascular structures within bioprinted scaffolds. HUVECs were selected in this study as they are as an extensively studied vascular cell type to characterize angiogenesis,^[^ ^80^ ^]^ have intrinsic tube formation capabilities,^[^ ^33^ ^]^ have previously been used to vascularize organoids,^[^ ^33^ ^]^ and have been engineered to constitutively express green fluorescent protein (GFP), which facilitates visualization. Before culturing HUVECs in the bioprinted scaffolds, we needed to define optimal media conditions that could allow for EC growth and also support organoid development. Therefore, we tested a variety of media compositions on 2D HUVEC cultures and measured cell viability via an AlamarBlue assay (Figure S6A,B, Supporting Information). Ultimately, we found that allowing HUVECs to culture in their typical media for a period of at least 3 days before switching to a 50/50 mix with organoid media results in enhanced viability (day 3: *p* < 0.0001, day 6: *p* < 0.0001, Figure S6C, Supporting Information). This strategy allowed for a period of HUVEC‐only seeding in the bioprinted scaffolds prior to the addition of organoids.

**Figure 5:**
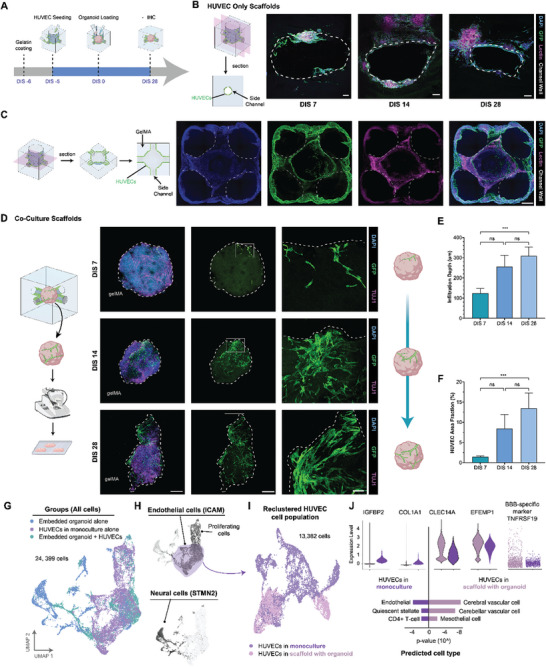
Coculture of human umbilical vein endothelial cells (HUVECs) and cortical organoids within microchanneled gelatin methacrylate (GelMA) scaffolds. A) Schematic of the coculture workflow. GelMA microchanneled scaffolds are coated with gelatin for 24 h and then seeded with HUVECs. HUVECs are cultured for 5 days and then day 25–30 organoids are manually loaded through the top channel “days in scaffold” (DIS 0). Scaffolds are then cultured for up to 4 weeks (DIS 28). B) HUVEC only scaffolds are sectioned coronally to observe the coverage of the microchannels. There is an increase in HUVEC coverage of the microchannel from DIS 7 to DIS 28, shown by DAPI (blue), GFP (green), and Lectin (magenta). The white dashed line depicts the channel wall. Scale bar, 100 µm. C) Whole mount imaging of DIS 35 HUVEC only bioprinted scaffolds, preserving the four interconnected side channels, demonstrating almost complete coverage of the microchanneled surface. The stains are DAPI (blue), GFP (green), and Lectin (magenta). Channel walls are highlighted by the dashed white lines. Scale bar, 1000 µm. D) Immunohistochemistry (IHC) of cocultured organoids, which reveals progressive HUVEC infiltration into the organoid. Organoids are manually removed from the scaffold, sectioned, and stained with DAPI (blue), GFP (green), and TUJ1 (magenta). Scale bars are 200 µm for the first two columns and 50 µm for the magnified third column. Quantification of E) HUVEC infiltration depth into the organoid and F) HUVEC area fraction following 7, 14, or 28 days of coculture. Stained sections from two cell lines are pooled and analyzed. A total of *n* = 5 samples per time point were used. One‐way analysis of variance (ANOVA) with Tukey's multiple comparisons test demonstrates a significant difference in HUVEC infiltration depth (*p* = 0.0005) and area coverage (*p* = 0.0004) from DIS 7 to DIS 28. ^*^ *p* < 0.05, ^**^ *p* < 0.01, ^***^ *p* < 0.001. All error bars represent ± standard error of the mean (SEM). G) Uniform Manifold Approximation and Projection (UMAP) plot of scRNAseq data from embedded only organoids (*n* = 7 scaffolds, 5839 cells), HUVEC monoculture (9825 cells), and embedded organoids cultured with HUVECs (*n* = 12 scaffolds, 8735 cells) for a total of 24 399 cells. One hiPSC line was used. H) Cell type specific clusters, depicted by ICAM2 (ECs) and STMN2 (neural lineage). I) UMAP of reclusterd EC (*n* = 13 382 cells), revealing a separation based on the presence or absence of human cortical organoids (hCOs). J) Differential gene expression (DGE) demonstrates a shift toward cerebral vascular identity in HUVECs cultured with hCOs compared to monoculture HUVECs and increased expression of the blood–brain barrier (BBB) specific marker TNFRSF19. Schematics created with BioRender.com.

Having confirmed the optimal media to culture the ECs, we began by adding HUVECs alone to scaffolds so that we could assess the ability of these cells to form luminal coverage of the bioprinted channels. HUVECs were manually loaded through the top and side channels of the bioprinted constructs and allowed to culture for 5 days before manually seeding organoids (**Figure** **5**A). To first assess luminal coverage of seeded HUVECs, we sectioned scaffolds coronally (to preserve microchannel integrity) at either 7‐, 14‐, or 28‐days post‐seeding. We observed progressive HUVEC coverage of the channel walls from DIS 7 to DIS 28. Most channels were lined with HUVECs by DIS 14 (Figure 5B). We next assessed HUVEC coverage of the four interconnected side channels. Whole‐mount imaging of 3D cultured constructs illustrated almost complete coverage of the microchannels surface by the HUVECs at DIS 35 (Figure 5C). Next, we repeated these culture conditions, but added organoids (day 25–30) 5 days after the constructs were initially seeded with HUVECs. We allowed the organoid‐HUVEC coculture to develop for either 7, 14, or 28 days before assessing vascular infiltration into each organoid. To accurately quantify HUVEC infiltration into the organoid parenchyma, organoids were manually removed from the scaffold, sectioned, and stained for both endothelial and organoid markers (Figure 5D). The first metric we used to quantify the extent of HUVEC‐organoid interactions was infiltration distance, defined by the farthest tangential distance HUVECs traveled into the organoid from the border. We observed a progressive increase in HUVEC infiltration depth over the 28‐day culture, with the most statistically significant increase occurring between DIS 7 and DIS 28 (*p* = 0.0005, Figure 5E). As an alternative quantification approach to assess HUVEC infiltration, we also measured the area fraction of HUVECs within each organoid section. Similarly, we observed a persistent increase in HUVEC coverage over the 28‐day culture period, with a statistically significant increase between DIS 7 and DIS 28 (*p* = 0.0004, Figure 5F).

We next performed single cell RNA sequencing (scRNAseq) on 24,399 total cells from embedded only organoids (*n* = 7 scaffolds, 5839 cells), HUVEC monoculture (9825 cells), and embedded organoids cultured with HUVECs (*n* = 12 scaffolds, 8735 cells) (Figure 5G). All cells clustered into three predictable groups belonging to either endothelial (ICAM+), neuroectodermal (STMN2+), or proliferating (TOP2A+) identities (Figure 5H). Neuronal populations were intermixed between embedded only samples and those also containing HUVECs. We were next interested in understanding if HUVECs interacting with the GelMA‐encapsulated organoid resulted in transcriptomic changes compared to HUVEC monocultures. Therefore, we specifically subset the EC population (*n* = 13 382 cells) and reclustered this group, which revealed distinct separation based upon the presence or absence of hCOs in the culture condition (Figure 2I). Differential expression of monoculture HUVECs versus those cocultured with hCOs in the GelMA scaffold (Table S7, Supporting Information) revealed several transcriptomic changes indicative of a neural environment. When these genes were used to predict cell identity, we detected a shift toward cerebral vascular identity in HUVECs cultured in the GelMA scaffold with hCOs (Figure 5J). While not statistically significant, we did also observe an increase in the expression of the BBB‐specific marker TNFRSF19^[^ ^81^ ^]^ in our HUVECs cultured in GelMA scaffolds containing an organoid compared to the monoculture controls (Figure 5J). Altogether, these results indicate the ability of our 3D bioprinted GelMA platform to culture organoids and integrate multiple cell types with the goal of incorporating vasculature into cortical organoids using a system that has defined vascular structures.

## 3 Discussion

Currently, the vast majority of applied 3D bioprinting approaches in the field of neuroscience involve direct printing of differentiated cells into complex architectures.^[^ ^59^, ^82^, ^83^, ^84^, ^85^ ^]^ In this study, we instead use bioprinting capabilities to shape the microenvironment of preformed 3D in vitro organoid structures. The advantage of this approach is that it maintains the features of self‐organization and cell–cell communication that are inherently present in organoid cultures. Such features are difficult to replicate when cells are printed directly within bioinks. Using embedded 3D bioprinting, we can reproducibly print up to 16 microchanneled scaffold at once with high fidelity and scalability and can culture organoids in the scaffold for up to 60 days with no detrimental effects.

Mechanical properties of the tissue microenvironment have profound effects on cell responses.^[^ ^73^ ^]^ However, current 3D in vitro brain models rarely have the capacity to tune these physiomechanical parameters. Only a small number of studies have characterized the stiffness properties of the developing human fetal cortex and predict a range between 0.1 to 10 kPa,^[^ ^86^ ^]^ which was our benchmark for these neural cultures.^[^ ^73^, ^74^, ^75^ ^]^ In this work, our scaffolds ranged from 7 to 9 kPa, depending on the region of the scaffold that we tested. These values are within the high range of expected values for the developing human brain, although those numbers are technically challenging to confirm. The tradeoff between a soft scaffold (closer to 0.1–3kPA) and improved printing fidelity and higher stiffness levels is a balance that could be tested and optimized in future studies. Additionally, the mechanical environment of the brain is not static, and factors such as sex,^[^ ^87^ ^]^ aging,^[^ ^87^ ^]^ and disease pathology^[^ ^88^, ^89^ ^]^ are known to influence the physical parameters of the parenchymal space. Furthermore, the stiffness of the developing skull (1–50 MPa)^[^ ^90^ ^]^ is orders of magnitude higher than that of the brain ECM, but little is known how this physical constraint influences neurodevelopmental processes. Given the photosensitivity of GelMA, our bioprinted scaffolds offer a unique opportunity to rapidly test varying stiffness conditions to elucidate the relationship between physical–mechanical properties on developing neural cells.

Pore size can also influence cell migration and nutrient/oxygen exchange in bulk hydrogels.^[^ ^71^ ^]^ Numerous studies have created hydrogels with mean pore sizes ranging from 11 to 500 µm and observed proliferation and migration of encapsulated cells. With the goal of incorporating vasculature, we aimed for a minimum pore size of approximately 30–40 µm to ensure efficient exchange of metabolic components and EC migration.^[^ ^91^, ^92^, ^93^, ^94^ ^]^ The mean pore size within our bioprinted scaffold was 82.79 ± 27.57 µm (SD), which we believe will be sufficient for nutrient and oxygen exchange to embedded hCOs. Future work should focus on varying the pore size and elucidating the effect on EC migration and infiltration into the organoid. This can be achieved by varying the GelMA concentration or UV cross‐linking time/intensity. We also observed that the pores in the center of our scaffold were irregular in size and shape, as previously reported.^[^ ^72^ ^]^ Such asymmetric structural features could result in anisotropic mechanical properties of the scaffold. The brain, itself, is well characterized for its anisotropic biomechanical characteristics, which are largely a consequence of the organization of axonal tracts.^[^ ^95^ ^]^ The degree and directionality of anisotropy within neural cultures could have future implications in the development of axonal outgrowth or assembloid models in which regional organoids are fused and encouraged to establish new neuronal connections.

All organoid cultures used in this study were cultured statically. One potential concern is that the GelMA scaffold could hinder nutrient and gas exchange due to increasing diffusion distance. As demonstrated by our GelMA pore assessment, these scaffolds are quite porous, which we believe provides sufficient nutrient, gas, and waste exchange to support embedded hCO culture. This claim is further validated by our observation of no quantifiable differences between organoid growth and cell viability between suspended and embedded hCOs. One of our interesting observations was that we consistently saw evidence of decreased cell proliferating genes in GelMA‐embedded organoids, although their proliferative capacity and overall gross size did not appear substantively different. One possible explanation for this discrepancy is that embedded organoids are globally more prone toward differentiation, and thus as a whole exhibit decreased proliferative signature while maintaining a similar pool of actively dividing cells.

Our transcriptomic analyses also demonstrate a downregulation of ECM component and ECM organization genes in the embedded organoids. We hypothesize that this is a response to cellular sensing of abundant ECM presence within the scaffold‐embedded organoids. In fact, we observe an upregulation in ECM remodeling in embedded samples at DIS 30 and 60. Previous studies have demonstrated that 3D neural cultures generate their own ECM,^[^ ^79^ ^]^ which could explain the upregulation observed for these genes in the suspended cortical organoid samples. The transcriptomic analysis also revealed an upregulation of synaptic and noradrenergic signaling related genes in embedded samples. We believe that the upregulation of signaling markers could be a result of cells embedded within the GelMA scaffold focusing their transcriptional programming on ECM remodeling and neuronal specification rather than ECM generation. This is consistent with literature reports that ECM remodeling provides morphogenic cues that ultimately dictate cell proliferation, migration, and differentiation.^[^ ^96^, ^97^, ^98^ ^]^ Alternatively, this could indicate that cells grown within a physically stiffer ECM environment are pushed toward a more hindbrain differentiation fate. In fact, the relationship between the mechanical properties of the ECM and neural cell differentiation is highly reported in literature.^[^ ^99^, ^100^, ^101^, ^102^ ^]^ One such study demonstrates that neural progenitor cells cultured on methyacrylamide chitosan are favored to differentiate into neurons at stiffnesses of 1 kPa but are favored to differentiate into oligodendrocytes at scaffold stiffnesses greater than 7 kPa.^[^ ^99^ ^]^

As a control in this study, we compared Matrigel droplet encapsulated organoids to our GelMA embedded organoids. While we did observe a significant decrease in organoid growth in our embedded organoids compared to Matrigel encapsulated and suspended organoids at DIS 12, we believe this may be a line specific feature, as we did not observe this trend in two other hiPSC lines we tested. Despite the decrease in organoid growth, we found similar cell viability via our live/dead assay and TUNEL staining across the three culture conditions. Given the batch‐to‐batch variability and cost of Matrigel, a cost effective and highly reproducible matrix material would serve as an attractive alternative for organoid culture. In addition to demonstrating no significant changes in cell viability or the transcriptomic profile of GelMA embedded hCOs compared to Matrigel encapsulated or suspended hCO controls, we show that our facile fabrication protocol results in GelMA with consistent chemical structure and mechanical properties via NMR and microindentation. Therefore, we propose that GelMA might be considered in future studies as a chemically defined alternative organoid culturing matrix. While several groups have used GelMA to support organoid cultures, simple casting strategies remain limited by a lack of architectural control.^[^ ^103^ ^]^ Therefore, the ability to tune the architecture of the extracellular matrix is imperative as we move toward more complex 3D in vitro model systems. 3D bioprinting the GelMA hydrogel provides one such approach.

While 3D bioprinted organoid scaffolds have many advantages, there are several caveats to the system presented here. In these studies, we use UV cross‐linking to seal embedded organoids within the bioprinted GelMA scaffold, which may result in small amounts of UV exposure to the organoid cells. Transitioning our 3D bioprinting paradigm to a visible light photo cross‐linker, such as lithium‐acylphosphinate (LAP), could mitigate any potential damage because of UV exposure.^[^ ^104^ ^]^ This would ultimately require the optimization of cross‐linking length and intensity to achieve suitable scaffold fidelity and mechanical properties. As previously mentioned, the brain ECM is primarily comprised of glycosaminoglycans, proteoglycans, glycoproteins, and low amounts of fibrous proteins, such as collagen.^[^ ^44^ ^]^ GelMA, our selected bioink, is comprised of denatured collagen, which is not typically abundant in the brain. While we do not observe any negative effects on organoid culture in the GelMA scaffold, future work should focus on the addition of other biomimetic ECM components, such as hyaluronic acid or heparin sulfate to better recapitulate the brain microenvironment. Other groups have worked on functionalizing GelMA for neural tissue engineering applications by incorporating additional components to create a more instructive microenvironment. Examples include adding dopamine to enhance neural stem cell differentiation^[^ ^60^ ^]^ and mixing GelMA with glycidyl methacrylate‐hyaluronic acid.^[^ ^62^ ^]^ Further, varying concentrations of methacrylated alginate were added to GelMA bioinks to elucidate the relationship between ECM stiffness and neuroblastoma cell cluster dynamics.^[^ ^48^ ^]^ Adding the intrinsically conductive polymer poly(3,4‐ethylenedioxythiophene):polystyrene sulfonate has also been evaluated to electrically stimulate encapsulated dorsal root ganglion cells.^[^ ^105^ ^]^

Finally, our platform is capable of robustly supporting the coculture of HUVECs and organoids. While it has been shown that HUVECs adopt a brain EC‐like morphology when cultured with organoids, they may not represent the most ideal vascular cell source to investigate EC‐neuroepithelial interactions due to expression differences compared to human brain microvascular ECs (HBMECs).^[^ ^33^ ^]^ In the future, it will be imperative to move toward more physiologically relevant cell sources, such as iPSC‐derived ECs, HBMECs, and pericytes. Additionally, future work aims to incorporate perfusion, which will allow us to characterize the functionality of the vascular cells and assess the impact of the infiltrating ECs on organoid differentiation from the neural perspective. The addition of perfusion will also allow us to elucidate the role of flow in EC differentiation, EC luminal compartment formation and barrier function, and EC‐organoid interactions. Ultimately, the addition of flow to this work will enhance our ability to better model the dynamic processes involved in human brain development and incorporate functional vasculature into organoid model systems.

## 4 Conclusion

Overall, we demonstrate that we can create reproducible 3D bioprinted GelMA scaffolds that support the long‐term culture of cortical organoids and the coculture of ECs and hCOs. Through viability and transcriptomic characterization, we show that the GelMA scaffolds do not hinder neuroectodermal differentiation or organoid health. Furthermore, we validate that we can study EC‐organoid interactions and observe progressive EC infiltration into the cortical organoid. Moving forward, this platform can readily be tuned to incorporate additional ECM components, incorporate flow forces within EC‐organoid interactions, and include additional cell types. Altogether, this platform provides a new toolbox to create more complex and physiologically relevant models of human brain development and function.

## 5 Experimental Section

### Culture of hiPSCs

Two male (8858.3^[^ ^5^ ^]^ and C3.1 (Coyne Scientific)) and two female (1363.1^[^ ^5^ ^]^ and C4.1 (Coyne Scientific)) hiPSC lines were used in this study to form hCO cultures. A total of 1363.1, 8858.3 and C4.1 were used for the viability studies and 8858.3 and C3.1 were used for HUVEC and hCO cocultures. hiPSCs were cultured on vitronectin coated plates (ThermoFisher, Cat. A14700) and maintained in Essential 8 media (ThermoFisher, Cat. A1517001) in tissue culture incubators (37 °C with 5% CO_2_). Cultures were tested for and maintained mycoplasma free.

### Generations of hCS from hiPSCs

When hiPS cells reach 80–90% confluency, they were formed via the AggreWell or Dispase method.^[^ ^3^, ^24^ ^]^ Newly formed organoids were transferred to an ultra‐low attachment plastic dish (VWR, Cat. 76441–543) and cultured in Essential 8 media supplemented with ROCK inhibitor Y‐27632 (10 × 10^−6^ m) (R&D Systems, Cat. 1254). This is considered day 0 and the media is not changed for 48 h. The subsequent neural induction and differentiation timeline is the same. From day 2 to day 5, young spheroids were fed daily with Neural Induction Media supplemented with 5 × 10^−6^ m Dorsomorphin (DM) (Sigma Aldrich, Cat. P5499), 10 × 10^−6^ m SB‐431542 (Sigma Aldrich, Cat. S4317), and 20 ng mL^−1^ FGF2 (R&D Systems, Cat. 233‐FB‐01 M). On day 6, the media was replaced with neural media (Thermo Fischer Scientific, Cat. 10888022 or US Biological Life Sciences, Cat. N1020‐02) supplemented with EGF (20 ng mL^−1^) (R&D Systems, Cat. 236‐EG‐01 M) and FGF2 (20 ng mL^−1^). Media changes occur daily for the first 10 days (6–15) and then every other day from days 16 to 24. From day 25 to 43, organoids were fed every other day with neural media and supplemented with 20 mg mL^−1^ BDNF (PeproTech, Cat. 450‐02) and 20 mg mL^−1^ NT‐3 (PeproTech, Cat. 450‐03) to promote differentiation of the progenitors. After day 43, organoids were fed with only neural media every 3–4 days. All cultures are maintained in 37 °C, with 5% CO_2_ tissue culture incubators.

### GelMA and Carbopol Solution Preparation

GelMA was synthesized following the protocol previously described.^[^ ^42^ ^]^ Porcine gelatin powder (Sigma Aldrich, Cat. G2500) was fully dissolved at 10% w/v in PBS at 50 °C. Methacrylic anhydride (MA)(Sigma Aldrich, Cat. 276685) was then added in a dropwise fashion for gelatin modification at 50 °C for 3 h. The solution was then diluted with additional warm PBS to stop the reaction for 10 min. Following the dilution, the mixture was dialyzed against deionized water for 1 week at 40 °C, with water change 2–3 times a day. The solution was then lyophilized and stored away from light at −20 °C until use. A total of 10% w/v GelMA solution was prepared by reconstituting lyophilized GelMA powder into sterilized PBS with 0.5% w/v Irgacure (2‐hydroxy‐4′‐(2‐hydroxyethoxy)−2‐methylpropiophenone) (Sigma Aldrich, Cat. 410896). The reconstituted GelMA solution was stored away from ambient light at 4 °C and used within 2 weeks of reconstitution.

The Carbopol solution was prepared as previously described.^[^ ^69^ ^]^ Carbopol powder (Lubrizol, ETD 2020 NF polymer) was added into Milli‐Q water at a concentration of 0.5% w/v. The media was then stirred at a speed over 800 rpm at room temperature for 24 h. The Carbopol was aliquoted into 50 mL centrifuge tubes and pH balanced by adding 280 µL of 4 m NaOH. The pH balanced Carbopol medium was centrifuged at 3500 rpm for 10 min for degassing and then stored at 4 °C until used for bioprinting.

### ^1^H NMR Acquisition and Analysis

The chemical composition and methacrylation degree of GelMA bioinks were examined using ^1^H NMR at the Emory University NMR center following a previously published protocol method.^[^ ^106^, ^107^ ^]^ Briefly, we dissolved 10 mg of lyophilized GelMA foam (or gelatin powder) in 7.5 mL of deuterium oxide at 37 °C for 1 h. For each spectrum, the peak area of aromatic acids was used as a reference. To quantify the degree of methacrylation (substitution) (DM), the peak area of lysine methylene protons was entered into the following equation:

(1) $$\mathrm{DM}\;=\left(1-\frac{\mathrm{the}\;\mathrm{area}\;\mathrm{of}\;\mathrm{the}\;\mathrm{lysine}\;\mathrm{methylene}\;\mathrm{of}\;\mathrm{GelMA}}{\mathrm{the}\;\mathrm{area}\;\mathrm{of}\;\mathrm{the}\;\mathrm{lysine}\;\mathrm{methylene}\;\mathrm{of}\;\mathrm{Gelatin}}\right)\;\times 100\%$$

GelMA materials synthesized in three different (random) batches were used for the NMR analysis (*n* = 3).

### Mechanical Testing: GelMA Bioink Quality Control

After synthesis of each batch of GelMA, a quality control procedure was conducted to ensure batch‐to‐batch consistency of the bioink materials. For this purpose, reconstituted GelMA (10% w/v) was mixed with Irgacure photoinitiator (0.5%) and cast into 24‐well plates (300 µL), followed by cross‐linking with UV light at two different intensities (2.5 and 20 mW cm^−2^) for 2 min. The resulting GelMA discs were then probed by microindentation testing (Mach‐1 mechanical testing system, Biomomentum Inc., Quebec, Canada) to measure their elastic modulus.^[^ ^42^, ^108^, ^109^, ^110^ ^]^ Briefly, indentation tests were performed on the GelMA surface (*n* = 3) using a 500 µm spherical indenter with an indenting depth of 100 µm at 2 µm s^−1^. Subsequently, the force–displacement unloading data were used to calculate the stiffness (*S*) from the slope of the linear tread line at initial 5–20%.^[^ ^111^ ^]^ The reduced elastic modulus (*E*_r_) was calculated using the equations and assumptions below, as previously described^[^ ^111^, ^112^ ^]^:

(2) $$E_{r}=\;\frac{\sqrt{\pi }}{2\beta }\frac{S}{\sqrt{A\left(h_{c}\right)}}$$

where β constant = 1, *S* is the samples stiffness, and *A(h*_c_) is the projected contact area at the contact depth of *h*_c_, which can be determined using the following equation:

(3) $$A\;\left(h_{c}\right)=\;2\pi Rh_{c}-\;\pi h_{c}^{2}$$

where *h*_c_ can be found using:

(4) $$h_{c}=h_{\max}\;-\;\varepsilon \frac{P_{\max}}{S}$$

*H*_max_ and *P*_max_ in Equation (4) are the peak unloading displacement and peak unloading force, respectively. *ε* is the constant equal to 0.75 for the spherical probe.^[^ ^113^ ^]^

Finally, the elastic modulus, *E*, can then be calculated using the equation:

(5) $$\frac{1}{E_{r}}=\frac{\left(1-v^{2}\right)}{E}\;+\frac{\left(1-v_{i}^{2}\right)}{E_{i}}$$

In this equation, *v* and *v*_i_ are the Poisson's ratio of tested material and the indenter tip, where both are equal to 0.5. *E*_i_ is the elastic modulus of the probe with a value of 2 GPa.

### Mechanical Testing of Bioprinted Scaffolds

Mechanical properties of the bioprinted cubic scaffolds followed a similar workflow to the microindentation used to quality control GelMA. A 500 µm probe was used to indent the top and bottom surfaces of the cubic scaffold. Next, the constructs were sliced at a 2.5 mm depth and indentation was conducted on the side channel luminal surface and the central cavity where the organoid is placed. All indentations were performed at a depth of 300 µm at 7.5 µm s^−1^, with three indentation points for the top, bottom, and side channel surfaces, and one indentation point for the central cavity for each sample, with a total of *n* = 4 samples. All mechanical tests were performed at room temperature and hydrating the GelMA samples in 1× PBS between measurements. The force–displacement unloading curves were recorded and the slope of the linear trend line at the initial 5–20% displacement was used to determine the stiffness (*S*). Using Equations ((2), (3), (4), (5)), we determined the elastic modulus of different regions within the bioprinted scaffolds. A one‐way ANOVA with Tukey's multiple comparisons test with a significance level of *p* < 0.05 was performed to assess stiffness differences between the different regions of the bioprinted scaffolds.

### 3D Bioprinting of Two‐Layer Constructs

The two‐layer constructs used to quantify the strand fidelity are described from Ning et al.^[^ ^69^ ^]^ The designed strands are cylindrical with a diameter of *D*_d_ = 200 µm. The angle between the first and second layer is *α*_d_ = 60°. The interstrand area between four strands is *A*_d_ = 1.95 mm^2^. For printing, 10% GelMA solution was loaded into printing syringes of the BioX printer (CELLINK, USA). A 27 gauge (200 µm inner diameter) with a 0.5‐inch needle length (Nordson EFD, Cat. 76243–800) was used for all prints. The printing syringe was precooled for 20 min at a controlled temperature between 22 °Cand 24 °C. The printing pressure was varied to ensure consistent flow from the printing nozzle and the printing speed was 8 mm s^−1^. All prints were printed into a 0.5% Carbopol support bath.

### 3D Bioprinting of Cubic Scaffold

Using Fusion 360 (Autodesk, USA), a computer‐aided design software, we created a 5 × 5 × 5 mm cubic scaffold. The scaffold contains a 3 mm top channel, four interconnected 1 mm side channels, and a central cavity, which is used to house the organoid. The STL file of the cubic scaffold was sliced using the Slic3r program. Each layer is 0.2 mm in thickness. The G‐code was then uploaded to the BioX printer. As described above, the 10% GelMA solution was loaded into printing syringes using a 27‐gauge, 0.5‐inch needle. Printing can occur on the bench top (with the BioX clean chamber fan on) or in a biosafety cabinet. After loading the GelMA syringe into the temperature‐controlled nozzle, we ran a “Clean Chamber” cycle on the BioX printer, to UV sterilize the printing chamber. Following the sterilization cycle, we turned on the “Clean Chamber Fan,” which initiates the flow of filtered air into the printing chamber. The GelMA was precooled for 20 min between 22 °C and 24 °C. The printing pressure was varied to make sure there was a consistent flow of the GelMA out of the printing nozzle. The printing speed was set to 8 mm s^−1^. A 0.5% Carbopol support bath was used for all prints. Following printing, samples are UV cross‐linked (320–365 nm) at 25 mW cm^−2^ for 2 min (1 min side^−1^). Scaffolds were recovered from the Carbopol bath and washed in PBS and antibiotic/antimycotic (Thermo Scientific, Cat. 15 240 062) twice and placed on a rocker overnight at room temperature. Anti/anti washes were done to further ensure the sterility of the bioprinted scaffolds but are optional. The scaffolds are then stored at 4 °C, up to 2 weeks, until ready to be used.

### Printing Fidelity Characterization

The printing fidelity was assessed as previously described.^[^ ^69^ ^]^ Optical microscopy images for all bioprinted constructs were acquired using the ECHO Revolve (Echo, USA) and processed using ImageJ (National Institutes of Health, Maryland USA). For the two‐ layer constructs, we quantified the strand diameter ratio, strand angle ratio, and interstrand area ratio. A total of *n* = 9 two‐layer prints were used. Three measurements of strand diameter, strand angle, and interstrand area were taken from each sample, resulting in 27 data points.

Strand diameter ratio was defined as the ratio of the diameter of the actual printed strand (*D*_p_) and the designed (CAD) strand diameter (*D*_d_ = 200 µm).

(6) $$D\;=\;\frac{\mathrm{Diameter}\;\mathrm{of}\;\mathrm{printed}\;\mathrm{strand},\;\;\;D_{p}}{\mathrm{Diameter}\;\mathrm{of}\;\mathrm{designed}\;\mathrm{strand},\;\;\;D_{d}}$$

The strand angle ratio was used to quantify the distortion of the printed layers and is defined as the ratio of the actual angle between two printed strands (*α*_p_) and the designed angle between two strands (*α*_d_ = 60°).

(7) $$\alpha \;=\frac{\mathrm{Measured}\;\mathrm{angle}\;\mathrm{between}\;\mathrm{two}\;\mathrm{printed}\;\mathrm{strands},\;\;\;\alpha_{p}}{\mathrm{Designed}\;\mathrm{angle}\;\mathrm{between}\;\mathrm{two}\;\mathrm{printed}\;\mathrm{strands},\;\;\alpha_{d}}$$

The interstrand area ratio was defined as the area of the parallelogram created by four strands formed from two consecutive printed layers. The interstrand area ratio is the ratio of the actual surface area (*A*_p_) between printed strands and the designed surface area between strands (*A*_d_ = 1.95 mm^2^).

(8) $$A\;=\;\frac{\mathrm{Measured}\;\mathrm{area}\;\mathrm{between}\;\mathrm{printed}\;\mathrm{strands},\;\;A_{p}}{\mathrm{Designed}\;\mathrm{area}\;\mathrm{between}\;\mathrm{printed}\;\mathrm{strands},\;\;A_{d}}$$

For bulk fidelity measurements, *n* = 12 scaffolds were printed and manually measured. The length, height, top channel diameter, and side channel diameter ratio were quantified by acquiring images of the top, bottom, and two adjacent sides of the cubic scaffold. For the top and bottom surfaces, the length and width of the printed scaffold were measured. These measurements were considered to be the “length” parameter. Furthermore, for the top surface, multiple measurements were taken to determine the diameter of the top channel. For the sides of the cube, the length and height of the scaffold were measured, in addition to taking multiple measurements for the side channel diameter.

The length ratio is defined as the ratio of the length (and width) of the printed scaffold (*l_p_*) and the designed (CAD) length of the scaffold (*l_d_* = 5 mm). This parameter was used to assess deformation during the layer printing. A high printing fidelity was achieved when the length ratio values are close or equal to 1, while length deformation exists when the ratio values are far from 1.

(9) $$l\;=\;\frac{\mathrm{Measured}\;\mathrm{length}\;\mathrm{of}\;\mathrm{printed}\;\mathrm{scaffold},\;\;\;l_{p}}{\mathrm{Desinged}\;\mathrm{length}\;\mathrm{of}\;\mathrm{printed}\;\mathrm{scaffold},\;\;l_{d}}$$

The height ratio is defined as the ratio of the height of the printed scaffold (*h*_p_) and the designed (CAD) height of the scaffold (*h*_d_ = 5 mm). This parameter is used to assess any deformation that is caused by gravity. A high printing fidelity is achieved when the height ratio values are close or equal to 1, while scaffold height deformation occurs when the ratio values are greater or less than 1.

(10) $$h\;=\;\frac{\mathrm{Measured}\;\mathrm{height}\;\mathrm{of}\;\mathrm{printed}\;\mathrm{scaffold},\;\;h_{p}}{\mathrm{Desinged}\;\mathrm{height}\;\mathrm{of}\;\mathrm{printed}\;\mathrm{scaffold},\;\;h_{d}}$$

The top channel diameter ratio is defined as the ratio of the diameter of the printed top channel (*D*_p‐TC_) and the designed (CAD) diameter of the top channel (*D*_d‐TC_ = 3 mm).

(11) $$D_{\mathrm{TC}}=\;\frac{\mathrm{Measured}\;\mathrm{diameter}\;\mathrm{of}\;\mathrm{the}\;\mathrm{top}\;\mathrm{channel},\;\;\;D_{p-TC}}{\mathrm{Desinged}\;\mathrm{diameter}\;\mathrm{of}\;\mathrm{the}\;\mathrm{top}\;\mathrm{channel},\;\;\;D_{d-TC}}$$

The side channel diameter is defined as the ratio of the diameter of the printed side channel (*D*_p‐SC_) and the designed (CAD) diameter of the side channel (*D*_d‐SC_ = 1 mm).

(12) $$D_{\mathrm{SC}}=\;\frac{\mathrm{Measured}\;\mathrm{diameter}\;\mathrm{of}\;\mathrm{the}\;\mathrm{side}\;\mathrm{channel},\;\;\;D_{p-\mathrm{SC}}}{\mathrm{Desinged}\;\mathrm{diameter}\;\mathrm{of}\;\mathrm{the}\;\mathrm{side}\;\mathrm{channel},\;\;D_{d-\mathrm{SC}}}$$

### GelMA Pore Size Quantification

To determine the porosity of the GelMA scaffolds, a total of *n* = 4 scaffolds were prepared. Following UV cross‐linking, the printed scaffolds were placed in DI water for 3 days. The water was changed twice a day to ensure that the Carbopol was removed. Following the DI water washes, samples were transferred to a Petri dish and a Kimwipe was used to remove as much of the bulk fluid as possible. A total of 50 mL of 100% EtOH was poured into a container and 5–10 pieces of dry ice were placed in the container to keep the temperature low. The Petri dish containing the samples was placed on top of the EtOH pool. Scaffolds were kept in the solution for 2–5 min as they gradually freeze dry. EtOH and dry ice were added as needed to maintain the temperature. Freeze‐dried constructs were transferred into a 50 mL conical tube and preserved in −80 °C until ready to lyophilize. To lyophilize the samples, a Kimwipe was taped to cover the conical tube, placed in a jar, and connected to the lyophilizer for 5 days. Samples are removed from the lyophilizer and carefully transferred to a 12‐well plate and stored at room temperature until imaging.

Lyophilized scaffolds were imaged using a scanning electron microscope (Axia ChemiSEM, Thermo Fisher) at the Materials Characterization Facility (MCF), Institute for Materials (IMat) at Georgia Institute of Technology. Four samples were imaged, with a total of four images taken randomly per sample. Using ImageJ, the area of 15 pores per image was manually measured (*n* = 240 measurements (60 measurements per scaffold)). Using the measured area, the pore diameter was back calculated and the values were used to generate a histogram of the pore size. A Gaussian distribution fit was applied to the histogram of the pore size. To assess if the data was normally distributed, D'Agostino‐Pearson omnibus, Anderson‐Darling, Shapiro–Wilk, and Kolmogorov–Smirnov normality tests were ran, with a significance level of *p* < 0.05, which demonstrated a normal distribution.

Since not all pores were circular, two perpendicular diameters for *n* = 20 pores per sample (*n* = 80 total) were measured from the images acquired above. Using these values, the aspect ratio of the pores was found. The more circular a pore is, the closer the aspect ratio will be to 1.

### Embedding hCS into GelMA Scaffold for Long‐Term Culture

When preparing to embed the organoids, cubic scaffolds were removed from the 4 °C fridge and washed three times with PBS and antibiotic/antimycotic. The PBS was aspirated off and the scaffolds were placed so that the top channel was facing up. To ensure that the top channels were sufficiently open to allow for embedding, they are poked with a plunger. A tip of a P1000 was cut off to transfer an organoid from a culture dish to a cubic scaffold and seeded through the top channel. Following seeding, 25 µL of 10% GelMA was added to the top channel cavity and the scaffold was cross‐linked for 1 min at 25 mW cm^−2^ to seal the top channel. Embedded organoids were seeded between day 20 and 25 of the neural induction and differentiation timeline. After seeding, the embedded organoids followed the same feeding paradigm as the suspended controls. From days in scaffold (DIS) 0 to 30, size images were taken every 6 days. From DIS 30 to 60, size images were taken every 10 days. ImageJ was used to measure the diameter of the suspended and embedded organoids, which was characterized as the organoid size. The normalized growth was determined by taking the average of the suspended and embedded organoid size at DIS 0 and normalizing the measured values on subsequent days to these values. Using GraphPad Prism, a two‐way ANOVA with Tukey's multiple comparison test was performed with a significance level of *p* < 0.05 on the organoid size data. On DIS 30 and DIS 60, samples were fixed for immunohistochemistry (IHC), dissociated for cell viability analysis (1363 AggreWell and 8858 Dispase), and RNA was extracted for bulk RNA sequencing.

### Encapsulating hCS into Matrigel Droplets

To compare the GelMA embedded to other matrix based hCO culturing approaches, organoids were encapsulated from 1 hiPSC line in Matrigel by adapting the protocol from Agoglia et al.^[^ ^114^ ^]^ Briefly, Matrigel aliquots were thawed overnight at 4 °C and P1000 tips were precooled in the freezer. Ultralow attachment plates were prepared by washing 10 cm tissue culture treated plated with 5 mL of Anti‐Adherence Rinsing solution (STEMCELL Technologies, Cat. # 0 7010). After aspirating the Anti‐Adherence Rinsing solution, plates were washed with DMEM/F12 to remove any excessing rinsing solution. A total of 12 mL of organoid patterning media was added to the plate and stored in the incubator (37 °C, 5% CO_2_) until ready to use. Individual day 20 organoids were moved into 1.5 mL microtubes by cutting a P1000 tip and excess media was removed. Using the precooled, cut P1000 tip, Matrigel was added to the microtube and pipetted up and down. Organoids were gently resuspended in Matrigel and small droplets were made on a 10 cm tissue culture dish and placed the Matrigel droplets in the incubator (37 °C, 5% CO_2_) for 30 min. After incubation, 5 mL of DMEM/F12 was added to the plate and droplets were gently scraped off the plate using a cell scraper. Matrigel droplets containing hCOs were moved into the previously prepared ultralow attachment plates containing the appropriate patterning media and cultured statically. Media changes and supplements are based on the suspended organoid differentiation protocol.

### Cell Viability

To assess the viability of control suspended organoids and the organoids embedded in our GelMA scaffolds, a LIVE/DEAD assay (ThermoFisher L3224) was used. Organoids were first dissociated into a single cell suspension following an established protocol.^[^ ^3^ ^]^ Briefly, an organoid was chopped into small pieces using a scalpel and digested with papain. Following digestion, cells were spun down and resuspended in media containing 2 × 10^−6^ m calcein‐AM and 4 × 10^−6^ m ethidium homodimer‐1. The resuspended cells were plated into a 24‐well plate then sat at room temperature for 30 min. Each well was imaged under the fluorescent mode on the Revolve epifluorescence microscope (ECHO) and three images were randomly taken for each sample. Cells that are alive will fluoresce green (495 nm/515 nm Ex/Em) and dead cells will fluoresce red (495 nm/635 nm Ex/Em). At DIS 30 for both cell lines, there was a total of *n* = 6 samples for each group. At DIS 60 the number of samples dissociated and quantified for cell viability were *n* = 4 and 6 for the 1363.1 and 8858.3 cell lines, respectively. The acquired fluorescent images were then processed using the analyze particles functions on ImageJ. The mean and standard deviation was expressed. Using GraphPad Prism, a two‐way ANOVA with Šidák's multiple comparison test was performed with a significance level of p < 0.05.

### Immunohistochemistry

Samples were fixed using 4% paraformaldehyde (PFA) for 1–2 h at 4 °C. Following fixing, samples were washed three times with PBS and stored in PBS at 4 °C, until prepared to be sectioned. Samples were embedded in 4% agarose (ThermoFisher, Cat.16520050) and sectioned using a vibratome (VT 1200, Leica BioSystems). Sections were approximately 300 µm thick and stored in PBS until staining. To stain, samples were first blocked for 30 min at room temperature, using blocking buffer containing 10% donkey serum and 0.3% Triton X. The blocking buffer was removed and primary antibodies, diluted in blocking buffer, were added to the sections, and incubated overnight on a rocker at 4 °C. The primary antibody solution was removed and three, 15‐min PBS washes are performed at room temperature. Secondary antibodies and DAPI were diluted in blocking buffer, added to the sections, and incubated for 2 h at room temperature, protected from light. The secondary antibody solution was removed and three, 15‐min PBS washes were performed at room temperature. Samples were mounted onto microscope slides and allowed to air dry for 5–10 min. Mounting media (Thermo Fisher, Cat. 00‐4958‐02) was placed on the slides and covered with a coverslip. Samples dry for 24–48 h and were stored at 4 °C until ready for imaging. A list of the primary and secondary antibodies used in this work are listed below.

### Primary Antibodies

**Table:** 

Antibody	Host species	Clonality	Company	Catalog number	Dilution
Mouse anti‐TUBB3 (TUJ1)	Mouse	Monoclonal	BioLegend	801201	1:1000
Chicken anti‐GFP	Chicken	Polyclonal	Aves	GFP‐1020	1:2000
Ulex Europaeus Agglutinin I (UEA I), Rhodamine	*Ulex europaeus (gorse)*	N/A	Vector Laboratories	RL‐1062‐2	1:50
Mouse anti‐Nestin	Mouse	Monoclonal	Abcam	Ab22035	1:500
Rabbit anti‐Pax6	Rabbit	Monoclonal	Cell Signaling Technology	60433S	1:200

### Secondary Antibodies

**Table:** 

Antibody	Company	Catalog number	Dilution
DAPI	Invitrogen	D1306	1:1000
Goat anti‐Chicken (488)	Abcam	Ab150173	1:1000
Goat anti‐Rabbit (594)	Abcam	Ab150088	1:1000
Donkey anti‐Mouse (647)	Abcam	Ab150111	1:1000

### EdU Exposure and Quantification

For the second batch of line 1 and line 2, DIS 28 suspended and embedded organoids to EdU (Fisher Scientific, Cat. C10640) (concentration). A total of 48 h later (DIS 30) we fixed the EdU exposed samples in 4% PFA for 1–2 h at 4 °C. Following sectioning, samples were stained with EdU to quantify the degree of active cell division following the manufacturer's recommendations. A total of *n* = 4 samples per condition (suspended or embedded) per cell line (line 1 or line 2) were sectioned and stained (*n* = 16 organoids). Confocal images were taken on the Leica Stellaris 5 confocal microscope. These images were then processed using ImageJ. Using the analyze particle function in ImageJ, the total number of EdU+ cells were counted and normalized by the number of DAPI cells. A two‐way ANOVA with Sidak's multiple comparisons test was performed with a significance level of *p* < 0.05.

### TUNEL Staining and Quantification

To ensure that live/dead analysis was accurate and not undercounting dead or dying cells, TUNEL staining was performed on fixed and sectioned hCOs from suspended, embedded, and Matrigel encapsulated conditions. Tissue sections were placed in a 24‐well plate and stained following the Click‐iT Plus TUNEL Alexa Fluor 594 Assay Kit (Thermo Fisher, Cat. C10618) protocol for cells grown on coverslips. One important difference is that DAPI was used instead of Hoechst 33 342 to stain nuclei. After staining, hCO sections were mounted onto coverslips and confocal images were taken on the Leica Stellaris 5 confocal microscope. The acquired images were then processed using ImageJ and the analyze particle function to count the number of TUNEL+ and total DAPI cells. A total of *n* = 3 organoids per condition were used for the analysis (9 organoids total). For each organoid, three separate tissue sections were imaged to ensure there was no regional bias in the percentage of TUNEL+ cells. The percentage of TUNEL+ cells across the three tissue sections were averaged to determine the overall amount of cell death within that organoid. A one‐way ANOVA with Tukey's multiple comparisons test was run, with a significance level of *p* < 0.05, and showed no significant difference in the percentage of TUNEL+ cells between the three different culture conditions.

### RNA Sequencing

RNA was extracted using the miRNeasy Micro Kit and RNase‐Free DNase set (Qiagen, Cat. 217 084 and 79 254 respectively). The quality of the RNA was validated by running a picochip (Agilent RNA 6000 Pico, Cat. 5067‐1513) on the Bioanalyzer 2100 (Agilent) and the concentration of RNA was determined using the Nanodrop (Thermo Fisher). Samples with high RNA integrity number (>8) were selected. Samples were sent to Admera Health for library preparation (TruSeq Stranded mRNA kit with Poly‐A selection or NEB Ultra II Kit with Poly‐A Selection) and were bulk sequenced at 40 million pair end reads per sample. Following sequencing, sequencing reads were trimmed using the Trimmomatic software,^[^ ^115^ ^]^ mapped to the GRCh38/hg38 human reference genome using STAR aligner,^[^ ^116^ ^]^ and the featureCounts software summarized the mapped reads.^[^ ^117^ ^]^ After obtaining the feature counts data, batch effect normalization was performed using the sva package^[^ ^118^ ^]^ and normalized count data were obtained. This work then adapted established bulk RNA sequencing processing pipelines,^[^ ^119^, ^120^ ^]^ to perform DGE on normalized count data. Differential expression was performed using the edgeR package.^[^ ^119^ ^]^ Cutoffs to determine differentially expressed genes were set to a log fold change of ± 1 and an adjusted *p* value < 0.01. After acquiring a list of differentially expressed genes, GO analysis was performed using gProfiler.^[^ ^121^ ^]^

### Identifying Optimal HUVEC Media

GFP positive HUVECs (ATCC) were cultured in tissue culture flasks (VWR, Cat. 10062–860) at 37 °C with a 5% CO_2_ and fed with VEGF Endothelial Medium Complete Kit (ATCC, PCS‐100‐030 and PCS‐100‐041). To test the different media conditions, HUVECs were harvested at 90%‐confluency and passaged into a tissue culture treated 24‐well plate (VWR 29442‐044) at a seeding density of 2000 cells per well. Each condition (S3A,B) had three technical replicate wells. Media was changed every 2–3 days. When testing the role of switching from normal HUVEC media to the 50/50 HUVEC:neurobasal mix, HUVECs were cultured in their control media for 3 days. On day 3, the media was switched to the 50/50 mix and cultured for another 3 days.

Cell viability was assessed using the alamarBlue assay (ThermoFisher, DAL1025), as previously described.^[^ ^42^ ^]^ Briefly, alamarBlue was added to fresh media in a 1:9 volumetric ratio and then added to the sample wells. HUVECs were incubated in the media and alamarBlue mix for 4 h. After the incubation, 100 µL of media was collected from each sample and placed in a 96‐well plate (VWR 29442‐056). A total of 100 µL of fresh media (± alamarBlue) from each media condition tested was also collected and placed in the 96‐well plate to normalize the alamarBlue reduction. The absorbance was read using a microplate reader (BioTek Instruments, USA) at the wavelengths of 550 and 600 nm. Percentage of AlamarBlue reduction was calculated on days 1, 3, and 6 (*n* = 3 wells per condition). Two‐way ANOVA with Tukey's or Dunnett's multiple comparison test and significance level of *p* < 0.05 was performed.

### Coculture of Endothelial Cells and Cortical Organoids

To prepare the printed samples for the coculture experiments, printed scaffolds were washed with 1× PBS and then submerged in 0.5% gelatin (Sigma Aldrich, G2500) with 1% antimycotic–antibiotic at 37 °C overnight. The gelatin was removed, and scaffolds were washed three times with 1× PBS. Scaffolds were kept in PBS while the HUVECs (Lifeline Cell Technology, Frederick, MD) were prepared, following previous published protocols.^[^ ^42^ ^]^

GFP positive HUVECs were cultured in tissue culture flasks at 37 °C with 5% CO_2_ and fed with VEGF Endothelial Medium Complete Kit. Scaffolds were removed from the PBS, oriented to ensure the top channel was facing upward, and poked to ensure the top channel is open. HUVECs were harvested at 90% confluency and manually pipetted through the top channel of the gelatin precoated GelMA construct at a density of 500 000 cells per construct. For this purpose, cells were distributed equally into the four lateral channels of the cube. Cells were incubated for 4 h to ensure cells attachment and transferred to a tissue culture flask. HUVEC laden scaffolds were cultured at 37 °C with 5% CO_2_ for 5 days, with media changes occurring every 2–3 days.

After 5 days of HUVEC only culture, scaffolds were prepared for cortical organoids to be manually embedded, as previously described above. Organoids were between day 18 and 30 when seeded. Following seeding, 25 µL of 10% GelMA was added to the top channel cavity and the scaffold was UV cross‐linked (320–365 nm) for 1 min at 25 mW cm^−2^ to seal the top channel. This was designated as DIS 0. Scaffolds were fed with 50% HUVEC media and 50% neural media and all supplements were added to the media. On DIS 0, 7, 14, 21, and 28, size images were taken on the ECHO Revolve. On DIS 7, 14, and 28 samples were fixed for IHC. On DIS 28, samples were prepared for single cell RNA sequencing. As a control, hCOs were loaded into scaffolds that did not contain HUVECs but that were fed with the composite media (50% HUVEC, 50% neural media).

### Infiltration Quantification—HUVEC Infiltration Depth

To quantify the infiltration of the ECs into the cortical organoids following coculture, 10× confocal images were taken. Using ImageJ, a boundary was first drawn around the organoid. The perpendicular We was then measured from that boundary to the furthest ECs that had entered the organoid. This was considered infiltration distance. A total of *n* = 4–6 scaffolds were used for each timepoint (*n* = 12–18 organoids total). One‐way ANOVA with Tukey's multiple comparisons test with significance level *p <* 0.05 was performed.

### Infiltration Quantification—HUVEC Area Fraction

To determine how much of the organoid area was covered by HUVECs, a border was first drawn around the organoid in ImageJ using the DAPI channel. This mask was then applied to the GFP channel to remove any background noise and ensure measuring only the area of the organoid. The GFP channel was then thresholded and measured the area fraction. One‐way ANOVA with Tukey's multiple comparisons test with significance level *p <* 0.05 was performed.

### Single Cell Capture and Analysis

HUVECs were cultured in T‐75 flasks with VEGF endothelial medium (Lifeline Cell Technology, LL‐0003) and dissociated using Trypsin‐EDTA (0.05%, Gibco) at 90–95% confluency. After harvesting, the cells were resuspended in VEGF endothelial medium to maintain cell viability >95%, sent to Emory Integrated Genomics Core for 10× chromium single‐cell RNA‐seq library preparation, and sequenced using NovaSeq SP.

Embedded only and coculture scaffolds were cultured for 28 days before being dissociated for single cell capture. Scaffolds were dissociated with papain, as described in the Cell Viability Experimental Section, and collagenase type IV (Thermo Fisher, Cat. 17104019) at a concentration of 4 mg mL^−1^. Collagenase was added to remove any unwanted GelMA debris. Furthermore, transcriptional and translational inhibitors, Actinomycin D (Millipre Sigma, Cat. A1410) and Protector RNase Inhibitor (Millipore Sigma, Cat. 3335399001) were also added.^[^ ^122^ ^]^ Actinomycin D and Protector RNase Inhibitor were added to the digestion solution at a concentration of 1:1000 and 1:2000, respectively. Following the digestion, the dissociated cells from coculture samples were centrifuged, resuspended in panning buffer containing CellTracker Deep Red (Thermo Fisher, Cat. C34565), and then filtered to remove any debris. The cells were then brought to the Emory Flow Core to sort for live organoid (647+) and HUVEC (GFP+, 647+) and cells. Following sorting, the organoid and HUVECs were manually mixed at a ratio of 80:20 to enrich organoid cells.

Embedded only scaffolds were also dissociated with papain, collagenase, Actinomycin D, and Protector RNase Inhibitor. Following the digestion, the cell solution was centrifuged, resuspended in panning buffer, and filtered. Single cell capture and library preparation for the embedded and coculture samples was completed using Chromium Next GEM Single Cell 3′ Reagent Kits (v3.1 Dual Index – 10×Genomics) following manufacturer's protocols. Embedded and coculture samples were sent to Admera Health for sequencing, using the NovaSeq X.

The CellRanger pipeline^[^ ^123^ ^]^ (10× Genomics) was used to align reads to the GRCh28 human reference genome and output a cell‐by‐gene count matrix. Cells expressing a minimum of 250 genes and less than 20% mitochondrial genes were filtered, normalized, and variable features were identified. The three datasets (embedded organoid cells, HUVEC monoculture, and coculture cells) were then integrated and dimension reduction analysis was performed for visualization and unsupervised clustering analysis. A total of 24 399 cells from embedded organoids (*n* = 7 scaffolds, 5839 cells), 2D HUVEC monoculture (9825 cells), and embedded organoids cultured with HUVECs (*n* = 12 scaffolds, 8735 cells) were clustered. The FindAllMarkers function was next run to identify marker genes from each cluster to aid in naming clusters. After identifying neuronal and EC populations, the EC population was reclustered and DGE was performed between HUVECs cultured in the GelMA scaffolds with organoids and HUVECs from the monoculture. Using Enrichr,^[^ ^124^, ^125^ ^]^ the predicted cell type can be determined based on the differentially expressed gene list.

### Statistical Analysis

Experimental data were processed and expressed using mean values ± standard deviation (SD) or standard error of the mean (SEM). GraphPad Prism was used for all statistical processing. Statistical significance was determined by one‐way or two‐way ANOVA and multiple comparisons (Tukey's, Šidák's, or Dunnett's) were performed with a significance level of *p* < 0.05. In the entire study, significance is denoted as follows: ^*^ *p* < 0.05, ^**^ *p* < 0.01, ^***^ *p* < 0.001. The specific multiple comparison test used for each quantification is presented in each Experimental Section. Sample size (*n*) has been presented in each Experimental Section.

## Conflict of Interest

The authors declare no conflict of interest.

## Supporting information

Supporting Information

Supplemental Movie 1

## Data Availability

The data that support the findings of this study are available from the corresponding author upon reasonable request.
