# Supplementary material for: A 3D Bioprinted Cortical Organoid Platform for Modeling Human Brain Development
Source: Adv Healthc Mater. 2024 Jun 8;13(27):2401603. doi: 10.1002/adhm.202401603 (PMC11518656; doi:10.1002/adhm.202401603)
Supplement: Supplementary file 1 — Supporting Information [file ADHM-13-0-s002.pdf]

ADVANCED HEALTHCARE MATERIALS

Supporting Information

for *Adv. Healthcare Mater.*, DOI 10.1002/adhm.202401603

A 3D Bioprinted Cortical Organoid Platform for Modeling Human Brain Development

Melissa A. Cadena, Anson Sing, Kylie Taylor, Linqi Jin, Liqun Ning, Mehdi Salar Amoli, Yamini Singh, The Brain Organoid Hub, Samantha N. Lanjewar, Martin L. Tomov, Vahid Serpooshan and Steven A. Sloan**

A 3D BIOPRINTED CORTICAL ORGANOID PLATFORM FOR MODELING HUMAN BRAIN DEVELOPMENT

Melissa A. Cadena[1,2], Anson Sing[2], Kylie Taylor[2], Linqi Jin[1], Liqun Ning[1,3], Mehdi Salar Amoli[1], Yamini Singh[1], The Brain Organoid Hub[2], Samantha N. Lanjewar[2], Martin L. Tomov[1,4], Vahid Serpooshan[1,4,5,*], Steven A. Sloan[2*]

[1] Department of Biomedical Engineering, Emory University School of Medicine and Georgia Institute of Technology, Atlanta, GA 30322, USA
[2] Department of Human Genetics, Emory University School of Medicine, Atlanta, GA 30322, USA
[3] Department of Mechanical Engineering, Cleveland State University, Cleveland, OH, United States
[4] Department of Pediatrics, Emory University School of Medicine, Atlanta, GA 30322, USA
[5] Children's Healthcare of Atlanta, Atlanta, GA 30322, US

* Co-corresponding authors

Corresponding Author Emails: vahid.serpooshan@emory.edu and steven.a.sloan@emory.edu

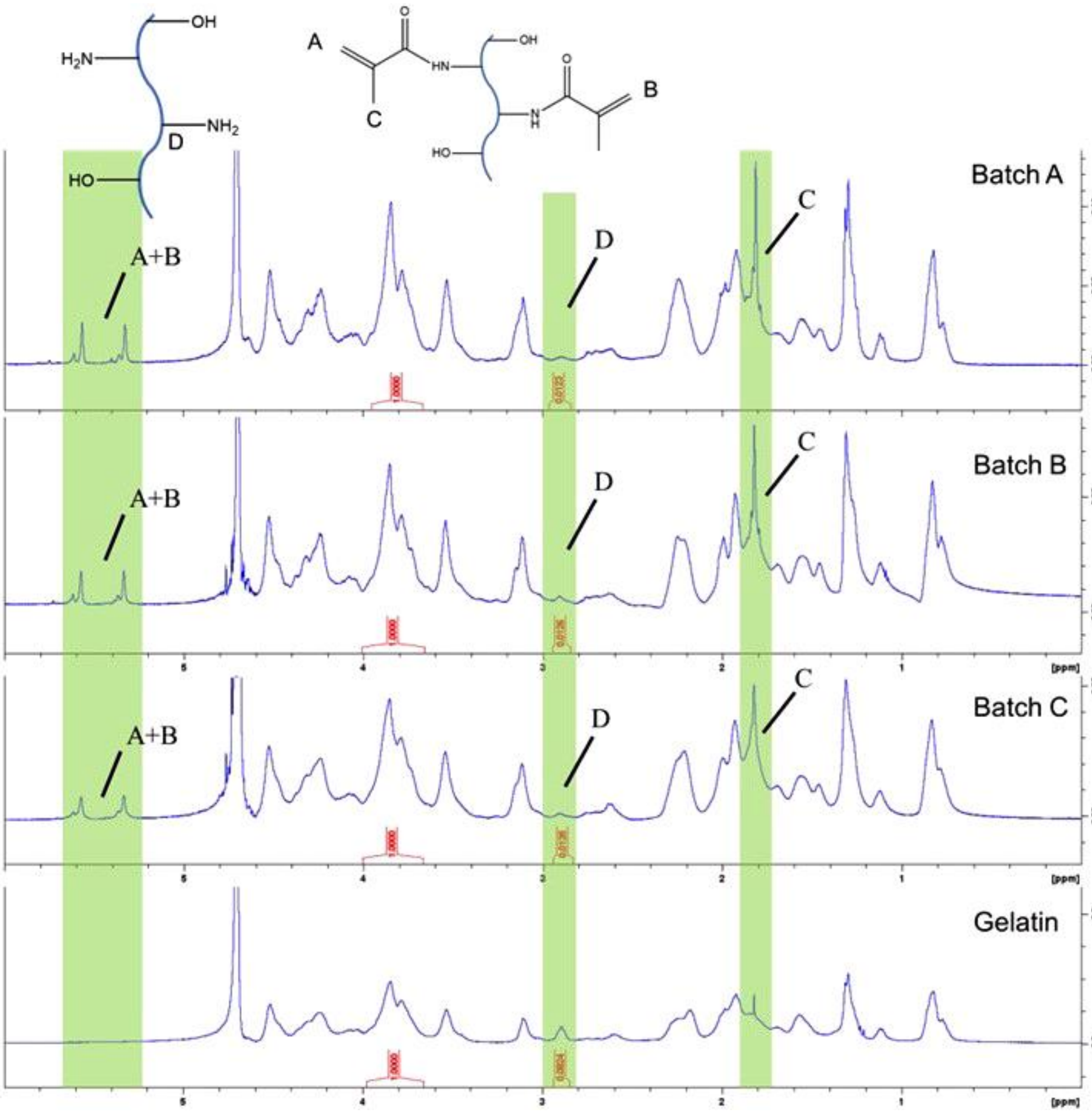

Figure S1: ^{1}H NMR spectra of three randomly selected batches of gelatin methacrylate (GelMA) synthesized in-house for use in the study.

Peaks A and B at ~ 5.3 and 5.5 ppm correspond to the acrylic proton of methacryloyl groups. Peak C at the 1.9 ppm signal is attributed to the methyl proton of the methacryloyl group. The intensity of the signal at 2.9 ppm (Peak D), corresponding to the unmodified lysine methylene, and is reduced significantly in GelMA samples compared to gelatin, demonstrating effective grafting of methacryloyl groups onto gelatin backbone. As calculated through the peak integration, the reduction in area under the lysine methylene peak shows a substitution degree of 85.07 % for batch A, 84.71 % for batch B, and 83.62 % for batch C.

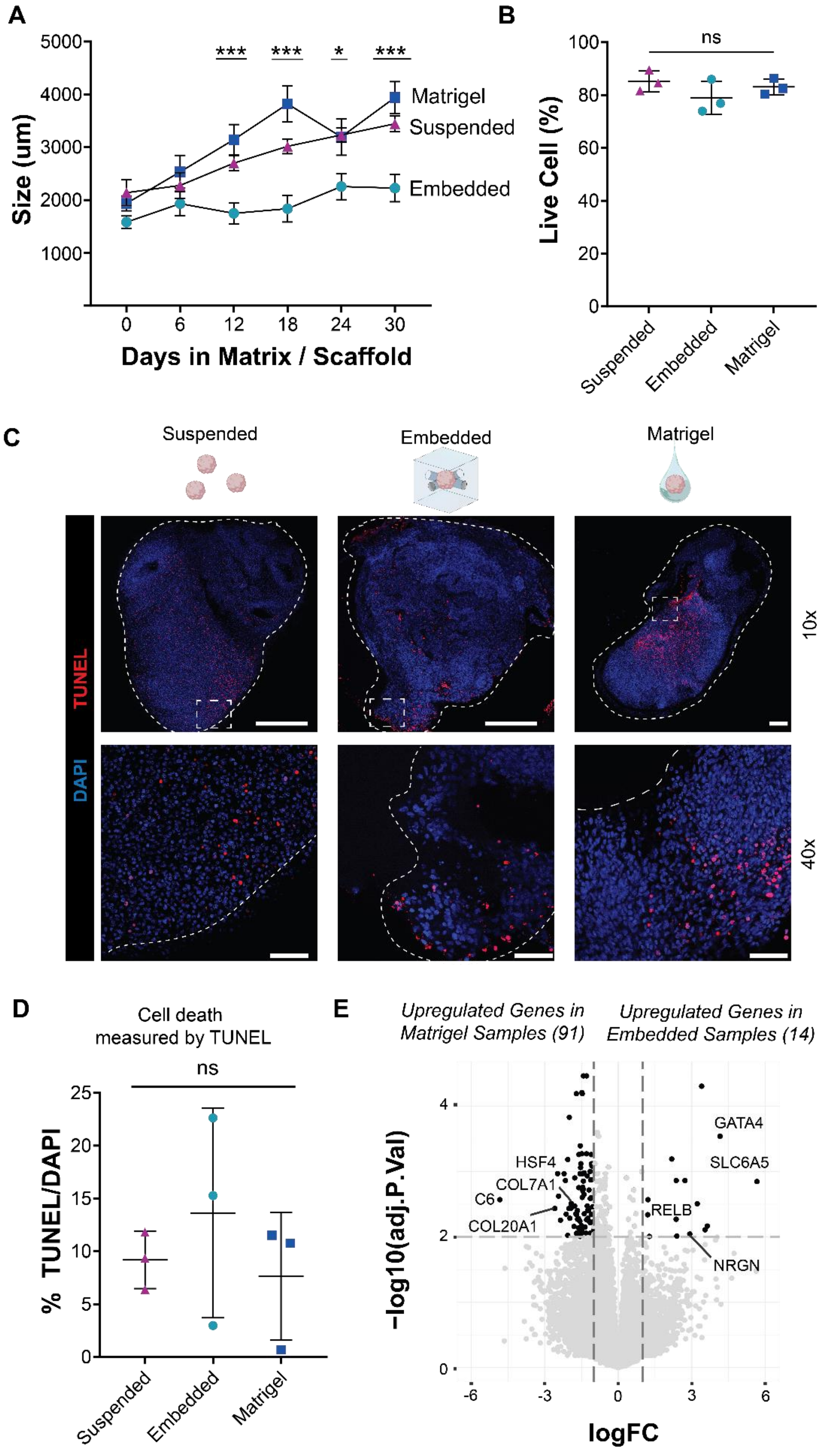

A
5000
4000
3000
2000
1000
Size (um)
***
***
*
***
Matrigel
Suspended
Embedded
0
6
12
18
24
30
Days in Matrix / Scaffold
B
100
80
60
40
20
0
Live Cell (%)
ns
Suspended
Embedded
Matrigel
C
Suspended
Embedded
Matrigel
TUNEL
DAPI
10x
40x
D
Cell death
measured by TUNEL
ns
25
20
15
10
5
0
% TUNEL/DAPI
Suspended
Embedded
Matrigel
E
Upregulated Genes in
Matrigel Samples (91)
Upregulated Genes in
Embedded Samples (14)
-log10(adj.P.Val)
4
2
0
HSF4
COL7A1
C6
COL20A1
GATA4
SLC6A5
RELB
NRGN
-6
-3
0
3
6
logFC

Figure S2: Assessment of matrix composition on organoid growth and viability.

(A) Size measurements performed on suspended (control), Matrigel droplet embedded, or GelMA scaffold embedded hCOs from DIS 0 to 30. A total of *n* = 5-6 samples per condition were used for the entirety of the 30 days. All data is presented from 1 hiPSC line. A Two-way ANOVA demonstrates a significant difference between the culture condition and age of the organoid ($p = 0.04$). Performing Tukey's multiple comparisons test, we find that after day 12 there is a growth advantage in Matrigel and suspended conditions compared to hCOs. Significance levels demonstrate the comparison tests between the embedded and Matrigel conditions. * $p<0.05$, ** $p < 0.01$, *** $p<0.001$. Error bars represent ± SEM. **(B)** Live/dead quantification of dissociated single cell suspensions from *n* = 3 samples per condition at DIS 30. Live cell percentages are as follows: 85.29 ± 3.99% for suspended, 78.95 ± 6.27% for embedded, and 83.12 ± 2.98% for Matrigel encapsulated hCOs, respectively. A $p= 0.30$ was determined by a one-way ANOVA with Tukey's multiple comparisons test. Error bars represent ± SD **(C)** TUNEL staining on sectioned suspended, Matrigel-encapsulated, and GelMA embedded organoids to test rates of cell death. The top row are images taken at 10x magnification and the bottom row taken at 40x magnification. Scale bars are 250 µm (10x) and 50 µm (40x). **(D)** Quantification of the percentage of TUNEL positive cells normalized to the number of DAPI cells. The percentage of TUNEL+ cells are as follows: 9.18 ± 2.71% for suspended, 13.63 ± 9.92% for embedded, and 7.65 ± 6.04% for Matrigel encapsulated hCOs. $p = 0.57$ was determined by a one-way ANOVA with Tukey's multiple comparisons test. Error bars represent ± SD **(E)** Differential gene expression analysis between Matrigel-encapsulated and GelMA embedded hCOs reveals a small number of differentially expressed genes (14 upregulated in GelMA embedded samples and 91 upregulated in Matrigel-encapsulated samples). Cutoffs are logFC ± 1 and adjusted p. value < 0.01. Schematics created with BioRender.com

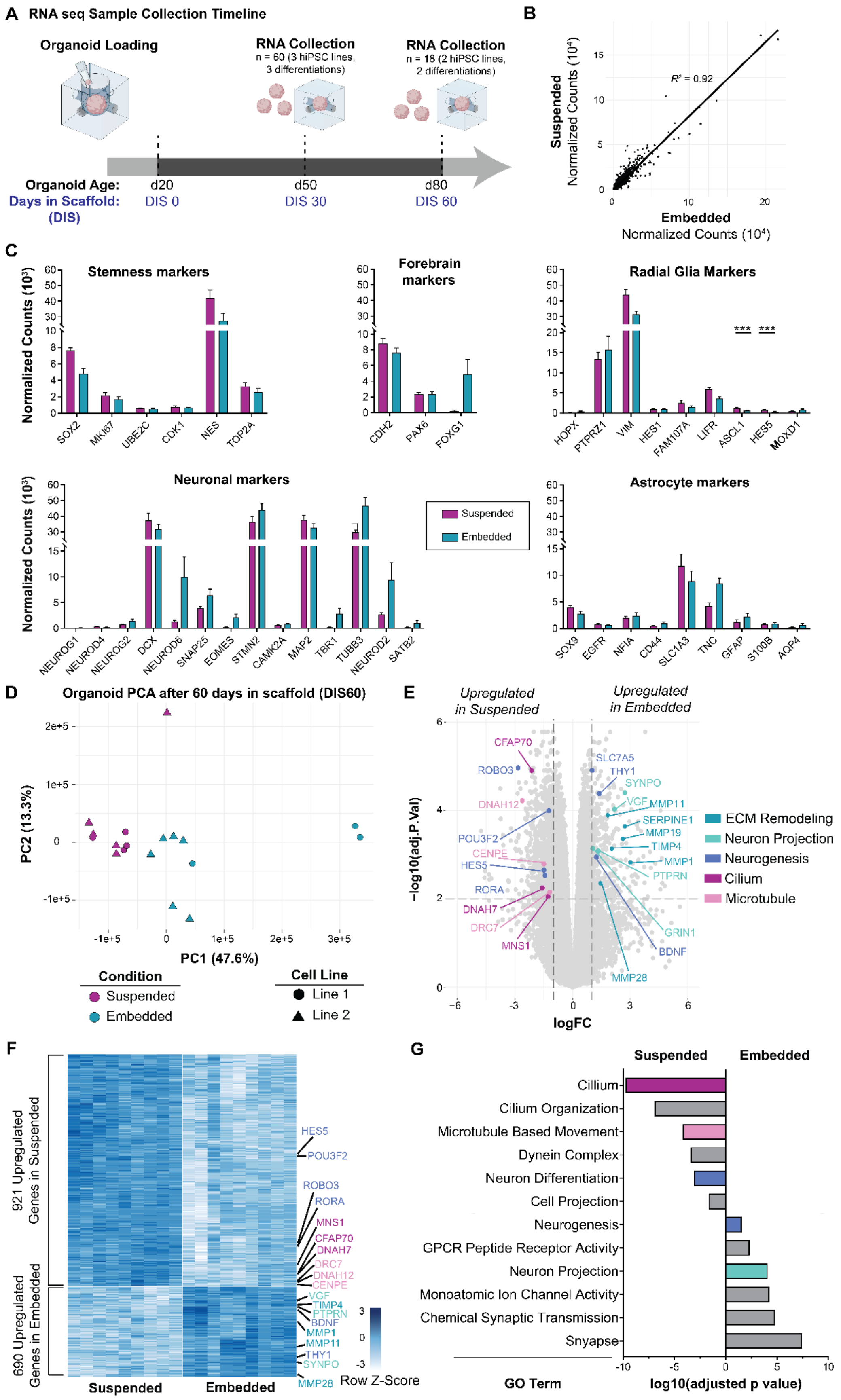

A RNA seq Sample Collection Timeline
Organoid Loading
RNA Collection
n = 60 (3 hiPSC lines, 3 differentiations)
RNA Collection
n = 18 (2 hiPSC lines, 2 differentiations)
Organoid Age:
d20
d50
d80
Days in Scaffold: (DIS)
DIS 0
DIS 30
DIS 60
B
Suspended Normalized Counts (10⁴)
Embedded Normalized Counts (10⁴)
R² = 0.92
C
Stemness markers
Forebrain markers
Radial Glia Markers
Normalized Counts (10³)
SOX2
MKI67
UBE2C
CDK1
NES
TOP2A
CDH2
PAX6
FOXG1
HOPX
PTPRZ1
VIM
HES1
FAM107A
LIFR
ASCL1
HES5
MOXD1
Neuronal markers
Suspended
Embedded
Astrocyte markers
NEUROG1
NEUROD4
NEUROG2
DCX
NEUROD6
SNAP25
EOMES
STMN2
CAMK2A
MAP2
TBR1
TUBB3
NEUROD2
SATB2
SOX9
EGFR
NFIA
CD44
SLC1A3
TNC
GFAP
S100B
AQP4
D
Organoid PCA after 60 days in scaffold (DIS60)
PC2 (13.3%)
PC1 (47.6%)
Condition
Suspended
Embedded
Cell Line
Line 1
Line 2
E
Upregulated in Suspended
Upregulated in Embedded
−log10(adj.P.Val)
logFC
CFAP70
ROBO3
DNAH12
POU3F2
CENPE
HES5
RORA
DNAH7
DRC7
MNS1
SLC7A5
THY1
SYNPO
VGF
MMP11
SERPINE1
MMP19
TIMP4
MMP1
PTPRN
GRIN1
BDNF
MMP28
ECM Remodeling
Neuron Projection
Neurogenesis
Cilium
Microtubule
F
921 Upregulated Genes in Suspended
690 Upregulated Genes in Embedded
Suspended
Embedded
Row Z-Score
G
Suspended
Embedded
Cilium
Cilium Organization
Microtubule Based Movement
Dynein Complex
Neuron Differentiation
Cell Projection
Neurogenesis
GPCR Peptide Receptor Activity
Neuron Projection
Monoatomic Ion Channel Activity
Chemical Synaptic Transmission
Snyapse
GO Term
log10(adjusted p value)

Figure S3: Transcriptomic assessment of embedded and suspended organoids at DIS 60.

(A) Schematic depicting the RNA-seq sample collection timeline. A total of n = 24 samples per condition were sequenced at DIS 30 and n = 9 suspended and n = 9 embedded samples were sequenced at DIS 60. DIS 60 samples include organoids from two different hiPSC lines that are pooled together for analyses. **(B)** Normalized counts of suspended and embedded samples at DIS 60 reveals that there is a correlation in gene expression across the two culture conditions (R^2 = 0.92). **(C)** Comparison of the normalized expression of different neuroectodermal related genes. * p_adjusted <0.05, ** p_adjusted < 0.01, *** p_adjusted <0.001, edgeR, Bonferroni. Error bars represent ± SEM**.** **(D)** Principal Component Analysis (PCA) reveals that the primary variation is a result of culture condition (PC1= 47.6%), followed by cell line differences (PC2 = 13.3%). **(E)** Volcano plot highlighting the upregulated genes in the suspended and embedded samples. Cutoffs are logFC ± 1 and adjusted p. value < 0.01. **(F)** Heatmap demonstrating the differentially expressed genes and highlighting matrix remodeling, neuron projection, neurogenesis, cillium, and microtubule related genes. **(G)** Gene ontology analysis using upregulated genes for suspended and embedded samples. Schematics created with BioRender.com.

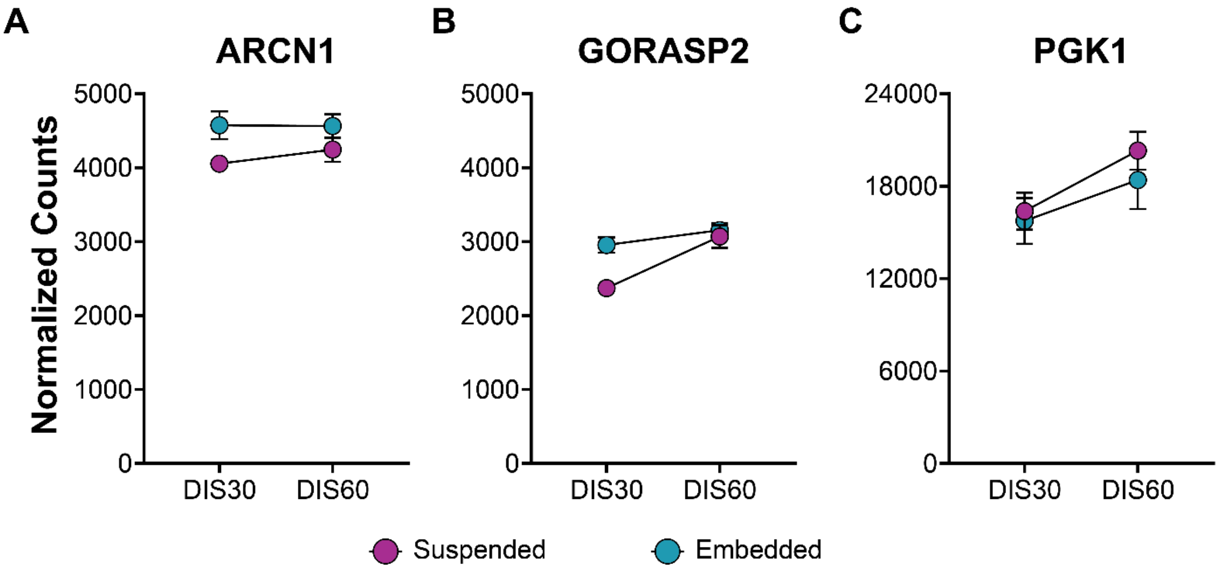

Figure S4: Expression levels of cell stress markers identified in Bhaduri et al. [78] at DIS 30 and 60 in suspended and embedded organoids.

Normalized count data of ER stress genes **(A)** ARCN1 and **(B)** GORASP2, and glycolysis gene **(C)** PGK1 of suspended and embedded organoids at DIS 30 and DIS 60. According to differential gene expression analysis, there is no significant difference in the expression of ARCN1, GORASP2, or PGK1 between suspended and embedded organoids at each timepoint. Error bars represent ± SEM.

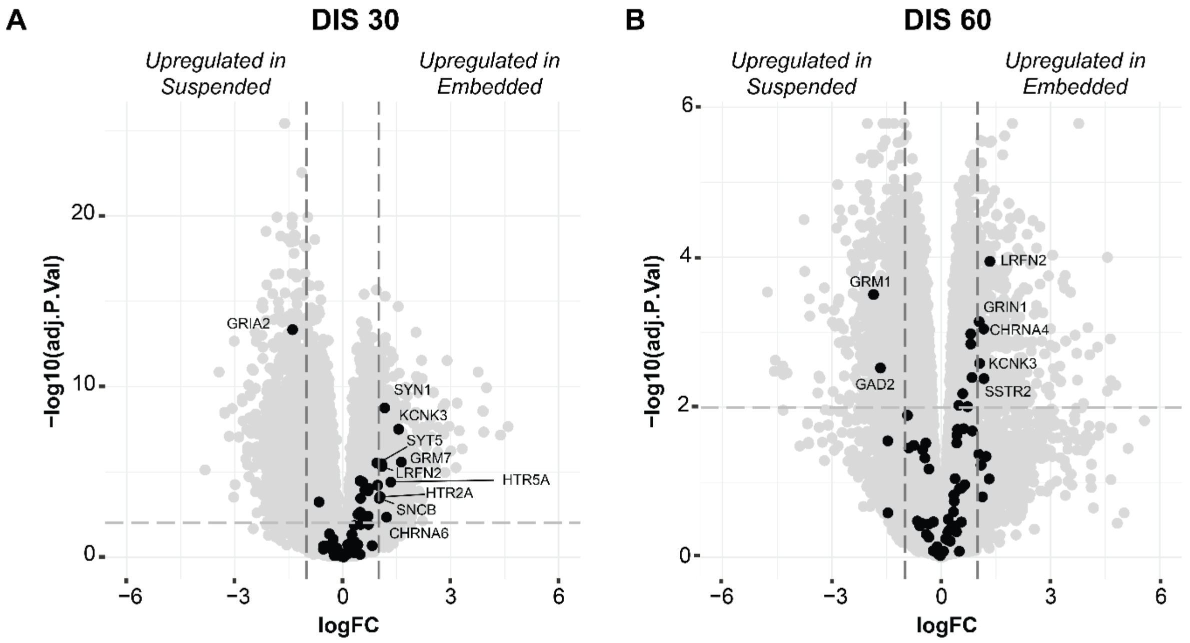

Figure S5: Chemical synaptic signaling gene expression at DIS 30 and 60, using the curated gene list from Simao et. al. [79].

(A) Chemical synaptic signaling genes from Simao et. al. [79] plotted against our differential expression plots at DIS 30 and **(B)** DIS 60.

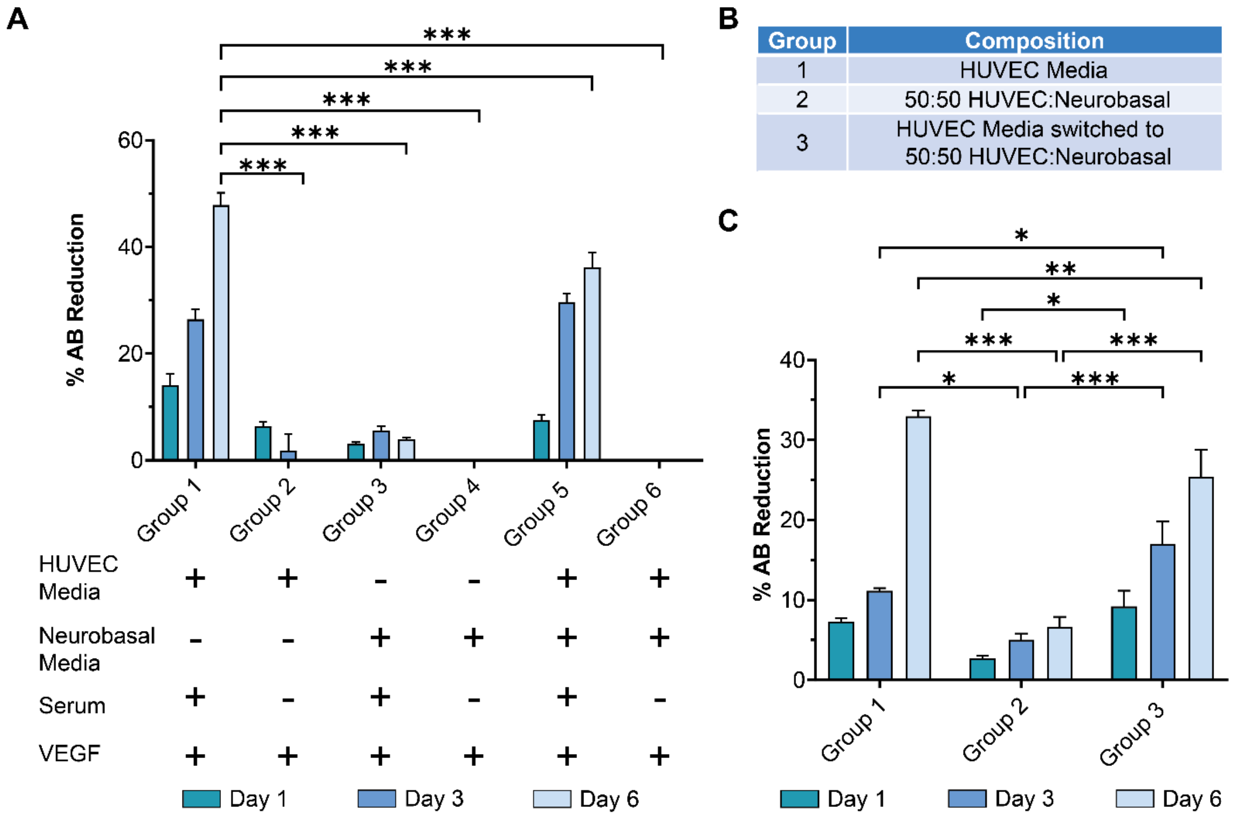

Figure S6: Testing different media compositions to determine optimal media conditions to culture HUVECs in.

(A) Percent of alamarBlue (AB) reduction at day 1, 3, and 6 of culture with different media compositions to elucidate the role of serum on HUVEC viability. A two-way ANOVA with Dunnett's multiple comparison test was run by comparing the mean of the control group (group 1) to the mean of the other groups at each time point. Only significances on day 6 are shown. **(B)** Media compositions to test the effect on HUVEC viability when the cells are allowed to culture in their normal media for a few days before swapping to the 50/50 media mix. **(C)** Percent of alamarBlue (AB) reduction at day 1, 3, and 6 of culture when testing different media conditions. For group 3, HUVECs are cultured in their normal media until day 3 and then the media is switched to the 50/50 media mix. Two-way ANOVA with Tukey's multiple comparison test comparing the mean of each group at each time point. All error bars represent ± SEM. *$p<0.05$, **$p<0.01$, ***$p<0.001$.

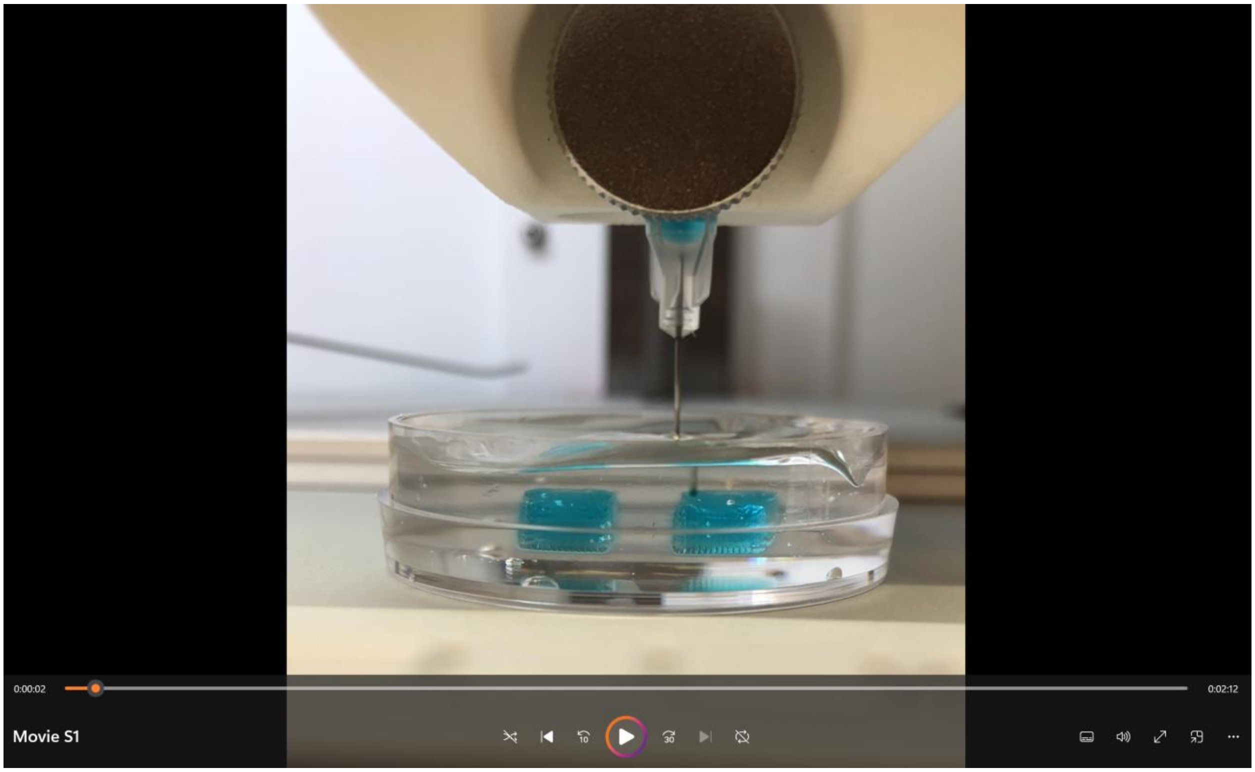

Movie S1: Representative embedded bioprinting process, using GelMA with blue food coloring, to manufacture multiple vascular constructs.

Table S1: Elastic modulus of six randomly selected batches of GelMA crosslinked at distinct UV intensities. Data presented as mean ± SD.

GelMA Batch #	Elastic modulus at 2.5 mW/cm^2 (kPa)	Elastic modulus at 20 mW/cm^2 (kPa)
34	34.67 ± 4.90	54.58 ± 7.37
35	36.9 ± 5.32	56.77 ± 2.84
43	37.53 ± 1.07	54.53 ± 1.07
46	33.65 ± 2.05	50.5 ± 1.74
55	31.50 ± 5.70	60.72 ± 3.86
57	30.84 ± 1.93	55.34 ± 2.20

Table S2: List of Upregulated genes in GelMA Scaffold Embedded and Matrigel Encapsulated Organoids at DIS 30 following differentialy gene expression analysis

Upregulated Genes in Embedded Samples (14)						
geneID	***logFC***	***AveExpr***	***t***	***P.Value***	***adj.P.Val***	***B***
SLC6A5	5.660647806	-2.814026889	9.532527951	2.63E-06	0.001419067	3.842076571
GATA4	4.156676106	-4.184524188	13.44835558	1.09E-07	0.000288331	4.947747173
MYH2	3.636549068	-4.015737874	7.111368551	3.41E-05	0.006824149	1.981360756
FFAR4	3.544348784	-3.078583771	6.917280327	4.30E-05	0.007777532	1.813550139
BEND2	3.404148309	-4.434763215	18.34454766	5.61E-09	4.95E-05	5.705127269
BANCR	3.226068597	-4.174251079	8.265056804	9.36E-06	0.003105957	2.762702449
NRGN	2.919146202	2.025489159	6.695614405	5.63E-05	0.009054543	2.301764517
FLJ33581	2.719225726	-4.662527089	9.697482888	2.25E-06	0.001373419	3.453537436
NWD2	2.389907057	2.118069245	6.59592513	6.37E-05	0.009723669	2.177654696
TNFAIP3	2.367511657	3.230828345	9.702211781	2.24E-06	0.001373419	5.429823686
RELB	2.363323625	3.097790554	7.411854966	2.40E-05	0.005373949	3.103222167
NCR2	2.182167189	-4.842051331	11.7401423	3.90E-07	0.000643782	4.200713301
ABCA12	1.268656836	0.652217277	6.563744363	6.63E-05	0.009849643	2.114610517
TMEM179	1.216986669	3.985179882	8.447333063	7.72E-06	0.002695753	4.184931125

Upregulated Genes in Matrigel Samples (91)						
geneID	***logFC***	***AveExpr***	***t***	***P.Value***	***adj.P.Val***	***B***
C6	-4.830145081	-0.493351455	-8.444085861	7.75E-06	0.002695753	3.432464877
COL20A1	-2.592142592	1.615350563	-7.976103549	1.28E-05	0.00366626	3.581736323
HSF4	-2.474679426	3.631213066	-10.3912348	1.20E-06	0.001078272	5.987888002
LMNTD2	-2.435003558	3.55139538	-8.687680163	6.03E-06	0.002378893	4.463312612
STRC	-2.35910925	2.13028585	-7.356123371	2.56E-05	0.005542631	3.034064358
LOC101929380	-2.211427848	-4.874601633	-10.28576791	1.32E-06	0.001078272	3.655656932
LINC00894	-2.160864228	3.203894597	-9.682933872	2.28E-06	0.001373419	5.374719713
LOC388849	-2.124415696	0.509161734	-7.666083551	1.80E-05	0.004482274	3.105004328
GOLGA8A	-2.062997386	7.111924495	-11.64351014	4.21E-07	0.000654304	7.044360507
UNC13D	-2.061556694	2.626823161	-6.618667338	6.19E-05	0.009518404	2.209065026
PDIA2	-2.013349206	3.632086616	-7.977289918	1.27E-05	0.00366626	3.736517161
WSB1	-1.997133969	9.438725933	-14.98604715	3.91E-08	0.00014752	9.431356664
ADCY10P1	-1.973564869	2.061429887	-7.487553642	2.20E-05	0.005191626	3.169225626
MSH5-SAPCD1	-1.927320959	0.868092599	-8.004612671	1.24E-05	0.00363333	3.490042079
FTCD	-1.924524925	1.346299294	-8.232349737	9.69E-06	0.003105957	3.812546311
MUC1	-1.907648725	2.561771601	-8.236369237	9.64E-06	0.003105957	3.981886012
JAK3	-1.805947215	2.696954245	-7.084338175	3.52E-05	0.006938078	2.755103953
COL7A1	-1.779958377	5.084469858	-8.138721999	1.07E-05	0.003357437	3.829290905
LOC101927248	-1.777090208	1.123509746	-10.00143333	1.70E-06	0.00125045	5.189772007
AP1G2	-1.729629324	4.43482614	-7.711769267	1.71E-05	0.004438689	3.397670483
GOLGA8B	-1.712695123	6.067928782	-16.72940951	1.36E-08	6.44E-05	10.42467907
LOC100288152	-1.702773114	4.229132078	-6.736357336	5.35E-05	0.008809753	2.245628977
KBTBD11-OT1	-1.701694744	0.37324515	-7.63386931	1.86E-05	0.004561384	3.074371637
LOC100131564	-1.668334571	4.947648162	-7.86881426	1.44E-05	0.003962045	3.51844698
PABPC1L	-1.660375346	3.446285098	-7.433985946	2.34E-05	0.005373949	3.127171649
CCDC88B	-1.637460978	2.757246386	-6.737291025	5.35E-05	0.008809753	2.347598115
UBA7	-1.627908356	1.725902153	-7.472879142	2.24E-05	0.005211369	3.141804354
DERL3	-1.614730433	2.90796571	-6.989150457	3.94E-05	0.007283125	2.639967285
KIAA1875	-1.611095555	2.156601843	-9.131433997	3.87E-06	0.001763794	4.771272958
NRBP2	-1.604792559	6.598953899	-12.04421541	3.07E-07	0.000549418	7.370985333
LOC102724814	-1.566610424	2.013853086	-7.30541853	2.71E-05	0.005736962	2.984034508
LENG8	-1.558286836	7.914817769	-10.22942865	1.39E-06	0.001078272	5.793661856
TMPRSS6	-1.554409071	1.153096993	-6.567867466	6.60E-05	0.009849643	2.109118737
LINC01001	-1.551279522	0.784211145	-7.01555103	3.82E-05	0.007106284	2.560460832
MAMDC4	-1.550363511	4.379256852	-12.84084375	1.69E-07	0.000405103	7.926937964
AMY2B	-1.53761885	4.391581175	-10.88003987	7.88E-07	0.000867957	6.45820255
SLC9A3	-1.530954775	3.161646902	-6.904350764	4.36E-05	0.007846203	2.526072586
GSDMB	-1.529498911	4.485423801	-11.20160628	6.02E-07	0.000758166	6.721507813
CCDC78	-1.515926017	2.898075499	-7.019828097	3.80E-05	0.007106284	2.673452506

AGER	-1.508227205	3.654492243	-10.23592565	1.38E-06	0.001078272	5.888413401
TMEM154	-1.499493089	-0.935430694	-7.139015253	3.30E-05	0.006703257	2.371324034
LOC100505915	-1.494833013	0.415993317	-6.818014158	4.85E-05	0.008426482	2.298640662
ZNF83	-1.486462616	5.992586436	-17.39577215	9.37E-09	6.19E-05	10.77923743
KAT2A	-1.485145038	6.224388001	-12.24558468	2.63E-07	0.000534826	7.535584225
CCDC154	-1.480855739	2.115516665	-6.84572627	4.68E-05	0.0082009	2.475401462
AOC3	-1.471524744	2.757477915	-8.600229126	6.59E-06	0.002490116	4.348232488
MAN2C1	-1.469951968	6.323197391	-16.60855098	1.46E-08	6.44E-05	10.37491697
CSAD	-1.453563955	5.417022477	-9.195178595	3.64E-06	0.001739466	4.896780702
KCND1	-1.452079589	3.13362525	-10.23996781	1.37E-06	0.001078272	5.848103261
PAQR6	-1.44805088	4.047989345	-8.924152705	4.75E-06	0.00196199	4.695792261
MST1	-1.43269265	3.804430108	-7.038851341	3.71E-05	0.007106284	2.648793859
PNPLA7	-1.427457545	2.240415297	-9.050919463	4.19E-06	0.001845443	4.721975183
CATSPERG	-1.423186202	3.638467325	-9.567125137	2.55E-06	0.001402058	5.30492324
ASIC3	-1.420645738	3.596316181	-20.29738817	2.11E-09	3.43E-05	11.33043506
LOC283710	-1.409497751	-0.626635603	-6.685280152	5.70E-05	0.00908082	1.964464855
TMC4	-1.3994022	1.060276998	-8.754313722	5.64E-06	0.002256819	4.239704523
COL11A2	-1.390049745	4.440401993	-10.52468105	1.07E-06	0.001046733	6.162389759
CCDC183	-1.385725128	3.028540718	-7.684850151	1.76E-05	0.004438689	3.424552115
LINC00471	-1.355338419	1.788603576	-7.028935745	3.76E-05	0.007106284	2.677713338
MST1P2	-1.320778047	1.791845322	-6.74099679	5.32E-05	0.008809753	2.34778008
RNF139-AS1	-1.302611304	2.000242203	-6.725562143	5.43E-05	0.008854202	2.334096072
NSUN5P1	-1.293356296	4.527676143	-19.86683514	2.60E-09	3.43E-05	11.61752257
LINC01089	-1.293114744	4.847509292	-12.28860217	2.55E-07	0.000534826	7.570961142
UNC5CL	-1.292073425	2.152338216	-7.388223986	2.47E-05	0.005475706	3.085302128
CAPN10-AS1	-1.265680543	2.786536684	-8.130715193	1.08E-05	0.003357437	3.888790224
NSUN5P2	-1.263968062	2.896223385	-10.11313115	1.54E-06	0.001162542	5.728061013
PIGL	-1.248907539	3.456859738	-7.904710996	1.38E-05	0.003921065	3.654127017
NEIL1	-1.242241569	3.184531236	-7.468734308	2.25E-05	0.005211369	3.182134082
ITIH4	-1.241371581	3.595641053	-7.866336912	1.44E-05	0.003962045	3.608349522
AHSA2	-1.20862177	6.028341309	-7.892597755	1.40E-05	0.003931241	3.445930671
LENG8-AS1	-1.206551573	2.937355522	-7.323704555	2.66E-05	0.005707819	3.022748378
YJEFN3	-1.1944833	3.989696101	-8.980814858	4.49E-06	0.001914223	4.75293434
NSUN6	-1.149166096	4.865273492	-10.62260881	9.82E-07	0.000998479	6.241557519
KCNAB3	-1.145779794	3.241106166	-8.508355266	7.25E-06	0.002589735	4.285669653
MAPK12	-1.140957788	3.787761621	-6.734689083	5.37E-05	0.008809753	2.272022584
ECM1	-1.140944795	2.654615143	-7.229400513	2.96E-05	0.006087583	2.920166744
CCDC159	-1.132108331	2.651037418	-6.739563256	5.33E-05	0.008809753	2.346056677
EXD3	-1.121147312	4.267990879	-9.80681385	2.04E-06	0.001345156	5.528618098
PTCH2	-1.107640857	3.07006098	-8.579924493	6.73E-06	0.002501616	4.355601454
LOC401320	-1.104685872	4.240287513	-12.02464521	3.12E-07	0.000549418	7.349609348
SERHL2	-1.101672451	3.503665233	-7.644022211	1.84E-05	0.004551747	3.36726627
MAPK13	-1.098651305	2.655793736	-6.692519428	5.65E-05	0.009054543	2.292451289
HDAC10	-1.093467544	4.687548577	-9.842194997	1.97E-06	0.001335337	5.550316357
SGK494	-1.088735943	5.35005494	-11.29322949	5.59E-07	0.000758166	6.795176735
PRRT2	-1.074371089	6.721535679	-11.04997216	6.83E-07	0.0008208	6.548777408
PPP1R3E	-1.065875084	4.884515918	-6.640013412	6.03E-05	0.009433841	2.02160148
PAGR1	-1.048744281	6.160636453	-8.07724159	1.14E-05	0.003474349	3.648689376
OSBPL7	-1.042918716	4.557487483	-11.20734355	5.99E-07	0.000758166	6.730439349
HNRNPU-AS1	-1.036194719	5.858512467	-7.273802656	2.81E-05	0.005904671	2.731758921
LINC00632	-1.02732057	4.632571076	-6.766110593	5.16E-05	0.008809753	2.216301724
RRP7BP	-1.009722419	3.779343129	-6.8792581	4.50E-05	0.007979542	2.446583413

Table S3: List of Upregulated genes in Suspended and Embedded Samples at DIS 30 following differential gene expression analysis

Upregulated Genes in Embedded Samples (579)						
geneID	***logFC***	***AveExpr***	***t***	***P.Value***	***adj.P.Val***	***B***
SLC6A2	4.595610949	3.343557476	7.34379185	9.59980059639496e-10	2.2219923744703e-08	12.0527789
UTS2	4.387287974	-1.42640891	6.984021998	3.76285962027365e-09	7.00941989730045e-08	10.78331952
PRRG2	3.995476334	0.712179608	9.023163958	1.72656421725029e-12	1.17021210139941e-10	18.14633681
DBH	3.920324505	3.194091241	7.11331705	2.30325930088055e-09	4.67604094471394e-08	11.19100408
KRT80	3.905244357	-2.178515812	7.979539301	8.62657147064649e-11	2.84830225885504e-09	14.36348382
CXCL8	3.892507609	-2.785155313	7.999223992	8.00816236486974e-11	2.69655739860639e-09	14.426504
FOLR3	3.763705501	-2.494714093	9.709870801	1.38191863733704e-13	1.41035734906294e-11	20.42621787
GAL	3.322019657	-0.865111526	6.412626646	3.27464394364674e-08	4.47562892256537e-07	8.716655792
GRP	3.239871502	-0.099931134	7.168219948	1.86983073965862e-09	3.92263777312669e-08	11.44281406
GPR50	3.201509909	1.793818118	4.487821204	3.65299051175388e-05	0.000193817	1.91936516
EGR4	3.180674881	-1.227363027	8.341187886	2.20643703643676e-11	9.43733821749722e-10	15.63919635
MMP1	3.147864271	-1.472693312	5.616939027	6.4069691841662e-07	5.72676152309951e-06	5.894533164
FGF3	3.003692271	-1.31922753	6.319640341	4.64951747937607e-08	6.0274985547988e-07	8.381729524
PRLHR	2.956395444	-2.690494056	7.353585229	9.24940201399984e-10	2.14841338696009e-08	12.08163384
ADRA1B	2.9008844	1.009229252	10.22595348	2.13819907314864e-14	2.9904241323036e-12	22.39123311
NKX6-2	2.844818011	-0.072031398	5.414896055	1.34623563585589e-06	1.09425112431054e-05	5.181716831
RELB	2.842033962	3.218405794	8.315649441	2.42886232181788e-11	1.02232671580592e-09	15.63775065
MS4A3	2.810937131	-2.135120561	7.018681002	3.29900402626844e-09	6.27562386260089e-08	10.88150802
MC5R	2.785775069	-2.327040564	8.164427122	4.29270678206808e-11	1.58807934829844e-09	14.97602203
BCYRN1	2.76968644	5.585369173	7.206354109	1.61775359043758e-09	3.47942072058882e-08	11.31522193
TMEM156	2.65004046	-2.562681657	7.318397935	1.05714971723996e-09	2.41982447802205e-08	11.94796885
SYK	2.636944692	-1.721915078	6.282879994	5.33975478444197e-08	6.81534226060622e-07	8.247290372
TNFRSF9	2.570759162	-1.339801048	5.627457182	6.16297389136766e-07	5.53724979165606e-06	5.933775094
VGF	2.522046236	7.802983914	8.799121324	3.97188719316772e-12	2.2724868869481e-10	17.14566913
MYH2	2.466571838	-3.685792843	6.901306856	5.15034381734334e-09	9.07593587492244e-08	10.4611241
TIMP4	2.442160319	-0.986362639	7.671090499	2.77283796365741e-10	7.85577983851623e-09	13.22538168
NEURL3	2.410178435	-1.605830046	7.407706437	7.53146486051142e-10	1.81807498317715e-08	12.27265305
CD300E	2.364587135	-3.66382198	5.898539916	2.25399934850971e-07	2.33098453752571e-06	6.885075172
OR2W3	2.350404147	-2.358257549	6.832514461	6.68587856654663e-09	1.13359735823943e-07	10.20328375
TH	2.330339698	5.14519957	6.500808421	2.34727862648904e-08	3.36655539522436e-07	8.699606109
IL31RA	2.268182159	-1.129675414	7.058503066	2.83613916967743e-09	5.5531604942284e-08	11.02241906
ARHGAP36	2.239235724	-0.993928262	3.799797294	0.000360483	0.001432017	-0.040999082
RAMP1	2.238062622	0.168059633	5.778822787	3.51869296272751e-07	3.42703062209934e-06	6.45019925
NGB	2.236677199	0.716911496	7.318324144	1.05744595941726e-09	2.41982447802205e-08	12.01121018
NKX2-2	2.218012893	-2.760672319	3.189977289	0.002336778	0.007359474	-1.728946704
KRT75	2.210705857	-3.265377177	4.253686321	8.10953673744162e-05	0.000390028	1.358129903
TMIE	2.210212904	0.431137501	9.426404455	3.89765363281357e-13	3.4457083102395e-11	19.56394504
PHOX2A	2.205619252	3.063578218	4.352409085	5.80606970425573e-05	0.000290887	1.30845913
ADRA2A	2.193770531	3.885765609	10.23633215	2.06006430161059e-14	2.92761718733724e-12	22.57180724
STYK1	2.138372351	0.467589093	7.830836054	1.51395729413772e-10	4.56310526293527e-09	13.87079135
EGR2	2.121423295	0.299022749	4.602403032	2.45755105763427e-05	0.000137482	2.404452172
TRIM58	2.118763472	1.454972939	8.901062802	2.7173455641328e-12	1.6588359190929e-10	17.78304012
DRD5	2.113297299	-2.796790283	6.703608536	1.08978783925413e-08	1.73636901476821e-07	9.734007344
NPFFR2	2.10225746	-2.015189091	5.547870439	8.26423928780013e-07	7.16930216916379e-06	5.659320979
TTC39A	2.102143734	1.108974995	8.795355332	4.02804122721972e-12	2.28047806561277e-10	17.38625687
SUSD5	2.097862249	1.974071962	7.120533053	2.24100753967891e-09	4.57425114257394e-08	11.26389768
OSR2	2.079930886	-3.018484991	4.306240107	6.79077134201857e-05	0.000333272	1.529794755
BHLHA15	2.078761183	-1.960341277	7.169592091	1.86011337577193e-09	3.90535161729781e-08	11.40757964
SYTL5	2.063313294	0.802443996	5.138756041	3.6715824375081e-06	2.61875171534408e-05	4.17994219
FFAR4	2.059580869	-1.93318147	6.184581864	7.72754496750786e-08	9.28886749095658e-07	7.893256364
SLC18A3	2.05695871	1.922736771	9.11328217	1.2364289330052e-12	8.92965190877775e-11	18.56316315
FAM189A2	2.050664462	0.765909788	9.51379188	2.82881649773056e-13	2.57841746498317e-11	19.9088632
TMEM179	2.037141139	4.798527693	11.53334822	2.17320730399504e-16	6.67958007750009e-14	27.0459662
TG	2.035945097	-0.143651632	7.940754437	9.98876091931306e-11	3.21565366582857e-09	14.23389963
SYNDIG1L	2.029988829	0.104999551	5.850017716	2.70048791993773e-07	2.72242552203334e-06	6.703936803
MIR4754	2.029307081	-2.440016409	5.869720727	2.50949080152675e-07	2.55521457460542e-06	6.781529346
PTHLH	2.008323685	-1.351541682	3.747361099	0.000426093	0.001657774	-0.185301223

HTR6	2.007426793	-0.85554748	7.918139464	1.08805743771492e-10	3.45265573242718e-09	14.09724468
UGT3A2	1.99831583	-2.609953793	5.154734126	3.46589794501199e-06	2.48546067228709e-05	4.313076383
GPNMB	1.996747016	2.9349078	6.027356391	1.39324199772301e-07	1.53832772455356e-06	7.166601246
RAMP3	1.973373437	-3.374608137	4.352050785	5.81314830799971e-05	0.000291131	1.665538405
DUSP27	1.970624064	-3.326365053	6.278142111	5.43583012868422e-08	6.90131113311768e-07	8.22601534
P3H2	1.969512218	1.229774112	7.484677137	5.62341622500029e-10	1.42379081489878e-08	12.62239217
KCNK12	1.969022739	1.456136173	6.330945901	4.45565435327453e-08	5.83051047129235e-07	8.386118426
FAM83G	1.967772531	1.231891919	6.346404812	4.20352500545915e-08	5.5500387846804e-07	8.45278703
ATP10A	1.959542042	-0.737237942	5.58478816	7.21359444770164e-07	6.3537801411562e-06	5.785512192
APOL6	1.957734817	-1.013292977	5.158499137	3.4191072521726e-06	2.4558130261532e-05	4.320090461
MMP8	1.943362731	-3.464231038	5.068532186	4.72736144051833e-06	3.25666783834301e-05	4.015454938
CLEC2L	1.935988416	1.365592617	7.637979456	3.14369960855753e-10	8.74709597400012e-09	13.1845382
C14orf105	1.925564953	-1.072576522	4.356139943	5.73285886174792e-05	0.0002876	1.676150681
GABRA5	1.912103168	3.059308524	5.807820582	3.15936335820821e-07	3.12075678802383e-06	6.35407218
GATA4	1.911361821	-3.24669132	3.390607662	0.001287758	0.004398981	-1.192973176
DRGX	1.908132598	-0.567936181	3.70256625	0.000491075	0.00187825	-0.339829867
GALR1	1.900637408	-0.997768163	5.300653717	2.0424042545518e-06	1.57167020845903e-05	4.805757277
KL	1.897316504	-2.628738814	5.058749028	4.89637231316542e-06	3.36258273197978e-05	3.989834319
DMBT1	1.896991863	-3.260571904	5.703253734	4.65664406638449e-07	4.35251317562735e-06	6.199785108
MMP19	1.891474805	-1.043414891	4.797144183	1.24247725867991e-05	7.62358434974144e-05	3.108052659
CD28	1.888068948	-2.711941674	5.043564583	5.17052683634111e-06	3.53159007403113e-05	3.93910714
COL8A1	1.884677978	-1.917704916	3.581225726	0.000718211	0.002630421	-0.655806007
IGFBP3	1.858493997	2.9906768	6.622338679	1.48243909843887e-08	2.26112594858826e-07	9.349922043
PPAP2C	1.855922566	1.507039465	5.372665069	1.57092229922599e-06	1.24884779354708e-05	4.94181132
SRPX2	1.855128658	-1.273950829	4.169842646	0.00010743	0.000497322	1.094390654
SLC5A10	1.836117056	0.145337149	6.049559704	1.28214897539976e-07	1.43727921402637e-06	7.41380498
NIPAL2	1.830965997	-0.334967895	5.436894565	1.24208095332752e-06	1.01994177817044e-05	5.263354145
PTGER4	1.828093696	-1.078149194	5.326479271	1.85912808576495e-06	1.45219659252437e-05	4.894844813
SOSTDC1	1.828004297	1.949736048	6.681739724	1.18389526539595e-08	1.86495253576943e-07	9.644018055
RBP4	1.824389623	0.618332004	4.993683812	6.18163324224832e-06	4.12207647558905e-05	3.692777701
C1orf158	1.822385097	-1.023073818	4.875357416	9.42147974591186e-06	5.96784026177063e-05	3.368456404
REM1	1.812266664	-3.163976999	3.478044762	0.000987228	0.003486825	-0.946335337
LGI3	1.81111781	2.38992835	5.818047923	3.04150814291948e-07	3.02241296021769e-06	6.454700608
EGR3	1.809549586	0.732469913	4.773251626	1.35165155613136e-05	8.18515591826352e-05	2.939543637
PHF24	1.795466953	5.047149531	8.655423999	6.79164626815122e-12	3.4994851034316e-10	16.76107126
FAM131C	1.795364134	2.408949786	7.670275148	2.78142138447801e-10	7.87166075545045e-09	13.27791231
TPBGL	1.791542888	1.521830551	6.367550043	3.88149139723674e-08	5.18928137110184e-07	8.514981654
PTGER2	1.78461393	-2.552051793	6.249492824	6.05452511004812e-08	7.56329216606343e-07	8.117223513
NR0B1	1.784131949	-0.824548246	6.594680994	1.64598821446205e-08	2.47206854959519e-07	9.358311865
EGR1	1.781719076	5.273758609	5.082208383	4.50067279150652e-06	3.12329440530039e-05	3.507135374
ME1	1.7808865	1.736350107	6.738552728	9.54656001785854e-09	1.54150409866863e-07	9.864037208
SNORD116-29	1.778007185	-3.614985677	5.02603377	5.50584448859652e-06	3.72261792834942e-05	3.869466202
OOSP1	1.771967894	-3.779798242	4.440494963	4.29792207501816e-05	0.000224077	1.935203225
MAFA	1.765576943	1.116723171	4.504036203	3.45452811647085e-05	0.000184509	2.014072177
PTPRN	1.753635854	6.983929646	9.03729013	1.6384428042187e-12	1.12783746468523e-10	18.05601037
MYLK4	1.752015997	0.124041634	8.218442073	3.50205898541663e-11	1.34745160351554e-09	15.25241486
P4HA3	1.747176852	-2.390521968	4.478532751	3.77163747201896e-05	0.000199511	2.081106748
BCAN	1.746703691	4.734491942	5.036172212	5.30940231930434e-06	3.60687307905864e-05	3.407609049
CHRM4	1.741162042	1.547484674	6.151713473	8.74253493177114e-08	1.03442894293423e-06	7.72728378
GOLT1A	1.738178612	0.088534787	5.248125838	2.47187207602167e-06	1.85358849887889e-05	4.594347648
KCNS2	1.736839742	2.114201213	6.773697206	8.35609308905106e-09	1.37446551725505e-07	9.97284823
LUCAT1	1.735670093	-3.316081834	4.070543851	0.000149414	0.00066344	0.789033815
TRIM47	1.734895836	1.830269881	6.065313032	1.20870685249015e-07	1.36654184054201e-06	7.395031763
CD300LB	1.732805579	-2.721942343	4.558882673	2.85812719325781e-05	0.000156157	2.34186694
TMEM119	1.724101888	-1.338516651	3.360304172	0.001410811	0.004755057	-1.27615313
SIX2	1.720835529	2.001158781	4.12747532	0.00012372	0.000563163	0.707505011
UBD	1.717368547	-3.01101879	3.976002794	0.000203865	0.000871261	0.511236677
SERPINE1	1.710255315	1.298607599	4.471663027	3.86179625573748e-05	0.000203832	1.890569305
SYT17	1.707520312	3.604280267	8.752497691	4.72620841631566e-12	2.60809743357979e-10	17.2189659
BPIFC	1.705232611	-2.257673214	4.718575382	1.63804403351519e-05	9.65835778226791e-05	2.859314163
KCNK13	1.703432674	-0.946312805	4.917116394	8.12257181546022e-06	5.24435615041671e-05	3.507653379
MSC	1.696370928	-1.364127206	3.919247296	0.000245274	0.001026878	0.332779899
SERPINA3	1.696342031	-0.916076184	4.01298427	0.000180603	0.000783503	0.608478992

CYB561	1.691541339	5.324168337	8.227563806	3.38376822705514e-11	1.30922396785407e-09	15.14244752
NWD2	1.69077119	3.17328101	5.366887306	1.60440966392358e-06	1.27050211643176e-05	4.756436172
NPSR1	1.679375591	-3.871189814	5.12995061	3.7900203566848e-06	2.69377811476874e-05	4.216469791
PAX1	1.679342356	-1.972122501	4.095912585	0.000137384	0.000616757	0.876433295
FAM163B	1.676366814	2.629818659	5.508589691	9.54845352216154e-07	8.11035578249666e-06	5.318730224
LINC01512	1.670737857	0.19211402	6.950591536	4.27188764480517e-09	7.7341648024065e-08	10.6660853
PTPRH	1.656229909	2.533788544	6.307164027	4.87320868276404e-08	6.28052292108737e-07	8.223393722
SLC10A4	1.651640071	2.268717811	7.096262864	2.45734030420207e-09	4.93953431642382e-08	11.15635464
C1QL2	1.646897124	0.087056116	6.153575842	8.68163773233947e-08	1.02906605461403e-06	7.786935341
MCOLN3	1.643597834	-3.13072492	3.374250075	0.001352858	0.004588158	-1.232311647
GPR83	1.641671506	1.608565702	6.336948635	4.3560127527715e-08	5.71994461470487e-07	8.397547853
SLC6A17	1.640180485	5.504972039	7.840203862	1.46121373685388e-10	4.43447333022487e-09	13.67450827
KLKP1	1.639729039	1.153579212	4.729482976	1.57653189592001e-05	9.34779443805597e-05	2.757210774
RASAL3	1.637332166	-1.059492973	4.892213989	8.87431278929673e-06	5.66058663029634e-05	3.426702493
AQP5	1.634206172	-1.01679407	4.838805328	1.07241013713004e-05	6.69035099239044e-05	3.24857853
CD300C	1.633214431	-2.776755122	5.111053648	4.05703060249751e-06	2.85515148870651e-05	4.167288195
TMEM132E	1.632269291	4.495645713	5.610594481	6.55874227219471e-07	5.84515288202707e-06	5.475098371
GRM7	1.632094408	3.188972746	5.854985607	2.65101294836224e-07	2.67867833578207e-06	6.508532789
HRH1	1.631130953	-0.277004819	7.71209136	2.37375801797068e-10	6.8951149108812e-09	13.40360632
KCNA1	1.629843026	1.715480378	5.30598163	2.00319021273841e-06	1.54554369215745e-05	4.687996066
GABRG3	1.629513653	1.377933835	4.941480738	7.44768465986555e-06	4.8572575527813e-05	3.454707507
KCNS1	1.62467887	-0.472009202	4.108823288	0.000131627	0.000594445	0.875510756
TREML3P	1.618996098	-4.467405062	4.83793415	1.075720955e-05	6.70625754799884e-05	3.214187409
SLC7A4	1.612209753	0.241329848	4.761816136	1.40717610101714e-05	8.47479742041152e-05	2.933853161
BOK	1.606071233	3.607225725	7.771859555	1.89276989190379e-10	5.59637433475311e-09	13.58071949
CITED1	1.597365511	4.120005791	4.429484813	4.46316586756075e-05	0.000231732	1.414377284
CARTPT	1.597154905	0.360273258	3.157880889	0.002565706	0.007978742	-1.946424843
CNN1	1.593324029	1.624496524	5.294733129	2.08686869404036e-06	1.60076030730031e-05	4.656003742
GSC2	1.592688344	-3.814533609	4.58652517	2.59690099973762e-05	0.000144028	2.406640694
MARCH11	1.586775183	0.344988908	5.550721234	8.17798241841685e-07	7.10146548180067e-06	5.63712512
HMOX1	1.58654446	3.753552942	4.142788722	0.000117574	0.000537779	0.531608243
MAGEB17	1.583510673	-3.424606799	3.942926105	0.000227097	0.000960611	0.397015101
PTRF	1.582643857	2.942591561	5.106613415	4.12240718788546e-06	2.89345696222455e-05	3.865283183
LINC01561	1.581670989	-2.343715216	4.48386751	3.70304193804977e-05	0.000196079	2.100362891
LRRC2	1.580653743	-0.869235809	4.560714472	2.84005034309961e-05	0.000155362	2.331843687
ITPKA	1.576292716	0.559165227	6.11701393	9.95804082549352e-08	1.15905280995275e-06	7.645280729
OTOP2	1.576196931	-3.26412887	4.672213633	1.92675097366971e-05	0.000111298	2.701055125
FAM159A	1.574113154	-0.540573377	5.008286157	5.86705860740105e-06	3.94214438661494e-05	3.800828326
PPAPDC1A	1.570273005	-0.190457438	9.655487845	1.68500961554301e-13	1.65575684638098e-11	20.27714473
NPBWR1	1.570180789	-3.113738696	4.018708572	0.00017724	0.000771571	0.637734644
SV2C	1.566314215	4.216170063	7.227746412	1.49153157367989e-09	3.25294175635979e-08	11.49140666
LINC00941	1.566289417	-3.699754587	4.373054198	5.41205716031348e-05	0.000273688	1.722522829
GPR68	1.566207099	2.071738656	8.133794193	4.81836235747895e-11	1.75432193106393e-09	14.99888706
LOC100126784	1.563409522	1.604124359	7.243669697	1.40401603183001e-09	3.09012121310265e-08	11.72905647
LCAL1	1.562159643	-4.21572699	4.077390723	0.00014607	0.00065067	0.773560613
KLHDC7B	1.560447743	-0.183019176	4.218390255	9.1313951445386e-05	0.000431172	1.19791429
APOF	1.560038178	-2.768361003	5.250878232	2.44730787105066e-06	1.83865991203758e-05	4.641220812
MALL	1.558438443	-4.268662793	4.902639952	8.55161757405992e-06	5.48785888164909e-05	3.437990473
LKAAEAR1	1.555840869	-0.546034005	4.386027887	5.17789462416841e-05	0.000263308	1.752586042
KCNK3	1.555338546	5.554677519	7.233770991	1.45779618573701e-09	3.19253741322174e-08	11.38734704
HKDC1	1.552913108	0.030890162	4.023766423	0.000174318	0.000760983	0.576340818
WBSCR17	1.544376794	5.280037443	12.77520169	3.38850195713954e-18	2.03564255075158e-15	31.15579769
DYSF	1.536499345	0.303056793	4.386052489	5.17746009352227e-05	0.000263308	1.696619689
TMEM151A	1.535495622	4.624900635	6.818950461	7.03875924066873e-09	1.1828068849879e-07	9.91879195
ITGA11	1.533067428	0.564724312	3.567287092	0.000749961	0.002734682	-0.833256765
KCNE4	1.531382886	-1.620625245	3.293772643	0.001721119	0.005652174	-1.448571734
GALR2	1.527190705	-1.220347388	4.286474001	7.26031100935852e-05	0.000353234	1.464889981
OPRD1	1.515971872	-2.405887136	4.493296059	3.58476948690673e-05	0.000190733	2.13218682
ADAP1	1.515651863	5.485283962	8.083905391	5.81632762975486e-11	2.0363309700306e-09	14.58796258
KIAA0226L	1.501960697	0.681336635	7.13855163	2.09280733348666e-09	4.31506834992613e-08	11.35500417
HOXD9	1.50166881	-1.457206512	3.779585977	0.000384533	0.001514583	-0.077169881
HAS1	1.49444078	1.643357759	5.623018228	6.26480483867248e-07	5.61728583109327e-06	5.814296629
TNFRSF12A	1.494233151	3.53930243	5.19733699	2.97130570055218e-06	2.17202775394623e-05	4.111763467

HTR7	1.491359263	-2.171220557	4.077156999	0.000146183	0.000651063	0.824666999
B4GALT1-AS1	1.489765693	-1.512349542	4.421936861	4.58000513512749e-05	0.0002371	1.897286908
SBF1P1	1.487594633	-2.344268198	3.806596968	0.000352721	0.001405833	0.01327834
LINC01127	1.485110261	-3.430717886	3.887263684	0.000272064	0.00112314	0.229626104
GCH1	1.482599722	2.187290712	6.497142675	2.38001467439844e-08	3.40242984793802e-07	8.946787255
ADGRF4	1.479232618	-2.332352085	3.364929514	0.001391334	0.004700555	-1.242164687
IL32	1.466818438	-1.009960149	3.392228259	0.001281473	0.004381473	-1.190397884
BDNF	1.466702538	3.048253301	7.067581667	2.74004922639899e-09	5.39699859921047e-08	10.99589231
KRT15	1.463214766	-4.377919678	4.733305481	1.55551486538926e-05	9.24599155314466e-05	2.86948106
PALM3	1.462003485	4.11602903	5.227150745	2.66722050636367e-06	1.9765248008049e-05	4.145399523
SPTB	1.460543367	3.826096483	6.438814902	2.96648507103003e-08	4.10970125170528e-07	8.584154148
CRLF2	1.459124538	-2.590717406	4.164988529	0.000109186	0.000504475	1.0991097
LOC102724467	1.458012425	-0.265879504	4.017185298	0.000178129	0.000774803	0.578239271
TMEM229B	1.457525465	4.318656294	6.414994208	3.24552141231054e-08	4.44041757202922e-07	8.442849369
TRPM8	1.457017203	-0.255937401	5.738151882	4.09181609909685e-07	3.89481364592823e-06	6.315271372
FOXL2	1.451951792	-3.386324813	4.189631627	0.000100553	0.000469597	1.155221052
RTL1	1.451894444	2.119113899	3.837789766	0.000319127	0.001289904	-0.208985007
FOXE1	1.448712385	-3.01866862	3.174606178	0.002443906	0.007646796	-1.76398829
QPCT	1.447567894	0.794902894	6.081074789	1.13940797482283e-07	1.30268040650916e-06	7.507307235
ACOT4	1.447292277	-1.926825953	4.470419707	3.8783366250559e-05	0.00020446	2.056123833
LINC01166	1.445605774	-0.380110631	4.496750813	3.54236135026564e-05	0.000188781	2.099136798
OSGIN1	1.445318416	2.095863114	8.400203176	1.76754472986228e-11	7.83917950410229e-10	15.97607478
SLC24A3	1.444256312	4.175574835	7.666009335	2.82676463744797e-10	7.98289205786989e-09	13.13529974
LOC101926963	1.443959666	-3.681978262	3.793548231	0.00036776	0.001456331	-0.05958037
HAP1	1.443433173	4.118991723	7.410806505	7.4433520101789e-10	1.80174105938698e-08	12.18433219
MYHAS	1.442930558	-1.923023231	4.005348492	0.000185186	0.000800755	0.604133264
C1orf233	1.442620822	4.508482822	7.718315009	2.31843085287139e-10	6.7492381865583e-09	13.3011327
ASCL2	1.44202322	-2.174443323	3.830856892	0.000326317	0.00131607	0.084417047
CCKBR	1.438836144	1.664291634	5.608001649	6.62178634081625e-07	5.89140620487364e-06	5.758499727
SFRP5	1.437695196	0.065192228	4.756435566	1.43406791335198e-05	8.62104097194287e-05	2.92620787
KRT86	1.437183308	-2.682775774	4.592950041	2.53960849385815e-05	0.000141325	2.454147295
FCMR	1.435628548	-3.970095944	3.980464663	0.000200912	0.00086045	0.48830117
C6orf141	1.434846779	-2.512324355	3.739203039	0.000437279	0.0016963	-0.181276701
CCL2	1.434463397	0.543428562	4.928702259	7.7944771454596e-06	5.05474520083252e-05	3.474821869
EGFL6	1.434158408	2.745888484	4.713175086	1.6693614604005e-05	9.82329285012609e-05	2.534385429
ADRA1D	1.431714784	-0.31678573	6.356406498	4.0480178630266e-08	5.37694754640112e-07	8.51553549
IL1RL2	1.4210581	-4.337835513	4.86207128	9.87594365543731e-06	6.21697591436472e-05	3.299816277
ETS1	1.420528992	1.534271596	3.194797054	0.002304107	0.007271307	-1.985185647
SLC6A11	1.419892573	2.192832242	7.362447802	8.94335069282376e-10	2.09203175985319e-08	12.14530175
CCL18	1.41830383	-3.758833616	3.389659138	0.001291451	0.004409884	-1.219208752
PLCG2	1.41828626	0.313893795	5.073133186	4.64987214021402e-06	3.21501622501379e-05	3.980864622
SLC2A6	1.417993326	4.00348483	8.655987709	6.77734795139562e-12	3.49893824998517e-10	16.83542374
DRD2	1.417733357	3.124861923	7.13215963	2.14422453045824e-09	4.41076163529981e-08	11.23003694
SPATA8	1.417071847	-1.821751245	4.774517498	1.34563866947411e-05	8.15732710369957e-05	3.043751037
SLC1A4	1.416323138	6.944307441	9.517934721	2.78620983812277e-13	2.55721821705205e-11	19.82206544
VWA5B1	1.41594074	0.639171951	4.117914191	0.000127714	0.000579049	0.816268804
OPRL1	1.414976449	5.003486922	6.429215441	3.07593825483572e-08	4.24354258298917e-07	8.421733016
IL15RA	1.414775149	-4.035614189	4.072137876	0.000148629	0.000660287	0.766084411
FADS6	1.413804302	-0.418859469	4.733776252	1.55294549593211e-05	9.23902955074805e-05	2.877319113
ERICH5	1.412384919	-1.691675915	5.233684877	2.60478881771324e-06	1.93623123786879e-05	4.58172348
RAB3IL1	1.411569995	3.547697563	6.507103003	2.29210935393071e-08	3.29637250013333e-07	8.865841912
MIR1470	1.410369004	-3.601761728	4.162686663	0.000110028	0.000507745	1.063999707
PCYT2	1.40895046	5.306211315	10.37128089	1.27101096834159e-14	1.96471537579961e-12	22.97741665
IRX6	1.408889581	1.414173902	3.83395704	0.000323082	0.001303822	-0.137735784
LOC102724094	1.408529525	-2.441783211	3.820407026	0.000337449	0.001354972	0.057608845
GPR137	1.406996694	6.15075281	7.293721053	1.16099479789334e-09	2.61401835542715e-08	11.56680877
MTUS2-AS1	1.404979634	-2.184649721	4.352067258	5.81282267617672e-05	0.000291131	1.682063396
SLC4A3	1.40222222	6.112403343	7.982613835	8.52691588043858e-11	2.82800461063530e-09	14.16251591
TBX21	1.398304643	-2.800443309	3.558831858	0.000769868	0.002797623	-0.704096472
LOC101927157	1.398081375	-2.953699465	4.335702583	6.14520402602237e-05	0.000305676	1.626144932
GSG1L	1.397772933	3.086126881	6.450777126	2.83550385612662e-08	3.96775401953387e-07	8.702024889
GZMB	1.395819822	-3.680558269	3.762583887	0.00040595	0.001588525	-0.150787467
CHRDL2	1.394737333	-2.012280009	6.077630119	1.15420790192328e-07	1.31505075308354e-06	7.509644382

TACR1	1.394266084	3.550336	3.614378868	0.000647766	0.002400449	-1.068463285
KCNK4	1.393839328	0.829554384	4.176605807	0.00010503	0.00048732	0.982676653
SHROOM1	1.393643396	0.171352668	5.578140957	7.39249098256025e-07	6.5026859947426e-06	5.739969812
ACTG2	1.390302681	0.278040826	3.257993212	0.001913616	0.006185206	-1.670688184
LINC01586	1.385419764	-0.443313873	4.889477894	8.96095799026942e-06	5.70484110204219e-05	3.396924709
KLK13	1.384984324	-3.893984041	3.471961705	0.001005767	0.003544251	-0.997127528
S100B	1.384461207	2.252570813	4.191325257	9.99848138375563e-05	0.000467273	0.875967736
SCG2	1.383261251	8.152833749	6.177771835	7.92774098488812e-08	9.48145675813773e-07	7.286602761
GALR3	1.379916493	-3.451857736	4.387672588	5.14892274838267e-05	0.000262142	1.777264433
HS3ST2	1.379539554	2.477533537	5.402106697	1.4107040697006e-06	1.1389474854733e-05	4.95238047
PAK6	1.377510565	4.071808179	7.521054818	4.89835021773437e-10	1.26320089078412e-08	12.60075276
LOC400661	1.376042633	-4.500437899	4.199221617	9.73758057508107e-05	0.000457101	1.138692475
ASIC2	1.374070579	3.969954929	4.794492323	1.25415498325142e-05	7.68275287886091e-05	2.657005978
TRIB3	1.372933832	5.747460848	5.900409743	2.23834386410535e-07	2.31751442851143e-06	6.3932398
GJC2	1.369253444	1.747598468	6.790882013	7.82911413854987e-09	1.29503738438228e-07	10.05370784
HIST1H3D	1.366235419	-1.663129072	3.945110375	0.000225487	0.000954565	0.420685402
SPHK1	1.365496447	2.033029132	4.487219285	3.66056767136402e-05	0.00019418	1.860801095
RTP1	1.365396132	0.357861523	8.142780122	4.65779921935921e-11	1.70762284001833e-09	14.99706124
MYH7	1.362249147	2.685043642	6.568298084	1.8187248370994e-08	2.69173312536665e-07	9.170912812
STK32B	1.361926447	3.810136771	6.813263871	7.19217554321077e-09	1.20399478235396e-07	9.97790572
HIF1A-AS1	1.360009119	-3.152248228	4.305217953	6.8143118189536e-05	0.000334303	1.524657099
NRGN	1.359089372	3.995841034	5.747214346	3.95659430101011e-07	3.78929917241305e-06	6.022868052
EPHX1	1.358457245	5.056233788	7.95222147	9.56495928518279e-11	3.11751626122363e-09	14.12726456
SPR	1.358356785	-0.941213562	4.052063029	0.000158812	0.000700815	0.729468906
HSPB7	1.358191017	-1.702196328	3.678132187	0.000530416	0.002009668	-0.367239275
NXPH3	1.357666586	3.274808937	4.943885818	7.38412866300224e-06	4.82576171288171e-05	3.258742146
OOSP2	1.355970894	-4.409296203	4.019078172	0.000177025	0.000770761	0.584469894
CRP	1.355426919	-4.608736049	4.83093158	1.10269991186129e-05	6.85827453417166e-05	3.185435028
DCLK3	1.353569008	1.258852114	7.493323345	5.44189711008772e-10	1.38244495508712e-08	12.65358141
P2RY2	1.35308429	-2.366556648	3.212906943	0.002185149	0.006944902	-1.647817706
TLL2	1.352515412	1.488163188	5.723344755	4.32266859512893e-07	4.07784079140053e-06	6.183100225
TRIM29	1.352358381	-3.05415501	3.525419233	0.000853572	0.003063889	-0.807977095
LOC100132111	1.348624482	-2.559464003	4.743730578	1.4995791834293e-05	8.96795849673909e-05	2.946920921
LINC00900	1.347617413	-1.061038675	4.505518033	3.43692473831809e-05	0.000183866	2.160879883
LINC01574	1.347469445	-2.866341435	4.033546675	0.0001688	0.000739493	0.690871593
MIR4651	1.346779397	-3.394370926	3.912035079	0.000251084	0.001047987	0.305531539
SHISA4	1.340380913	3.684767823	10.64827893	4.7481138442941e-15	9.02927289541194e-13	24.01990108
NTSR2	1.340044483	-3.53056447	3.888184048	0.000271255	0.001120501	0.22869552
MAOA	1.336862414	4.076586385	7.139340049	2.08655128833092e-09	4.30889142222275e-08	11.16995102
PLD5	1.336412719	1.172543018	5.769691289	3.64001749590668e-07	3.52183684001835e-06	6.369695362
ANKRD34C-AS	1.336275083	-4.092336654	3.977133256	0.000203112	0.00086889	0.472550138
HTR5A	1.335699247	1.77686651	5.003762135	5.96279835927418e-06	4.00342009221982e-05	3.628578743
NQO1	1.33378328	3.704179814	4.940466933	7.47463576990471e-06	4.8700282796621e-05	3.19271158
TMEM217	1.329800872	-0.463485485	5.448829789	1.1889444163088e-06	9.82105242384078e-06	5.307512187
HS6ST3	1.329710602	6.043627285	5.326566023	1.85854063318133e-06	1.45216685063204e-05	4.289966534
NPW	1.327292524	-1.603911628	4.597336987	2.5011998391834e-05	0.000139511	2.464383728
ZNF771	1.324909875	3.106268627	5.383213368	1.5115549229848e-06	1.20855811492006e-05	4.817808829
BEGAIN	1.323475625	5.061231961	6.360426724	3.98713631476305e-08	5.30941935557339e-07	8.157540435
BFSP1	1.322948267	0.139860629	5.117683452	3.96130963831784e-06	2.80046262823363e-05	4.142440995
MYRIP	1.318780023	2.64030544	5.672938611	5.20950548217402e-07	4.78467193920452e-06	5.902975644
SUPT20HL1	1.317010383	-3.781912656	4.198506286	9.76094108439061e-05	0.000457782	1.169037591
PDE2A	1.310799309	4.493086726	6.659241084	1.28917494149888e-08	2.01042839107021e-07	9.331059662
HOXC13	1.308370952	-2.041285379	4.020218022	0.000176363	0.00076826	0.653281204
RAET1G	1.308105094	-1.848414865	3.881339057	0.000277326	0.001143258	0.232837279
SRXN1	1.305797064	4.208800018	5.705933099	4.61066995265501e-07	4.31259160858209e-06	5.846677596
SCARNA20	1.30562464	-3.789190279	3.584488976	0.000710965	0.002607597	-0.672673563
NANS	1.305018372	4.878209101	8.5553155	9.87817361010020e-12	4.7560976873548e-10	16.39346424
NGEF	1.304656809	4.140722351	4.810083028	1.18701036518835e-05	7.3291859338995e-05	2.687237233
CCL20	1.303005349	-4.527729985	4.10809109	0.000131947	0.000595688	0.852781745
SPRED3	1.301444318	5.763263936	10.1669201	2.64306470603701e-14	3.56449639666716e-12	22.22251736
SKAP1	1.299407412	-2.477770216	4.225386615	8.91939215421934e-05	0.000422748	1.288802137
LINC00494	1.296344181	-2.937253936	3.307517016	0.001652155	0.005450751	-1.405360571
CACNA1S	1.29435082	-2.339211017	4.228664067	8.82172861749599e-05	0.000419171	1.298471495

PRR29-AS1	1.290324414	-1.779289946	3.825722732	0.000331741	0.00133591	0.067707722
LINC00856	1.288835085	-2.593059694	3.310926104	0.001635458	0.00540511	-1.384336254
HCRTR1	1.28747499	0.688198101	5.213501333	2.80243099855484e-06	2.06456685018952e-05	4.440519289
CABP1	1.281898255	1.709267616	6.124562343	9.68008548756246e-08	1.13168376688518e-06	7.614870762
ALDH1A2	1.279904136	0.108417341	3.494524851	0.000938622	0.003336103	-0.998321749
RSPO4	1.276314346	1.255344565	5.784559855	3.44452631018775e-07	3.36844853707705e-06	6.417259158
RCAN1	1.274598961	5.692766268	8.911198904	2.6168185629557e-12	1.60488086019972e-10	17.65198888
OR2L13	1.274467189	0.054048661	3.490075105	0.000951517	0.003374221	-1.005899929
LY6H	1.270600659	6.267819688	5.594386804	6.96279588635134e-07	6.17402159221486e-06	5.236594325
VEGFB	1.268925893	5.258342277	6.873733071	5.71827397680081e-09	9.99016100652848e-08	10.05465601
COLEC10	1.266375968	-2.329082646	3.561822322	0.00076277	0.002776029	-0.689004123
TAL1	1.264448477	-1.896458774	3.29892897	0.001694934	0.005576574	-1.423097803
PLEKHF1	1.263107092	0.85568576	4.774094638	1.34764433305867e-05	8.16650221360379e-05	2.928754383
BATF3	1.260892216	0.079086216	4.701574847	1.73862730757515e-05	0.000101765	2.742599096
PHLDA2	1.259816111	0.862828151	3.878553656	0.000279834	0.001151441	0.055125839
PNMA5	1.259029151	1.567254062	4.518840871	3.28253499441634e-05	0.000176898	2.013441244
LOC100133669	1.2534352	-3.090496197	3.350770174	0.001451773	0.004872362	-1.294255215
S100A2	1.252306282	-2.423151951	5.075085975	4.61736238885191e-06	3.19420936991684e-05	4.048190188
GABRE	1.252043999	3.100462783	5.762943476	3.73232549024476e-07	3.59797810662435e-06	6.180315666
NUPR1	1.251376372	1.643015847	3.291946004	0.001730487	0.005677294	-1.736316268
AK5	1.25076077	3.557064422	5.275540482	2.23767164594687e-06	1.70211149977881e-05	4.382326413
LINC01605	1.249329326	-2.575655619	3.582090607	0.000716284	0.002624918	-0.631427578
ARHGAP22	1.248815202	1.605714276	4.33239118	6.21467419579106e-05	0.000308609	1.400898549
KIAA0319	1.248515765	3.006136275	8.464429722	1.38884552972099e-11	6.35144530918945e-10	16.18600647
KCNA5	1.247981818	1.866697298	6.152888548	8.70406216264683e-08	1.03079961982636e-06	7.706927238
MTNR1B	1.244100242	-3.419648144	3.465110017	0.001027044	0.003608167	-0.992749788
CSRNP1	1.24360813	3.723175773	5.418139893	1.33035203142099e-06	1.08367319712022e-05	4.868841491
SSTR4	1.242166047	-4.424545094	3.909808806	0.000252904	0.001054251	0.251102151
LOC285484	1.242010177	-0.461075346	4.582029602	2.63773778660686e-05	0.000145987	2.380469288
CYBB	1.241798346	-1.763432006	4.921318976	8.00202371688237e-06	5.17663957191267e-05	3.531306007
FBXO41	1.241064277	6.226997085	7.21019114	1.5943526535401e-09	3.43468000741855e-08	11.24530434
RIMBP3	1.234834373	0.387284514	6.43220853	3.04138643795065e-08	4.20465312313543e-07	8.786124832
DHRS13	1.233205165	5.47404177	6.639726422	1.38802035965293e-08	2.13482948032159e-07	9.157471581
LOC101927657	1.232812998	-4.025066945	3.720792812	0.000463564	0.001785689	-0.289828783
LPAL2	1.231410415	-1.37614561	3.834332028	0.000322693	0.001302451	0.08930901
SLC7A5	1.230854466	7.811575649	6.468398545	2.65297598821255e-08	3.7480552804074e-07	8.382004407
VAX2	1.229001611	0.786413093	4.236754849	8.58505450081293e-05	0.000409471	1.176136598
KIRREL3	1.228406088	5.183266179	7.276236982	1.24069066756674e-09	2.77689893444469e-08	11.57321603
HEPACAM	1.223688681	-2.670233437	3.248805352	0.001966228	0.006336663	-1.5527281
TRPV2	1.22361253	0.501151651	3.32346172	0.001575423	0.005228267	-1.516021423
TNFAIP3	1.223066745	2.985663817	5.612805728	6.50544572321787e-07	5.80352503549841e-06	5.650849461
ISLR2	1.221420226	7.453525514	4.891182796	8.90687113570963e-06	5.67587571673608e-05	2.674049159
C17orf102	1.220846891	1.37642968	3.780386657	0.000383552	0.001511168	-0.297307941
SPOCD1	1.22012276	-1.822894296	3.284313669	0.001770151	0.005784448	-1.462231979
CHRNA6	1.219904697	-0.673275551	3.373772675	0.001354804	0.00459299	-1.267697771
SLC5A1	1.21821226	-3.508080722	4.123246546	0.000125471	0.000570251	0.945389928
TMPRSS3	1.21700333	-3.586189968	3.224919422	0.002109457	0.0067334	-1.657323935
KBTBD12	1.215369727	-3.382573037	3.319122688	0.001595963	0.005286479	-1.393515976
THEM5	1.212791457	-1.635496186	5.050475513	5.04392839875581e-06	3.45583616807445e-05	3.963017377
DUSP26	1.2095809	5.060596277	5.941721824	1.91868897045486e-07	2.02381107565975e-06	6.607852426
MSC-AS1	1.209464619	-0.968616995	3.103180969	0.003005036	0.009165949	-1.96796077
KLF2	1.208308986	-0.022537025	5.158025657	3.42495719312421e-06	2.45876951346693e-05	4.288377803
KCNC2	1.207325605	3.074239947	6.632724559	1.42529847534646e-08	2.18658819494097e-07	9.375399197
AANAT	1.204963819	-1.321855246	5.172551167	3.24989938096319e-06	2.34904540161335e-05	4.374092422
FIBCD1	1.204316737	3.256971594	4.989986677	6.26387281968404e-06	4.16745407104727e-05	3.418625519
NPY	1.201199181	-0.275509321	3.150668094	0.002619972	0.008124557	-1.904493844
ETNK2	1.199588524	5.785462003	7.589867738	3.77292503716834e-10	1.01351349092958e-08	12.70372312
SYNPO	1.198992714	2.243477441	5.306883905	1.99662328075355e-06	1.54197420273957e-05	4.637734746
GBA	1.198326547	3.895433804	7.219617912	1.53828876258064e-09	3.33018729412728e-08	11.48631308
PEMT	1.198153954	5.209734092	7.652381808	2.97663989722485e-10	8.36147953276773e-09	12.98479122
MGLL	1.196374295	2.746099216	4.797483166	1.24099221324758e-05	7.61624034659236e-05	2.817835068
HTR1E	1.195417123	-0.923892053	5.459808145	1.14205340303713e-06	9.47517187774024e-06	5.353649538
LINC00944	1.193899968	-5.004251413	3.977828471	0.000202651	0.000867058	0.421911646

DGAT2	1.192199048	1.905439415	7.075749266	2.65638471329615e-09	5.26753316778373e-08	11.09647203
MMP7	1.191753263	-4.55826094	3.530801043	0.000839528	0.003020861	-0.870138581
CTSD	1.191577102	7.385586064	11.28731359	5.07252502927751e-16	1.3681842254989e-13	26.10375078
MIP	1.190871998	-2.063259828	5.310445619	1.97090707374485e-06	1.52708794624521e-05	4.844747727
ADAMTSL2	1.189306949	1.726303347	4.590014782	2.56562828653642e-05	0.000142623	2.231904301
CCL5	1.189267701	-4.407113632	3.763542184	0.000404713	0.001584154	-0.185068137
SCN2B	1.188927495	2.434737692	6.623351066	1.47677018478749e-08	2.25508181943893e-07	9.392417341
COL5A3	1.18869415	2.848292065	3.462693687	0.001034649	0.003631507	-1.414387535
GPR1	1.188379926	0.597190426	4.509192699	3.39364811285878e-05	0.000182067	2.071301055
ADM2	1.18655573	2.929443888	5.354212293	1.68035994616305e-06	1.32627514054726e-05	4.733445746
SNORD116-1	1.183556583	-1.767834106	3.637416688	0.000602754	0.002253232	-0.478881467
TSSK1B	1.183260868	-2.871503811	3.563686202	0.000758378	0.002763088	-0.69246203
SLC6A9	1.182078251	5.116578019	6.044056992	1.30883318555756e-07	1.45976318961362e-06	6.97749708
CCDC149	1.181007068	4.206439894	13.23738252	7.57554396443077e-19	5.26958825294206e-16	32.63708555
TXNRD2	1.180091581	2.554386541	6.072801982	1.17527371204666e-07	1.33445060268597e-06	7.359666539
RFX8	1.177166297	-3.051909562	3.435683245	0.001123386	0.003901518	-1.058722409
TDRD10	1.177123344	-2.641233919	3.821137005	0.000336659	0.001352625	0.060369948
LYPD6B	1.175257672	-0.572471153	4.412446161	4.73114962775887e-05	0.000243731	1.837883277
PSEN2	1.172584262	4.474193129	9.423406055	3.9408066620154e-13	3.47205418665507e-11	19.61648493
UAP1L1	1.17221201	3.446304084	5.951553236	1.84955099532701e-07	1.95842434847483e-06	6.827974817
PRKAR1B	1.170887332	6.454534397	6.943592382	4.38687302447622e-09	7.92605705098974e-08	10.22729926
RHBDD2	1.169237183	7.454239324	9.954223729	5.69107713157608e-14	6.86905213785162e-12	21.38820421
KCNK15	1.168930314	-2.037778932	4.357328858	5.70971789338566e-05	0.000286875	1.701125856
SYN1	1.167449257	6.411971889	8.12753857	4.93340844591021e-11	1.78392319358064e-09	14.68706166
SQSTM1	1.16338114	7.628186522	5.823251524	2.98322730225965e-07	2.97008087685986e-06	5.998725284
GPR45	1.161473139	0.724072104	5.996346517	1.56455878369573e-07	1.6977004240324e-06	7.204473672
ABLIM2	1.15982435	3.788551479	6.774491885	8.33096124172728e-09	1.37118492218292e-07	9.832986354
UBASH3A	1.157991794	-3.944101487	3.258543369	0.001910508	0.006177427	-1.586591726
TMEM54	1.157955759	1.156874753	5.069594578	4.70935682500458e-06	3.24680305047851e-05	3.908571725
RIN3	1.157323244	0.647297687	4.015210838	0.000179288	0.000779203	0.494414758
AKR1B10	1.156057911	-4.589360141	3.560587368	0.000765693	0.002785902	-0.787121672
SPRR2G	1.155558004	-4.427416973	3.62206833	0.000632397	0.002350414	-0.599855597
SMKR1	1.155098348	1.395647036	6.231131708	6.48736286753149e-08	8.01310573259158e-07	8.02071509
MPPED1	1.154561714	1.249482827	4.083734964	0.000143037	0.000638061	0.64644545
MANEAL	1.15414907	6.012825942	10.98627931	1.4457211613773e-15	3.32302151814663e-13	25.10447426
OTOP3	1.153946412	-4.361923271	3.57965566	0.000721722	0.002641552	-0.717943074
RASL10B	1.153852685	5.351229988	7.432629394	6.85160061871657e-10	1.68629757127128e-08	12.14513474
NUDT14	1.153014554	3.981939196	4.849903412	1.031097229712e-05	6.45699906964637e-05	2.841747291
IL7R	1.151671643	-4.753885053	4.249658403	8.22028812848591e-05	0.000394311	1.283968916
GJA10	1.151533999	-4.77546453	3.8317714	0.000325359	0.00131241	-0.005082023
MCHR1	1.149463841	3.088209984	4.941218797	7.45463897445535e-06	4.8593951174298e-05	3.269815839
HM13	1.148529666	6.796939065	9.798974775	9.99235130416234e-14	1.08694576964166e-11	20.84918562
DUSP5	1.148436581	2.842680425	4.49907941	3.51405348626489e-05	0.000187461	1.803584893
NPPB	1.14812062	-2.189808287	3.076679296	0.003242373	0.009792693	-2.000723796
OASL	1.146583688	-4.054138416	3.109258805	0.002952949	0.009024778	-1.989153356
CALY	1.146519898	5.127833598	4.013289785	0.000180422	0.000783103	-0.068599723
MAPK8IP1	1.14453969	7.975143829	8.951197189	2.25531286542196e-12	1.46231899338358e-10	17.69870234
INHBA	1.14425783	-1.659944776	3.089973625	0.003121204	0.009475455	-1.975691756
ZNF358	1.143173632	6.510677636	7.427958359	6.97416664178822e-10	1.71168195768234e-08	12.04857353
ABCG5	1.142164068	-2.740199311	3.810977105	0.000347807	0.001389178	0.027666323
ZNF860	1.140613239	-1.085274654	4.191089247	0.000100064	0.000467477	1.166016871
SLC9A3R2	1.140295834	3.676830979	6.40508635	3.36913920700092e-08	4.59053900302347e-07	8.471309354
NFAM1	1.139691781	-0.33303756	6.385201104	3.63156228233587e-08	4.88265950198292e-07	8.618744949
CHAC1	1.139184232	5.157810614	5.161075943	3.38744228894755e-06	2.43514446624288e-05	3.780277812
C7orf76	1.13871487	-3.043965535	3.520022649	0.00086788	0.003109333	-0.822506504
RPEL1	1.13738587	-3.25987906	3.350315894	0.001453753	0.00487653	-1.302724603
PRR19	1.136815306	1.574968627	8.270212934	2.88174435162149e-11	1.16294883124291e-09	15.50146817
BTBD6	1.135594078	5.172541179	9.568101608	2.31878818042173e-13	2.16581370929638e-11	20.09497107
ATP1A3	1.134260601	9.453685781	8.309936025	2.48162894306705e-11	1.03465138567967e-09	15.26142585
LINC01314	1.131533861	1.393920513	3.355499623	0.001431315	0.004811642	-1.5307632
PRR18	1.130072417	-0.221571632	7.679539355	2.68544206375123e-10	7.6739773049877e-09	13.2911157
FOXN3-AS1	1.129452802	-0.65308926	3.87065706	0.000287063	0.001177336	0.160892191
FOXD4L1	1.128981235	-1.067309105	5.008759619	5.85712667338512e-06	3.93747277104753e-05	3.818249739

ATP6V0B	1.128956488	7.484911148	6.011267349	1.47966458258545e-07	1.62088578166105e-06	6.696124382
MAFG-AS1	1.12846935	2.936034676	6.67587283	1.21049261817051e-08	1.90571479309714e-07	9.546795203
MLC1	1.127974818	4.536071607	5.774660611	3.57348814655872e-07	3.47339296203242e-06	6.054623313
GAMT	1.126273569	4.773111866	7.616594096	3.40924584749093e-10	9.33850730432412e-09	12.88920429
PDZRN3-AS1	1.125492072	-0.059025341	4.18313299	0.000102763	0.000478313	1.077022431
KRT18	1.122091442	2.77355461	3.233661448	0.002055933	0.00658721	-2.046726937
ISM2	1.120702891	0.44410503	3.828472313	0.000328825	0.001324774	-0.055361532
LOC101926941	1.119728328	-0.693327268	5.434107437	1.25482334053304e-06	1.02944585227529e-05	5.261249197
ELOVL7	1.119120328	0.448086085	3.178730195	0.00241472	0.007567973	-1.903574534
SLC6A12	1.118632649	0.072097566	4.300492599	6.92417217298563e-05	0.000338813	1.438315183
BEND6	1.117112469	2.26375003	4.447451406	4.19660556757951e-05	0.0002194	1.701279004
SDSL	1.115844615	3.242858077	3.284643003	0.001768422	0.005782372	-1.971958402
RPS6KA2-IT1	1.114829872	-0.397897131	5.351407416	1.69764055707335e-06	1.33938726451037e-05	4.968192
BAALC	1.114111287	5.738936007	6.085243348	1.12174972669551e-07	1.28415809985891e-06	7.070125952
LY6E	1.113734403	4.389526953	5.451081403	1.179174787057e-06	9.74644376056209e-06	4.902145549
DBNDD1	1.113572211	5.793660879	6.819763865	7.0170833567268e-09	1.17991453160534e-07	9.804228085
PNPLA1	1.112351789	-2.519184397	4.652510702	2.0640034611954e-05	0.000118167	2.650299377
CHRNB2	1.111304023	5.79778398	10.01116247	4.63249763669258e-14	5.77598160522146e-12	21.65997592
ATP2B3	1.110013997	4.567018034	7.012993839	3.37099908337414e-09	6.36041554764585e-08	10.64321503
PALM2	1.10998086	4.107572049	5.377367052	1.54417986777716e-06	1.2305488828747e-05	4.673565501
SLC35F4	1.108118201	1.637627877	3.841718678	0.00031512	0.001275584	-0.143531973
ATP6V0C	1.108045561	8.172878546	7.421215031	7.15499020883147e-10	1.74633292880926e-08	11.95348982
CYP4A11	1.107980907	-0.727485967	4.187455823	0.000101288	0.000472611	1.134825771
HRH2	1.107979871	-0.452441916	4.538064296	3.0716257946945e-05	0.000166617	2.236665703
ASTN2	1.107453802	4.329411456	7.995938265	8.10820422530673e-11	2.7198497752225e-09	14.35145689
SNORD116-2	1.106860037	-1.997567455	3.161786707	0.002536762	0.007898956	-1.781411973
CABLES1	1.106562743	3.071133089	6.282105332	5.35534717732942e-08	6.83194459161913e-07	8.078975499
TEX261	1.104700351	5.929987612	10.64574	4.79096406682924e-15	9.04568236989267e-13	23.91381814
PTDSS1	1.104019344	6.350898651	7.608800684	3.51150853027001e-10	9.55918691870518e-09	12.73856533
NENF	1.103723751	3.133707198	5.324191622	1.87468551588617e-06	1.46123388419546e-05	4.603450157
GPX3	1.103183078	3.392332422	5.523673187	9.0335329845551e-07	7.74265166604232e-06	5.284285856
STK32C	1.102988076	5.078861136	7.653413062	2.96502488381146e-10	8.33771305891365e-09	12.99868515
LAMP3	1.102957545	0.37833297	3.366176869	0.001386126	0.004685953	-1.385734845
DACT2	1.102715832	0.782573251	5.252615977	2.43192311816206e-06	1.82986119505772e-05	4.56911649
ZNF469	1.100268809	1.422620673	4.336202549	6.13478100278443e-05	0.000305387	1.430820278
PITPNM1	1.099446234	7.150195987	7.353751571	9.24356232480761e-10	2.14841338696009e-08	11.73788985
DRD1	1.099178709	-0.265417608	5.775652551	3.56035299250442e-07	3.46250223145215e-06	6.447471498
DOC2B	1.098952623	4.900664641	5.541171083	8.47049870438066e-07	7.32180157792328e-06	5.165118258
C9orf50	1.098931216	-2.117750004	5.12917196	3.80067278336215e-06	2.69989743839322e-05	4.230261076
RASGEF1C	1.097587692	3.225882102	6.265616973	5.6981767654281e-08	7.18949434083824e-07	8.002887695
TCTA	1.096844377	4.283070995	6.493640067	2.41171858500437e-08	3.4458895868876e-07	8.732636797
MS4A2	1.094553462	-4.507024841	3.14746515	0.002644414	0.008185945	-1.919051985
HRAS	1.092002795	5.728759546	7.363886997	8.89461637153193e-10	2.08432087365872e-08	11.85537379
LRFN2	1.091743212	2.549838949	5.670364623	5.2593362559049e-07	4.81931966590415e-06	5.900798964
REEP2	1.091494549	7.482181432	7.385363812	8.19815105232314e-10	1.95051059195371e-08	11.8435771
SLC37A1	1.090478675	4.879660474	8.569846401	9.3549383137963e-12	4.54557140530474e-10	16.44388343
ADGRA1	1.089990599	4.807649655	5.820676622	3.01192678392088e-07	2.99639671356344e-06	6.190039598
PEBP4	1.089262787	-1.877196132	4.600166564	2.47672785828097e-05	0.00013838	2.478345305
SPRN	1.088384992	4.420572709	5.846448627	2.7365962559582e-07	2.75252849443467e-06	6.328964408
SYS1-DBNDD2	1.086595064	0.12101109	5.25898091	2.37638209931251e-06	1.79459127380765e-05	4.628948585
TNFRSF11A	1.084288646	-1.913749893	3.894524033	0.000265746	0.001100669	0.277710006
PNMA6A	1.083612563	3.7114628	6.144881343	8.9695943325578e-08	1.05750797052855e-06	7.506214623
TMEM255B	1.081673693	0.804059137	4.314380577	6.60609343410977e-05	0.000325357	1.421302943
TEX38	1.081588844	-2.690406825	3.558563484	0.000770508	0.002799179	-0.70047063
CDK5R2	1.081474829	5.462665529	5.145967618	3.57730220252393e-06	2.55910227657145e-05	3.694293634
LRRTM1	1.080465559	2.899604674	5.569550691	7.6301930033938e-07	6.6828658601295e-06	5.503435066
SYT5	1.079881023	6.265347464	5.789202489	3.38564361819591e-07	3.31454510221379e-06	5.943552448
PCSK1	1.07835148	5.613722884	5.847623986	2.72465265719117e-07	2.74228340144825e-06	6.205944196
SYT9	1.074626615	4.008848653	8.577070843	9.10525638815116e-12	4.44057642265682e-10	16.54008059
NPB	1.074358288	1.373715143	4.444320596	4.24191275266937e-05	0.000221442	1.786778616
CYGB	1.073926609	3.877475848	5.482129032	1.05227632523568e-06	8.80773277547647e-06	5.076359417
PLA2G4C	1.072184366	3.135058022	6.088581652	1.10780469767888e-07	1.27039486220155e-06	7.36152334
C1orf216	1.071931417	5.253239609	5.886450512	2.35787378951886e-07	2.42323786463266e-06	6.381899139

BAD	1.069672571	5.873397782	5.739200125	4.07594402988248e-07	3.88110333364134e-06	5.788519338
NCS1	1.069272345	7.803600681	7.466408776	6.02712711959117e-10	1.50297218068069e-08	12.13717492
HIST1H4H	1.068819952	-1.375265411	3.571724173	0.000739711	0.002701781	-0.669657332
COL8A2	1.067343004	0.315691379	4.618579831	2.32309507861015e-05	0.000130986	2.451718633
MAL2	1.067181983	3.757190524	4.700281271	1.74652342038751e-05	0.000102114	2.359793214
KCNIP1	1.065913057	4.177558549	6.515088201	2.2239774480736e-08	3.21061692435443e-07	8.82388694
TMEM198	1.064900993	5.475113272	9.923919959	6.35076506245766e-14	7.56170148179924e-12	21.36314905
IRAK1	1.063473142	4.480243358	7.022675943	3.24935168655677e-09	6.19697785936185e-08	10.68772045
CDHR1	1.063127363	3.19848699	3.589322727	0.000700361	0.002572629	-1.09651882
KIF17	1.062969674	3.676689574	8.119926183	5.07713202845583e-11	1.82590246133569e-09	14.8681597
ABCA3	1.062457651	7.463583641	9.840218217	8.60224527107054e-14	9.71722860043622e-12	20.97518461
ATP6V0D1	1.059888127	6.865291438	10.41721874	1.07885187749228e-14	1.75053087296277e-12	23.06722018
NXPH4	1.058789897	6.232514711	4.265731154	7.78692344719362e-05	0.000375948	0.636063374
FIBP	1.058551332	6.093761814	10.02514219	4.40447507045145e-14	5.51770092593569e-12	21.69535764
CYP46A1	1.057547259	3.779529626	6.085758944	1.11958460699425e-07	1.28223483174519e-06	7.280745107
PPARG	1.056895397	-1.384791399	3.216400658	0.002162875	0.006884961	-1.646289723
THEM6	1.055750048	3.61194434	6.640838118	1.38219174390535e-08	2.13034835957143e-07	9.35251529
HPCAL4	1.054735329	6.808264505	6.028761198	1.38593830261475e-07	1.53410833974103e-06	6.791826781
C19orf24	1.054539386	3.700774771	6.321180187	4.62262714083008e-08	6.00441784833226e-07	8.157410252
ACOT7	1.054035527	7.181415491	7.367443055	8.77533679366867e-10	2.06552517780093e-08	11.78802077
CUEDC1	1.052731413	6.419073411	6.846496009	6.34060766483131e-09	1.08129859615798e-07	9.863173722
ALK	1.052572106	3.708796098	5.702948289	4.66191379994231e-07	4.35505356020748e-06	5.89235707
CAMK2N2	1.05124418	5.612032589	4.448692035	4.1787826372595e-05	0.000218642	1.287779309
SPATA2L	1.051232559	4.809984096	7.121671653	2.23133974653483e-09	4.55803736631801e-08	11.0252131
DGCR5	1.050167	4.654042939	6.080888233	1.14020465757902e-07	1.3030276573189e-06	7.161375149
ATP8A2	1.050063192	5.149704126	7.060641989	2.81320141177976e-09	5.52051617799364e-08	10.76263177
DMRT2	1.047715942	-2.514257598	3.355601066	0.001430879	0.004810789	-1.259270777
GALNT9	1.047107764	4.21044992	4.712937705	1.67075143949932e-05	9.82709675128738e-05	2.341784266
DYNC1I1	1.046213125	4.472172345	6.828515967	6.7880271152068e-09	1.14870627872126e-07	9.960941665
VSIG2	1.045076882	-0.934450996	3.810925053	0.000347865	0.0013892	0.001808249
LOC648987	1.043942111	2.885168888	3.602826844	0.000671528	0.002479814	-1.014491536
USH1G	1.043332509	-0.380606662	5.535614327	8.64543419386263e-07	7.44622880568169e-06	5.607374214
SLC36A1	1.04319228	4.572000696	8.578606569	9.05304985652727e-12	4.42843348429313e-10	16.50092782
ATP6V0A1	1.042707176	7.965041688	9.141239413	1.11492357530062e-12	8.11867076196178e-11	18.4018666
GABRQ	1.041287207	1.484426904	6.142408111	9.05322640956773e-08	1.06499302930175e-06	7.692587888
ZDHHC14	1.039818347	3.599143004	5.75817968	3.79888538861196e-07	3.65813979880437e-06	6.105312694
HOXD11	1.03915192	-4.886404042	3.26637836	0.001866761	0.006050037	-1.629367215
TPGS1	1.038994798	4.336476219	3.84527802	0.000311532	0.0012628	-0.490598739
HTR2A	1.037833348	0.483339155	4.361423953	5.63070439789184e-05	0.000283552	1.600659117
SURF2	1.036831402	4.747068729	6.078489396	1.15049826323897e-07	1.31252138939127e-06	7.141703274
RASGEF1A	1.035771545	2.166314317	5.514153595	9.35522168811358e-07	7.96670666500987e-06	5.377701805
CDH22	1.035601757	3.82312112	3.910442727	0.000252384	0.001052577	-0.217108176
LIMS2	1.035449983	1.008121717	4.756947131	1.43148964500779e-05	8.61141688358917e-05	2.856822624
ADORA1	1.0339098	3.984465597	5.832861171	2.8784926991383e-07	2.87881942929711e-06	6.330874368
CPA4	1.033354014	0.006617052	3.725445997	0.000456781	0.001763157	-0.322167035
EMP1	1.033226599	1.860480681	3.565160277	0.000754921	0.002752012	-0.992287348
UBTD1	1.031442203	3.531670703	7.165610058	1.88845397925659e-09	3.95293365567151e-08	11.31735523
KRT37	1.030490035	-4.574450741	3.572439413	0.000738072	0.002696911	-0.75202638
SNORA2B	1.029965971	-2.929322433	3.433301878	0.001131546	0.00392728	-1.060201126
ABHD14A	1.029009521	4.06055582	5.234864547	2.59367186486298e-06	1.93013875011045e-05	4.172225935
SLC45A1	1.028639739	3.91744964	7.593750826	3.71776585723952e-10	1.00399878724531e-08	12.88321072
RGS6	1.028547746	2.709497703	4.019139936	0.000176989	0.000770732	0.272025128
IRAK2	1.027927534	1.38025733	6.05280318	1.26667421945705e-07	1.42174096148231e-06	7.373930886
TMEFF2	1.026498734	5.319020422	4.272277214	7.61685266295398e-05	0.000368545	0.736702037
CPNE7	1.024089276	3.552914609	3.555135242	0.000778728	0.002825161	-1.247511674
TEC	1.022748947	-0.390620999	3.387834925	0.00129858	0.004430795	-1.252979776
C15orf27	1.020309331	3.178223811	4.32678104	6.33411554878722e-05	0.00031348	1.194209243
RET	1.019053284	4.458363333	5.313133554	1.95171622443845e-06	1.51600690451312e-05	4.399371665
STS	1.018822156	4.062061301	8.30646585	2.51423837995432e-11	1.04630164182066e-09	15.53136152
SNCB	1.017673943	5.205859429	4.287155078	7.24362003498097e-05	0.000352746	0.797453559
SEC11C	1.01598845	6.781429632	5.072575086	4.6592046005999e-06	3.21906925451875e-05	3.339156437
AMER3	1.015904264	5.013606737	6.386107463	3.61917023995923e-08	4.87095351083718e-07	8.251613343
TICAM1	1.015515021	-0.65561534	4.454721815	4.09320327525055e-05	0.000214716	1.977885506

DHCR7	1.015405126	6.440366717	5.808264489	3.15415527865721e-07	3.11794265073844e-06	6.001652002
IRF4	1.015400918	-0.633616387	3.253983112	0.001936414	0.006247433	-1.597661084
PARD6A	1.012575687	3.964429596	7.678306174	2.69802408808196e-10	7.69157007610948e-09	13.19513817
FBLL1	1.01123943	5.711712295	4.999868276	6.04643007126169e-06	4.0492851804829e-05	3.157729991
ACHE	1.011038001	5.400047163	4.777537393	1.33139977689568e-05	8.08012898074921e-05	2.417693147
ABLIM3	1.010243372	5.854431467	5.439004963	1.23251757070043e-06	1.01303286524641e-05	4.702368446
HCN2	1.009067549	4.479796927	6.073124659	1.17385399998633e-07	1.33398464237484e-06	7.151956412
QRFP	1.008612754	-1.679975864	4.81309955	1.17443193554503e-05	7.2566057391916e-05	3.174141382
CCDC86	1.008334538	4.300695188	6.613165344	1.53480464510251e-08	2.32890305304217e-07	9.174551221
LOC151484	1.006972773	-1.614144667	4.121360543	0.00012626	0.000573047	0.963901625
TMEM120A	1.005777452	5.054791093	7.410374816	7.45555974575723e-10	1.80304492918208e-08	12.08549615
SLC22A3	1.005381575	-1.452564273	3.112921309	0.002921971	0.008950802	-1.915597709
NOXO1	1.00514887	1.901073265	6.8216706	6.96653274160308e-09	1.17365430184063e-07	10.15757676
SYP	1.003858087	8.273424619	5.553368119	8.09869238987734e-07	7.04418347948759e-06	4.989488112
FOXD4	1.001834333	0.995958494	4.647434134	2.10088814496261e-05	0.000119941	2.491742471
PQLC2	1.001795241	4.656896008	9.303871051	6.11684054148174e-13	5.03696093560706e-11	19.1660068
RPS10	1.000795484	2.001851598	5.093909111	4.31523980362104e-06	3.01359930592114e-05	3.915120722
HIST1H2BK	1.000199992	3.690969806	4.217764219	9.15060165590977e-05	0.000431925	0.771795447

Upregulated Genes in Suspended Samples (1671)

geneID	***logFC***	***AveExpr***	***t***	***P.Value***	***adj.P.Val***	***B***
EVX1-AS	-3.823146161	-0.631352148	-5.525821778	8.96245327054962e-07	7.6892089354248e-06	5.5697904
GOLGA2P6	-3.434017025	-2.214395747	-9.70842676	1.38920877277321e-13	1.41234444195055e-11	20.38985083
LINC01021	-3.280407099	2.261558384	-7.941700546	9.95309259688265e-11	3.21244766286317e-09	14.29298668
TRIM43	-3.275811596	-3.312337881	-7.88716086	1.22332685221746e-10	3.80425866878402e-09	13.9848445
PRAME	-3.153607701	-2.030955385	-6.930151372	4.61642617616989e-09	8.28239802830861e-08	10.56762756
TRIM43B	-3.068496755	-3.676474955	-8.100976343	5.45342866184728e-11	1.9349057693773e-09	14.75793406
CDC20B	-3.028710486	-0.227210756	-4.334481732	6.17072805373389e-05	0.00030683	1.574825079
MUC19	-3.014179903	0.16579926	-11.12593615	8.88097275120089e-16	2.19393226852797e-13	25.25580246
TRIM49B	-2.99083901	-2.650026433	-4.897263988	8.71654511467825e-06	5.57609963737392e-05	3.438463557
BIRC7	-2.884411677	-1.745024958	-6.89676291	5.2399007471662e-09	9.22145781956352e-08	10.44494195
ALMS1-IT1	-2.789479229	0.993469535	-8.531807928	1.07876610775294e-11	5.11021944914577e-10	16.42806828
LINC00894	-2.787803446	2.601022149	-8.413020289	1.68447194242272e-11	7.52122413075333e-10	16.01706107
MBD3L2	-2.771205226	-3.926985155	-7.580779824	3.90524425082035e-10	1.03955006326218e-08	12.91175199
AIF1	-2.6912598	-2.285321318	-5.99041759	1.59961895142342e-07	1.73147943255427e-06	7.208450563
CENPE	-2.668431981	3.682290137	-7.22288401	1.51932771361024e-09	3.29724051345315e-08	11.53609262
TMPRSS15	-2.641568519	-0.21689063	-5.735778501	4.12797940705633e-07	3.92217396357728e-06	6.307389284
CCDC152	-2.641402563	-0.090543629	-6.313091146	4.76563546013024e-08	6.16291791182107e-07	8.360689336
APOBEC3H	-2.636999792	-3.502297394	-6.492584683	2.42135362746022e-08	3.45505181083682e-07	8.993053337
C9orf173	-2.613654069	-1.912651976	-9.145683362	1.09674833979034e-12	8.07530609071809e-11	18.40629203
BLACE	-2.608391045	-3.398336926	-6.259621099	5.82818336537779e-08	7.34301100557822e-07	8.161541158
ATP1A4	-2.606175899	-0.807988533	-5.239530227	2.55015974322426e-06	1.90419131335161e-05	4.591351442
LCNL1	-2.59351568	0.391744173	-7.708482447	2.40644447424736e-10	6.96709242911333e-09	13.42272198
RGL3	-2.593206851	-1.997570053	-6.457346221	2.76604147860943e-08	3.88082666688338e-07	8.866325347
STX16-NPEPL1	-2.571133869	0.705379783	-8.728194342	5.17494685951819e-12	2.78593422276261e-10	17.11758544
MROH5	-2.564151201	-1.833608765	-8.666781072	6.50934019236232e-12	3.40278751929825e-10	16.73865457
LOC10012807	-2.542363587	-2.762882234	-7.052517161	2.90133002101295e-09	5.65491962965769e-08	10.98632274
SSPO	-2.516462843	3.357230546	-9.854032053	8.18163181125186e-14	9.40282928986176e-12	21.2250749
LINC00896	-2.510372584	-3.910043103	-7.616918559	3.40505364095 95e-10	9.3366994700708e-09	13.03991034
MAPK15	-2.496800897	2.433608883	-7.607741555	3.5256412437415e-10	9.58778549339703e-09	13.04834873
ANKRD20A19I	-2.48259242	0.314007912	-9.776178978	1.08557153793423e-13	1.14779649848862e-11	20.76250272
LPAR6	-2.476219842	0.24335671	-5.645420576	5.76734540106901e-07	5.22502759500997e-06	5.976579805
TBC1D26	-2.433701173	0.704785328	-11.5393283	2.12909319102919e-16	6.62097886099702e-14	26.7330406
LOC101927501	-2.431429466	-2.400766622	-6.252988711	5.97542970630331e-08	7.49637083183272e-07	8.135724322
FAM71E2	-2.429213411	-3.297396719	-7.440503672	6.6498441501165e-10	1.64276009738345e-08	12.37791004
NPEPL1	-2.422289055	0.488254045	-8.991620082	1.94094780574856e-12	1.29231922794337e-10	18.03922901
C9orf173-AS1	-2.420691183	-2.110229344	-7.868069375	1.31496379993129e-10	4.04638394919484e-09	13.90379042
C1orf168	-2.420411005	-0.092832271	-4.57603145	2.69320130449825e-05	0.000148559	2.343017555
LOC10050639	-2.41394982	-3.567000955	-7.514686518	5.01815424533589e-10	1.28906580337185e-08	12.656444
COL4A6	-2.410152151	4.750353578	-5.00164224	6.00818718718538e-06	4.03184594868929e-05	3.29863796
KDM4E	-2.403477355	-3.912516227	-7.623816817	3.3171370264443e-10	9.15259739248456e-09	13.06449052
ADCY10P1	-2.40220736	1.311856792	-11.12037748	9.05448541346687e-16	2.21608530494602e-13	25.47116226
BST2	-2.382840387	-1.289590938	-3.593386751	0.000691562	0.002544197	-0.637382068

CLEC4F	-2.379229777	-2.762574339	-6.492508309	2.42205236086734e-08	3.45505181083682e-07	8.984011769
FAM90A27P	-2.364146079	-3.443039346	-5.943740841	1.90428440708288e-07	2.01022163468138e-06	7.043770881
TIE1	-2.36033014	-0.729073938	-4.790372252	1.27251171199828e-05	7.77897828012273e-05	3.078494401
TRIM49	-2.341245074	-3.917081182	-7.017321264	3.31607628404077e-09	6.29461265660656e-08	10.88431206
LRRC74B	-2.325392067	-1.418875528	-5.797733065	3.28003806857888e-07	3.21830906706553e-06	6.530443671
ALPPL2	-2.322307704	-3.532318285	-4.826667634	1.11945139281986e-05	6.94937967740895e-05	3.203563993
GIPR	-2.319569207	1.078594341	-5.880898279	2.40715693743964e-07	2.46526072558473e-06	6.778463242
LOC101930452	-2.276201398	-0.700103959	-6.097902515	1.0697738044534e-07	1.23481794642431e-06	7.590357067
COL6A4P2	-2.267568116	1.014413336	-7.423962088	7.08076770090631e-10	1.73301789479682e-08	12.39980033
MPZL2	-2.251629716	-2.461308315	-4.684599374	1.84508243193073e-05	0.000107166	2.744361624
WSB1	-2.247287081	8.506118713	-12.78500454	3.28159431928225e-18	2.01726471259507e-15	31.1155264
LINC01573	-2.243688292	0.065597548	-11.39976856	3.43999521955368e-16	9.88362974331114e-14	26.13769498
LHX9	-2.227931547	4.469160398	-4.451111457	4.14423695768215e-05	0.000217067	1.454237516
LOC100131564	-2.223074765	4.164911908	-12.95558903	1.88219868119244e-18	1.21346726195024e-15	31.73577638
SLC25A21-AS1	-2.218920677	1.805055961	-10.31776008	1.53893693621338e-14	2.28532135027687e-12	22.80869468
KLK4	-2.214694302	-1.936382086	-4.478134606	3.7768062810392e-05	0.000199665	2.071637593
HESX1	-2.21466962	-0.388999868	-10.85913401	2.2576132672157e-15	4.93185880101757e-13	24.26904831
LINC00106	-2.212865576	-0.255683725	-12.70178537	4.30973508878333e-18	2.47650494786543e-15	30.09453445
MT1G	-2.202420833	-3.626287745	-3.708487828	0.00048197	0.001847971	-0.308244856
POTEF	-2.19616999	2.421059414	-3.785868362	0.000376897	0.001488277	-0.395398807
MSH5-SAPCD1	-2.182122332	-0.298836938	-8.249639459	3.11382411952503e-11	1.23744123013725e-09	15.32744698
LOC102467212	-2.178670049	-3.25721256	-6.706415553	1.07826173727074e-08	1.72007800249109e-07	9.750607373
LOC102723766	-2.171530885	-3.045038475	-7.084957292	2.5651201065934e-09	5.10700929237935e-08	11.09617537
ACSS3	-2.170776511	1.91721801	-6.577998269	1.75320955456599e-08	2.60644477816889e-07	9.265455563
CENPF	-2.165781334	5.817291986	-5.9494974	1.86380093396933e-07	1.97142257253347e-06	6.580976651
LOC100507661	-2.158609822	-0.577345914	-7.546288682	4.45113756732885e-10	1.15804054446066e-08	12.78561579
SCNN1A	-2.155242141	-3.057515931	-3.75456842	0.00041644	0.001624521	-0.150295189
LEUTX	-2.15176063	-4.615954258	-7.692144825	2.56015371684407e-10	7.34772455997169e-09	13.3424674
CCDC144B	-2.147655044	-1.680882573	-7.201775789	1.64612524964387e-09	3.52609633094298e-08	11.52296796
ASPM	-2.144492679	4.046389961	-5.660707324	5.45053653913827e-07	4.96807008065662e-06	5.714820639
LIN28A	-2.142880581	0.176314915	-4.334621348	6.16780391095371e-05	0.000306742	1.543033363
PTPRVP	-2.138720483	-2.339798838	-7.98271306	8.52371898837008e-11	2.82800461063530e-09	14.29440454
MECOM	-2.136420564	1.095194248	-4.013804971	0.000180117	0.000781908	0.453762724
ZNF83	-2.12809014	5.226992156	-16.68674846	2.44990999695446e-23	8.09480886868715e-20	42.84938641
GALNT5	-2.124991642	-1.501237238	-3.683031397	0.000522294	0.001983306	-0.369695223
GOLGA6L2	-2.122091483	-1.393238053	-6.954465585	4.20954216673462e-09	7.64747959404098e-08	10.64080352
GOLGA8A	-2.121287577	6.375726471	-12.06867986	3.52858600802465e-17	1.35175527463936e-14	28.7971293
CFTR	-2.11907935	-2.847167048	-4.450054122	4.15929974457996e-05	0.000217752	1.989688625
OR10AD1	-2.117861756	-3.583786333	-6.276118629	5.47738465909457e-08	6.9507301341261e-07	8.222245865
LINC00032	-2.117666625	-2.494341615	-5.190690359	3.04361809012363e-06	2.22034441959182e-05	4.434968101
MIR647	-2.116130216	-0.112941803	-8.87400637	3.00512717721293e-12	1.7863693982692e-10	17.56290302
SPATA21	-2.113716716	-2.047369951	-5.095338488	4.29310858715858e-06	3.00131550606619e-05	4.110515658
AMY2B	-2.105608811	3.261525939	-13.85310476	1.07430983784646e-19	1.09220122860752e-16	34.47488669
LINC01054	-2.100543465	-3.229003741	-5.471065154	1.09586919630723e-06	9.12349935936665e-06	5.393750308
TAC3	-2.091764293	1.226469127	-3.158776914	0.002559039	0.007959881	-2.037396569
AGBL2	-2.089176121	-2.002197634	-6.594134288	1.64939610930382e-08	2.47437499189715e-07	9.347425471
AASS	-2.088606095	4.807380728	-8.060942236	6.34300926396518e-11	2.19744120412047e-09	14.56710981
PVRL4	-2.080259043	-1.865110521	-5.327866923	1.84975331601961e-06	1.44658371012859e-05	4.901376483
ANPEP	-2.074916623	-1.764605612	-3.834871801	0.000322134	0.00130079	0.080357193
MAN2C1	-2.073658147	5.949442632	-9.149745737	1.08039470960611e-12	8.01730925295449e-11	18.52575192
LINC00893	-2.071559375	2.986333916	-8.557279903	9.80575441187779e-12	4.7298450067366e-10	16.53138488
NOXA1	-2.065576279	1.533417658	-6.849173764	6.27654080824833e-09	1.07244863079785e-07	10.28017111
INSRR	-2.060764282	-1.547816193	-4.881890267	9.20560363868714e-06	5.84932021589945e-05	3.392555393
ATP6V0D2	-2.054558402	-3.766972488	-4.426970963	4.50175318822067e-05	0.000233461	1.892078562
RAD51AP2	-2.046539368	-2.876244114	-5.681999698	5.03777800385927e-07	4.64956654944177e-06	6.12512181
MIR6845	-2.043138788	-1.714949952	-7.561671373	4.19881958309386e-10	1.10325445367714e-08	12.80565549
FER1L4	-2.038514219	2.596146917	-6.110996271	1.01852973983663e-07	1.18341963134513e-06	7.501455546
C1QTNF9B	-2.037720187	-2.449842776	-6.062532944	1.22135702192827e-07	1.37848548935226e-06	7.458469134
GMNC	-2.034790828	-1.703467728	-3.227454554	0.002093802	0.006688275	-1.634436066
ANKRD18A	-2.033717179	1.089664244	-6.074783412	1.16658266297804e-07	1.32739801361517e-06	7.473737539
LOC100507250	-2.03159596	-1.320765613	-5.784436778	3.44610103428126e-07	3.36874218340076e-06	6.483661414
HNRNPU-AS1	-2.022567722	4.723401502	-9.902273143	6.86873835169008e-14	8.06939381556551e-12	21.34165727
TBC1D28	-2.020982964	-3.603642591	-6.521131498	2.17375893294854e-08	3.15535254665727e-07	9.096344217

COL4A5	-2.014854504	5.767942456	-5.621937811	6.28984075332151e-07	5.63400069917139e-06	5.385187845
PRODH2	-2.013410372	-3.358364598	-6.203550851	7.19601387277696e-08	8.75746936920412e-07	7.961639207
LOC100506274	-2.000879397	-1.242510153	-8.190412813	3.89216307598775e-11	1.45967322000878e-09	15.04708158
HAVCR1	-1.997508288	-3.517941982	-5.213925209	2.79813226982517e-06	2.06197463864758e-05	4.50836112
POU5F1P3	-1.995720162	-1.435212603	-5.634726653	5.999735401059e-07	5.40528308984978e-06	5.960978877
TRH	-1.992284106	4.249474372	-5.017069804	5.68546756843724e-06	3.83181958787613e-05	3.399907419
LMNTD2	-1.984256328	4.016261066	-8.630602669	7.45223624544935e-12	3.73077577037808e-10	16.74514714
LINC00173	-1.977797293	1.75013284	-9.180129601	9.65614468190519e-13	7.31349204518051e-11	18.79880166
PRSS37	-1.968717702	-2.871331432	-5.589576002	7.08739803829059e-07	6.26372049789969e-06	5.804250244
IFITM5	-1.964256503	-3.803261547	-7.042708942	3.01140101270351e-09	5.82725936813995e-08	10.97148176
PLA2G4D	-1.96075929	-4.142391392	-6.628082486	1.45056151901024e-08	2.21762247726997e-07	9.488965466
SERPIND1	-1.960364002	-2.972156109	-3.6769466	0.0005324	0.002016467	-0.372700128
LINC01480	-1.957112931	-4.14864058	-5.080619015	4.52645811279964e-06	3.13871635088229e-05	4.042278099
OR7E12P	-1.954296212	0.346820855	-6.399768364	3.43741197987899e-08	4.66432807310787e-07	8.669817661
LST1	-1.953907947	-2.846518987	-4.996316884	6.12371046197765e-06	4.08964220923333e-05	3.780890919
COL7A1	-1.945884356	5.439879532	-13.39245364	4.61097161057069e-19	3.65367434487612e-16	33.13239045
KLHL6	-1.944522923	-2.332561066	-4.486726202	3.66678618512872e-05	0.000194393	2.106667726
NAALADL2-AS	-1.943132902	-2.284148537	-4.460827082	4.00829289486043e-05	0.000210692	2.023355798
UCP1	-1.937752365	-2.93468435	-6.631401726	1.43245256210023e-08	2.19501556950698e-07	9.47429187
MIS18BP1	-1.937676697	3.016111502	-5.92983091	2.00574366340175e-07	2.1072266396939e-06	6.799779976
CEP290	-1.937628892	5.545718382	-11.00388853	1.35938922158712e-15	3.17988807913383e-13	25.19264336
LCN12	-1.937279683	-1.268014873	-9.779604678	1.07213215931205e-13	1.14272860351192e-11	20.54502863
LINC00958	-1.936522902	-1.331985751	-8.686336046	6.05061394102894e-12	3.18597367137884e-10	16.78571961
LOC102723828	-1.931448846	-2.560607799	-3.508615707	0.000898876	0.00320691	-0.84860172
SNORD117	-1.929014171	-1.11018981	-6.833469325	6.66171271098308e-09	1.13022498131846e-07	10.20682115
TRAF3IP3	-1.925712128	-3.637248425	-5.392399552	1.46166125217817e-06	1.17542110979086e-05	5.119151867
USP32P1	-1.920366414	-0.757731383	-6.215850972	6.87094185433088e-08	8.42441218621929e-07	8.008647314
SLFN13	-1.920151125	-1.715735531	-3.982297399	0.000199711	0.00085614	0.525207548
LINC01363	-1.918170147	-3.758942642	-4.902789832	8.54706359208951e-06	5.48626838100296e-05	3.452482033
ANKRD33	-1.916222597	-1.99116723	-6.047985716	1.28972604491068e-07	1.4430301701224e-06	7.407978538
ZGRF1	-1.915914738	2.983012809	-8.225816461	3.40611297257883e-11	1.31436181319965e-09	15.30824561
LOC100996634	-1.914599343	-3.154364966	-5.148939482	3.53914662868507e-06	2.53317798093779e-05	4.291997249
HRAT56	-1.91372729	-4.205624367	-6.567432322	1.82468947859671e-08	2.69904963557621e-07	9.271879032
FAM166A	-1.912839584	-1.623159659	-6.280102447	5.39587142927492e-08	6.86707123206663e-07	8.230516966
CSAD	-1.909420184	4.82565201	-12.11740234	2.99593920272533e-17	1.18196508874087e-14	29.00578857
CACNA1F	-1.907047733	-2.956420049	-5.086803275	4.42693673476204e-06	3.08264538224354e-05	4.084080693
MST1P2	-1.906782444	1.422256963	-7.345885795	9.52378145975994e-10	2.20826416952486e-08	12.11011636
HBA2	-1.903536135	-4.377868826	-5.958964517	1.79907147530427e-07	1.91444671122052e-06	7.096880216
KIF14	-1.902484941	2.388989399	-5.643970408	5.79832917742122e-07	5.25067609272953e-06	5.827048188
CD300LG	-1.899747066	-3.100592733	-4.721232043	1.62284920721404e-05	9.58587108252262e-05	2.865043176
PDK4	-1.899060003	0.674825915	-6.121319879	9.7985256640955e-08	1.14350741226948e-06	7.657541525
LOC100270804	-1.895757934	1.077542845	-10.85234602	2.31210466428077e-15	5.00949693368309e-13	24.5492587
CYP3A4	-1.894532718	-1.200176055	-7.253365117	1.35326441258789e-09	2.98338934261348e-08	11.70737188
LINC00202-1	-1.889753646	0.24457103	-5.781331148	3.48607315099498e-07	3.3996791600411e-06	6.455646524
MIR1914	-1.889240207	-0.046253427	-7.185220034	1.7529370494712e-09	3.71873074066391e-08	11.5114256
FAM132A	-1.887203811	1.527201446	-7.854659933	1.38341355824371e-10	4.23238085475186e-09	13.97926976
TRIM49C	-1.885801605	-4.188200349	-5.078127308	4.5671753218e-06	3.1636306415393e-05	4.032845138
LOC101927437	-1.883079675	-2.709129631	-4.988308803	6.30154929406243e-06	4.18830406059724e-05	3.755085811
LOC101409256	-1.883063151	-2.496102134	-6.341500994	4.28192760190221e-08	5.63710164188306e-07	8.442435227
AHSA2	-1.880563299	5.264344217	-14.3514468	2.28997596125673e-20	2.63177976451735e-17	36.10668142
GOLGA6A	-1.879919326	-1.873776693	-4.962649681	6.90622758060617e-06	4.54562533959569e-05	3.665636449
CHRNE	-1.879076129	-1.399128232	-6.485282177	2.48907990796642e-08	3.53730372082131e-07	8.960722567
DDX12P	-1.876226172	2.662357369	-6.068453194	1.19457467177853e-07	1.35345873549601e-06	7.338364912
FAM111B	-1.87504628	2.198802553	-5.417860227	1.33171411203183e-06	1.0844485250566e-05	5.038804861
SAP25	-1.874162873	2.160880607	-7.495490875	5.39731902263044e-10	1.37576985270193e-08	12.64060444
PTGES2-AS1	-1.873182364	-1.890106987	-6.747499647	9.22832950803667e-09	1.49835647350082e-07	9.89144893
UACA	-1.87312513	3.957364009	-6.870759922	5.78312854134159e-09	1.00437212045521e-07	10.18209871
ADAT2	-1.872731602	2.733435132	-9.211818106	8.58952969197035e-13	6.61944718215313e-11	18.92507626
FMOD	-1.872317416	-2.052451096	-3.381997597	0.001321645	0.004498461	-1.205763159
LINC01198	-1.871931432	-3.592565251	-3.860769908	0.000296367	0.0012108	0.14350816
SPTLC3	-1.869742484	-2.39703661	-3.951729049	0.000220676	0.000935396	0.441827076
LOC101929239	-1.869488384	-3.47384657	-3.607077892	0.000662689	0.002451281	-0.594034392
LINC00441	-1.865557371	-0.335192402	-8.59570741	8.49164656464289e-12	4.18768085155234e-10	16.55553708

ADAMTS20	-1.861940946	0.923839374	-3.932129023	0.000235218	0.000991157	0.215984445
LOC440910	-1.859266119	-1.460812755	-4.785169472	1.29606874505386e-05	7.89013936849579e-05	3.073798539
HSF4	-1.859106268	3.722431921	-8.782503369	4.2257559651144e-12	2.38673947491173e-10	17.3223425
ATHL1	-1.8581292	2.295366664	-8.882103544	2.91592703419264e-12	1.7398803452554e-10	17.73290757
THRB-AS1	-1.855915164	-3.438524154	-6.106168225	1.0371347276251e-07	1.20292155573998e-06	7.617352824
SCART1	-1.855138033	2.216204403	-7.0636103	2.78167614608074e-09	5.47083672391013e-08	11.03853391
GNRH1	-1.852165356	1.059855271	-5.400224495	1.42044561104217e-06	1.14611229660188e-05	5.069485536
MIR548AR	-1.849250807	-1.374128393	-5.624637978	6.2274558387556e-07	5.58948523551194e-06	5.925968196
PIF1	-1.84638904	1.776782135	-4.488222978	3.64794135565632e-05	0.000193627	1.895458072
PCOLCE-AS1	-1.841722101	-1.002278957	-6.449645996	2.84763863862314e-08	3.97841607477408e-07	8.836672451
MMRN1	-1.840566726	2.861072936	-5.791086296	3.36203665481906e-07	3.29387379158014e-06	6.310581765
NUF2	-1.837473266	3.185318483	-5.770497695	3.62913852260564e-07	3.51259679853661e-06	6.202547174
LOC399715	-1.836392748	-0.148143601	-5.376837938	1.54716661969989e-06	1.23218605780437e-05	5.048841299
TNFRSF13C	-1.835501719	-0.612736528	-5.873575595	2.47372010431398e-07	2.5226791480452e-06	6.796900293
BCL6B	-1.835122405	-2.272668628	-4.53112041	3.14623891067584e-05	0.000170314	2.250668135
EDNRB-AS1	-1.835041467	-0.266102544	-5.729567214	4.22412598584581e-07	3.99772009251208e-06	6.285360309
USP43	-1.831327489	1.231187466	-9.499211094	2.98406048543391e-13	2.7105728801194e-11	19.9052444
CEP152	-1.830271564	2.347805169	-6.538858084	2.03287850074228e-08	2.97207286560401e-07	9.090236665
CMTM5	-1.827109195	-2.358735831	-4.486472822	3.66998568453172e-05	0.000194484	2.10768283
GOLGA8B	-1.825209266	5.526060854	-17.7280464	1.42031685330826e-24	1.19850012142001e-20	45.67197497
LOC100129216	-1.824566242	-1.962709486	-6.25526346	5.92451559076187e-08	7.45017700335911e-07	8.139720294
FBXO22-AS1	-1.822617015	-4.481583321	-7.912347371	1.11215568834467e-10	3.50854439611237e-09	14.12906301
HDC	-1.822595936	-2.50465333	-4.300654152	6.9203879572011e-05	0.00033869	1.518955847
MBD3L3	-1.82257228	-4.640282623	-6.018297915	1.44126389150473e-07	1.58539028065521e-06	7.3056613
WDR87	-1.819447096	-1.769476829	-5.185422997	3.10215309236197e-06	2.257687574075e-05	4.416610757
CYP3A43	-1.818643777	-1.772559894	-4.427187033	4.49842382171459e-05	0.000233334	1.913446496
AMY2A	-1.816583288	-4.05095855	-5.27930878	2.20723900781264e-06	1.68478049937949e-05	4.723609593
CYP2F1	-1.81484104	-3.196265936	-5.676197764	5.14708029249947e-07	4.74051475162504e-06	6.105413149
MIR6878	-1.81361462	-3.183868805	-6.430645979	3.05937583678428e-08	4.22289720593832e-07	8.765396361
TRIM6	-1.813366661	-2.538962212	-4.389167673	5.12272330491909e-05	0.000261054	1.799206492
LINC00854	-1.812704358	-3.065549496	-4.799171036	1.23362369418462e-05	7.5780560326242e-05	3.122411153
PLAG1	-1.811382266	3.57988252	-8.184011094	3.9872141317558e-11	1.48861625910595e-09	15.11755206
FAR2P1	-1.809341859	4.546023109	-4.043442776	0.000163389	0.000718852	0.112746514
CHMP4C	-1.807330208	-2.237721606	-4.153198101	0.000113566	0.000521794	1.056523027
KIF18A	-1.805373948	2.193158116	-5.493308164	1.00996992833767e-06	8.48856442472164e-06	5.306281117
OCLM	-1.800112245	-1.450777158	-5.000447856	6.03390934262128e-06	4.04499938253889e-05	3.791060295
ERVH48-1	-1.799378367	-0.132117977	-4.451488438	4.13887934738423e-05	0.00021694	1.93814807
ADAM33	-1.797126446	-0.814821524	-3.493284918	0.000942198	0.003346114	-0.922451962
MIR4712	-1.795722906	1.752958669	-9.546418762	2.51025549875636e-13	2.3281959157413e-11	20.10621639
OTX2-AS1	-1.794812274	-1.607984546	-3.140148317	0.002701053	0.008343689	-1.859235881
CCDC144CP	-1.78995091	1.87518374	-9.958507102	5.60357690370063e-14	6.79446551814307e-12	21.56672109
MYO15B	-1.789709023	3.370042445	-7.54639163	4.44939946794817e-10	1.15804054446066e-08	12.75752116
ZSCAN4	-1.788617753	-3.495648999	-4.614502719	2.35628922059069e-05	0.000132716	2.506924801
MIR125B1	-1.786102771	-0.427815457	-5.810285432	3.13055215384321e-07	3.09692683692132e-06	6.572266936
MANEA-AS1	-1.785408018	1.105091423	-12.98423706	1.71506553087219e-18	1.13335817943861e-15	31.40087161
MST1L	-1.784674789	0.173980174	-8.873807167	3.00735588934208e-12	1.7863693982692e-10	17.59613702
GPR182	-1.775428893	-2.145166898	-4.96847695	6.76409015033956e-06	4.46207124891255e-05	3.687415975
NLRP12	-1.774710549	-3.211001499	-3.880794418	0.000277815	0.001144719	0.218378126
DQX1	-1.774396261	-0.552457101	-8.938519152	2.36410036603609e-12	1.50066403906644e-10	17.73431824
MIR3149	-1.772233907	-3.642134053	-4.967024366	6.79925013937387e-06	4.48190969910398e-05	3.670519477
LOC101927765	-1.771132848	0.223248639	-12.61159083	5.79661592669627e-18	3.26004146362473e-15	29.99583409
VWA2	-1.77010426	-0.594647697	-8.130191547	4.8842850783403e-11	1.76857955446259e-09	14.87484579
ZNF441	-1.769481026	4.62024815	-5.509466198	9.5177531798633e-07	8.08947812872433e-06	5.094974125
TTC14	-1.768497273	6.080729481	-14.39855789	1.98184093716619e-20	2.49457149962448e-17	36.24769997
SMC2-AS1	-1.766763367	-3.116280366	-5.47234839	1.09072304144628e-06	9.08637950033076e-06	5.39870328
MUC1	-1.766349739	1.922779272	-10.19265918	2.40961699806789e-14	3.28316526339838e-12	22.38803678
LRRC70	-1.765464795	-1.007970354	-7.957479224	9.37671886785999e-11	3.06371829214021e-09	14.22145411
LOC100506142	-1.764539783	-0.409414896	-5.537927725	8.57217504332567e-07	7.39759395756538e-06	5.616486174
CCDC39	-1.761994327	3.457207796	-12.27389578	1.7748504991699e-17	7.95163105839964e-15	29.50897342
LXN	-1.761310689	-1.248771822	-6.602735733	1.59658473596766e-08	2.40881988161147e-07	9.37878573
SERHL	-1.760216593	1.064624355	-11.29894685	4.87241424031833e-16	1.35490595580499e-13	26.03533794
FAM72C	-1.757737639	-0.670035339	-5.32852216	1.84534285016306e-06	1.44441656968789e-05	4.896810137
ZNF117	-1.757546595	5.498723721	-9.721218378	1.32595688971984e-13	1.36377503758617e-11	20.63598141

NPHP3-ACAD1	-1.755835872	0.008332323	-4.842616082	1.05804480010785e-05	6.61009175165466e-05	3.217779047
MIR4674	-1.755506494	-3.285646679	-4.79620538	1.24659913711137e-05	7.64177991448626e-05	3.108460794
LINC00899	-1.74972102	-1.841648728	-7.028444843	3.17896571509258e-09	6.09351709550705e-08	10.89214383
MIAT	-1.749662983	10.43782451	-8.651301024	6.89715032150942e-12	3.54693335502838e-10	16.51520659
ACTG1P17	-1.747117351	-1.023808262	-6.810790486	7.25994112321782e-09	1.21073831993701e-07	10.12505807
TCP10	-1.742034438	-4.326763427	-5.383661366	1.5090831493897e-06	1.20694689524412e-05	5.078032798
MEG3	-1.739693694	-1.070796732	-4.091000149	0.000139638	0.000624859	0.853081943
CCDC144A	-1.735840883	-1.272206887	-6.275936664	5.48113696414612e-08	6.95215419257554e-07	8.215691767
LOC102724814	-1.735647809	1.636154255	-10.3433827	1.40421380470106e-14	2.12100477140931e-12	22.8880745
MAP3K19	-1.735317484	-2.285125055	-3.328528205	0.001551755	0.005162685	-1.344974539
IFITM1	-1.732117207	2.436496395	-3.617438105	0.000641609	0.002380637	-0.906786142
CLDN18	-1.732027791	-1.903268404	-5.362329034	1.63132516396682e-06	1.28949814770141e-05	5.020932945
GOLGA6C	-1.728803646	-2.05251243	-4.613254286	2.36654578147908e-05	0.000133209	2.516059997
SLC5A12	-1.726475454	-2.425649005	-4.702814364	1.73109394094789e-05	0.000101347	2.810698938
DAPL1	-1.725998078	-0.467385481	-4.172384268	0.000106522	0.000493636	1.072660272
AP1G2	-1.72371431	4.004821474	-16.34476087	6.40864283413679e-23	1.53999687304307e-19	41.79955636
INO80B	-1.722011983	-0.247439258	-12.05792401	3.6585349269984e-17	1.38151505321927e-14	28.138378
COL13A1	-1.720283353	0.709686295	-3.507154903	0.000902921	0.003219169	-1.020614714
LINC00624	-1.719793834	-3.602474028	-4.506840865	3.42128407769144e-05	0.000183263	2.15475989
MIR6832	-1.713167743	-3.949827819	-6.074675652	1.16705367670777e-07	1.32739801361517e-06	7.508684576
PABPC4L	-1.711961803	0.055242686	-4.920533441	8.02442203122583e-06	5.18858971505363e-05	3.477132003
DDX60	-1.71182204	0.146821335	-4.417677809	4.64724224812547e-05	0.000240204	1.809844729
CHAD	-1.710413937	-0.965916002	-4.025253473	0.000173468	0.000758024	0.648584945
LVCAT8	-1.709507609	-3.851115247	-5.010738306	5.81579778486355e-06	3.91267454434457e-05	3.813193869
LOC100129617	-1.709238344	-0.299278714	-6.190200329	7.5661667422545e-08	9.11976678057515e-07	7.920668991
LINC00115	-1.707685247	0.806107617	-10.40923103	1.11002392048105e-14	1.78338679869906e-12	23.01372192
HRC	-1.707629534	0.159198778	-4.783678512	1.30289786185651e-05	7.92259010408399e-05	3.01164348
TRIM59	-1.706256225	2.676014551	-5.17736474	3.19385415080363e-06	2.31486555437873e-05	4.141011146
FLJ37201	-1.701880416	0.899312316	-10.90347437	1.93218741776344e-15	4.25612583447843e-13	24.6949629
ZEB2-AS1	-1.69972697	-3.264499646	-4.63950299	2.15980628073738e-05	0.000122907	2.594224929
MORF4L2-AS1	-1.698337134	1.163576365	-7.556703761	4.27869545895252e-10	1.12089947538644e-08	12.88660275
DDX4	-1.697956246	-2.859398544	-4.846451391	1.04377740435211e-05	6.53021730869567e-05	3.282129773
ACTG1P4	-1.69267214	0.20663438	-5.798199559	3.27435810755157e-07	3.21512287729981e-06	6.516245883
SLURP1	-1.690483004	-4.142758892	-4.730387517	1.57153355487541e-05	9.32443242559408e-05	2.868265286
LOC101927583	-1.690297667	0.282222861	-9.832556083	8.8448782675704e-14	9.92951626699285e-12	20.96140466
GOLGA6L5P	-1.688486628	-0.890567612	-4.709650396	1.69011702209164e-05	9.9233370157593e-05	2.818755358
TCTEX1D4	-1.6876473	-1.708172427	-5.370257875	1.58478951501867e-06	1.25800859114187e-05	5.047924034
TSPO2	-1.685191283	-1.835075055	-4.205925563	9.5212681934246e-05	0.00044814	1.218443013
MYCBPAP	-1.681335973	-1.341763097	-5.674195272	5.18534737542477e-07	4.76911228860831e-06	6.09838239
MIR3192	-1.680680791	-3.095585759	-5.238928293	2.55573270984128e-06	1.90781368876686e-05	4.598454168
GOLGA6B	-1.680296826	-1.167466086	-5.441609818	1.22081373953213e-06	1.00546383571057e-05	5.292345869
LOC100507053	-1.680054359	2.045169755	-8.731098279	5.11915051249481e-12	2.76716780157005e-10	17.18395912
BTBD8	-1.678442352	-0.656376725	-7.876097247	1.27562072032142e-10	3.93907505844112e-09	13.96280457
TMA16	-1.676525965	3.474276269	-7.840397604	1.46014258304302e-10	4.43447333022487e-09	13.84530393
ARL5C	-1.675704101	-2.777767195	-5.693846803	4.82167657611889e-07	4.46618841351568e-06	6.165771224
C16orf89	-1.674728601	-1.601353252	-4.417602476	4.64844012700247e-05	0.000240219	1.883631141
EQTN	-1.673557464	-3.827452238	-5.179733493	3.16662481936312e-06	2.2989122178035e-05	4.387016919
PMEL	-1.67265687	2.07721436	-4.623712811	2.28195355818453e-05	0.000128914	2.310094113
ZFC3H1	-1.672548297	5.713909939	-12.00039517	4.44042219916766e-17	1.6531504224028e-14	28.5865191
SLC39A5	-1.672367356	-0.651134653	-6.193437021	7.47472516210083e-08	9.03658669120439e-07	7.9304349
LOC101927131	-1.670562093	-3.4715765	-3.585745909	0.000708193	0.002598872	-0.654598665
STRC	-1.668373361	0.81926938	-6.200254198	7.28570720356639e-08	8.85439533387909e-07	7.936612391
C9orf135	-1.667760071	-3.296707629	-3.730665268	0.000449286	0.001737778	-0.228426223
MIR3916	-1.667181245	-0.156764976	-7.161275891	1.91979163315185e-09	4.00203882011852e-08	11.42134794
STPG2-AS1	-1.666218944	-3.060114674	-3.817034918	0.000341118	0.001367422	0.034457589
UNC13D	-1.663844458	1.54059803	-8.498980255	1.2200314449049e-11	5.6515194553195e-10	16.33432085
ATAD5	-1.662390472	3.696291397	-6.955104406	4.19934917741839e-09	7.63420885878269e-08	10.51953187
UBA7	-1.66154599	0.816543226	-7.610023634	3.49526041696743e-10	9.52476480429898e-09	13.07898728
LOC101241902	-1.661019543	0.770273377	-9.987663808	5.04293633318649e-14	6.17129333773697e-12	21.56488612
CORO6	-1.660212131	1.135641617	-12.16308187	2.57053619830345e-17	1.09591908596379e-14	28.84999087
ZNF436-AS1	-1.659075551	3.319066112	-11.17035622	7.60971952939675e-16	1.95289044971402e-13	25.81949476
CCDC14	-1.657047366	6.120881726	-10.74650267	3.35604840265324e-15	6.77178835323153e-13	24.26346638
LOC100507487	-1.656358595	-0.662477157	-5.897131986	2.26585912660341e-07	2.34141729059843e-06	6.880109652

LOC440896	-1.654254664	-1.958509547	-5.202416615	2.9171847707474e-06	2.14015945171152e-05	4.475774842
COLCA1	-1.653668726	-1.058953489	-6.498530474	2.36756812062578e-08	3.39013695192315e-07	9.008718401
LOC101927865	-1.651846751	-3.0411457	-4.800312538	1.22866469883701e-05	7.5493552642569e-05	3.12670868
HMGB2	-1.651727474	6.975924623	-6.871172233	5.77409096883932e-09	1.00346184470302e-07	9.92995105
TMEM92	-1.650676874	-3.550728437	-3.60460214	0.000667823	0.002468546	-0.604128387
FAM95C	-1.65042646	-0.188020179	-4.508893232	3.39715496061468e-05	0.000182181	2.127199672
LOC100130370	-1.649445962	0.734493689	-6.252059118	5.99636115703153e-08	7.51906140720182e-07	8.126733393
LOC645752	-1.647559355	-2.840388656	-3.180863342	0.002399752	0.007528203	-1.7399069
MIR1302-3	-1.645638378	-1.96016831	-4.405710145	4.84135898152789e-05	0.000248682	1.849348955
C11orf21	-1.645457066	-3.346747383	-4.22868968	8.82096946951054e-05	0.000419171	1.278374948
PSTPIP1	-1.637852855	-1.184363946	-6.795011933	7.70749231700606e-09	1.2797245252225e-07	10.06391711
TMEM92-AS1	-1.636427628	-3.527428208	-4.50682358	3.42148800392327e-05	0.000183263	2.157002027
ZAN	-1.634521955	-3.964159466	-3.761844957	0.000406907	0.001591325	-0.166452392
RDH10-AS1	-1.634058343	-2.22156269	-4.308154762	6.74688878017601e-05	0.000331365	1.542976146
SEC31B	-1.633499183	4.778090746	-8.821134111	3.65905367685921e-12	2.11640625471377e-10	17.38878981
UPB1	-1.632102655	-0.93230447	-6.736440369	9.62327741574683e-09	1.55199567986843e-07	9.861815418
DEPDC1	-1.631770178	2.462818871	-4.803008291	1.21703100295236e-05	7.48656748918772e-05	2.871276527
MYO1A	-1.62995408	-3.735081027	-4.381950534	5.25040403074043e-05	0.000266636	1.74985076
PDE6C	-1.628078939	-0.625052102	-7.320586263	1.04840210709971e-09	2.40977503451884e-08	11.97348248
AMT	-1.625337631	3.68423393	-8.843231433	3.36992870328666e-12	1.97073728791983e-10	17.54601537
C18orf54	-1.623983786	3.12196181	-7.629049591	3.25196647099507e-10	9.01040143897407e-09	13.08241486
SELV	-1.621694031	-0.400874048	-7.403698842	7.64692116867259e-10	1.83922718154252e-08	12.28567407
SALL4	-1.621622694	2.100196742	-3.676483965	0.000533176	0.002018828	-0.690867361
KIZ-AS1	-1.621056551	-2.775699758	-5.155124242	3.46102055109759e-06	2.48263653262313e-05	4.316514682
SNORA76A	-1.615936433	-3.366125913	-4.510434033	3.37914931373822e-05	0.000181436	2.173127485
SIGLEC10	-1.613861002	-0.114549819	-4.552330115	2.92371410431778e-05	0.000159345	2.264016701
LOC728175	-1.613734773	-2.122711728	-6.319211633	4.65703165977155e-08	6.03428028738928e-07	8.362483329
NRBP2	-1.613110168	6.219697388	-23.46673049	1.44497908183652e-30	3.81951320701847e-26	59.22672107
SLCO1A2	-1.612954052	0.240250682	-4.182717333	0.000102906	0.000478725	1.05367503
RFX3-AS1	-1.612129544	0.846153922	-8.209708678	3.61920264452914e-11	1.37649472665955e-09	15.26341364
DDX43	-1.61095757	-4.039398982	-4.892466706	8.86635147800707e-06	5.65687348824912e-05	3.410967753
TFPI	-1.609382912	0.458270809	-4.629116966	2.23940787312e-05	0.000126754	2.477466311
CYP2D7	-1.608849187	-3.742829953	-5.117119961	3.9693581444433e-06	2.80315372247047e-05	4.175553594
PRTG	-1.608396264	4.706359167	-4.706052184	1.71156589303864e-05	0.000100417	2.263027492
LINC00632	-1.607809929	3.856328806	-7.585376578	3.8377449989234e-10	1.02571398944937e-08	12.86129604
TRIM51	-1.607515054	-4.620647074	-5.338010476	1.78262668216317e-06	1.40071852228356e-05	4.91262344
DNAH12	-1.606830964	0.282925577	-6.037066582	1.3435281771402e-07	1.49153634213973e-06	7.365579343
GOLGA4	-1.606434689	6.371269247	-7.533904899	4.6652567393777e-10	1.20662163788621e-08	12.45861512
UTAT33	-1.60601975	-2.036645077	-6.080705511	1.14098550318914e-07	1.30304719300527e-06	7.520656513
BRDT	-1.60578991	-4.267563038	-4.622010509	2.29551822192166e-05	0.00012957	2.506983583
LINC00482	-1.605157608	-1.748379442	-4.78721057	1.28677662561672e-05	7.84803104405323e-05	3.084993995
SLC9C1	-1.604800473	-3.777230269	-4.339562357	6.06518196553934e-05	0.000302721	1.613489907
PNISR	-1.603329171	7.662710178	-13.43542434	4.02049412828665e-19	3.32105379040628e-16	33.22608812
LOC102724890	-1.60277445	-2.827907468	-5.259027234	2.375982483841e-06	1.79459127380765e-05	4.668811331
LYPD2	-1.601854048	-3.704166573	-4.19141847	9.99536240158581e-05	0.00046721	1.149700426
CNPY1	-1.600016033	3.601819445	-6.01616742	1.45279491002544e-07	1.59475614022851e-06	7.049756386
IRS4	-1.599725972	1.87411525	-4.456222399	4.07217257615632e-05	0.000213656	1.776935511
SAPCD1-AS1	-1.598609453	-3.657205518	-4.895202872	8.78060157739997e-06	5.60757771189692e-05	3.429803456
ZAP70	-1.598515446	-1.464404167	-5.548264907	8.25225041896722e-07	7.16125198045176e-06	5.661221992
BTNL2	-1.595982438	-3.162504551	-3.552853839	0.000784245	0.002843225	-0.734926872
RHOXF1-AS1	-1.59573779	-1.277796269	-4.587633134	2.58693168883527e-05	0.000143631	2.429232438
HCCAT5	-1.593127577	-2.829050192	-3.907681213	0.000254655	0.001060548	0.311942466
MIR378I	-1.592494471	-2.039671632	-4.541973417	3.03038410161066e-05	0.00016465	2.287193985
MSLN	-1.592461315	-2.449405035	-4.257808783	7.99768562951465e-05	0.000385209	1.388262271
LOC101929679	-1.591665133	1.388483137	-5.954741336	1.82766632175507e-07	1.93785414692947e-06	7.021339769
ABI3BP	-1.590809163	0.014547698	-5.590151292	7.07238242188672e-07	6.25440898486891e-06	5.787604916
KIF15	-1.589616849	3.876919613	-5.143795011	3.60545204883263e-06	2.57714748531079e-05	3.880250385
ITLN2	-1.588650727	-3.270021458	-3.804937168	0.000354601	0.001412685	-0.008973405
FOXS1	-1.588167887	-1.462321282	-4.946433776	7.31737783300306e-06	4.78762990742004e-05	3.612229149
RDM1	-1.587149173	0.300469982	-5.88320194	2.38658623021127e-07	2.45084047487081e-06	6.815485501
LOC642846	-1.586591094	2.414237391	-3.558787381	0.000769974	0.002797624	-1.077255488
PKN2-AS1	-1.584700888	-3.309797718	-5.340569815	1.76607302807168e-06	1.38977696787791e-05	4.944165529
GALNT3	-1.584604996	-0.951481456	-4.446837565	4.20545139877472e-05	0.000219776	1.9678105

BTAF1	-1.583411597	7.026571578	-10.46520497	9.09313121292912e-15	1.54076113686766e-12	23.23389788
HSPB2	-1.582732697	-3.254699658	-4.656371662	2.03637549021843e-05	0.000116839	2.649618827
FAM72D	-1.581992001	0.141249389	-5.487141313	1.03309556281931e-06	8.67465534053459e-06	5.422012936
SNORD124	-1.580670905	-1.949747953	-4.915545142	8.16809824617351e-06	5.27246253824431e-05	3.511556291
LOC102723769	-1.578663857	-3.861112229	-3.822301878	0.000335403	0.001347988	0.017631196
FRY-AS1	-1.577598374	0.376071646	-8.66659508	6.51386707814065e-12	3.40278751929825e-10	16.87914235
LINC01355	-1.575411922	1.914088896	-6.450478912	2.83869808713111e-08	3.96864912325769e-07	8.792850157
LOC101929378	-1.575139736	1.436339621	-6.23641627	6.35972646432187e-08	7.88862738767808e-07	8.039001761
POMC	-1.574525932	-1.788408923	-3.537783563	0.000821634	0.00296576	-0.767817454
DUOXA1	-1.574456573	-2.177422252	-3.905182431	0.000256727	0.001067325	0.306112543
DMRTA1	-1.574423012	0.426150614	-3.983963616	0.000198625	0.000852591	0.419922781
TOP2A	-1.574281969	6.841415687	-4.571755707	2.73343148781383e-05	0.000150245	1.614468155
LOC644656	-1.572451582	0.896932181	-12.14910546	2.69377306134673e-17	1.13023021159648e-14	28.75193471
BRCA2	-1.572450624	2.091032463	-4.733323907	1.55541422417804e-05	9.24599155314466e-05	2.675358854
TTK	-1.571457366	3.231254926	-5.175543066	3.21495139237307e-06	2.32632932260053e-05	4.07064055
CCDC155	-1.571020239	-3.045065321	-3.72751636	0.000453794	0.001753931	-0.227807862
LINC01126	-1.569808842	-1.287712757	-5.860371942	2.59838566171135e-07	2.63760092918649e-06	6.74866384
ANGPTL3	-1.568942743	-1.632304459	-4.94279221	7.41296177362754e-06	4.840718308589e-05	3.600945505
LOC145845	-1.567515361	-0.833555615	-3.193202855	0.002314866	0.007298288	-1.74215076
BCRP3	-1.565955215	0.18457111	-7.054440918	2.88021726743865e-09	5.62280524595316e-08	11.04336519
LINC01583	-1.565933	-3.33565556	-3.79410568	0.000367105	0.001453955	-0.043483677
ZMAT1	-1.565923926	4.366356068	-8.883227688	2.90375556896553e-12	1.73653780439968e-10	17.64761978
CCDC18	-1.564909681	3.048862255	-9.265897254	7.0357229322656e-13	5.68731695010938e-11	19.11463533
MTBP	-1.563361412	2.425890716	-7.19829558	1.66802434710501e-09	3.56434014284776e-08	11.52404752
SLC17A9	-1.563128169	-1.949047477	-5.199129937	2.95209074031277e-06	2.16156826976973e-05	4.465063747
MPHOSPH10	-1.562056919	5.266165068	-10.84130006	2.40363168107396e-15	5.12380614724418e-13	24.63487369
DUS1L	-1.561547108	4.801555434	-7.239367203	1.42714349795314e-09	3.13580083802122e-08	11.47415572
ZNF648	-1.561338685	-2.616905442	-4.092473691	0.000138958	0.000622555	0.876492058
MST1	-1.561080514	3.69050884	-9.831898338	8.86602556398692e-14	9.92951626699285e-12	21.13418959
MIR217HG	-1.560882301	-0.618766093	-3.705799968	0.000486083	0.001860771	-0.328617165
LOC101928100	-1.559633044	-4.060818818	-4.768579375	1.37407334299227e-05	8.31521993482479e-05	2.997538937
PAX5	-1.558913696	3.40562437	-3.564405552	0.000756689	0.002758076	-1.195166164
NUDT16P1	-1.557998388	-1.836755636	-4.951045972	7.198051909806e-06	4.7130568771836e-05	3.629524671
LOC100505920	-1.557514555	-3.231853509	-3.900758521	0.000260434	0.001081038	0.277649214
RBM43	-1.557063046	-0.904995397	-5.138910886	3.66953261958565e-06	2.61868771805663e-05	4.254028054
LOC392232	-1.556668141	-2.142252467	-4.356625645	5.72339418722683e-05	0.000287289	1.696396609
CASC9	-1.556165988	-0.284459558	-5.033762158	5.35546591925731e-06	3.63256429673411e-05	3.876486062
GPR78	-1.555274373	-3.344808057	-4.40630122	4.8315891119775e-05	0.000248325	1.839762861
LOC101929234	-1.55308743	-0.892301431	-7.011800409	3.38630509293082e-09	6.38446522977463e-08	10.84974351
CASC5	-1.553005499	3.429479043	-4.685611903	1.83855777733739e-05	0.000106857	2.356611137
NAALAD2	-1.552421944	3.574659436	-7.685490772	2.62554196438972e-10	7.52721808510992e-09	13.2586726
ST18	-1.551124394	4.228023545	-4.669783726	1.9431829257586e-05	0.000112075	2.198787885
GOLIM4	-1.55078891	5.260165575	-7.372097238	8.62163829376134e-10	2.03842365848831e-08	11.92915103
MIR6870	-1.550475448	-4.617443091	-5.114452866	4.0076719112643e-06	2.82568129182313e-05	4.143795952
VWA3A	-1.548078042	1.338714374	-5.484086744	1.04474312893635e-06	8.7557689052551e-06	5.342758239
HOTS	-1.546868417	-2.211864641	-5.308291792	1.98641877780878e-06	1.53529261853273e-05	4.837158583
DLGAP5	-1.54627635	3.7649403	-4.506643144	3.42361744283907e-05	0.000183303	1.712351139
LINC00487	-1.54606573	-1.486880581	-5.426739025	1.2891357852189e-06	1.05367118771463e-05	5.2420168
P2RY4	-1.545504838	-2.803891223	-5.092447093	4.33799249359387e-06	3.02868873700916e-05	4.104758134
PHLDB2	-1.543994327	2.867736004	-7.04131372	3.02739400575507e-09	5.85392141581007e-08	10.91121658
HMMR	-1.543008939	2.523454062	-5.066399087	4.76371618864096e-06	3.27914869829028e-05	3.769114762
NTF4	-1.542689721	-1.810790472	-4.270736879	7.65654414810843e-05	0.000370195	1.423174156
LTB	-1.538725414	-2.353124033	-5.508032848	9.56800818298401e-07	8.12235076169685e-06	5.522260818
PPP1R14A	-1.538169265	-0.539961171	-4.548211449	2.96568860442652e-05	0.000161467	2.27472154
C10orf128	-1.537084188	-2.327677533	-3.740734771	0.000435157	0.001689255	-0.176883645
GOLGA6L4	-1.536351751	-1.183456829	-6.115284572	1.00228291271609e-07	1.1655672781269e-06	7.646168717
POLQ	-1.535915021	1.890459005	-4.092608393	0.000138896	0.000622383	0.605089732
SNORA54	-1.535490011	-3.793886139	-4.193609566	9.92231778863743e-05	0.000464289	1.153212246
LRP1-AS	-1.533856775	-3.904376686	-4.489454148	3.632511380126e-05	0.000192906	2.089137372
LINC01280	-1.533672488	-1.99434941	-3.982776996	0.000199398	0.000855213	0.538437726
HEMK1	-1.532571698	4.475914686	-5.920498458	2.07680222974705e-07	2.17141296413094e-06	6.598170787
LOC101928844	-1.532140284	0.149737819	-3.46836049	0.001016897	0.003578703	-1.076009368
MIR155HG	-1.532088776	-2.012229261	-5.774265294	3.57873626686595e-07	3.47526582446979e-06	6.445913565

KPNA7	-1.53202692	-3.615395032	-4.058207963	0.000155626	0.000688132	0.740907223
SMC4	-1.531702404	5.596097724	-5.588517286	7.11511395924081e-07	6.28169029006721e-06	5.268890604
MIR1251	-1.531420652	-3.436179079	-4.579152579	2.66420035771879e-05	0.000147174	2.393760333
LOC339874	-1.530038719	-0.64166197	-8.227443736	3.38529893992892e-11	1.30922396785407e-09	15.2169013
CPSF4L	-1.529989544	-2.827365182	-4.638526155	2.16717365776329e-05	0.000123273	2.600213673
CKAP2L	-1.529309488	3.106400732	-4.970022887	6.7268666811607e-06	4.44195021191908e-05	3.368948225
BRD7P3	-1.528501749	-3.848274155	-3.438909558	0.001112418	0.003868002	-1.087115594
RSPO1	-1.528192005	3.760615337	-3.514790848	0.000881968	0.003154188	-1.391035039
N4BP2L1	-1.527791173	1.218283399	-7.768124047	1.91974029388398e-10	5.6697759986855e-09	13.661771
RBM47	-1.527628203	1.665532733	-3.213234877	0.002183049	0.006939811	-1.953308623
LOC101928307	-1.527469562	3.200906797	-12.27590479	1.76299763771255e-17	7.95163105839964e-15	29.50271493
MEF2C-AS1	-1.526693361	-1.417596942	-5.866391197	2.54079915540947e-07	2.58112775076627e-06	6.769273087
LOC101927854	-1.526613212	-4.6334458	-5.382508524	1.51545190362455e-06	1.2113075345784e-05	5.066585907
PDCL3P4	-1.525274892	-0.575674357	-6.961827899	4.0935531851108e-09	7.47787777070033e-08	10.68571684
NEAT1	-1.524421482	7.46169708	-10.93232906	1.74627929177038e-15	3.91181360333614e-13	24.8675206
LUC7L3	-1.524361502	8.536423676	-16.19566407	9.78704728330621e-23	2.15584184033028e-19	41.51079843
LOC101927762	-1.52397502	-3.546337052	-4.765570224	1.38870643482766e-05	8.3845767911831e-05	3.001742269
ESPNP	-1.523274343	-3.425949204	-4.006101526	0.000184729	0.000799041	0.588803146
CD37	-1.523112244	2.178553492	-4.804981573	1.208583656757e-05	7.4432646316537e-05	2.907878349
DNAH2	-1.522779916	2.422348524	-5.173485231	3.23894872511289e-06	2.34241126267877e-05	4.152666564
TRIM22	-1.521199605	2.374557108	-7.630390676	3.23547169514482e-10	8.97410527993315e-09	13.12946421
MIR1304	-1.519555618	-1.492527892	-4.261086426	7.90982606815698e-05	0.000381325	1.390520695
FMR1-AS1	-1.519033058	-2.481386939	-5.447801027	1.19343471936899e-06	9.85198623893833e-06	5.315014506
INTU	-1.518757774	4.45786882	-9.211992394	8.58400385577613e-13	6.61944718215313e-11	18.84783796
SKINTL	-1.51800735	-2.781519165	-3.867374527	0.00029012	0.001187994	0.193218237
PCDHGB8P	-1.516999216	-1.853653623	-4.329111153	6.28423838159212e-05	0.00031142	1.607610539
ECT2	-1.516598082	4.504021363	-5.927776648	2.02117513834907e-07	2.12006835047543e-06	6.621373608
LINC01089	-1.516273762	5.081257044	-11.48887676	2.5316074027055e-16	7.60431573587663e-14	26.87890896
CLDN10	-1.516138445	-1.787092722	-5.662322553	5.41808499916491e-07	4.94019457685154e-06	6.056569099
FSIP2	-1.51508885	4.560075072	-6.545169852	1.98494411684293e-08	2.91004036830334e-07	8.898594897
BCHE	-1.51430639	3.937862817	-9.005124983	1.84606487809307e-12	1.24165478174641e-10	18.12415421
TTLL6	-1.513595032	-2.106020661	-3.706645249	0.000484786	0.001856882	-0.278355526
OR52N4	-1.513546824	-4.7754634	-5.055883654	4.94698875771635e-06	3.39381660609178e-05	3.938230197
SEMA3B-AS1	-1.510252642	-0.63605121	-6.090191483	1.10114165717275e-07	1.2638505177615e-06	7.563595515
LOC101927055	-1.509274824	-0.741711716	-5.405924036	1.3911490785128e-06	1.12556607261491e-05	5.164862661
MIR3136	-1.508881663	-3.835535102	-4.071390611	0.000148996	0.000661808	0.77261606
ALOX15	-1.508514646	-0.646429261	-3.438979121	0.001112183	0.003867692	-1.088823427
CFAP74	-1.507765728	-0.935203558	-3.735570435	0.000442349	0.001714209	-0.219248161
MIR631	-1.507132523	-1.850770607	-6.152263751	8.72449745511379e-08	1.0327570140216e-06	7.772949099
ADAMTS6	-1.506895537	2.482117084	-4.968275592	6.76895336803598e-06	4.46416527887463e-05	3.433579553
MIR3124	-1.506784049	-3.256041716	-4.50733996	3.41540102373611e-05	0.000183011	2.166133312
LTBR	-1.505257001	-3.484460609	-3.56920744	0.000745508	0.002720699	-0.702026472
KC6	-1.504968919	3.020613317	-9.228589973	8.07384273661896e-13	6.3516632457455e-11	18.98003049
NOSTRIN	-1.503345965	-0.951223696	-3.403845704	0.001237258	0.004249539	-1.162576075
LOC101927577	-1.503107993	-1.541549498	-4.009083594	0.00018293	0.000792336	0.613900913
LOC101927391	-1.502985344	2.264376529	-10.9039698	1.92883252828821e-15	4.25612583447843e-13	24.86437223
ERN2	-1.501921151	-1.621842075	-4.596721127	2.50655739494169e-05	0.00013978	2.462503802
YY2	-1.501877085	0.639796075	-6.921215626	4.77563526114211e-09	8.50063076483295e-08	10.56152396
PLCZ1	-1.501359787	-4.724102667	-4.506686387	3.42310698471492e-05	0.000183303	2.110123851
PRAMEF2	-1.500970487	-4.779007757	-5.063507057	4.81344485174066e-06	3.31165506939253e-05	3.964070671
KIF20B	-1.500575617	3.955637153	-6.633006972	1.42377576009955e-08	2.18552059620856e-07	9.290010317
ROBO4	-1.496080263	-1.607003493	-4.853266491	1.01888904489181e-05	6.39418189069927e-05	3.303722468
ATG16L2	-1.495596789	2.915721822	-9.366786527	4.85236637099456e-13	4.13750323498384e-11	19.48190097
SPAG8	-1.495483065	1.813006417	-8.468689024	1.36682731065071e-11	6.27245595528304e-10	16.22730652
AGER	-1.493956004	3.506683787	-7.9162575	1.09582943866781e-10	3.46898916794086e-09	14.1242286
LOC101927018	-1.493370229	-1.557850504	-5.969836271	1.7274856671576e-07	1.84815917749733e-06	7.131710405
KCNN4	-1.49334327	-1.22843653	-4.931461444	7.71827942117702e-06	5.01147825939504e-05	3.561529635
SLC28A2	-1.493193108	-1.555109098	-3.37709959	0.001341298	0.004555381	-1.216234644
SDCBP2	-1.49292698	-3.245205138	-5.040682746	5.22423607111392e-06	3.55907814607614e-05	3.925314653
SPN	-1.4921241	-2.23580271	-4.588632094	2.57797522619289e-05	0.000143279	2.440849808
PCP2	-1.491316336	-0.390145193	-6.983241566	3.77402228486874e-09	7.02031886389412e-08	10.77007969
POU2F2	-1.491046597	6.048957379	-6.063731389	1.2158877209265e-07	1.37289876664887e-06	6.970318207
MIR3153	-1.490378416	-3.516387221	-3.844333286	0.00031248	0.001266257	0.098336543

CENPC	-1.489422039	3.649631557	-9.770110875	1.10979553072041e-13	1.16409624061637e-11	20.91392047
HMGN5	-1.489266875	3.426325281	-9.886540873	7.27173308789192e-14	8.46756478908577e-12	21.33714309
ITGA2B	-1.489227635	2.150199429	-6.547657818	1.96636034309605e-08	2.88760016383655e-07	9.134041595
FAM231B	-1.48905149	-4.167524227	-4.629360235	2.23751101251673e-05	0.000126674	2.534974342
GALNT12	-1.486399779	1.948955617	-5.725462761	4.28887365995181e-07	4.0502964434979e-06	6.154504943
MIR296	-1.486318891	-3.658391613	-3.80333197	0.000356428	0.001418681	-0.029377548
ZRANB2-AS1	-1.486313486	1.622482633	-10.44741633	9.68777390753034e-15	1.63106323374363e-12	23.24784567
CNTRL	-1.486188146	4.800010471	-8.304157233	2.53617060891678e-11	1.0507617195219e-09	15.46449419
XRCC2	-1.48480875	1.801599996	-4.918496496	8.08278934673627e-06	5.22122118285141e-05	3.333265125
HRH4	-1.483484935	-3.564934921	-4.333198561	6.19766604327128e-05	0.000307938	1.600704794
LENG8	-1.483369747	7.605332298	-12.20052155	2.26776614002519e-17	9.82686268512884e-15	29.20178972
C400927-CSNK	-1.48333971	1.226697156	-11.21671243	6.47836494638341e-16	1.69547149136389e-13	25.79640711
HMGA2	-1.482922787	3.990294555	-3.513646883	0.000885077	0.003164085	-1.427722341
PLEKHH2	-1.482286102	2.004328096	-5.904347538	2.20572575618109e-07	2.2873263598719e-06	6.794555565
PWRN2	-1.480930416	-3.446490146	-3.596646008	0.000684581	0.002522027	-0.621969577
DNAH11	-1.48078439	0.829937311	-3.193603912	0.002312155	0.007290611	-1.905438483
TPR	-1.479853274	7.066070017	-7.579099516	3.93021377792416e-10	1.04409387730522e-08	12.59248379
ANG	-1.479666867	-0.148670085	-6.941218261	4.42657408206846e-09	7.99232463875106e-08	10.62574423
EDRF1-AS1	-1.478606082	-0.938292021	-6.640515933	1.38387843896317e-08	2.13170505694135e-07	9.518784036
ROBO3	-1.47853265	7.421280327	-5.072040516	4.66816085280704e-06	3.22346905552627e-05	3.30708769
ZBBX	-1.478418483	2.079064767	-6.743026297	9.38609560149648e-09	1.52023691810267e-07	9.858733884
CHKB	-1.478334488	-2.566956664	-4.016436475	0.000178567	0.000776455	0.645965336
ZNF192P1	-1.477714917	2.423701105	-7.506336239	5.17970170601569e-10	1.32669627127047e-08	12.66689345
DOC2GP	-1.477613573	-0.632923247	-3.311093053	0.001634645	0.005403096	-1.442463623
RTCA-AS1	-1.476820528	0.745620549	-9.339697946	5.36087923469021e-13	4.55640259841049e-11	19.30263253
LOC101928205	-1.476248557	-2.077121325	-3.810596987	0.000348231	0.001390451	0.02600413
UNC5CL	-1.47565035	1.611077175	-6.642199098	1.37508934136473e-08	2.12187603971359e-07	9.513108821
LRRC19	-1.474116177	-2.053724989	-3.983769584	0.000198751	0.000852994	0.543108181
CAPN12	-1.473934453	-1.108902192	-4.851200439	1.02637204736276e-05	6.43350541331273e-05	3.292821157
LINC01001	-1.472768517	-0.271739836	-5.030871828	5.41122726646314e-06	3.66588126424293e-05	3.866006581
NKTR	-1.47246608	7.223692006	-8.945665109	2.30214907511996e-12	1.48060113145124e-10	17.70447726
CCDC114	-1.471794971	0.365265563	-4.073798947	0.000147815	0.000657335	0.702101399
UGT8	-1.471624002	0.770392131	-6.687349254	1.15900984582186e-08	1.8312078454638e-07	9.706891085
LOC100506497	-1.471396419	-3.382769875	-3.830530365	0.000326659	0.001317136	0.062655273
MTR	-1.470444196	5.469601639	-9.406129559	4.19899426146235e-13	3.6631028156183e-11	19.48679432
TMC4	-1.469628744	0.70118043	-6.866344363	5.88080447676098e-09	1.01599545577924e-07	10.36085861
STAM-AS1	-1.469324515	0.259147311	-10.564749	6.3835561265326e-15	1.16370026960439e-12	23.44766215
LOC101927636	-1.46836204	-4.080444922	-4.574780954	2.70490694637561e-05	0.00014908	2.359771788
XIRP2	-1.467430687	-4.214067297	-3.308823721	0.001645736	0.005433643	-1.466797653
CFAP70	-1.467087662	2.312422535	-8.333606542	2.27024207262837e-11	9.66333473523119e-10	15.72795609
ARR3	-1.467074316	-0.37030031	-5.914144544	2.12660496641398e-07	2.21570946303589e-06	6.938665442
CSNK1A1P1	-1.463465752	-2.583935703	-3.803108397	0.000356683	0.001419483	0.008032986
RANBP3L	-1.463271832	-1.24024602	-3.526202589	0.000851514	0.003057747	-0.806236088
NRG4	-1.463140389	1.631721848	-10.54835001	6.76613326058066e-15	1.22499452381458e-12	23.59526024
CGB7	-1.463081731	-2.430027966	-4.037504815	0.000166616	0.000731222	0.710739422
PRAMEF14	-1.462005497	-4.891491723	-5.719939114	4.37756275409079e-07	4.11933486218875e-06	6.242258557
MIR708	-1.461372301	-4.160514171	-4.632319723	2.21456012275442e-05	0.000125509	2.544955211
MYL7	-1.460989968	-2.354278991	-4.823646176	1.13147231102544e-05	7.0190583424866e-05	3.210228168
HPN	-1.460884273	-2.154072454	-3.488749892	0.000955389	0.003385229	-0.897902736
CAPN11	-1.460199407	-3.668521359	-4.299241651	6.95354243741034e-05	0.000340061	1.489800604
SLC35A3	-1.459586474	3.846598242	-7.121679467	2.23127353682831e-09	4.55803736631801e-08	11.12527618
USP45	-1.459251872	3.63563792	-10.51432069	7.63551092545046e-15	1.35456013619082e-12	23.5524663
PARD3-AS1	-1.459104289	-1.473970174	-9.23546252	7.87164113822134e-13	6.24423963869286e-11	18.65186855
CNGA4	-1.458176866	-0.605238358	-4.76707177	1.38138564922054e-05	8.34991238642727e-05	2.996040927
HCG23	-1.457901701	-2.136156218	-3.685395536	0.000518417	0.00197	-0.338412407
VTRNA2-1	-1.457260573	-3.156446506	-3.326710385	0.001560208	0.005188196	-1.362729169
C6orf25	-1.456944055	1.843310378	-6.411514475	3.28841402189281e-08	4.49212650339497e-07	8.653681776
MIR30C2	-1.456519542	-3.340812759	-3.335856849	0.001518113	0.005063506	-1.34616398
PLBD1	-1.455598993	-1.970741704	-4.075370585	0.000147049	0.000654369	0.821035437
FAM13A-AS1	-1.454430637	1.514164634	-8.132249808	4.84651209721153e-11	1.76214380007692e-09	14.99662753
DSG2	-1.454423079	3.21606417	-4.236321827	8.59756387054033e-05	0.000409772	0.898620776
LOC101929577	-1.453646671	-2.193783328	-5.381156364	1.52295557203083e-06	1.21656949638836e-05	5.086402392
WDR11	-1.45283849	5.478593971	-8.080908868	5.88248470455001e-11	2.05405175951612e-09	14.57268359

UPK3B	-1.452739755	-2.180098167	-5.216084716	2.7763323038855e-06	2.04819402145145e-05	4.523640123
RAD51AP1	-1.452473815	3.018043311	-5.395164989	1.44696198026283e-06	1.16430885918683e-05	4.869612504
CDK1	-1.451456956	4.643686313	-4.747376052	1.48048793055521e-05	8.87788962530984e-05	2.407763231
C1QTNF8	-1.450084311	-3.080259917	-4.318172139	6.52175126610044e-05	0.000321622	1.56756646
KCNJ2-AS1	-1.447594476	-0.068824727	-4.900322966	8.62232082837844e-06	5.52384407311021e-05	3.415128404
ZNF518A	-1.447504749	5.30885171	-8.849741098	3.28921992629718e-12	1.93410841009764e-10	17.45078755
SNORD10	-1.446999431	-0.555926602	-6.484289653	2.49842944777295e-08	3.54783253315764e-07	8.970884814
SPACA6P-AS	-1.446575672	-1.032957225	-7.222026017	1.52428596288758e-09	3.30528719089478e-08	11.59422499
UPK3A	-1.446333043	-3.963833423	-3.193759702	0.002311103	0.007288162	-1.761303212
ZNF663P	-1.446247061	-0.167688032	-8.057902131	6.41623453116724e-11	2.21699774329861e-09	14.65569943
MIR3186	-1.44422773	-1.693819576	-4.905035391	8.47911835900891e-06	5.44927147054905e-05	3.476525259
PDIA2	-1.44405165	3.888377971	-11.34465843	4.16034616075691e-16	1.16989819220519e-13	26.4174702
LINC00475	-1.443461853	-3.662710681	-3.770114393	0.000396327	0.001556397	-0.127599517
KLK2	-1.442867679	-4.518313094	-4.756519626	1.43364394488292e-05	8.62045277413337e-05	2.941129218
TNFSF10	-1.442335952	-3.338868147	-4.198723892	9.7538289779508e-05	0.000457539	1.185335082
ANLN	-1.442116973	3.472275463	-5.097158681	4.26508785490593e-06	2.98488396263512e-05	3.765598151
COQ3	-1.441896443	2.64723791	-6.953457654	4.22567491048904e-09	7.67151544704374e-08	10.60017266
FREM1	-1.440109248	2.997835956	-4.735609087	1.54298177984938e-05	9.19423746320075e-05	2.578319387
PDE6B	-1.439867102	2.687288656	-7.452565466	6.35226549699515e-10	1.57809618310219e-08	12.45252156
AIRE	-1.439792196	-4.046299819	-4.765676858	1.38818528886609e-05	8.38334515435171e-05	2.988503499
CCDC150	-1.43972922	1.394318333	-6.21190293	6.97365869437316e-08	8.51998638733497e-07	7.951396685
CPT1B	-1.438563327	-0.66594495	-5.815360541	3.07204751221117e-07	3.04817687275818e-06	6.591948386
KCTD4	-1.438541888	-0.289652616	-4.091611008	0.000139356	0.000623808	0.808767221
EGFEM1P	-1.438508753	2.581824282	-6.847559511	6.31508498785313e-09	1.07764132655857e-07	10.21189485
MBNL3	-1.438017532	2.806806638	-4.08976382	0.000140211	0.000627317	0.483560881
LOC401320	-1.437101478	3.75100972	-15.94491538	2.00644202169157e-22	3.53575213062489e-19	40.65825303
ZNF730	-1.436546752	2.182436704	-8.271858021	2.86395452080192e-11	1.15753684783421e-09	15.5037036
SNAI1	-1.436176161	0.883197661	-4.549942984	2.94797088020944e-05	0.000160568	2.181096828
PCK1	-1.435515183	-3.477484369	-3.822641181	0.000335038	0.001347341	0.035543816
NDST4	-1.434945375	1.906738455	-5.645681076	5.76179702941363e-07	5.22475406101168e-06	5.871693432
CPZ	-1.434319663	-0.734075267	-3.606591486	0.000663695	0.002454658	-0.607707738
DSC2	-1.434155185	2.028522182	-6.517248582	2.20589378011684e-08	3.18799290813715e-07	9.02984716
KIF18B	-1.433826416	2.938000772	-3.926247887	0.000239759	0.001006441	-0.044375536
FANCM	-1.432720293	2.748928937	-8.442448148	1.50827321452112e-11	6.8384538386684e-10	16.11538162
LOC101928371	-1.431936289	-2.502239848	-3.955066391	0.000218288	0.00092602	0.461683502
YPEL4	-1.431933154	3.56322541	-10.09429951	3.43232785626799e-14	4.49142189231346e-12	22.07308393
TPBG	-1.431659762	5.965771124	-4.990961599	6.24208277144263e-06	4.15504844869159e-05	3.109457862
STK26	-1.430368874	3.87928047	-4.722513793	1.61556761832507e-05	9.547126951752e-05	2.421326758
DUOX2	-1.430303629	-1.569744423	-3.850074023	0.000306759	0.001246896	0.137777915
LTK	-1.4300374	-1.875905464	-5.61446508	6.46573278160353e-07	5.77199306369896e-06	5.890266669
KIF23	-1.429971377	3.592227595	-4.756252074	1.434993813363e-05	8.62464562724516e-05	2.573738318
AURKAPS1	-1.429617605	0.624102467	-6.215835268	6.87134743445269e-08	8.42441218621929e-07	7.998737413
PPEF1-AS1	-1.429249461	-4.514609206	-4.643034713	2.13337336401254e-05	0.000121612	2.566089044
IL11RA	-1.426331006	4.470149959	-11.91131466	5.99842969583397e-17	2.11408656199972e-14	28.32330982
CAPS2	-1.425451876	1.948882922	-6.467780754	2.65917277133471e-08	3.75480309106251e-07	8.853165889
LOC101928323	-1.425345942	0.210537285	-6.059430334	1.23563003900798e-07	1.39221691479531e-06	7.447116206
PRAMEF1	-1.425095568	-4.787861969	-4.910629663	8.31214535228202e-06	5.35498264920474e-05	3.44558795
GSDMD	-1.424559618	-2.541834598	-3.427256341	0.001152518	0.003988544	-1.06456694
INPP5D	-1.424508149	2.798086325	-4.250558953	8.19539997579119e-05	0.000393376	0.997748013
SBK3	-1.424051772	-3.80000415	-4.094560146	0.000138001	0.000619212	0.845603644
SLC26A5	-1.422970712	-1.51101504	-3.778542568	0.000385816	0.001519409	-0.073833071
CA4	-1.422549139	-0.704761592	-3.71471984	0.000472563	0.001815588	-0.296609797
KRTAP19-1	-1.422402062	-4.861598079	-5.143197947	3.6132261511746e-06	2.5813082933513e-05	4.233295945
LINC00954	-1.42173125	-2.742052309	-4.071318358	0.000149032	0.000661855	0.809761745
IQCF1	-1.420910427	-2.492997403	-3.392335435	0.001281058	0.004380622	-1.159934917
DPY19L4	-1.419289075	4.295480495	-6.939892009	4.44890822486725e-09	8.02716662852669e-08	10.39918865
COL2A1	-1.418747417	3.917973304	-3.890847291	0.000268928	0.00111193	-0.288544456
PPIEL	-1.417648063	2.897062878	-7.950714655	9.61960248514017e-11	3.13146493213929e-09	14.29118398
SPINK13	-1.416921767	-2.125322837	-4.530363924	3.15447348158799e-05	0.000170725	2.252851421
GTF3C3	-1.416609738	5.222058612	-6.557842478	1.89207655886992e-08	2.78780711709078e-07	8.874643633
FAAHP1	-1.416330952	-2.486342034	-4.206202922	9.51241991017974e-05	0.000447804	1.229969669
LIPH	-1.4149522	-2.107210709	-4.22840942	8.82927942645804e-05	0.000419367	1.295891431
ACTN3	-1.413868935	-1.519324966	-3.885203252	0.000273883	0.001129768	0.242306368

C1orf167	-1.412958285	-4.046094112	-4.127837846	0.000123571	0.000562861	0.938090965
RSRP1	-1.41235462	4.683384602	-17.54580942	2.3180786685646e-24	1.22547546892336e-20	45.12904054
RUFY4	-1.41117275	-3.396538772	-3.404475542	0.001234903	0.004242552	-1.160237428
LOC102723927	-1.410636472	0.018532067	-7.317664492	1.06009788731871e-09	2.41982447802205e-08	11.9943077
PRAM1	-1.410600745	0.366875235	-5.722193983	4.34114075358136e-07	4.09330236858282e-06	6.240660539
GABPB1-AS1	-1.409693449	4.855747987	-10.84785905	2.34885155651336e-15	5.04773928400957e-13	24.67639389
C6orf201	-1.409395332	0.145865123	-6.385635277	3.62562086476162e-08	4.87715197548315e-07	8.62048032
PWRN1	-1.409237239	-1.117296896	-3.567366431	0.000749776	0.002734387	-0.69347956
COL11A2	-1.408724457	4.209223139	-12.44298492	1.01163675549442e-17	5.24325379568314e-15	30.08277515
CYP4F30P	-1.408425829	-4.039594735	-4.57504311	2.70244885570243e-05	0.000149007	2.362143429
PNPLA7	-1.40705732	1.409499928	-8.392548086	1.8191109683847e-11	8.05436519720482e-10	15.94522197
CEP44	-1.406277856	4.327995869	-11.16739254	7.68850132928743e-16	1.95413611189476e-13	25.80320872
CAPN10-AS1	-1.406123543	2.298113686	-14.4399279	1.74603952257013e-20	2.42663300875053e-17	36.07425315
PRR25	-1.40558903	-0.74943894	-6.857632579	6.07837252000201e-09	1.04602617722144e-07	10.30415873
SNORD54	-1.405301218	-1.028026337	-5.399011109	1.42676078851972e-06	1.15050542778956e-05	5.145166351
RNPC3	-1.405271764	4.283292239	-17.0518905	8.90376628518221e-24	3.36218934594602e-20	43.76666987
WDR64	-1.404319434	-3.411593572	-3.94039355	0.000228977	0.000967635	0.390420421
TBC1D32	-1.404311156	3.723550119	-8.848864259	3.29997689612997e-12	1.93410841009764e-10	17.56295333
SNHG22	-1.403892938	-2.517335892	-4.720505131	1.62699303746575e-05	9.60414648843946e-05	2.871821365
SPATA1	-1.403635527	-2.139016654	-4.663220323	1.98825503889453e-05	0.000114326	2.683279187
EN2	-1.403569673	5.429126792	-3.778361252	0.000386039	0.001520061	-0.828027487
GDF7	-1.403428159	0.343703988	-3.304206669	0.001668522	0.005498615	-1.552528505
IFI44	-1.402758824	1.860379082	-4.436135692	4.36262229525647e-05	0.000227137	1.710327608
NTN5	-1.402750931	0.307671237	-5.671006581	5.24686438467845e-07	4.81062664863702e-06	6.061787194
UTRN	-1.402678493	4.973502771	-5.900150075	2.24051152363648e-07	2.31885047393434e-06	6.46465555
PTPN7	-1.402307388	-3.714665308	-4.143260749	0.000117389	0.000537063	0.999711141
MFSD7	-1.401137053	-1.57427527	-5.562850624	7.82082325955402e-07	6.83397756098484e-06	5.711706925
IL21R-AS1	-1.400421131	-1.103443767	-5.155503307	3.4562877842903e-06	2.47991463089428e-05	4.314148068
IMPG1	-1.399769689	-0.327124324	-6.739425603	9.51503705366583e-09	1.53735314449602e-07	9.892828136
HYMAI	-1.399441931	-0.137794162	-10.35673135	1.33880708765601e-14	2.05748184581461e-12	22.67176037
CCDC168	-1.399014338	-2.277233679	-3.535073236	0.000828536	0.002988225	-0.76432006
MIR34A	-1.398164437	-1.05291321	-4.367318754	5.51883035867867e-05	0.000278555	1.718402841
GPR32	-1.397441099	-4.430116974	-3.635930996	0.000605564	0.002262202	-0.560001439
SGK2	-1.397225995	0.74480682	-5.186257167	3.09280981460639e-06	2.25274846595455e-05	4.342187554
PIGL	-1.396971857	3.293419802	-17.4568476	2.94811573336751e-24	1.29879238633506e-20	44.63743748
CCDC88B	-1.396960537	2.788595168	-9.840956978	8.57920886222974e-14	9.71722860043622e-12	21.18111864
FLJ34503	-1.396275722	-2.798156677	-3.817518727	0.000340589	0.001365923	0.045145399
FAM83H-AS1	-1.396115726	-0.004086556	-5.398699434	1.42838740236519e-06	1.15111476239997e-05	5.118856488
LOC100507006	-1.395000854	1.37798163	-6.986668027	3.72525784975011e-09	6.958992278618e-08	10.78824547
LOC100505984	-1.3945815	-4.046367326	-4.465085805	3.95008235230065e-05	0.00020791	2.005512197
OLIG3	-1.394558538	3.053139231	-3.759415132	0.000410067	0.001601786	-0.56942433
LINC00939	-1.393274419	-4.688641546	-3.740483015	0.000435505	0.001690164	-0.270773883
LOC101927418	-1.393252096	-2.010052944	-5.326628681	1.85811644656834e-06	1.45216685063204e-05	4.899852193
MYLK-AS2	-1.392946691	-2.342284898	-4.257338841	8.01036013620228e-05	0.000385673	1.389152468
KRTCAP3	-1.392518184	0.853013154	-8.008655578	7.72782065040533e-11	2.61214172956732e-09	14.53394925
ETAA1	-1.392368114	3.9899235	-9.784344914	1.05381182453786e-13	1.13233365682964e-11	20.95020752
CD207	-1.392202708	-3.802819093	-3.931160385	0.000235961	0.000993651	0.346706475
GRIA2	-1.391996592	7.038740975	-11.66262998	1.3963400275716e-16	4.64121569295057e-14	27.40113295
ARHGAP11A	-1.39166269	3.750985139	-5.307110549	1.9949770580912e-06	1.54145655002995e-05	4.469565359
LOC101929294	-1.391337977	-3.986219572	-3.800546482	0.00035962	0.001429018	-0.0527405
LINC00664	-1.387555732	-0.036599508	-5.773239398	3.59239140416116e-07	3.48724502336364e-06	6.434156753
FIRRE	-1.387360947	1.349956436	-3.593155746	0.000692059	0.002545672	-0.847876058
GPHA2	-1.385409442	-4.031237554	-4.406074933	4.83532713658982e-05	0.000248469	1.816456815
RHBDL1	-1.384844175	2.80553525	-8.301910667	2.55769779796768e-11	1.05664214940073e-09	15.59516334
ZNF90	-1.38439989	1.581109827	-5.008366391	5.86537435545968e-06	3.94201475560299e-05	3.661391947
CCDC183-AS1	-1.384206227	1.933186178	-11.99152864	4.57514640695877e-17	1.65664171198824e-14	28.44997988
LINC00342	-1.383300656	4.234605424	-9.315775532	5.85443161314083e-13	4.86635820220602e-11	19.240179
CCDC17	-1.381260783	0.205820142	-6.184700038	7.72411572342739e-08	9.28886749095658e-07	7.896450473
NUSAP1	-1.381223609	5.242010411	-5.083292247	4.48317148130372e-06	3.11406560722758e-05	3.498653182
GPR20	-1.381138638	-4.026195952	-3.356694334	0.00142619	0.004798439	-1.324356217
TNRC18P1	-1.3802923	-1.819284441	-4.576639178	2.68753034801312e-05	0.000148308	2.400273436
PLD4	-1.379251397	-3.302671443	-3.624416151	0.000627774	0.002335531	-0.536077342
ANKRD20A12P	-1.378929367	-2.002997553	-3.237476664	0.002032977	0.006523939	-1.583873491

LOC388849	-1.378896175	0.514440246	-6.978847158	3.83749662832513e-09	7.09842885769897e-08	10.771831
KLHL14	-1.377162511	3.219559688	-7.123407102	2.21668426461153e-09	4.53510953300901e-08	11.18823253
INTS6-AS1	-1.375437021	0.834001681	-8.496850826	1.22981237099335e-11	5.6776812038452e-10	16.30238415
TRIM53AP	-1.375433384	-4.995652088	-5.167940883	3.304485000246e-06	2.38393700904756e-05	4.313265475
PRAP1	-1.374227327	-1.561421565	-3.441375686	0.001104104	0.003842123	-1.03613381
PGM5-AS1	-1.374142729	-2.547607546	-3.160402325	0.002546986	0.007926122	-1.782798575
LINC01556	-1.374054328	-3.998582228	-4.147898737	0.000115589	0.000529894	1.002546979
CFAP58	-1.37398051	-1.052048156	-5.670010442	5.26622980937281e-07	4.82336287426028e-06	6.083868394
TH2LCRR	-1.373867448	-2.965339382	-4.121393731	0.000126246	0.000573047	0.957202406
ADIRF	-1.373723029	-1.708950841	-4.49285093	3.59026943886794e-05	0.00019091	2.129687842
SNORD115-13	-1.373112086	-1.856879015	-4.310843135	6.68573780715094e-05	0.000328789	1.552597458
ACSL5	-1.373027847	0.026189669	-7.128354238	2.17543275497886e-09	4.46453524940653e-08	11.30820503
LOC101927078	-1.372341407	-3.10552997	-4.582659519	2.63197820764034e-05	0.000145699	2.413235078
TEPP	-1.372316986	-0.422994647	-5.999354083	1.54706648442796e-07	1.68209444010837e-06	7.241191911
EFCAB13	-1.371979916	1.509465044	-6.855733697	6.12230768091364e-09	1.05221689811177e-07	10.30202317
HFM1	-1.371729144	3.10193075	-8.236754442	3.2686401086383e-11	1.27621807964012e-09	15.34001433
CD164L2	-1.370383429	-3.689557606	-3.624193742	0.00062821	0.002336826	-0.553748719
CEP162	-1.370103298	3.396836736	-8.273224332	2.84926320036537e-11	1.15513150575549e-09	15.45777712
ZBTB20-AS4	-1.369300534	-2.662217173	-4.795810396	1.24833734378427e-05	7.65066102672142e-05	3.117789102
ZNF732	-1.369085867	0.616972834	-5.607628476	6.63090915446975e-07	5.89753774159149e-06	5.823810368
LOC101928973	-1.368908275	-3.86296032	-4.397927882	4.97179880523151e-05	0.000254196	1.796708819
LOC101928020	-1.368002965	-0.811239536	-5.415650917	1.34252278937763e-06	1.09190476589596e-05	5.199548591
AVPR2	-1.367256666	-2.812688637	-3.894568077	0.000265708	0.001100669	0.273553967
SNORD3D	-1.366735512	-4.027111519	-4.025051213	0.000173584	0.000758402	0.622122117
JAK3	-1.365963222	2.272458747	-5.306854235	1.99683888513844e-06	1.54197420273957e-05	4.634481078
MIR124-1	-1.365855267	-3.28860653	-3.712749654	0.000475517	0.00182641	-0.279900648
HHIPL2	-1.365759397	-2.571646053	-4.039288486	0.00016564	0.000727314	0.716085397
TMEM191B	-1.36484373	1.468137105	-4.474354983	3.82621931159682e-05	0.000202037	1.876529494
RBP3	-1.364537529	-2.031872275	-3.893568236	0.00026657	0.001103644	0.274103316
COL28A1	-1.363190655	0.99888987	-6.865586725	5.89772882283952e-09	1.0175892034864e-07	10.35353862
ACKR4	-1.362963769	-1.377989117	-4.034960123	0.000168017	0.000736516	0.693193269
C19orf67	-1.362810787	-2.534941185	-3.806137283	0.000353241	0.001407479	0.018582117
SNORD22	-1.362236848	0.59831582	-8.904646513	2.68136688783225e-12	1.64066136449236e-10	17.74819484
COL11A1	-1.361661142	5.693341958	-5.552686168	8.11904791890698e-07	7.05724411839751e-06	5.127402144
NKAPP1	-1.361623312	-0.742527635	-5.572263777	7.55431374366942e-07	6.62518829417431e-06	5.741570016
PLCG1-AS1	-1.360974443	0.372110502	-11.10860536	9.43334172264139e-16	2.26683201595073e-13	25.27645283
MYO3A	-1.360484055	1.048895787	-4.700758349	1.74360721848644e-05	0.000101966	2.665870157
ALOX15P1	-1.360152761	-1.280793014	-3.394670775	0.001272054	0.004352649	-1.169735415
DPEP1	-1.35941433	-2.586224219	-3.878151198	0.000280198	0.00115276	0.230901526
GOLGA6L17P	-1.35938284	-2.161144272	-5.042787439	5.1849569060862e-06	3.53778951725804e-05	3.939400204
SGOL2	-1.35680665	3.668266696	-6.471684993	2.6202520718232e-08	3.70975484812547e-07	8.719412128
MAFTRR	-1.356234078	-3.346536389	-3.841809328	0.000315028	0.001275407	0.097749742
ARPC4-TTLL3	-1.355943037	-1.704175691	-5.919320054	2.08595049707196e-07	2.17850373327155e-06	6.953032148
LINC01515	-1.355469146	-0.163270976	-5.258698057	2.37882354344801e-06	1.79552948954772e-05	4.640304437
HPR	-1.354878307	-3.706037205	-4.524382623	3.2203261480743e-05	0.000173933	2.20875644
GUSBP2	-1.353640475	0.967369228	-10.54053625	6.956483230541e-15	1.24243730562764e-12	23.48860608
C1QTNF9	-1.353594809	-3.740234117	-4.193203369	9.93582001694009e-05	0.000464757	1.154261901
TMEM106A	-1.353105581	-0.885380032	-4.692868658	1.79245172542241e-05	0.00010443	2.762911404
LINC00472	-1.353028916	3.15682303	-6.722691245	1.01378732955764e-08	1.62605828168672e-07	9.701538153
NKG7	-1.352933957	-1.09401849	-5.495432864	1.00212139143439e-06	8.44407865469727e-06	5.478184719
RHBG	-1.352871018	-2.788708903	-3.169624902	0.002479601	0.00774652	-1.766740717
MEGF6	-1.352837922	5.181240938	-7.612174253	3.46686976228431e-10	9.47067745151315e-09	12.83539948
A2MP1	-1.352610664	-3.739307164	-3.733018401	0.000445945	0.001725868	-0.239932432
NRIR	-1.352298272	-4.061107779	-3.873954945	0.000284022	0.001166854	0.162542868
SNORD58C	-1.351944452	-0.542360047	-6.662537286	1.27318608931059e-08	1.99182822400816e-07	9.610840223
F11	-1.351745433	-4.210341277	-4.014831959	0.000179511	0.000779916	0.582031632
FBXO24	-1.351409762	-0.348986249	-6.101066655	1.05716064675345e-07	1.22292898799273e-06	7.602684098
NSUN6	-1.35108061	4.276595225	-15.3714027	1.06556774134167e-21	1.56478622816024e-18	39.09164561
PCDHGA12	-1.349493407	-1.302091028	-3.869474827	0.00028816	0.001181288	0.193351601
CRYBG3	-1.348571573	4.191073896	-5.22940181	2.64554756302117e-06	1.96266513425031e-05	4.138166664
GOLGA6L7P	-1.348251202	-2.405248025	-3.986821947	0.000196776	0.000845614	0.558949229
KRT12	-1.347974144	-3.6974759	-3.399915074	0.001252053	0.004291989	-1.187277888
KIF11	-1.347739303	4.500175615	-4.402231075	4.89925528836565e-05	0.000251022	1.264996989

UFL1	-1.347310873	5.221516029	-8.287455371	2.70066993015609e-11	1.11021474749325e-09	15.36444752
VWA8	-1.347131415	3.683281785	-5.559388309	7.92117074595137e-07	6.9056829263764e-06	5.378380267
LRIG3	-1.346479828	1.85911311	-3.894117849	0.000266096	0.001101944	-0.009423165
SH3D21	-1.346044727	3.820361215	-5.177046213	3.19753323352807e-06	2.31689681912959e-05	4.000659773
CD4	-1.345490176	0.48699	-3.526811293	0.000849918	0.00305352	-0.944251382
FBLN2	-1.34545013	3.68994472	-6.731638687	9.79996214155631e-09	1.57952682492535e-07	9.683504517
EXPH5	-1.345323345	-0.809114258	-3.093386042	0.003090793	0.009396082	-2.007011724
MIR124-2	-1.344820976	2.012808681	-4.008308126	0.000183396	0.000794057	0.325011941
LRRTM2	-1.344344153	5.092656582	-9.149143038	1.08280535819043e-12	8.01730925295449e-11	18.56935432
WNT5A	-1.344205314	4.60000648	-7.161332012	1.91938255327346e-09	4.00203882011852e-08	11.19682876
TIPARP-AS1	-1.343822639	-1.546877762	-3.764764049	0.000403142	0.001578936	-0.111654986
FAM72B	-1.343688221	1.176180551	-4.831261069	1.10141574291398e-05	6.85189981935637e-05	3.0933812
ANP32E	-1.343354355	6.510264066	-8.323378953	2.35926454252918e-11	9.98307829481174e-10	15.41603498
SNORD27	-1.343326921	-1.961828014	-4.571864268	2.73240283021057e-05	0.00015022	2.38643766
CTC-338M12.4	-1.343162493	2.076858832	-16.46666019	4.54199608888373e-23	1.33398425130515e-19	41.65953181
ACSM4	-1.342688881	-2.479729598	-3.570368826	0.000742828	0.00271204	-0.66206753
USP32P2	-1.342620655	-0.387072496	-3.8228378	0.000334827	0.001346696	-0.001186929
ARG1	-1.342077552	-2.191678808	-4.244980707	8.35074824991507e-05	0.000399593	1.349623711
AGBL3	-1.341998736	1.667445099	-16.02824572	1.57929534306578e-22	2.98182241451842e-19	40.33443416
MIR124-2HG	-1.341996526	6.561042561	-7.941728818	9.95202870469035e-11	3.21244766286317e-09	13.98122321
PREX2	-1.341647133	3.317458802	-5.145165787	3.58766602124417e-06	2.56512783174323e-05	3.951436874
SCGB2B3P	-1.341176751	-4.428292222	-4.035369478	0.000167791	0.000735647	0.633690625
PAUPAR	-1.340845033	-1.875623604	-3.849726418	0.000307103	0.0012481	0.142212008
ZIC5	-1.340008012	4.540963469	-4.868856608	9.64122293839899e-06	6.08952081076943e-05	2.834653074
LOC100507547	-1.339864108	0.649956933	-8.342069548	2.19913478958629e-11	9.42135006371709e-10	15.73398175
MIR3657	-1.339003987	-3.146499284	-3.370357796	0.001368802	0.004635093	-1.242949909
LOC101927267	-1.338188313	0.366791562	-3.803043697	0.000356757	0.001419563	-0.124197366
PTPN13	-1.337403376	6.012196243	-6.124107946	9.69659754714479e-08	1.13311301044951e-06	7.193725087
LY6G6C	-1.3371316	-0.215547648	-4.656120675	2.03816044561731e-05	0.000116916	2.609771659
ADCY4	-1.335702185	-2.576908985	-3.515354144	0.000880441	0.003150066	-0.81892478
CSNK1G2-AS1	-1.334882788	-2.329895826	-4.250173605	8.20604072452124e-05	0.000393738	1.36768848
MIR133A1HG	-1.334733942	-2.745891146	-3.224141209	0.002114285	0.006745552	-1.620921267
POU6F2	-1.333802987	1.181153072	-3.182673767	0.002387117	0.007493903	-1.977937294
GRIN3B	-1.333183268	1.482629005	-8.542728268	1.03551416699655e-11	4.9407483711588e-10	16.4928299
POF1B	-1.332888526	-3.121007218	-4.198827711	9.7504376238659e-05	0.000457461	1.192483352
LOC100506801	-1.332637052	-1.449312519	-5.96424052	1.76397100939464e-07	1.87950366566914e-06	7.111407168
CRABP2	-1.332372336	5.654026253	-6.121155847	9.80455548533907e-08	1.14370615685776e-06	7.21030743
FOXP4-AS1	-1.331885954	-1.536144156	-4.720182452	1.628835811534e-05	9.61264054616618e-05	2.866535002
SNORD53	-1.331546029	-4.443926242	-3.6232712	0.000630024	0.002342255	-0.597413554
DDX11L5	-1.33083702	-3.244544588	-3.195705408	0.002297998	0.007255493	-1.718158757
CEP55	-1.33043887	2.495998222	-4.435984277	4.36488656517173e-05	0.000227165	1.636856863
C5orf34	-1.330398463	2.597551796	-9.422514832	3.95372568449732e-13	3.47205418665507e-11	19.68513341
DLEU2L	-1.329132952	0.721310832	-7.945565341	9.80871173010516e-11	3.18518030911388e-09	14.30034016
SNORD98	-1.328875759	-2.362658537	-4.120239619	0.000126731	0.000575087	0.965523604
MPZ	-1.328010728	1.474728863	-4.744942151	1.4932076244857e-05	8.9379431924888e-05	2.774717746
SNORD51	-1.32798299	-3.287259496	-4.575758375	2.69575327459017e-05	0.000148669	2.386689434
FCN3	-1.327667142	-3.792322356	-3.150958664	0.002617765	0.008119619	-1.864357247
EREG	-1.327521092	-4.043789753	-3.393416531	0.001276882	0.004368038	-1.223984904
MXD3	-1.32621685	3.921491723	-5.710730879	4.52946687300241e-07	4.24565240617279e-06	5.895747342
MLPH	-1.32517457	-2.050419728	-3.460258784	0.001042367	0.003653744	-0.975160852
CASC18	-1.325125837	-3.679883939	-4.193883811	9.91321179401415e-05	0.000463945	1.158666961
ARHGAP11B	-1.323650951	0.443191242	-4.74705689	1.48214982550223e-05	8.88382456632665e-05	2.869039982
LOC101060019	-1.323405614	-4.165050201	-3.692501092	0.00050693	0.001931904	-0.379935571
LOC101926964	-1.323317972	0.811776366	-4.40995084	4.77168830896929e-05	0.00024558	1.729178858
TMEM213	-1.322244138	-1.89718792	-3.315530267	0.001613161	0.005340235	-1.374832768
C20orf144	-1.322241722	-0.367489877	-8.284897182	2.72679546577669e-11	1.11697382566194e-09	15.45021625
MUSTN1	-1.3217887	-0.138947278	-4.71951394	1.63266009748423e-05	9.63305900821441e-05	2.814814693
MIR6840	-1.321325166	-2.759264295	-3.689506382	0.000511741	0.001947712	-0.327835295
GK5	-1.320115949	5.403771801	-9.459733445	3.44885877393085e-13	3.07985418822008e-11	19.68485963
ZNF107	-1.319164835	3.241303767	-6.098135574	1.06883968632626e-07	1.23427869937361e-06	7.38602815
EID3	-1.318936263	-0.899568289	-3.095130309	0.003075356	0.009351303	-1.99483609
MAPK13	-1.318625638	3.179480201	-6.769334107	8.49543065943726e-09	1.39564772293912e-07	9.872664032
CFAP44-AS1	-1.318393351	-4.549396906	-4.149445955	0.000114995	0.000527535	0.980510498

HIF3A	-1.318097831	4.22271587	-4.680555778	1.87136555605294e-05	0.000108454	2.231583047
TDRP	-1.318078179	5.118516992	-13.2689401	6.84582313245776e-19	4.89069305027719e-16	32.74269001
DOCK4-AS1	-1.317451291	-3.055514941	-3.306140066	0.001658945	0.005471102	-1.413947409
CDHR5	-1.317181535	1.725722453	-3.595154079	0.000687768	0.002532003	-0.887727455
COL20A1	-1.31718014	1.157059534	-5.054803296	4.96620638501384e-06	3.40591788935869e-05	3.857488302
NLRP6	-1.316573541	-4.28274265	-4.039283749	0.000165643	0.000727314	0.653406276
TROAP	-1.315819705	3.296453464	-4.040336466	0.000165069	0.000725038	0.262540699
LOC101559451	-1.315620762	-2.331623506	-4.598779718	2.4886928222965e-05	0.000138931	2.476628922
MIR3685	-1.315315163	0.616339417	-8.337793988	2.234774550786e-11	9.54310108254061e-10	15.71679332
ITPR1-AS1	-1.314950339	-0.652459173	-6.519995168	2.18311458036458e-08	3.16678216419996e-07	9.096780398
MIR6884	-1.314385341	-3.446767633	-4.199467226	9.72957226229956e-05	0.000456968	1.18430804
FUT6	-1.313383046	-0.63766401	-3.61993173	0.000636632	0.002364493	-0.576945982
LINC01424	-1.313052313	-1.211784359	-5.111622288	4.04873214574854e-06	2.85007022126688e-05	4.167189536
FLJ31104	-1.312034211	-0.321507993	-6.87197086	5.75662568451104e-09	1.00240373332464e-07	10.37104574
BORA	-1.311963207	2.973658266	-6.98139575	3.80055513879261e-09	7.04488597361186e-08	10.67874332
SNHG21	-1.311878301	2.263063645	-16.39139421	5.61667620766565e-23	1.48465602197226e-19	41.52579617
LOC102723854	-1.311213773	-3.943207613	-3.472390228	0.00100445	0.003540083	-0.998162037
ASMTL-AS1	-1.311070773	2.342407996	-12.42758918	1.0646025825449e-17	5.37372715942327e-15	29.91367587
LOC100507639	-1.310879973	-4.304371454	-4.126893295	0.000123959	0.000564119	0.923066513
MIR548L	-1.310682276	-4.431394336	-4.082247062	0.000143743	0.00064095	0.778094736
NEB	-1.310312164	1.880236564	-6.19153437	7.52834406894818e-08	9.09075919481531e-07	7.845977858
LOC202181	-1.31016999	2.953372202	-9.6268442	1.87074694524737e-13	1.81799463248985e-11	20.41632308
TMEM191C	-1.309960486	1.542029811	-11.06756296	1.08839808246933e-15	2.59185824449656e-13	25.34717388
MFNG	-1.309625441	1.836514988	-4.914445854	8.20009847723128e-06	5.28977849861933e-05	3.314238037
LHX5-AS1	-1.309569169	3.701329584	-3.922265581	0.000242882	0.001017608	-0.162164506
NSUN5P2	-1.309071028	2.183746259	-14.3748946	2.13097511127321e-20	2.56036659619476e-17	35.85910961
H19	-1.308760261	-4.871208863	-5.367929288	1.59831880478292e-06	1.26605816502328e-05	5.008772751
COL18A1-AS1	-1.306556133	-4.252674322	-3.723494116	0.000459615	0.0017728	-0.294063409
CREB3L3	-1.306358663	-4.276766804	-3.557685336	0.000772605	0.002806029	-0.77563795
LINC01590	-1.306334769	0.828844406	-7.160335979	1.92665585723646e-09	4.01318315794573e-08	11.43465334
UVSSA	-1.305958266	4.828272884	-8.255325035	3.04787267208913e-11	1.21698517131921e-09	15.27685741
GPR63	-1.305808975	2.945928308	-6.918968247	4.81653256312905e-09	8.56765849536945e-08	10.44866196
ADH6	-1.305723017	-0.728375197	-6.237084465	6.34376661213826e-08	7.87622277400896e-07	8.085010953
DNHD1	-1.305555619	4.444775112	-4.670640604	1.93737281858099e-05	0.000111862	2.168791054
SNORA38	-1.305416063	-4.103050881	-3.722270739	0.0004614	0.001778729	-0.289448627
RLN2	-1.303896584	-1.567753723	-4.017524335	0.000177931	0.00077409	0.643597547
CCDC78	-1.301686378	3.140708133	-10.42964199	1.03211567816935e-14	1.70511960756565e-12	23.25894473
TRIM45	-1.301106442	4.041437507	-7.38445602	8.22645362987487e-10	1.9537264282759e-08	12.08852067
LOC101929516	-1.300540862	-2.29712299	-3.687895717	0.000514347	0.001956221	-0.325048398
RPS6KA2-AS1	-1.299706525	-3.908811048	-3.456155045	0.001055498	0.003693894	-1.04179512
CENPK	-1.299601833	2.891892311	-5.636857244	5.95270810601345e-07	5.37023663366053e-06	5.746135592
CABP4	-1.299213152	-1.747805366	-4.443800736	4.24948183652037e-05	0.000221726	1.973970048
LOC101927623	-1.298420147	-2.535292252	-4.551363664	2.9335111195549e-05	0.000159813	2.322911394
DCST1	-1.298304385	-0.691153981	-6.50609887	2.30082275649227e-08	3.30530695230218e-07	9.046102597
AWAT1	-1.297997536	-4.401866942	-3.725517716	0.000456677	0.001763014	-0.296740641
TTC26	-1.297824957	3.21543838	-5.745168792	3.98672410001317e-07	3.81400934258589e-06	6.102312644
TCIRG1	-1.296854593	0.853365066	-6.794329798	7.72744969433453e-09	1.28142834234846e-07	10.09563523
LINC01285	-1.29674171	-2.577540144	-3.917888961	0.000246359	0.001030384	0.349943977
MTMR11	-1.296707752	1.978707269	-6.442671136	2.92361600042109e-08	4.06378792597405e-07	8.758285065
DIAPH3	-1.296282149	1.515946637	-4.043208866	0.000163515	0.000719205	0.490193364
CYYR1	-1.295822633	1.23165094	-3.38819264	0.001297179	0.004426586	-1.42043864
DCST2	-1.295459544	0.962758676	-8.80778434	3.84568450597466e-12	2.21466184197011e-10	17.42871759
CMA1	-1.295093455	-3.251701461	-4.366336448	5.53732167748587e-05	0.000279382	1.715475286
DPRX	-1.294749039	-4.706353098	-5.026602547	5.49463720881897e-06	3.71742373536503e-05	3.84147903
RPGR	-1.293873207	3.465259901	-6.255648149	5.91594811496388e-08	7.44294414673204e-07	7.941446246
MEIS1-AS3	-1.292003112	1.874211718	-3.668768385	0.000546278	0.002061351	-0.689889956
SAR1B	-1.290282833	4.850514937	-7.354478963	9.21806939926822e-10	2.14490517984909e-08	11.8966036
ZNF600	-1.289387997	-0.175086191	-6.844263316	6.3945248776649e-09	1.08979030361906e-07	10.27471114
SLC34A2	-1.289366186	-1.677306659	-3.555841363	0.000777028	0.002819768	-0.711441041
RFPL4B	-1.288351933	-3.053483657	-3.196243577	0.002294386	0.007245819	-1.707962738
MKI67	-1.288259738	6.021539659	-3.71650793	0.000469896	0.001807445	-1.072305107
MIR100HG	-1.287998283	7.245994892	-8.511703887	1.1631963580554e-11	5.4515548461841e-10	16.08808198
LOC100128568	-1.2872376	-0.493448401	-5.732485821	4.17867479420328e-07	3.96464145137026e-06	6.298939635

LOC653160	-1.287087739	0.214462046	-4.974171203	6.62797369923733e-06	4.38540916142193e-05	3.648524293
ACADL	-1.286297998	-1.329937434	-3.387208474	0.001301037	0.004438032	-1.187591815
TMPRSS6	-1.286069089	0.338922525	-5.976744444	1.68347449403224e-07	1.8089138740144e-06	7.147773558
RPS14P3	-1.286060966	-0.318022678	-5.390914288	1.4696166910888e-06	1.1793071644065e-05	5.102520758
CL2L2-PABPN	-1.285755551	-2.902100192	-4.141588571	0.000118044	0.000539559	1.021379645
C20orf203	-1.284949125	2.127671626	-8.108349257	5.30380369314043e-11	1.88942645580567e-09	14.90323032
MBL1P	-1.284927874	-2.143778617	-4.563829712	2.80956395387213e-05	0.000153854	2.362530273
LOC400997	-1.284730574	-3.060124659	-3.959234453	0.00021534	0.000915127	0.459768119
PAPLN	-1.284157017	1.560530409	-3.978040759	0.000202511	0.000866596	0.282808639
STX19	-1.283734127	-3.503262589	-3.196955939	0.002289613	0.007235067	-1.727328819
RNU6-26P	-1.283526978	-2.305817429	-3.661253481	0.000559335	0.002104612	-0.401424265
HES7	-1.283408057	1.599705809	-4.806186027	1.20345581781302e-05	7.41341124032895e-05	2.96903056
MAMDC4	-1.283159899	4.537482364	-11.4263008	3.13952094120444e-16	9.11944582844581e-14	26.68307303
PUS10	-1.282744837	2.116705445	-10.04731199	4.06583434002848e-14	5.19189367681028e-12	21.89388638
FAM111A	-1.281041164	2.591220548	-6.193368671	7.47664475983203e-08	9.03658669120439e-07	7.796682776
SRPK3	-1.279713841	0.41229466	-6.007475176	1.50079716397461e-07	1.64131449877289e-06	7.255536955
PDCD4	-1.279686206	5.556993773	-9.228952362	8.06305122733087e-13	6.3516632457455e-11	18.82963136
ARGLU1	-1.279024626	7.520323206	-14.20657909	3.57750559105344e-20	3.94017522034648e-17	35.6406171
LINC-PINT	-1.278982545	2.410378757	-7.651992963	2.98103128554063e-10	8.36492568691034e-09	13.20700207
LOC101927901	-1.277555664	-3.679869555	-3.872909604	0.000284983	0.001170258	0.177123782
LOC644285	-1.277201761	1.318335543	-6.038240246	1.33763987991837e-07	1.48624779091561e-06	7.325245243
MIRLET7DHG	-1.276819412	0.812286337	-8.364677553	2.01995542439032e-11	8.76740258340053e-10	15.82433243
ALDH1L1-AS1	-1.276431316	-3.392380424	-3.914933069	0.000248734	0.001039327	0.314695168
C4orf47	-1.276262848	1.457534464	-4.353464889	5.78525988780034e-05	0.000290009	1.482705205
P2RX2	-1.275230299	-1.533642024	-3.135496983	0.002737649	0.008441936	-1.857451954
C11orf65	-1.274534796	-0.44866732	-5.296618037	2.0726101999185e-06	1.59213325819371e-05	4.780689354
COL9A2	-1.274266876	3.411186383	-4.482092276	3.72573219987074e-05	0.000197241	1.674538806
FMN1	-1.274261574	0.277290378	-3.927590493	0.000238715	0.001003014	0.259463892
NDC80	-1.27403563	3.302910748	-4.588333764	2.58064681502222e-05	0.000143391	2.0426299
CALML6	-1.273827249	-3.303182717	-3.510012661	0.000895024	0.003194893	-0.861354606
MUC4	-1.27252675	-0.668851534	-4.925918409	7.87210414966185e-06	5.09758277775629e-05	3.527841391
NEIL1	-1.272259102	2.936072704	-6.300769371	4.99197760584751e-08	6.42106783724414e-07	8.160230195
GOLGA6L9	-1.271764503	4.749441674	-8.738947151	4.97135015593801e-12	2.69831003433079e-10	17.08138796
HERC2P2	-1.271674719	6.436585326	-12.41833001	1.09779921540823e-17	5.37372715942327e-15	29.95357725
C17orf75	-1.27141839	4.877423031	-6.239682363	6.28209324381418e-08	7.81065713611195e-07	7.723876809
L3MBTL1	-1.270763079	4.32276826	-10.46793746	9.00510989219721e-15	1.53569077277709e-12	23.36752986
MIR135A2	-1.270349116	2.464245455	-4.234202145	8.65905389968215e-05	0.000412331	0.98464663
LOC729737	-1.270068086	2.549223766	-5.899012444	2.25003279203998e-07	2.32779322082164e-06	6.726798372
MACC1	-1.269840893	-2.615104509	-3.352871468	0.00144265	0.004847192	-1.269482173
LOC101928068	-1.269720876	1.401729992	-10.40427464	1.12982166694486e-14	1.78829797139841e-12	23.07923564
GOLGA6L3	-1.268422681	1.399894294	-9.333412022	5.4863572579673e-13	4.60383750475713e-11	19.33334912
PLCH1	-1.268228477	4.961419454	-6.180641761	7.84275224496581e-08	9.39743744746968e-07	7.495973907
TARBP1	-1.26784564	5.39527545	-12.89097094	2.32233532940621e-18	1.46157832767129e-15	31.52599899
TSSC2	-1.266202419	2.998188187	-5.296635974	2.07247497319586e-06	1.59213325819371e-05	4.521189671
ACRBP	-1.265932888	0.798680255	-6.960432795	4.11528440051813e-09	7.51238346401213e-08	10.70352429
SLC6A16	-1.26586553	2.13169021	-4.820275828	1.14503057986685e-05	7.09484137778258e-05	2.962409331
PLA2G4B	-1.263905418	1.139354583	-7.810380116	1.63586084898939e-10	4.89148301146342e-09	13.81613774
FAM157C	-1.263265397	-2.745202212	-3.363674414	0.001396594	0.004715311	-1.24466678
REST	-1.262554388	4.213278869	-5.439092806	1.23212108294535e-06	1.01302197777587e-05	4.88003865
PEX3	-1.261186174	3.835410657	-9.15902517	1.04395378895957e-12	7.83944048396824e-11	18.69181502
DLEC1	-1.26070353	0.117186119	-4.296364776	7.02154676975367e-05	0.000342816	1.42166567
CDC25C	-1.260607083	1.941274652	-4.206773753	9.49423437224776e-05	0.000447107	0.958084407
PCBP2-OT1	-1.257671921	-0.900330212	-3.868277977	0.000289276	0.001185308	0.170199438
PBK	-1.257414521	3.236925556	-3.604764684	0.000667485	0.002467641	-1.05662945
LRRCC1	-1.256723872	3.862992226	-8.689516887	5.97913206584768e-12	3.15461872049005e-10	16.96609652
ZNF334	-1.256469433	4.895157649	-9.008372062	1.82395618356086e-12	1.23305968798118e-10	18.06495871
LOC101929613	-1.256342075	-4.058941578	-3.916210793	0.000247704	0.001035353	0.289892829
FBXO43	-1.255604618	-0.99094781	-5.818542594	3.03591958920857e-07	3.01799407677887e-06	6.603185399
ADGRE2	-1.254890027	-3.793186424	-3.798791599	0.000361645	0.001435338	-0.048511291
MIR186	-1.254862852	0.764050347	-7.389344796	8.07518069157116e-10	1.92645533592329e-08	12.27227101
WDR72	-1.254814451	-0.048867456	-4.32897931	6.2870503158297e-05	0.000311501	1.53778722
LRP2BP	-1.254792598	2.707639014	-7.882085158	1.24704558490763e-10	3.86891501712011e-09	14.04577129
TNFSF13B	-1.254012598	-1.378318959	-4.855736608	1.01001274962006e-05	6.3475194985038e-05	3.3127312

ERVW-1	-1.252896442	0.629849465	-5.390992808	1.46919504648362e-06	1.1793071644065e-05	5.060927021
LOC100507462	-1.251651683	-1.227551612	-5.561820255	7.85055411072961e-07	6.85089788078295e-06	5.708163946
SMIM5	-1.251329859	-1.065950854	-4.053239003	0.000158198	0.000698453	0.73938077
NEK7	-1.249909512	3.802648344	-6.530840583	2.09543333792945e-08	3.05339522720447e-07	8.923510796
PCDHGA5	-1.249831263	-2.092964437	-4.388662776	5.13155644488377e-05	0.000261353	1.80225589
PLK4	-1.249408337	3.574560634	-5.286955038	2.14673649166359e-06	1.64286872276038e-05	4.418523489
PRORSD1P	-1.246500256	-1.11969307	-4.827693084	1.11540013947202e-05	6.92585667997741e-05	3.215457809
CD2AP	-1.246350852	3.81605481	-7.258095942	1.32917069814229e-09	2.93813173293957e-08	11.63654545
FAM227B	-1.244560104	1.556567098	-6.586475058	1.69788597772373e-08	2.53417391581995e-07	9.310158916
TAS2R43	-1.242991132	-3.588113401	-3.388386698	0.00129642	0.004424566	-1.213476251
PIWIL2	-1.242663481	0.629241511	-3.74901019	0.000423866	0.001650566	-0.311447769
MIR7856	-1.242493546	-4.105292003	-3.819393609	0.000338548	0.001358971	-0.00258487
GLYCTK-AS1	-1.242270976	2.943809606	-3.266892178	0.001863926	0.00604307	-1.979657505
SNORD11	-1.242251314	-2.854961671	-3.299978876	0.001689649	0.005559876	-1.422086421
GUCY2EP	-1.241921457	-3.989656214	-3.31243336	0.001628127	0.005384246	-1.44312747
MIR5087	-1.241278099	-3.730139098	-3.641510566	0.000595075	0.002226413	-0.505548549
MIR1915	-1.241213309	-3.454944653	-3.375336909	0.001348438	0.004576103	-1.242961806
MIR769	-1.24098674	-4.263510842	-4.14769667	0.000115667	0.000530067	0.989888431
LOC100147773	-1.240558106	-0.400652964	-9.31654839	5.83779276595007e-13	4.86635820220602e-11	19.07593093
KIF12	-1.240210813	-0.024337708	-3.703096111	0.000490254	0.001875651	-0.385176263
MUC5B	-1.240123868	-0.900976376	-4.901885846	8.57456659693303e-06	5.49724760748801e-05	3.455451439
ZNF680	-1.239550616	3.773268021	-7.154964356	1.96635852378542e-09	4.08622286629088e-08	11.25483161
LAMA5-AS1	-1.239448043	-4.701464174	-4.105430368	0.000133117	0.000600354	0.834841126
LOC100507468	-1.238283927	-1.231879589	-6.292564602	5.14859247076004e-08	6.59044768908475e-07	8.271372304
FANCB	-1.237871444	0.719889152	-5.374269076	1.56174857448975e-06	1.24305028815078e-05	4.9969564
VN1R1	-1.237749607	1.463497505	-7.19795827	1.6701622842164e-09	3.56602582057286e-08	11.56301568
PHACTR2	-1.237540943	3.078781722	-5.076206167	4.598814581697e-06	3.18221114759154e-05	3.738063119
MIR1289-1	-1.237418353	-3.552163545	-3.804852129	0.000354698	0.001412857	-0.019938301
IKZF1	-1.237343036	1.419596289	-5.386597456	1.49298209168022e-06	1.19515431948465e-05	4.990488353
ZNF100	-1.236690833	3.359997008	-5.767521385	3.66945169329711e-07	3.54512487605711e-06	6.166746391
LINC01158	-1.236054414	5.076887083	-4.8478597	1.03858596007182e-05	6.50081522201714e-05	2.695232765
SASS6	-1.235430982	3.322851939	-9.846040875	8.42235794602399e-14	9.63758387823602e-12	21.19443158
HP09053	-1.235011894	-1.547179846	-4.297515176	6.99427616279255e-05	0.0003418	1.509973381
MIR7111	-1.234668885	-3.301172399	-3.886932114	0.000272356	0.001123994	0.234184004
FBXW12	-1.234391106	-1.130085505	-6.40998644	3.30742706941423e-08	4.5157654817059e-07	8.690692573
TDRD6	-1.234346646	0.265549532	-8.157954448	4.39875589870246e-11	1.61939156922566e-09	15.04704321
LINC01343	-1.234154589	-2.162155562	-4.151640137	0.000114157	0.000523782	1.061711168
STARD9	-1.233886531	5.549550648	-5.517616184	9.23692368190517e-07	7.8761162478645e-06	5.011354469
LOC100134868	-1.233749117	0.011435853	-5.542148973	8.4400772973651e-07	7.30266982655488e-06	5.618911241
CCDC171	-1.23262257	2.629117889	-8.52854384	1.09204318787194e-11	5.16386003309821e-10	16.4352907
SLC13A4	-1.232243966	2.763580295	-4.303234589	6.86021552899315e-05	0.000336119	1.170404925
CCND2	-1.232183077	7.127995029	-4.652204957	2.06620687866152e-05	0.000118242	1.86880334
SACS-AS1	-1.232110046	-0.569575619	-6.575318401	1.7710706410185e-08	2.6256147085834e-07	9.297113547
MIR17HG	-1.231842215	2.080684391	-5.244209036	2.50724762704996e-06	1.87532757571623e-05	4.432034915
NODAL	-1.231727098	0.994256136	-5.306074427	2.00251383533392e-06	1.5454729404199e-05	4.740337086
LIPI	-1.230989385	-1.239388759	-5.289726885	2.12520994652585e-06	1.62733703697908e-05	4.772571222
SEC14L4	-1.230724978	-2.945474122	-3.35310485	0.00144164	0.004844503	-1.281529774
TMEM123	-1.229775878	5.976294605	-5.831392507	2.89426033785886e-07	2.88938679018062e-06	6.117344493
RBBP6	-1.229643139	6.652008015	-9.450457533	3.56826314166492e-13	3.17575419608178e-11	19.58494981
AKAP9	-1.229277625	7.233469771	-7.32354531	1.036688689317e-09	2.38533864616573e-08	11.62025482
ZNF138	-1.229170916	4.816154487	-10.40547732	1.12498518936922e-14	1.78829797139841e-12	23.12194297
CRACR2B	-1.229063459	2.206301084	-7.419437286	7.2034375065212e-10	1.75491671529838e-08	12.35421832
CDCA2	-1.228988197	2.478181485	-3.896526254	0.000264029	0.001094585	-0.078824453
SNORA25	-1.228734034	-0.523035209	-5.133545918	3.74121408645759e-06	2.66123552064945e-05	4.224457202
STON1	-1.228705634	3.101255818	-4.51917758	3.27872205921526e-05	0.000176766	1.837168834
TTC32	-1.228682449	3.151194463	-13.38648833	4.69961516762336e-19	3.65367434487612e-16	33.03648315
DPPA5	-1.228499477	-3.918961601	-3.302076322	0.001679136	0.005530104	-1.467232457
ZMYND15	-1.228137745	-2.192247779	-3.682389405	0.000523351	0.001986466	-0.340826789
TCTE3	-1.228121963	0.17770873	-8.320477076	2.38515556203722e-11	1.00553137115359e-09	15.62537081
NCKAP1L	-1.227989923	-1.54552141	-3.976049076	0.000203834	0.000871261	0.519016894
LPAR4	-1.227971793	2.133894339	-3.551655886	0.000787157	0.002852999	-1.06605332
MIR3671	-1.227312133	-1.713226401	-4.278718035	7.45303821510236e-05	0.000361445	1.452673042
LY75	-1.226816272	-3.641584256	-3.240528061	0.002014791	0.006474225	-1.618193202

LOC10192968(	-1.226583581	-4.085477022	-3.665108452	0.0005526	0.002082829	-0.455215431
OVOL1	-1.226360735	-1.305184799	-3.236066971	0.002041431	0.006549477	-1.596805349
LINC00260	-1.226134545	0.393154093	-3.102341643	0.003012296	0.009185975	-2.100352294
MIR4640	-1.226073115	-1.91197017	-4.10194246	0.000134665	0.00060682	0.906125432
LINC00304	-1.225990419	-0.290072295	-3.901734913	0.000259611	0.001078131	0.227842825
KEL	-1.225891481	1.196019343	-4.146060353	0.000116299	0.00053278	0.846563304
MIR7851	-1.225865276	-2.556388422	-4.415584465	4.68064086884692e-05	0.000241647	1.888244181
CATSPERG	-1.225597818	3.308520533	-7.253768827	1.35119141221491e-09	2.98130572613329e-08	11.66620547
LOC10192835{	-1.223751851	-0.797571927	-4.433193798	4.40682087170386e-05	0.000229032	1.916070174
CCNA2	-1.222903433	4.433443808	-4.434642339	4.38500398405862e-05	0.000228032	1.378402569
ZCCHC11	-1.222785216	6.266616446	-9.803898079	9.81516818565099e-14	1.08101808604714e-11	20.88856095
CASC15	-1.221779964	2.750199956	-5.7533759	3.86719436982181e-07	3.71309657746095e-06	6.179856733
SDHAF3	-1.221695884	3.201951364	-10.3496181	1.37326914948075e-14	2.09824412879911e-12	22.97867879
LOC10012877(	-1.221665955	-0.952853229	-5.125350709	3.85337844809782e-06	2.73219829717193e-05	4.208841787
KLK1	-1.220776543	-4.822031279	-4.031570424	0.000169902	0.000743791	0.598932108
LCA5	-1.219842387	3.871306274	-8.827331153	3.57554103732707e-12	2.07263763683479e-10	17.47319205
PRR15L	-1.21953553	-3.676108764	-3.437959378	0.001115638	0.003877666	-1.080359107
MRPL16	-1.219429248	3.946540299	-7.323499673	1.03686834806659e-09	2.38533864616573e-08	11.86854374
ZSWIM2	-1.219197322	-1.716948247	-5.874701598	2.4633672486735e-07	2.51600411453581e-06	6.795913379
BUB1	-1.218920471	3.905359901	-4.264680863	7.81455163670503e-05	0.000377145	0.8937127
SCML1	-1.216854522	4.271148562	-8.078463499	5.93703263328546e-11	2.07036389967856e-09	14.66396087
WDR36	-1.216482682	4.939419175	-7.39770806	7.82282355148134e-10	1.87471164946787e-08	12.04911776
IQUB	-1.215915778	0.442841535	-6.033029437	1.36397980374218e-07	1.51106781862183e-06	7.346068891
TYMSOS	-1.213505648	0.707216196	-5.734353867	4.14983844241533e-07	3.94152639412018e-06	6.268188081
LINC01004	-1.213300095	2.914779998	-5.536949339	8.60308272777205e-07	7.4170021442661e-06	5.384652405
MIR7641-2	-1.213046921	-2.377207761	-3.52260578	0.000861003	0.003087626	-0.794199192
TMEM88B	-1.212876193	-0.655350049	-4.782513333	1.3082593092726e-05	7.94970536137991e-05	3.049075564
LOC10192974	-1.212284339	-0.876701777	-4.714456879	1.66187547395272e-05	9.78577732301006e-05	2.833312505
ZNF483	-1.212213204	2.882807039	-5.085025801	4.45531857513327e-06	3.09832770051296e-05	3.791279607
LOC10013305(	-1.212193094	-1.424341611	-6.44791174	2.86634428245909e-08	3.9982099429151e-07	8.818838351
ITGB1BP2	-1.212132136	0.114320475	-5.246709477	2.48460714677668e-06	1.86208167594976e-05	4.586773902
KIF20A	-1.212039411	3.612917263	-3.850227939	0.000306607	0.001246662	-0.372788993
STAG3L5P	-1.210192949	-0.992872988	-5.858761785	2.61400824876451e-07	2.64938957207026e-06	6.744311671
PCDHB18P	-1.210052572	-0.418459017	-4.84009739	1.06751818031807e-05	6.66140416910945e-05	3.230208653
FAM138B	-1.209978905	-4.053248792	-3.200354466	0.002266969	0.007172089	-1.74873845
TNFRSF10C	-1.209838529	-0.995820544	-3.769858026	0.000396651	0.001557207	-0.115439343
TIGD4	-1.209609965	1.557597329	-8.633561466	7.37021730216359e-12	3.6967163937019e-10	16.82434632
CES4A	-1.209157522	3.247827882	-8.376193946	1.93439573957314e-11	8.47958251809895e-10	15.84723357
ABCA1	-1.208991042	5.043103676	-4.050623256	0.000159568	0.000703563	0.056629012
OFD1	-1.208487465	5.01410697	-11.66088689	1.40467315641829e-16	4.64121569295057e-14	27.46388774
MVP	-1.208366903	4.109803514	-5.172340511	3.25237407808672e-06	2.35019147091488e-05	3.945336941
ZNF121	-1.208346683	3.694025997	-5.73738567	4.10345647238772e-07	3.90167859477067e-06	6.018416988
LENG8-AS1	-1.208315827	2.51809954	-8.752556114	4.72517824974821e-12	2.60809743357979e-10	17.25815943
MTFR2	-1.208203341	0.139123972	-4.811639104	1.1805053747659e-05	7.29242780350244e-05	3.105031612
LENEP	-1.207834569	-3.160713524	-3.194451963	0.002306432	0.007277775	-1.717417766
LIN28B	-1.207242834	3.629157063	-7.084874865	2.56592301204728e-09	5.10700929237935e-08	11.00650137
COL9A1	-1.207185285	3.00288446	-4.436131982	4.36267776912225e-05	0.000227137	1.574701599
UBR5-AS1	-1.206726199	-0.487423334	-7.167621415	1.87408536247176e-09	3.92844555005678e-08	11.43108566
HJURP	-1.206106565	3.5605716	-3.767878909	0.000399161	0.0015652	-0.61611898
ZNF217	-1.205478066	4.7254567	-5.043208075	5.17714159987372e-06	3.53519462437257e-05	3.414041124
ATP2A1	-1.205160285	1.62129502	-5.951483804	1.85003049148476e-07	1.95842434847483e-06	6.991761694
IQGAP3	-1.204913936	3.618705622	-4.058342081	0.000155557	0.000687942	0.273610122
ANKRD36	-1.204832393	5.145903952	-7.612069563	3.46824642419125e-10	9.47067745151315e-09	12.83571287
ZNF662	-1.204832178	0.045436917	-3.790272514	0.000371631	0.001470338	-0.133597162
KNTC1	-1.204501654	4.683410553	-6.185962167	7.68758453489739e-08	9.2534572864728e-07	7.545121996
SPESP1	-1.204216222	-3.674253441	-3.418800885	0.001182468	0.004079674	-1.133533867
BUB1B	-1.204123855	4.179787623	-4.220836889	9.05670771289609e-05	0.000428104	0.713582229
LOC10050591:	-1.203552805	-0.522347942	-5.265183972	2.32345702009963e-06	1.76027341393791e-05	4.674715082
CHD1	-1.202865297	5.966455247	-7.071759093	2.69693404915438e-09	5.32795648141239e-08	10.73806023
ZNF80	-1.202438776	-2.258921952	-4.286070823	7.27020916302548e-05	0.00035365	1.481183425
TENM1	-1.202393949	5.823453048	-5.293422599	2.09683922802405e-06	1.60700931616004e-05	4.184180942
FAM72A	-1.202339907	0.142124955	-4.343502502	5.98453874139145e-05	0.000298978	1.570155511
CXCR6	-1.201845303	-4.47641965	-3.443365633	0.001097437	0.003823457	-1.111471442

RNY5	-1.20173969	-3.876904775	-3.082062078	0.003192792	0.009668354	-2.048947508
JMJD1C	-1.201642196	6.45425412	-7.785995725	1.79410001732313e-10	5.33447083890913e-09	13.39955887
RGS5	-1.201046502	1.869025625	-6.44409037	2.90799440617425e-08	4.04776282982644e-07	8.770022841
ADAMTS9-AS2	-1.200596657	-0.387009584	-4.495795413	3.55403999343129e-05	0.000189365	2.095735806
SNORD15A	-1.200587292	-3.323136421	-3.228248891	0.002088919	0.006674291	-1.635132069
PI4KAP1	-1.200402862	4.765277448	-4.89626717	8.74746732085735e-06	5.5931737709778e-05	2.898533075
DEPDC1B	-1.19943981	3.325988249	-4.789733718	1.2753801173471e-05	7.79471043741873e-05	2.719729107
RHOH	-1.199253743	-0.399369799	-3.481336982	0.000977331	0.003456024	-0.99110042
RBM41	-1.198051481	4.843949961	-8.096478129	5.54678807054248e-11	1.95752001426768e-09	14.68011761
KIAA1551	-1.197776677	4.824212851	-7.863840797	1.33617418469971e-10	4.10687118885669e-09	13.81028172
SLC16A8	-1.1972737	0.933614265	-9.040167254	1.62105720740109e-12	1.12171217704799e-10	18.25849644
GOLGA8T	-1.197137869	-1.45534944	-5.095695004	4.28760601689591e-06	2.99826163610078e-05	4.115761488
TET1	-1.196714445	5.334395314	-8.043818248	6.76668917740938e-11	2.31691541465367e-09	14.44103238
ZNF382	-1.195921722	3.935884272	-10.12820679	3.03788662734702e-14	4.03519885530973e-12	22.18078478
MNS1	-1.195433678	2.370044525	-5.320228727	1.90194007625944e-06	1.47951683448398e-05	4.67059265
LINC01237	-1.194879829	-1.444771198	-5.775788213	3.55856029416307e-07	3.46203254529306e-06	6.451174972
SNORD59B	-1.194551357	-2.349353879	-3.58096078	0.000718802	0.002631958	-0.628870783
ZFHX4-AS1	-1.194467683	3.122270716	-5.245805159	2.49277203192452e-06	1.86714205496914e-05	4.327139171
RFPL3	-1.194444835	-3.29186494	-3.766240725	0.00040125	0.001571992	-0.123249885
LOC100506804	-1.193698854	-0.324699553	-7.748003176	2.07177918049868e-10	6.07806205084589e-09	13.53107231
NPNT	-1.193373059	2.352657296	-3.084126665	0.003173964	0.009619053	-2.393038371
PAXBP1	-1.193142562	6.114700184	-8.586075341	8.80340878730224e-12	4.33334272764916e-10	16.41671901
LINC01219	-1.193007047	-3.021918075	-3.425007631	0.001160412	0.004010685	-1.086905849
PNN	-1.192896396	7.731007725	-8.24215468	3.20283590983406e-11	1.26083078944166e-09	15.06090845
GOLGA2P5	-1.19282161	1.673212254	-6.641254716	1.38001380989001e-08	2.12823249923119e-07	9.505209661
FANCD2	-1.192671619	3.725468303	-4.879001273	9.3004645717671e-06	5.90391882866282e-05	2.973740479
LEP	-1.192638076	-4.717093047	-4.15607741	0.000112481	0.000517356	0.991851026
GOLGA6L10	-1.192138159	1.832265918	-9.069742891	1.45275288044892e-12	1.02129300236453e-10	18.40736215
SNORD26	-1.19205667	-0.99364943	-6.045344628	1.30254007443198e-07	1.45458562684666e-06	7.401440517
ZNF204P	-1.191962743	2.97584856	-5.750640534	3.90663298637375e-07	3.74824064351424e-06	6.146935645
EFEMP1	-1.191819143	1.633835359	-3.190425834	0.00233372	0.007350716	-2.015128974
COL27A1	-1.191708	4.598863053	-6.249908508	6.04506588324871e-08	7.56219718371572e-07	7.79076287
BDH2	-1.191612813	4.474003904	-6.690930425	1.14339630558922e-08	1.80870105000836e-07	9.445664386
MOG	-1.191263118	-4.297443737	-3.935541353	0.000232622	0.000981938	0.336209481
STAB1	-1.191119718	-2.173984821	-3.231568386	0.00206863	0.006621471	-1.592028076
LOC339166	-1.190840472	-2.91544165	-3.562040606	0.000762254	0.002774535	-0.698631623
MGC32805	-1.190656481	-2.604201314	-3.777784281	0.00038675	0.001522409	-0.065643218
ZBTB18	-1.190446369	6.155169195	-5.546941739	8.29253283081452e-07	7.18912824916104e-06	5.069246094
MTX3	-1.189561125	5.319226004	-7.304317741	1.11520691021544e-09	2.52598665447513e-08	11.66254968
RBBP8NL	-1.188882823	-4.593861016	-3.360437305	0.001410246	0.004754119	-1.350048785
FLJ35934	-1.188706258	0.004534242	-8.130451495	4.8794982939589e-11	1.76857955446259e-09	14.92995678
CDCA7L	-1.18799484	3.208077409	-3.755748278	0.00041488	0.001618913	-0.603494382
LOC100335030	-1.187571105	-2.998226585	-3.98337507	0.000199008	0.00085368	0.534820224
LINC01311	-1.187535167	0.868888676	-7.879599756	1.25882751046631e-10	3.89632173116581e-09	14.06465792
GSDMB	-1.187528926	3.942458602	-7.978044943	8.67542956245815e-11	2.85600891529779e-09	14.31731618
CNFN	-1.187365276	-1.434624011	-3.790725916	0.000371093	0.001468429	-0.034260202
CARD14	-1.186784607	1.960328405	-8.463109613	1.39574183548672e-11	6.36097309265869e-10	16.20690901
MIR1285-1	-1.186537765	-3.652385858	-3.073064405	0.003276073	0.009877639	-2.059426804
FAM201A	-1.18588248	-0.263122475	-3.501027338	0.000920077	0.003273711	-0.947126389
GJA1	-1.184977279	4.313348239	-3.240716105	0.002013676	0.006471427	-2.252105344
MLLT4-AS1	-1.184486897	4.586960353	-8.3735458	1.95374182387857e-11	8.5502082169838e-10	15.73659546
KCNAB3	-1.184458852	2.590260278	-6.003149857	1.52526672130798e-07	1.66257217502407e-06	7.101143319
MIR558	-1.184317115	-4.779529282	-4.022350314	0.000175131	0.000764029	0.573279408
SEPT7-AS1	-1.183878165	1.13741489	-7.593697772	3.71851402683449e-10	1.00399878724531e-08	13.02226674
EDNRB	-1.183199498	7.214791081	-3.919229707	0.000245288	0.001026878	-0.526116889
LAMA1	-1.183115048	5.077814947	-6.50738493	2.28966883745476e-08	3.29465521940347e-07	8.696293758
LOC100270746	-1.182897942	-0.441346807	-6.021068032	1.42640676976058e-07	1.57231902189664e-06	7.318349728
LOC101928043	-1.182781155	-0.894758636	-4.894658753	8.79758890922774e-06	5.61571281423851e-05	3.431050553
LOC151475	-1.182571093	-3.833368634	-3.913734086	0.000249704	0.001042719	0.293077146
LINC01023	-1.180953604	-0.677326567	-5.964595239	1.76163564601922e-07	1.87914911344737e-06	7.118426637
LOC100129083	-1.180753894	-4.461505247	-4.068176142	0.000150587	0.000668313	0.733123008
CENPQ	-1.180620852	2.534401344	-6.422485383	3.15506307577144e-08	4.33911458282344e-07	8.642711589
TNFRSF10A	-1.180547637	-2.143169381	-3.413983676	0.00119986	0.004135599	-1.098468876

MAP4K1	-1.180485433	1.239928474	-5.439678172	1.22948220994839e-06	1.01124629032379e-05	5.190899039
NUP210L	-1.180360673	-0.331820879	-3.82907231	0.000328192	0.001322828	0.012885681
ZNF32-AS3	-1.180062448	-2.552234069	-3.305660175	0.001661317	0.005478243	-1.395218448
LOC285819	-1.179799815	-1.521918562	-4.445326815	4.22729965875366e-05	0.000220787	1.978804726
N4BP2L2-IT2	-1.17959293	0.886177592	-4.829217991	1.10940223951423e-05	6.89509273385364e-05	3.109973368
TMEM75	-1.179584639	-4.389043282	-3.411546096	0.001208753	0.004161909	-1.194572629
ZNF705E	-1.179383796	-0.453849207	-5.491639671	1.01617580719252e-06	8.53800861777492e-06	5.455840604
SLC15A1	-1.17926498	-4.290854162	-3.43768985	0.001116552	0.003880335	-1.115424743
SNORD19	-1.178422317	-1.491821658	-4.286895677	7.24997272249955e-05	0.000352891	1.476935637
CCDC159	-1.177667391	2.541637761	-10.79230384	2.85582972429852e-15	5.94394859073881e-13	24.50065257
LINC01481	-1.177597902	1.20074423	-13.46340837	3.67771063479569e-19	3.20698651075044e-16	32.89718256
ADAMTS4	-1.175741497	1.553052244	-4.18149375	0.000103328	0.000480265	0.920068515
SEC1P	-1.175415631	-0.522928392	-4.996727219	6.11473198051315e-06	4.08467805006076e-05	3.760445453
CECR2	-1.175171531	6.037411464	-5.023657585	5.5529086915937e-06	3.75204589583068e-05	3.214698409
ZNF141	-1.174914519	3.076798949	-8.38785995	1.85143462711704e-11	8.1837744980911e-10	15.89928685
CCL17	-1.174890485	-3.374333063	-3.283933847	0.001772147	0.005789495	-1.488080312
RNASE4	-1.174844137	1.749706944	-6.196006464	7.4029172568e-08	8.96022638435131e-07	7.870374232
LINC01558	-1.174618023	-2.662027044	-3.15342503	0.002599104	0.008070259	-1.803866611
HIST1H2AM	-1.174406143	-2.144521928	-3.579048254	0.000723085	0.002645807	-0.636189377
RDH16	-1.174177165	-0.490166777	-4.257391005	8.00895229139775e-05	0.000385673	1.339744793
MYBL1	-1.173930946	0.476350347	-4.389623899	5.11475439627211e-05	0.000260749	1.691783492
CSNK1A1L	-1.173787156	-2.514506915	-4.310595084	6.69135746199871e-05	0.000329004	1.55721712
TSPEAR	-1.172906706	-0.347646435	-3.223601509	0.002117639	0.006754623	-1.704384612
ARAP3	-1.172639069	3.320225507	-5.537335625	8.59086662610566e-07	7.41130474960349e-06	5.34070537
PERM1	-1.172579221	-2.699755938	-3.265599222	0.001871069	0.006063255	-1.508264791
STARD4-AS1	-1.171949417	5.588700412	-6.578887882	1.74732013874449e-08	2.59915099760457e-07	8.915928905
C1RL	-1.171087981	1.984956931	-6.044984567	1.30429678040818e-07	1.45593229715074e-06	7.303904865
RTKN2	-1.170918085	3.709063259	-5.442632685	1.21624797785444e-06	1.00246594320631e-05	4.954898083
MPHOSPH9	-1.170843657	3.952191962	-7.623439336	3.32188857275225e-10	9.15555745638887e-09	12.99063654
CEP135	-1.17045271	3.447576564	-7.052163799	2.905224854188e-09	5.65491962965769e-08	10.90104082
FRA10AC1	-1.170436133	4.881034405	-10.37370211	1.260069850721e-14	1.95926037435931e-12	23.00511697
LINC00640	-1.17008625	-3.969905127	-3.382648786	0.001319053	0.004491374	-1.249314818
EFS	-1.169908263	5.227684705	-5.750468919	3.90912056970748e-07	3.74926647384172e-06	5.885736682
ITGB7	-1.169281995	-1.336151565	-4.277771619	7.47689297157594e-05	0.000362237	1.445858502
SLC4A1	-1.169270481	-2.663412028	-4.278475419	7.4591463958723e-05	0.000361643	1.453415671
NEK2	-1.169195192	3.216012898	-3.925661478	0.000240217	0.0010082	-0.085640441
LOC148696	-1.169034799	-3.826216181	-3.107713285	0.002966113	0.009062915	-1.979282138
EXD1	-1.169026937	-2.332666149	-3.754355523	0.000416722	0.001625382	-0.129130437
SNORD6	-1.168914008	-1.359565553	-4.935614257	7.60497155951683e-06	4.94641272718279e-05	3.578586005
NUP62CL	-1.16890676	0.96668571	-4.593060556	2.53863387420782e-05	0.000141301	2.313959471
BTN3A3	-1.168285505	0.826119311	-4.051618008	0.000159045	0.000701678	0.588333191
DPY19L2P4	-1.168238444	1.37492136	-10.42109821	1.06403241350379e-14	1.74692973826992e-12	23.13512296
MIR1254-1	-1.168208132	0.518367436	-6.442536352	2.92510391616801e-08	4.06378792597405e-07	8.821111932
EFEMP2	-1.167920884	2.501752939	-4.613145047	2.36744531284819e-05	0.000133231	2.22220685
HMGB3P1	-1.167333799	-4.194094872	-3.098387457	0.003046722	0.009281319	-2.026213963
CCNB2	-1.167148372	4.008140109	-3.697331848	0.00049926	0.001906522	-0.89365872
ZNF846	-1.166894629	2.673712412	-9.899749846	6.931825210839e-14	8.10747503531448e-12	21.38913474
CHST9	-1.166454751	-0.891895095	-3.092816674	0.003095848	0.009409284	-2.002020295
TMEM26-AS1	-1.166348141	-4.125768899	-3.559811833	0.000767535	0.002791065	-0.760604025
BRCA1	-1.165909354	3.473769913	-4.497521249	3.53297094354678e-05	0.000188356	1.716437736
TYW5	-1.165821895	4.022745312	-9.309488813	5.99156474303397e-13	4.96473450948642e-11	19.22884566
PAQR6	-1.164921013	3.702235054	-4.900357533	8.62126181365602e-06	5.52384407311021e-05	3.050065947
LOC101928891	-1.164646462	-4.43900211	-3.570409228	0.000742735	0.00271204	-0.7490392
SNORA58	-1.164079359	-3.81341091	-3.663084165	0.000556127	0.002094628	-0.447048936
SLC10A1	-1.163423769	-2.684094193	-3.248595572	0.001967445	0.006339813	-1.553135395
ITGB3BP	-1.163262031	3.183894052	-7.071232955	2.70232677727535e-09	5.33064206744174e-08	10.99539106
LCA10	-1.163192351	-0.455504239	-6.963316881	4.07048606923495e-09	7.45118824571243e-08	10.69617784
LOC339539	-1.163032235	-1.139543452	-4.730971482	1.56831490859675e-05	9.30951447988723e-05	2.896935671
IL7	-1.16283274	0.445110145	-7.674166615	2.7406931533631e-10	7.78139013134766e-09	13.30421734
C21orf58	-1.162715063	3.377638957	-5.74662462	3.9652573990586e-07	3.7962205298557e-06	6.088479872
GREM2	-1.162440778	3.773044466	-3.868066156	0.000289474	0.001185935	-0.341484162
ZNF30-AS1	-1.162077267	-1.154617356	-5.001426587	6.0128235015145e-06	4.03290950559586e-05	3.795484769
LOC101929762	-1.161839565	-4.398645718	-3.287272431	0.001754674	0.005743814	-1.536954142

ATAD2	-1.161401006	4.334739208	-4.470174048	3.88161289348177e-05	0.000204551	1.508182504
LOC101928035	-1.160954475	-3.93907844	-3.657860969	0.000565326	0.002125039	-0.468464396
SNORA8	-1.160838098	1.749549082	-8.69739566	5.80570964987e-12	3.08157275451835e-10	17.05940684
AACSP1	-1.16056654	2.391188216	-3.641984938	0.000594191	0.002223736	-0.834974311
ZSCAN16-AS1	-1.160521896	2.926531775	-9.389603939	4.46189503793327e-13	3.82926206291201e-11	19.56364512
LAG3	-1.160104419	1.857691497	-4.816797525	1.15919005161114e-05	7.1670373875913e-05	2.978105587
C22orf15	-1.159876719	-1.203523903	-4.452950696	4.11816169269271e-05	0.00021594	1.997261528
EME1	-1.159815674	2.359006159	-4.804601058	1.21020810677488e-05	7.44979759813237e-05	2.883873415
TCERG1	-1.159673376	6.945686664	-10.44015565	9.94162542424338e-15	1.66320876480396e-12	23.14577336
ZNF493	-1.15962569	4.714111078	-8.974477534	2.06849061966142e-12	1.36010976491319e-10	17.95267896
HIPK1-AS1	-1.159430275	-1.29708316	-5.430816022	1.27003755996485e-06	1.03988302506683e-05	5.256312185
SNORD7	-1.159018682	-2.530650346	-3.436097486	0.001121972	0.003897119	-1.037866999
MIR331	-1.158192885	-0.859822682	-6.368593413	3.86625302589645e-08	5.17451474600106e-07	8.551050314
LOC101928650	-1.15817745	-1.653496344	-5.206566286	2.8736935553576e-06	2.11118237211693e-05	4.491321945
CCDC162P	-1.156286205	-3.832638893	-3.430020798	0.001142883	0.003958828	-1.110457568
NEIL3	-1.155580507	0.969071976	-3.253044696	0.001941785	0.006263235	-1.763804398
LINC00599	-1.155162395	6.203278603	-6.452169504	2.82063707180351e-08	3.9532290413034e-07	8.398206676
BTG3	-1.154570018	5.395338287	-7.036620972	3.08181084255074e-09	5.92891444788559e-08	10.64871029
CALR3	-1.154441805	-3.166134611	-3.907347326	0.000254931	0.001061028	0.30026728
OPA1-AS1	-1.153911734	-2.253780002	-3.447606757	0.001083358	0.003780881	-1.002865681
KCND1	-1.15140522	2.753858585	-5.118967967	3.94302247344345e-06	2.79126708732005e-05	3.923647664
MED23	-1.15116866	5.068786259	-6.529528173	2.10585423955678e-08	3.06688953797269e-07	8.779149496
CREBRF	-1.151108552	4.570987263	-7.682986471	2.65058246964781e-10	7.58255913638535e-09	13.15487594
CYP3A5	-1.151102173	-0.273205503	-5.358328237	1.65531570381735e-06	1.30767961742391e-05	4.988142009
LOC101060091	-1.150483055	-0.333450161	-7.013522392	3.36424237047518e-09	6.36041554764585e-08	10.88167772
SPICE1	-1.149590008	3.999097121	-8.935633766	2.38958786053775e-12	1.50553260650271e-10	17.86239032
TAPBPL	-1.149206518	-0.152346184	-4.551686428	2.93023566677147e-05	0.000159668	2.263370614
TNFRSF4	-1.149033558	-3.215177796	-3.317301375	0.001604661	0.00531329	-1.390393921
NKAP	-1.148880228	4.207666766	-8.301841989	2.55835877734826e-11	1.05664214940073e-09	15.50148848
SLC22A11	-1.147631948	-4.254947888	-3.798798633	0.000361637	0.001435338	-0.071680707
LINC01529	-1.14744152	-2.861691276	-3.50786941	0.000900941	0.003213408	-0.849467032
ERV3-1	-1.146387737	4.5496293	-7.05058113	2.92273360285964e-09	5.6806336267933e-08	10.78332833
LCN15	-1.14586433	-4.43747724	-3.339482884	0.001501724	0.005013271	-1.396935757
H1FX-AS1	-1.145570977	2.091809385	-5.67346014	5.19946608146572e-07	4.77877214643197e-06	5.953243884
LOC101929125	-1.145440994	-2.892780465	-3.287062682	0.001755767	0.005745968	-1.458396089
SPEM1	-1.145160817	-3.539763257	-3.611713774	0.000653176	0.002418801	-0.582370574
LOC101929140	-1.144944078	-0.411010605	-5.413876099	1.35126847918133e-06	1.09800429481095e-05	5.18468377
PHF3	-1.14455635	6.317301813	-8.82754641	3.57267489098798e-12	2.07263763683479e-10	17.3036203
MIR6719	-1.14423759	-2.937163672	-3.135719524	0.002735888	0.008438473	-1.861718049
TRIM64B	-1.144223384	-4.258913296	-3.31226678	0.001628936	0.005386247	-1.459772971
SEMA6A-AS1	-1.144115141	0.236187914	-4.641431395	2.14533385849532e-05	0.000122215	2.532391363
KHDC1L	-1.143479318	-1.924367312	-3.136554775	0.002729286	0.008421059	-1.844853949
IMPA2	-1.143327504	2.077453448	-3.871776632	0.000286027	0.001173635	-0.105937283
PROCA1	-1.142012274	3.267281678	-7.049115404	2.93904265244145e-09	5.7081347855977e-08	10.90567528
RNU5F-1	-1.141719107	-2.959100423	-3.292160035	0.001729387	0.005675094	-1.447405295
ADARB2-AS1	-1.141166922	-4.182138209	-3.275705286	0.001815917	0.00590698	-1.554291609
ARID3A	-1.141027675	3.232086083	-5.70280982	4.66430470584019e-07	4.35505356020748e-06	5.946168012
WDR31	-1.140428956	2.266834869	-4.633096738	2.20857257802083e-05	0.000125279	2.315276091
MAGEE2	-1.139654794	-1.505117543	-4.709154498	1.69305736151564e-05	9.9383933459789e-05	2.832074608
ADAM7	-1.139245287	-4.706191145	-3.846026415	0.000310782	0.001260149	0.042360863
MIR219A1	-1.139121549	1.28905304	-3.635876777	0.000605667	0.002262202	-0.718447163
TGDS	-1.138474919	2.856823211	-6.423728383	3.14029817013361e-08	4.32556026738622e-07	8.619859019
DIAPH2	-1.137738325	2.383092566	-3.995022699	0.000191562	0.000825758	0.236866345
ANGPTL5	-1.137637901	-4.107050982	-3.191923837	0.002323532	0.007322116	-1.774381376
RBM28	-1.137352133	4.924508852	-7.414436872	7.34147725632545e-10	1.78525545829301e-08	12.11213273
KIAA1524	-1.137222621	3.296142101	-5.635772222	5.97661141072856e-07	5.38628603545134e-06	5.6970927
MALAT1	-1.137129221	10.99875745	-3.341389263	0.001493175	0.004990403	-2.380983902
SNX20	-1.137106525	-0.74314357	-3.652423643	0.000575058	0.002156406	-0.475687548
PDC	-1.137002029	-1.540606056	-4.336298494	6.1327827556391e-05	0.000305387	1.633411037
ADAMTS3	-1.13629228	3.569415399	-3.918823467	0.000245612	0.001027908	-0.156194669
SYTL1	-1.136205395	0.117755973	-4.477484706	3.78525827659413e-05	0.000200071	2.003869933
SGOL1	-1.135676255	1.706885956	-4.048193608	0.000160851	0.00070863	0.482657556
FTCD	-1.135360625	1.023926373	-7.014247944	3.35498937049801e-09	6.34806256480844e-08	10.89742796

MIRLET7F1	-1.135309186	-3.256233481	-3.257474249	0.001916552	0.006192728	-1.5537247
LOC10192777(	-1.135077395	-1.530738655	-3.369630386	0.001371801	0.004643825	-1.228270275
NSUN5P1	-1.134699658	4.101650737	-20.26288293	2.21575458057445e-27	2.92845204141622e-23	51.76458078
FUT3	-1.134104912	1.80546719	-3.632232733	0.000612614	0.002285566	-0.790772677
SNORA18	-1.134010089	0.822844048	-7.201012693	1.65090226597028e-09	3.53346555436376e-08	11.58351117
C8orf37-AS1	-1.133748524	-3.734942247	-3.638438444	0.000600829	0.002246987	-0.514508466
CFAP69	-1.133632513	2.201653141	-6.614927448	1.52460465743851e-08	2.31874999482579e-07	9.376435695
SPANXB1	-1.133621624	-4.525568803	-3.603756009	0.000669587	0.002474372	-0.658694545
LINC00051	-1.13337547	-3.581545707	-3.258598533	0.001910197	0.006177175	-1.566440482
VCAN	-1.133277604	8.095403008	-5.49747217	9.94644982205213e-07	8.39982454141546e-06	4.794137814
DTWD1	-1.133221781	3.609122294	-8.975662394	2.05940993434614e-12	1.35751578041325e-10	18.0335204
SYCP2L	-1.132836063	2.113910449	-7.310446047	1.08955584645893e-09	2.4700025462649e-08	11.95431045
MOGAT1	-1.132522651	-4.460905667	-3.870201728	0.000287485	0.001178885	0.129983596
IDUA	-1.132443726	2.405280173	-5.687015323	4.94514264738711e-07	4.57205161239536e-06	5.973512184
INHBC	-1.131931423	-0.647605167	-3.115492547	0.002900406	0.008896081	-1.963872121
LRRIQ3	-1.131853874	-0.278257734	-5.326003278	1.8623546075434e-06	1.45386583674681e-05	4.876522329
BRIP1	-1.131648934	2.435120763	-3.467034692	0.001021024	0.003591793	-1.349497141
MIR563	-1.131519083	-3.486959103	-3.448321354	0.001081002	0.003774654	-1.042237315
SENP7	-1.131201292	6.270112645	-10.34621996	1.39004690724416e-14	2.11167298271178e-12	22.83636572
BISPR	-1.130421735	-2.376902756	-3.114950385	0.00290494	0.008908956	-1.895434694
LOC10012920:	-1.129471081	-0.817094175	-6.650092584	1.33460809448714e-08	2.06664884367771e-07	9.557976671
PVRIG	-1.129257654	3.822645304	-5.805660894	3.184823464434e-07	3.14003874059619e-06	6.250230743
MYO15A	-1.129182425	2.349609612	-4.520385846	3.26507503297268e-05	0.000176134	1.930587843
TBX6	-1.128875941	-0.668166988	-3.962402367	0.000213126	0.000906298	0.438148536
ROCK1	-1.128782848	5.865482885	-7.62781324	3.26724768052283e-10	9.03380313172176e-09	12.8379979
RNF139-AS1	-1.128550097	1.647973708	-12.13030437	2.86903543367779e-17	1.16672636336008e-14	28.85712046
CASP8AP2	-1.128054665	4.975822135	-8.00144845	7.94113729043075e-11	2.67739900507597e-09	14.31146789
UCN2	-1.127606663	-2.010598445	-4.385876217	5.18057430598183e-05	0.000263393	1.794516809
MIR4648	-1.126780526	-3.593130346	-3.442072576	0.001101765	0.003835312	-1.064699944
RFTN2	-1.125856242	3.71326853	-6.034372414	1.35714238579211e-07	1.50538584488639e-06	7.098605682
NHLH2	-1.12548612	5.662925624	-6.667559919	1.249202233225e-08	1.96314878899146e-07	9.241296763
MKRN3	-1.125428861	3.686336746	-5.907957548	2.17623720542862e-07	2.25940605071071e-06	6.639199854
EBF1	-1.125052972	6.056239519	-5.296821902	2.0710738201915e-06	1.5918782869765e-05	4.177613913
GCC2	-1.12505011	5.028630433	-6.55316851	1.92581335329427e-08	2.83277820632318e-07	8.871112492
CHRDL1	-1.124977099	4.493284903	-3.54043379	0.000814938	0.002943198	-1.428062749
GRAMD1C	-1.12484732	2.011468523	-8.120130619	5.07321786460878e-11	1.82590246133569e-09	14.94864294
TBC1D8B	-1.124755164	-0.026071942	-3.787447588	0.000375001	0.001482117	-0.136075468
CFAP44	-1.123887899	3.17145555	-5.234649749	2.5956925897609e-06	1.93109885238249e-05	4.28150241
SNORD58A	-1.123508232	-1.59240122	-3.635415135	0.000606543	0.002265153	-0.481997246
UBE2E1-AS1	-1.123499456	-1.492004302	-5.687890337	4.92915534229242e-07	4.55886505118319e-06	6.144837637
MIR3064	-1.123180399	2.326131768	-5.52199167	9.08954930989394e-07	7.78057179107599e-06	5.38990366
KLHL24	-1.122990924	6.079915566	-6.60350815	1.59192551269729e-08	2.40316202610665e-07	8.971521366
BTBD19	-1.122857864	0.45700836	-3.584871375	0.000710121	0.002604861	-0.775473941
WDHD1	-1.12278805	4.091392405	-6.06808304	1.19623196296077e-07	1.35475576165133e-06	7.177923145
SAT1	-1.12257706	6.53008822	-7.483709733	5.64409941175992e-10	1.42763692480736e-08	12.25612765
PLAGL1	-1.121984085	5.160029248	-6.815543922	7.13026618175134e-09	1.19590308364361e-07	9.839660368
TMEM63A	-1.121800232	2.469830026	-6.008655635	1.49418692503359e-07	1.63476171313795e-06	7.131613491
CHRNA2	-1.121195378	-0.799991284	-3.249541973	0.00196196	0.006324451	-1.595994297
SNORD102	-1.121057039	-0.784589959	-6.034146469	1.35829034016021e-07	1.50602720475902e-06	7.364048192
SNORD33	-1.121013844	-3.94610258	-3.191174194	0.002328625	0.007335542	-1.766595301
SIGLEC1	-1.119979297	-1.107516621	-3.176921437	0.00242748	0.007604359	-1.764774595
CAPN13	-1.119936156	-3.340056905	-3.89603341	0.000264451	0.001095989	0.260141406
P2RY12	-1.119689323	-3.731325838	-3.095187074	0.003074855	0.009350855	-2.006403116
PCMTD1	-1.119146198	5.296636849	-13.4563665	3.76107826706253e-19	3.20698651075044e-16	33.33386906
F11R	-1.11906893	3.680378267	-5.027555613	5.47590807761832e-06	3.70664989028643e-05	3.49312771
CYP2A7	-1.119047822	-2.912790185	-3.341962346	0.001490614	0.004983104	-1.310466463
TPTE2P6	-1.118969993	-2.071172259	-3.630163149	0.000616594	0.002298142	-0.490317175
CD163L1	-1.118498877	0.01452897	-5.168830926	3.29387707763318e-06	2.37758199871868e-05	4.323320877
CEP83	-1.118302211	3.080629525	-5.702151427	4.67568958929284e-07	4.35798670358877e-06	5.960209551
MIR661	-1.117978142	-4.492755752	-3.665874401	0.000551271	0.002078413	-0.476586669
PAGR1	-1.117869068	6.143590341	-11.43491855	3.04774579720108e-16	8.95122940637956e-14	26.64943068
SNORA55	-1.117690774	-2.44281262	-4.356895498	5.71814223950359e-05	0.000287189	1.704242377
GOLGB1	-1.117521947	6.971839437	-6.013200681	1.46900468627131e-07	1.61054337918746e-06	6.725591676

KLRAP1	-1.117218978	2.322690534	-7.911321114	1.11648096229295e-10	3.51751385891414e-09	14.17019843
RHBDF2	-1.116880444	0.251916354	-3.85219945	0.000304667	0.001240489	0.033056575
SERINC4	-1.116877652	2.987137588	-5.310095186	1.97342270523633e-06	1.5283762779816e-05	4.568840447
NOL8	-1.11627819	5.01026586	-8.423377688	1.62021509182977e-11	7.27116222790089e-10	15.88555435
RNASEH2B-AS	-1.116125979	-4.132532061	-3.347723916	0.001465097	0.004910221	-1.354889172
ARHGEF39	-1.115735202	1.549980983	-4.743069344	1.50306777551248e-05	8.98678817238665e-05	2.759217041
CDC37L1-AS1	-1.115634888	0.510538106	-4.632196185	2.21551354271716e-05	0.000125536	2.481593271
CENPJ	-1.115336386	4.673385551	-9.120041061	1.20588425802455e-12	8.73291468283918e-11	18.49052221
LOC101927932	-1.115162202	-3.264260914	-3.79309131	0.000368298	0.001458241	-0.043053316
PKN2	-1.114998489	5.606395391	-7.210782993	1.59077336859629e-09	3.42976447407061e-08	11.28599523
STK31	-1.114589754	-0.635109687	-6.402680699	3.39985433992888e-08	4.61810636008942e-07	8.677433487
MIR320A	-1.114321176	-4.094506252	-3.464353416	0.001029419	0.003615552	-1.028921008
PVRL3-AS1	-1.114072377	-0.537384561	-6.794667628	7.71755927750981e-09	1.28059161570883e-07	10.08614157
MIR2116	-1.114063272	-4.547413349	-3.859462155	0.000297619	0.001214977	0.092611268
BEAN1-AS1	-1.113953662	-0.126699462	-7.590342782	3.766133284009e-10	1.01271822071424e-08	12.97502627
HSD17B13	-1.112298664	-1.769013762	-4.411343162	4.74902730793736e-05	0.000244605	1.873542518
LOC101928008	-1.110799005	-2.980476585	-3.395602087	0.00126848	0.004340982	-1.166319645
NCAPG	-1.110159603	4.179007784	-4.999974553	6.04413225721775e-06	4.04877212253008e-05	3.331349643
LINC01511	-1.109235538	-4.836643321	-3.773236643	0.000392401	0.001543274	-0.183521716
COX6B2	-1.109099406	0.103430547	-5.800206028	3.25003854724838e-07	3.19361594495972e-06	6.525707189
SNORD115-4	-1.108202144	-2.997582002	-3.202958993	0.002249759	0.007125315	-1.687470231
NEXN-AS1	-1.108068738	-1.941023922	-3.799508179	0.000360817	0.001432912	-0.000421944
WASIR2	-1.107987233	-2.195427487	-3.172984842	0.002455471	0.007678394	-1.743788593
CYP39A1	-1.107860682	0.294469341	-5.3911661	1.46826492010673e-06	1.17929646408937e-05	5.079142761
OR5K2	-1.107581206	-4.206374402	-3.603232809	0.000670679	0.002477508	-0.640712214
LOC100131347	-1.106377245	-1.043840995	-6.757555707	8.88326330009968e-09	1.4512441212085e-07	9.933163958
ZNF724P	-1.106310711	0.068538077	-5.267674046	2.30253978722267e-06	1.74687785137428e-05	4.661174594
LOC100289511	-1.106044898	0.159850487	-5.236021574	2.58281370281442e-06	1.92476782087661e-05	4.547506409
LINC00479	-1.10594692	-5.080782229	-4.301498723	6.90063744037706e-05	0.000337786	1.429073042
CDC14A	-1.105084029	2.434044925	-4.478622125	3.7704781477087e-05	0.00019949	1.782590909
DPEP2	-1.104456369	-4.924190711	-4.024446149	0.000173929	0.000759722	0.570435004
SNORD87	-1.103966126	-0.737182249	-5.351351613	1.69798612468287e-06	1.33938726451037e-05	4.976331898
LINC01069	-1.103719297	-4.581799448	-3.246083254	0.001982076	0.006377627	-1.661094362
RTTN	-1.103506774	3.995689847	-6.183534873	7.75799351735326e-08	9.31276306286097e-07	7.614060257
TAS2R19	-1.102063778	-0.473473659	-6.157551616	8.55304158508293e-08	1.01655822040691e-06	7.803958183
VWA7	-1.101887227	1.723645189	-7.171129854	1.8492830949399e-09	3.88569952691148e-08	11.45533096
MIR302B	-1.10130853	-4.807530972	-3.971082782	0.000207169	0.000883129	0.414423067
MIR25	-1.100676713	-0.754218387	-5.377076034	1.5458219043919e-06	1.23148614824566e-05	5.065307554
HMCN1	-1.100169609	2.17068686	-3.332505227	0.001533412	0.005108088	-1.695380949
WDR11-AS1	-1.09992147	0.516743805	-5.430905284	1.26962256384658e-06	1.03988302506683e-05	5.206524121
DUSP5P1	-1.099333572	0.708633426	-5.050233036	5.04831821712559e-06	3.45794753649341e-05	3.874990305
KIAA1875	-1.098574602	1.903007814	-10.19648426	2.37674520981136e-14	3.25515575807998e-12	22.40443988
ALOX12P2	-1.098439455	-2.545908526	-3.257049835	0.001918956	0.006197919	-1.525401617
TMEM69	-1.096643297	3.744432665	-6.009153968	1.49140508136065e-07	1.63239381017002e-06	7.002330006
ARHGEF26-AS	-1.096495756	-0.274467783	-4.129961328	0.000122701	0.0005592	0.926109392
SEPT10	-1.096345652	4.155700211	-3.26366733	0.00188179	0.006093516	-2.166722273
TCEA3	-1.096106399	0.026514353	-4.309490222	6.71644417884566e-05	0.000330115	1.469889306
MIR590	-1.096014923	-1.013286827	-3.450018412	0.001075428	0.003756678	-1.031079374
PAPPA	-1.095834032	2.649518731	-4.303277891	6.85921012528182e-05	0.000336119	1.18354282
ASIC3	-1.095063474	3.525033551	-7.388518238	8.10055984324767e-10	1.93076734298076e-08	12.15016421
ITIH4	-1.094709365	2.851334233	-6.224192848	6.65883022057219e-08	8.20572770258204e-07	7.885468459
GAL3ST4	-1.094319244	4.795507608	-5.649169067	5.6880134031553e-07	5.16493501496406e-06	5.563498354
EIF2D	-1.094082208	4.513090378	-6.197552192	7.36004979013133e-08	8.92013737288131e-07	7.605426976
NDST3	-1.092747578	3.378893148	-5.920406071	2.07751802152126e-07	2.17141296413094e-06	6.719510809
PKD1L2	-1.0923832	0.237600403	-3.560025604	0.000767027	0.002789601	-0.825212818
MIRLET7D	-1.091430306	-1.445001726	-5.27687092	2.22688013024624e-06	1.69585486841829e-05	4.729969477
DEPDC7	-1.090377047	0.809925826	-5.990587336	1.5986044104497e-07	1.73109014262257e-06	7.179291426
TXLNB	-1.089413415	-0.551056114	-4.744630715	1.49484290351373e-05	8.94570578867522e-05	2.918878623
LOC283710	-1.08932678	-1.08311861	-4.568068226	2.76859739779076e-05	0.000151894	2.363321597
SNORA40	-1.08923692	1.489582648	-7.318292226	1.05757412153582e-09	2.41982447802205e-08	12.00533009
ANKRD33B	-1.088699739	2.762349768	-3.820272615	0.000337594	0.001355351	-0.349302976
CD9	-1.088541835	4.605844526	-3.467942854	0.001018195	0.003582795	-1.654133946
UBE2Q2P1	-1.087966829	2.368466338	-8.891499909	2.81574736021068e-12	1.70707912780846e-10	17.76631466

ZNF37BP	-1.08793804	5.156102207	-7.913301134	1.10815101592747e-10	3.50379854114961e-09	13.96477679
PRICKLE4	-1.087836601	2.933096332	-9.285614709	6.54246302782083e-13	5.35408437196247e-11	19.1878153
LRRC69	-1.087445603	-2.436132285	-3.228304658	0.002088577	0.006674003	-1.598668559
SCOC-AS1	-1.086427272	0.882614668	-5.50505361	9.67330433282908e-07	8.18996982165481e-06	5.445511414
CBR4	-1.085801822	4.883032676	-13.31158099	5.97140431556979e-19	4.38450361870712e-16	32.87957344
BPIFB1	-1.085712591	-4.634238652	-3.800674247	0.000359473	0.001428649	-0.088495453
LOC10192963	-1.084806215	-4.749058457	-3.75915543	0.000410406	0.001602874	-0.219275195
COPG2IT1	-1.08448333	6.657539032	-5.308554326	1.98452160275482e-06	1.53529261853273e-05	4.180898064
KIAA0101	-1.084301887	3.447498488	-3.920188317	0.000244526	0.001024011	-0.135250619
LOC10192822	-1.084080242	0.260411932	-6.778843252	8.19467960692768e-09	1.3504361973187e-07	10.04344362
KCNT2	-1.084077979	3.84238973	-7.17682866	1.80969394972391e-09	3.81464435191804e-08	11.32854876
LOC643770	-1.083688974	-1.535813615	-3.866567912	0.000290876	0.001190575	0.193594375
CD27	-1.083244672	-1.949791423	-3.376328851	0.001344416	0.00456421	-1.202401181
AGK	-1.083178789	4.929327689	-7.587053696	3.81341002204031e-10	1.02231102548267e-08	12.75964555
CCDC15	-1.082556354	2.232825286	-8.366031576	2.00970184947654e-11	8.74662415336708e-10	15.84803905
SNORD28	-1.081868115	-0.134759292	-5.328892902	1.84285194819035e-06	1.44301644790974e-05	4.881330388
IFT74	-1.081815716	3.849341029	-7.83313921	1.50081548210688e-10	4.52865931946703e-09	13.78348739
MIR641	-1.081783481	-0.528364125	-4.626149885	2.26267014718708e-05	0.000127879	2.527751614
FHOD1	-1.080806765	2.77243501	-5.269551785	2.28688917003789e-06	1.73655103222096e-05	4.44916881
CRNDE	-1.080562453	5.288103981	-4.356961427	5.71685981936901e-05	0.000287179	1.014738784
SFTPA2	-1.080290062	-1.387584943	-4.049283819	0.000160274	0.000706347	0.740960803
DNMT3L	-1.080140797	-5.101203353	-4.238107227	8.54609991002886e-05	0.000408055	1.22632603
STK3	-1.080074538	2.24292466	-5.509810212	9.5057305099667e-07	8.08185830073817e-06	5.35414053
CD46	-1.079904129	6.445713517	-9.590327971	2.13777425113988e-13	2.02536870180575e-11	20.10358304
LY6D	-1.079515054	-5.095068783	-4.388783523	5.12944263825164e-05	0.000261296	1.707939636
MIR4516	-1.079489208	-4.39237781	-3.699655464	0.000495611	0.00189421	-0.371781349
ZNF431	-1.078622302	3.93885179	-7.835296315	1.48861077044522e-10	4.50211081180533e-09	13.78354565
BBX	-1.078300339	6.251572828	-8.994813249	1.91807935744899e-12	1.28355928241643e-10	17.92591838
BRCAT54	-1.078285078	1.663108437	-10.57798173	6.09081470294555e-15	1.11804517390944e-12	23.70334027
C1S	-1.078262788	2.272277227	-4.979482611	6.50343555745221e-06	4.30948388293141e-05	3.491294401
EXTL3-AS1	-1.077378538	-0.852637499	-6.492671002	2.42056415325265e-08	3.45505181083682e-07	8.993982556
LOC10013007	-1.07705519	-4.527662406	-3.313565769	0.001622639	0.005368114	-1.474012662
SULT2B1	-1.077043586	-1.664828635	-3.37919777	0.001332846	0.004530167	-1.198215971
RNF151	-1.076538919	-3.49281895	-3.199569386	0.002272181	0.007187718	-1.71952533
CHTF18	-1.076148976	4.913227466	-8.268189742	2.90377521639529e-11	1.16827230281548e-09	15.31423907
CAPRIN2	-1.076072911	5.567989436	-11.62731844	1.57533304622853e-16	5.07814370865351e-14	27.32760151
PTER	-1.076055535	2.610964797	-3.391858809	0.001282903	0.004385751	-1.586785209
SMC2	-1.075715743	4.865318943	-5.814077164	3.08673881804664e-07	3.06160477213609e-06	6.155364665
CFAP43	-1.075558129	2.399092626	-4.380549458	5.27554729197302e-05	0.000267656	1.464653415
FOXN2	-1.075093139	4.717802969	-6.558439696	1.88780847952957e-08	2.78323236900095e-07	8.922613731
PPWD1	-1.075005152	4.774939366	-9.683131644	1.5233846609194e-13	1.52528889174555e-11	20.53604553
ZNF92	-1.074590035	4.838593817	-6.768057025	8.53665169434655e-09	1.40067854895507e-07	9.693260646
IZUMO4	-1.074380708	1.836106692	-8.221119642	3.46691212042258e-11	1.33587300406895e-09	15.32167404
RARG	-1.074200007	1.152347145	-3.993477077	0.000192534	0.000829274	0.373627941
PTTG1	-1.074115784	4.55784988	-4.180583296	0.000103643	0.00048156	0.530212092
CCNB3	-1.073953295	1.419996599	-4.308414826	6.740949576518e-05	0.000331196	1.339451057
P4HA1	-1.073828843	6.494419654	-4.304365254	6.83401031021429e-05	0.000335083	0.74291793
GPC6	-1.073722769	4.01406699	-4.398892893	4.95544020442337e-05	0.000253458	1.315255234
TNFSF9	-1.073710843	2.18697455	-4.402970752	4.88688995085107e-05	0.000250485	1.562408223
RAD54B	-1.073638384	2.539853537	-5.728316416	4.24375407026637e-07	4.01485867356303e-06	6.109068036
PMAIP1	-1.073504241	3.009164415	-4.019966465	0.000176509	0.000768768	0.234953789
FAM60A	-1.07339499	6.101083467	-5.673923749	5.1905577373055e-07	4.77224391896334e-06	5.532111014
TMED7-TICAM	-1.073187553	-3.573185355	-3.352629071	0.001443699	0.004849192	-1.310745805
CCDC66	-1.072936467	4.47024463	-8.809898007	3.81550957460291e-12	2.20208219618949e-10	17.36351465
C1RL-AS1	-1.071968445	0.471509843	-5.611343778	6.54063413791571e-07	5.83294811631329e-06	5.843281603
LOC10192955	-1.071789955	-2.563605096	-3.187146979	0.002356166	0.007410822	-1.711488227
RRP7BP	-1.071566245	3.962654817	-15.81857643	2.88882085542495e-22	4.77251260446548e-19	40.33793444
MNAT1	-1.07143701	3.804354869	-9.077228214	1.41301970083243e-12	9.96009326722765e-11	18.39374587
PLS1	-1.071069708	0.981188318	-5.875110481	2.45961848530989e-07	2.51314632478533e-06	6.756057969
PGM5P2	-1.070483784	1.141759849	-5.742809345	4.02175967522092e-07	3.83780409729656e-06	6.273007811
ACSM3	-1.070036002	-1.259101121	-5.95313085	1.83868917745573e-07	1.94875986478298e-06	7.072909992
LOC10192909	-1.069271023	-1.193800858	-5.667992371	5.30567916382283e-07	4.85109018807779e-06	6.076637202
LOC10050707	-1.068555665	-0.225230318	-3.457085093	0.001052508	0.003685871	-1.074560874

GALNT7	-1.068303901	4.522550841	-8.206925607	3.65735322786405e-11	1.38680990313378e-09	15.11945363
RRM2B	-1.068268465	5.740663051	-6.144104694	8.9957736173448e-08	1.06012164078143e-06	7.284543077
METTL21B	-1.067634117	2.026977726	-6.153150427	8.69551101926079e-08	1.03024851085666e-06	7.694019307
BOD1L1	-1.067555673	6.983658681	-7.063282827	2.78513670596290e-09	5.47357015232098e-08	10.64920822
GS1-24F4.2	-1.06751783	-2.070826722	-5.311944802	1.96018063227444e-06	1.52124059462449e-05	4.850389184
LOC10012952(	-1.067487117	-4.672878465	-3.787672974	0.000374731	0.001481272	-0.129626899
SCAF11	-1.066910276	6.189648146	-7.178547156	1.79792282589226e-09	3.79892038823423e-08	11.12459448
ALS2CL	-1.066575894	1.05033481	-4.290333334	7.16622280833207e-05	0.000349299	1.319131992
NPHS1	-1.066369252	0.940753446	-5.562389215	7.83412321641814e-07	6.84107627947079e-06	5.64424401
GOLGA8N	-1.066225544	1.168033646	-8.551645803	1.00149029277733e-11	4.81316234708784e-10	16.51654461
NUDT12	-1.066143322	4.311143985	-8.645451683	7.04966412560645e-12	3.5835340736953e-10	16.76805213
MELK	-1.066050033	3.095969681	-4.203825266	9.58853120844829e-05	0.000451065	0.806385416
KLRC4	-1.065059078	-5.097783845	-3.545162402	0.00080312	0.002905279	-0.868127809
HIST1H3C	-1.064576871	-4.382672366	-3.078932144	0.003221535	0.009737545	-2.089458304
LOC339666	-1.0644667	0.551123534	-4.705291917	1.71613179427116e-05	0.000100604	2.720725509
OPN5	-1.064106794	-2.628007283	-3.107888169	0.002964621	0.009059402	-1.920950226
MIR5047	-1.063119561	4.651100211	-5.834597561	2.85996055952579e-07	2.86245124838869e-06	6.254437668
SNORD38A	-1.062996382	-2.168508497	-3.465221147	0.001026695	0.003607422	-0.952912725
MAD2L1	-1.062858206	4.185690458	-4.375290583	5.37097060446373e-05	0.000271923	1.213696953
CATSPER4	-1.062492878	-3.360197934	-3.401218387	0.001247129	0.004280261	-1.166986274
BRWD3	-1.062440729	5.431359203	-8.203547935	3.70419741309582e-11	1.40075894449731e-09	15.03020022
MYEOV	-1.062028064	-3.416572164	-3.36481641	0.001391808	0.004701553	-1.269778496
PCOLCE	-1.061984198	2.247143991	-4.088700401	0.000140705	0.000629107	0.546161651
NR2E3	-1.061775922	-0.141738878	-3.498566386	0.000927054	0.003296762	-0.965025674
AOC3	-1.061675244	2.028814625	-4.943453255	7.3955200729888e-06	4.83158136651787e-05	3.392376606
SGK494	-1.061081807	4.872725817	-7.066285878	2.75356249922718e-09	5.41957688325182e-08	10.80776431
LOC644838	-1.060824768	-2.789955674	-3.982305679	0.000199705	0.00085614	0.538068855
LOC10272370	-1.06052786	-1.999449052	-4.109831231	0.000131187	0.000592764	0.934228318
LOC10192945	-1.05968066	-2.559044886	-3.589845803	0.000699222	0.002569518	-0.607059842
C1orf54	-1.059272669	3.348297514	-8.474650166	1.33659915218748e-11	6.14440441561244e-10	16.20479506
SLC12A3	-1.058810008	-4.235889794	-3.285192633	0.00176554	0.005775091	-1.531862786
IZUMO1	-1.058605339	-2.312414687	-3.429094167	0.001146104	0.00396832	-1.052231944
ZNF252P-AS1	-1.057145163	-0.7355483	-5.702670189	4.66671689585118e-07	4.3557672213289e-06	6.196734321
BNIPL	-1.056754827	-0.646498654	-4.291084944	7.14803727435627e-05	0.000348541	1.455302506
CDCP2	-1.056547042	-2.483735862	-3.47799775	0.00098737	0.00348686	-0.919781203
LOC10192971	-1.055785977	0.12009428	-4.727547127	1.58728139908643e-05	9.39677698142253e-05	2.825351381
GATA1	-1.055625342	-2.595609161	-3.229339129	0.002082235	0.006657763	-1.60095185
SNORD29	-1.055151312	-1.434835617	-4.595395362	2.51812869859724e-05	0.000140307	2.461690101
CTSW	-1.054588697	-1.255411119	-3.98847185	0.000195716	0.000841333	0.551728564
LOC389705	-1.05424656	0.890314243	-6.202104486	7.23522973421021e-08	8.8011425478315e-07	7.938710757
GPR84	-1.054173179	-4.141984874	-3.233878954	0.002054618	0.006584592	-1.664396679
CAHM	-1.053928757	1.693480591	-5.465550809	1.11825768452025e-06	9.29233114584215e-06	5.245682245
LOC10192922	-1.053465361	-1.813590388	-3.692938459	0.000506231	0.001929518	-0.311455957
OXR1	-1.053301931	5.698787244	-14.42350115	1.83606326088642e-20	2.42663300875053e-17	36.3253949
SLC44A5	-1.053146835	4.087304853	-7.43092649	6.89603236114021e-10	1.69407828440538e-08	12.25562493
SULT1C4	-1.052035724	1.752321488	-5.881035291	2.40592857700167e-07	2.46526072558473e-06	6.726929861
FAM205BP	-1.052020566	-5.091250864	-4.293660938	7.08604938371987e-05	0.000345696	1.40336236
ESCO1	-1.051927842	4.609427121	-7.636402309	3.16255509125088e-10	8.79030691136011e-09	12.9749799
PARP14	-1.051874147	1.800370809	-3.775274048	0.00038986	0.001534421	-0.365960279
CUBN	-1.051558936	2.554683344	-5.308362347	1.98590874433938e-06	1.53529261853273e-05	4.608913065
HIST1H3I	-1.051415474	-4.35070997	-3.513026328	0.000886768	0.003168845	-0.906925223
FAM157B	-1.051340878	-2.225899338	-3.35236295	0.001444853	0.004850983	-1.262393355
GTPBP3	-1.051340408	4.306827637	-6.76325239	8.69352889536781e-09	1.423767343812e-07	9.732164684
PRKD3	-1.050932293	4.7263735	-5.706694894	4.59768088150397e-07	4.30501235355276e-06	5.779524197
RPL29P2	-1.050826728	-4.761215243	-3.324178174	0.001572055	0.005219057	-1.461845197
ZNF726	-1.050786939	1.097859811	-7.296364351	1.14940009760368e-09	2.59232873549129e-08	11.93137828
RFESD	-1.050307018	-0.529647507	-7.814454953	1.61081684719002e-10	4.83299906036024e-09	13.75458448
LINC00853	-1.049857468	-3.158475526	-3.20228303	0.002254214	0.00713515	-1.69631953
NEUROD4	-1.049738376	3.674491243	-3.678811352	0.000529283	0.0020061	-0.901805933
PLCD1	-1.049703184	4.706748327	-7.600271785	3.62694695438413e-10	9.83293218925496e-09	12.82993068
SLC4A7	-1.049449412	5.665050575	-8.312934093	2.45379811112596e-11	1.02628552961064e-09	15.42278916
PABPN1	-1.049309485	4.630365616	-7.675322559	2.72871051536217e-10	7.7556994680181e-09	13.11913748
LOC10192946	-1.04908883	-2.654416036	-3.409407244	0.001216608	0.004184592	-1.115583979

SATB1	-1.048851778	5.424480749	-5.966683156	1.74795146513682e-07	1.86605820185628e-06	6.656377083
LOC101927211	-1.048374871	-2.374245347	-3.42630134	0.001155864	0.003999078	-1.060739712
MOB1B	-1.048306793	6.100931747	-7.103880804	2.38728015666903e-09	4.817021097804e-08	10.84865816
LOC442132	-1.048215337	-4.167634786	-3.323263686	0.001576355	0.005230047	-1.423944056
SBSPON	-1.048011161	0.42178153	-6.472270766	2.61446174878323e-08	3.70354058979566e-07	8.930404796
CYMP	-1.047565003	-4.960956286	-3.097163186	0.003057455	0.009310797	-2.086353109
AGAP9	-1.047374503	3.397025742	-4.504253299	3.45194364617478e-05	0.000184408	1.747886421
ZNF678	-1.047275495	4.598785479	-7.495235366	5.40255482918131e-10	1.37577776300337e-08	12.445928
SPACA6P	-1.047010158	2.911419726	-7.882591536	1.24465872076096e-10	3.86604746955046e-09	14.03611475
TMEM105	-1.046508324	-1.592831475	-3.511927669	0.00088977	0.003177852	-0.830778795
TSSK3	-1.046486921	3.309007938	-4.956665728	7.05523830233644e-06	4.6275710681305e-05	3.29384956
MIR1908	-1.046471397	-3.055923875	-3.099062755	0.003040817	0.009267602	-1.962205624
SLC16A4	-1.046425125	1.021391074	-5.138837646	3.67050202224485e-06	2.61868771805663e-05	4.156345524
WDR90	-1.046263314	4.487445574	-10.22840237	2.11949748084972e-14	2.98003600592025e-12	22.50996378
SLC44A4	-1.046078663	-1.840083924	-3.378503178	0.001335638	0.004538491	-1.197209166
SNORD5	-1.046005048	0.753096611	-5.529229139	8.85086275374365e-07	7.60828797299856e-06	5.538475815
MRPS31P5	-1.045751689	4.549591474	-9.830708921	8.90439631737939e-14	9.92951626699285e-12	21.0831686
NXF3	-1.045390087	-4.183979853	-3.188159535	0.002349213	0.00739247	-1.789127135
SKA1	-1.045151513	1.467348047	-3.365299235	0.001389789	0.004696533	-1.515488651
FAM83A	-1.044552698	-4.446855726	-3.724117435	0.000458708	0.001769561	-0.303292498
SENP3-EIF4A1	-1.043211914	-1.719991248	-4.479941996	3.75339766542281e-05	0.000198666	2.09274402
RAPGEF6	-1.042704403	5.966904884	-9.064923345	1.47892889900619e-12	1.03693707128463e-10	18.19945881
MCM8	-1.042425774	3.221711755	-5.66605093	5.34390652525057e-07	4.87929123253707e-06	5.813937385
MXRA8	-1.042116269	4.257609697	-6.243438376	6.19397922018854e-08	7.70472718716441e-07	7.804117164
C6orf10	-1.041324134	-4.448885901	-3.137856678	0.002719026	0.008392341	-1.939981115
COL21A1	-1.040668211	2.072253041	-3.241694757	0.002007878	0.006453581	-1.93367589
APOA5	-1.04055943	-4.882096908	-3.444143199	0.001094843	0.003815424	-1.137977907
LINC00648	-1.040091365	1.453468588	-5.587305778	7.14696132468805e-07	6.30228298880958e-06	5.696800563
MIR550A3	-1.039832557	-4.595804039	-3.321135344	0.001586404	0.005260749	-1.458075012
TMF1	-1.039582169	6.02950238	-6.865962945	5.88931863509984e-09	1.01680182548396e-07	9.958361255
DFNB59	-1.039086069	1.108760466	-8.043815324	6.76676389404394e-11	2.31691541465367e-09	14.66908601
SMCR5	-1.038603363	-1.85568851	-3.685253062	0.00051865	0.001970601	-0.333098253
RBMS3-AS1	-1.037964124	-5.226736035	-5.2693719	2.28838392691985e-06	1.73718702872695e-05	4.654028551
CEP83-AS1	-1.037732584	0.974077861	-7.979456478	8.62927211553855e-11	2.84830225885504e-09	14.43150633
CKS2	-1.036993664	4.520441013	-5.241833027	2.52894972542685e-06	1.89033736790281e-05	4.135665202
PROM2	-1.036952242	2.452068923	-7.024952612	3.2213901052984e-09	6.1658946164629e-08	10.87676648
GAPDHS	-1.036918213	2.921354261	-11.58510972	1.82001374142703e-16	5.79619556953501e-14	27.20744853
SLC52A1	-1.036639549	-2.62576773	-3.195499604	0.002299381	0.007258992	-1.691723629
SPAG4	-1.036434667	1.988396089	-3.7086806	0.000481676	0.001847113	-0.587929355
STX16	-1.036277056	5.779898573	-6.880868111	5.56558089606254e-09	9.76211014104984e-08	10.03151871
MAGOH2P	-1.035379332	0.144785969	-4.653222507	2.05888258413176e-05	0.000117951	2.577398995
LOC102723809	-1.035027976	-1.514938208	-3.526783772	0.00084999	0.00305352	-0.790123743
PABPC1L	-1.034973166	2.794657594	-4.704798886	1.71909913132853e-05	0.000100734	2.495283307
MIR4305	-1.034907796	-4.878927891	-3.921527313	0.000243465	0.00101989	0.258867928
MAGEC3	-1.034869865	-0.310084305	-4.5102626	3.3811480566039e-05	0.000181507	2.137969719
TAS2R31	-1.034357047	-0.528398516	-6.216120579	6.86398255888424e-08	8.42319642427981e-07	8.012500886
SYCP2	-1.034064675	0.794058806	-6.040214739	1.32779156767813e-07	1.47779008456572e-06	7.358389159
ZNF563	-1.033843586	0.732934509	-6.607308139	1.56920056989475e-08	2.37156538959565e-07	9.415356272
TNFAIP8	-1.033593208	1.015338296	-3.602475863	0.000672263	0.002482181	-0.785489754
RFPL3S	-1.033590143	-0.389379921	-4.746536185	1.48486508755936e-05	8.89606501800921e-05	2.917412061
TEX14	-1.033432275	1.244503925	-5.780742384	3.49370268477651e-07	3.40520070304932e-06	6.401963204
FLJ33360	-1.033417119	-2.031145431	-3.848775495	0.000308044	0.001251158	0.147563507
COL9A3	-1.03312315	3.083039951	-3.962799282	0.00021285	0.00090527	0.046424044
MIR4701	-1.033097655	-4.317607952	-3.451667486	0.001070037	0.003739825	-1.077803365
OMA1	-1.03275722	2.172385681	-8.502460843	1.20421235450149e-11	5.59419071468151e-10	16.34898274
MIR4666A	-1.032263886	-4.22445317	-3.412547097	0.001205094	0.004150931	-1.181278797
LOC285847	-1.03222761	0.607353209	-5.631852636	6.0637547408022e-07	5.45953893029972e-06	5.909012837
TSPAN19	-1.032039258	0.815178042	-6.500327744	2.35154551462774e-08	3.3708461273403e-07	9.023816685
CLDND2	-1.031855047	0.888327721	-5.89094819	2.3186857653077e-07	2.38667526613623e-06	6.817692953
SKIL	-1.031829535	5.610273712	-8.31104475	2.47129998571893e-11	1.03197270967628e-09	15.4192545
DLEU2	-1.031519587	1.238256638	-4.341200128	6.0315341131848e-05	0.000301155	1.463672682
LINC00458	-1.031099457	-4.731548653	-3.117115656	0.002886869	0.008858674	-2.015448116
RIPK3	-1.030670457	-4.086184906	-3.20332518	0.002247349	0.007120242	-1.74260547

ANGPT2	-1.030600559	1.642740614	-3.787025458	0.000375507	0.001483673	-0.312038288
LOC339260	-1.030534373	2.843388267	-6.510792439	2.26037545676274e-08	3.25782467004413e-07	8.94202106
KANTR	-1.030474769	1.602426897	-7.474700429	5.84041422756962e-10	1.46054559392004e-08	12.57899985
CRIP1	-1.030404111	0.490197255	-3.690887359	0.000509517	0.001940365	-0.470187696
ZNF708	-1.030400656	4.726329415	-7.944419829	9.85128527100789e-11	3.19508004378591e-09	14.11889982
GPBAR1	-1.030240969	-1.34401837	-4.348647546	5.88080264311007e-05	0.000294257	1.670366491
TEK	-1.029769715	-0.68772176	-3.523038981	0.000859855	0.003084346	-0.850388663
HYPK	-1.029589567	-1.542124152	-5.379525741	1.53205332884737e-06	1.22235936134689e-05	5.080853231
ANP32A-IT1	-1.02909022	-0.88370634	-3.439730631	0.001109643	0.003859369	-1.069462731
LOC100288123	-1.028604031	0.812249217	-6.469141472	2.64554307443755e-08	3.7395529458079e-07	8.91041436
PAIP2B	-1.028440784	4.845864315	-7.343914461	9.59533258811038e-10	2.2219923744703e-08	11.85295721
SNORD59A	-1.028389823	-2.771029462	-3.156723306	0.002574343	0.007999955	-1.799587658
LOC101928324	-1.028324806	-1.038537586	-5.320970529	1.89680882406412e-06	1.47682909120727e-05	4.877128346
LOC100505622	-1.028274192	-4.024507086	-3.267555262	0.001860272	0.006033446	-1.566591103
IKZF2	-1.028065603	2.800572916	-6.654514234	1.31245342621521e-08	2.04071067147922e-07	9.477465522
SPAG17	-1.027818038	0.421433964	-3.636447963	0.000604585	0.002259437	-0.622660573
CCNL2	-1.027598193	7.357384358	-12.12000236	2.96991660811305e-17	1.18196508874087e-14	28.93733132
ENOSF1	-1.027538549	3.363938265	-5.697847254	4.75079787059707e-07	4.41708899449499e-06	5.912566469
FBXW10	-1.027511709	-1.827065983	-3.892459667	0.000267528	0.001106835	0.275134118
LINC01410	-1.027195366	-0.111245123	-4.223464245	8.97716320315421e-05	0.000424952	1.207176485
LOC101927056	-1.027095298	-0.761523436	-4.969237352	6.74575578673231e-06	4.45331075701037e-05	3.676270689
ZNF624	-1.02694374	3.708369426	-9.694611468	1.46094377287251e-13	1.47958340031951e-11	20.63888744
KLK14	-1.026816379	-0.528664431	-4.383124682	5.22942296239141e-05	0.000265672	1.741122934
GPX8	-1.026520378	3.557446959	-4.539906778	3.05211961259158e-05	0.000165679	1.845029683
CELA2A	-1.026340512	-2.332004073	-3.197232817	0.00228776	0.007230942	-1.67836008
KIF2C	-1.026288953	4.236459029	-3.372722417	0.001359095	0.004605764	-1.874863132
RNVU1-15	-1.026266626	-3.498713941	-3.375138215	0.001349245	0.004577666	-1.245273864
LINC01159	-1.025978628	2.882949183	-4.925323689	7.88878584714826e-06	5.1071338794433e-05	3.237006736
ARMCX4	-1.025870404	5.765244502	-15.68465978	4.25989816434812e-22	6.62364048107141e-19	40.05560436
MBNL2	-1.02560794	3.427956768	-5.025227522	5.5217694381468e-06	3.73195938528597e-05	3.516438826
PPT2-EGFL8	-1.025094151	-0.256947579	-5.378836117	1.5359170756087e-06	1.22507230113352e-05	5.058567899
LOC105376671	-1.024986754	-2.466970775	-3.272138688	0.001835203	0.005961229	-1.482201838
SPC24	-1.0248815	1.766218621	-3.672667694	0.000539618	0.002039132	-0.66791064
MIR6509	-1.024843948	-5.022066867	-4.681600951	1.86453711881461e-05	0.000108153	2.668913357
RNU6-45P	-1.024219529	-2.408636566	-3.4107988	0.001211492	0.004169709	-1.10395778
LOC102724050	-1.024151577	-2.957845608	-3.304910697	0.001665029	0.005489113	-1.412785424
HNRNPA1L2	-1.023069729	4.659902821	-7.94169113	9.95344696358693e-11	3.21244766286317e-09	14.11465422
CKAP2	-1.022990765	5.590058803	-5.363073759	1.626897494802e-06	1.28715299252024e-05	4.449847222
POLI	-1.022987806	5.042938859	-7.399536706	7.76870537706408e-10	1.86512433453165e-08	12.04320443
LOC101927188	-1.022367679	-1.781196718	-4.006711034	0.00018436	0.000797836	0.618131184
LIPJ	-1.022097251	-3.749458281	-3.296133375	0.001709084	0.005617535	-1.473726898
GOLGA8R	-1.021807364	-0.07731959	-3.376863837	0.001342251	0.004558032	-1.311928935
FCHO2	-1.021681213	4.10631437	-8.535395976	1.06435836028119e-11	5.06010513261022e-10	16.37620659
ST7-AS1	-1.021505598	1.421940072	-5.694794424	4.80479288983405e-07	4.45631896340293e-06	6.082238202
TTLL3	-1.021449254	2.509891605	-6.96386178	4.06207706129087e-09	7.44094823015256e-08	10.64625526
MEM256-PLSC	-1.021340368	-1.488425304	-4.331159891	6.24069969110291e-05	0.000309669	1.618200332
SNORA71C	-1.021101136	-1.861748675	-3.826404559	0.000331016	0.001333192	0.079736001
LOC101928812	-1.020564944	1.068442907	-7.061393417	2.80518719678031e-09	5.50887913614367e-08	11.06941615
CKMT2-AS1	-1.019825614	2.527591098	-6.848009912	6.30430687777078e-09	1.07649705232632e-07	10.21541795
HAPLN3	-1.019534207	0.701023869	-5.2676046	2.30312062316625e-06	1.74687785137428e-05	4.625497198
OGT	-1.019393908	7.700043116	-10.14281449	2.88235987825293e-14	3.84795043746767e-12	22.05933745
DCDC2B	-1.018827612	1.8428091	-9.197682672	9.04982930407235e-13	6.93374313027665e-11	18.86797697
SERHL2	-1.017880868	3.3728359	-8.603241526	8.25554272493673e-12	4.07885534295799e-10	16.67772131
GULP1	-1.017149164	4.082429685	-5.264606784	2.32833220994169e-06	1.76295632498965e-05	4.272062258
MBOAT1	-1.016885884	1.043786134	-4.061659163	0.000153863	0.000681478	0.596287858
FCER2	-1.016684736	-4.807929467	-3.335148478	0.001521334	0.00507297	-1.43521666
COL25A1	-1.01653952	2.130439891	-5.199747738	2.9454981825715e-06	2.15733869381858e-05	4.270005711
LOC101929550	-1.016430028	-2.991942431	-3.240066661	0.002017531	0.006478305	-1.588453661
MUC6	-1.016051268	1.187834081	-3.692402986	0.000507087	0.001932223	-0.541501856
MGAM2	-1.015948865	-1.275458351	-3.515103194	0.000881121	0.003152072	-0.831377217
SMC5-AS1	-1.015800952	-0.260763445	-5.523765639	9.03046305289507e-07	7.74253097233784e-06	5.562877574
FRRS1	-1.015787388	-0.384726697	-4.083243638	0.00014327	0.000638948	0.788869088
CCDC79	-1.015784572	-4.285621567	-3.139233594	0.002708214	0.008362875	-1.925112466

BAZ2B	-1.015060128	6.540058809	-8.237116026	3.26419195558265e-11	1.27621807964012e-09	15.08906993
LOC283177	-1.014894573	1.85637433	-7.265868852	1.29051193494497e-09	2.87046480662797e-08	11.79998527
THRB	-1.014849068	3.340997658	-4.465272197	3.94755354905806e-05	0.000207819	1.625971352
IL3RA	-1.014841776	4.27886455	-5.38981105	1.47555354937432e-06	1.18271081030358e-05	4.69212611
BLM	-1.014744925	2.76207626	-3.611810868	0.000652978	0.002418407	-0.97334743
PPP1R32	-1.014310726	2.939902176	-7.384219582	8.2338411875283e-10	1.9537264282759e-08	12.17995074
SMC5	-1.013058573	5.702628522	-6.795900846	7.68156233081074e-09	1.27622084909064e-07	9.717937318
LOC100129055	-1.01258911	-0.095516298	-5.667248808	5.32028797882232e-07	4.86110807682852e-06	6.061415843
NEK5	-1.012484558	-0.541423961	-4.259205245	7.96013726632778e-05	0.000383534	1.34840835
SNORA71E	-1.012425662	-1.62069394	-3.869533778	0.000288106	0.001181246	0.204868709
NEDD1	-1.012135345	4.374713986	-5.801013396	3.24030335871811e-07	3.18760471458861e-06	6.164414927
CTSK	-1.011253613	2.127752506	-5.202121575	2.92030154705131e-06	2.14185157583816e-05	4.278507423
RFX3	-1.011118098	6.442807951	-5.947516722	1.87763292850185e-07	1.9844650619388e-06	6.510819376
CDRT15P2	-1.010578483	-4.976952304	-3.568747458	0.000746573	0.002724206	-0.791181264
FCER1G	-1.009698079	-2.218849965	-3.098129284	0.003048983	0.009287135	-1.936036787
ITGA2	-1.009441293	2.221214225	-3.255784902	0.001926139	0.006218079	-1.914711451
SNORD115-25	-1.0093056	-4.971771581	-4.17204521	0.000106643	0.000494022	1.026335474
C10orf11	-1.00836454	-3.462829017	-3.075188899	0.003256228	0.009826678	-2.043558749
GNB3	-1.008236057	3.713990282	-8.541281066	1.04114434946352e-11	4.95866100709354e-10	16.42742536
PTCHD4	-1.008178155	2.73501412	-7.265727338	1.29120560574853e-09	2.87046480662797e-08	11.7528069
TMEM147-AS1	-1.008154245	2.934804474	-10.4809898	8.59637330735368e-15	1.49492062916631e-12	23.4363458
LOC100130872	-1.008143832	-2.208004094	-3.585487335	0.000708763	0.0026006	-0.613293346
TAS2R5	-1.008112319	0.043221849	-5.164211536	3.34929986998017e-06	2.4110033622872e-05	4.305936902
SRSF5	-1.007654156	8.231056961	-11.63647607	1.52679722709786e-16	4.98244828442934e-14	27.28165059
PADI2	-1.007614961	1.797449569	-5.505116387	9.67107382629572e-07	8.18996982165481e-06	5.376806478
LOC102723582	-1.007531057	0.264218242	-6.333941742	4.40564499627595e-08	5.7736447291305e-07	8.432577084
PIK3C2A	-1.007470634	5.849169156	-6.365107524	3.91739842589278e-08	5.22972689856686e-07	8.096183068
MEIS1	-1.007440126	6.077974772	-3.847748841	0.000309064	0.001254529	-0.68023469
MAFA-AS1	-1.007363396	-0.890123995	-4.75249625	1.45407480334149e-05	8.73138556945151e-05	2.958894656
RIF1	-1.006865441	5.824323191	-5.815704138	3.06812600686577e-07	3.04623452727139e-06	6.068693086
LOC440300	-1.006471284	0.206132515	-5.829936717	2.90997422928836e-07	2.90221951293542e-06	6.628090183
GOLGA8O	-1.006327193	-0.98836614	-3.266449924	0.001866366	0.006049498	-1.535922154
LOC440982	-1.006041246	0.836774558	-3.635880904	0.000605659	0.002262202	-0.668263588
SELENBP1	-1.005660945	2.006735973	-4.028236355	0.000171775	0.00075137	0.384562646
DICER1-AS1	-1.004549304	3.380089204	-6.868256119	5.83831426576868e-09	1.00931432954 26e-07	10.22051922
ITGA10	-1.004413301	0.156451518	-3.632834543	0.000611462	0.002282233	-0.607313878
PDXDC2P	-1.004358783	4.2311316	-7.861968296	1.3456758497934e-10	4.12647908788736e-09	13.8559735
SPAG5	-1.004021882	4.701793739	-4.608428669	2.40660251584974e-05	0.000135233	1.91959811
REL	-1.003478842	3.205770452	-5.771991193	3.60907519125143e-07	3.49830159627242e-06	6.198444625
HCG25	-1.003197777	-0.650076304	-6.816987286	7.09135026226503e-09	1.19013118401557e-07	10.16259152
UHRF1	-1.003065318	4.527003245	-3.422735751	0.00116844	0.004036775	-1.774931269
PRAMEF8	-1.002395785	-5.035824112	-3.582781265	0.000714749	0.002620381	-0.755352059
SCARNA6	-1.002177055	-4.053955491	-3.29561436	0.001711723	0.00562551	-1.492334913
GSDMA	-1.001812026	-4.671493347	-3.296430494	0.001707575	0.005613971	-1.53098635
KCTD9	-1.001751297	4.083269729	-8.149929701	4.53389313259693e-11	1.66450551630465e-09	14.94490008
LOC158435	-1.001621097	-2.12883238	-4.183584796	0.000102608	0.000477759	1.163922457
ANKRD31	-1.001513309	-0.319201926	-5.544309454	8.37324876541281e-07	7.25434561180455e-06	5.63613759
BAZ1A	-1.001476199	5.168910388	-5.344555866	1.74059300050436e-06	1.3705419953033e-05	4.424177353
COL19A1	-1.001344149	0.667506921	-3.974193089	0.000205074	0.00087544	0.363991249
FLJ33534	-1.001312746	-3.821516421	-3.104940033	0.002989873	0.009124122	-1.986017733
KCNK6	-1.001038222	-0.929566282	-4.713395432	1.66807223968892e-05	9.81789212017308e-05	2.83158141
GABPA	-1.000887481	5.311228698	-9.027107065	1.70149728150611e-12	1.16216221297289e-10	18.10011929
SNHG19	-1.000716957	2.51856859	-5.198346504	2.96047167434868e-06	2.16650464474138e-05	4.225749861
SAP30L-AS1	-1.000674395	-0.372695131	-4.320821843	6.4634314927338e-05	0.000319163	1.533062967

Table S4: GO Terms based on the upregulated genes in the embedded and suspended samples at DIS 30

Embedded GO Terms based on Upregulated Genes					
source	***term_name***	***term_id***	***adj_p_value***	***neg_log10_ adj_p_value***	***intersections***
GO:MF	G protein-coupled peptide receptor activity	GO:0008528	4.786807774138947e-10	9.31995401	GAL,PRLHR,MC5R,RAMP1,NPFFR2,RAMP3,GALR1,NPSR1,GPR83,NPBWR1,GALR2,OPRD1,CCKBR,OPRL1,TACR1,GALR3,NTSR2,HCRTR1,SSTR4,MCHR1
GO:MF	peptide receptor activity	GO:0001653	1.3896472655122566e-9	8.857095422826601	GAL,PRLHR,MC5R,RAMP1,NPFFR2,RAMP3,GALR1,NPSR1,GPR83,NPBWR1,GALR2,OPRD1,CCKBR,OPRL1,TACR1,GALR3,NTSR2,HCRTR1,SSTR4,MCHR1
GO:MF	transmembrane signaling receptor activity	GO:0004888	1.4021957695256696e-9	8.853191347503996	GAL,GPR50,PRLHR,ADRA1B,MC5R,CD300E,OR2W3,IL31RA,RAMP1,ADRA2A,DRD5,NPFFR2,FFAR4,HTR6,RAMP3,GABRA5,GALR1,PTGER4,SOSTDC1,PTGER2,CHRM4,CD300LB,NPSR1,PTPRH,GPR83,CD300C,GRM7,HRH1,GABRG3,NPBWR1,GPR68,ITGA11,GALR2,OPRD1,HTR7,ADGRF4,CRLF2,CCKBR,FCMR,ADRA1D,IL1RL2,DRD2,OPRL1,IL15RA,TACR1,GALR3,SPHK1,P2RY2,NTSR2,HTR5A,HCRTR1,OR2L13,GABRE,MTNR1B,SSTR4,CHRNA6,NPY,HTR1E,GPR45,IL7R,MCHR1,NFAM1,CHRNB2,HRH2,DRD1,ADGRA1,TNFRSF11A,PPARG,ALK,GABRQ,HTR2A,ADORA1,RET
GO:MF	inorganic molecular entity transmembrane transporter activity	GO:0015318	7.232379541218261e-9	8.140718791004538	SLC6A2,SLC18A3,KCNK12,GABRA5,SLC5A10,NIPAL2,KCNS2,KCNK13,SLC10A4,MCOLN3,AQP5,KCNA1,GABRG3,KCNS1,OTOP2,KCNK3,KCNE4,TRPM8,SLC24A3,SLC6A11,SLC1A4,SLC4A3,KCNK4,ASIC2,CACNA1S,GABRE,KCNA5,TRPV2,SLC5A1,KCNC2,MIP,SCN2B,SLC6A9,KCNK15,OTOP3,ATP1A3,ATP6V0B,SLC6A12,ATP2B3,ATP6V0C,SLC37A1,NCS1,KCNIP1,ATP6V0D1,SLC36A1,ATP6V0A1,GABRQ,SLC45A1,HCN2
GO:MF	transmembrane transporter activity	GO:0022857	8.495665869385026e-9	8.070802576528125	SLC6A2,SLC18A3,KCNK12,GABRA5,SLC5A10,NIPAL2,RBP4,KCNS2,KCNK13,SLC10A4,MCOLN3,SLC6A17,AQP5,KCNA1,GABRG3,KCNS1,SLC7A4,OTOP2,SV2C,KCNK3,KCNE4,TRPM8,SLC24A3,SLC6A11,SLC2A6,SLC1A4,SLC4A3,KCNK4,ASIC2,GJC2,CACNA1S,GABRE,KCNA5,CYBB,SLC7A5,TRPV2,CHRNA6,SLC5A1,KCNC2,MIP,SCN2B,SLC6A9,KCNK15,OTOP3,GJA10,ABCG5,ATP1A3,ATP6V0B,SLC6A12,CHRNB2,ATP2B3,SLC35F4,ATP6V0C,SLC37A1,NCS1,KCNIP1,ABCA3,ATP6V0D1,SLC36A1,ATP6V0A1,GABRQ,SLC45A1,HCN2,TMEM120A,SLC22A3,PQLC2
GO:MF	transporter activity	GO:0005215	9.639004258448116e-9	8.015967827860358	SLC6A2,SLC18A3,KCNK12,ATP10A,GABRA5,SLC5A10,NIPAL2,RBP4,KCNS2,KCNK13,SLC10A4,MCOLN3,SLC6A17,AQP5,KCNA1,GABRG3,KCNS1,SLC7A4,OTOP2,SV2C,APOF,KCNK3,KCNE4,TRPM8,SLC24A3,SLC6A11,SLC2A6,SLC1A4,SLC4A3,KCNK4,ASIC2,GJC2,CACNA1S,GABRE,KCNA5,CYBB,SLC7A5,TRPV2,CHRNA6,SLC5A1,KCNC2,MIP,SCN2B,SLC6A9,KCNK15,OTOP3,GJA10,ABCG5,ATP1A3,ATP6V0B,SLC6A12,CHRNB2,ATP2B3,SLC35F4,ATP6V0C,PITPNM1,SLC37A1,NCS1,KCNIP1,ABCA3,ATP6V0D1,ATP8A2,SLC36A1,ATP6V0A1,GABRQ,SLC45A1,HCN2,TMEM120A,SLC22A3,PQLC2
GO:MF	monoatomic ion transmembrane transporter activity	GO:0015075	1.3165760714728032e-8	7.880554042394295	SLC6A2,SLC18A3,KCNK12,GABRA5,SLC5A10,NIPAL2,KCNS2,KCNK13,SLC10A4,MCOLN3,KCNA1,GABRG3,KCNS1,OTOP2,KCNK3,KCNE4,TRPM8,SLC24A3,SLC6A11,SLC1A4,SLC4A3,KCNK4,ASIC2,CACNA1S,GABRE,KCNA5,CYBB,TRPV2,CHRNA6,SLC5A1,KCNC2,SCN2B,SLC6A9,KCNK15,OTOP3,ATP1A3,ATP6V0B,SLC6A12,CHRNB2,ATP2B3,ATP6V0C,NCS1,KCNIP1,ATP6V0D1,SLC36A1,ATP6V0A1,GABRQ,SLC45A1,HCN2,TMEM120A
GO:MF	molecular transducer activity	GO:0060089	1.9965692994455656e-8	7.6997156111673695	FOLR3,GAL,GPR50,PRLHR,ADRA1B,MC5R,TNFRSF9,CD300E,OR2W3,IL31RA,RAMP1,ADRA2A,DRD5,NPFFR2,FFAR4,HTR6,RAMP3,GABRA5,GALR1,DMBT1,CD28,PTGER4,SOSTDC1,PTGER2,CHRM4,CD300LB,NPSR1,PTPRH,GPR83,CD300C,GRM7,HRH1,GABRG3,NPBWR1,GPR68,ITGA11,GALR2,OPRD1,HTR7,ADGRF4,CRLF2,CCKBR,FCMR,ADRA1D,IL1RL2,DRD2,OPRL1,IL15RA,TACR1,GALR3,SPHK1,P2RY2,NTSR2,HTR5A,HCRTR1,OR2L13,GABRE,MTNR1B,SSTR4,CHRNA6,NPY,HTR1E,SQSTM1,GPR45,IL7R,MCHR1,NFAM1,CHRNB2,HRH2,DRD1,ADGRA1,TNFRSF11A,PPARG,ALK,GABRQ,HTR2A,ADORA1,RET

GO:MF	signaling receptor activity	GO:0038023	1.9965692994455656e-8	7.6997156111673695	FOLR3,GAL,GPR50,PRLHR,ADRA1B,MC5R,TNFRSF9,CD300E,OR2W3,IL31RA,RAMP1,ADRA2A,DRD5,NPFFR2,FFAR4,HTR6,RAMP3,GABRA5,GALR1,DMBT1,CD28,PTGER4,SOSTDC1,PTGER2,CHRM4,CD300LB,NPSR1,PTPRH,GPR83,CD300C,GRM7,HRH1,GABRG3,NPBWR1,GPR68,ITGA11,GALR2,OPRD1,HTR7,ADGRF4,CRLF2,CCKBR,FCMR,ADRA1D,IL1RL2,DRD2,OPRL1,IL15RA,TACR1,GALR3,SPHK1,P2RY2,NTSR2,HTR5A,HCRTR1,OR2L13,GABRE,MTNR1B,SSTR4,CHRNA6,NPY,HTR1E,SQSTM1,GPR45,IL7R,MCHR1,NFAM1,CHRNB2,HRH2,DRD1,ADGRA1,TNFRSF11A,PPARG,ALK,GABRQ,HTR2A,ADORA1,RET
GO:MF	neuropeptide receptor activity	GO:0008188	2.5664101888787962e-8	7.590673929204435	GAL,PRLHR,NPFFR2,GALR1,NPSR1,GPR83,NPBWR1,GALR2,TACR1,GALR3,NTSR2,SSTR4,MCHR1
GO:MF	neurotransmitter receptor activity	GO:0030594	1.0113070459667859e-7	6.99511697	DRD5,HTR6,GABRA5,CHRM4,HRH1,GABRG3,HTR7,DRD2,HTR5A,GABRE,CHRNA6,HTR1E,CHRNB2,HRH2,DRD1,GABRQ,HTR2A
GO:MF	monoatomic cation transmembrane transporter activity	GO:0008324	4.640038588538739e-7	6.333478407652069	SLC6A2,SLC18A3,KCNK12,SLC5A10,NIPAL2,KCNS2,KCNK13,SLC10A4,MCOLN3,KCNA1,KCNS1,OTOP2,KCNK3,KCNE4,TRPM8,SLC24A3,SLC6A11,KCNK4,ASIC2,CACNA1S,KCNA5,TRPV2,CHRNA6,SLC5A1,KCNC2,SCN2B,SLC6A9,KCNK15,OTOP3,ATP1A3,ATP6V0B,SLC6A12,CHRNB2,ATP2B3,ATP6V0C,NCS1,KCNIP1,ATP6V0D1,SLC36A1,ATP6V0A1,SLC45A1,HCN2
GO:MF	G protein-coupled receptor activity	GO:0004930	5.154014372516522e-7	6.287854374707133	GAL,GPR50,PRLHR,ADRA1B,MC5R,OR2W3,RAMP1,ADRA2A,DRD5,NPFFR2,FFAR4,HTR6,RAMP3,GALR1,PTGER4,PTGER2,CHRM4,NPSR1,GPR83,GRM7,HRH1,NPBWR1,GPR68,GALR2,OPRD1,HTR7,ADGRF4,CCKBR,ADRA1D,DRD2,OPRL1,TACR1,GALR3,SPHK1,P2RY2,NTSR2,HTR5A,HCRTR1,OR2L13,MTNR1B,SSTR4,NPY,HTR1E,GPR45,MCHR1,HRH2,DRD1,ADGRA1,PPARG,HTR2A,ADORA1
GO:MF	G protein-coupled amine receptor activity	GO:0008227	6.415359990833253e-7	6.192778968650572	ADRA1B,ADRA2A,HTR6,CHRM4,HRH1,HTR7,ADRA1D,HTR5A,HTR1E,HRH2,HTR2A
GO:MF	inorganic cation transmembrane transporter activity	GO:0022890	8.003203030528595e-7	6.096736165497672	SLC6A2,SLC18A3,KCNK12,SLC5A10,NIPAL2,KCNS2,KCNK13,SLC10A4,MCOLN3,KCNA1,KCNS1,OTOP2,KCNK3,KCNE4,TRPM8,SLC24A3,SLC6A11,KCNK4,ASIC2,CACNA1S,KCNA5,TRPV2,SLC5A1,KCNC2,SCN2B,SLC6A9,KCNK15,OTOP3,ATP1A3,ATP6V0B,SLC6A12,ATP2B3,ATP6V0C,NCS1,KCNIP1,ATP6V0D1,SLC36A1,ATP6V0A1,SLC45A1,HCN2
GO:MF	channel activity	GO:0015267	1.0161E-06	5.993049595693588	KCNK12,GABRA5,KCNS2,KCNK13,MCOLN3,AQP5,KCNA1,GABRG3,KCNS1,OTOP2,KCNK3,KCNE4,TRPM8,SLC24A3,SLC1A4,KCNK4,ASIC2,GJC2,CACNA1S,GABRE,KCNA5,CYBB,TRPV2,CHRNA6,KCNC2,MIP,SCN2B,KCNK15,OTOP3,GJA10,CHRNB2,ATP6V0C,NCS1,KCNIP1,GABRQ,HCN2,TMEM120A
GO:MF	passive transmembrane transporter activity	GO:0022803	1.0722E-06	5.96972741	KCNK12,GABRA5,KCNS2,KCNK13,MCOLN3,AQP5,KCNA1,GABRG3,KCNS1,OTOP2,KCNK3,KCNE4,TRPM8,SLC24A3,SLC1A4,KCNK4,ASIC2,GJC2,CACNA1S,GABRE,KCNA5,CYBB,TRPV2,CHRNA6,KCNC2,MIP,SCN2B,KCNK15,OTOP3,GJA10,CHRNB2,ATP6V0C,NCS1,KCNIP1,GABRQ,HCN2,TMEM120A
GO:MF	salt transmembrane transporter activity	GO:1901702	1.3821E-06	5.85945385	SLC6A2,SLC18A3,KCNK12,GABRA5,SLC5A10,KCNS2,KCNK13,SLC10A4,MCOLN3,AQP5,KCNA1,GABRG3,KCNS1,KCNK3,KCNE4,TRPM8,SLC24A3,SLC6A11,SLC1A4,SLC4A3,KCNK4,ASIC2,CACNA1S,GABRE,KCNA5,TRPV2,SLC5A1,KCNC2,MIP,SCN2B,SLC6A9,KCNK15,ATP1A3,SLC6A12,ATP2B3,NCS1,KCNIP1,GABRQ,HCN2,PQLC2
GO:MF	monoatomic ion channel activity	GO:0005216	5.2661E-06	5.278509639480863	KCNK12,GABRA5,KCNS2,KCNK13,MCOLN3,KCNA1,GABRG3,KCNS1,OTOP2,KCNK3,KCNE4,TRPM8,SLC24A3,SLC1A4,KCNK4,ASIC2,CACNA1S,GABRE,KCNA5,CYBB,TRPV2,CHRNA6,KCNC2,SCN2B,KCNK15,OTOP3,CHRNB2,ATP6V0C,NCS1,KCNIP1,GABRQ,HCN2,TMEM120A
GO:MF	G protein-coupled serotonin receptor activity	GO:0004993	1.287E-05	4.890417343065344	HTR6,CHRM4,HRH1,HTR7,HTR5A,HTR1E,HRH2,HTR2A
GO:MF	metal ion transmembrane transporter activity	GO:0046873	5.831E-05	4.2342565805062025	SLC6A2,KCNK12,SLC5A10,NIPAL2,KCNS2,KCNK13,SLC10A4,MCOLN3,KCNA1,KCNS1,KCNK3,KCNE4,TRPM8,SLC24A3,SLC6A11,KCNK4,ASIC2,CACNA1S,KCNA5,TRPV2,SLC5A1,KCNC2,SCN2B,SLC6A9,KCNK15,ATP1A3,SLC6A12,ATP2B3,NCS1,KCNIP1,HCN2
GO:MF	serotonin receptor activity	GO:0099589	8.0418E-05	4.094646883290234	HTR6,CHRM4,HRH1,HTR7,HTR5A,HTR1E,HRH2,HTR2A

GO:MF	monoatomic cation channel activity	GO:0005261	8.1132E-05	4.090806186923778	KCNK12,KCNS2,KCNK13,MCOLN3,KCNA1,KCNS1,OTOP2,KCNK3,KCNE4,TRPM8,SLC24A3,KCNK4,ASIC2,CACNA1S,KCNA5,TRPV2,CHRNA6,KCNC2,SCN2B,KCNK15,OTOP3,CHRNB2,ATP6V0C,NCS1,KCNIP1,HCN2
GO:MF	galanin receptor activity	GO:0004966	0.00017242	3.7634111948925466	GAL,GALR1,GALR2,GALR3
GO:MF	monoatomic ion gated channel activity	GO:0022839	0.00019778	3.7038157157744234	KCNK12,GABRA5,KCNS2,KCNK13,MCOLN3,KCNA1,GABRG3,KCNS1,KCNK3,KCNE4,TRPM8,KCNK4,ASIC2,CACNA1S,GABRE,KCNA5,CYBB,CHRNA6,KCNC2,SCN2B,CHRNB2,NCS1,KCNIP1,GABRQ,HCN2
GO:MF	gated channel activity	GO:0022836	0.00023461	3.6296606560753997	KCNK12,GABRA5,KCNS2,KCNK13,MCOLN3,KCNA1,GABRG3,KCNS1,KCNK3,KCNE4,TRPM8,KCNK4,ASIC2,CACNA1S,GABRE,KCNA5,CYBB,CHRNA6,KCNC2,SCN2B,CHRNB2,NCS1,KCNIP1,GABRQ,HCN2
GO:MF	potassium ion transmembrane transporter activity	GO:0015079	0.00082737	3.0823008865609776	KCNK12,KCNS2,KCNK13,KCNA1,KCNS1,KCNK3,KCNE4,SLC24A3,KCNK4,KCNA5,KCNC2,SCN2B,KCNK15,ATP1A3,KCNIP1,HCN2
GO:MF	potassium channel activity	GO:0005267	0.0008771	3.0569524398659227	KCNK12,KCNS2,KCNK13,KCNA1,KCNS1,KCNK3,KCNE4,KCNK4,KCNA5,KCNC2,SCN2B,KCNK15,KCNIP1,HCN2
GO:MF	catecholamine binding	GO:1901338	0.00449258	2.347504048848423	TH,ADRA2A,DRD5,DRD2,DRD1
GO:MF	dopamine binding	GO:0035240	0.00569763	2.2443059086273633	TH,DRD5,DRD2,DRD1
GO:MF	signaling receptor binding	GO:0005102	0.00656284	2.182907928858528	UTS2,CXCL8,GAL,GRP,FGF3,SYK,VGF,ADRA2A,NPFFR2,SYTL5,TG,PTHLH,GPNMB,GABRA5,KL,SRPX2,SERPINE1,BOK,CARTPT,APOF,ITGA11,OPRD1,IL32,BDNF,OSGIN1,HAP1,CCKBR,CCL2,EGFL6,CCL18,DRD2,S100B,SCG2,RTP1,NXPH3,CRP,P2RY2,NPW,RAET1G,CCL20,LY6H,VEGFB,COLEC10,KCNA5,RIMBP3,NPY,GBA,CCL5,ADM2,SQSTM1,MCHR1,NPPB,INHBA,SLC9A3R2,CHAC1,NENF,PITPNM1,REEP2,NPB,NXPH4,ADORA1,QRFP
GO:MF	hormone activity	GO:0005179	0.00663674	2.1780449873051038	UTS2,GAL,GRP,VGF,TG,PTHLH,KL,CARTPT,NPY,ADM2,NPPB,INHBA,QRFP
GO:MF	potassium ion leak channel activity	GO:0022841	0.014366417170096535	1.8426515267346528	KCNK12,KCNK13,KCNK3,KCNK4,KCNK15
GO:MF	voltage-gated monoatomic ion channel activity	GO:0005244	0.015285290627791142	1.8157262993751808	KCNK12,KCNS2,KCNK13,KCNA1,KCNS1,KCNK3,KCNE4,KCNK4,CACNA1S,KCNA5,CYBB,KCNC2,SCN2B,NCS1,KCNIP1,HCN2
GO:MF	signaling receptor regulator activity	GO:0030545	0.016852400156599653	1.773338237194142	UTS2,CXCL8,GAL,GRP,FGF3,VGF,TG,PTHLH,GPNMB,KL,CARTPT,IL32,BDNF,OSGIN1,CCL2,CCL18,SCG2,CCL20,LY6H,VEGFB,COLEC10,NPY,CCL5,ADM2,LYPD6B,NPPB,INHBA,LY6E,NENF,QRFP
GO:MF	voltage-gated channel activity	GO:0022832	0.017250873952098057	1.7631888981003214	KCNK12,KCNS2,KCNK13,KCNA1,KCNS1,KCNK3,KCNE4,KCNK4,CACNA1S,KCNA5,CYBB,KCNC2,SCN2B,NCS1,KCNIP1,HCN2
GO:MF	active monoatomic ion transmembrane transporter activity	GO:0022853	0.017976774980345583	1.745288217712159	SLC6A2,SLC18A3,SLC5A10,SLC10A4,SLC24A3,SLC6A11,SLC4A3,SLC5A1,SLC6A9,ATP1A3,ATP6V0B,SLC6A12,ATP2B3,ATP6V0C,ATP6V0D1,SLC36A1,ATP6V0A1,SLC45A1
GO:MF	hormone binding	GO:0042562	0.02300749	1.638130767482077	MC5R,RAMP1,GALR1,GALR2,CCKBR,GALR3,HCRTR1,MCHR1,INHBA,ATP1A3
GO:MF	neuropeptide activity	GO:0160041	0.028825065912703066	1.5402296909546684	GAL,GRP,VGF,CARTPT,NPY,QRFP
GO:MF	neuropeptide hormone activity	GO:0005184	0.028825065912703066	1.5402296909546684	GAL,GRP,VGF,CARTPT,NPY,QRFP
GO:MF	narrow pore channel activity	GO:0022842	0.03603821	1.4432367928787702	KCNK12,KCNK13,KCNK3,KCNK4,KCNK15

GO:MF	leak channel activity	GO:0022840	0.03603821	1.4432367928787702	KCNK12,KCNK13,KCNK3,KCNK4,KCNK15
GO:MF	active transmembrane transporter activity	GO:0022804	0.047997821692943594	1.3187784719619366	SLC6A2,SLC18A3,SLC5A10,SLC10A4,SLC6A17,SLC24A3,SLC6A11,SLC1A4,SLC4A3,SLC7A5,SLC5A1,SLC6A9,ABCG5,ATP1A3,ATP6V0B,SLC6A12,ATP2B3,ATP6V0C,SLC37A1,ABCA3,ATP6V0D1,SLC36A1,ATP6V0A1,SLC45A1,SLC22A3
GO:BP	cell-cell signaling	GO:0007267	6.727250107579414e-22	21.172162425695756	SLC6A2,UTS2,DBH,GAL,GRP,GPR50,FGF3,ADRA1B,SYK,VGF,TH,ADRA2A,EGR2,DRD5,BHLHA15,FFAR4,SLC18A3,PTHLH,HTR6,GPNMB,GABRA5,GATA4,GALR1,SOSTDC1,RBP4,EGR3,PHF24,TPBGL,EGR1,MAFA,PTPRN,CHRM4,SYT17,GRM7,HRH1,KCNA1,GABRG3,CITED1,CARTPT,ITPKA,NPBWR1,SV2C,GPR68,KCNK3,DYSF,HTR7,FOXL2,HAP1,SFRP5,CCL2,ADRA1D,CCL18,PLCG2,DRD2,SLC1A4,TACR1,S100B,GALR3,ASIC2,GJC2,HTR5A,NQO1,BEGAIN,MYRIP,CCL20,HCRTR1,RSPO4,LY6H,GABRE,KCNA5,MTNR1B,VAX2,TNFAIP3,CHRNA6,NPY,HTR1E,CCL5,SCN2B,SLC6A9,PRKAR1B,SYN1,SQSTM1,RASL10B,GJA10,INHBA,CHRNB2,ATP6V0C,HRH2,DRD1,DOC2B,HRAS,LRFN2,TNFRSF11A,LRRTM1,SYT5,PCSK1,SYT9,BAD,TMEM198,CYP46A1,PPARG,GABRQ,TPGS1,HTR2A,ADORA1,SNCB,AMER3,ACHE,HCN2,SYP
GO:BP	signaling	GO:0023052	1.3280415134145885e-18	17.876788349090674	SLC6A2,UTS2,DBH,CXCL8,GAL,GRP,GPR50,FGF3,PRLHR,ADRA1B,RELB,MC5R,SYK,VGF,TIMP4,OR2W3,TH,IL31RA,ARHGAP36,RAMP1,NKX2-2,ADRA2A,EGR2,TRIM58,DRD5,NPFFR2,SUSD5,BHLHA15,FFAR4,SLC18A3,TG,PTHLH,HTR6,GPNMB,RAMP3,FAM83G,MMP8,GABRA5,GATA4,GALR1,KL,CD28,IGFBP3,PTGER4,SOSTDC1,RBP4,EGR3,PHF24,TPBGL,PTGER2,NR0B1,EGR1,MAFA,PTPRN,CHRM4,UBD,SERPINE1,SYT17,NPSR1,GPR83,RASAL3,GRM7,HRH1,KCNA1,GABRG3,BOK,CITED1,CARTPT,HMOX1,ITPKA,NPBWR1,SV2C,GPR68,KCNK3,DYSF,ITGA11,GALR2,OPRD1,ADAP1,TNFRSF12A,HTR7,ADGRF4,IL32,BDNF,PALM3,CRLF2,TRPM8,FOXL2,OSGIN1,HAP1,CCKBR,SFRP5,CCL2,ADRA1D,IL1RL2,CCL18,PLCG2,DRD2,SLC1A4,OPRL1,IL15RA,GPR137,GSG1L,GZMB,CHRDL2,TACR1,S100B,SCG2,GALR3,PAK6,ASIC2,TRIB3,GJC2,SPHK1,STK32B,NRGN,NXPH3,DCLK3,P2RY2,NTSR2,MAOA,HTR5A,NQO1,NPW,BEGAIN,MYRIP,PDE2A,NGEF,CCL20,SPRED3,SKAP1,HCRTR1,ALDH1A2,RSPO4,RCAN1,OR2L13,LY6H,VEGFB,COLEC10,PLEKHF1,PHLDA2,GABRE,NUPR1,ARHGAP22,KIAA0319,KCNA5,MTNR1B,CSRNP1,SSTR4,CYBB,VAX2,TNFAIP3,CHRNA6,DUSP26,KLF2,KCNC2,NPY,GBA,MGLL,HTR1E,CTSD,ADAMTSL2,CCL5,SCN2B,ADM2,TSSK1B,SLC6A9,RFX8,PSEN2,PRKAR1B,RHBDD2,SYN1,SQSTM1,GPR45,UBASH3A,RIN3,RASL10B,IL7R,GJA10,MCHR1,DUSP5,NPPB,OASL,CALY,MAPK8IP1,INHBA,ABCG5,NFAM1,CHAC1,KRT18,BEND6,LY6E,CHRNB2,PALM2,ATP6V0C,HRH2,NENF,STK32C,DACT2,PITPNM1,DRD1,DOC2B,RASGEF1C,MS4A2,HRAS,LRFN2,ADGRA1,TNFRSF11A,LRRTM1,SYT5,PCSK1,SYT9,NPB,PLA2G4C,BAD,TMEM198,IRAK1,NXPH4,FIBP,CYP46A1,PPARG,ALK,GABRQ,TPGS1,HTR2A,RASGEF1A,LIMS2,ADORA1,RGS6,IRAK2,TEC,RET,SNCB,AMER3,TICAM1,IRF4,ACHE,HCN2,QRFP,SYP

GO:BP	multicellular organismal process	GO:0032501	1.6992201700797068e-18	17.769750345406578	SLC6A2,UTS2,DBH,KRT80,CXCL8,FOLR3,GAL,GRP,FGF3,PRLHR,ADRA1B,NKX6-2,RELB,SYK,TNFRSF9,VGF,MYH2,OR2W3,TH,IL31RA,RAMP1,NKX2-2,KRT75,TMIE,PHOX2A,ADRA2A,EGR2,TRIM58,DRD5,OSR2,BHLHA15,FFAR4,TG,PTHLH,HTR6,GPNMB,RAMP3,MMP8,GABRA5,GATA4,DRGX,GALR1,KL,MMP19,CD28,COL8A1,IGFBP3,SRPX2,PTGER4,SOSTDC1,RBP4,EGR3,PHF24,NR0B1,EGR1,PTPRN,BCAN,TMEM119,SIX2,UBD,SERPINE1,SYT17,MSC,SERPINA3,NPSR1,PAX1,C1QL2,MCOLN3,GPR83,SLC6A17,RASAL3,AQP5,TMEM132E,GRM7,HRH1,KCNA1,GABRG3,BOK,CITED1,CARTPT,CNN1,HMOX1,ITPKA,GPR68,MALL,DYSF,ITGA11,KCNE4,GALR2,OPRD1,HOXD9,TNFRSF12A,HTR7,GCH1,IL32,BDNF,CRLF2,TMEM229B,TRPM8,FOXL2,FOXE1,SLC24A3,HAP1,ASCL2,CCKBR,SFRP5,KRT86,CCL2,ADRA1D,IL1RL2,ETS1,PLCG2,DRD2,SLC1A4,OPRL1,IL15RA,IRX6,GPR137,SLC4A3,TBX21,CHRDL2,TACR1,KCNK4,ACTG2,S100B,SCG2,GALR3,PAK6,ASIC2,GJC2,SPHK1,RTP1,MYH7,NRGN,HSPB7,OOSP2,CRP,P2RY2,TLL2,NTSR2,HTR5A,NPW,BEGAIN,BFSP1,PDE2A,HOXC13,RAET1G,NGEF,SPRED3,CACNA1S,HCRTR1,CABP1,ALDH1A2,RSPO4,RCAN1,OR2L13,LY6H,VEGFB,TAL1,BATF3,PHLDA2,S100A2,GABRE,NUPR1,ARHGAP22,KIAA0319,KCNA5,MTNR1B,CSRNP1,SSTR4,CYBB,RIMBP3,SLC7A5,VAX2,KIRREL3,TRPV2,TNFAIP3,ISLR2,SPOCD1,CHRNA6,SLC5A1,TMPRSS3,KLF2,KCNC2,NPY,ETNK2,GBA,PEMT,MGLL,DGAT2,MIP,ADAMTSL2,CCL5,SCN2B,ADM2,TSSK1B,SLC6A9,RFX8,PRKAR1B,SYN1,SQSTM1,UBASH3A,SPRR2G,RASL10B,IL7R,GJA10,MCHR1,HM13,DUSP5,NPPB,MAPK8IP1,INHBA,ZNF358,ABCG5,NFAM1,CHAC1,BTBD6,ATP1A3,GAMT,BEND6,CHRNB2,ATP2B3,CYP4A11,HRH2,ASTN2,CABLES1,DACT2,PITPNM1,DRD1,TCTA,HRAS,REEP2,TNFRSF11A,CDK5R2,LRRTM1,PCSK1,NPB,CYGB,PLA2G4C,BAD,NCS1,COL8A2,MAL2,IRAK1,CDHR1,ABCA3,FIBP,CYP46A1,PPARG,HPCAL4,ALK,ATP8A2,DMRT2,USH1G,GABRQ,HOXD11,TPGS1,HTR2A,LIMS2,ADORA1,TEC,RET,TICAM1,IRF4,FBLL1,ACHE,HCN2,QRFP,TMEM120A,SLC22A3
GO:BP	cell communication	GO:0007154	2.02093623454115e-18	17.69444738931686	SLC6A2,UTS2,DBH,CXCL8,GAL,GRP,GPR50,FGF3,PRLHR,ADRA1B,RELB,MC5R,SYK,VGF,TIMP4,OR2W3,TH,IL31RA,ARHGAP36,RAMP1,NKX2-2,ADRA2A,EGR2,TRIM58,DRD5,NPFFR2,SUSD5,BHLHA15,FFAR4,SLC18A3,TG,PTHLH,HTR6,GPNMB,RAMP3,FAM83G,MMP8,GABRA5,GATA4,GALR1,KL,CD28,IGFBP3,PTGER4,SOSTDC1,RBP4,EGR3,PHF24,TPBGL,PTGER2,NR0B1,EGR1,MAFA,PTPRN,CHRM4,UBD,SERPINE1,SYT17,NPSR1,GPR83,RASAL3,GRM7,HRH1,KCNA1,GABRG3,BOK,CITED1,CARTPT,HMOX1,ITPKA,NPBWR1,SV2C,GPR68,KCNK3,DYSF,ITGA11,GALR2,OPRD1,ADAP1,TNFRSF12A,HTR7,ADGRF4,IL32,BDNF,PALM3,CRLF2,TRPM8,FOXL2,OSGIN1,HAP1,CCKBR,SFRP5,CCL2,ADRA1D,IL1RL2,CCL18,PLCG2,DRD2,SLC1A4,OPRL1,IL15RA,GPR137,GSG1L,GZMB,CHRDL2,TACR1,S100B,SCG2,GALR3,PAK6,ASIC2,TRIB3,GJC2,SPHK1,STK32B,NRGN,NXPH3,DCLK3,P2RY2,NTSR2,MAOA,HTR5A,NQO1,NPW,BEGAIN,MYRIP,PDE2A,NGEF,CCL20,SPRED3,SKAP1,HCRTR1,ALDH1A2,RSPO4,RCAN1,OR2L13,LY6H,VEGFB,COLEC10,PLEKHF1,PHLDA2,GABRE,NUPR1,ARHGAP22,KIAA0319,KCNA5,MTNR1B,CSRNP1,SSTR4,CYBB,SLC7A5,VAX2,TNFAIP3,CHRNA6,DUSP26,KLF2,KCNC2,NPY,GBA,MGLL,HTR1E,CTSD,ADAMTSL2,CCL5,SCN2B,ADM2,TSSK1B,SLC6A9,RFX8,PSEN2,PRKAR1B,RHBDD2,SYN1,SQSTM1,GPR45,UBASH3A,RIN3,RASL10B,IL7R,GJA10,MCHR1,DUSP5,NPPB,OASL,CALY,MAPK8IP1,INHBA,ABCG5,NFAM1,CHAC1,ATP1A3,KRT18,BEND6,LY6E,CHRNB2,PALM2,ATP6V0C,HRH2,NENF,STK32C,DACT2,PITPNM1,DRD1,DOC2B,RASGEF1C,MS4A2,HRAS,LRFN2,ADGRA1,TNFRSF11A,LRRTM1,SYT5,PCSK1,SYT9,NPB,PLA2G4C,BAD,TMEM198,IRAK1,NXPH4,FIBP,CYP46A1,PPARG,ALK,GABRQ,TPGS1,HTR2A,RASGEF1A,LIMS2,ADORA1,RGS6,IRAK2,TEC,RET,SNCB,AMER3,TICAM1,IRF4,ACHE,HCN2,QRFP,SYP
GO:BP	chemical synaptic transmission	GO:0007268	2.4009595380780866e-17	16.61961515878407	SLC6A2,UTS2,DBH,VGF,TH,ADRA2A,EGR2,DRD5,SLC18A3,HTR6,GABRA5,EGR3,PHF24,CHRM4,GRM7,HRH1,KCNA1,GABRG3,CARTPT,ITPKA,NPBWR1,SV2C,KCNK3,DYSF,HTR7,HAP1,CCL2,DRD2,SLC1A4,TACR1,S100B,GALR3,ASIC2,HTR5A,NQO1,BEGAIN,HCRTR1,GABRE,MTNR1B,CHRNA6,NPY,HTR1E,SCN2B,SLC6A9,PRKAR1B,SYN1,SQSTM1,CHRNB2,HRH2,DRD1,DOC2B,HRAS,LRFN2,LRRTM1,SYT5,SYT9,CYP46A1,GABRQ,TPGS1,HTR2A,ADORA1,SNCB,ACHE,SYP
GO:BP	anterograde trans-synaptic signaling	GO:0098916	2.4009595380780866e-17	16.61961515878407	SLC6A2,UTS2,DBH,VGF,TH,ADRA2A,EGR2,DRD5,SLC18A3,HTR6,GABRA5,EGR3,PHF24,CHRM4,GRM7,HRH1,KCNA1,GABRG3,CARTPT,ITPKA,NPBWR1,SV2C,KCNK3,DYSF,HTR7,HAP1,CCL2,DRD2,SLC1A4,TACR1,S100B,GALR3,ASIC2,HTR5A,NQO1,BEGAIN,HCRTR1,GABRE,MTNR1B,CHRNA6,NPY,HTR1E,SCN2B,SLC6A9,PRKAR1B,SYN1,SQSTM1,CHRNB2,HRH2,DRD1,DOC2B,HRAS,LRFN2,LRRTM1,SYT5,SYT9,CYP46A1,GABRQ,TPGS1,HTR2A,ADORA1,SNCB,ACHE,SYP

GO:BP	trans-synaptic signaling	GO:0099537	3.644240526143691e-17	16.43839296657016	SLC6A2,UTS2,DBH,VGF,TH,ADRA2A,EGR2,DRD5,SLC18A3,HTR6,GABRA5,EGR3,PHF24,CHRM4,GRM7,HRH1,KCNA1,GABRG3,CARTPT,ITPKA,NPBWR1,SV2C,KCNK3,DYSF,HTR7,HAP1,CCL2,DRD2,SLC1A4,TACR1,S100B,GALR3,ASIC2,HTR5A,NQO1,BEGAIN,HCRTR1,GABRE,MTNR1B,CHRNA6,NPY,HTR1E,SCN2B,SLC6A9,PRKAR1B,SYN1,SQSTM1,CHRNB2,HRH2,DRD1,DOC2B,HRAS,LRFN2,LRRTM1,SYT5,SYT9,CYP46A1,GABRQ,TPGS1,HTR2A,ADORA1,SNCB,ACHE,SYP
GO:BP	synaptic signaling	GO:0099536	4.3695395514370027e-17	16.359564325227716	SLC6A2,UTS2,DBH,VGF,TH,ADRA2A,EGR2,DRD5,SLC18A3,HTR6,GABRA5,EGR3,PHF24,CHRM4,GRM7,HRH1,KCNA1,GABRG3,CARTPT,ITPKA,NPBWR1,SV2C,KCNK3,DYSF,HTR7,HAP1,CCL2,DRD2,SLC1A4,TACR1,S100B,GALR3,ASIC2,HTR5A,NQO1,BEGAIN,HCRTR1,LY6H,GABRE,MTNR1B,CHRNA6,NPY,HTR1E,SCN2B,SLC6A9,PRKAR1B,SYN1,SQSTM1,CHRNB2,HRH2,DRD1,DOC2B,HRAS,LRFN2,LRRTM1,SYT5,SYT9,CYP46A1,GABRQ,TPGS1,HTR2A,ADORA1,SNCB,ACHE,SYP
GO:BP	regulation of biological quality	GO:0065008	9.299890990578998e-16	15.031522142033966	SLC6A2,UTS2,DBH,GAL,GRP,PRLHR,ADRA1B,SYK,VGF,ADRA2A,EGR2,DRD5,FFAR4,SLC18A3,TG,KCNK12,ATP10A,GABRA5,GALR1,KL,SRPX2,PTGER4,RBP4,PTGER2,EGR1,MAFA,PTPRN,SERPINE1,KCNK13,C1QL2,AQP5,HRH1,KCNA1,GABRG3,BOK,CARTPT,ITPKA,SV2C,GPR68,KCNK3,DYSF,KCNE4,GALR2,OPRD1,HTR7,GCH1,BDNF,PALM3,SPTB,FOXL2,FOXE1,CCKBR,CCL2,ADRA1D,SLC6A11,PLCG2,DRD2,OPRL1,SLC4A3,GSG1L,GZMB,TACR1,KCNK4,S100B,ASIC2,MYH7,CRP,P2RY2,NTSR2,MYRIP,PDE2A,NGEF,ALDH1A2,GABRE,ARHGAP22,KIAA0319,KCNA5,MTNR1B,SLC7A5,TRPV2,ISLR2,CHRNA6,KLF2,KCNC2,AANAT,NPY,GBA,DGAT2,CCL5,SCN2B,ADM2,PRKAR1B,KCNK15,SYN1,SQSTM1,RIN3,AKR1B10,RASL10B,IL7R,MCHR1,NPPB,CALY,INHBA,ATP1A3,ATP6V0B,SLC6A12,CHRNB2,PALM2,ATP6V0C,CYP4A11,HRH2,NENF,DRD1,DOC2B,HRAS,LRFN2,LRRTM1,SYT5,PCSK1,SYT9,BAD,ABCA3,ATP6V0D1,FIBP,CYP46A1,PPARG,ATP8A2,ATP6V0A1,GABRQ,HTR2A,ADORA1,TEC,RET,DHCR7,HCN2,QRFP,SLC22A3,SYP
GO:BP	regulation of transport	GO:0051049	1.9557761964987516e-15	14.708680843917852	GAL,GRP,SYK,ADRA2A,TRIM58,FFAR4,SLC18A3,RAMP3,KCNK12,GALR1,RBP4,REM1,LGI3,PTPRN,KCNS2,SERPINE1,SYT17,KCNK13,NPSR1,GRM7,KCNA1,KCNS1,BOK,CARTPT,SV2C,GPR68,KCNK3,DYSF,KCNE4,GALR2,OPRD1,FOXL2,HAP1,CCL2,PLCG2,DRD2,OPRL1,IL15RA,GSG1L,GZMB,TACR1,KCNK4,ASIC2,TRIB3,GJC2,SPHK1,P2RY2,MYRIP,CACNA1S,CABP1,COLEC10,GABRE,KCNA5,MTNR1B,SSTR4,CYBB,SLC7A5,TRPV2,CHRNA6,KCNC2,CCL5,SCN2B,SLC6A9,PSEN2,PRKAR1B,KCNK15,SYN1,RIN3,RASL10B,MCHR1,NPPB,CALY,INHBA,ABCG5,MLC1,CHRNB2,CYP4A11,DRD1,DOC2B,HRAS,REEP2,TNFRSF11A,CDK5R2,LRRTM1,SYT5,PCSK1,SYT9,BAD,KCNIP1,ABCA3,PPARG,ATP8A2,USH1G,HTR2A,ADORA1,PARD6A,ACHE,ABLIM3,HCN2,SYP
GO:BP	neuropeptide signaling pathway	GO:0007218	4.605198162809919e-14	13.33675167726665	GAL,GRP,PRLHR,NPFFR2,GALR1,NPSR1,GPR83,CARTPT,NPBWR1,GALR2,OPRD1,OPRL1,GALR3,NXPH3,NTSR2,NPW,HCRTR1,SSTR4,NPY,MCHR1,NPPB,NPB,NXPH4,QRFP
GO:BP	regulation of localization	GO:0032879	1.6114524668105162e-13	12.7927825	GAL,GRP,SYK,ADRA2A,TRIM58,FFAR4,SLC18A3,RAMP3,KCNK12,GALR1,RBP4,REM1,LGI3,PTPRN,KCNS2,SERPINE1,SYT17,KCNK13,NPSR1,MCOLN3,GRM7,KCNA1,KCNS1,BOK,CARTPT,SV2C,GPR68,KCNK3,DYSF,KCNE4,GALR2,OPRD1,BDNF,FOXL2,HAP1,CCL2,PLCG2,DRD2,OPRL1,IL15RA,GSG1L,GZMB,TACR1,KCNK4,ASIC2,TRIB3,GJC2,SPHK1,CRP,DCLK3,P2RY2,TRIM29,MYRIP,CACNA1S,CABP1,COLEC10,GABRE,KCNA5,MTNR1B,SSTR4,CYBB,SLC7A5,TRPV2,CHRNA6,KCNC2,CTSD,CCL5,SCN2B,SLC6A9,PSEN2,PRKAR1B,KCNK15,SYN1,SQSTM1,RIN3,RASL10B,MCHR1,NPPB,CALY,INHBA,ABCG5,MLC1,CHRNB2,CYP4A11,ASTN2,DRD1,DOC2B,HRAS,REEP2,TNFRSF11A,CDK5R2,LRRTM1,SYT5,PCSK1,SYT9,BAD,KCNIP1,ABCA3,PPARG,ATP8A2,USH1G,HTR2A,ADORA1,PARD6A,ACHE,ABLIM3,HCN2,SYP
GO:BP	circulatory system process	GO:0003013	1.8246672574826467e-13	12.738816321023666	UTS2,DBH,ADRA1B,TH,ADRA2A,DRD5,RAMP3,GATA4,KL,SLC6A17,HRH1,CARTPT,KCNE4,HTR7,GCH1,SLC24A3,ADRA1D,DRD2,SLC1A4,OPRL1,SLC4A3,TACR1,ASIC2,MYH7,HSPB7,CRP,P2RY2,PDE2A,VEGFB,KCNA5,MTNR1B,SLC7A5,SLC5A1,KLF2,NPY,SCN2B,ADM2,SLC6A9,RASL10B,NPPB,ATP1A3,ATP2B3,CYP4A11,HRH2,DRD1,PPARG,HTR2A,ADORA1,HCN2,QRFP,SLC22A3
GO:BP	monoatomic ion transport	GO:0006811	7.8868924862257e-13	12.103094079417053	SLC6A2,GAL,GRP,RAMP1,ADRA2A,BHLHA15,SLC18A3,TG,RAMP3,KCNK12,ATP10A,GABRA5,SLC5A10,NIPAL2,REM1,KCNS2,KCNK13,NPSR1,SLC10A4,MCOLN3,SLC6A17,KCNA1,GABRG3,KCNS1,OTOP2,KCNK3,KCNE4,GALR2,OPRD1,TRPM8,SLC24A3,HAP1,CCL2,SLC6A11,PLCG2,DRD2,SLC1A4,OPRL1,SLC4A3,KCNK4,ASIC2,GJC2,CACNA1S,CABP1,GABRE,KCNA5,MTNR1B,CYBB,TRPV2,CHRNA6,SLC5A1,KCNC2,CCL5,SCN2B,SLC6A9,PSEN2,KCNK15,OTOP3,MCHR1,ATP1A3,ATP6V0B,MLC1,SLC6A12,CHRNB2,ATP2B3,ATP6V0C,DRD1,NCS1,KCNIP1,ATP6V0D1,SLC36A1,ATP6V0A1,GABRQ,HTR2A,ADORA1,HCN2,TMEM120A,SLC22A3

GO:BP	monoatomic ion transmembrane transport	GO:0034220	1.139254654814849e-12	11.943379188283128	SLC6A2,GAL,GRP,ADRA2A,BHLHA15,SLC18A3,RAMP3,KCNK12,ATP10A,GABRA5,SLC5A10,NIPAL2,REM1,KCNS2,KCNK13,NPSR1,MCOLN3,SLC6A17,KCNA1,GABRG3,KCNS1,OTOP2,KCNK3,KCNE4,GALR2,TRPM8,SLC24A3,HAP1,CCL2,SLC6A11,PLCG2,DRD2,SLC1A4,OPRL1,SLC4A3,KCNK4,ASIC2,GJC2,CACNA1S,CABP1,GABRE,KCNA5,MTNR1B,CYBB,TRPV2,CHRNA6,SLC5A1,KCNC2,SCN2B,SLC6A9,PSEN2,KCNK15,OTOP3,ATP1A3,ATP6V0B,SLC6A12,CHRNB2,ATP2B3,ATP6V0C,DRD1,NCS1,KCNIP1,ATP6V0D1,SLC36A1,ATP6V0A1,GABRQ,HTR2A,HCN2,TMEM120A
GO:BP	response to stimulus	GO:0050896	2.3803216367282343e-12	11.623364355708834	SLC6A2,UTS2,DBH,CXCL8,GAL,GRP,GPR50,MMP1,FGF3,PRLHR,ADRA1B,RELB,MC5R,SYK,VGF,TIMP4,NEURL3,OR2W3,TH,IL31RA,ARHGAP36,RAMP1,NGB,NKX2-2,ADRA2A,EGR2,TRIM58,DRD5,NPFFR2,SUSD5,BHLHA15,FFAR4,TG,PTHLH,HTR6,UGT3A2,GPNMB,RAMP3,FAM83G,MMP8,GABRA5,GATA4,DRGX,GALR1,KL,DMBT1,MMP19,CD28,IGFBP3,PTGER4,SOSTDC1,RBP4,EGR3,PHF24,TPBGL,PTGER2,NR0B1,EGR1,ME1,MAFA,PTPRN,CHRM4,UBD,SERPINE1,SYT17,BPIFC,MSC,SERPINA3,NPSR1,GPR83,RASAL3,AQP5,CD300C,GRM7,HRH1,KCNA1,GABRG3,BOK,CITED1,CARTPT,HMOX1,ITPKA,NPBWR1,GPR68,KCNK3,DYSF,ITGA11,GALR2,OPRD1,ADAP1,HAS1,TNFRSF12A,HTR7,GCH1,ADGRF4,IL32,BDNF,PALM3,CRLF2,TMEM229B,TRPM8,OSGIN1,HAP1,ASCL2,CCKBR,SFRP5,FCMR,CCL2,ADRA1D,IL1RL2,ETS1,SLC6A11,CCL18,PLCG2,DRD2,OPRL1,IL15RA,GPR137,TBX21,GSG1L,GZMB,CHRDL2,TACR1,KCNK4,S100B,SCG2,GALR3,PAK6,ASIC2,TRIB3,GJC2,SPHK1,RTP1,MYH7,STK32B,NRGN,EPHX1,HSPB7,NXPH3,CRP,DCLK3,P2RY2,TRIM29,NTSR2,MAOA,HTR5A,NQO1,NPW,BEGAIN,PDE2A,RAET1G,SRXN1,NGEF,CCL20,SPRED3,SKAP1,CACNA1S,HCRTR1,CABP1,ALDH1A2,RSPO4,RCAN1,OR2L13,LY6H,VEGFB,COLEC10,PLEKHF1,BATF3,PHLDA2,GABRE,NUPR1,ARHGAP22,KIAA0319,KCNA5,MTNR1B,CSRNP1,SSTR4,CYBB,SLC7A5,VAX2,TRPV2,TNFAIP3,CHRNA6,SLC5A1,DUSP26,KLF2,KCNC2,AANAT,NPY,GBA,MGLL,HTR1E,DGAT2,CTSD,MIP,ADAMTSL2,CCL5,SCN2B,ADM2,TSSK1B,SLC6A9,TXNRD2,RFX8,PSEN2,PRKAR1B,RHBDD2,SQSTM1,GPR45,UBASH3A,RIN3,AKR1B10,IL7R,GJA10,MCHR1,HM13,DUSP5,NPPB,OASL,CALY,MAPK8IP1,INHBA,ABCG5,NFAM1,CHAC1,ATP1A3,MLC1,KRT18,BEND6,LY6E,CHRNB2,PALM2,ATP6V0C,HRH2,NENF,GPX3,STK32C,LAMP3,DACT2,PITPNM1,DRD1,RASGEF1C,MS4A2,HRAS,ADGRA1,TNFRSF11A,SYT5,PCSK1,SYT9,NPB,CYGB,PLA2G4C,BAD,TMEM198,IRAK1,ABCA3,ATP6V0D1,NXPH4,FIBP,CYP46A1,PPARG,ALK,ATP8A2,GABRQ,HTR2A,RASGEF1A,LIMS2,ADORA1,RGS6,IRAK2,TMEFF2,CPNE7,TEC,RET,AMER3,TICAM1,IRF4,ACHE,HCN2,QRFP,TMEM120A,SLC22A3,SYP,HIST1H2BK
GO:BP	transmembrane transport	GO:0055085	3.48458093914844e-12	11.457849443321644	SLC6A2,GAL,GRP,ADRA2A,BHLHA15,FFAR4,SLC18A3,RAMP3,KCNK12,ATP10A,GABRA5,SLC5A10,NIPAL2,REM1,KCNS2,KCNK13,NPSR1,SLC10A4,MCOLN3,SLC6A17,AQP5,KCNA1,GABRG3,KCNS1,SLC7A4,OTOP2,SV2C,KCNK3,KCNE4,GALR2,TRPM8,SLC24A3,HAP1,CCL2,SLC6A11,PLCG2,SLC2A6,DRD2,SLC1A4,OPRL1,SLC4A3,GSG1L,KCNK4,ASIC2,TRIB3,GJC2,CACNA1S,CABP1,GABRE,KCNA5,MTNR1B,CYBB,SLC7A5,TRPV2,CHRNA6,SLC5A1,KCNC2,MIP,SCN2B,SLC6A9,PSEN2,KCNK15,OTOP3,GJA10,ABCG5,ATP1A3,ATP6V0B,SLC6A12,CHRNB2,ATP2B3,SLC35F4,ATP6V0C,DRD1,SLC37A1,NCS1,KCNIP1,ABCA3,ATP6V0D1,SLC36A1,ATP6V0A1,GABRQ,HTR2A,SLC45A1,HCN2,TMEM120A,SLC22A3,PQLC2
GO:BP	inorganic ion transmembrane transport	GO:0098660	3.720365100998978e-12	11.429414438191042	SLC6A2,GAL,GRP,ADRA2A,BHLHA15,SLC18A3,RAMP3,KCNK12,GABRA5,SLC5A10,NIPAL2,REM1,KCNS2,KCNK13,NPSR1,MCOLN3,SLC6A17,KCNA1,GABRG3,KCNS1,OTOP2,KCNK3,KCNE4,GALR2,TRPM8,SLC24A3,HAP1,SLC6A11,PLCG2,DRD2,SLC1A4,OPRL1,SLC4A3,KCNK4,ASIC2,GJC2,CACNA1S,CABP1,GABRE,KCNA5,MTNR1B,TRPV2,SLC5A1,KCNC2,SCN2B,SLC6A9,PSEN2,KCNK15,OTOP3,ATP1A3,ATP6V0B,SLC6A12,ATP2B3,ATP6V0C,DRD1,SLC37A1,NCS1,KCNIP1,ATP6V0D1,SLC36A1,ATP6V0A1,GABRQ,HTR2A,HCN2
GO:BP	system process	GO:0003008	5.6588711851941084e-12	11.247270191944883	UTS2,DBH,GAL,ADRA1B,NKX6-2,MYH2,OR2W3,TH,TMIE,ADRA2A,EGR2,DRD5,FFAR4,RAMP3,GABRA5,GATA4,DRGX,GALR1,KL,RBP4,PHF24,SERPINA3,NPSR1,SLC6A17,AQP5,GRM7,HRH1,KCNA1,GABRG3,CARTPT,CNN1,HMOX1,KCNE4,GALR2,HTR7,GCH1,BDNF,TRPM8,FOXL2,SLC24A3,CCKBR,SFRP5,CCL2,ADRA1D,DRD2,SLC1A4,OPRL1,SLC4A3,TACR1,KCNK4,S100B,GALR3,PAK6,ASIC2,SPHK1,RTP1,MYH7,HSPB7,CRP,P2RY2,NTSR2,BEGAIN,PDE2A,CACNA1S,CABP1,RCAN1,OR2L13,VEGFB,GABRE,KCNA5,MTNR1B,SLC7A5,VAX2,TRPV2,CHRNA6,SLC5A1,TMPRSS3,KLF2,NPY,GBA,MGLL,MIP,SCN2B,ADM2,SLC6A9,PRKAR1B,RASL10B,GJA10,NPPB,INHBA,ABCG5,ATP1A3,GAMT,CHRNB2,ATP2B3,CYP4A11,HRH2,DRD1,REEP2,PPARG,ATP8A2,USH1G,GABRQ,HTR2A,ADORA1,HCN2,QRFP,TMEM120A,SLC22A3

GO:BP	signal transduction	GO:0007165	6.352555749730625e-12	11.197051514912912	UTS2,DBH,CXCL8,GAL,GRP,GPR50,FGF3,PRLHR,ADRA1B,RELB,MC5R,SYK,VGF,TIMP4,OR2W3,IL31RA,ARHGAP36,RAMP1,NKX2-2,ADRA2A,TRIM58,DRD5,NPFFR2,SUSD5,BHLHA15,FFAR4,TG,PTHLH,HTR6,GPNMB,RAMP3,FAM83G,MMP8,GABRA5,GATA4,GALR1,KL,CD28,IGFBP3,PTGER4,SOSTDC1,PHF24,TPBGL,PTGER2,NR0B1,EGR1,MAFA,CHRM4,UBD,SERPINE1,NPSR1,GPR83,RASAL3,GRM7,HRH1,KCNA1,GABRG3,BOK,CITED1,CARTPT,HMOX1,ITPKA,NPBWR1,GPR68,ITGA11,GALR2,OPRD1,ADAP1,TNFRSF12A,HTR7,ADGRF4,IL32,BDNF,PALM3,CRLF2,TRPM8,OSGIN1,HAP1,CCKBR,SFRP5,CCL2,ADRA1D,IL1RL2,CCL18,PLCG2,DRD2,OPRL1,IL15RA,GPR137,GSG1L,GZMB,CHRDL2,TACR1,S100B,SCG2,GALR3,PAK6,ASIC2,TRIB3,SPHK1,STK32B,NRGN,NXPH3,DCLK3,P2RY2,NTSR2,MAOA,HTR5A,NPW,BEGAIN,PDE2A,NGEF,CCL20,SPRED3,SKAP1,HCRTR1,ALDH1A2,RSPO4,RCAN1,OR2L13,LY6H,VEGFB,COLEC10,PLEKHF1,PHLDA2,GABRE,NUPR1,ARHGAP22,KIAA0319,KCNA5,MTNR1B,CSRNP1,SSTR4,CYBB,VAX2,TNFAIP3,CHRNA6,DUSP26,KLF2,KCNC2,NPY,GBA,MGLL,HTR1E,CTSD,ADAMTSL2,CCL5,ADM2,TSSK1B,SLC6A9,RFX8,PSEN2,PRKAR1B,RHBDD2,SQSTM1,GPR45,UBASH3A,RIN3,IL7R,MCHR1,DUSP5,NPPB,OASL,CALY,MAPK8IP1,INHBA,ABCG5,NFAM1,CHAC1,KRT18,BEND6,LY6E,CHRNB2,PALM2,ATP6V0C,HRH2,NENF,STK32C,DACT2,PITPNM1,DRD1,RASGEF1C,MS4A2,HRAS,ADGRA1,TNFRSF11A,NPB,PLA2G4C,BAD,TMEM198,IRAK1,NXPH4,FIBP,PPARG,ALK,GABRQ,HTR2A,RASGEF1A,LIMS2,ADORA1,RGS6,IRAK2,TEC,RET,AMER3,TICAM1,IRF4,ACHE,QRFP,SYP
GO:BP	transport	GO:0006810	1.2394655134565195e-11	10.906765552416848	SLC6A2,CXCL8,FOLR3,GAL,GRP,SYK,VGF,NEURL3,TH,RAMP1,NGB,ADRA2A,EGR2,TRIM58,BHLHA15,SYTL5,FFAR4,SLC18A3,TG,RAMP3,KCNK12,ATP10A,APOL6,GABRA5,GALR1,DMBT1,SLC5A10,NIPAL2,RBP4,REM1,LGI3,MAFA,PTPRN,GOLT1A,KCNS2,SIX2,SERPINE1,SYT17,KCNK13,NPSR1,SLC10A4,MCOLN3,SLC6A17,AQP5,GRM7,KCNA1,GABRG3,KCNS1,SLC7A4,BOK,CITED1,CARTPT,OTOP2,SV2C,GPR68,APOF,KCNK3,DYSF,KCNE4,GALR2,OPRD1,B4GALT1-AS1,TRPM8,FOXL2,SLC24A3,HAP1,CCKBR,CCL2,SLC6A11,PLCG2,SLC2A6,DRD2,SLC1A4,OPRL1,IL15RA,RAB3IL1,SLC4A3,GSG1L,GZMB,TACR1,KCNK4,SCG2,ASIC2,TRIB3,GJC2,SPHK1,RTP1,CRP,P2RY2,MYRIP,CACNA1S,CABP1,COLEC10,PLEKHF1,GABRE,KCNA5,MTNR1B,SSTR4,CYBB,SLC7A5,TRPV2,CHRNA6,SLC5A1,TMPRSS3,KCNC2,MIP,CCL5,SCN2B,SLC6A9,PSEN2,PRKAR1B,KCNK15,SYN1,SQSTM1,RIN3,OTOP3,RASL10B,GJA10,MCHR1,HM13,NPPB,CALY,MAPK8IP1,INHBA,ABCG5,ATP1A3,ATP6V0B,MLC1,KRT18,SLC6A12,CHRNB2,ATP2B3,SLC35F4,ATP6V0C,CYP4A11,HRH2,ASTN2,TEX261,PITPNM1,DRD1,DOC2B,HRAS,REEP2,SLC37A1,SPRN,TNFRSF11A,CDK5R2,LRRTM1,SYT5,PCSK1,SYT9,CYGB,BAD,NCS1,KCNIP1,KIF17,ABCA3,ATP6V0D1,PPARG,ATP8A2,DYNC1I1,USH1G,SLC36A1,ATP6V0A1,GABRQ,ZDHHC14,HTR2A,ADORA1,SLC45A1,SNCB,PARD6A,ACHE,ABLIM3,HCN2,TMEM120A,SLC22A3,SYP,PQLC2
GO:BP	anatomical structure development	GO:0048856	1.836922735187396e-11	10.73590911064436	KRT80,CXCL8,GAL,FGF3,NKX6-2,RELB,SYK,TNFRSF9,VGF,TH,IL31RA,RAMP1,NKX2-2,KRT75,TMIE,PHOX2A,EGR2,TRIM58,OSR2,BHLHA15,TG,PTHLH,HTR6,GPNMB,ATP10A,MMP8,GABRA5,GATA4,DRGX,KL,DMBT1,MMP19,CD28,COL8A1,IGFBP3,SRPX2,PTGER4,SOSTDC1,RBP4,EGR3,NR0B1,EGR1,PTPRN,BCAN,TMEM119,SIX2,UBD,SERPINE1,SYT17,MSC,PAX1,C1QL2,MCOLN3,SLC6A17,AQP5,TMEM132E,GRM7,KCNA1,BOK,CITED1,CARTPT,GSC2,HMOX1,ITPKA,GPR68,MALL,KCNK3,ITGA11,GALR2,HOXD9,TNFRSF12A,BDNF,KRT15,PALM3,FOXL2,FOXE1,SLC24A3,HAP1,ASCL2,CCKBR,SFRP5,KRT86,CCL2,IL1RL2,ETS1,PLCG2,DRD2,IL15RA,IRX6,GPR137,TBX21,CHRDL2,SHROOM1,ACTG2,S100B,SCG2,PAK6,ASIC2,GJC2,SPHK1,MYH7,NRGN,HSPB7,OOSP2,TLL2,HTR5A,BFSP1,PDE2A,HOXC13,NGEF,SPRED3,CACNA1S,ALDH1A2,RSPO4,RCAN1,LY6H,VEGFB,COLEC10,TAL1,BATF3,PHLDA2,NUPR1,ARHGAP22,KIAA0319,MTNR1B,CSRNP1,SSTR4,CYBB,RIMBP3,SLC7A5,VAX2,KIRREL3,TRPV2,TNFAIP3,ISLR2,KLF2,KCNC2,NPY,ETNK2,GBA,PEMT,DGAT2,MIP,ADAMTSL2,SCN2B,COL5A3,ADM2,TSSK1B,RFX8,SYN1,SPRR2G,IL7R,GJA10,HM13,DUSP5,NPPB,INHBA,ZNF358,NFAM1,CHAC1,BTBD6,FOXD4L1,GAMT,KRT18,BEND6,PNPLA1,CHRNB2,PALM2,CYP4A11,ASTN2,CABLES1,DACT2,PITPNM1,DRD1,TCTA,HRAS,TNFRSF11A,CDK5R2,LRRTM1,PCSK1,BAD,NCS1,COL8A2,MAL2,CDHR1,ABCA3,CYP46A1,PPARG,HPCAL4,ALK,ATP8A2,DMRT2,USH1G,HOXD11,TPGS1,CDH22,LIMS2,ADORA1,EMP1,KRT37,TMEFF2,TEC,RET,IRF4,FBLL1,ACHE,FOXD4

GO:BP	cellular response to stimulus	GO:0051716	2.5499036775003467e-11	10.593476224711214	UTS2,DBH,CXCL8,GAL,GRP,GPR50,MMP1,FGF3,PRLHR,ADRA1B,RELB,MC5R,SYK,VGF,TIMP4,OR2W3,TH,IL31RA,ARHGAP36,RAMP1,NKX2-2,ADRA2A,EGR2,TRIM58,DRD5,NPFFR2,SUSD5,BHLHA15,FFAR4,TG,PTHLH,HTR6,UGT3A2,GPNMB,RAMP3,FAM83G,MMP8,GABRA5,GATA4,GALR1,KL,CD28,IGFBP3,PTGER4,SOSTDC1,EGR3,PHF24,TPBGL,PTGER2,NR0B1,EGR1,MAFA,PTPRN,CHRM4,UBD,SERPINE1,SYT17,MSC,NPSR1,GPR83,RASAL3,AQP5,GRM7,HRH1,KCNA1,GABRG3,BOK,CITED1,CARTPT,HMOX1,ITPKA,NPBWR1,GPR68,KCNK3,ITGA11,GALR2,OPRD1,ADAP1,TNFRSF12A,HTR7,GCH1,ADGRF4,IL32,BDNF,PALM3,CRLF2,TRPM8,OSGIN1,HAP1,CCKBR,SFRP5,CCL2,ADRA1D,IL1RL2,CCL18,PLCG2,DRD2,OPRL1,IL15RA,GPR137,TBX21,GSG1L,GZMB,CHRDL2,TACR1,KCNK4,S100B,SCG2,GALR3,PAK6,ASIC2,TRIB3,SPHK1,STK32B,NRGN,EPHX1,NXPH3,DCLK3,P2RY2,NTSR2,MAOA,HTR5A,NQO1,NPW,BEGAIN,PDE2A,SRXN1,NGEF,CCL20,SPRED3,SKAP1,CACNA1S,HCRTR1,ALDH1A2,RSPO4,RCAN1,OR2L13,LY6H,VEGFB,COLEC10,PLEKHF1,BATF3,PHLDA2,GABRE,NUPR1,ARHGAP22,KIAA0319,KCNA5,MTNR1B,CSRNP1,SSTR4,CYBB,SLC7A5,VAX2,TNFAIP3,CHRNA6,DUSP26,KLF2,KCNC2,AANAT,NPY,GBA,MGLL,HTR1E,DGAT2,CTSD,ADAMTSL2,CCL5,ADM2,TSSK1B,SLC6A9,TXNRD2,RFX8,PSEN2,PRKAR1B,RHBDD2,SQSTM1,GPR45,UBASH3A,RIN3,AKR1B10,IL7R,MCHR1,HM13,DUSP5,NPPB,OASL,CALY,MAPK8IP1,INHBA,ABCG5,NFAM1,CHAC1,ATP1A3,MLC1,KRT18,BEND6,LY6E,CHRNB2,PALM2,ATP6V0C,HRH2,NENF,GPX3,STK32C,DACT2,PITPNM1,DRD1,RASGEF1C,MS4A2,HRAS,ADGRA1,TNFRSF11A,SYT5,SYT9,NPB,CYGB,PLA2G4C,BAD,TMEM198,IRAK1,ATP6V0D1,NXPH4,FIBP,CYP46A1,PPARG,ALK,GABRQ,HTR2A,RASGEF1A,LIMS2,ADORA1,RGS6,IRAK2,CPNE7,TEC,RET,AMER3,TICAM1,IRF4,ACHE,HCN2,QRFP,SYP
GO:BP	feeding behavior	GO:0007631	3.080648099454792e-11	10.511357908042482	GAL,PRLHR,TH,NPSR1,GPR83,CARTPT,GALR2,OPRD1,DRD2,OPRL1,TACR1,GALR3,NPW,HCRTR1,NPY,ADM2,MCHR1,DRD1,NPB,ATP8A2,QRFP
GO:BP	regulation of multicellular organismal process	GO:0051239	4.387769250521422e-11	10.357756219716348	DBH,CXCL8,GAL,GRP,FGF3,ADRA1B,NKX6-2,RELB,SYK,TNFRSF9,TH,IL31RA,NKX2-2,PHOX2A,ADRA2A,EGR2,OSR2,FFAR4,TG,PTHLH,GPNMB,MMP8,GATA4,GALR1,KL,CD28,SRPX2,PTGER4,SOSTDC1,RBP4,EGR3,EGR1,BCAN,TMEM119,SIX2,SERPINE1,NPSR1,RASAL3,HRH1,KCNA1,CARTPT,CNN1,HMOX1,ITPKA,GPR68,KCNE4,GCH1,IL32,BDNF,CRLF2,TRPM8,FOXL2,HAP1,ASCL2,CCL2,ADRA1D,IL1RL2,ETS1,PLCG2,DRD2,OPRL1,IL15RA,GPR137,SLC4A3,TBX21,TACR1,ASIC2,GJC2,SPHK1,MYH7,HSPB7,CRP,P2RY2,BEGAIN,RAET1G,SPRED3,CACNA1S,VEGFB,TAL1,PHLDA2,NUPR1,KIAA0319,KCNA5,MTNR1B,CYBB,SLC7A5,TRPV2,TNFAIP3,ISLR2,KLF2,GBA,PEMT,MGLL,CCL5,SCN2B,ADM2,PRKAR1B,UBASH3A,IL7R,NPPB,MAPK8IP1,INHBA,ABCG5,NFAM1,ATP1A3,GAMT,CHRNB2,ATP2B3,HRH2,TCTA,HRAS,TNFRSF11A,LRRTM1,CYGB,BAD,IRAK1,PPARG,ATP8A2,DMRT2,HTR2A,ADORA1,TEC,RET,TICAM1,IRF4,ACHE,HCN2,QRFP
GO:BP	developmental process	GO:0032502	6.477213139008558e-11	10.188611811827949	KRT80,CXCL8,GAL,FGF3,NKX6-2,RELB,SYK,TNFRSF9,VGF,TH,IL31RA,RAMP1,NKX2-2,KRT75,TMIE,PHOX2A,EGR2,TRIM58,OSR2,BHLHA15,FFAR4,TG,PTHLH,HTR6,GPNMB,ATP10A,MMP8,GABRA5,GATA4,DRGX,KL,DMBT1,MMP19,CD28,COL8A1,IGFBP3,SRPX2,PTGER4,SOSTDC1,RBP4,EGR3,NR0B1,EGR1,PTPRN,BCAN,TMEM119,SIX2,UBD,SERPINE1,SYT17,MSC,PAX1,C1QL2,MCOLN3,SLC6A17,AQP5,TMEM132E,GRM7,KCNA1,BOK,CITED1,CARTPT,GSC2,HMOX1,ITPKA,GPR68,MALL,KCNK3,ITGA11,GALR2,HOXD9,TNFRSF12A,BDNF,KRT15,PALM3,FOXL2,FOXE1,OSGIN1,SLC24A3,HAP1,ASCL2,CCKBR,SFRP5,KRT86,CCL2,EGFL6,IL1RL2,ETS1,PLCG2,DRD2,IL15RA,IRX6,GPR137,TBX21,CHRDL2,SHROOM1,ACTG2,S100B,SCG2,PAK6,ASIC2,TRIB3,GJC2,SPHK1,MYH7,NRGN,HSPB7,OOSP2,CRP,TLL2,HTR5A,BFSP1,PDE2A,HOXC13,NGEF,SPRED3,CACNA1S,ALDH1A2,RSPO4,RCAN1,LY6H,VEGFB,COLEC10,TAL1,BATF3,PHLDA2,NUPR1,ARHGAP22,KIAA0319,MTNR1B,CSRNP1,SSTR4,CYBB,RIMBP3,SLC7A5,VAX2,KIRREL3,TRPV2,TNFAIP3,ISLR2,SPOCD1,KLF2,KCNC2,NPY,ETNK2,GBA,PEMT,DGAT2,MIP,ADAMTSL2,SCN2B,COL5A3,ADM2,TSSK1B,RFX8,SYN1,SQSTM1,SPRR2G,IL7R,GJA10,HM13,DUSP5,NPPB,INHBA,ZNF358,NFAM1,CHAC1,BTBD6,FOXD4L1,GAMT,KRT18,BEND6,PNPLA1,CHRNB2,PALM2,CYP4A11,ASTN2,CABLES1,DACT2,PITPNM1,DRD1,TCTA,HRAS,TNFRSF11A,CDK5R2,LRRTM1,PCSK1,BAD,NCS1,COL8A2,MAL2,CDHR1,ABCA3,ATP6V0D1,CYP46A1,PPARG,HPCAL4,ALK,ATP8A2,DMRT2,USH1G,ATP6V0A1,HOXD11,TPGS1,HTR2A,CDH22,LIMS2,ADORA1,EMP1,KRT37,TMEFF2,TEC,RET,IRF4,FBLL1,ACHE,TMEM120A,SYP,FOXD4

GO:BP	blood circulation	GO:0008015	8.796693486526506e-11	10.055680540395857	UTS2,DBH,ADRA1B,TH,ADRA2A,DRD5,GATA4,KL,HRH1,CARTPT,KCNE4,HTR7,GCH1,ADRA1D,DRD2,OPRL1,SLC4A3,TACR1,ASIC2,MYH7,HSPB7,CRP,P2RY2,PDE2A,VEGFB,KCNA5,MTNR1B,KLF2,NPY,SCN2B,ADM2,RASL10B,NPPB,ATP1A3,ATP2B3,CYP4A11,HRH2,DRD1,PPARG,HTR2A,ADORA1,HCN2,QRFP
GO:BP	G protein-coupled receptor signaling pathway, coupled to cyclic nucleotide second messenger	GO:0007187	1.1294788808935214e-10	9.94712189	MC5R,HTR6,CHRM4,HRH1,OPRD1,HTR7,CCL2,GALR3,HTR5A,MTNR1B,SSTR4,NPY,HTR1E,HRH2,DRD1,HTR2A
GO:BP	establishment of localization	GO:0051234	1.2306289868526598e-10	9.909872859537366	SLC6A2,CXCL8,FOLR3,GAL,GRP,SYK,VGF,NEURL3,TH,RAMP1,NGB,ADRA2A,EGR2,TRIM58,BHLHA15,SYTL5,FFAR4,SLC18A3,TG,RAMP3,KCNK12,ATP10A,APOL6,GABRA5,GALR1,DMBT1,SLC5A10,NIPAL2,RBP4,REM1,LGI3,MAFA,PTPRN,GOLT1A,KCNS2,SIX2,SERPINE1,SYT17,KCNK13,NPSR1,SLC10A4,MCOLN3,SLC6A17,AQP5,GRM7,KCNA1,GABRG3,KCNS1,SLC7A4,BOK,CITED1,CARTPT,OTOP2,SV2C,GPR68,APOF,KCNK3,DYSF,KCNE4,GALR2,OPRD1,B4GALT1-AS1,TRPM8,FOXL2,SLC24A3,HAP1,CCKBR,CCL2,SLC6A11,PLCG2,SLC2A6,DRD2,SLC1A4,OPRL1,IL15RA,RAB3IL1,SLC4A3,GSG1L,GZMB,TACR1,KCNK4,SCG2,ASIC2,TRIB3,GJC2,SPHK1,RTP1,CRP,P2RY2,MYRIP,CACNA1S,CABP1,COLEC10,PLEKHF1,GABRE,KCNA5,MTNR1B,SSTR4,CYBB,SLC7A5,TRPV2,TNFAIP3,CHRNA6,SLC5A1,TMPRSS3,KCNC2,CTSD,MIP,CCL5,SCN2B,SLC6A9,PSEN2,PRKAR1B,KCNK15,SYN1,SQSTM1,RIN3,OTOP3,RASL10B,GJA10,MCHR1,HM13,NPPB,CALY,MAPK8IP1,INHBA,ABCG5,ATP1A3,ATP6V0B,MLC1,KRT18,SLC6A12,CHRNB2,ATP2B3,SLC35F4,ATP6V0C,CYP4A11,HRH2,ASTN2,TEX261,PITPNM1,DRD1,DOC2B,HRAS,REEP2,SLC37A1,SPRN,TNFRSF11A,CDK5R2,LRRTM1,SYT5,PCSK1,SYT9,CYGB,BAD,NCS1,KCNIP1,KIF17,ABCA3,ATP6V0D1,PPARG,ATP8A2,DYNC1I1,USH1G,SLC36A1,ATP6V0A1,GABRQ,ZDHHC14,HTR2A,ADORA1,SLC45A1,SNCB,PARD6A,ACHE,ABLIM3,HCN2,TMEM120A,SLC22A3,SYP,PQLC2
GO:BP	G protein-coupled receptor signaling pathway	GO:0007186	1.55969748221541e-10	9.806959628918014	CXCL8,GAL,GRP,GPR50,PRLHR,ADRA1B,MC5R,OR2W3,RAMP1,ADRA2A,DRD5,NPFFR2,BHLHA15,FFAR4,PTHLH,HTR6,RAMP3,GALR1,PTGER4,PHF24,PTGER2,CHRM4,NPSR1,GPR83,GRM7,HRH1,CARTPT,NPBWR1,GPR68,GALR2,OPRD1,HTR7,ADGRF4,CCKBR,CCL2,ADRA1D,CCL18,DRD2,OPRL1,TACR1,GALR3,SPHK1,NXPH3,P2RY2,NTSR2,HTR5A,NPW,PDE2A,CCL20,HCRTR1,OR2L13,MTNR1B,SSTR4,NPY,MGLL,HTR1E,CCL5,ADM2,GPR45,MCHR1,NPPB,HRH2,DRD1,ADGRA1,NPB,NXPH4,PPARG,HTR2A,ADORA1,RGS6,QRFP,SYP
GO:BP	inorganic cation transmembrane transport	GO:0098662	2.77705073443132e-10	9.55641619	SLC6A2,GAL,GRP,ADRA2A,BHLHA15,SLC18A3,RAMP3,KCNK12,SLC5A10,NIPAL2,REM1,KCNS2,KCNK13,NPSR1,MCOLN3,SLC6A17,KCNA1,KCNS1,OTOP2,KCNK3,KCNE4,GALR2,TRPM8,SLC24A3,HAP1,SLC6A11,PLCG2,DRD2,OPRL1,KCNK4,ASIC2,GJC2,CACNA1S,CABP1,KCNA5,MTNR1B,TRPV2,SLC5A1,KCNC2,SCN2B,SLC6A9,PSEN2,KCNK15,OTOP3,ATP1A3,ATP6V0B,SLC6A12,ATP2B3,ATP6V0C,DRD1,NCS1,KCNIP1,ATP6V0D1,SLC36A1,ATP6V0A1,HTR2A,HCN2
GO:BP	monoatomic cation transport	GO:0006812	3.1031339993271437e-10	9.508199470335315	SLC6A2,GAL,GRP,RAMP1,ADRA2A,BHLHA15,SLC18A3,RAMP3,KCNK12,SLC5A10,NIPAL2,REM1,KCNS2,KCNK13,NPSR1,SLC10A4,MCOLN3,SLC6A17,KCNA1,KCNS1,OTOP2,KCNK3,KCNE4,GALR2,OPRD1,TRPM8,SLC24A3,HAP1,CCL2,SLC6A11,PLCG2,DRD2,OPRL1,KCNK4,ASIC2,GJC2,CACNA1S,CABP1,KCNA5,MTNR1B,TRPV2,SLC5A1,KCNC2,CCL5,SCN2B,SLC6A9,PSEN2,KCNK15,OTOP3,MCHR1,ATP1A3,ATP6V0B,SLC6A12,CHRNB2,ATP2B3,ATP6V0C,DRD1,NCS1,KCNIP1,ATP6V0D1,SLC36A1,ATP6V0A1,HTR2A,ADORA1,HCN2
GO:BP	regulation of secretion	GO:0051046	3.647270714308125e-10	9.438032000501178	GAL,GRP,SYK,ADRA2A,FFAR4,SLC18A3,GALR1,RBP4,LGI3,PTPRN,SYT17,NPSR1,GRM7,CARTPT,SV2C,GPR68,DYSF,FOXL2,HAP1,DRD2,TACR1,P2RY2,MYRIP,KCNA5,MTNR1B,SSTR4,CHRNA6,CCL5,SYN1,RASL10B,NPPB,INHBA,CHRNB2,CYP4A11,DOC2B,TNFRSF11A,CDK5R2,SYT5,PCSK1,SYT9,BAD,PPARG,HTR2A,ADORA1,PARD6A,ACHE,SYP
GO:BP	vascular process in circulatory system	GO:0003018	6.026228050458698e-10	9.219954437352044	UTS2,DBH,ADRA1B,ADRA2A,DRD5,SLC6A17,HRH1,HTR7,GCH1,SLC24A3,ADRA1D,SLC1A4,SLC4A3,TACR1,ASIC2,CRP,P2RY2,PDE2A,KCNA5,MTNR1B,SLC7A5,SLC5A1,KLF2,SLC6A9,NPPB,HRH2,DRD1,HTR2A,ADORA1,SLC22A3

GO:BP	localization	GO:0051179	7.0002287611939e-10	9.15488777	SLC6A2,CXCL8,FOLR3,GAL,GRP,SYK,VGF,NEURL3,TH,RAMP1,NGB,ADRA2A,EGR2,TRIM58,BHLHA15,SYTL5,FFAR4,SLC18A3,TG,RAMP3,KCNK12,ATP10A,APOL6,GABRA5,GALR1,DMBT1,SLC5A10,NIPAL2,RBP4,REM1,LGI3,NR0B1,MAFA,PTPRN,GOLT1A,KCNS2,SIX2,SERPINE1,SYT17,KCNK13,NPSR1,SLC10A4,C1QL2,MCOLN3,SLC6A17,AQP5,GRM7,KCNA1,GABRG3,KCNS1,SLC7A4,BOK,CITED1,CARTPT,OTOP2,SV2C,GPR68,APOF,KCNK3,DYSF,KCNE4,GALR2,OPRD1,B4GALT1-AS1,BDNF,TRPM8,FOXL2,SLC24A3,HAP1,CCKBR,CCL2,SLC6A11,PLCG2,SLC2A6,DRD2,SLC1A4,OPRL1,IL15RA,RAB3IL1,SLC4A3,GSG1L,GZMB,TACR1,KCNK4,SCG2,ASIC2,TRIB3,GJC2,SPHK1,RTP1,CRP,DCLK3,P2RY2,TRIM29,MYRIP,SKAP1,CACNA1S,CABP1,COLEC10,PLEKHF1,GABRE,KCNA5,MTNR1B,SSTR4,CYBB,SLC7A5,HEPACAM,TRPV2,TNFAIP3,CHRNA6,SLC5A1,TMPRSS3,KCNC2,GBA,DGAT2,CTSD,MIP,CCL5,SCN2B,SLC6A9,PSEN2,PRKAR1B,KCNK15,SYN1,SQSTM1,RIN3,OTOP3,RASL10B,GJA10,MCHR1,HM13,NPPB,CALY,MAPK8IP1,INHBA,ABCG5,SLC9A3R2,ATP1A3,ATP6V0B,MLC1,KRT18,SLC6A12,CHRNB2,ATP2B3,PALM2,SLC35F4,ATP6V0C,CYP4A11,HRH2,ASTN2,TEX261,PITPNM1,DRD1,DOC2B,HRAS,REEP2,SLC37A1,SPRN,TNFRSF11A,CDK5R2,LRRTM1,SYT5,PCSK1,SYT9,CYGB,PLA2G4C,BAD,NCS1,KCNIP1,KIF17,ABCA3,ATP6V0D1,CYP46A1,PPARG,ATP8A2,DYNC1I1,USH1G,SLC36A1,ATP6V0A1,GABRQ,ZDHHC14,TPGS1,HTR2A,ADORA1,SLC45A1,SNCB,PARD6A,ACHE,ABLIM3,HCN2,TMEM120A,SLC22A3,S
GO:BP	regulation of cell communication	GO:0010646	8.335374183886451e-10	9.0790749	DBH,CXCL8,GAL,GRP,ADRA1B,SYK,VGF,RAMP1,ADRA2A,EGR2,TRIM58,DRD5,NPFFR2,FFAR4,SLC18A3,HTR6,GPNMB,RAMP3,MMP8,GATA4,GALR1,KL,CD28,IGFBP3,SOSTDC1,RBP4,PHF24,TPBGL,NR0B1,EGR1,UBD,SERPINE1,NPSR1,RASAL3,GRM7,HRH1,BOK,CITED1,CARTPT,HMOX1,ITPKA,SV2C,GPR68,DYSF,TNFRSF12A,BDNF,PALM3,CRLF2,FOXL2,HAP1,SFRP5,CCL2,CCL18,PLCG2,DRD2,OPRL1,GPR137,GSG1L,CHRDL2,TACR1,S100B,SCG2,PAK6,SPHK1,MAOA,BEGAIN,MYRIP,PDE2A,NGEF,CCL20,SPRED3,HCRTR1,RSPO4,RCAN1,VEGFB,PLEKHF1,PHLDA2,NUPR1,ARHGAP22,KIAA0319,KCNA5,MTNR1B,SSTR4,TNFAIP3,CHRNA6,DUSP26,KLF2,GBA,MGLL,CTSD,ADAMTSL2,CCL5,TSSK1B,SLC6A9,RFX8,PRKAR1B,SYN1,SQSTM1,UBASH3A,RASL10B,IL7R,DUSP5,OASL,MAPK8IP1,INHBA,NFAM1,CHAC1,BEND6,CHRNB2,ATP6V0C,NENF,DACT2,DRD1,DOC2B,HRAS,LRFN2,TNFRSF11A,LRRTM1,BAD,TMEM198,IRAK1,CYP46A1,PPARG,ALK,HTR2A,RASGEF1A,LIMS2,ADORA1,RGS6,IRAK2,RET,AMER3,TICAM1,IRF4,ACHE,HCN2,SYP
GO:BP	monoatomic cation transmembrane transport	GO:0098655	8.757127418910676e-10	9.057638331127404	SLC6A2,GAL,GRP,ADRA2A,BHLHA15,SLC18A3,RAMP3,KCNK12,SLC5A10,NIPAL2,REM1,KCNS2,KCNK13,NPSR1,MCOLN3,SLC6A17,KCNA1,KCNS1,OTOP2,KCNK3,KCNE4,GALR2,TRPM8,SLC24A3,HAP1,SLC6A11,PLCG2,DRD2,OPRL1,KCNK4,ASIC2,GJC2,CACNA1S,CABP1,KCNA5,MTNR1B,TRPV2,SLC5A1,KCNC2,SCN2B,SLC6A9,PSEN2,KCNK15,OTOP3,ATP1A3,ATP6V0B,SLC6A12,ATP2B3,ATP6V0C,DRD1,NCS1,KCNIP1,ATP6V0D1,SLC36A1,ATP6V0A1,HTR2A,HCN2
GO:BP	positive regulation of transport	GO:0051050	1.1440114729803632e-9	8.941569620099745	GAL,GRP,SYK,ADRA2A,FFAR4,SLC18A3,RAMP3,GALR1,RBP4,SERPINE1,NPSR1,KCNA1,BOK,CARTPT,GPR68,KCNK3,GALR2,FOXL2,HAP1,CCL2,PLCG2,DRD2,IL15RA,GZMB,TACR1,ASIC2,GJC2,P2RY2,MYRIP,COLEC10,SSTR4,SLC7A5,TRPV2,KCNC2,CCL5,RASL10B,MCHR1,NPPB,CALY,INHBA,MLC1,CHRNB2,CYP4A11,DRD1,DOC2B,HRAS,TNFRSF11A,CDK5R2,PCSK1,SYT9,BAD,ABCA3,PPARG,ATP8A2,ADORA1,PARD6A,ACHE,ABLIM3

GO:BP	regulation of biological process	GO:0050789	1.3990221685843471e-9	8.854175403722916	SLC6A2,UTS2,DBH,CXCL8,GAL,GRP,GPR50,EGR4,MMP1,FGF3,PRLHR,ADRA1B,NKX6-2,RELB,MC5R,SYK,TNFRSF9,VGF,TIMP4,OR2W3,TH,IL31RA,ARHGAP36,RAMP1,NKX2-2,PHOX2A,ADRA2A,STYK1,EGR2,TRIM58,DRD5,NPFFR2,SUSD5,OSR2,BHLHA15,FFAR4,SLC18A3,TG,PTHLH,HTR6,GPNMB,RAMP3,P3H2,KCNK12,FAM83G,ATP10A,MMP8,GABRA5,GATA4,DRGX,GALR1,KL,CD28,COL8A1,IGFBP3,SRPX2,PTGER4,SOSTDC1,RBP4,REM1,LGI3,EGR3,PHF24,TPBGL,PTGER2,NR0B1,EGR1,ME1,MAFA,PTPRN,BCAN,CHRM4,KCNS2,TMEM119,SIX2,UBD,SERPINE1,SYT17,KCNK13,MSC,SERPINA3,NPSR1,PAX1,C1QL2,MCOLN3,GPR83,RASAL3,GRM7,HRH1,KCNA1,GABRG3,KCNS1,BOK,CITED1,CARTPT,CNN1,GSC2,HMOX1,MAGEB17,ITPKA,NPBWR1,SV2C,GPR68,KCNK3,DYSF,ITGA11,KCNE4,GALR2,OPRD1,ADAP1,HOXD9,HAS1,TNFRSF12A,HTR7,GCH1,ADGRF4,IL32,BDNF,PALM3,SPTB,CRLF2,TRPM8,FOXL2,FOXE1,OSGIN1,SLC24A3,HAP1,ASCL2,CCKBR,SFRP5,FCMR,CCL2,EGFL6,ADRA1D,IL1RL2,ETS1,CCL18,PLCG2,SLC2A6,DRD2,SLC1A4,OPRL1,IL15RA,IRX6,GPR137,SLC4A3,TBX21,GSG1L,GZMB,CHRDL2,TACR1,KCNK4,ACTG2,S100B,SCG2,GALR3,PAK6,ASIC2,TRIB3,GJC2,SPHK1,MYH7,STK32B,NRGN,HSPB7,NXPH3,CRP,DCLK3,P2RY2,TLL2,TRIM29,NTSR2,MAOA,HTR5A,NQO1,NPW,ZNF771,BEGAIN,MYRIP,PDE2A,HOXC13,RAET1G,NGEF,CCL20,SPRED3,SKAP1,CACNA1S,HCRTR1,CABP1,ALDH1A2,RSPO4,RCAN1,OR2L13,LY6H,VEGFB,COLEC10,TAL1,PLEKHF1,BATF3,PHLDA2,PNMA5,GABRE,NUPR1,ARHGAP22,KIAA0319,KCNA5,MTNR1B,CSRNP1,SSTR4,CYBB,SLC7A5,VAX2,HEPACAM,TRPV2,TNFAIP3,ISLR2,SPOCD1,CHRNA6,DUSP26,KLF2,KCNC2,NPY,SYNPO,GBA,PEMT,MGLL,HTR1E,DGAT2,MMP7,CTSD,MIP,ADAMTSL2,CCL5,SCN2B,ADM2,TSSK1B,SLC6A9,RFX8,PSEN2,PRKAR1B,RHBDD2,KCNK15,SYN1,SQSTM1,GPR45,UBASH3A,RIN3,RASL10B,IL7R,GJA10,MCHR1,DUSP5,NPPB,OASL,CALY,MAPK8IP1,INHBA,ZNF358,ABCG5,ZNF860,NFAM1,CHAC1,ATP1A3,FOXD4L1,ATP6V0B,MLC1,GAMT,KRT18,BEND6,LY6E,DBNDD1,CHRNB2,ATP2B3,PALM2,ATP6V0C,CYP4A11,HRH2,ASTN2,CABLES1,TEX261,NENF,STK32C,LAMP3,DACT2,ZNF469,PITPNM1,DRD1,DOC2B,RASGEF1C,TCTA,MS4A2,HRAS,LRFN2,REEP2,ADGRA1,TNFRSF11A,CDK5R2,LRRTM1,SYT5,PCSK1,SYT9,NPB,CYGB,PLA2G4C,BAD,NCS1,KCNIP1,TMEM198,IRAK1,ABCA3,ATP6V0D1,NXPH4,FIBP,CYP46A1,PPARG,ALK,CAMK2N2,ATP8A2,DMRT2,USH1G,ATP6V0A1,GABRQ,HOXD11,TPGS1,HTR2A,RASGEF1A,LIMS2,ADORA1,RGS6,IRAK2,TMEFF2,TEC,RET,SNCB,AMER3,TICAM1,IRF4,PARD6A,FBLL1,ACHE,ABLIM3,HCN2,QRFP,NOXO1,SYP,FOXD4
GO:BP	regulation of signaling	GO:0023051	1.5144690597416388e-9	8.819739594777962	DBH,CXCL8,GAL,GRP,ADRA1B,SYK,VGF,RAMP1,ADRA2A,EGR2,TRIM58,DRD5,NPFFR2,FFAR4,SLC18A3,HTR6,GPNMB,RAMP3,MMP8,GATA4,GALR1,KL,CD28,IGFBP3,SOSTDC1,RBP4,PHF24,TPBGL,NR0B1,EGR1,UBD,SERPINE1,NPSR1,RASAL3,GRM7,HRH1,BOK,CITED1,CARTPT,HMOX1,ITPKA,SV2C,GPR68,DYSF,TNFRSF12A,BDNF,PALM3,CRLF2,FOXL2,HAP1,SFRP5,CCL2,CCL18,PLCG2,DRD2,OPRL1,GPR137,GSG1L,CHRDL2,TACR1,S100B,SCG2,PAK6,SPHK1,MAOA,BEGAIN,MYRIP,PDE2A,NGEF,CCL20,SPRED3,HCRTR1,RSPO4,RCAN1,VEGFB,PLEKHF1,PHLDA2,NUPR1,ARHGAP22,KIAA0319,KCNA5,MTNR1B,SSTR4,TNFAIP3,CHRNA6,DUSP26,KLF2,GBA,MGLL,CTSD,ADAMTSL2,CCL5,TSSK1B,SLC6A9,RFX8,PRKAR1B,SYN1,SQSTM1,UBASH3A,RASL10B,IL7R,DUSP5,OASL,MAPK8IP1,INHBA,NFAM1,CHAC1,BEND6,CHRNB2,ATP6V0C,NENF,DACT2,DRD1,DOC2B,HRAS,LRFN2,TNFRSF11A,LRRTM1,BAD,TMEM198,IRAK1,CYP46A1,PPARG,ALK,HTR2A,RASGEF1A,LIMS2,ADORA1,RGS6,IRAK2,RET,AMER3,TICAM1,IRF4,ACHE,SYP
GO:BP	response to organic substance	GO:0010033	1.8233193178126335e-9	8.739137266715314	DBH,CXCL8,GAL,RELB,SYK,VGF,TIMP4,TH,IL31RA,RAMP1,NKX2-2,ADRA2A,EGR2,DRD5,NPFFR2,BHLHA15,FFAR4,HTR6,UGT3A2,RAMP3,GATA4,KL,MMP19,PTGER4,RBP4,PTGER2,NR0B1,EGR1,ME1,MAFA,PTPRN,CHRM4,UBD,SERPINE1,SYT17,MSC,GPR83,HRH1,BOK,CITED1,HMOX1,ITPKA,GPR68,HTR7,GCH1,PALM3,CRLF2,CCL2,IL1RL2,CCL18,PLCG2,DRD2,OPRL1,IL15RA,TBX21,TACR1,KCNK4,TRIB3,SPHK1,EPHX1,HSPB7,P2RY2,HTR5A,NQO1,PDE2A,CCL20,CACNA1S,HCRTR1,ALDH1A2,LY6H,KCNA5,SSTR4,CYBB,SLC7A5,TNFAIP3,KLF2,KCNC2,AANAT,GBA,HTR1E,DGAT2,CTSD,CCL5,RHBDD2,AKR1B10,IL7R,HM13,OASL,CALY,INHBA,CHAC1,ATP1A3,MLC1,KRT18,CHRNB2,HRH2,GPX3,LAMP3,DRD1,HRAS,TNFRSF11A,SYT5,PCSK1,SYT9,BAD,IRAK1,ABCA3,PPARG,ALK,HTR2A,ADORA1,IRAK2,CPNE7,RET,TICAM1,ACHE,HCN2,SYP

GO:BP	biological regulation	GO:0065007	1.8509631086990324e-9	8.73260224	SLC6A2,UTS2,DBH,CXCL8,GAL,GRP,GPR50,EGR4,MMP1,FGF3,PRLHR,ADRA1B,NKX6-2,RELB,MC5R,SYK,TNFRSF9,VGF,TIMP4,OR2W3,TH,IL31RA,ARHGAP36,RAMP1,NKX2-2,PHOX2A,ADRA2A,STYK1,EGR2,TRIM58,DRD5,NPFFR2,SUSD5,OSR2,BHLHA15,FFAR4,SLC18A3,TG,PTHLH,HTR6,GPNMB,RAMP3,P3H2,KCNK12,FAM83G,ATP10A,MMP8,GABRA5,GATA4,DRGX,GALR1,KL,CD28,COL8A1,IGFBP3,SRPX2,PTGER4,SOSTDC1,RBP4,REM1,LGI3,EGR3,PHF24,TPBGL,PTGER2,NR0B1,EGR1,ME1,MAFA,PTPRN,BCAN,CHRM4,KCNS2,TMEM119,SIX2,UBD,SERPINE1,SYT17,KCNK13,MSC,SERPINA3,NPSR1,PAX1,C1QL2,MCOLN3,GPR83,RASAL3,AQP5,GRM7,HRH1,KCNA1,GABRG3,KCNS1,BOK,CITED1,CARTPT,CNN1,GSC2,HMOX1,MAGEB17,ITPKA,NPBWR1,SV2C,GPR68,KCNK3,DYSF,ITGA11,KCNE4,GALR2,OPRD1,ADAP1,HOXD9,HAS1,TNFRSF12A,HTR7,GCH1,ADGRF4,IL32,BDNF,PALM3,SPTB,CRLF2,TRPM8,FOXL2,FOXE1,OSGIN1,SLC24A3,HAP1,ASCL2,CCKBR,SFRP5,FCMR,CCL2,EGFL6,ADRA1D,IL1RL2,ETS1,SLC6A11,CCL18,PLCG2,SLC2A6,DRD2,SLC1A4,OPRL1,IL15RA,IRX6,GPR137,SLC4A3,TBX21,GSG1L,GZMB,CHRDL2,TACR1,KCNK4,ACTG2,S100B,SCG2,GALR3,PAK6,ASIC2,TRIB3,GJC2,SPHK1,MYH7,STK32B,NRGN,HSPB7,NXPH3,CRP,DCLK3,P2RY2,TLL2,TRIM29,NTSR2,MAOA,HTR5A,NQO1,NPW,ZNF771,BEGAIN,MYRIP,PDE2A,HOXC13,RAET1G,NGEF,CCL20,SPRED3,SKAP1,CACNA1S,HCRTR1,CABP1,ALDH1A2,RSPO4,RCAN1,OR2L13,LY6H,VEGFB,COLEC10,TAL1,PLEKHF1,BATF3,PHLDA2,PNMA5,GABRE,NUPR1,ARHGAP22,KIAA0319,KCNA5,MTNR1B,CSRNP1,SSTR4,CYBB,SLC7A5,VAX2,HEPACAM,TRPV2,TNFAIP3,ISLR2,SPOCD1,CHRNA6,DUSP26,KLF2,KCNC2,AANAT,NPY,SYNPO,GBA,PEMT,MGLL,HTR1E,DGAT2,MMP7,CTSD,MIP,ADAMTSL2,CCL5,SCN2B,ADM2,TSSK1B,SLC6A9,RFX8,PSEN2,PRKAR1B,RHBDD2,KCNK15,SYN1,SQSTM1,GPR45,UBASH3A,RIN3,AKR1B10,RASL10B,IL7R,GJA10,MCHR1,HM13,DUSP5,NPPB,OASL,CALY,MAPK8IP1,INHBA,ZNF358,ABCG5,ZNF860,NFAM1,CHAC1,ATP1A3,FOXD4L1,ATP6V0B,MLC1,GAMT,KRT18,SLC6A12,BEND6,LY6E,DBNDD1,CHRNB2,ATP2B3,PALM2,ATP6V0C,CYP4A11,HRH2,ASTN2,CABLES1,TEX261,NENF,STK32C,LAMP3,DACT2,ZNF469,PITPNM1,DRD1,DOC2B,RASGEF1C,TCTA,MS4A2,HRAS,LRFN2,REEP2,ADGRA1,TNFRSF11A,CDK5R2,LRRTM1,SYT5,PCSK1,SYT9,NPB,CYGB,PLA2G4C,BAD,NCS1,KCNIP1,TMEM198,IRAK1,ABCA3,ATP6V0D1,NXPH4,FIBP,CYP46A1,PPARG,ALK,CAMK2N2,ATP8A2,DMRT2,USH1G,ATP6V0A1,GABRQ,HOXD11,TPGS1,HTR2A,RASGEF1A,LIMS2,ADORA1,RGS6,IRAK2,TMEFF2,TEC,RET,SNCB,AMER3,TICAM1,DHCR7,IRF4,PARD6A,FBLL1,ACHE,ABLIM3,HCN2,QRFP,SLC22A3,NOXO1,SYP,FOXD4
GO:BP	regulation of system process	GO:0044057	1.982888644921816e-9	8.702701674223285	DBH,GAL,ADRA1B,TH,ADRA2A,EGR2,GATA4,GALR1,NPSR1,HRH1,KCNA1,CARTPT,CNN1,KCNE4,GCH1,FOXL2,ADRA1D,DRD2,SLC4A3,TACR1,ASIC2,SPHK1,MYH7,HSPB7,BEGAIN,CACNA1S,KCNA5,MTNR1B,GBA,MGLL,SCN2B,ADM2,PRKAR1B,NPPB,INHBA,ABCG5,ATP1A3,ATP2B3,HRH2,PPARG,HTR2A,ADORA1,HCN2
GO:BP	metal ion transport	GO:0030001	2.4107183390547664e-9	8.617853528295285	SLC6A2,GAL,GRP,RAMP1,ADRA2A,BHLHA15,RAMP3,KCNK12,SLC5A10,NIPAL2,REM1,KCNS2,KCNK13,NPSR1,SLC10A4,MCOLN3,SLC6A17,KCNA1,KCNS1,KCNK3,KCNE4,GALR2,OPRD1,TRPM8,SLC24A3,HAP1,CCL2,SLC6A11,PLCG2,DRD2,OPRL1,KCNK4,ASIC2,GJC2,CACNA1S,CABP1,KCNA5,MTNR1B,TRPV2,SLC5A1,KCNC2,CCL5,SCN2B,SLC6A9,PSEN2,KCNK15,MCHR1,ATP1A3,SLC6A12,CHRNB2,ATP2B3,DRD1,NCS1,KCNIP1,HTR2A,ADORA1,HCN2
GO:BP	behavior	GO:0007610	2.8232821183566094e-9	8.549245722517712	DBH,GAL,GRP,PRLHR,TH,EGR2,DRD5,GABRA5,SRPX2,EGR1,NPSR1,MCOLN3,GPR83,HRH1,CARTPT,GALR2,OPRD1,HOXD9,BDNF,DRD2,OPRL1,TACR1,KCNK4,S100B,GALR3,PAK6,NPW,HCRTR1,RCAN1,TAL1,MTNR1B,KIRREL3,NPY,GBA,ADM2,PRKAR1B,MCHR1,CHRNB2,DRD1,LRRTM1,NPB,ALK,ATP8A2,TPGS1,HTR2A,ADORA1,QRFP

GO:BP	response to chemical	GO:0042221	3.14570803 52749494e-9	8.502281588 309408	SLC6A2,DBH,CXCL8,GAL,RELB,SYK,VGF,TIMP4,OR2W3,TH,IL31RA,RAMP1,NKX2-2,ADRA2A,EGR2,DRD5,NPFFR2,BHLHA15,FFAR4,HTR6,UGT3A2,GPNMB,RAMP3,GATA4,DRGX,KL,MMP19,PTGER4,RBP4,EGR3,PTGER2,NR0B1,EGR1,ME1,MAFA,PTPRN,CHRM4,UBD,SERPINE1,SYT17,MSC,GPR83,AQP5,HRH1,KCNA1,GABRG3,BOK,CITED1,CARTPT,HMOX1,ITPKA,GPR68,KCNK3,OPRD1,HTR7,GCH1,PALM3,CRLF2,CCL2,IL1RL2,ETS1,SLC6A11,CCL18,PLCG2,DRD2,OPRL1,IL15RA,TBX21,TACR1,KCNK4,SCG2,ASIC2,TRIB3,GJC2,SPHK1,RTP1,EPHX1,HSPB7,P2RY2,HTR5A,NQO1,PDE2A,SRXN1,CCL20,CACNA1S,HCRTR1,ALDH1A2,OR2L13,LY6H,VEGFB,COLEC10,NUPR1,KCNA5,SSTR4,CYBB,SLC7A5,TNFAIP3,SLC5A1,KLF2,KCNC2,AANAT,NPY,GBA,HTR1E,DGAT2,CTSD,CCL5,SCN2B,TXNRD2,RHBDD2,SQSTM1,RIN3,AKR1B10,IL7R,HM13,OASL,CALY,INHBA,ABCG5,CHAC1,ATP1A3,MLC1,KRT18,CHRNB2,HRH2,NENF,GPX3,LAMP3,DRD1,HRAS,TNFRSF11A,SYT5,PCSK1,SYT9,CYGB,BAD,IRAK1,ABCA3,ATP6V0D1,CYP46A1,PPARG,ALK,HTR2A,ADORA1,IRAK2,CPNE7,RET,TICAM1,ACHE,HCN2,SYP
GO:BP	response to oxygen-containing compound	GO:1901700	6.78505977 25258115e-9	8.168446322 099486	DBH,CXCL8,GAL,VGF,TH,NKX2-2,ADRA2A,EGR2,DRD5,HTR6,UGT3A2,RAMP3,GATA4,KL,MMP19,PTGER4,RBP4,PTGER2,EGR1,ME1,MAFA,PTPRN,CHRM4,SERPINE1,SYT17,HRH1,CITED1,HMOX1,ITPKA,GPR68,HTR7,GCH1,PALM3,CCL2,PLCG2,DRD2,OPRL1,TACR1,KCNK4,TRIB3,SPHK1,P2RY2,HTR5A,NQO1,PDE2A,ALDH1A2,LY6H,KCNA5,CYBB,SLC7A5,TNFAIP3,KLF2,KCNC2,AANAT,GBA,HTR1E,DGAT2,CTSD,CCL5,TXNRD2,SQSTM1,AKR1B10,CALY,INHBA,ATP1A3,MLC1,CHRNB2,HRH2,GPX3,DRD1,HRAS,TNFRSF11A,SYT5,PCSK1,SYT9,BAD,IRAK1,PPARG,ALK,HTR2A,IRAK2,CPNE7,RET,TICAM1,ACHE,HCN2
GO:BP	adenylate cyclase-modulating G protein-coupled receptor signaling pathway	GO:0007188	7.52781756 4559827e-9	8.123330914 509737	ADRA1B,MC5R,RAMP1,ADRA2A,DRD5,PTHLH,RAMP3,GALR1,PTGER4,PTGER2,CHRM4,GRM7,GALR2,OPRD1,ADGRF4,ADRA1D,DRD2,OPRL1,GALR3,HTR5A,PDE2A,SSTR4,HTR1E,ADM2,MCHR1,DRD1,ADORA1
GO:BP	positive regulation of multicellular organismal process	GO:0051240	9.23251779 1004678e-9	8.034679846 793397	DBH,CXCL8,GAL,GRP,ADRA1B,NKX6-2,SYK,NKX2-2,ADRA2A,EGR2,OSR2,FFAR4,MMP8,GATA4,GALR1,KL,CD28,SRPX2,PTGER4,EGR3,EGR1,BCAN,TMEM119,SERPINE1,RASAL3,HRH1,CARTPT,HMOX1,ITPKA,GPR68,GCH1,IL32,BDNF,CRLF2,TRPM8,FOXL2,HAP1,CCL2,ADRA1D,IL1RL2,ETS1,PLCG2,DRD2,IL15RA,TBX21,TACR1,ASIC2,GJC2,SPHK1,P2RY2,RAET1G,CACNA1S,VEGFB,MTNR1B,CYBB,SLC7A5,TRPV2,ISLR2,GBA,PEMT,CCL5,ADM2,PRKAR1B,IL7R,NPPB,INHBA,NFAM1,CHRNB2,HRH2,HRAS,TNFRSF11A,LRRTM1,BAD,IRAK1,PPARG,ATP8A2,HTR2A,RET,TICAM1,IRF4,ACHE
GO:BP	blood vessel diameter maintenance	GO:0097746	1.47821360 39170329e-8	7.83026281	UTS2,DBH,ADRA1B,ADRA2A,DRD5,HRH1,HTR7,GCH1,ADRA1D,TACR1,ASIC2,CRP,P2RY2,KCNA5,MTNR1B,KLF2,NPPB,HRH2,DRD1,HTR2A,ADORA1
GO:BP	regulation of tube diameter	GO:0035296	1.47821360 39170329e-8	7.83026281	UTS2,DBH,ADRA1B,ADRA2A,DRD5,HRH1,HTR7,GCH1,ADRA1D,TACR1,ASIC2,CRP,P2RY2,KCNA5,MTNR1B,KLF2,NPPB,HRH2,DRD1,HTR2A,ADORA1
GO:BP	regulation of tube size	GO:0035150	1.69768318 09779263e-8	7.770143353 898264	UTS2,DBH,ADRA1B,ADRA2A,DRD5,HRH1,HTR7,GCH1,ADRA1D,TACR1,ASIC2,CRP,P2RY2,KCNA5,MTNR1B,KLF2,NPPB,HRH2,DRD1,HTR2A,ADORA1

GO:BP	multicellular organism development	GO:0007275	2.5213200093977564e-8	7.598372029573229	CXCL8,GAL,FGF3,NKX6-2,SYK,VGF,TH,RAMP1,NKX2-2,TMIE,PHOX2A,EGR2,OSR2,BHLHA15,TG,PTHLH,HTR6,GPNMB,MMP8,GABRA5,GATA4,DRGX,KL,MMP19,CD28,COL8A1,SRPX2,PTGER4,SOSTDC1,RBP4,EGR3,NR0B1,EGR1,PTPRN,BCAN,TMEM119,SIX2,SERPINE1,SYT17,MSC,PAX1,C1QL2,MCOLN3,SLC6A17,AQP5,TMEM132E,GRM7,KCNA1,BOK,CITED1,CARTPT,HMOX1,ITPKA,GPR68,MALL,GALR2,HOXD9,TNFRSF12A,BDNF,FOXL2,FOXE1,HAP1,ASCL2,CCKBR,SFRP5,CCL2,IL1RL2,ETS1,DRD2,IL15RA,IRX6,GPR137,TBX21,CHRDL2,S100B,SCG2,PAK6,ASIC2,GJC2,SPHK1,MYH7,NRGN,HSPB7,TLL2,HTR5A,BFSP1,PDE2A,HOXC13,NGEF,SPRED3,CACNA1S,ALDH1A2,RSPO4,LY6H,VEGFB,TAL1,PHLDA2,NUPR1,ARHGAP22,KIAA0319,MTNR1B,CSRNP1,SSTR4,CYBB,SLC7A5,VAX2,KIRREL3,TRPV2,TNFAIP3,ISLR2,KLF2,KCNC2,NPY,ETNK2,GBA,PEMT,MIP,ADAMTSL2,SCN2B,ADM2,RFX8,SYN1,IL7R,GJA10,HM13,DUSP5,NPPB,INHBA,ZNF358,NFAM1,CHAC1,BTBD6,BEND6,CHRNB2,CYP4A11,ASTN2,CABLES1,DACT2,PITPNM1,DRD1,TCTA,TNFRSF11A,CDK5R2,LRRTM1,PCSK1,BAD,NCS1,COL8A2,MAL2,CDHR1,ABCA3,CYP46A1,PPARG,HPCAL4,ALK,ATP8A2,DMRT2,USH1G,HOXD11,LIMS2,ADORA1,RET,IRF4,FBLL1,ACHE
GO:BP	phospholipase C-activating G protein-coupled receptor signaling pathway	GO:0007200	2.76712417267258e-8	7.557971351774658	GRP,ADRA1B,ADRA2A,DRD5,FFAR4,GPR83,HRH1,GALR2,OPRD1,CCKBR,ADRA1D,DRD2,TACR1,P2RY2,NTSR2,DRD1,HTR2A
GO:BP	regulation of cellular process	GO:0050794	2.9738263276481933e-8	7.526684398001834	UTS2,DBH,CXCL8,GAL,GRP,GPR50,EGR4,MMP1,FGF3,PRLHR,ADRA1B,NKX6-2,RELB,MC5R,SYK,TNFRSF9,VGF,TIMP4,OR2W3,IL31RA,ARHGAP36,RAMP1,NKX2-2,PHOX2A,ADRA2A,STYK1,EGR2,TRIM58,DRD5,NPFFR2,SUSD5,OSR2,BHLHA15,FFAR4,SLC18A3,TG,PTHLH,HTR6,GPNMB,RAMP3,P3H2,KCNK12,FAM83G,ATP10A,MMP8,GABRA5,GATA4,DRGX,GALR1,KL,CD28,COL8A1,IGFBP3,SRPX2,PTGER4,SOSTDC1,RBP4,REM1,LGI3,EGR3,PHF24,TPBGL,PTGER2,NR0B1,EGR1,ME1,MAFA,PTPRN,BCAN,CHRM4,KCNS2,TMEM119,SIX2,UBD,SERPINE1,SYT17,KCNK13,MSC,NPSR1,PAX1,C1QL2,MCOLN3,GPR83,RASAL3,GRM7,HRH1,KCNA1,GABRG3,KCNS1,BOK,CITED1,CARTPT,CNN1,GSC2,HMOX1,MAGEB17,ITPKA,NPBWR1,SV2C,GPR68,KCNK3,DYSF,ITGA11,KCNE4,GALR2,OPRD1,ADAP1,HOXD9,HAS1,TNFRSF12A,HTR7,GCH1,ADGRF4,IL32,BDNF,PALM3,SPTB,CRLF2,TRPM8,FOXL2,FOXE1,OSGIN1,SLC24A3,HAP1,ASCL2,CCKBR,SFRP5,FCMR,CCL2,EGFL6,ADRA1D,IL1RL2,ETS1,CCL18,PLCG2,SLC2A6,DRD2,OPRL1,IL15RA,IRX6,GPR137,TBX21,GSG1L,GZMB,CHRDL2,TACR1,KCNK4,ACTG2,S100B,SCG2,GALR3,PAK6,ASIC2,TRIB3,GJC2,SPHK1,STK32B,NRGN,NXPH3,CRP,DCLK3,P2RY2,TRIM29,NTSR2,MAOA,HTR5A,NQO1,NPW,ZNF771,BEGAIN,MYRIP,PDE2A,HOXC13,RAET1G,NGEF,CCL20,SPRED3,SKAP1,CACNA1S,HCRTR1,CABP1,ALDH1A2,RSPO4,RCAN1,OR2L13,LY6H,VEGFB,COLEC10,TAL1,PLEKHF1,BATF3,PHLDA2,PNMA5,GABRE,NUPR1,ARHGAP22,KIAA0319,KCNA5,MTNR1B,CSRNP1,SSTR4,CYBB,SLC7A5,VAX2,HEPACAM,TRPV2,TNFAIP3,ISLR2,SPOCD1,CHRNA6,DUSP26,KLF2,KCNC2,NPY,SYNPO,GBA,MGLL,HTR1E,MMP7,CTSD,MIP,ADAMTSL2,CCL5,SCN2B,ADM2,TSSK1B,SLC6A9,RFX8,PSEN2,PRKAR1B,RHBDD2,KCNK15,SYN1,SQSTM1,GPR45,UBASH3A,RIN3,RASL10B,IL7R,MCHR1,DUSP5,NPPB,OASL,CALY,MAPK8IP1,INHBA,ZNF358,ABCG5,ZNF860,NFAM1,CHAC1,FOXD4L1,ATP6V0B,MLC1,KRT18,BEND6,LY6E,DBNDD1,CHRNB2,PALM2,ATP6V0C,HRH2,ASTN2,CABLES1,TEX261,NENF,STK32C,LAMP3,DACT2,ZNF469,PITPNM1,DRD1,DOC2B,RASGEF1C,TCTA,MS4A2,HRAS,LRFN2,REEP2,ADGRA1,TNFRSF11A,CDK5R2,LRRTM1,SYT5,PCSK1,SYT9,NPB,CYGB,PLA2G4C,BAD,NCS1,KCNIP1,TMEM198,IRAK1,ABCA3,ATP6V0D1,NXPH4,FIBP,CYP46A1,PPARG,ALK,CAMK2N2,ATP8A2,DMRT2,USH1G,ATP6V0A1,GABRQ,HOXD11,HTR2A,RASGEF1A,LIMS2,ADORA1,RGS6,IRAK2,TMEFF2,TEC,RET,SNCB,AMER3,TICAM1,IRF4,PARD6A,FBLL1,ACHE,ABLIM3,HCN2,QRFP,NOXO1,SYP,FOXD4
GO:BP	regulation of monoatomic ion transport	GO:0043269	4.9075575560051285e-8	7.309134598277351	GAL,GRP,ADRA2A,RAMP3,KCNK12,REM1,KCNS2,KCNK13,NPSR1,KCNA1,KCNS1,KCNK3,KCNE4,GALR2,OPRD1,HAP1,CCL2,PLCG2,DRD2,OPRL1,KCNK4,ASIC2,GJC2,CACNA1S,CABP1,GABRE,KCNA5,MTNR1B,CYBB,TRPV2,KCNC2,CCL5,SCN2B,SLC6A9,PSEN2,KCNK15,MCHR1,DRD1,KCNIP1,HTR2A,ADORA1,HCN2

GO:BP	regulation of secretion by cell	GO:1903530	6.16505741895544e-8	7.210062874198926	GAL,GRP,SYK,ADRA2A,FFAR4,SLC18A3,GALR1,RBP4,LGI3,SYT17,GRM7,CARTPT,SV2C,GPR68,DYSF,FOXL2,HAP1,DRD2,TACR1,MYRIP,KCNA5,MTNR1B,CHRNA6,CCL5,SYN1,RASL10B,INHBA,CHRNB2,DOC2B,TNFRSF11A,CDK5R2,SYT5,PCSK1,SYT9,BAD,PPARG,HTR2A,ADORA1,PARD6A,ACHE,SYP
GO:BP	system development	GO:0048731	1.5462302234734489e-7	6.810725842021693	CXCL8,FGF3,NKX6-2,SYK,VGF,TH,RAMP1,NKX2-2,PHOX2A,EGR2,OSR2,BHLHA15,TG,PTHLH,HTR6,GPNMB,GABRA5,GATA4,DRGX,MMP19,CD28,COL8A1,SRPX2,PTGER4,RBP4,EGR3,NR0B1,EGR1,PTPRN,BCAN,TMEM119,SIX2,SERPINE1,SYT17,MSC,PAX1,C1QL2,MCOLN3,SLC6A17,AQP5,TMEM132E,GRM7,KCNA1,BOK,CITED1,HMOX1,ITPKA,GPR68,MALL,GALR2,HOXD9,TNFRSF12A,BDNF,FOXL2,FOXE1,HAP1,ASCL2,CCKBR,SFRP5,CCL2,ETS1,DRD2,IL15RA,IRX6,TBX21,CHRDL2,S100B,SCG2,PAK6,ASIC2,GJC2,SPHK1,MYH7,NRGN,HSPB7,HTR5A,BFSP1,PDE2A,NGEF,SPRED3,CACNA1S,ALDH1A2,LY6H,VEGFB,TAL1,NUPR1,ARHGAP22,KIAA0319,MTNR1B,CSRNP1,SSTR4,CYBB,SLC7A5,VAX2,KIRREL3,TRPV2,TNFAIP3,ISLR2,KLF2,KCNC2,NPY,GBA,MIP,ADAMTSL2,SCN2B,ADM2,RFX8,SYN1,IL7R,GJA10,NPPB,INHBA,ZNF358,CHAC1,BTBD6,BEND6,CHRNB2,CYP4A11,ASTN2,CABLES1,DACT2,PITPNM1,DRD1,TNFRSF11A,CDK5R2,LRRTM1,PCSK1,BAD,NCS1,COL8A2,MAL2,CDHR1,ABCA3,CYP46A1,PPARG,HPCAL4,ALK,ATP8A2,DMRT2,USH1G,HOXD11,LIMS2,ADORA1,RET,ACHE
GO:BP	regulation of potassium ion transport	GO:0043266	1.607135377850091e-7	6.793947538683493	GAL,GRP,ADRA2A,KCNS2,KCNA1,KCNS1,KCNK3,KCNE4,GALR2,DRD2,KCNA5,MTNR1B,KCNC2,DRD1,KCNIP1,HTR2A,ADORA1
GO:BP	signal release	GO:0023061	1.61467463472765e-7	6.791914977096473	GAL,GRP,SYK,VGF,ADRA2A,FFAR4,SLC18A3,GALR1,RBP4,MAFA,PTPRN,SYT17,CARTPT,SV2C,GPR68,DYSF,FOXL2,DRD2,TACR1,MYRIP,KCNA5,MTNR1B,CHRNA6,CCL5,SYN1,RASL10B,INHBA,CHRNB2,DOC2B,TNFRSF11A,SYT5,SYT9,BAD,PPARG,HTR2A,ADORA1,SYP
GO:BP	cellular response to chemical stimulus	GO:0070887	1.6557528007054472e-7	6.781004501671446	CXCL8,RELB,SYK,TH,IL31RA,RAMP1,ADRA2A,EGR2,DRD5,NPFFR2,BHLHA15,FFAR4,HTR6,UGT3A2,RAMP3,GATA4,PTGER4,EGR3,PTGER2,NR0B1,EGR1,PTPRN,CHRM4,SERPINE1,SYT17,MSC,AQP5,HRH1,KCNA1,BOK,HMOX1,ITPKA,GPR68,KCNK3,OPRD1,HTR7,GCH1,PALM3,CRLF2,CCL2,IL1RL2,CCL18,PLCG2,DRD2,IL15RA,TBX21,KCNK4,SCG2,ASIC2,TRIB3,SPHK1,EPHX1,P2RY2,HTR5A,NQO1,PDE2A,SRXN1,CCL20,CACNA1S,HCRTR1,ALDH1A2,LY6H,VEGFB,SSTR4,CYBB,SLC7A5,TNFAIP3,KLF2,KCNC2,AANAT,GBA,HTR1E,DGAT2,CTSD,CCL5,TXNRD2,RHBDD2,SQSTM1,RIN3,AKR1B10,IL7R,OASL,CALY,INHBA,ATP1A3,MLC1,KRT18,CHRNB2,HRH2,GPX3,DRD1,HRAS,TNFRSF11A,SYT5,SYT9,CYGB,BAD,IRAK1,ATP6V0D1,CYP46A1,PPARG,ALK,HTR2A,IRAK2,CPNE7,RET,TICAM1,ACHE,HCN2,SYP
GO:BP	secretion	GO:0046903	2.0411172919937432e-7	6.690132038007093	GAL,GRP,SYK,VGF,ADRA2A,SYTL5,FFAR4,SLC18A3,GALR1,RBP4,LGI3,MAFA,PTPRN,SYT17,NPSR1,AQP5,GRM7,CARTPT,SV2C,GPR68,DYSF,FOXL2,HAP1,CCKBR,DRD2,RAB3IL1,TACR1,SCG2,P2RY2,MYRIP,KCNA5,MTNR1B,SSTR4,CHRNA6,CCL5,SYN1,RASL10B,NPPB,INHBA,CHRNB2,CYP4A11,HRH2,DOC2B,TNFRSF11A,CDK5R2,SYT5,PCSK1,SYT9,BAD,PPARG,HTR2A,ADORA1,PARD6A,ACHE,SYP
GO:BP	positive regulation of secretion	GO:0051047	2.1712909121799915e-7	6.663281985347784	GAL,GRP,SYK,FFAR4,SLC18A3,GALR1,RBP4,CARTPT,GPR68,FOXL2,DRD2,TACR1,P2RY2,MYRIP,SSTR4,RASL10B,NPPB,INHBA,CHRNB2,CYP4A11,DOC2B,TNFRSF11A,CDK5R2,PCSK1,BAD,PPARG,ADORA1,PARD6A,ACHE
GO:BP	positive regulation of cell communication	GO:0010647	3.9685037656533694e-7	6.401373203262667	GAL,GRP,ADRA1B,SYK,ADRA2A,TRIM58,FFAR4,SLC18A3,HTR6,GPNMB,RAMP3,MMP8,GATA4,GALR1,KL,CD28,IGFBP3,RBP4,UBD,NPSR1,BOK,CITED1,CARTPT,HMOX1,GPR68,TNFRSF12A,BDNF,CRLF2,FOXL2,HAP1,CCL2,CCL18,PLCG2,DRD2,GPR137,TACR1,S100B,SPHK1,MAOA,MYRIP,CCL20,HCRTR1,RSPO4,VEGFB,PLEKHF1,NUPR1,KIAA0319,MTNR1B,SSTR4,TNFAIP3,KLF2,GBA,CTSD,CCL5,PRKAR1B,SQSTM1,RASL10B,IL7R,OASL,MAPK8IP1,INHBA,NFAM1,ATP6V0C,NENF,DRD1,DOC2B,HRAS,TNFRSF11A,LRRTM1,BAD,TMEM198,IRAK1,CYP46A1,PPARG,HTR2A,RASGEF1A,LIMS2,ADORA1,RET,TICAM1
GO:BP	response to endogenous stimulus	GO:0009719	4.296584420209069e-7	6.366876650676863	CXCL8,GAL,FGF3,SYK,VGF,TIMP4,TH,RAMP1,NKX2-2,ADRA2A,EGR2,DRD5,NPFFR2,FFAR4,HTR6,RAMP3,FAM83G,GATA4,KL,MMP19,PTGER4,SOSTDC1,EGR3,PTGER2,NR0B1,EGR1,ME1,CHRM4,GPR83,HRH1,CITED1,OPRD1,HAS1,HTR7,BDNF,HAP1,CCL2,DRD2,CHRDL2,TACR1,TRIB3,SPHK1,P2RY2,HTR5A,NQO1,PDE2A,SPRED3,CACNA1S,HCRTR1,LY6H,VEGFB,SSTR4,CYBB,SLC7A5,KLF2,KCNC2,AANAT,GBA,HTR1E,CTSD,ADAMTSL2,CCL5,CALY,INHBA,ATP1A3,CHRNB2,HRH2,DRD1,HRAS,TNFRSF11A,PCSK1,ABCA3,FIBP,PPARG,ALK,HTR2A,TICAM1,ACHE,HCN2

GO:BP	cellular response to oxygen-containing compound	GO:1901701	5.103947135400249e-7	6.292093832508457	CXCL8,TH,ADRA2A,DRD5,HTR6,UGT3A2,RAMP3,GATA4,PTGER4,PTGER2,EGR1,PTPRN,CHRM4,SERPINE1,SYT17,HRH1,ITPKA,GPR68,HTR7,GCH1,CCL2,PLCG2,DRD2,KCNK4,TRIB3,SPHK1,P2RY2,HTR5A,NQO1,PDE2A,ALDH1A2,LY6H,CYBB,SLC7A5,TNFAIP3,KLF2,KCNC2,AANAT,HTR1E,DGAT2,CTSD,CCL5,SQSTM1,AKR1B10,CALY,INHBA,ATP1A3,MLC1,CHRNB2,HRH2,DRD1,HRAS,SYT5,SYT9,BAD,IRAK1,PPARG,ALK,HTR2A,IRAK2,CPNE7,RET,TICAM1,ACHE,HCN2
GO:BP	homeostatic process	GO:0042592	5.368239507861485e-7	6.270168116046555	SLC6A2,DBH,SYK,VGF,TH,IL31RA,ADRA2A,TRIM58,DRD5,BHLHA15,FFAR4,GATA4,KL,RBP4,EGR1,PTPRN,TMEM119,SERPINA3,CYB561,NPSR1,MCOLN3,KCNA1,BOK,CARTPT,HMOX1,GPR68,MALL,HKDC1,TRPM8,SLC24A3,HAP1,CCKBR,CCL2,ETS1,PLCG2,DRD2,GPR137,SLC4A3,P2RY2,NQO1,HCRTR1,TAL1,KCNA5,MTNR1B,TRPV2,TNFAIP3,TMPRSS3,KLF2,GBA,PEMT,DGAT2,CCL5,TXNRD2,SQSTM1,IL7R,NPPB,INHBA,ABCG5,ATP1A3,ATP6V0B,PNPLA1,ATP2B3,ATP6V0C,CYP4A11,DRD1,TNFRSF11A,BAD,CDHR1,ABCA3,ATP6V0D1,PPARG,ALK,USH1G,ATP6V0A1,HTR2A,ADORA1,IRF4,ACHE,PQLC2
GO:BP	animal organ development	GO:0048513	5.944225172907438e-7	6.225904747447525	KRT80,CXCL8,GAL,FGF3,NKX6-2,SYK,VGF,TH,NKX2-2,KRT75,TMIE,PHOX2A,EGR2,OSR2,BHLHA15,TG,PTHLH,HTR6,GABRA5,GATA4,DRGX,KL,MMP19,COL8A1,PTGER4,SOSTDC1,RBP4,EGR3,NR0B1,EGR1,PTPRN,BCAN,TMEM119,SIX2,SERPINE1,MSC,PAX1,MCOLN3,SLC6A17,AQP5,TMEM132E,KCNA1,BOK,CITED1,GPR68,KCNK3,ITGA11,HOXD9,FOXL2,FOXE1,SLC24A3,HAP1,ASCL2,CCKBR,KRT86,CCL2,DRD2,CHRDL2,ACTG2,SPHK1,MYH7,HSPB7,TLL2,HTR5A,BFSP1,PDE2A,HOXC13,SPRED3,CACNA1S,ALDH1A2,RCAN1,LY6H,VEGFB,TAL1,PHLDA2,NUPR1,MTNR1B,CSRNP1,SSTR4,SLC7A5,VAX2,KIRREL3,TNFAIP3,KLF2,KCNC2,NPY,ETNK2,GBA,DGAT2,MIP,ADAMTSL2,COL5A3,RFX8,SPRR2G,IL7R,NPPB,INHBA,GAMT,PNPLA1,CHRNB2,CYP4A11,DACT2,PITPNM1,DRD1,HRAS,TNFRSF11A,CDK5R2,PCSK1,BAD,COL8A2,ABCA3,PPARG,ALK,ATP8A2,USH1G,HOXD11,LIMS2,RET,ACHE
GO:BP	regulation of monoatomic ion transmembrane transport	GO:0034765	8.698816262287626e-7	6.060539842293305	GAL,GRP,ADRA2A,RAMP3,KCNK12,REM1,KCNS2,KCNK13,NPSR1,KCNA1,KCNS1,KCNK3,KCNE4,GALR2,HAP1,CCL2,PLCG2,DRD2,OPRL1,KCNK4,ASIC2,GJC2,CACNA1S,CABP1,KCNA5,MTNR1B,CYBB,KCNC2,SCN2B,SLC6A9,PSEN2,KCNK15,DRD1,KCNIP1,HCN2
GO:BP	adenylate cyclase-activating G protein-coupled receptor signaling pathway	GO:0007189	8.840114150119715e-7	6.053542127017806	ADRA1B,MC5R,RAMP1,ADRA2A,DRD5,PTHLH,RAMP3,GALR1,PTGER4,PTGER2,GALR2,ADGRF4,ADRA1D,DRD2,OPRL1,HTR5A,SSTR4,ADM2,DRD1
GO:BP	regulation of blood circulation	GO:1903522	8.900833290630518e-7	6.050569333064975	DBH,ADRA1B,TH,ADRA2A,GATA4,HRH1,KCNE4,GCH1,ADRA1D,DRD2,SLC4A3,TACR1,ASIC2,MYH7,HSPB7,KCNA5,MTNR1B,SCN2B,ADM2,ATP1A3,ATP2B3,HRH2,HTR2A,ADORA1,HCN2
GO:BP	response to organic cyclic compound	GO:0014070	9.950798758787695e-7	6.002142056680339	VGF,TH,NKX2-2,DRD5,HTR6,UGT3A2,RAMP3,KL,MMP19,PTGER2,NR0B1,EGR1,CHRM4,GPR83,HRH1,CITED1,HTR7,CCL2,DRD2,OPRL1,TACR1,EPHX1,P2RY2,HTR5A,NQO1,PDE2A,CACNA1S,ALDH1A2,SSTR4,CYBB,KLF2,KCNC2,AANAT,GBA,HTR1E,CCL5,CALY,INHBA,ATP1A3,MLC1,CHRNB2,HRH2,DRD1,TNFRSF11A,PCSK1,ABCA3,PPARG,ALK,HTR2A,ADORA1,TICAM1,HCN2
GO:BP	regulation of metal ion transport	GO:0010959	1.0012E-06	5.99946564	GAL,GRP,ADRA2A,RAMP3,REM1,KCNS2,NPSR1,KCNA1,KCNS1,KCNK3,KCNE4,GALR2,OPRD1,HAP1,CCL2,PLCG2,DRD2,OPRL1,GJC2,CABP1,KCNA5,MTNR1B,TRPV2,KCNC2,CCL5,SCN2B,PSEN2,MCHR1,DRD1,KCNIP1,HTR2A,ADORA1
GO:BP	export from cell	GO:0140352	1.1571E-06	5.936614243757755	GAL,GRP,SYK,VGF,ADRA2A,SYTL5,FFAR4,SLC18A3,GALR1,RBP4,LGI3,MAFA,PTPRN,SYT17,GRM7,CARTPT,SV2C,GPR68,DYSF,KCNE4,FOXL2,HAP1,DRD2,RAB3IL1,TACR1,SCG2,MYRIP,KCNA5,MTNR1B,CHRNA6,CCL5,SYN1,RASL10B,INHBA,ATP1A3,CHRNB2,ATP2B3,DOC2B,TNFRSF11A,CDK5R2,SYT5,PCSK1,SYT9,BAD,ABCA3,PPARG,HTR2A,ADORA1,PARD6A,ACHE,SYP
GO:BP	potassium ion transport	GO:0006813	1.846E-06	5.733779858540558	GAL,GRP,ADRA2A,KCNK12,KCNS2,KCNK13,KCNA1,KCNS1,KCNK3,KCNE4,GALR2,SLC24A3,DRD2,KCNK4,KCNA5,MTNR1B,KCNC2,KCNK15,ATP1A3,DRD1,KCNIP1,HTR2A,ADORA1,HCN2

GO:BP	positive regulation of signaling	GO:0023056	2.2612E-06	5.645669405208499	GAL,GRP,ADRA1B,SYK,ADRA2A,TRIM58,FFAR4,SLC18A3,HTR6,GPNMB,RAMP3,MMP8,GATA4,GALR1,KL,CD28,IGFBP3,RBP4,UBD,NPSR1,BOK,CITED1,CARTPT,HMOX1,GPR68,TNFRSF12A,BDNF,CRLF2,FOXL2,HAP1,CCL2,CCL18,PLCG2,DRD2,GPR137,TACR1,S100B,SPHK1,MAOA,MYRIP,CCL20,HCRTR1,RSPO4,VEGFB,PLEKHF1,NUPR1,KIAA0319,SSTR4,TNFAIP3,KLF2,CTSD,CCL5,PRKAR1B,SQSTM1,RASL10B,IL7R,OASL,MAPK8IP1,INHBA,NFAM1,ATP6V0C,NENF,DRD1,DOC2B,HRAS,TNFRSF11A,LRRTM1,BAD,TMEM198,IRAK1,CYP46A1,PPARG,HTR2A,RASGEF1A,LIMS2,ADORA1,RET,TICAM1
GO:BP	regulation of transmembrane transport	GO:0034762	3.3452E-06	5.4755760071317585	GAL,GRP,ADRA2A,FFAR4,RAMP3,KCNK12,REM1,KCNS2,KCNK13,NPSR1,KCNA1,KCNS1,KCNK3,KCNE4,GALR2,HAP1,CCL2,PLCG2,DRD2,OPRL1,GSG1L,KCNK4,ASIC2,TRIB3,GJC2,CACNA1S,CABP1,KCNA5,MTNR1B,CYBB,SLC7A5,KCNC2,SCN2B,SLC6A9,PSEN2,KCNK15,DRD1,KCNIP1,HCN2
GO:BP	regulation of cation channel activity	GO:2001257	6.2485E-06	5.204225045163535	GAL,GRP,REM1,KCNS2,KCNA1,KCNS1,KCNK3,KCNE4,GALR2,HAP1,PLCG2,DRD2,OPRL1,ASIC2,CABP1,MTNR1B,KCNC2,HCN2
GO:BP	secretion by cell	GO:0032940	6.7224E-06	5.172478508350629	GAL,GRP,SYK,VGF,ADRA2A,SYTL5,FFAR4,SLC18A3,GALR1,RBP4,LGI3,MAFA,PTPRN,SYT17,GRM7,CARTPT,SV2C,GPR68,DYSF,FOXL2,HAP1,DRD2,RAB3IL1,TACR1,SCG2,MYRIP,KCNA5,MTNR1B,CHRNA6,CCL5,SYN1,RASL10B,INHBA,CHRNB2,DOC2B,TNFRSF11A,CDK5R2,SYT5,PCSK1,SYT9,BAD,PPARG,HTR2A,ADORA1,PARD6A,ACHE,SYP
GO:BP	vasoconstriction	GO:0042310	7.3844E-06	5.131687137507708	DBH,ADRA1B,ADRA2A,DRD5,HRH1,HTR7,ADRA1D,TACR1,ASIC2,CRP,KCNA5,MTNR1B,HRH2,HTR2A
GO:BP	vasoconstriction	GO:1903351	1.332E-05	4.875493018057909	DRD5,HTR6,CHRM4,HRH1,HTR7,DRD2,HTR5A,HTR1E,CALY,HRH2,DRD1,ALK,HTR2A
GO:BP	regulation of hormone levels	GO:0010817	1.4368E-05	4.842607297545738	GAL,GRP,PRLHR,VGF,ADRA2A,FFAR4,TG,GALR1,RBP4,EGR1,MAFA,PTPRN,CARTPT,GPR68,FOXL2,FOXE1,DRD2,TACR1,MYRIP,ALDH1A2,KCNA5,MTNR1B,SLC7A5,AANAT,DGAT2,CCL5,AKR1B10,RASL10B,INHBA,DOC2B,PCSK1,BAD,CYP46A1,PPARG,ADORA1,DHCR7
GO:BP	response to abiotic stimulus	GO:0009628	1.5813E-05	4.800987657007976	DBH,MMP1,RELB,VGF,TH,NGB,NPFFR2,GATA4,DRGX,PTGER4,PHF24,EGR1,AQP5,HRH1,KCNA1,HMOX1,GPR68,KCNK3,OPRD1,TRPM8,ASCL2,DRD2,TACR1,KCNK4,ASIC2,NQO1,PDE2A,VEGFB,KIAA0319,KCNA5,CYBB,SLC7A5,TRPV2,KCNC2,AANAT,GBA,SCN2B,TXNRD2,PSEN2,GJA10,ABCG5,MLC1,CHRNB2,PITPNM1,DRD1,HRAS,TNFRSF11A,CYGB,BAD,IRAK1,ATP6V0D1,PPARG,ATP8A2,HTR2A,ADORA1,TMEM120A
GO:BP	response to dopamine	GO:1903350	1.5883E-05	4.799054773514997	DRD5,HTR6,CHRM4,HRH1,HTR7,DRD2,HTR5A,HTR1E,CALY,HRH2,DRD1,ALK,HTR2A
GO:BP	monoamine transport	GO:0015844	1.6423E-05	4.784559449948816	SLC6A2,SYK,ADRA2A,SLC18A3,SYT17,CARTPT,DRD2,CHRNA6,CHRNB2,DRD1,SYT5,SYT9,HTR2A,SLC22A3
GO:BP	regulation of membrane potential	GO:0042391	2.5282E-05	4.597185819142546	KCNK12,GABRA5,KCNK13,KCNA1,GABRG3,BOK,KCNK3,KCNE4,OPRD1,DRD2,SLC4A3,TACR1,KCNK4,ASIC2,NTSR2,GABRE,KCNA5,MTNR1B,CHRNA6,KCNC2,GBA,SCN2B,PRKAR1B,KCNK15,ATP1A3,CHRNB2,DRD1,BAD,GABRQ,ADORA1,HCN2
GO:BP	amine transport	GO:0015837	3.7959E-05	4.420687963641657	TH,ADRA2A,SLC18A3,SYT17,GRM7,CARTPT,DRD2,SLC7A5,CHRNA6,CHRNB2,DRD1,SYT5,SYT9,HTR2A,ADORA1
GO:BP	G protein-coupled serotonin receptor signaling pathway	GO:0098664	4.728E-05	4.325324048716431	HTR6,CHRM4,HRH1,HTR7,HTR5A,HTR1E,HRH2,HTR2A
GO:BP	regulation of monoatomic ion transmembrane transporter activity	GO:0032412	6.7393E-05	4.171385753581016	GAL,GRP,ADRA2A,REM1,KCNS2,KCNA1,KCNS1,KCNK3,KCNE4,GALR2,HAP1,CCL2,PLCG2,DRD2,OPRL1,GSG1L,ASIC2,CABP1,MTNR1B,KCNC2,SCN2B,SLC6A9,HCN2
GO:BP	regulation of amine transport	GO:0051952	6.9408E-05	4.158591145957403	ADRA2A,SLC18A3,SYT17,GRM7,CARTPT,DRD2,SLC7A5,CHRNA6,CHRNB2,DRD1,SYT5,SYT9,HTR2A,ADORA1

GO:BP	cellular response to organic substance	GO:0071310	7.1462E-05	4.145922263166681	CXCL8,SYK,TH,IL31RA,RAMP1,ADRA2A,EGR2,DRD5,NPFFR2,BHLHA15,FFAR4,HTR6,UGT3A2,RAMP3,GATA4,PTGER4,PTGER2,NR0B1,EGR1,PTPRN,CHRM4,SERPINE1,MSC,HRH1,BOK,GPR68,HTR7,PALM3,CRLF2,CCL2,IL1RL2,CCL18,PLCG2,DRD2,IL15RA,TBX21,KCNK4,TRIB3,SPHK1,P2RY2,HTR5A,PDE2A,CCL20,CACNA1S,HCRTR1,ALDH1A2,LY6H,SSTR4,CYBB,SLC7A5,TNFAIP3,KLF2,AANAT,GBA,HTR1E,DGAT2,CTSD,CCL5,RHBDD2,IL7R,OASL,CALY,INHBA,ATP1A3,MLC1,KRT18,CHRNB2,HRH2,DRD1,HRAS,TNFRSF11A,BAD,IRAK1,PPARG,ALK,HTR2A,IRAK2,RET,TICAM1,ACHE,HCN2,SYP
GO:BP	regulation of monoatomic cation transmembrane transport	GO:1904062	7.8589E-05	4.104635978847422	GAL,GRP,ADRA2A,RAMP3,REM1,KCNS2,NPSR1,KCNA1,KCNS1,KCNK3,KCNE4,GALR2,HAP1,PLCG2,DRD2,OPRL1,ASIC2,GJC2,CABP1,MTNR1B,KCNC2,SCN2B,PSEN2,DRD1,KCNIP1,HCN2
GO:BP	chemical homeostasis	GO:0048878	8.1566E-05	4.088492948386269	DBH,VGF,TH,ADRA2A,DRD5,BHLHA15,GATA4,KL,RBP4,PTPRN,CYB561,NPSR1,MCOLN3,KCNA1,BOK,CARTPT,HMOX1,GPR68,MALL,HKDC1,TRPM8,SLC24A3,HAP1,CCKBR,PLCG2,DRD2,SLC4A3,P2RY2,HCRTR1,KCNA5,MTNR1B,TMPRSS3,DGAT2,CCL5,NPPB,ABCG5,ATP1A3,ATP6V0B,PNPLA1,ATP2B3,ATP6V0C,CYP4A11,DRD1,BAD,ABCA3,ATP6V0D1,PPARG,ATP6V0A1,HTR2A,ADORA1,PQLC2
GO:BP	positive regulation of secretion by cell	GO:1903532	8.3972E-05	4.0758639538417425	GAL,GRP,SYK,FFAR4,SLC18A3,GALR1,RBP4,CARTPT,GPR68,FOXL2,DRD2,TACR1,MYRIP,RASL10B,INHBA,CHRNB2,DOC2B,TNFRSF11A,CDK5R2,PCSK1,BAD,PPARG,PARD6A,ACHE
GO:BP	organic substance transport	GO:0071702	8.6322E-05	4.0638788017169425	SLC6A2,FOLR3,GAL,GRP,SYK,VGF,NEURL3,TH,RAMP1,ADRA2A,EGR2,BHLHA15,SYTL5,FFAR4,SLC18A3,RAMP3,ATP10A,APOL6,GALR1,DMBT1,SLC5A10,RBP4,REM1,MAFA,PTPRN,GOLT1A,SIX2,SYT17,NPSR1,SLC10A4,MCOLN3,SLC6A17,AQP5,GRM7,SLC7A4,CARTPT,GPR68,APOF,OPRD1,B4GALT1-AS1,TRPM8,SLC24A3,HAP1,CCL2,SLC6A11,PLCG2,SLC2A6,DRD2,SLC1A4,OPRL1,RAB3IL1,SLC4A3,GZMB,SCG2,TRIB3,GJC2,RTP1,MYRIP,CACNA1S,CABP1,KCNA5,MTNR1B,SSTR4,SLC7A5,TRPV2,CHRNA6,SLC5A1,MIP,CCL5,SLC6A9,PSEN2,SQSTM1,RASL10B,MCHR1,HM13,INHBA,ABCG5,MLC1,KRT18,SLC6A12,CHRNB2,ATP2B3,CYP4A11,ASTN2,PITPNM1,DRD1,DOC2B,HRAS,REEP2,SLC37A1,SPRN,TNFRSF11A,SYT5,PCSK1,SYT9,BAD,NCS1,KIF17,ABCA3,PPARG,ATP8A2,SLC36A1,ZDHHC14,HTR2A,ADORA1,SLC45A1,PARD6A,ACHE,ABLIM3,SLC22A3,PQLC2
GO:BP	regulation of signal transduction	GO:0009966	9.8242E-05	4.007703102826818	DBH,CXCL8,GRP,ADRA1B,SYK,RAMP1,ADRA2A,TRIM58,NPFFR2,FFAR4,HTR6,GPNMB,RAMP3,MMP8,GATA4,KL,CD28,IGFBP3,SOSTDC1,PHF24,TPBGL,NR0B1,EGR1,UBD,SERPINE1,NPSR1,RASAL3,BOK,CITED1,CARTPT,HMOX1,TNFRSF12A,BDNF,PALM3,CRLF2,HAP1,SFRP5,CCL2,CCL18,PLCG2,DRD2,OPRL1,GPR137,GSG1L,CHRDL2,S100B,SCG2,PAK6,SPHK1,MAOA,BEGAIN,PDE2A,NGEF,CCL20,SPRED3,HCRTR1,RSPO4,RCAN1,VEGFB,PLEKHF1,PHLDA2,NUPR1,ARHGAP22,KIAA0319,MTNR1B,SSTR4,TNFAIP3,DUSP26,KLF2,GBA,MGLL,CTSD,ADAMTSL2,CCL5,TSSK1B,SLC6A9,RFX8,PRKAR1B,SQSTM1,UBASH3A,IL7R,DUSP5,OASL,MAPK8IP1,INHBA,NFAM1,CHAC1,BEND6,ATP6V0C,NENF,DACT2,HRAS,TNFRSF11A,BAD,TMEM198,IRAK1,PPARG,ALK,HTR2A,RASGEF1A,LIMS2,ADORA1,RGS6,IRAK2,RET,AMER3,TICAM1,IRF4,SYP
GO:BP	regulation of transporter activity	GO:0032409	0.000109	3.962576597237861	GAL,GRP,ADRA2A,REM1,KCNS2,KCNA1,KCNS1,KCNK3,KCNE4,GALR2,HAP1,CCL2,PLCG2,DRD2,OPRL1,GSG1L,ASIC2,CABP1,MTNR1B,KCNC2,SCN2B,SLC6A9,PPARG,HCN2
GO:BP	cellular response to catecholamine stimulus	GO:0071870	0.0001234	3.9086861930624766	DRD5,HTR6,CHRM4,HRH1,HTR7,DRD2,HTR5A,HTR1E,CALY,HRH2,DRD1,ALK,HTR2A
GO:BP	cellular response to monoamine stimulus	GO:0071868	0.0001234	3.9086861930624766	DRD5,HTR6,CHRM4,HRH1,HTR7,DRD2,HTR5A,HTR1E,CALY,HRH2,DRD1,ALK,HTR2A
GO:BP	regulation of transmembrane transporter activity	GO:0022898	0.00015266	3.8162654102097058	GAL,GRP,ADRA2A,REM1,KCNS2,KCNA1,KCNS1,KCNK3,KCNE4,GALR2,HAP1,CCL2,PLCG2,DRD2,OPRL1,GSG1L,ASIC2,CABP1,MTNR1B,KCNC2,SCN2B,SLC6A9,HCN2
GO:BP	modulation of chemical synaptic transmission	GO:0050804	0.00016173	3.791209895895548	VGF,ADRA2A,EGR2,DRD5,SLC18A3,PHF24,GRM7,HRH1,ITPKA,SV2C,DYSF,HAP1,CCL2,DRD2,TACR1,S100B,BEGAIN,CHRNA6,SLC6A9,PRKAR1B,SYN1,SQSTM1,CHRNB2,DRD1,HRAS,LRFN2,LRRTM1,CYP46A1,HTR2A,ADORA1,ACHE,SYP

GO:BP	regulation of trans-synaptic signaling	GO:0099177	0.00016941	3.7710492707543137	VGF,ADRA2A,EGR2,DRD5,SLC18A3,PHF24,GRM7,HRH1,ITPKA,SV2C,DYSF,HAP1,CCL2,DRD2,TACR1,S100B,BEGAIN,CHRNA6,SLC6A9,PRKAR1B,SYN1,SQSTM1,CHRNB2,DRD1,HRAS,LRFN2,LRRTM1,CYP46A1,HTR2A,ADORA1,ACHE,SYP
GO:BP	hormone transport	GO:0009914	0.00018238	3.7390264714309276	GAL,GRP,VGF,ADRA2A,FFAR4,GALR1,RBP4,MAFA,PTPRN,CARTPT,GPR68,FOXL2,DRD2,TACR1,MYRIP,KCNA5,MTNR1B,SLC7A5,CCL5,RASL10B,INHBA,DOC2B,BAD,PPARG,ADORA1
GO:BP	response to monoamine	GO:0071867	0.00018769	3.726569077866451	DRD5,HTR6,CHRM4,HRH1,HTR7,DRD2,HTR5A,HTR1E,CALY,HRH2,DRD1,ALK,HTR2A
GO:BP	response to catecholamine	GO:0071869	0.00018769	3.726569077866451	DRD5,HTR6,CHRM4,HRH1,HTR7,DRD2,HTR5A,HTR1E,CALY,HRH2,DRD1,ALK,HTR2A
GO:BP	catecholamine transport	GO:0051937	0.00024567	3.6096461811193965	SLC6A2,ADRA2A,SYT17,CARTPT,DRD2,CHRNA6,CHRNB2,DRD1,SYT5,SYT9,HTR2A,SLC22A3
GO:BP	serotonin receptor signaling pathway	GO:0007210	0.00027192	3.5655616303696713	HTR6,CHRM4,HRH1,HTR7,HTR5A,HTR1E,HRH2,HTR2A
GO:BP	positive regulation of MAPK cascade	GO:0043410	0.00027847	3.5552164782014652	ADRA1B,SYK,ADRA2A,FFAR4,GPNMB,RAMP3,MMP8,GATA4,KL,IGFBP3,NPSR1,CARTPT,CCL2,CCL18,PLCG2,DRD2,SPHK1,CCL20,HCRTR1,VEGFB,SSTR4,CCL5,MAPK8IP1,INHBA,NENF,HRAS,TNFRSF11A,IRAK1,HTR2A,ADORA1,RET
GO:BP	regulation of MAPK cascade	GO:0043408	0.0002822	3.549438697820748	ADRA1B,SYK,ADRA2A,NPFFR2,FFAR4,GPNMB,RAMP3,MMP8,GATA4,KL,IGFBP3,NPSR1,CARTPT,CCL2,CCL18,PLCG2,DRD2,PAK6,SPHK1,CCL20,SPRED3,HCRTR1,VEGFB,SSTR4,DUSP26,GBA,CCL5,DUSP5,MAPK8IP1,INHBA,NENF,HRAS,TNFRSF11A,IRAK1,PPARG,HTR2A,ADORA1,RET
GO:BP	regulation of vasoconstriction	GO:0019229	0.00028841	3.53998574	DBH,ADRA1B,ADRA2A,HRH1,ADRA1D,TACR1,ASIC2,KCNA5,MTNR1B,HRH2,HTR2A
GO:BP	hormone secretion	GO:0046879	0.00037463	3.4263999646423673	GAL,GRP,VGF,ADRA2A,FFAR4,GALR1,RBP4,MAFA,PTPRN,CARTPT,GPR68,FOXL2,DRD2,TACR1,MYRIP,KCNA5,MTNR1B,CCL5,RASL10B,INHBA,DOC2B,BAD,PPARG,ADORA1
GO:BP	regulation of blood pressure	GO:0008217	0.00039965	3.3983196730265535	UTS2,ADRA1B,DRD5,KL,CARTPT,GCH1,DRD2,OPRL1,TACR1,ASIC2,NPY,ADM2,RASL10B,NPPB,CYP4A11,PPARG,ADORA1,QRFP
GO:BP	positive regulation of biological process	GO:0048518	0.00040259	3.395135459702039	DBH,CXCL8,GAL,GRP,EGR4,MMP1,FGF3,ADRA1B,NKX6-2,RELB,SYK,IL31RA,RAMP1,NKX2-2,PHOX2A,ADRA2A,EGR2,TRIM58,OSR2,BHLHA15,FFAR4,SLC18A3,PTHLH,HTR6,GPNMB,RAMP3,ATP10A,MMP8,GATA4,GALR1,KL,CD28,COL8A1,IGFBP3,SRPX2,PTGER4,RBP4,EGR3,EGR1,MAFA,PTPRN,BCAN,TMEM119,SIX2,UBD,SERPINE1,SYT17,NPSR1,PAX1,RASAL3,HRH1,KCNA1,BOK,CITED1,CARTPT,HMOX1,ITPKA,GPR68,KCNK3,GALR2,OPRD1,HOXD9,TNFRSF12A,GCH1,IL32,BDNF,CRLF2,TRPM8,FOXL2,FOXE1,SLC24A3,HAP1,ASCL2,CCKBR,CCL2,EGFL6,ADRA1D,IL1RL2,ETS1,CCL18,PLCG2,DRD2,IL15RA,IRX6,GPR137,TBX21,GZMB,TACR1,ACTG2,S100B,SCG2,GALR3,ASIC2,TRIB3,GJC2,SPHK1,CRP,P2RY2,MAOA,NQO1,MYRIP,PDE2A,HOXC13,RAET1G,CCL20,SKAP1,CACNA1S,HCRTR1,ALDH1A2,RSPO4,VEGFB,COLEC10,TAL1,PLEKHF1,PHLDA2,PNMA5,NUPR1,KIAA0319,KCNA5,MTNR1B,CSRNP1,SSTR4,CYBB,SLC7A5,TRPV2,TNFAIP3,ISLR2,DUSP26,KLF2,KCNC2,NPY,SYNPO,GBA,PEMT,MMP7,CTSD,MIP,CCL5,ADM2,SLC6A9,PRKAR1B,SQSTM1,UBASH3A,RASL10B,IL7R,MCHR1,NPPB,OASL,CALY,MAPK8IP1,INHBA,NFAM1,MLC1,BEND6,CHRNB2,ATP6V0C,CYP4A11,HRH2,TEX261,NENF,LAMP3,DRD1,DOC2B,HRAS,TNFRSF11A,CDK5R2,LRRTM1,PCSK1,SYT9,BAD,TMEM198,IRAK1,ABCA3,CYP46A1,PPARG,ALK,ATP8A2,DMRT2,HTR2A,RASGEF1A,LIMS2,ADORA1,IRAK2,TEC,RET,TICAM1,IRF4,PARD6A,ACHE,ABLIM3
GO:BP	response to organonitrogen compound	GO:0010243	0.00041427	3.3827164679756407	DBH,GAL,SYK,VGF,TH,EGR2,DRD5,HTR6,RAMP3,KL,MMP19,EGR1,CHRM4,HRH1,CITED1,HTR7,DRD2,TRIB3,P2RY2,HTR5A,NQO1,PDE2A,CACNA1S,LY6H,CYBB,SLC7A5,TNFAIP3,KLF2,KCNC2,AANAT,HTR1E,CTSD,RHBDD2,HM13,CALY,INHBA,ATP1A3,CHRNB2,HRH2,DRD1,HRAS,TNFRSF11A,PCSK1,PPARG,ALK,HTR2A,ADORA1,TICAM1,ACHE,HCN2
GO:BP	MAPK cascade	GO:0000165	0.00045289	3.3440031333696245	ADRA1B,SYK,IL31RA,ADRA2A,NPFFR2,FFAR4,GPNMB,RAMP3,MMP8,GATA4,KL,IGFBP3,PTGER4,NPSR1,CARTPT,CCL2,CCL18,PLCG2,DRD2,SCG2,PAK6,SPHK1,CCL20,SPRED3,HCRTR1,VEGFB,SSTR4,DUSP26,GBA,CCL5,DUSP5,MAPK8IP1,INHBA,NENF,HRAS,TNFRSF11A,IRAK1,PPARG,HTR2A,ADORA1,RET

GO:BP	dopamine transport	GO:0015872	0.00073129	3.1359116738001513	SLC6A2,SYT17,DRD2,CHRNA6,CHRNB2,DRD1,SYT5,SYT9,HTR2A,SLC22A3
GO:BP	regulation of hormone secretion	GO:0046883	0.00079692	3.098584526614217	GAL,GRP,ADRA2A,FFAR4,GALR1,RBP4,CARTPT,GPR68,FOXL2,DRD2,TACR1,MYRIP,KCNA5,MTNR1B,CCL5,RASL10B,INHBA,DOC2B,BAD,PPARG,ADORA1
GO:BP	potassium ion transmembrane transport	GO:0071805	0.00082414	3.083998260343302	GAL,GRP,KCNK12,KCNS2,KCNK13,KCNA1,KCNS1,KCNK3,KCNE4,GALR2,SLC24A3,KCNK4,KCNA5,MTNR1B,KCNC2,KCNK15,ATP1A3,KCNIP1,HCN2
GO:BP	positive regulation of signal transduction	GO:0009967	0.0009403	3.0267335120341663	GRP,ADRA1B,SYK,ADRA2A,TRIM58,FFAR4,HTR6,GPNMB,RAMP3,MMP8,GATA4,KL,CD28,IGFBP3,UBD,NPSR1,BOK,CITED1,CARTPT,HMOX1,TNFRSF12A,BDNF,CRLF2,HAP1,CCL2,CCL18,PLCG2,DRD2,GPR137,S100B,SPHK1,MAOA,CCL20,HCRTR1,RSPO4,VEGFB,PLEKHF1,NUPR1,KIAA0319,SSTR4,TNFAIP3,KLF2,CTSD,CCL5,PRKAR1B,IL7R,OASL,MAPK8IP1,INHBA,NFAM1,ATP6V0C,NENF,HRAS,TNFRSF11A,BAD,TMEM198,IRAK1,PPARG,HTR2A,RASGEF1A,LIMS2,ADORA1,RET,TICAM1
GO:BP	response to lipid	GO:0033993	0.00095533	3.019847831116532	CXCL8,SYK,TH,NKX2-2,RAMP3,GATA4,KL,PTGER4,RBP4,PTGER2,NR0B1,SERPINE1,GPR83,CITED1,GCH1,PALM3,CCL2,PLCG2,OPRL1,TACR1,KCNK4,HTR5A,NQO1,ALDH1A2,SSTR4,CYBB,SLC7A5,TNFAIP3,AANAT,GBA,DGAT2,CCL5,INHBA,ATP1A3,MLC1,GPX3,TNFRSF11A,PCSK1,BAD,IRAK1,ABCA3,PPARG,IRAK2,RET,TICAM1
GO:BP	organic hydroxy compound transport	GO:0015850	0.00098178	3.007986703447819	SLC6A2,GAL,SYK,ADRA2A,SLC18A3,GALR1,RBP4,SYT17,SLC10A4,CARTPT,DRD2,CHRNA6,SLC5A1,ABCG5,CHRNB2,DRD1,SYT5,SYT9,ABCA3,PPARG,HTR2A,SLC22A3
GO:BP	positive regulation of cellular process	GO:0048522	0.00105431	2.9770299106411224	CXCL8,GAL,GRP,EGR4,MMP1,FGF3,ADRA1B,NKX6-2,RELB,SYK,IL31RA,RAMP1,NKX2-2,PHOX2A,ADRA2A,EGR2,TRIM58,OSR2,BHLHA15,FFAR4,SLC18A3,PTHLH,HTR6,GPNMB,RAMP3,ATP10A,MMP8,GATA4,GALR1,KL,CD28,COL8A1,IGFBP3,SRPX2,PTGER4,RBP4,EGR3,EGR1,MAFA,PTPRN,BCAN,TMEM119,SIX2,UBD,SERPINE1,SYT17,NPSR1,PAX1,RASAL3,HRH1,KCNA1,BOK,CITED1,CARTPT,HMOX1,ITPKA,GPR68,KCNK3,GALR2,OPRD1,HOXD9,TNFRSF12A,IL32,BDNF,CRLF2,FOXL2,FOXE1,SLC24A3,HAP1,ASCL2,CCKBR,CCL2,EGFL6,ADRA1D,IL1RL2,ETS1,CCL18,PLCG2,DRD2,IL15RA,IRX6,GPR137,TBX21,GZMB,TACR1,ACTG2,S100B,SCG2,GALR3,ASIC2,GJC2,SPHK1,CRP,MAOA,NQO1,MYRIP,PDE2A,HOXC13,RAET1G,CCL20,SKAP1,HCRTR1,ALDH1A2,RSPO4,VEGFB,COLEC10,TAL1,PLEKHF1,PHLDA2,PNMA5,NUPR1,KIAA0319,KCNA5,MTNR1B,CSRNP1,SSTR4,CYBB,SLC7A5,TRPV2,TNFAIP3,ISLR2,DUSP26,KLF2,KCNC2,SYNPO,GBA,MMP7,CTSD,MIP,CCL5,ADM2,SLC6A9,PRKAR1B,SQSTM1,RASL10B,IL7R,OASL,CALY,MAPK8IP1,INHBA,NFAM1,MLC1,BEND6,CHRNB2,ATP6V0C,TEX261,NENF,LAMP3,DRD1,DOC2B,HRAS,TNFRSF11A,CDK5R2,LRRTM1,PCSK1,SYT9,BAD,TMEM198,IRAK1,ABCA3,CYP46A1,PPARG,ALK,ATP8A2,DMRT2,HTR2A,RASGEF1A,LIMS2,ADORA1,RET,TICAM1,IRF4,PARD6A,ACHE,ABLIM3
GO:BP	cellular response to endogenous stimulus	GO:0071495	0.00130835	2.883275664314995	CXCL8,FGF3,TH,RAMP1,ADRA2A,DRD5,NPFFR2,FFAR4,HTR6,RAMP3,FAM83G,GATA4,KL,PTGER4,SOSTDC1,EGR3,PTGER2,NR0B1,EGR1,CHRM4,HRH1,CITED1,OPRD1,HAS1,HTR7,BDNF,HAP1,CCL2,DRD2,CHRDL2,TRIB3,SPHK1,P2RY2,HTR5A,PDE2A,SPRED3,CACNA1S,HCRTR1,LY6H,VEGFB,SSTR4,CYBB,SLC7A5,KLF2,AANAT,HTR1E,CTSD,ADAMTSL2,CCL5,CALY,INHBA,ATP1A3,CHRNB2,HRH2,DRD1,HRAS,FIBP,PPARG,ALK,HTR2A,ACHE,HCN2
GO:BP	muscle contraction	GO:0006936	0.00151434	2.8197767667902327	UTS2,ADRA1B,MYH2,ADRA2A,GATA4,KCNA1,CNN1,KCNE4,GALR2,HTR7,DRD2,TACR1,SPHK1,MYH7,CACNA1S,VEGFB,KCNA5,SCN2B,GAMT,CHRNB2,DRD1,ATP8A2,HTR2A,ADORA1
GO:BP	neurotransmitter transport	GO:0006836	0.00159512	2.7972076671423194	SLC6A2,TH,SLC18A3,SLC6A17,SV2C,DYSF,SLC6A11,DRD2,SLC6A9,SYN1,SLC6A12,DRD1,DOC2B,SYT5,SYT9,GABRQ,SLC22A3,SYP
GO:BP	positive regulation of hormone secretion	GO:0046887	0.00163428	2.7866738205967625	GAL,GRP,FFAR4,GALR1,RBP4,GPR68,FOXL2,DRD2,TACR1,MYRIP,RASL10B,INHBA,DOC2B,BAD,PPARG
GO:BP	regulation of potassium ion transmembrane transporter activity	GO:1901016	0.00234094	2.6306103610702736	GAL,GRP,KCNS2,KCNA1,KCNS1,KCNK3,KCNE4,GALR2,MTNR1B,KCNC2

GO:BP	positive regulation of intracellular signal transduction	GO:1902533	0.00261922	2.5818278729092854	ADRA1B,SYK,ADRA2A,TRIM58,FFAR4,HTR6,GPNMB,RAMP3,MMP8,GATA4,KL,CD28,IGFBP3,UBD,NPSR1,BOK,CARTPT,HMOX1,CCL2,CCL18,PLCG2,DRD2,GPR137,S100B,SPHK1,CCL20,HCRTR1,VEGFB,PLEKHF1,NUPR1,KIAA0319,SSTR4,CCL5,MAPK8IP1,INHBA,NENF,HRAS,TNFRSF11A,BAD,IRAK1,PPARG,HTR2A,RASGEF1A,ADORA1,RET,TICAM1
GO:BP	adenylate cyclase-inhibiting G protein-coupled receptor signaling pathway	GO:0007193	0.00307915	2.5115689368455274	ADRA2A,CHRM4,GRM7,OPRD1,DRD2,OPRL1,HTR5A,PDE2A,HTR1E,MCHR1,ADORA1
GO:BP	response to nitrogen compound	GO:1901698	0.00320287	2.4944607223055124	DBH,GAL,SYK,VGF,TH,EGR2,DRD5,HTR6,RAMP3,KL,MMP19,EGR1,CHRM4,HRH1,CITED1,HTR7,DRD2,TRIB3,P2RY2,HTR5A,NQO1,PDE2A,CACNA1S,LY6H,CYBB,SLC7A5,TNFAIP3,KLF2,KCNC2,AANAT,HTR1E,CTSD,RHBDD2,HM13,CALY,INHBA,ATP1A3,CHRNB2,HRH2,DRD1,HRAS,TNFRSF11A,PCSK1,PPARG,ALK,HTR2A,ADORA1,TICAM1,ACHE,HCN2
GO:BP	anatomical structure morphogenesis	GO:0009653	0.00446988	2.3497043957741526	CXCL8,FGF3,SYK,TH,RAMP1,TMIE,PHOX2A,EGR2,OSR2,GPNMB,ATP10A,MMP8,GATA4,DRGX,MMP19,COL8A1,SRPX2,SOSTDC1,RBP4,EGR3,NR0B1,TMEM119,SIX2,SERPINE1,SYT17,PAX1,AQP5,CITED1,GSC2,HMOX1,ITPKA,HOXD9,TNFRSF12A,BDNF,PALM3,FOXL2,FOXE1,SFRP5,CCL2,ETS1,DRD2,SHROOM1,ACTG2,S100B,SCG2,PAK6,SPHK1,MYH7,HOXC13,NGEF,CACNA1S,ALDH1A2,LY6H,VEGFB,TAL1,PHLDA2,ARHGAP22,KIAA0319,CSRNP1,CYBB,VAX2,KIRREL3,TRPV2,TNFAIP3,ISLR2,KLF2,GBA,ADM2,IL7R,DUSP5,NPPB,INHBA,ZNF358,FOXD4L1,GAMT,KRT18,CHRNB2,PALM2,ASTN2,DACT2,TCTA,HRAS,CDK5R2,COL8A2,CDHR1,PPARG,ATP8A2,DMRT2,USH1G,HOXD11,CDH22,LIMS2,TMEFF2,RET,FOXD4
GO:BP	dopamine metabolic process	GO:0042417	0.00532927	2.273332001577892	DBH,TH,GCH1,DRD2,MAOA,CHRNB2,DRD1,SNCB
GO:BP	cell differentiation	GO:0030154	0.0055954	2.252168694025814	KRT80,FGF3,NKX6-2,RELB,SYK,TNFRSF9,TH,IL31RA,NKX2-2,KRT75,PHOX2A,EGR2,TRIM58,OSR2,BHLHA15,FFAR4,PTHLH,MMP8,GABRA5,GATA4,DRGX,DMBT1,MMP19,CD28,COL8A1,IGFBP3,PTGER4,SOSTDC1,EGR3,NR0B1,EGR1,BCAN,TMEM119,SIX2,UBD,SERPINE1,SYT17,PAX1,MCOLN3,TMEM132E,GRM7,KCNA1,BOK,CITED1,CARTPT,ITPKA,GPR68,ITGA11,GALR2,HOXD9,TNFRSF12A,BDNF,KRT15,FOXL2,FOXE1,OSGIN1,HAP1,ASCL2,SFRP5,KRT86,CCL2,EGFL6,IL1RL2,ETS1,PLCG2,DRD2,IL15RA,IRX6,GPR137,TBX21,CHRDL2,S100B,PAK6,TRIB3,GJC2,MYH7,OOSP2,CRP,TLL2,BFSP1,PDE2A,NGEF,SPRED3,CACNA1S,ALDH1A2,RCAN1,TAL1,BATF3,NUPR1,ARHGAP22,KIAA0319,RIMBP3,SLC7A5,VAX2,KIRREL3,TRPV2,ISLR2,SPOCD1,KLF2,NPY,GBA,TSSK1B,SYN1,SQSTM1,SPRR2G,IL7R,INHBA,NFAM1,CHAC1,BTBD6,FOXD4L1,BEND6,PNPLA1,CHRNB2,ASTN2,DACT2,DRD1,TCTA,TNFRSF11A,CDK5R2,PCSK1,BAD,NCS1,CDHR1,PPARG,ALK,ATP8A2,USH1G,TPGS1,HTR2A,KRT37,TMEFF2,RET,IRF4,ACHE,TMEM120A,FOXD4
GO:BP	cellular developmental process	GO:0048869	0.00566799	2.2465709097585416	KRT80,FGF3,NKX6-2,RELB,SYK,TNFRSF9,TH,IL31RA,NKX2-2,KRT75,PHOX2A,EGR2,TRIM58,OSR2,BHLHA15,FFAR4,PTHLH,MMP8,GABRA5,GATA4,DRGX,DMBT1,MMP19,CD28,COL8A1,IGFBP3,PTGER4,SOSTDC1,EGR3,NR0B1,EGR1,BCAN,TMEM119,SIX2,UBD,SERPINE1,SYT17,PAX1,MCOLN3,TMEM132E,GRM7,KCNA1,BOK,CITED1,CARTPT,ITPKA,GPR68,ITGA11,GALR2,HOXD9,TNFRSF12A,BDNF,KRT15,FOXL2,FOXE1,OSGIN1,HAP1,ASCL2,SFRP5,KRT86,CCL2,EGFL6,IL1RL2,ETS1,PLCG2,DRD2,IL15RA,IRX6,GPR137,TBX21,CHRDL2,S100B,PAK6,TRIB3,GJC2,MYH7,OOSP2,CRP,TLL2,BFSP1,PDE2A,NGEF,SPRED3,CACNA1S,ALDH1A2,RCAN1,TAL1,BATF3,NUPR1,ARHGAP22,KIAA0319,RIMBP3,SLC7A5,VAX2,KIRREL3,TRPV2,ISLR2,SPOCD1,KLF2,NPY,GBA,TSSK1B,SYN1,SQSTM1,SPRR2G,IL7R,INHBA,NFAM1,CHAC1,BTBD6,FOXD4L1,BEND6,PNPLA1,CHRNB2,ASTN2,DACT2,DRD1,TCTA,TNFRSF11A,CDK5R2,PCSK1,BAD,NCS1,CDHR1,PPARG,ALK,ATP8A2,USH1G,TPGS1,HTR2A,KRT37,TMEFF2,RET,IRF4,ACHE,TMEM120A,FOXD4
GO:BP	import into cell	GO:0098657	0.00588361	2.2303559490315346	SLC6A2,CXCL8,SYK,NEURL3,RAMP1,SLC18A3,RAMP3,DMBT1,SERPINE1,DYSF,CCL2,SLC6A11,PLCG2,DRD2,SLC1A4,IL15RA,GSG1L,SPHK1,CRP,CACNA1S,COLEC10,SLC7A5,SLC5A1,TMPRSS3,SLC6A9,RIN3,CALY,ATP1A3,MLC1,SLC6A12,TEX261,DRD1,HRAS,LRRTM1,SYT5,PPARG,USH1G,SLC36A1,ADORA1,SNCB,ACHE,HCN2,SLC22A3,SYP

GO:BP	cognition	GO:0050890	0.00615284	2.210924351623785	DBH,TH,EGR2,DRD5,GABRA5,HRH1,GALR2,BDNF,DRD2,SLC1A4,OPRL1,TACR1,KCNK4,S100B,GALR3,PAK6,RCAN1,PRKAR1B,CHRNB2,DRD1,HTR2A,ADORA1
GO:BP	regulation of multicellular organismal development	GO:2000026	0.00655361	2.1835192857919834	CXCL8,GAL,NKX6-2,SYK,NKX2-2,EGR2,OSR2,TG,PTHLH,GPNMB,GATA4,KL,CD28,SRPX2,RBP4,EGR3,BCAN,TMEM119,SIX2,SERPINE1,CARTPT,HMOX1,ITPKA,GPR68,BDNF,HAP1,ASCL2,IL1RL2,ETS1,DRD2,IL15RA,GPR137,TBX21,ASIC2,GJC2,SPHK1,SPRED3,VEGFB,TAL1,PHLDA2,NUPR1,KIAA0319,CYBB,SLC7A5,TRPV2,TNFAIP3,ISLR2,KLF2,ADM2,IL7R,NPPB,INHBA,NFAM1,TCTA,LRRTM1,BAD,PPARG,IRF4
GO:BP	calcium ion transport	GO:0006816	0.00688314	2.16221319	RAMP1,ADRA2A,BHLHA15,RAMP3,REM1,NPSR1,MCOLN3,OPRD1,TRPM8,SLC24A3,HAP1,CCL2,PLCG2,DRD2,OPRL1,GJC2,CACNA1S,CABP1,TRPV2,CCL5,PSEN2,MCHR1,CHRNB2,ATP2B3,DRD1,NCS1,HTR2A
GO:BP	muscle system process	GO:0003012	0.00705488	2.1515101279878213	UTS2,ADRA1B,MYH2,ADRA2A,GATA4,KCNA1,CNN1,HMOX1,KCNE4,GALR2,HTR7,DRD2,TACR1,SPHK1,MYH7,CACNA1S,VEGFB,KCNA5,SCN2B,GAMT,CHRNB2,DRD1,PPARG,ATP8A2,HTR2A,ADORA1
GO:BP	regulation of potassium ion transmembrane transport	GO:1901379	0.00725402	2.139421282103755	GAL,GRP,KCNS2,KCNA1,KCNS1,KCNK3,KCNE4,GALR2,MTNR1B,KCNC2,KCNIP1
GO:BP	ERK1 and ERK2 cascade	GO:0070371	0.00754482	2.1223512377648714	SYK,FFAR4,GPNMB,RAMP3,GATA4,PTGER4,NPSR1,CCL2,CCL18,DRD2,CCL20,SPRED3,HCRTR1,VEGFB,SSTR4,DUSP26,CCL5,DUSP5,INHBA,HRAS,TNFRSF11A,HTR2A
GO:BP	regulation of catecholamine secretion	GO:0050433	0.0084544	2.072917295221709	ADRA2A,SYT17,CARTPT,DRD2,CHRNA6,CHRNB2,SYT5,SYT9,HTR2A
GO:BP	regulation of anatomical structure size	GO:0090066	0.00860651	2.0651729833361236	UTS2,DBH,ADRA1B,ADRA2A,DRD5,HRH1,HTR7,GCH1,BDNF,SPTB,ADRA1D,TACR1,ASIC2,CRP,P2RY2,KIAA0319,KCNA5,MTNR1B,TRPV2,ISLR2,KLF2,RIN3,IL7R,NPPB,HRH2,DRD1,HTR2A,ADORA1,RET
GO:BP	learning or memory	GO:0007611	0.00867699	2.0616310257557813	DBH,TH,EGR2,DRD5,GABRA5,HRH1,GALR2,BDNF,DRD2,OPRL1,TACR1,KCNK4,S100B,GALR3,PAK6,RCAN1,PRKAR1B,CHRNB2,DRD1,HTR2A
GO:BP	peptide secretion	GO:0002790	0.00898626	2.0464208057986806	GAL,GRP,VGF,ADRA2A,FFAR4,RBP4,MAFA,PTPRN,CARTPT,GPR68,DRD2,MYRIP,KCNA5,MTNR1B,CCL5,RASL10B,DOC2B,BAD,ADORA1
GO:BP	memory	GO:0007613	0.00907826	2.0419975047932764	DBH,TH,HRH1,BDNF,DRD2,TACR1,KCNK4,S100B,PAK6,RCAN1,CHRNB2,DRD1,HTR2A
GO:BP	catecholamine metabolic process	GO:0006584	0.00984501	2.006784019061785	DBH,TH,KL,GCH1,DRD2,MAOA,CHRNB2,DRD1,SNCB
GO:BP	catechol-containing compound metabolic process	GO:0009712	0.00984501	2.006784019061785	DBH,TH,KL,GCH1,DRD2,MAOA,CHRNB2,DRD1,SNCB
GO:BP	positive regulation of monoatomic ion transport	GO:0043270	0.010193132809279247	1.991692317202561	GAL,ADRA2A,RAMP3,NPSR1,KCNA1,KCNK3,GALR2,HAP1,CCL2,PLCG2,ASIC2,GJC2,TRPV2,KCNC2,CCL5,MCHR1,DRD1,ADORA1
GO:BP	positive regulation of ERK1 and ERK2 cascade	GO:0070374	0.011280122959334006	1.9476861662862572	FFAR4,GPNMB,RAMP3,GATA4,NPSR1,CCL2,CCL18,DRD2,CCL20,HCRTR1,VEGFB,SSTR4,CCL5,INHBA,HRAS,TNFRSF11A,HTR2A
GO:BP	rhythmic behavior	GO:0007622	0.01136525	1.9444210153648975	TH,EGR2,EGR1,DRD2,OPRL1,MTNR1B,CHRNB2,ADORA1
GO:BP	nervous system process	GO:0050877	0.01286007	1.890756772206577	DBH,NKX6-2,OR2W3,TH,TMIE,ADRA2A,EGR2,DRD5,FFAR4,GABRA5,DRGX,RBP4,PHF24,GRM7,HRH1,KCNA1,GABRG3,CARTPT,GALR2,GCH1,BDNF,TRPM8,SFRP5,CCL2,DRD2,SLC1A4,OPRL1,TACR1,KCNK4,S100B,GALR3,PAK6,ASIC2,RTP1,MYH7,NTSR2,BEGAIN,CABP1,RCAN1,OR2L13,GABRE,MTNR1B,VAX2,TRPV2,CHRNA6,TMPRSS3,GBA,MGLL,MIP,PRKAR1B,GJA10,CHRNB2,DRD1,REEP2,ATP8A2,USH1G,GABRQ,HTR2A,ADORA1,TMEM120A
GO:BP	cellular homeostasis	GO:0019725	0.013443615348694959	1.871483922141944	SLC6A2,TH,ADRA2A,DRD5,GATA4,PTPRN,CYB561,NPSR1,MCOLN3,BOK,CARTPT,HMOX1,GPR68,HKDC1,TRPM8,SLC24A3,HAP1,CCL2,PLCG2,DRD2,SLC4A3,P2RY2,NQO1,HCRTR1,TMPRSS3,DGAT2,CCL5,TXNRD2,ATP1A3,ATP6V0B,ATP2B3,ATP6V0C,DRD1,BAD,ATP6V0D1,ATP6V0A1,HTR2A,ADORA1,PQLC2

GO:BP	multicellular organismal response to stress	GO:0033555	0.0141497	1.849252728059017	SLC6A2,DBH,GRP,ADRA2A,GABRA5,GCH1,TACR1,KIAA0319,PRKAR1B,DRD1,RET
GO:BP	catecholamine secretion	GO:0050432	0.015255269329917828	1.8165801205378256	ADRA2A,SYT17,CARTPT,DRD2,CHRNA6,CHRNB2,SYT5,SYT9,HTR2A
GO:BP	positive regulation of potassium ion transport	GO:0043268	0.016096671682797197	1.7932639139821362	GAL,ADRA2A,KCNA1,KCNK3,GALR2,KCNC2,DRD1,ADORA1
GO:BP	response to mechanical stimulus	GO:0009612	0.016437962873827122	1.7841520047688366	GATA4,PTGER4,PHF24,KCNA1,DRD2,TACR1,KCNK4,ASIC2,PDE2A,KIAA0319,KCNA5,TNFRSF11A,BAD,PPARG,ATP8A2,HTR2A,TMEM120A
GO:BP	response to interleukin-1	GO:0070555	0.018162609297780144	1.7408217592167037	CXCL8,EGR1,CITED1,CCL2,IL1RL2,CCL18,CCL20,KLF2,CCL5,TNFRSF11A,PCSK1,IRAK1,IRAK2
GO:BP	cellular response to organic cyclic compound	GO:0071407	0.019797054917962062	1.7033994121632117	DRD5,HTR6,UGT3A2,RAMP3,NR0B1,EGR1,CHRM4,HRH1,HTR7,CCL2,DRD2,P2RY2,HTR5A,PDE2A,CACNA1S,SSTR4,KLF2,AANAT,HTR1E,CCL5,CALY,INHBA,ATP1A3,MLC1,HRH2,DRD1,PPARG,ALK,HTR2A,HCN2
GO:BP	phenol-containing compound metabolic process	GO:0018958	0.020485047826421823	1.6885630177627382	DBH,TH,TG,KL,CITED1,GCH1,FOXE1,DRD2,MAOA,CHRNB2,DRD1,SNCB
GO:BP	cell surface receptor signaling pathway	GO:0007166	0.020970664286758412	1.6783878121906317	DBH,CXCL8,FGF3,SYK,TIMP4,IL31RA,NKX2-2,SUSD5,FAM83G,GATA4,KL,CD28,IGFBP3,SOSTDC1,TPBGL,EGR1,CHRM4,SERPINE1,GRM7,BOK,CITED1,HMOX1,ITGA11,GALR2,ADAP1,TNFRSF12A,ADGRF4,BDNF,PALM3,CRLF2,HAP1,CCKBR,SFRP5,CCL2,IL1RL2,CCL18,PLCG2,DRD2,IL15RA,CHRDL2,SCG2,SPHK1,P2RY2,NTSR2,NGEF,CCL20,SPRED3,SKAP1,RSPO4,VEGFB,COLEC10,KIAA0319,KCNA5,CSRNP1,VAX2,TNFAIP3,CHRNA6,CTSD,ADAMTSL2,CCL5,RFX8,PSEN2,PRKAR1B,UBASH3A,IL7R,MCHR1,NPPB,OASL,INHBA,NFAM1,CHAC1,KRT18,BEND6,LY6E,CHRNB2,PALM2,ATP6V0C,DACT2,MS4A2,HRAS,ADGRA1,TNFRSF11A,BAD,TMEM198,IRAK1,FIBP,PPARG,ALK,LIMS2,ADORA1,IRAK2,TEC,RET,AMER3,TICAM1
GO:BP	intracellular chemical homeostasis	GO:0055082	0.021836308444016145	1.6608207797906915	TH,ADRA2A,DRD5,GATA4,PTPRN,CYB561,NPSR1,MCOLN3,BOK,CARTPT,HMOX1,GPR68,HKDC1,TRPM8,SLC24A3,HAP1,PLCG2,DRD2,SLC4A3,P2RY2,HCRTR1,TMPRSS3,DGAT2,CCL5,ATP1A3,ATP6V0B,ATP2B3,ATP6V0C,DRD1,BAD,ATP6V0D1,ATP6V0A1,HTR2A,ADORA1,PQLC2
GO:BP	negative regulation of blood pressure	GO:0045776	0.022393952870347983	1.6498692401298438	DRD5,KL,GCH1,DRD2,OPRL1,ADM2,NPPB,ADORA1
GO:BP	regulation of molecular function	GO:0065009	0.022399811436975767	1.6497556375687656	GAL,GRP,SYK,TIMP4,ADRA2A,TRIM58,DRD5,NPFFR2,RAMP3,MMP8,GALR1,REM1,NR0B1,EGR1,KCNS2,SERPINE1,SERPINA3,RASAL3,KCNA1,KCNS1,BOK,CARTPT,HMOX1,KCNK3,KCNE4,GALR2,OPRD1,ADAP1,GCH1,IL32,BDNF,FOXL2,HAP1,SFRP5,CCL2,CCL18,PLCG2,DRD2,OPRL1,GSG1L,GALR3,ASIC2,TRIB3,SPHK1,NQO1,BEGAIN,NGEF,CCL20,CABP1,RCAN1,PHLDA2,NUPR1,ARHGAP22,MTNR1B,TNFAIP3,KCNC2,GBA,CTSD,CCL5,SCN2B,SLC6A9,PSEN2,PRKAR1B,HM13,OASL,MAPK8IP1,NFAM1,DBNDD1,LAMP3,DRD1,RASGEF1C,HRAS,TNFRSF11A,CDK5R2,BAD,IRAK1,PPARG,ALK,CAMK2N2,HTR2A,RASGEF1A,ADORA1,RGS6,IRAK2,RET,TICAM1,IRF4,HCN2
GO:BP	peptide transport	GO:0015833	0.023017513140728637	1.6379416002371134	GAL,GRP,VGF,ADRA2A,FFAR4,RBP4,MAFA,PTPRN,CARTPT,GPR68,DRD2,MYRIP,KCNA5,MTNR1B,CCL5,RASL10B,DOC2B,BAD,ADORA1
GO:BP	neurotransmitter reuptake	GO:0098810	0.023277920635613097	1.6330558168405298	SLC6A2,SLC18A3,SLC6A11,DRD2,SLC6A12,DRD1,SLC22A3
GO:BP	regulation of systemic arterial blood pressure	GO:0003073	0.023713192998163917	1.6250099640260733	ADRA1B,DRD5,KL,DRD2,TACR1,ASIC2,ADM2,RASL10B,NPPB,CYP4A11,ADORA1
GO:BP	sensory perception of pain	GO:0019233	0.02513512	1.5997189820154505	PHF24,KCNA1,CCL2,OPRL1,KCNK4,MGLL,CHRNB2,HTR2A,ADORA1,TMEM120A

GO:BP	peptide hormone secretion	GO:0030072	0.02574552	1.5892983269113112	GAL,GRP,VGF,ADRA2A,FFAR4,RBP4,MAFA,PTPRN,CARTPT,GPR68,DRD2,MYRIP,KCNA5,MTNR1B,CCL5,RASL10B,DOC2B,BAD
GO:BP	heart process	GO:0003015	0.02574552	1.5892983269113112	ADRA1B,TH,RAMP3,GATA4,KCNE4,GCH1,DRD2,SLC4A3,MYH7,HSPB7,VEGFB,KCNA5,SCN2B,ADM2,ATP1A3,ATP2B3,ADORA1,HCN2
GO:BP	biogenic amine metabolic process	GO:0006576	0.02618733	1.5819087508290974	DBH,TH,KL,GCH1,DRD2,MAOA,AANAT,CHRNB2,DRD1,SNCB,SLC22A3
GO:BP	regulation of heart contraction	GO:0008016	0.026458667954129964	1.5774320238950363	ADRA1B,TH,GATA4,KCNE4,GCH1,DRD2,SLC4A3,MYH7,HSPB7,KCNA5,SCN2B,ADM2,ATP1A3,ATP2B3,ADORA1,HCN2
GO:BP	regulation of ERK1 and ERK2 cascade	GO:0070372	0.028671728693796267	1.542546121509621	SYK,FFAR4,GPNMB,RAMP3,GATA4,NPSR1,CCL2,CCL18,DRD2,CCL20,SPRED3,HCRTR1,VEGFB,SSTR4,DUSP26,CCL5,INHBA,HRAS,TNFRSF11A,HTR2A
GO:BP	immune system process	GO:0002376	0.029053517749072215	1.536801276474371	DBH,CXCL8,GAL,GRP,RELB,SYK,TNFRSF9,NEURL3,CD300E,IL31RA,TRIM58,GPNMB,MMP8,DMBT1,CD28,PTGER4,RBP4,EGR3,EGR1,CD300LB,UBD,SERPINE1,BPIFC,PAX1,RASAL3,CD300C,CITED1,CARTPT,HMOX1,GPR68,DYSF,OPRD1,GCH1,IL32,CRLF2,TMEM229B,FOXE1,FCMR,CCL2,IL1RL2,ETS1,CCL18,PLCG2,DRD2,IL15RA,GPR137,TBX21,GZMB,TACR1,SCG2,SPHK1,CRP,TRIM29,NQO1,RAET1G,CCL20,SKAP1,VEGFB,COLEC10,TAL1,BATF3,CYBB,SLC7A5,TNFAIP3,KLF2,GBA,CTSD,CCL5,SQSTM1,UBASH3A,RIN3,IL7R,OASL,MAPK8IP1,INHBA,NFAM1,CHRNB2,HRH2,LAMP3,TCTA,MS4A2,HRAS,TNFRSF11A,BAD,IRAK1,PPARG,ADORA1,IRAK2,TEC,RET,TICAM1,IRF4,HIST1H2BK
GO:BP	positive regulation of response to stimulus	GO:0048584	0.02937292	1.5320528101435218	CXCL8,GRP,ADRA1B,SYK,ADRA2A,TRIM58,FFAR4,HTR6,GPNMB,RAMP3,MMP8,GATA4,KL,CD28,IGFBP3,PTGER4,UBD,SERPINE1,NPSR1,BOK,CITED1,CARTPT,HMOX1,TNFRSF12A,BDNF,CRLF2,HAP1,CCL2,ETS1,CCL18,PLCG2,DRD2,GPR137,TBX21,S100B,SCG2,SPHK1,MAOA,PDE2A,RAET1G,CCL20,SKAP1,HCRTR1,RSPO4,VEGFB,COLEC10,PLEKHF1,NUPR1,KIAA0319,SSTR4,TNFAIP3,KLF2,NPY,CTSD,CCL5,PRKAR1B,UBASH3A,IL7R,OASL,MAPK8IP1,INHBA,NFAM1,ATP6V0C,NENF,HRAS,TNFRSF11A,BAD,TMEM198,IRAK1,PPARG,HTR2A,RASGEF1A,LIMS2,ADORA1,IRAK2,TEC,RET,TICAM1,IRF4
GO:BP	monoatomic ion homeostasis	GO:0050801	0.030649732869778762	1.51357331	DRD5,KL,CYB561,NPSR1,MCOLN3,KCNA1,BOK,HMOX1,TRPM8,SLC24A3,HAP1,CCKBR,PLCG2,DRD2,SLC4A3,P2RY2,HCRTR1,KCNA5,TMPRSS3,CCL5,NPPB,ATP1A3,ATP6V0B,ATP2B3,ATP6V0C,CYP4A11,DRD1,ATP6V0D1,ATP6V0A1,HTR2A,ADORA1
GO:BP	regulation of cell population proliferation	GO:0042127	0.03370251	1.47233771	DBH,CXCL8,GAL,EGR4,FGF3,SYK,TNFRSF9,IL31RA,ADRA2A,OSR2,PTHLH,GPNMB,P3H2,CD28,IGFBP3,RBP4,EGR3,PTGER2,EGR1,PTPRN,BCAN,TMEM119,SIX2,RASAL3,BOK,CITED1,CNN1,HMOX1,ITPKA,BDNF,CRLF2,OSGIN1,HAP1,ASCL2,CCKBR,SFRP5,CCL2,ADRA1D,ETS1,DRD2,IL15RA,TACR1,S100B,SCG2,GJC2,SPHK1,CRP,NGEF,ALDH1A2,VEGFB,TAL1,PHLDA2,NUPR1,KIAA0319,KCNA5,SSTR4,SLC7A5,TRPV2,TNFAIP3,ISLR2,CCL5,IL7R,MAPK8IP1,INHBA,CHRNB2,HRAS,TNFRSF11A,BAD,NCS1,IRAK1,PPARG,ALK,ATP8A2,HTR2A,LIMS2,ADORA1,RET,TICAM1
GO:BP	regulation of dopamine secretion	GO:0014059	0.03430296	1.4646683791014574	SYT17,DRD2,CHRNA6,CHRNB2,SYT5,SYT9,HTR2A
GO:BP	temperature homeostasis	GO:0001659	0.03502303	1.4556463128414685	DBH,SYK,FFAR4,EGR1,TRPM8,DRD2,TRPV2,PEMT,SQSTM1,DRD1,TNFRSF11A,HTR2A,ADORA1,IRF4,ACHE
GO:BP	response to oxygen levels	GO:0070482	0.037276438619676555	1.4285655867071425	TH,NGB,EGR1,HMOX1,KCNK3,OPRD1,ASCL2,DRD2,VEGFB,KCNA5,CYBB,SLC7A5,TXNRD2,PSEN2,CHRNB2,CYGB,BAD,IRAK1,ATP6V0D1,PPARG,ADORA1
GO:BP	cellular response to organonitrogen compound	GO:0071417	0.03763663	1.4243892359704893	TH,DRD5,HTR6,EGR1,CHRM4,HRH1,HTR7,DRD2,TRIB3,P2RY2,HTR5A,PDE2A,CACNA1S,LY6H,CYBB,SLC7A5,KLF2,AANAT,HTR1E,CTSD,CALY,INHBA,ATP1A3,CHRNB2,HRH2,DRD1,HRAS,PPARG,ALK,HTR2A,ACHE,HCN2

GO:BP	regulation of response to stimulus	GO:0048583	0.03837137	1.4159926458713255	DBH,CXCL8,GRP,ADRA1B,SYK,RAMP1,ADRA2A,TRIM58,NPFFR2,FFAR4,HTR6,GPNMB,RAMP3,MMP8,GATA4,KL,CD28,IGFBP3,PTGER4,SOSTDC1,PHF24,TPBGL,NR0B1,EGR1,UBD,SERPINE1,NPSR1,RASAL3,BOK,CITED1,CARTPT,HMOX1,TNFRSF12A,GCH1,BDNF,PALM3,CRLF2,HAP1,SFRP5,CCL2,IL1RL2,ETS1,CCL18,PLCG2,DRD2,OPRL1,GPR137,TBX21,GSG1L,CHRDL2,S100B,SCG2,PAK6,SPHK1,MYH7,MAOA,BEGAIN,PDE2A,RAET1G,NGEF,CCL20,SPRED3,SKAP1,HCRTR1,RSPO4,RCAN1,VEGFB,COLEC10,PLEKHF1,PHLDA2,NUPR1,ARHGAP22,KIAA0319,MTNR1B,SSTR4,TNFAIP3,DUSP26,KLF2,NPY,GBA,MGLL,CTSD,ADAMTSL2,CCL5,TSSK1B,SLC6A9,RFX8,PRKAR1B,SQSTM1,UBASH3A,RIN3,IL7R,DUSP5,OASL,MAPK8IP1,INHBA,NFAM1,CHAC1,MLC1,BEND6,ATP6V0C,NENF,DACT2,DRD1,HRAS,TNFRSF11A,BAD,TMEM198,IRAK1,PPARG,ALK,HTR2A,RASGEF1A,LIMS2,ADORA1,RGS6,IRAK2,TEC,RET,AMER3,TICAM1,IRF4,SYP
GO:BP	amide transport	GO:0042886	0.040554790275728565	1.391957840159497	FOLR3,GAL,GRP,VGF,ADRA2A,FFAR4,RBP4,MAFA,PTPRN,GRM7,CARTPT,GPR68,DRD2,SLC1A4,MYRIP,KCNA5,MTNR1B,CCL5,RASL10B,DOC2B,BAD,ADORA1
GO:BP	inflammatory response	GO:0006954	0.041818780564860894	1.3786286353555863	CXCL8,RELB,SYK,IL31RA,FFAR4,MMP8,CD28,PTGER4,PTGER2,SERPINE1,SERPINA3,HRH1,HMOX1,GPR68,CCL2,IL1RL2,ETS1,CCL18,PLCG2,TACR1,SCG2,SPHK1,CRP,PDE2A,CCL20,NUPR1,CYBB,TNFAIP3,GBA,MGLL,CCL5,NFAM1,TNFRSF11A,PLA2G4C,PPARG,ADORA1,IRAK2,TICAM1
GO:BP	adenylate cyclase-activating adrenergic receptor signaling pathway	GO:0071880	0.0428208	1.3683452679800663	ADRA1B,ADRA2A,DRD5,ADRA1D,DRD2,DRD1
GO:BP	detection of abiotic stimulus	GO:0009582	0.04331196	1.3633921689728108	NPFFR2,DRGX,PHF24,KCNA1,TACR1,KCNK4,ASIC2,GJA10,PITPNM1,ATP8A2,HTR2A,ADORA1,TMEM120A
GO:BP	response to external stimulus	GO:0009605	0.0457449	1.339657296757112	CXCL8,GRP,RELB,SYK,VGF,NEURL3,TH,IL31RA,TRIM58,BHLHA15,FFAR4,GPNMB,MMP8,GATA4,DRGX,KL,DMBT1,CD28,PTGER4,EGR3,PHF24,PTGER2,UBD,SERPINE1,BPIFC,NPSR1,KCNA1,CITED1,CARTPT,GCH1,PALM3,TMEM229B,CCL2,IL1RL2,ETS1,CCL18,PLCG2,DRD2,IL15RA,TBX21,GZMB,TACR1,KCNK4,SCG2,ASIC2,SPHK1,CRP,TRIM29,NQO1,PDE2A,RAET1G,CCL20,ALDH1A2,VEGFB,COLEC10,BATF3,NUPR1,KIAA0319,KCNA5,CYBB,SLC7A5,TNFAIP3,NPY,GBA,MGLL,CCL5,TSSK1B,RIN3,IL7R,GJA10,OASL,ABCG5,NENF,PITPNM1,DRD1,HRAS,TNFRSF11A,PCSK1,BAD,IRAK1,PPARG,ALK,ATP8A2,HTR2A,ADORA1,IRAK2,TICAM1,IRF4,TMEM120A,SLC22A3,HIST1H2BK
GO:BP	regulation of developmental process	GO:0050793	0.04824346	1.3165615210835442	CXCL8,GAL,FGF3,NKX6-2,SYK,NKX2-2,EGR2,TRIM58,OSR2,BHLHA15,FFAR4,TG,PTHLH,GPNMB,ATP10A,GATA4,KL,CD28,IGFBP3,SRPX2,SOSTDC1,RBP4,EGR3,BCAN,TMEM119,SIX2,SERPINE1,SYT17,C1QL2,CITED1,CARTPT,HMOX1,ITPKA,GPR68,BDNF,PALM3,HAP1,ASCL2,SFRP5,CCL2,IL1RL2,ETS1,DRD2,IL15RA,GPR137,TBX21,S100B,ASIC2,TRIB3,GJC2,SPHK1,CRP,TLL2,NGEF,SPRED3,VEGFB,TAL1,PHLDA2,NUPR1,KIAA0319,CYBB,SLC7A5,TRPV2,TNFAIP3,ISLR2,KLF2,ADM2,IL7R,NPPB,INHBA,NFAM1,GAMT,BEND6,CHRNB2,PALM2,TCTA,LRRTM1,BAD,PPARG,ALK,ATP8A2,DMRT2,HTR2A,RET,IRF4
GO:BP	locomotory behavior	GO:0007626	0.048454519790364636	1.3146657061578548	DBH,TH,EGR1,MCOLN3,OPRD1,HOXD9,DRD2,OPRL1,PAK6,RCAN1,TAL1,CHRNB2,DRD1,LRRTM1,ALK,QRFP
GO:BP	cellular response to nitrogen compound	GO:1901699	0.049427942142217254	1.3060274703582317	TH,DRD5,HTR6,EGR1,CHRM4,HRH1,HTR7,DRD2,TRIB3,P2RY2,HTR5A,PDE2A,CACNA1S,LY6H,CYBB,SLC7A5,KLF2,KCNC2,AANAT,HTR1E,CTSD,CALY,INHBA,ATP1A3,CHRNB2,HRH2,DRD1,HRAS,PPARG,ALK,HTR2A,ACHE,HCN2
GO:BP	heart contraction	GO:0060047	0.04952189	1.3052027791026763	ADRA1B,TH,GATA4,KCNE4,GCH1,DRD2,SLC4A3,MYH7,HSPB7,VEGFB,KCNA5,SCN2B,ADM2,ATP1A3,ATP2B3,ADORA1,HCN2

GO:CC	synapse	GO:0045202	3.7952424369399224e-21	20.42076047651099	SLC6A2,UTS2,DBH,RELB,VGF,TH,IL31RA,ADRA2A,DRD5,SLC18A3,HTR6,GABRA5,SRPX2,LGI3,EGR3,PTPRN,BCAN,CHRM4,C1QL2,SLC6A17,GRM7,HRH1,KCNA1,GABRG3,CARTPT,ITPKA,NPBWR1,SV2C,KCNK3,DYSF,OPRD1,HTR7,BDNF,SPTB,HAP1,SLC6A11,DRD2,SLC1A4,GSG1L,GALR3,PAK6,ASIC2,SPHK1,HTR5A,NQO1,BEGAIN,MYRIP,PDE2A,HCRTR1,CABP1,LY6H,GABRE,ARHGAP22,MTNR1B,KIRREL3,CHRNA6,KCNC2,NPY,SYNPO,HTR1E,SCN2B,SLC6A9,PSEN2,PRKAR1B,SYN1,CALY,MAPK8IP1,ATP1A3,SLC6A12,BAALC,CHRNB2,ATP2B3,ATP6V0C,HRH2,DRD1,DOC2B,HRAS,LRFN2,ADGRA1,LRRTM1,SYT5,PCSK1,SYT9,NCS1,MAL2,ATP6V0D1,CYP46A1,CAMK2N2,ATP6V0A1,GABRQ,TPGS1,HTR2A,ADORA1,SNCB,ACHE,ABLIM3,SLC22A3,SYP
GO:CC	cell periphery	GO:0071944	4.626987249071768e-18	17.33470169731562	SLC6A2,PRRG2,GPR50,MMP1,PRLHR,ADRA1B,MS4A3,MC5R,SYK,TNFRSF9,TIMP4,CD300E,OR2W3,TH,IL31RA,RAMP1,KRT75,ADRA2A,STYK1,DRD5,NPFFR2,SYTL5,FFAR4,SLC18A3,HTR6,GPNMB,RAMP3,P3H2,ATP10A,MMP8,GABRA5,GALR1,KL,DMBT1,MMP19,CD28,COL8A1,SRPX2,SLC5A10,PTGER4,REM1,TPBGL,PTGER2,PTPRN,BCAN,CHRM4,KCNS2,CD300LB,TMEM119,SERPINE1,KCNK13,SERPINA3,NPSR1,PTPRH,SLC10A4,MCOLN3,GPR83,SLC6A17,RASAL3,AQP5,CD300C,GRM7,HRH1,KCNA1,GABRG3,KCNS1,SLC7A4,OTOP2,NPBWR1,SV2C,GPR68,MALL,KCNK3,DYSF,ITGA11,KCNE4,GALR2,OPRD1,ADAP1,HAS1,TNFRSF12A,HTR7,PALM3,SPTB,CRLF2,TRPM8,SLC24A3,CCKBR,FCMR,EGFL6,ADRA1D,IL1RL2,SLC6A11,PLCG2,SLC2A6,DRD2,SLC1A4,OPRL1,IL15RA,SLC4A3,GSG1L,GZMB,TACR1,KCNK4,SHROOM1,ACTG2,GALR3,ASIC2,TRIB3,GJC2,SPHK1,RTP1,P2RY2,NTSR2,HTR5A,BFSP1,MYRIP,PDE2A,RAET1G,SPRED3,SKAP1,CACNA1S,HCRTR1,CABP1,OR2L13,LY6H,COLEC10,GABRE,KIAA0319,KCNA5,MTNR1B,SSTR4,CYBB,SLC7A5,KIRREL3,TRPV2,ISLR2,CHRNA6,SLC5A1,KCNC2,FIBCD1,SYNPO,MGLL,HTR1E,MMP7,CTSD,MIP,ADAMTSL2,SCN2B,COL5A3,SLC6A9,LYPD6B,PSEN2,PRKAR1B,KCNK15,GPR45,SPRR2G,OTOP3,RASL10B,IL7R,GJA10,MCHR1,HM13,CALY,MAPK8IP1,ABCG5,SLC9A3R2,NFAM1,ATP1A3,MLC1,KRT18,SLC6A12,LY6E,CHRNB2,ATP2B3,PALM2,ATP6V0C,CYP4A11,HRH2,ASTN2,LAMP3,PITPNM1,DRD1,DOC2B,RASGEF1C,MS4A2,HRAS,LRFN2,REEP2,ADGRA1,SPRN,TNFRSF11A,CDK5R2,LRRTM1,SYT9,PLA2G4C,NCS1,COL8A2,MAL2,KCNIP1,TMEM198,IRAK1,CDHR1,KIF17,ABCA3,ATP6V0D1,ALK,ATP8A2,VSIG2,USH1G,SLC36A1,ATP6V0A1,GABRQ,HTR2A,SURF2,RASGEF1A,CDH22,LIMS2,ADORA1,EMP1,RGS6,IRAK2,CPNE7,TEC,RET,AMER3,PARD6A,ACHE,HCN2,TMEM120A,SLC22A3,NOXO1,SYP
GO:CC	cell junction	GO:0030054	4.616264275753825e-16	15.335709336287291	SLC6A2,UTS2,DBH,RELB,VGF,MYH2,TH,IL31RA,ADRA2A,DRD5,SLC18A3,HTR6,GABRA5,SRPX2,LGI3,EGR3,PTPRN,BCAN,CHRM4,C1QL2,SLC6A17,GRM7,HRH1,KCNA1,GABRG3,CARTPT,CNN1,ITPKA,NPBWR1,SV2C,KCNK3,DYSF,ITGA11,OPRD1,HTR7,BDNF,SPTB,HAP1,SLC6A11,DRD2,SLC1A4,GSG1L,SHROOM1,GALR3,PAK6,ASIC2,GJC2,SPHK1,TRIM29,HTR5A,NQO1,BEGAIN,MYRIP,PDE2A,SKAP1,HCRTR1,CABP1,LY6H,GABRE,ARHGAP22,KCNA5,MTNR1B,KIRREL3,HEPACAM,CHRNA6,KCNC2,NPY,SYNPO,HTR1E,MIP,SCN2B,SLC6A9,PSEN2,PRKAR1B,SYN1,GJA10,CALY,MAPK8IP1,SLC9A3R2,ATP1A3,KRT18,SLC6A12,BAALC,CHRNB2,ATP2B3,ATP6V0C,HRH2,DRD1,DOC2B,HRAS,LRFN2,ADGRA1,LRRTM1,SYT5,PCSK1,SYT9,NCS1,MAL2,ATP6V0D1,CYP46A1,CAMK2N2,ATP6V0A1,GABRQ,TPGS1,HTR2A,CDH22,LIMS2,ADORA1,SNCB,PARD6A,ACHE,ABLIM3,SLC22A3,SYP,RPS10

GO:CC	plasma membrane	GO:0005886	6.536530241196081e-15	14.184652725220266	SLC6A2,PRRG2,GPR50,PRLHR,ADRA1B,MS4A3,MC5R,SYK,TNFRSF9,CD300E,OR2W3,TH,IL31RA,RAMP1,KRT75,ADRA2A,STYK1,DRD5,NPFFR2,SYTL5,FFAR4,SLC18A3,HTR6,GPNMB,RAMP3,ATP10A,GABRA5,GALR1,KL,CD28,SRPX2,SLC5A10,PTGER4,REM1,TPBGL,PTGER2,PTPRN,CHRM4,KCNS2,CD300LB,TMEM119,SERPINE1,KCNK13,NPSR1,PTPRH,SLC10A4,MCOLN3,GPR83,SLC6A17,AQP5,CD300C,GRM7,HRH1,KCNA1,GABRG3,KCNS1,SLC7A4,OTOP2,NPBWR1,SV2C,GPR68,MALL,KCNK3,DYSF,ITGA11,KCNE4,GALR2,OPRD1,ADAP1,HAS1,TNFRSF12A,HTR7,PALM3,SPTB,CRLF2,TRPM8,SLC24A3,CCKBR,FCMR,ADRA1D,IL1RL2,SLC6A11,PLCG2,SLC2A6,DRD2,SLC1A4,OPRL1,IL15RA,SLC4A3,GSG1L,GZMB,TACR1,KCNK4,SHROOM1,GALR3,ASIC2,TRIB3,GJC2,SPHK1,RTP1,P2RY2,NTSR2,HTR5A,BFSP1,PDE2A,RAET1G,SPRED3,SKAP1,CACNA1S,HCRTR1,CABP1,OR2L13,LY6H,COLEC10,GABRE,KIAA0319,KCNA5,MTNR1B,SSTR4,CYBB,SLC7A5,KIRREL3,TRPV2,ISLR2,CHRNA6,SLC5A1,KCNC2,SYNPO,MGLL,HTR1E,MIP,SCN2B,SLC6A9,LYPD6B,PSEN2,PRKAR1B,KCNK15,GPR45,SPRR2G,OTOP3,RASL10B,IL7R,GJA10,MCHR1,HM13,CALY,MAPK8IP1,ABCG5,SLC9A3R2,NFAM1,ATP1A3,MLC1,SLC6A12,LY6E,CHRNB2,ATP2B3,PALM2,ATP6V0C,CYP4A11,HRH2,LAMP3,PITPNM1,DRD1,DOC2B,RASGEF1C,MS4A2,HRAS,LRFN2,REEP2,ADGRA1,SPRN,TNFRSF11A,CDK5R2,LRRTM1,SYT9,PLA2G4C,NCS1,MAL2,KCNIP1,TMEM198,IRAK1,CDHR1,KIF17,ABCA3,ATP6V0D1,ALK,ATP8A2,VSIG2,USH1G,SLC36A1,ATP6V0A1,GABRQ,HTR2A,SURF2,RASGEF1A,CDH22,LIMS2,ADORA1,EMP1,RGS6,IRAK2,CPNE7,TEC,RET,AMER3,PARD6A,ACHE,HCN2,TMEM120A,SLC22A3,NOXO1,SYP
GO:CC	membrane	GO:0016020	1.7726689140052434e-13	12.751372371137734	SLC6A2,PRRG2,DBH,FOLR3,GPR50,PRLHR,ADRA1B,MS4A3,MC5R,TMEM156,SYK,TNFRSF9,CD300E,OR2W3,TH,IL31RA,RAMP1,KRT75,TMIE,ADRA2A,STYK1,DRD5,NPFFR2,SUSD5,SYTL5,FFAR4,SLC18A3,TMEM179,SYNDIG1L,HTR6,UGT3A2,GPNMB,RAMP3,KCNK12,ATP10A,APOL6,CLEC2L,GABRA5,GALR1,KL,DMBT1,CD28,SRPX2,SLC5A10,NIPAL2,PTGER4,REM1,TPBGL,PTGER2,NR0B1,PTPRN,MYLK4,BCAN,CHRM4,GOLT1A,KCNS2,CD300LB,TMEM119,SERPINE1,SYT17,KCNK13,CYB561,NPSR1,FAM163B,PTPRH,SLC10A4,MCOLN3,GPR83,SLC6A17,RASAL3,AQP5,CD300C,TMEM132E,GRM7,HRH1,KCNA1,GABRG3,KCNS1,SLC7A4,BOK,HMOX1,OTOP2,NPBWR1,SV2C,GPR68,MALL,KCNK3,HKDC1,DYSF,TMEM151A,ITGA11,KCNE4,GALR2,OPRD1,ADAP1,HAS1,TNFRSF12A,HTR7,GCH1,ADGRF4,IL32,PALM3,SPTB,CRLF2,TMEM229B,TRPM8,RTL1,SLC24A3,CCKBR,FCMR,EGFL6,ADRA1D,IL1RL2,SLC6A11,PLCG2,SLC2A6,DRD2,SLC1A4,OPRL1,IL15RA,FADS6,PCYT2,GPR137,SLC4A3,GSG1L,GZMB,TACR1,KCNK4,SHROOM1,GALR3,HS3ST2,ASIC2,TRIB3,GJC2,SPHK1,RTP1,EPHX1,P2RY2,SHISA4,NTSR2,MAOA,PLD5,HTR5A,TMEM217,HS6ST3,BEGAIN,BFSP1,PDE2A,RAET1G,SRXN1,NGEF,SPRED3,SKAP1,CACNA1S,HCRTR1,CABP1,OR2L13,LY6H,VEGFB,COLEC10,PLEKHF1,PHLDA2,GABRE,KIAA0319,KCNA5,MTNR1B,SSTR4,CYBB,DHRS13,SLC7A5,KIRREL3,HEPACAM,TRPV2,ISLR2,CHRNA6,SLC5A1,TMPRSS3,KCNC2,FIBCD1,SYNPO,GBA,PEMT,MGLL,HTR1E,DGAT2,CTSD,MIP,SCN2B,SLC6A9,LYPD6B,PSEN2,PRKAR1B,RHBDD2,KCNK15,SYN1,GPR45,TMEM54,SPRR2G,MANEAL,OTOP3,RASL10B,IL7R,GJA10,MCHR1,HM13,OASL,CALY,MAPK8IP1,ABCG5,SLC9A3R2,NFAM1,ATP1A3,ATP6V0B,MLC1,ELOVL7,SLC6A12,BAALC,LY6E,PNPLA1,CHRNB2,ATP2B3,PALM2,SLC35F4,ATP6V0C,CYP4A11,HRH2,ASTN2,TEX261,PTDSS1,NENF,LAMP3,PITPNM1,DRD1,DOC2B,RASGEF1C,TCTA,MS4A2,HRAS,LRFN2,REEP2,SLC37A1,ADGRA1,PEBP4,SPRN,SYS1-DBNDD2,TNFRSF11A,TMEM255B,TEX38,CDK5R2,LRRTM1,SYT5,PCSK1,SYT9,PLA2G4C,BAD,NCS1,MAL2,KCNIP1,TMEM198,IRAK1,CDHR1,KIF17,ABCA3,ATP6V0D1,FIBP,CYP46A1,ALK,ATP8A2,DMRT2,GALNT9,VSIG2,USH1G,SLC36A1,ATP6V0A1,GABRQ,ZDHHC14,HTR2A,SURF2,RASGEF1A,CDH22,LIMS2,ADORA1,EMP1,ABHD14A,SLC45A1,RGS6,IRAK2,TMEFF2,CPNE7,TEC,RET,STS,SEC11C,AMER3,TICAM1,DHCR7,IRF4,PARD6A,ACHE,HCN2,TMEM120A,SLC22A3,NOXO1,SYP,PQLC2,RPS10
GO:CC	somatodendritic compartment	GO:0036477	2.969811450380713e-11	10.527271122621706	SLC6A2,GAL,VGF,TH,NGB,ADRA2A,HTR6,GABRA5,PTPRN,CHRM4,GRM7,HRH1,KCNA1,GABRG3,ITPKA,OPRD1,HTR7,HAP1,DRD2,SLC1A4,TBX21,TACR1,S100B,ASIC2,GJC2,HTR5A,NQO1,GABRE,CYBB,KIRREL3,TMPRSS3,KCNC2,SYNPO,HTR1E,TXNRD2,PSEN2,SYN1,RIN3,MAPK8IP1,ATP1A3,HRH2,ASTN2,DRD1,SYT5,PCSK1,CYGB,NCS1,KCNIP1,KIF17,CYP46A1,TPGS1,HTR2A,ADORA1,RET,SNCB,HCN2,SLC22A3

GO:CC	presynapse	GO:0098793	3.2871552168367724e-11	10.483179788396573	SLC6A2,TH,IL31RA,ADRA2A,SLC18A3,GABRA5,LGI3,PTPRN,SLC6A17,GRM7,KCNA1,SV2C,DYSF,OPRD1,BDNF,HAP1,SLC6A11,DRD2,SPHK1,PDE2A,KIRREL3,CHRNA6,KCNC2,SLC6A9,PSEN2,SYN1,SLC6A12,CHRNB2,ATP2B3,ATP6V0C,DRD1,DOC2B,LRFN2,SYT5,PCSK1,SYT9,MAL2,ATP6V0D1,CYP46A1,ATP6V0A1,HTR2A,ADORA1,SNCB,SLC22A3,SYP
GO:CC	neuron projection	GO:0043005	6.210312585509264e-11	10.206886539800028	SLC6A2,GRP,PRLHR,TH,IL31RA,ADRA2A,SLC18A3,HTR6,GABRA5,LGI3,PTPRN,CHRM4,GRM7,HRH1,KCNA1,GABRG3,ITPKA,NPBWR1,OPRD1,HTR7,GCH1,HAP1,DRD2,SLC1A4,OPRL1,TACR1,ASIC2,GJC2,HTR5A,NQO1,MYRIP,NGEF,GABRE,SSTR4,CYBB,KIRREL3,HEPACAM,TRPV2,KCNC2,SYNPO,HTR1E,TXNRD2,PSEN2,SYN1,RIN3,MCHR1,CALY,MAPK8IP1,ATP1A3,BAALC,ATP2B3,HRH2,DRD1,CDK5R2,LRRTM1,SYT5,PCSK1,CYGB,NCS1,KCNIP1,CDHR1,KIF17,ATP6V0D1,CYP46A1,USH1G,TPGS1,HTR2A,ADORA1,RGS6,RET,SNCB,HCN2,SYP
GO:CC	cell body	GO:0044297	1.1649791360139815e-8	7.933681852487865	SLC6A2,GAL,VGF,TH,NGB,ADRA2A,GABRA5,PTPRN,TMEM132E,KCNA1,DRD2,SLC1A4,TBX21,TACR1,ACTG2,S100B,ASIC2,GJC2,HTR5A,NQO1,CYBB,TRPV2,TMPRSS3,KCNC2,SYNPO,TXNRD2,PSEN2,SYN1,RIN3,MAPK8IP1,ATP1A3,ASTN2,PITPNM1,SYT5,PCSK1,CYGB,HTR2A,ADORA1,RET,SNCB,SLC22A3
GO:CC	dendrite	GO:0030425	1.8449429545702488e-7	6.734017057634118	TH,HTR6,GABRA5,CHRM4,GRM7,HRH1,KCNA1,GABRG3,ITPKA,OPRD1,HTR7,HAP1,DRD2,SLC1A4,TACR1,ASIC2,HTR5A,NQO1,GABRE,CYBB,KIRREL3,KCNC2,SYNPO,HTR1E,TXNRD2,PSEN2,SYN1,RIN3,MAPK8IP1,HRH2,DRD1,PCSK1,NCS1,KCNIP1,KIF17,CYP46A1,TPGS1,HTR2A,ADORA1,RET,HCN2
GO:CC	dendritic tree	GO:0097447	2.0298713444406962e-7	6.692531487293746	TH,HTR6,GABRA5,CHRM4,GRM7,HRH1,KCNA1,GABRG3,ITPKA,OPRD1,HTR7,HAP1,DRD2,SLC1A4,TACR1,ASIC2,HTR5A,NQO1,GABRE,CYBB,KIRREL3,KCNC2,SYNPO,HTR1E,TXNRD2,PSEN2,SYN1,RIN3,MAPK8IP1,HRH2,DRD1,PCSK1,NCS1,KCNIP1,KIF17,CYP46A1,TPGS1,HTR2A,ADORA1,RET,HCN2
GO:CC	exocytic vesicle	GO:0070382	5.618027392106168e-7	6.250416147617254	TH,SYTL5,SLC18A3,LGI3,SLC6A17,SV2C,DYSF,OPRD1,BDNF,HAP1,DRD2,RAB3IL1,KIRREL3,SLC6A9,PSEN2,SYN1,ATP6V0C,SYT5,SYT9,MAL2,ATP6V0D1,ATP6V0A1,SYP
GO:CC	neuronal cell body	GO:0043025	7.907531744607198e-7	6.1019590559433725	SLC6A2,GAL,VGF,TH,NGB,ADRA2A,GABRA5,PTPRN,KCNA1,DRD2,SLC1A4,TBX21,S100B,ASIC2,GJC2,HTR5A,NQO1,CYBB,TMPRSS3,KCNC2,SYNPO,TXNRD2,PSEN2,RIN3,MAPK8IP1,ATP1A3,ASTN2,SYT5,PCSK1,CYGB,HTR2A,ADORA1,RET,SNCB,SLC22A3
GO:CC	presynaptic membrane	GO:0042734	1.5193E-06	5.818346587596255	SLC6A2,IL31RA,ADRA2A,GABRA5,KCNA1,OPRD1,SLC6A11,DRD2,PDE2A,CHRNA6,KCNC2,SLC6A9,PSEN2,CHRNB2,ATP2B3,DRD1,HTR2A,ADORA1,SYP
GO:CC	cell projection	GO:0042995	1.8986E-06	5.721557283742996	SLC6A2,GRP,PRLHR,TH,IL31RA,ADRA2A,DRD5,FFAR4,SLC18A3,HTR6,GABRA5,LGI3,PTPRN,CHRM4,PTPRH,GPR83,SLC6A17,AQP5,GRM7,HRH1,KCNA1,GABRG3,ITPKA,NPBWR1,GALR2,OPRD1,HTR7,GCH1,SPTB,HAP1,SLC6A11,PLCG2,DRD2,SLC1A4,OPRL1,TACR1,ACTG2,S100B,GALR3,ASIC2,GJC2,HTR5A,NQO1,MYRIP,NGEF,GABRE,SSTR4,CYBB,SLC7A5,KIRREL3,HEPACAM,TRPV2,SLC5A1,KCNC2,SYNPO,HTR1E,TSSK1B,TXNRD2,PSEN2,PRKAR1B,SYN1,SQSTM1,RIN3,MCHR1,CALY,MAPK8IP1,ATP1A3,BAALC,ATP2B3,HRH2,DRD1,CDK5R2,LRRTM1,SYT5,PCSK1,CYGB,NCS1,KCNIP1,CDHR1,KIF17,ATP6V0D1,CYP46A1,ATP8A2,USH1G,TPGS1,HTR2A,ADORA1,RGS6,RET,SNCB,PARD6A,ABLIM3,HCN2,SYP
GO:CC	plasma membrane bounded cell projection	GO:0120025	3.2948E-06	5.482165488979547	SLC6A2,GRP,PRLHR,TH,IL31RA,ADRA2A,DRD5,FFAR4,SLC18A3,HTR6,GABRA5,LGI3,PTPRN,CHRM4,PTPRH,GPR83,AQP5,GRM7,HRH1,KCNA1,GABRG3,ITPKA,NPBWR1,GALR2,OPRD1,HTR7,GCH1,HAP1,PLCG2,DRD2,SLC1A4,OPRL1,TACR1,ACTG2,S100B,GALR3,ASIC2,GJC2,HTR5A,NQO1,MYRIP,NGEF,GABRE,SSTR4,CYBB,SLC7A5,KIRREL3,HEPACAM,TRPV2,SLC5A1,KCNC2,SYNPO,HTR1E,TSSK1B,TXNRD2,PSEN2,PRKAR1B,SYN1,SQSTM1,RIN3,MCHR1,CALY,MAPK8IP1,ATP1A3,BAALC,ATP2B3,HRH2,DRD1,CDK5R2,LRRTM1,SYT5,PCSK1,CYGB,NCS1,KCNIP1,CDHR1,KIF17,ATP6V0D1,CYP46A1,USH1G,TPGS1,HTR2A,ADORA1,RGS6,RET,SNCB,PARD6A,ABLIM3,HCN2,SYP
GO:CC	synaptic vesicle	GO:0008021	3.9936E-06	5.3986343005221755	TH,SLC18A3,LGI3,SLC6A17,SV2C,DYSF,OPRD1,BDNF,HAP1,DRD2,KIRREL3,SLC6A9,PSEN2,SYN1,ATP6V0C,SYT5,SYT9,MAL2,ATP6V0D1,ATP6V0A1,SYP
GO:CC	synaptic membrane	GO:0097060	5.7676E-06	5.239003195569467	SLC6A2,IL31RA,ADRA2A,GABRA5,SRPX2,CHRM4,GRM7,KCNA1,GABRG3,OPRD1,SLC6A11,DRD2,GSG1L,HTR5A,PDE2A,GABRE,CHRNA6,KCNC2,SLC6A9,PSEN2,CHRNB2,ATP2B3,DRD1,LRFN2,LRRTM1,GABRQ,HTR2A,ADORA1,SYP
GO:CC	plasma membrane region	GO:0098590	9.1545E-06	5.038366555840495	SLC6A2,ADRA1B,IL31RA,ADRA2A,DRD5,FFAR4,SLC18A3,GABRA5,KL,SRPX2,SLC5A10,CHRM4,PTPRH,AQP5,GRM7,KCNA1,GABRG3,KCNE4,OPRD1,SLC6A11,PLCG2,DRD2,GSG1L,SHROOM1,SPHK1,HTR5A,PDE2A,SKAP1,GABRE,KCNA5,SLC7A5,CHRNA6,SLC5A1,KCNC2,MIP,SLC6A9,PSEN2,MCHR1,ABCG5,SLC9A3R2,ATP1A3,MLC1,SLC6A12,CHRNB2,ATP2B3,CYP4A11,PITPNM1,DRD1,LRFN2,LRRTM1,MAL2,CDHR1,KIF17,ATP6V0D1,SLC36A1,GABRQ,HTR2A,ADORA1,PARD6A,SLC22A3,SYP

GO:CC	axon	GO:0030424	1.4553E-05	4.837059783780106	TH,IL31RA,ADRA2A,SLC18A3,LGI3,PTPRN,GRM7,KCNA1,OPRD1,HAP1,DRD2,GJC2,NGEF,KIRREL3,HEPACAM,TRPV2,KCNC2,TXNRD2,PSEN2,SYN1,RIN3,CALY,MAPK8IP1,ATP1A3,ATP2B3,CDK5R2,LRRTM1,SYT5,PCSK1,NCS1,ATP6V0D1,TPGS1,HTR2A,ADORA1,RET,SNCB,HCN2,SYP
GO:CC	postsynapse	GO:0098794	4.8481E-05	4.314426158632706	ADRA2A,GABRA5,CHRM4,SLC6A17,GRM7,KCNA1,GABRG3,ITPKA,OPRD1,SPTB,HAP1,SLC6A11,DRD2,GSG1L,PAK6,ASIC2,HTR5A,CABP1,GABRE,CHRNA6,KCNC2,SYNPO,SLC6A9,PRKAR1B,SYN1,CALY,BAALC,CHRNB2,DRD1,LRFN2,ADGRA1,LRRTM1,NCS1,CYP46A1,GABRQ,HTR2A,ADORA1
GO:CC	synaptic vesicle membrane	GO:0030672	5.0068E-05	4.3004412088055615	SLC18A3,SLC6A17,SV2C,DYSF,OPRD1,DRD2,SLC6A9,SYN1,ATP6V0C,SYT5,SYT9,MAL2,ATP6V0D1,ATP6V0A1,SYP
GO:CC	exocytic vesicle membrane	GO:0099501	5.0068E-05	4.3004412088055615	SLC18A3,SLC6A17,SV2C,DYSF,OPRD1,DRD2,SLC6A9,SYN1,ATP6V0C,SYT5,SYT9,MAL2,ATP6V0D1,ATP6V0A1,SYP
GO:CC	postsynaptic membrane	GO:0045211	7.757E-05	4.110304120684957	ADRA2A,GABRA5,CHRM4,GRM7,KCNA1,GABRG3,OPRD1,SLC6A11,DRD2,GSG1L,HTR5A,GABRE,CHRNA6,KCNC2,SLC6A9,CHRNB2,DRD1,LRFN2,LRRTM1,GABRQ,HTR2A,ADORA1
GO:CC	transport vesicle	GO:0030133	0.00012487	3.903549415669324	DBH,VGF,TH,SYTL5,SLC18A3,LGI3,PTPRN,SLC6A17,SV2C,DYSF,OPRD1,BDNF,HAP1,DRD2,RAB3IL1,MYRIP,KIRREL3,SLC6A9,PSEN2,SYN1,ATP6V0C,SYT5,PCSK1,SYT9,MAL2,ATP6V0D1,ATP6V0A1,SYP
GO:CC	neuron projection terminus	GO:0044306	0.00030488	3.5158738256453703	TH,ADRA2A,SLC18A3,PTPRN,KCNA1,OPRD1,GCH1,DRD2,KCNC2,PCSK1,ATP6V0D1,ADORA1,SNCB,SYP
GO:CC	axon terminus	GO:0043679	0.00041447	3.3825120065320133	TH,ADRA2A,SLC18A3,PTPRN,KCNA1,OPRD1,DRD2,KCNC2,PCSK1,ATP6V0D1,ADORA1,SNCB,SYP
GO:CC	secretory vesicle	GO:0099503	0.00044698	3.3497144729830186	DBH,FOLR3,GAL,GRP,MS4A3,TH,SYTL5,SLC18A3,MMP8,DMBT1,LGI3,PTPRN,SERPINE1,SERPINA3,CYB561,SLC6A17,CARTPT,SV2C,DYSF,OPRD1,BDNF,QPCT,HAP1,DRD2,RAB3IL1,KLK13,SCG2,MYRIP,VEGFB,CYBB,KIRREL3,NPY,CTSD,TSSK1B,SLC6A9,PSEN2,SYN1,NFAM1,ATP6V0C,LAMP3,SYT5,PCSK1,SYT9,MAL2,ABCA3,ATP6V0D1,ATP6V0A1,SYP
GO:CC	dense core granule	GO:0031045	0.00050872	3.293525395660715	GRP,OPRD1,SCG2,MYRIP,NPY,SLC6A9,SYT5,SYT9
GO:CC	GABA-ergic synapse	GO:0098982	0.0009338	3.0297467028610914	ADRA2A,GABRA5,SLC6A17,GABRG3,SLC6A11,DRD2,KCNC2,NPY,ATP2B3,DRD1,LRRTM1
GO:CC	perinuclear region of cytoplasm	GO:0048471	0.00268367	2.5712714556285543	MS4A3,TH,KCNS2,KCNS1,HMOX1,BDNF,PLCG2,S100B,MYRIP,PDE2A,CABP1,ALDH1A2,PLEKHF1,NUPR1,KCNA5,CYBB,SLC5A1,AANAT,DGAT2,PSEN2,RHBDD2,MAPK8IP1,INHBA,MLC1,KRT18,LAMP3,HRAS,SYT5,PCSK1,NCS1,MAL2,PPARG,DYNC1I1,ATP6V0A1,ACHE,SYP
GO:CC	distal axon	GO:0150034	0.00506641	2.295299916149703	TH,ADRA2A,SLC18A3,PTPRN,KCNA1,OPRD1,HAP1,DRD2,NGEF,TRPV2,KCNC2,PSEN2,CDK5R2,LRRTM1,PCSK1,ATP6V0D1,ADORA1,SNCB,SYP
GO:CC	intracellular vesicle	GO:0097708	0.00544924	2.263663697248454	DBH,FOLR3,GAL,GRP,MS4A3,SYK,VGF,NEURL3,TH,SYTL5,FFAR4,SLC18A3,GPNMB,MMP8,DMBT1,LGI3,PTPRN,SERPINE1,SERPINA3,CYB561,MCOLN3,SLC6A17,AQP5,KCNA1,BOK,CARTPT,SV2C,MALL,DYSF,OPRD1,GCH1,BDNF,QPCT,HAP1,PLCG2,DRD2,SLC1A4,OPRL1,IL15RA,RAB3IL1,KLK13,SCG2,SPHK1,MYRIP,VEGFB,COLEC10,PLEKHF1,KIAA0319,CYBB,KIRREL3,TRPV2,SLC5A1,NPY,CTSD,TSSK1B,SLC6A9,PSEN2,PRKAR1B,SYN1,SQSTM1,RIN3,IL7R,CALY,NFAM1,ATP6V0B,MLC1,ATP6V0C,ASTN2,TEX261,LAMP3,SYT5,PCSK1,SYT9,MAL2,TMEM198,IRAK1,ABCA3,ATP6V0D1,ATP8A2,DYNC1I1,ATP6V0A1,HTR2A,IRAK2,RET,TICAM1,SYP

GO:CC	endomembrane system	GO:0012505	0.00589277	2.229680229041786	DBH,FOLR3,GAL,GRP,ADRA1B,MS4A3,VGF,NEURL3,TH,SYTL5,FFAR4,SLC18A3,SYNDIG1L,PTHLH,UGT3A2,GPNMB,P3H2,ATP10A,MMP8,DMBT1,COL8A1,IGFBP3,LGI3,PTPRN,P4HA3,BCAN,GOLT1A,TMEM119,SERPINE1,SYT17,SERPINA3,CYB561,MCOLN3,SLC6A17,AQP5,KCNA1,BOK,CARTPT,HMOX1,SV2C,MALL,DYSF,OPRD1,HAS1,HTR7,GCH1,BDNF,TRPM8,QPCT,HAP1,DRD2,IL15RA,RAB3IL1,PCYT2,KLK13,SCG2,HS3ST2,SPHK1,EPHX1,HS6ST3,MYRIP,PDE2A,RAET1G,SRXN1,CACNA1S,CABP1,VEGFB,COLEC10,PLEKHF1,KIAA0319,KCNA5,CYBB,KIRREL3,SLC5A1,TMPRSS3,DUSP26,NPY,SYNPO,GBA,PEMT,MGLL,DGAT2,CTSD,MIP,COL5A3,TSSK1B,SLC6A9,PSEN2,PRKAR1B,RHBDD2,SYN1,SQSTM1,UBASH3A,RIN3,MANEAL,HM13,CALY,MAPK8IP1,SLC9A3R2,NFAM1,CHAC1,ATP1A3,ATP6V0B,MLC1,ELOVL7,ATP6V0C,CYP4A11,ASTN2,PTDSS1,NENF,LAMP3,PITPNM1,DRD1,HRAS,REEP2,SLC37A1,LRRTM1,SYT5,PCSK1,SYT9,PLA2G4C,NCS1,COL8A2,MAL2,IRAK1,ABCA3,ATP6V0D1,FIBP,CYP46A1,ATP8A2,GALNT9,DYNC1I1,SLC36A1,ATP6V0A1,ZDHHC14,IRAK2,RET,SEC11C,TICAM1,DHCR7,ACHE,TMEM120A,SLC22A3,SYP
GO:CC	transport vesicle membrane	GO:0030658	0.00609581	2.214968684182206	DBH,SLC18A3,PTPRN,SLC6A17,SV2C,DYSF,OPRD1,DRD2,SLC6A9,SYN1,ATP6V0C,SYT5,SYT9,MAL2,ATP6V0D1,ATP6V0A1,SYP
GO:CC	cytoplasmic vesicle	GO:0031410	0.00880066	2.0554849246742735	DBH,FOLR3,GAL,GRP,MS4A3,SYK,VGF,NEURL3,TH,SYTL5,FFAR4,SLC18A3,GPNMB,MMP8,DMBT1,LGI3,PTPRN,SERPINE1,SERPINA3,CYB561,MCOLN3,SLC6A17,AQP5,KCNA1,BOK,CARTPT,SV2C,MALL,DYSF,OPRD1,GCH1,BDNF,QPCT,HAP1,DRD2,SLC1A4,OPRL1,IL15RA,RAB3IL1,KLK13,SCG2,SPHK1,MYRIP,VEGFB,COLEC10,PLEKHF1,KIAA0319,CYBB,KIRREL3,TRPV2,SLC5A1,NPY,CTSD,TSSK1B,SLC6A9,PSEN2,PRKAR1B,SYN1,SQSTM1,RIN3,IL7R,CALY,NFAM1,ATP6V0B,MLC1,ATP6V0C,ASTN2,TEX261,LAMP3,SYT5,PCSK1,SYT9,MAL2,TMEM198,IRAK1,ABCA3,ATP6V0D1,ATP8A2,DYNC1I1,ATP6V0A1,HTR2A,IRAK2,RET,TICAM1 SYP
GO:CC	vacuolar proton-transporting V-type ATPase, V0 domain	GO:0000220	0.015344903463707302	1.8140358394127452	ATP6V0B,ATP6V0C,ATP6V0D1,ATP6V0A1
GO:CC	proton-transporting V-type ATPase, V0 domain	GO:0033179	0.034916250489045564	1.456972399541134	ATP6V0B,ATP6V0C,ATP6V0D1,ATP6V0A1
GO:CC	apical plasma membrane	GO:0016324	0.0373311	1.4279292156543528	KL,SLC5A10,PTPRH,AQP5,KCNA1,KCNE4,SHROOM1,KCNA5,SLC7A5,SLC5A1,KCNC2,MIP,SLC6A9,PSEN2,ABCG5,SLC9A3R2,CYP4A11,MAL2,ATP6V0D1,SLC36A1,PARD6A,SLC22A3
GO:CC	transporter complex	GO:1990351	0.04202713	1.3764702630503296	ATP10A,GABRA5,KCNS2,KCNA1,GABRG3,KCNS1,KCNK4,CACNA1S,GABRE,KCNA5,CHRNA6,KCNC2,SCN2B,ABCG5,ATP1A3,ATP6V0B,CHRNB2,ATP6V0C,KCNIP1,ATP6V0D1,ATP8A2,GABRQ,HCN2

Suspended GO Terms Based on Uprgeulated Genes

source	***term_name***	***term_id***	***adj_p_value***	***neg_log10_adj_p_value***	***intersections***
GO:MF	extracellular matrix structural constituent	GO:0005201	3.518400681596042e-8	7.453654703850335	COL4A6,COL4A5,COL7A1,FMOD,MMRN1,COL13A1,ABI3BP,COL2A1,COL11A2,IMPG1,COL28A1,COL11A1,CD4,FBLN2,COL9A2,MUC4,COL9A1,NPNT,EFEMP1,COL27A1,LAMA1,EFEMP2,VCAN,HMCN1,PCOLCE,SBSPON,COL21A1,COL9A3,COL25A1,MUC6,COL19A1
GO:MF	extracellular matrix structural constituent conferring tensile strength	GO:0030020	1.4338783151132127e-7	6.8434877031432615	COL4A6,COL4A5,COL7A1,COL13A1,COL2A1,COL11A2,COL28A1,COL11A1,COL9A2,COL9A1,COL27A1,COL21A1,COL9A3,COL25A1,COL19A1

GO:MF	ion binding	GO:0043167	9.1129E-06	5.040344738428412	TRIM43,TRIM43B,TRIM49B,BIRC7,AIF1,CENPE,APOBEC3H,ATP1A4,MAPK15,NPEPL1,KDM4E,TIE1,TRIM49,LHX9,KLK4,MT1G,MSH5-SAPCD1,ACSS3,LIN28A,MECOM,ZNF83,GALNT5,CFTR,SPATA21,AMY2B,AGBL2,ANPEP,MAN2C1,INSRR,PRODH2,PLA2G4D,UCP1,LCN12,SLFN13,ZGRF1,CSAD,CACNA1F,HBA2,KIF14,PDK4,CYP3A4,TRIM49C,ADAT2,SPTLC3,ADAMTS20,PIF1,MMRN1,BCL6B,HDC,CYP3A43,AMY2A,CYP2F1,TRIM6,PLAG1,KIF18A,ADAM33,MYO15B,ZSCAN4,NLRP12,DQX1,VWA2,ZNF441,ZNF117,CCDC144A,MAP3K19,INO80B,DDX60,HRC,TRIM59,DDX4,ARL5C,ZFC3H1,UNC13D,ATAD5,UBA7,UPB1,MYO1A,PDE6C,SALL4,SIGLEC10,NRBP2,DDX43,TRIM51,DNAH12,ZAP70,KIF15,ITLN2,GALNT3,BTAF1,DMRTA1,TOP2A,TTK,ZMAT1,DUS1L,ZNF648,NAALAD2,ST18,P2RY4,POLQ,SMC4,CPSF4L,DNAH2,TRIM22,BCHE,TTLL6,ALOX15,ADAMTS6,ERN2,YY2,PLCZ1,KIF20B,SDCBP2,ITGA2B,GALNT12,XRCC2,DNAH11,ANG,ZBBX,CHKB,CAPN12,MTR,XIRP2,MYL7,CAPN11,USP45,CNGA4,DSG2,CDK1,ZNF518A,TNFSF10,FREM1,PDE6B,AIRE,MBNL3,ZNF730,SNAI1,PCK1,CPZ,DSC2,KIF18B,FANCM,YPEL4,STK26,DUOX2,LTK,KIF23,CAPS2,GSDMD,SBK3,CA4,COL2A1,ACTN3,RUFY4,COL11A2,UTRN,SGK2,ZNF90,ACSL5,HFM1,ZNF732,JAK3,COL11A1,MYO3A,DPEP1,MEGF6,PCDHGA12,KIF11,VWA8,CD4,FBLN2,ACSM4,ARG1,AGBL3,ZIC5,ADCY4,GRIN3B,CRABP2,FCN3,MLPH,GK5,ZNF107,MAPK13,CDHR5,NLRP6,MFNG,ADH6,DNHD1,TRIM45,CABP4,DCST1,SAR1B,ZNF600,SLC34A2,RFPL4B,MKI67,ACADL,PUS10,SRPK3,P2RX2,CALML6,NEIL1,L3MBTL1,PLCH1,PLA2G4B,REST,PBK,ZNF334,FBXO43,ADGRE2,NEK7,PCDHGA5,PLK4,PIWIL2,KIF12,MUC5B,ZNF680,IKZF1,ZNF100,STARD9,LIPI,RBBP6,ZNF138,CRACR2B,ZMYND15,LPAR4,OVOL1,KEL,ZSWIM2,BUB1,ZNF483,ITGB1BP2,KIF20A,ABCA1,ZNF121,LIN28B,COL9A1,ZNF217,ATP2A1,ZNF662,BUB1B,CHD1,ZNF80,JMJD1C,RHOH,TET1,ZNF382,RFPL3,NPNT,EFEMP1,COL27A1,STAB1,ZBTB18,GSDMB,MAP4K1,ADAMTS4,ZNF141,CSNK1A1L,ARAP3,ITGB7,NEK2,EFEMP2,ZNF846,BRCA1,TYW5,ATAD2,EME1,ZNF493,NEIL3,CALR3,KCND1,CYP3A5,PHF3,TRIM64B,IMPA2,ADAM7,SNX20,ADAMTS3,FTCD,VCAN,BRIP1,MYO15A,ROCK1,MKRN3,EBF1,TBC1D8B,SAT1,PLAGL1,CYP2A7,PKN2,STK31,CYP39A1,DPEP2,HMCN1,TCEA3,PAPPA,GAL3ST4,PKD1L2,PRICKLE4,CBR4,KCNT2,AGK,DNMT3L,STK3,ZNF431,C1S,RNF151,CHTF18,CAPRIN2,PTER,SMC2,ZNF92,RARG,P4HA1,RAD54B,MNAT1,PLS1,ACSM3,GALNT7,RRM2B,SCAF11,NUDT12,MELK,NR2E3,AOC3,GATA1,ESCO1,CUBN,GTPBP3,PRKD3,ZNF726,RFESD,PLCD1,MOB1B,AGAP9,ZNF678,TSSK3,RAPGEF6,MCM8,APOA5,ZNF563,TEX14,OMA1,RIPK3,ANGPT2,CRIP1,ZNF708,TEK,IKZF2,ENOSF1,ZNF624,KIF2C,MBNL2,POLI,FCHO2,TTLL3,OGT,FCER2,FRRS1,BAZ2B,THRB,BLM,SMC5,NEK5,ITGA2,PADI2,PIK3C2A,ITGA10,UHRF1,GSDMA,BAZ1A
GO:MF	microtubule motor activity	GO:0003777	0.00016912	3.7718124406403444	CENPE,KIF14,KIF18A,DNAH12,KIF15,DNAH2,KIF20B,DNAH11,KIF18B,KIF23,KIF11,DNHD1,KIF12,STARD9,KIF20A,KIF2C
GO:MF	cytoskeletal motor activity	GO:0003774	0.00051889	3.2849232546783944	CENPE,KIF14,KIF18A,MYO15B,MYO1A,DNAH12,KIF15,DNAH2,KIF20B,DNAH11,KIF18B,KIF23,MYO3A,KIF11,DNHD1,KIF12,STARD9,KIF20A,MYO15A,KIF2C
GO:MF	ATP-dependent activity	GO:0140657	0.00139241	2.856232987611491	CENPE,ATP1A4,MSH5-SAPCD1,CFTR,ATP6V0D2,ZGRF1,KIF14,PIF1,KIF18A,DQX1,DDX60,DDX4,ATAD5,UBA7,DDX43,DNAH12,KIF15,BTAF1,TOP2A,POLQ,SMC4,DNAH2,KIF20B,XRCC2,DNAH11,KIF18B,FANCM,KIF23,ACSL5,HFM1,MYO3A,KIF11,VWA8,ACSM4,DNHD1,TCIRG1,KIF12,STARD9,KIF20A,ABCA1,ATP2A1,CHD1,CECR2,ATAD2,BRIP1,CHTF18,SMC2,RAD54B,ACSM3,MCM8,KIF2C,BLM,SMC5

GO:MF	cation binding	GO:0043169	0.011138100606367898	1.9531888636061545	TRIM43,TRIM43B,TRIM49B,BIRC7,AIF1,APOBEC3H,ATP1A4,NPEPL1,KDM4E,TRIM49,LHX9,KLK4,MT1G,LIN28A,MECOM,ZNF83,GALNT5,SPATA21,AMY2B,AGBL2,ANPEP,MAN2C1,PLA2G4D,SLFN13,ZGRF1,CACNA1F,HBA2,CYP3A4,TRIM49C,ADAT2,ADAMTS20,PIF1,MMRN1,BCL6B,CYP3A43,AMY2A,CYP2F1,TRIM6,PLAG1,ADAM33,ZSCAN4,VWA2,ZNF441,ZNF117,INO80B,HRC,TRIM59,ZFC3H1,UNC13D,UPB1,PDE6C,SALL4,TRIM51,ITLN2,GALNT3,DMRTA1,TOP2A,ZMAT1,ZNF648,NAALAD2,ST18,CPSF4L,TRIM22,BCHE,TTLL6,ALOX15,ADAMTS6,ERN2,YY2,PLCZ1,ITGA2B,GALNT12,ANG,ZBBX,CAPN12,MTR,XIRP2,MYL7,CAPN11,USP45,DSG2,ZNF518A,TNFSF10,FREM1,PDE6B,AIRE,MBNL3,ZNF730,SNAI1,PCK1,CPZ,DSC2,YPEL4,STK26,DUOX2,CAPS2,CA4,COL2A1,ACTN3,RUFY4,COL11A2,UTRN,ZNF90,ZNF732,COL11A1,DPEP1,MEGF6,PCDHGA12,CD4,FBLN2,ACSM4,ARG1,AGBL3,ZIC5,ADCY4,GRIN3B,FCN3,MLPH,ZNF107,CDHR5,MFNG,ADH6,TRIM45,CABP4,DCST1,SAR1B,ZNF600,SLC34A2,RFPL4B,PUS10,CALML6,NEIL1,L3MBTL1,PLCH1,PLA2G4B,REST,ZNF334,FBXO43,ADGRE2,NEK7,PCDHGA5,PIWIL2,MUC5B,ZNF680,IKZF1,ZNF100,LIPI,RBBP6,ZNF138,CRACR2B,ZMYND15,OVOL1,KEL,ZSWIM2,ZNF483,ITGB1BP2,ABCA1,ZNF121,LIN28B,COL9A1,ZNF217,ATP2A1,ZNF662,ZNF80,JMJD1C,TET1,ZNF382,RFPL3,NPNT,EFEMP1,COL27A1,STAB1,ZBTB18,ADAMTS4,ZNF141,ARAP3,ITGB7,NEK2,EFEMP2,ZNF846,BRCA1,TYW5,EME1,ZNF493,NEIL3,CALR3,KCND1,CYP3A5,PHF3,TRIM64B,IMPA2,ADAM7,ADAMTS3,VCAN,BRIP1,ROCK1,MKRN3,EBF1,TBC1D8B,SAT1,PLAGL1,CYP2A7,CYP39A1,DPEP2,HMCN1,TCEA3,PAPPA,PKD1L2,PRICKLE4,DNMT3L,STK3,ZNF431,C1S,RNF151,CAPRIN2,PTER,ZNF92,RARG,P4HA1,MNAT1,PLS1,ACSM3,GALNT7,RRM2B,SCAF11,NUDT12,MELK,NR2E3,AOC3,GATA1,ESCO1,CUBN,PRKD3,ZNF726,RFESD,PLCD1,MOB1B,AGAP9,ZNF678,TSSK3,APOA5,ZNF563,OMA1,ANGPT2,CRIP1,ZNF708,IKZF2,ENOSF1,ZNF624,MBNL2,POLI,TTLL3,FCER2,FRRS1,BAZ2B,THRB,BLM,NEK5,ITGA2,PADI2,ITGA10,UHRF1,BAZ1A
GO:MF	metal ion binding	GO:0046872	0.012735452492839595	1.8949856198678425	TRIM43,TRIM43B,TRIM49B,BIRC7,AIF1,APOBEC3H,ATP1A4,NPEPL1,KDM4E,TRIM49,LHX9,KLK4,MT1G,LIN28A,MECOM,ZNF83,GALNT5,SPATA21,AMY2B,AGBL2,ANPEP,MAN2C1,PLA2G4D,SLFN13,ZGRF1,CACNA1F,HBA2,CYP3A4,TRIM49C,ADAT2,ADAMTS20,PIF1,MMRN1,BCL6B,CYP3A43,AMY2A,CYP2F1,TRIM6,PLAG1,ADAM33,ZSCAN4,VWA2,ZNF441,ZNF117,INO80B,HRC,TRIM59,ZFC3H1,UNC13D,UPB1,PDE6C,SALL4,TRIM51,ITLN2,GALNT3,DMRTA1,TOP2A,ZMAT1,ZNF648,NAALAD2,ST18,CPSF4L,TRIM22,TTLL6,ALOX15,ADAMTS6,ERN2,YY2,PLCZ1,ITGA2B,GALNT12,ANG,ZBBX,CAPN12,MTR,XIRP2,MYL7,CAPN11,USP45,DSG2,ZNF518A,TNFSF10,FREM1,PDE6B,AIRE,MBNL3,ZNF730,SNAI1,PCK1,CPZ,DSC2,YPEL4,STK26,DUOX2,CAPS2,CA4,COL2A1,ACTN3,RUFY4,COL11A2,UTRN,ZNF90,ZNF732,COL11A1,DPEP1,MEGF6,PCDHGA12,CD4,FBLN2,ACSM4,ARG1,AGBL3,ZIC5,ADCY4,FCN3,MLPH,ZNF107,CDHR5,MFNG,ADH6,TRIM45,CABP4,DCST1,SAR1B,ZNF600,SLC34A2,RFPL4B,PUS10,CALML6,NEIL1,L3MBTL1,PLCH1,PLA2G4B,REST,ZNF334,FBXO43,ADGRE2,NEK7,PCDHGA5,PIWIL2,MUC5B,ZNF680,IKZF1,ZNF100,LIPI,RBBP6,ZNF138,CRACR2B,ZMYND15,OVOL1,KEL,ZSWIM2,ZNF483,ITGB1BP2,ZNF121,LIN28B,COL9A1,ZNF217,ATP2A1,ZNF662,ZNF80,JMJD1C,TET1,ZNF382,RFPL3,NPNT,EFEMP1,COL27A1,STAB1,ZBTB18,ADAMTS4,ZNF141,ARAP3,ITGB7,NEK2,EFEMP2,ZNF846,BRCA1,TYW5,EME1,ZNF493,NEIL3,CALR3,KCND1,CYP3A5,PHF3,TRIM64B,IMPA2,ADAM7,ADAMTS3,VCAN,BRIP1,ROCK1,MKRN3,EBF1,TBC1D8B,PLAGL1,CYP2A7,CYP39A1,DPEP2,HMCN1,TCEA3,PAPPA,PKD1L2,PRICKLE4,DNMT3L,STK3,ZNF431,C1S,RNF151,CAPRIN2,PTER,ZNF92,RARG,P4HA1,MNAT1,PLS1,ACSM3,GALNT7,RRM2B,SCAF11,NUDT12,MELK,NR2E3,AOC3,GATA1,ESCO1,CUBN,PRKD3,ZNF726,RFESD,PLCD1,MOB1B,AGAP9,ZNF678,TSSK3,ZNF563,OMA1,ANGPT2,CRIP1,ZNF708,IKZF2,ENOSF1,ZNF624,MBNL2,POLI,TTLL3,FCER2,FRRS1,BAZ2B,THRB,BLM,NEK5,ITGA2,PADI2,ITGA10,UHRF1,BAZ1A
GO:MF	zinc ion binding	GO:0008270	0.03459639	1.460969165566433	TRIM43,TRIM43B,TRIM49B,APOBEC3H,TRIM49,MT1G,LIN28A,AGBL2,ANPEP,SLFN13,ZGRF1,TRIM49C,ADAT2,ADAMTS20,TRIM6,ADAM33,ZNF117,TRIM59,UPB1,TRIM51,ZMAT1,ST18,TRIM22,ZBBX,MTR,USP45,TNFSF10,AIRE,CPZ,CA4,UTRN,ZNF90,DPEP1,CD4,AGBL3,ADH6,TRIM45,NEIL1,L3MBTL1,RBBP6,ZSWIM2,ITGB1BP2,LIN28B,TET1,ADAMTS4,BRCA1,NEIL3,TRIM64B,ADAMTS3,TCEA3,PAPPA,PRICKLE4,RNF151,PTER,ZNF92,RARG,MNAT1,NUDT12,NR2E3,GATA1,ESCO1,CRIP1,THRB,BLM,UHRF1

GO:MF	transition metal ion binding	GO:0046914	0.04386818	1.3578504065671093	TRIM43,TRIM43B,TRIM49B,APOBEC3H,NPEPL1,TRIM49,MT1G,LIN28A,AGBL2,ANPEP,SLFN13,ZGRF1,HBA2,CYP3A4,TRIM49C,ADAT2,ADAMTS20,CYP3A43,CYP2F1,TRIM6,ADAM33,ZNF117,TRIM59,UPB1,TRIM51,GALNT3,ZMAT1,ST18,TRIM22,ALOX15,ANG,ZBBX,MTR,USP45,TNFSF10,AIRE,PCK1,CPZ,CA4,UTRN,ZNF90,DPEP1,CD4,ARG1,AGBL3,ADH6,TRIM45,NEIL1,L3MBTL1,RBBP6,ZSWIM2,ITGB1BP2,LIN28B,TET1,ADAMTS4,BRCA1,TYW5,NEIL3,CYP3A5,TRIM64B,ADAMTS3,CYP2A7,CYP39A1,TCEA3,PAPPA,PRICKLE4,RNF151,PTER,ZNF92,RARG,P4HA1,MNAT1,NUDT12,NR2E3,AOC3,GATA1,ESCO1,CRIP1,THRB,BLM,UHRF1
GO:MF	protein binding	GO:0005515	0.04533566	1.3435600766088291	TRIM43,PRAME,TRIM43B,CDC20B,TRIM49B,BIRC7,MBD3L2,AIF1,CENPE,TMPRSS15,CCDC152,APOBEC3H,ATP1A4,RGL3,MAPK15,LPAR6,NPEPL1,COL4A6,BST2,TIE1,TRIM49,LRRC74B,GIPR,MPZL2,WSB1,LHX9,KLK4,HESX1,MT1G,POTEF,MSH5-SAPCD1,ACSS3,CENPF,SCNN1A,LEUTX,ASPM,LIN28A,MECOM,ZNF83,GOLGA8A,CFTR,TAC3,AGBL2,AASS,NOXA1,INSRR,ATP6V0D2,RAD51AP2,C1QTNF9B,GMNC,ANKRD18A,COL4A5,HAVCR1,TRH,LMNTD2,COL7A1,KLHL6,MIS18BP1,CEP290,TRAF3IP3,ANKRD33,FAM166A,CACNA1F,HBA2,KIF14,CD300LG,PDK4,CYP3A4,TRIM49C,GOLGA6A,FAM111B,UACA,ADAT2,FMOD,SPTLC3,HSF4,GNRH1,MMRN1,NUF2,BCL6B,CEP152,CMTM5,GOLGA8B,HDC,MBD3L3,WDR87,TRIM6,CHMP4C,KIF18A,ERVH48-1,ADAM33,MYO15B,ZSCAN4,NLRP12,VWA2,ZNF441,TTC14,MUC1,LRRC70,LXN,NPHP3-ACAD11,IFITM1,CLDN18,DAPL1,AP1G2,INO80B,COL13A1,DDX60,CHAD,HRC,TRIM59,SLURP1,TSPO2,MYCBPAP,BTBD8,TMA16,C16ORF89,PMEL,ZFC3H1,UNC13D,ATAD5,UBA7,CORO6,CCDC14,HMGB2,TMEM92,PSTPIP1,ZAN,SEC31B,UPB1,DEPDC1,MYO1A,PDE6C,AMT,C18ORF54,SALL4,SIGLEC10,SLCO1A2,DDX43,PRTG,TRIM51,DNAH12,GOLGA4,BRDT,LYPD2,IRS4,ZAP70,BTNL2,MSLN,ABI3BP,KIF15,RDM1,BTAF1,HSPB2,FAM72D,POMC,DUOXA1,DMRTA1,TOP2A,BRCA2,TTK,ANGPTL3,SLC17A9,MPHOSPH10,DUS1L,ZNF648,MST1,PAX5,RBM43,GPR78,NAALAD2,DLGAP5,P2RY4,PHLDB2,HMMR,NTF4,LTB,POLQ,HEMK1,KPNA7,SMC4,RSPO1,RBM47,NEAT1,LUC7L3,CD37,DNAH2,TRIM22,INTU,ECT2,CLDN10,BCHE,TTLL6,ALOX15,LTBR,NOSTRIN,ERN2,KIF20B,ROBO4,ATG16L2,SPAG8,AGER,KCNN4,SDCBP2,SPN,PCP2,POU2F2,CENPC,ITGA2B,CNTRL,XRCC2,LENG8,HMGA2,PLEKHH2,DNAH11,TPR,ANG,ROBO3,UNC5CL,LRRC19,MTR,XIRP2,CFAP70,ARR3,RANBP3L,NRG4,CGB7,MYL7,HPN,SLC35A3,USP45,C6ORF25,DSG2,WDR11,RAD51AP1,CDK1,C1QTNF8,UPK3A,PDIA2,TNFSF10,ANLN,COQ3,FREM1,PDE6B,AIRE,CCDC150,CPT1B,KCTD4,MBNL3,SNAI1,CPZ,DSC2,KIF18B,FANCM,TPBG,STK26,DUOX2,LTK,KIF23,IL11RA,GSDMD,INPP5D,SBK3,SLC26A5,CA4,KRTAP19-1,IQCF1,COL2A1,SPINK13,GTF3C3,LIPH,ACTN3,RSRP1,RUFY4,PRAM1,COL11A2,CEP44,RNPC3,WDR64,TBC1D32,GDF7,IFI44,NTN5,UTRN,PTPN7,SGK2,CCDC88B,OLIG3,KRTCAP3,ETAA1,CD207,GRIA2,GPHA2,RHBDL1,CCDC17,NUSAP1,KLHL14,PRAP1,CFAP58,ADIRF,ACSL5,CD164L2,CEP162,AVPR2,JAK3,HHIPL2,RBP3,ACKR4,MYO3A,DPEP1,ARPC4-TTLL3,HPR,C1QTNF9,TMEM106A,NKG7,RHBG,MEGF6,F11,FBXO24,CRYBG3,KIF11,UFL1,VWA8,LRIG3,SH3D21,CD4,FBLN2,EXPH5,LRRTM2,WNT5A,FAM72B,ANP32E,ARG1,PREX2,PTPN13,LY6G6C,ADCY4,POU6F2,POF1B,CRABP2,CEP55,FCN3,EREG,MXD3,MLPH,ARHGAP11B,ZNF107,EID3,MAPK13,HIF3A,CDHR5,COL20A1,TROAP,BORA,NEB,CREB3L3,UVSSA,DNHD1,RLN2,TRIM45,CENPK,CABP4,DCST1,TTC26,TCIRG1,DIAPH3,RPGR,SAR1B,ZNF600,SLC34A2,RFPL4B,MKI67,ACADL,TMPRSS6,BCL2L2-
GO:MF	ATP binding	GO:0005524	0.04807474	1.318083099891188	CENPE,ATP1A4,MAPK15,TIE1,MSH5-SAPCD1,ACSS3,CFTR,INSRR,SLFN13,KIF14,PDK4,PIF1,KIF18A,MYO15B,NLRP12,DQX1,MAP3K19,DDX60,DDX4,ATAD5,UBA7,MYO1A,NRBP2,DDX43,DNAH12,ZAP70,KIF15,BTAF1,TOP2A,TTK,P2RY4,POLQ,SMC4,DNAH2,TTLL6,ERN2,KIF20B,XRCC2,DNAH11,CHKB,CDK1,KIF18B,FANCM,STK26,LTK,KIF23,SBK3,SGK2,ACSL5,HFM1,JAK3,MYO3A,KIF11,VWA8,ACSM4,ADCY4,GK5,MAPK13,NLRP6,DNHD1,MKI67,SRPK3,P2RX2,PBK,NEK7,PLK4,KIF12,STARD9,BUB1,KIF20A,ABCA1,ATP2A1,BUB1B,CHD1,MAP4K1,CSNK1A1L,NEK2,ATAD2,BRIP1,MYO15A,ROCK1,PKN2,STK31,KCNT2,AGK,STK3,CHTF18,SMC2,RAD54B,ACSM3,MELK,PRKD3,TSSK3,MCM8,TEX14,RIPK3,TEK,KIF2C,TTLL3,BLM,SMC5,NEK5,PIK3C2A

GO:BP	nuclear division	GO:0000280	6.005681675231414e-15	14.22143769084403	CENPE,MAPK15,MSH5-SAPCD1,CENPF,ASPM,KIF14,NUF2,CHMP4C,KIF18A,DDX4,BRDT,KIF15,TOP2A,BRCA2,TTK,MTBP,DLGAP5,SMC4,KIF20B,CENPC,TPR,RAD51AP1,CDK1,ANLN,KIF18B,FANCM,KIF23,NUSAP1,PRAP1,HFM1,KIF11,WNT5A,EREG,BORA,CENPK,MKI67,NDC80,L3MBTL1,CDC25C,FBXO43,PIWIL2,CDCA2,BUB1,KNTC1,BUB1B,FANCD2,MYBL1,NEK2,CCNB2,EME1,SPICE1,SGOL1,SYCP2L,BRIP1,NCAPG,CDC14A,DNMT3L,SMC2,PTTG1,RAD54B,MAD2L1,CKS2,SYCP2,TEX14,KIF2C,SPC24,SPAG5,ANKRD31
GO:BP	organelle fission	GO:0048285	1.1510064576535433e-13	12.938922239779911	CENPE,MAPK15,MSH5-SAPCD1,CENPF,ASPM,KIF14,NUF2,CHMP4C,KIF18A,DDX4,BRDT,KIF15,TOP2A,BRCA2,TTK,MTBP,DLGAP5,SMC4,KIF20B,CENPC,TPR,RAD51AP1,CDK1,ANLN,KIF18B,FANCM,KIF23,NUSAP1,PRAP1,HFM1,KIF11,WNT5A,EREG,BORA,CENPK,MKI67,C11ORF65,NDC80,L3MBTL1,CDC25C,FBXO43,PIWIL2,CDCA2,BUB1,MTFR2,KNTC1,BUB1B,FANCD2,MYBL1,NEK2,CCNB2,EME1,SPICE1,SGOL1,SYCP2L,BRIP1,NCAPG,CDC14A,DNMT3L,SMC2,PTTG1,RAD54B,MAD2L1,CKS2,SYCP2,TEX14,KIF2C,SPC24,SPAG5,ANKRD31
GO:BP	chromosome segregation	GO:0007059	2.2967046442125357e-9	8.638894851403224	CENPE,MAPK15,MSH5-SAPCD1,CENPF,ASPM,KIF14,NUF2,GOLGA8B,CHMP4C,KIF18A,KIF15,TOP2A,TTK,DLGAP5,SMC4,ECT2,CENPC,TPR,CDK1,KIF18B,KIF23,NUSAP1,PRAP1,KIF11,CENPK,MKI67,NDC80,NEK7,SASS6,STARD9,CDCA2,BUB1,HJURP,KNTC1,BUB1B,FANCD2,CENPQ,NEK2,CCNB2,BRCA1,SPICE1,SGOL1,BRIP1,CENPJ,NCAPG,SMC2,PTTG1,MAD2L1,SKA1,SYCP2,TEX14,KIF2C,SPC24,SMC5,SPAG5,ANKRD31
GO:BP	mitotic nuclear division	GO:0140014	3.3387146316294003e-9	8.476420699605402	CENPE,CENPF,KIF14,NUF2,CHMP4C,KIF18A,KIF15,TTK,MTBP,DLGAP5,SMC4,KIF20B,CENPC,TPR,CDK1,ANLN,KIF18B,KIF23,NUSAP1,PRAP1,KIF11,EREG,BORA,CENPK,MKI67,NDC80,L3MBTL1,CDC25C,FBXO43,CDCA2,BUB1,KNTC1,BUB1B,NEK2,SPICE1,SGOL1,NCAPG,CDC14A,SMC2,MAD2L1,TEX14,KIF2C,SPC24,SPAG5
GO:BP	meiotic cell cycle process	GO:1903046	5.723554002808167e-9	8.242334215175326	MAPK15,MSH5-SAPCD1,ASPM,NUF2,KIF18A,DDX4,BRDT,TOP2A,BRCA2,TTK,SMC4,CENPC,RAD51AP1,FANCM,HFM1,WNT5A,EREG,NDC80,CDC25C,FBXO43,PIWIL2,OVOL1,FANCD2,MYBL1,CCNB2,EME1,SGOL1,SYCP2L,BRIP1,DNMT3L,SMC2,PTTG1,RAD54B,CKS2,SYCP2,TEX14,ANKRD31
GO:BP	meiotic nuclear division	GO:0140013	5.734050287226998e-9	8.2415385	MAPK15,MSH5-SAPCD1,ASPM,NUF2,KIF18A,DDX4,BRDT,TOP2A,BRCA2,TTK,SMC4,CENPC,RAD51AP1,FANCM,HFM1,WNT5A,EREG,NDC80,FBXO43,PIWIL2,FANCD2,MYBL1,CCNB2,EME1,SGOL1,SYCP2L,BRIP1,DNMT3L,SMC2,PTTG1,RAD54B,CKS2,SYCP2,TEX14,ANKRD31
GO:BP	sexual reproduction	GO:0019953	1.387506344410087e-8	7.857765022377544	ATP1A4,MAPK15,MSH5-SAPCD1,ASPM,LIN28A,CFTR,PRSS37,NUF2,KIF18A,DDX4,MYCBPAP,EQTN,HMGB2,ZAN,BRDT,SLC9C1,GALNT3,TOP2A,BRCA2,TTK,MST1,PAX5,SMC4,FSIP2,PLCZ1,SPAG8,CENPC,XRCC2,ANG,CGB7,RAD51AP1,CDK1,FANCM,DUOX2,IQCF1,SPINK13,CFAP58,HFM1,WNT5A,EREG,TDRP,FUT6,DNHD1,DCST1,TTC26,DCST2,BCL2L2-PABPN1,NDC80,ACRBP,DLEC1,CDC25C,FBXO43,PIWIL2,TDRD6,NODAL,ZMYND15,OVOL1,CATSPERG,SPESP1,MNS1,FANCD2,LEP,GJA1,CECR2,MYBL1,NEK2,EXD1,CCNB2,EME1,CALR3,SPEM1,DIAPH2,SGOL1,CFAP69,SPANXB1,SYCP2L,BRIP1,CFAP44,CD9,DNMT3L,CD46,RNF151,SMC2,CFAP43,PTTG1,CCNB3,RAD54B,CATSPER4,IZUMO1,TSSK3,MCM8,TMF1,CKS2,SPAG4,PABPC1L,SYCP2,TEX14,SKIL,ANGPT2,SPAG17,KLK14,ANKRD31
GO:BP	nuclear chromosome segregation	GO:0098813	5.4489278289482366e-8	7.263688944288423	CENPE,MAPK15,MSH5-SAPCD1,CENPF,ASPM,KIF14,NUF2,CHMP4C,KIF18A,KIF15,TOP2A,TTK,DLGAP5,SMC4,ECT2,CENPC,TPR,CDK1,KIF18B,KIF23,NUSAP1,PRAP1,KIF11,CENPK,NDC80,BUB1,KNTC1,BUB1B,FANCD2,CENPQ,NEK2,CCNB2,SPICE1,SGOL1,BRIP1,NCAPG,SMC2,PTTG1,MAD2L1,SYCP2,TEX14,KIF2C,SPC24,SPAG5,ANKRD31
GO:BP	meiotic cell cycle	GO:0051321	6.372306469767219e-8	7.195703345405729	MAPK15,MSH5-SAPCD1,ASPM,NUF2,KIF18A,DDX4,BRDT,TOP2A,BRCA2,TTK,SMC4,CENPC,XRCC2,RAD51AP1,FANCM,HFM1,WNT5A,EREG,NDC80,CDC25C,FBXO43,PIWIL2,OVOL1,MNS1,FANCD2,MYBL1,NEK2,EXD1,CCNB2,EME1,SGOL1,SYCP2L,BRIP1,DNMT3L,SMC2,PTTG1,CCNB3,RAD54B,CKS2,SYCP2,TEX14,ANKRD31

GO:BP	reproductive process	GO:0022414	7.031918519240514e-8	7.1529261701938465	ATP1A4,MAPK15,LHX9,HESX1,MSH5-SAPCD1,ASPM,LIN28A,CFTR,TAC3,INSRR,PRSS37,GNRH1,NUF2,PLAG1,KIF18A,CCDC39,DDX4,MYCBPAP,EQTN,HMGB2,ZAN,BRDT,SLC9C1,GALNT3,DMRTA1,TOP2A,BRCA2,TTK,MST1,PAX5,SMC4,FSIP2,PLCZ1,SPAG8,CENPC,XRCC2,DNAH11,ANG,CGB7,DSG2,RAD51AP1,CDK1,TNFSF10,SNAI1,FANCM,DUOX2,IQCF1,SPINK13,GDF7,CFAP58,HFM1,WNT5A,EREG,TDRP,FUT6,DNHD1,RLN2,DCST1,TTC26,DCST2,BCL2L2-PABPN1,NDC80,ACRBP,PLA2G4B,DLEC1,CDC25C,FBXO43,PLK4,PIWIL2,TDRD6,NODAL,ZMYND15,OVOL1,CATSPERG,SPESP1,MNS1,FANCD2,LEP,GJA1,EDNRB,CECR2,MYBL1,NEK2,EXD1,CCNB2,EME1,CALR3,SPEM1,DIAPH2,SGOL1,CFAP69,SPANXB1,SYCP2L,BRIP1,NHLH2,CFAP44,PAPPA,CD9,DNMT3L,STK3,CD46,RNF151,SMC2,CFAP43,RARG,PTTG1,CCNB3,RAD54B,CATSPER4,IZUMO1,GATA1,TSSK3,MCM8,TMF1,CKS2,GAPDHS,SPAG4,PABPC1L,SYCP2,TEX14,SKIL,ANGPT2,CRIP1,SPAG17,KLK14,TTLL3,THRB,ITGA2,ANKRD31
GO:BP	mitotic sister chromatid segregation	GO:0000070	1.0795275149621306e-7	6.966766283905076	CENPE,CENPF,KIF14,NUF2,CHMP4C,KIF18A,KIF15,TTK,DLGAP5,SMC4,CENPC,TPR,CDK1,KIF18B,KIF23,NUSAP1,PRAP1,KIF11,CENPK,NDC80,BUB1,KNTC1,BUB1B,NEK2,SPICE1,SGOL1,NCAPG,SMC2,MAD2L1,TEX14,KIF2C,SPC24,SPAG5
GO:BP	reproduction	GO:0000003	1.0909254729076352e-7	6.962204917432294	ATP1A4,MAPK15,LHX9,HESX1,MSH5-SAPCD1,ASPM,LIN28A,CFTR,TAC3,INSRR,PRSS37,GNRH1,NUF2,PLAG1,KIF18A,CCDC39,DDX4,MYCBPAP,EQTN,HMGB2,ZAN,BRDT,SLC9C1,GALNT3,DMRTA1,TOP2A,BRCA2,TTK,MST1,PAX5,SMC4,FSIP2,PLCZ1,SPAG8,CENPC,XRCC2,DNAH11,ANG,CGB7,DSG2,RAD51AP1,CDK1,TNFSF10,SNAI1,FANCM,DUOX2,IQCF1,SPINK13,GDF7,CFAP58,HFM1,WNT5A,EREG,TDRP,FUT6,DNHD1,RLN2,DCST1,TTC26,DCST2,BCL2L2-PABPN1,NDC80,ACRBP,PLA2G4B,DLEC1,CDC25C,FBXO43,PLK4,PIWIL2,TDRD6,NODAL,ZMYND15,OVOL1,CATSPERG,SPESP1,MNS1,FANCD2,LEP,GJA1,EDNRB,CECR2,MYBL1,NEK2,EXD1,CCNB2,EME1,CALR3,SPEM1,DIAPH2,SGOL1,CFAP69,SPANXB1,SYCP2L,BRIP1,NHLH2,CFAP44,PAPPA,CD9,DNMT3L,STK3,CD46,RNF151,SMC2,CFAP43,RARG,PTTG1,CCNB3,RAD54B,CATSPER4,IZUMO1,GATA1,TSSK3,MCM8,TMF1,CKS2,GAPDHS,SPAG4,PABPC1L,SYCP2,TEX14,SKIL,ANGPT2,CRIP1,SPAG17,KLK14,TTLL3,THRB,ITGA2,ANKRD31
GO:BP	cell cycle process	GO:0022402	4.3890600573603803e-7	6.357628476479757	AIF1,CENPE,MAPK15,MSH5-SAPCD1,CENPF,ASPM,KIF14,NUF2,CEP152,GOLGA8B,CHMP4C,KIF18A,MUC1,DDX4,SLC39A5,ATAD5,BRDT,KIF15,TOP2A,BRCA2,TTK,MTBP,DLGAP5,SMC4,ECT2,KIF20B,CENPC,XRCC2,HMGA2,TPR,RAD51AP1,CDK1,ANLN,KIF18B,FANCM,KIF23,CEP44,ETAA1,NUSAP1,PRAP1,HFM1,KIF11,UFL1,WNT5A,CEP55,EREG,BORA,CENPK,MKI67,NDC80,L3MBTL1,CDC25C,FBXO43,NEK7,PLK4,PIWIL2,SASS6,STARD9,CCND2,CDCA2,OVOL1,CCNA2,BUB1,KIF20A,HJURP,IQGAP3,KNTC1,BUB1B,FANCD2,GJA1,CENPQ,CECR2,MYBL1,CEP135,NEK2,CCNB2,BRCA1,EME1,SPICE1,SGOL1,SYCP2L,BRIP1,ROCK1,PAGR1,CENPJ,PKN2,NCAPG,CDC14A,RTTN,DNMT3L,SMC2,PTTG1,CCNB3,RAD54B,MNAT1,RRM2B,MELK,MAD2L1,ESCO1,SKA1,CKS2,SYCP2,TEX14,CCNL2,KIF2C,SPC24,CKAP2,BLM,SMC5,SPAG5,ANKRD31
GO:BP	cell division	GO:0051301	1.0532E-06	5.977506207275486	CENPE,CENPF,ASPM,MIS18BP1,KIF14,NUF2,CHMP4C,TOP2A,BRCA2,SMC4,INTU,ECT2,KIF20B,CENPC,CNTRL,HMGA2,TPR,CDK1,ANLN,KIF18B,KIF23,NUSAP1,KIF11,CEP55,EREG,BORA,NDC80,MACC1,CDC25C,LRRCC1,CD2AP,CCND2,CDCA2,CCNA2,BUB1,KIF20A,IQGAP3,KNTC1,BUB1B,CDCA7L,CECR2,NEK2,CCNB2,ITGB3BP,SPICE1,SGOL1,BRIP1,ROCK1,CENPJ,PKN2,NCAPG,CDC14A,HMCN1,SMC2,PTTG1,CCNB3,BOD1L1,MAD2L1,SKA1,CKS2,SYCP2,TEX14,KIF2C,SPC24,CKAP2,BLM,SMC5,NEDD1,SPAG5
GO:BP	sister chromatid segregation	GO:0000819	1.6853E-06	5.7733264275405105	CENPE,MAPK15,CENPF,KIF14,NUF2,CHMP4C,KIF18A,KIF15,TOP2A,TTK,DLGAP5,SMC4,CENPC,TPR,CDK1,KIF18B,KIF23,NUSAP1,PRAP1,KIF11,CENPK,NDC80,BUB1,KNTC1,BUB1B,NEK2,SPICE1,SGOL1,NCAPG,SMC2,MAD2L1,TEX14,KIF2C,SPC24,SPAG5
GO:BP	regulation of nuclear division	GO:0051783	1.7691E-06	5.752256935951024	CENPF,NUF2,TTK,MTBP,DLGAP5,KIF20B,TPR,RAD51AP1,NUSAP1,PRAP1,WNT5A,EREG,BORA,MKI67,NDC80,L3MBTL1,CDC25C,FBXO43,PIWIL2,CDCA2,BUB1,KNTC1,BUB1B,NEK2,MAD2L1,TEX14,SPC24
GO:BP	regulation of mitotic nuclear division	GO:0007088	1.9837E-06	5.702520462830828	CENPF,NUF2,TTK,MTBP,DLGAP5,KIF20B,TPR,NUSAP1,PRAP1,EREG,BORA,MKI67,NDC80,L3MBTL1,CDC25C,FBXO43,CDCA2,BUB1,KNTC1,BUB1B,NEK2,MAD2L1,TEX14,SPC24

GO:BP	microtubule-based process	GO:0007017	7.3294E-06	5.1349338207659665	CENPE,ATP1A4,MAPK15,ASPM,KIF14,NUF2,CEP152,GOLGA8B,CHMP4C,KIF18A,CCDC39,DDX4,DNAH12,SLC9C1,KIF15,BRCA2,TTK,MST1,DLGAP5,PHLDB2,DNAH2,INTU,FSIP2,TTLL6,CFAP74,KIF20B,XRCC2,DNAH11,TPR,CFAP70,CDK1,KIF18B,KIF23,IQCF1,CEP44,CCDC88B,NUSAP1,CFAP58,KIF11,BORA,DNHD1,CCDC78,TTC26,RPGR,NDC80,NEK7,PLK4,KIF12,SASS6,STARD9,AKAP9,LCA5,KIF20A,OFD1,MNS1,GJA1,CEP135,NEK2,CCNB2,BRCA1,SPICE1,SPEM1,SGOL1,CFAP69,ROCK1,GCC2,CFAP44,CENPJ,CDC14A,RTTN,IFT74,CFAP43,CCDC66,MAD2L1,CATSPER4,SKA1,TMF1,GAPDHS,SPAG17,CCNL2,KIF2C,CKAP2,TTLL3,NEDD1,RFX3,SPAG5
GO:BP	cell cycle	GO:0007049	2.0127E-05	4.696230471596228	BIRC7,AIF1,CENPE,MAPK15,MSH5-SAPCD1,CENPF,ASPM,MECOM,GMNC,MIS18BP1,KIF14,NUF2,CEP152,GOLGA8B,CHMP4C,KIF18A,MUC1,INO80B,DDX4,SLC39A5,ATAD5,BRDT,KIF15,TOP2A,BRCA2,TTK,MTBP,DLGAP5,SMC4,ECT2,KIF20B,SPAG8,CENPC,CNTRL,XRCC2,HMGA2,TPR,RAD51AP1,CDK1,ANLN,KIF18B,FANCM,KIF23,CEP44,ETAA1,NUSAP1,PRAP1,HFM1,KIF11,UFL1,WNT5A,CEP55,EREG,MAPK13,BORA,CENPK,MKI67,NDC80,L3MBTL1,CDC25C,PBK,LRRCC1,FBXO43,NEK7,PLK4,CD2AP,PIWIL2,IKZF1,SASS6,STARD9,CCND2,CDCA2,OVOL1,CCNA2,BUB1,KIF20A,HJURP,IQGAP3,KNTC1,BUB1B,MNS1,FANCD2,LEP,GJA1,CENPQ,CECR2,MYBL1,CEP135,NEK2,EXD1,CCNB2,BRCA1,ITGB3BP,EME1,BTG3,SPICE1,SGOL1,SYCP2L,BRIP1,ROCK1,WDHD1,PLAGL1,PAGR1,CENPJ,PKN2,NCAPG,CDC14A,RTTN,HMCN1,DNMT3L,CHTF18,SMC2,PTTG1,CCNB3,RAD54B,MNAT1,RRM2B,BOD1L1,MELK,MAD2L1,ESCO1,SKA1,MCM8,CKS2,SYCP2,TEX14,SKIL,CCNL2,KIF2C,SPC24,CKAP2,BLM,SMC5,NEDD1,RIF1,SPAG5,UHRF1,ANKRD31
GO:BP	kinetochore organization	GO:0051383	2.4045E-05	4.6189671334903135	CENPE,CENPF,NUF2,DLGAP5,SMC4,CENPC,CENPK,NDC80,KNTC1,SMC2
GO:BP	meiosis I cell cycle process	GO:0061982	3.9183E-05	4.406906063516385	MAPK15,MSH5-SAPCD1,DDX4,BRDT,TOP2A,BRCA2,CENPC,RAD51AP1,FANCM,HFM1,NDC80,CDC25C,PIWIL2,FANCD2,MYBL1,CCNB2,EME1,BRIP1,DNMT3L,PTTG1,RAD54B,CKS2,SYCP2,ANKRD31
GO:BP	chromosome organization	GO:0051276	4.0346E-05	4.394198193618526	CENPE,MAPK15,MSH5-SAPCD1,CENPF,KIF14,PIF1,NUF2,CHMP4C,KIF18A,ZSCAN4,INO80B,HMGB2,KIF15,TOP2A,BRCA2,TTK,DLGAP5,POLQ,SMC4,ERN2,CENPC,HMGA2,TPR,CDK1,KIF18B,FANCM,KIF23,NUSAP1,PRAP1,HFM1,KIF11,EID3,CENPK,NDC80,NEK7,BUB1,HJURP,KNTC1,BUB1B,FANCD2,NEK2,ITGB3BP,SPICE1,SGOL1,BRIP1,NCAPG,CHTF18,SMC2,PTTG1,RAD54B,MNAT1,MAD2L1,ESCO1,MCM8,SYCP2,TEX14,KIF2C,SPC24,BLM,SMC5,RIF1,SPAG5,ANKRD31
GO:BP	regulation of cell cycle process	GO:0010564	4.0823E-05	4.389092683054017	AIF1,CENPE,MAPK15,CENPF,KIF14,NUF2,CHMP4C,MUC1,ATAD5,KIF15,BRCA2,TTK,MTBP,DLGAP5,SMC4,ECT2,KIF20B,HMGA2,TPR,RAD51AP1,CDK1,ANLN,KIF23,ETAA1,NUSAP1,PRAP1,KIF11,UFL1,WNT5A,EREG,BORA,MKI67,NDC80,L3MBTL1,CDC25C,FBXO43,PLK4,PIWIL2,SASS6,CCND2,CDCA2,OVOL1,BUB1,KIF20A,KNTC1,BUB1B,FANCD2,NEK2,BRCA1,EME1,SPICE1,BRIP1,PAGR1,CENPJ,PKN2,NCAPG,CDC14A,SMC2,MNAT1,RRM2B,MAD2L1,TEX14,CCNL2,KIF2C,SPC24,BLM,SMC5,SPAG5,ANKRD31
GO:BP	microtubule cytoskeleton organization	GO:0000226	6.2365E-05	4.205057328746428	CENPE,MAPK15,ASPM,NUF2,CEP152,GOLGA8B,CHMP4C,KIF18A,CCDC39,KIF15,BRCA2,TTK,DLGAP5,PHLDB2,DNAH2,FSIP2,TTLL6,CFAP74,XRCC2,TPR,CDK1,KIF18B,KIF23,CEP44,CCDC88B,NUSAP1,CFAP58,KIF11,BORA,CCDC78,TTC26,NDC80,NEK7,PLK4,SASS6,STARD9,AKAP9,KIF20A,MNS1,GJA1,CEP135,NEK2,CCNB2,BRCA1,SPICE1,SGOL1,CFAP69,ROCK1,GCC2,CFAP44,CENPJ,CDC14A,RTTN,CFAP43,CCDC66,MAD2L1,SKA1,SPAG17,CCNL2,KIF2C,CKAP2,TTLL3,NEDD1,SPAG5
GO:BP	regulation of chromosome segregation	GO:0051983	7.2965E-05	4.136883442644452	CENPE,MAPK15,CENPF,NUF2,TTK,DLGAP5,SMC4,TPR,CDK1,PRAP1,MKI67,NDC80,CDCA2,BUB1,KNTC1,BUB1B,NCAPG,SMC2,MAD2L1,TEX14,KIF2C,SPC24,SMC5
GO:BP	regulation of chromosome separation	GO:1905818	7.6033E-05	4.118996904320257	MAPK15,CENPF,NUF2,TTK,DLGAP5,SMC4,TPR,PRAP1,NDC80,BUB1,KNTC1,BUB1B,NCAPG,SMC2,MAD2L1,TEX14,SPC24
GO:BP	positive regulation of cell cycle process	GO:0090068	8.1718E-05	4.087684146702428	AIF1,MAPK15,KIF14,ATAD5,MTBP,DLGAP5,SMC4,ECT2,KIF20B,TPR,RAD51AP1,CDK1,KIF23,NUSAP1,PRAP1,WNT5A,EREG,NDC80,CDC25C,PLK4,PIWIL2,SASS6,CCND2,PAGR1,CENPJ,PKN2,NCAPG,CDC14A,SMC2,RRM2B,MAD2L1,SMC5,SPAG5,ANKRD31

GO:BP	mitotic cell cycle	GO:0000278	0.00011324	3.9459972060414072	AIF1,CENPE,CENPF,KIF14,NUF2,CHMP4C,KIF18A,MUC1,ATAD5,KIF15,BRCA2,TTK,MTBP,DLGAP5,SMC4,ECT2,KIF20B,CENPC,XRCC2,TPR,CDK1,ANLN,KIF18B,KIF23,NUSAP1,PRAP1,KIF11,CEP55,EREG,BORA,CENPK,MKI67,NDC80,L3MBTL1,CDC25C,PBK,FBXO43,NEK7,SASS6,CCND2,CDCA2,OVOL1,CCNA2,BUB1,KIF20A,IQGAP3,KNTC1,BUB1B,FANCD2,GJA1,MYBL1,NEK2,CCNB2,BRCA1,EME1,BTG3,SPICE1,SGOL1,ROCK1,WDHD1,CENPJ,PKN2,NCAPG,CDC14A,SMC2,CCNB3,MNAT1,RRM2B,MELK,MAD2L1,SKA1,CKS2,TEX14,KIF2C,SPC24,CKAP2,BLM,SMC5,NEDD1,SPAG5
GO:BP	mitotic cell cycle process	GO:1903047	0.00012558	3.9010958562069855	AIF1,CENPE,CENPF,KIF14,NUF2,CHMP4C,KIF18A,MUC1,ATAD5,KIF15,BRCA2,TTK,MTBP,DLGAP5,SMC4,ECT2,KIF20B,CENPC,TPR,CDK1,ANLN,KIF18B,KIF23,NUSAP1,PRAP1,KIF11,CEP55,EREG,BORA,CENPK,MKI67,NDC80,L3MBTL1,CDC25C,FBXO43,SASS6,CCND2,CDCA2,CCNA2,BUB1,KIF20A,IQGAP3,KNTC1,BUB1B,FANCD2,GJA1,NEK2,CCNB2,BRCA1,EME1,SPICE1,SGOL1,ROCK1,CENPJ,NCAPG,CDC14A,SMC2,CCNB3,MNAT1,RRM2B,MELK,MAD2L1,CKS2,TEX14,KIF2C,SPC24,CKAP2,BLM,SMC5,SPAG5
GO:BP	chromosome separation	GO:0051304	0.00031963	3.4953465295162904	MAPK15,CENPF,NUF2,TTK,DLGAP5,SMC4,TPR,PRAP1,NDC80,BUB1,KNTC1,BUB1B,NCAPG,SMC2,MAD2L1,TEX14,SPC24
GO:BP	meiosis I	GO:0007127	0.00032663	3.4859422608900887	MSH5-SAPCD1,DDX4,BRDT,TOP2A,BRCA2,CENPC,RAD51AP1,FANCM,HFM1,NDC80,PIWIL2,FANCD2,MYBL1,CCNB2,EME1,BRIP1,DNMT3L,PTTG1,RAD54B,CKS2,SYCP2,ANKRD31
GO:BP	cellular process involved in reproduction in multicellular organism	GO:0022412	0.00034105	3.467179713599752	ASPM,LIN28A,CFTR,PRSS37,KIF18A,DDX4,EQTN,HMGB2,BRDT,TOP2A,BRCA2,TTK,FSIP2,ANG,IQCF1,CFAP58,EREG,FUT6,DNHD1,DCST1,TTC26,DCST2,NDC80,ACRBP,PIWIL2,NODAL,ZMYND15,SPESP1,MNS1,MYBL1,CCNB2,SPEM1,DIAPH2,CFAP69,SPANXB1,BRIP1,CFAP44,CD9,DNMT3L,CFAP43,CATSPER4,IZUMO1,TMF1,PABPC1L,SYCP2,TEX14,ANGPT2,SPAG17
GO:BP	regulation of mitotic sister chromatid segregation	GO:0033047	0.00161602	2.791553255524511	CENPF,NUF2,TTK,TPR,CDK1,PRAP1,NDC80,BUB1,KNTC1,BUB1B,MAD2L1,TEX14,SPC24
GO:BP	spindle assembly checkpoint signaling	GO:0071173	0.00162292	2.7897026720629308	CENPF,NUF2,TTK,TPR,PRAP1,NDC80,BUB1,KNTC1,BUB1B,MAD2L1,TEX14,SPC24
GO:BP	mitotic spindle checkpoint signaling	GO:0071174	0.00162292	2.7897026720629308	CENPF,NUF2,TTK,TPR,PRAP1,NDC80,BUB1,KNTC1,BUB1B,MAD2L1,TEX14,SPC24
GO:BP	mitotic spindle assembly checkpoint signaling	GO:0007094	0.00162292	2.7897026720629308	CENPF,NUF2,TTK,TPR,PRAP1,NDC80,BUB1,KNTC1,BUB1B,MAD2L1,TEX14,SPC24
GO:BP	microtubule-based movement	GO:0007018	0.00163113	2.7875113823897752	CENPE,ATP1A4,KIF14,KIF18A,CCDC39,DDX4,DNAH12,SLC9C1,KIF15,MST1,DLGAP5,DNAH2,INTU,FSIP2,TTLL6,KIF20B,DNAH11,CFAP70,KIF18B,KIF23,IQCF1,CFAP58,KIF11,DNHD1,TTC26,RPGR,KIF12,STARD9,LCA5,KIF20A,OFD1,MNS1,SPEM1,CFAP69,CFAP44,IFT74,CFAP43,CATSPER4,TMF1,GAPDHS,SPAG17,KIF2C,TTLL3,RFX3
GO:BP	multicellular organism reproduction	GO:0032504	0.00170628	2.7679505313633572	ATP1A4,ASPM,LIN28A,CFTR,PRSS37,KIF18A,DDX4,MYCBPAP,EQTN,HMGB2,BRDT,SLC9C1,GALNT3,DMRTA1,TOP2A,BRCA2,TTK,MST1,PAX5,FSIP2,SPAG8,ANG,CGB7,DSG2,IQCF1,CFAP58,EREG,TDRP,FUT6,DNHD1,DCST1,TTC26,DCST2,BCL2L2-PABPN1,NDC80,ACRBP,DLEC1,CDC25C,PIWIL2,TDRD6,NODAL,ZMYND15,OVOL1,CATSPERG,SPESP1,MNS1,FANCD2,LEP,GJA1,EDNRB,MYBL1,CCNB2,CALR3,SPEM1,DIAPH2,CFAP69,SPANXB1,BRIP1,NHLH2,CFAP44,CD9,DNMT3L,RNF151,CFAP43,PTTG1,CATSPER4,IZUMO1,TSSK3,MCM8,TMF1,SPAG4,PABPC1L,SYCP2,TEX14,SKIL,ANGPT2,SPAG17,KLK14,THRB
GO:BP	negative regulation of nuclear division	GO:0051784	0.00181441	2.741264804661827	CENPF,NUF2,TTK,MTBP,TPR,PRAP1,NDC80,FBXO43,BUB1,KNTC1,BUB1B,MAD2L1,TEX14,SPC24

GO:BP	spindle checkpoint signaling	GO:0031577	0.0021019	2.6773871108705096	CENPF,NUF2,TTK,TPR,PRAP1,NDC80,BUB1,KNTC1,BUB1B,MAD2L1,TEX14,SPC24
GO:BP	negative regulation of mitotic nuclear division	GO:0045839	0.0025531	2.5929318679487814	CENPF,NUF2,TTK,MTBP,TPR,PRAP1,NDC80,BUB1,KNTC1,BUB1B,MAD2L1,TEX14,SPC24
GO:BP	negative regulation of mitotic metaphase/anaphase transition	GO:0045841	0.002702	2.568315166225254	CENPF,NUF2,TTK,TPR,PRAP1,NDC80,BUB1,KNTC1,BUB1B,MAD2L1,TEX14,SPC24
GO:BP	negative regulation of mitotic sister chromatid separation	GO:2000816	0.002702	2.568315166225254	CENPF,NUF2,TTK,TPR,PRAP1,NDC80,BUB1,KNTC1,BUB1B,MAD2L1,TEX14,SPC24
GO:BP	negative regulation of mitotic sister chromatid segregation	GO:0033048	0.002702	2.568315166225254	CENPF,NUF2,TTK,TPR,PRAP1,NDC80,BUB1,KNTC1,BUB1B,MAD2L1,TEX14,SPC24
GO:BP	negative regulation of sister chromatid segregation	GO:0033046	0.002702	2.568315166225254	CENPF,NUF2,TTK,TPR,PRAP1,NDC80,BUB1,KNTC1,BUB1B,MAD2L1,TEX14,SPC24
GO:BP	extracellular structure organization	GO:0043062	0.00303068	2.5184600137331623	COL4A6,TIE1,KLK4,COL4A5,COL7A1,FMOD,ADAMTS20,COL13A1,ABI3BP,PHLDB2,ADAMTS6,HPN,COL2A1,COL11A2,IMPG1,COL28A1,COL11A1,CMA1,TMPRSS6,PAPLN,COL9A2,WDR72,COL9A1,NPNT,COL27A1,LAMA1,ADAMTS4,EFEMP2,ADAMTS3,HMCN1,P4HA1,NPHS1,TEX14,COL9A3,CTSK,COL19A1
GO:BP	metaphase chromosome alignment	GO:0051310	0.00304738	2.516072935801432	CENPE,CENPF,KIF14,NUF2,CHMP4C,KIF18A,ECT2,CENPC,NDC80,CENPQ,NEK2,SPICE1,SGOL1,TEX14,KIF2C,SPC24,SPAG5
GO:BP	regulation of mitotic cell cycle	GO:0007346	0.00353499	2.4516116002002657	AIF1,CENPE,CENPF,KIF14,NUF2,CHMP4C,MUC1,ATAD5,BRCA2,TTK,MTBP,DLGAP5,KIF20B,TPR,CDK1,ANLN,NUSAP1,PRAP1,EREG,BORA,MKI67,NDC80,L3MBTL1,CDC25C,FBXO43,NEK7,SASS6,CCND2,CDCA2,OVOL1,BUB1,KNTC1,BUB1B,FANCD2,NEK2,BRCA1,EME1,BTG3,CENPJ,PKN2,CDC14A,MNAT1,RRM2B,MAD2L1,CKS2,TEX14,SPC24,BLM
GO:BP	negative regulation of chromosome segregation	GO:0051985	0.00437239	2.35928121	CENPF,NUF2,TTK,TPR,PRAP1,NDC80,BUB1,KNTC1,BUB1B,MAD2L1,TEX14,SPC24
GO:BP	negative regulation of chromosome separation	GO:1905819	0.00437239	2.35928121	CENPF,NUF2,TTK,TPR,PRAP1,NDC80,BUB1,KNTC1,BUB1B,MAD2L1,TEX14,SPC24
GO:BP	negative regulation of metaphase/anaphase transition of cell cycle	GO:1902100	0.00437239	2.35928121	CENPF,NUF2,TTK,TPR,PRAP1,NDC80,BUB1,KNTC1,BUB1B,MAD2L1,TEX14,SPC24
GO:BP	regulation of mitotic sister chromatid separation	GO:0010965	0.00486753	2.3126909948534538	CENPF,NUF2,TTK,DLGAP5,TPR,PRAP1,NDC80,BUB1,KNTC1,BUB1B,MAD2L1,TEX14,SPC24

GO:BP	gamete generation	GO:0007276	0.00582681	2.2345688675670825	ATP1A4,ASPM,LIN28A,CFTR,PRSS37,KIF18A,DDX4,MYCBPAP,HMGB2,BRDT,SLC9C1,GALNT3,TOP2A,BRCA2,TTK,MST1,PAX5,FSIP2,SPAG8,ANG,CGB7,IQCF1,CFAP58,EREG,TDRP,FUT6,DNHD1,TTC26,BCL2L2-PABPN1,NDC80,ACRBP,DLEC1,CDC25C,PIWIL2,TDRD6,NODAL,ZMYND15,OVOL1,CATSPERG,MNS1,FANCD2,LEP,GJA1,MYBL1,CCNB2,CALR3,SPEM1,DIAPH2,CFAP69,SPANXB1,BRIP1,CFAP44,DNMT3L,RNF151,CFAP43,PTTG1,CATSPER4,TSSK3,MCM8,TMF1,SPAG4,PABPC1L,SYCP2,TEX14,SKIL,ANGPT2,SPAG17
GO:BP	meiotic chromosome segregation	GO:0045132	0.0066414	2.177740574472448	MAPK15,MSH5-SAPCD1,ASPM,NUF2,TTK,SMC4,CENPC,NDC80,FANCD2,CCNB2,SGOL1,BRIP1,SMC2,PTTG1,SYCP2,ANKRD31
GO:BP	positive regulation of cell cycle	GO:0045787	0.00675259	2.1705295237732214	AIF1,MAPK15,KIF14,ATAD5,BRCA2,MTBP,DLGAP5,SMC4,ECT2,KIF20B,TPR,RAD51AP1,CDK1,KIF23,NUSAP1,PRAP1,WNT5A,EREG,NDC80,CDC25C,PLK4,PIWIL2,SASS6,CCND2,OVOL1,PAGR1,CENPJ,PKN2,NCAPG,CDC14A,SMC2,MNAT1,RRM2B,MAD2L1,SMC5,SPAG5,ANKRD31
GO:BP	extracellular matrix organization	GO:0030198	0.00746818	2.1267854679893605	COL4A6,TIE1,KLK4,COL4A5,COL7A1,FMOD,ADAMTS20,COL13A1,ABI3BP,PHLDB2,ADAMTS6,HPN,COL2A1,COL11A2,IMPG1,COL28A1,COL11A1,CMA1,TMPRSS6,PAPLN,COL9A2,WDR72,COL9A1,NPNT,COL27A1,LAMA1,ADAMTS4,EFEMP2,ADAMTS3,HMCN1,P4HA1,NPHS1,COL9A3,CTSK,COL19A1
GO:BP	meiotic cell cycle phase transition	GO:0044771	0.00856284	2.0673823383507366	MAPK15,TTK,NDC80,CDC25C,OVOL1,CCNB2
GO:BP	mitotic sister chromatid separation	GO:0051306	0.00888124	2.051526572634936	CENPF,NUF2,TTK,DLGAP5,TPR,PRAP1,NDC80,BUB1,KNTC1,BUB1B,MAD2L1,TEX14,SPC24
GO:BP	external encapsulating structure organization	GO:0045229	0.00920823	2.035823830672968	COL4A6,TIE1,KLK4,COL4A5,COL7A1,FMOD,ADAMTS20,COL13A1,ABI3BP,PHLDB2,ADAMTS6,HPN,COL2A1,COL11A2,IMPG1,COL28A1,COL11A1,CMA1,TMPRSS6,PAPLN,COL9A2,WDR72,COL9A1,NPNT,COL27A1,LAMA1,ADAMTS4,EFEMP2,ADAMTS3,HMCN1,P4HA1,NPHS1,COL9A3,CTSK,COL19A1
GO:BP	attachment of spindle microtubules to kinetochore	GO:0008608	0.00935707	2.0288600821784692	CENPE,NUF2,ECT2,CENPC,NDC80,NEK2,SGOL1,TEX14,KIF2C,SPC24,SPAG5
GO:BP	cytoskeleton organization	GO:0007010	0.011975671149615506	1.9217001381717025	AIF1,CENPE,MAPK15,BST2,POTEF,ASPM,NUF2,CEP152,GOLGA8B,CHMP4C,KIF18A,CCDC39,CORO6,PSTPIP1,MYO1A,KIF15,BRCA2,TTK,DLGAP5,PHLDB2,DNAH2,ECT2,FSIP2,TTLL6,ALOX15,CFAP74,NOSTRIN,XRCC2,PLEKHH2,TPR,ANG,UGT8,XIRP2,CDK1,ANLN,KIF18B,KIF23,ACTN3,CEP44,CCDC88B,NUSAP1,CFAP58,MYO3A,ARPC4-TTLL3,KRT12,KIF11,SH3D21,POF1B,BORA,NEB,CCDC78,TTC26,DIAPH3,FMN1,NDC80,NEK7,PLK4,CD2AP,PHACTR2,SASS6,STARD9,AKAP9,NCKAP1L,KIF20A,IQGAP3,TENM1,RHOH,MNS1,GJA1,CECR2,ARAP3,CEP135,NEK2,CCNB2,BRCA1,SPICE1,DIAPH2,SGOL1,FTCD,CFAP69,MYO15A,ROCK1,GCC2,CFAP44,KLHL24,F11R,CENPJ,CDC14A,RTTN,HMCN1,PRICKLE4,FHOD1,CFAP43,CCDC66,PLS1,NPHS1,MAD2L1,BRWD3,SKA1,SPAG17,CCNL2,KIF2C,CKAP2,TTLL3,NEDD1,SPAG5
GO:BP	cilium-dependent cell motility	GO:0060285	0.012331853714137359	1.9089716357067799	ATP1A4,CCDC39,DDX4,SLC9C1,MST1,DNAH2,FSIP2,TTLL6,DNAH11,IQCF1,CFAP58,DNHD1,MNS1,SPEM1,CFAP69,CFAP44,CFAP43,CATSPER4,TMF1,GAPDHS,TTLL3,RFX3
GO:BP	cilium or flagellum-dependent cell motility	GO:0001539	0.012331853714137359	1.9089716357067799	ATP1A4,CCDC39,DDX4,SLC9C1,MST1,DNAH2,FSIP2,TTLL6,DNAH11,IQCF1,CFAP58,DNHD1,MNS1,SPEM1,CFAP69,CFAP44,CFAP43,CATSPER4,TMF1,GAPDHS,TTLL3,RFX3
GO:BP	chromosome localization	GO:0050000	0.01336619	1.8739923562579077	CENPE,CENPF,KIF14,NUF2,CHMP4C,KIF18A,DLGAP5,ECT2,CENPC,NDC80,CENPQ,NEK2,SPICE1,SGOL1,TEX14,KIF2C,SPC24,SPAG5

GO:BP	organelle organization	GO:0006996	0.017290928391152496	1.7621816877943175	AIF1,CENPE,MAPK15,BST2,POTEF,MSH5-SAPCD1,CENPF,ASPM,GOLGA8A,GMNC,CEP290,KIF14,GOLGA6A,PIF1,NUF2,CEP152,GOLGA8B,CHMP4C,KIF18A,ZSCAN4,CCDC39,GOLGA6C,INO80B,DDX4,TSPO2,GOLGA6B,PMEL,CORO6,HMGB2,PSTPIP1,SEC31B,MYO1A,BRDT,KIF15,TOP2A,BRCA2,TTK,MTBP,DLGAP5,PHLDB2,POLQ,SMC4,DNAH2,INTU,ECT2,FSIP2,TTLL6,ALOX15,CFAP74,NOSTRIN,ERN2,KIF20B,ATG16L2,CENPC,XRCC2,HMGA2,PLEKHH2,TPR,ANG,UGT8,XIRP2,CFAP70,WDR11,RAD51AP1,CDK1,TNFSF10,ANLN,KIF18B,FANCM,KIF23,ACTN3,RUFY4,CEP44,TBC1D32,CCDC88B,NUSAP1,PRAP1,CFAP58,HFM1,CEP162,MYO3A,ARPC4-TTLL3,KRT12,KIF11,UFL1,SH3D21,LRRTM2,WNT5A,POF1B,EREG,ARHGAP11B,EID3,BORA,NEB,DNHD1,CCDC78,CENPK,TTC26,TCIRG1,DIAPH3,RPGR,SAR1B,MKI67,STX19,C11ORF65,FMN1,NDC80,L3MBTL1,ACRBP,PEX3,CDC25C,FBXO43,NEK7,PLK4,CD2AP,PIWIL2,PHACTR2,SASS6,STARD9,AKAP9,CDCA2,NCKAP1L,SDHAF3,LCA5,BUB1,IQUB,KIF20A,ABCA1,OFD1,MTFR2,HJURP,ATP2A1,IQGAP3,KNTC1,BUB1B,TENM1,RHOH,GOLGA8T,MNS1,FANCD2,MTX3,GJA1,CECR2,MYBL1,ARAP3,MPHOSPH9,CEP135,NEK2,CCNB2,BRCA1,ITGB3BP,EME1,SPICE1,DIAPH2,SGOL1,FTCD,CFAP69,SYCP2L,BRIP1,MYO15A,ROCK1,GCC2,CFAP44,KLHL24,F11R,CEP83,GOLGB1,CENPJ,NCAPG,CDC14A,RTTN,HMCN1,EIF2D,PRICKLE4,AGK,IFT74,FHOD1,DNMT3L,CHTF18,SMC2,CFAP43,PTTG1,RAD54B,PMAIP1,CCDC66,MNAT1,PLS1,RRM2B,NPHS1,GOLGA8N,MAD2L1,BRWD3,ESCO1,SLC4A7,WDR90,SKA1,MCM8,TMF1,CKS2,STX16,SYCP2,TEX14,OMA1,SPAG17,CCNL2,KIF2C,SPC24,CKAP2,GOLGA8R,TTLL3,OGT,BLM,SMC5,NEDD1,CTSK,RFX3,RIF1,GOLGA8O,SPAG5,ANKRD31
GO:BP	establishment of chromosome localization	GO:0051303	0.019372047361220057	1.7128244778512167	CENPE,CENPF,KIF14,NUF2,CHMP4C,KIF18A,ECT2,CENPC,NDC80,CENPQ,NEK2,SPICE1,SGOL1,TEX14,KIF2C,SPC24,SPAG5
GO:BP	multicellular organismal reproductive process	GO:0048609	0.03778653	1.422663000613828	ATP1A4,ASPM,LIN28A,CFTR,PRSS37,KIF18A,DDX4,MYCBPAP,HMGB2,BRDT,SLC9C1,GALNT3,TOP2A,BRCA2,TTK,MST1,PAX5,FSIP2,SPAG8,ANG,CGB7,DSG2,IQCF1,CFAP58,EREG,TDRP,FUT6,DNHD1,TTC26,BCL2L2-PABPN1,NDC80,ACRBP,DLEC1,CDC25C,PIWIL2,TDRD6,NODAL,ZMYND15,OVOL1,CATSPERG,MNS1,FANCD2,LEP,GJA1,EDNRB,MYBL1,CCNB2,CALR3,SPEM1,DIAPH2,CFAP69,SPANXB1,BRIP1,NHLH2,CFAP44,DNMT3L,RNF151,CFAP43,PTTG1,CATSPER4,TSSK3,MCM8,TMF1,SPAG4,PABPC1L,SYCP2,TEX14,SKIL,ANGPT2,SPAG17,KLK14
GO:BP	cilium movement	GO:0003341	0.04665057	1.3311430060733227	ATP1A4,CCDC39,DDX4,SLC9C1,MST1,FSIP2,TTLL6,DNAH11,CFAP70,IQCF1,CFAP58,DNHD1,OFD1,MNS1,SPEM1,CFAP69,CFAP44,CFAP43,CATSPER4,TMF1,GAPDHS,SPAG17,TTLL3,RFX3
GO:CC	condensed chromosome	GO:0000793	4.6168454403276594e-8	7.335654664226503	CENPE,CENPF,NUF2,CHMP4C,KIF18A,HMGB2,TOP2A,BRCA2,TTK,MTBP,SMC4,CENPC,TPR,EID3,CENPK,MKI67,NDC80,L3MBTL1,BUB1,HJURP,KNTC1,BUB1B,FANCD2,NEK2,BRCA1,ITGB3BP,SGOL1,SYCP2L,NCAPG,DNMT3L,SMC2,MAD2L1,SKA1,SYCP2,TEX14,KIF2C,SPC24,BLM,SMC5,RIF1,SPAG5
GO:CC	collagen trimer	GO:0005581	1.244002765914707e-7	6.90517865	COL4A6,C1QTNF9B,COL4A5,COL7A1,COL13A1,C1QTNF8,COL2A1,COL11A2,COL28A1,COL11A1,C1QTNF9,FCN3,COL20A1,COL9A2,COL9A1,COL27A1,SFTPA2,P4HA1,COL21A1,COL9A3,COL25A1,COL19A1
GO:CC	microtubule organizing center	GO:0005815	4.525525001619064e-7	6.344331031468656	TRIM43,BIRC7,MAPK15,CENPF,ASPM,AGBL2,CEP290,FAM166A,CEP152,PLAG1,KIF18A,CCDC14,KIF15,TOP2A,BRCA2,CCDC18,DLGAP5,HMMR,CKAP2L,INTU,ECT2,TTLL6,KIF20B,CNTRL,XRCC2,CFAP70,WDR11,CDK1,KIF23,CEP44,CCDC88B,CFAP58,CEP162,CEP55,CCDC78,TTC26,RPGR,C4ORF47,NDC80,NEIL1,LRRCC1,NEK7,PLK4,CD2AP,SASS6,STARD9,RBBP6,AKAP9,LCA5,OFD1,BUB1B,MPHOSPH9,CEP135,NEK2,CCNB2,BRCA1,SPICE1,SGOL1,FTCD,ROCK1,TMEM63A,CEP83,CENPJ,PKN2,CDC14A,RTTN,CCDC15,IFT74,CAPRIN2,CCNB3,CCDC66,BOD1L1,WDR90,SKA1,RAPGEF6,TEK,KIF2C,CKAP2,DCDC2B,PPP1R32,NEDD1,SPAG5
GO:CC	collagen-containing extracellular matrix	GO:0062023	1.8958E-05	4.722200439231296	COL4A6,COL4A5,COL7A1,FMOD,ADAMTS20,MMRN1,VWA2,COL13A1,ABI3BP,ANGPTL3,SPN,ANG,FREM1,COL2A1,COL11A2,IMPG1,RBP3,COL28A1,COL11A1,FBLN2,WNT5A,FCN3,COL20A1,CMA1,COL9A2,COL9A1,NPNT,EFEMP1,COL27A1,LAMA1,ADAMTS4,EFEMP2,IL7,ANGPTL5,ADAMTS3,VCAN,HMCN1,ITIH4,GPC6,PCOLCE,SBSPON,COL21A1,COL9A3,ANGPT2,HAPLN3,COL25A1,COL19A1
GO:CC	chromosome, centromeric region	GO:0000775	3.1038E-05	4.50810544	CENPE,CENPF,MIS18BP1,NUF2,CHMP4C,KIF18A,TOP2A,TTK,MTBP,SMC4,CENPC,TPR,SNAI1,CENPK,NDC80,IKZF1,BUB1,HJURP,KNTC1,BUB1B,CENPQ,NEK2,ITGB3BP,SGOL1,SYCP2L,NCAPG,MAD2L1,SKA1,TEX14,KIF2C,SPC24,SMC5,SPAG5,BAZ1A

GO:CC	centriole	GO:0005814	3.8005E-05	4.420161267404739	MAPK15,AGBL2,CEP290,CEP152,TOP2A,INTU,CEP44,CEP162,CEP55,CCDC78,LRRCC1,PLK4,SASS6,STARD9,OFD1,MPHOSPH9,CEP135,SPICE1,FTCD,ROCK1,CEP83,CENPJ,RTTN,WDR90,NEDD1
GO:CC	microtubule cytoskeleton	GO:0015630	0.00010304	3.987008958485771	TRIM43,BIRC7,CENPE,MAPK15,CENPF,ASPM,AGBL2,CEP290,FAM166A,KIF14,CEP152,PLAG1,CHMP4C,KIF18A,NPHP3-ACAD11,CCDC14,DNAH12,KIF15,TOP2A,BRCA2,TTK,CCDC18,DLGAP5,HMMR,KPNA7,CKAP2L,DNAH2,INTU,ECT2,TTLL6,KIF20B,SPAG8,CNTRL,XRCC2,DNAH11,TPR,CFAP70,WDR11,CDK1,KIF18B,KIF23,CEP44,PTPN7,CCDC88B,NUSAP1,CFAP58,CEP162,KIF11,CEP55,BORA,DNHD1,CCDC78,TTC26,RPGR,C4ORF47,NDC80,NEIL1,LRRCC1,NEK7,PLK4,CD2AP,KIF12,SASS6,STARD9,RBBP6,AKAP9,LCA5,KIF20A,OFD1,KNTC1,BUB1B,MNS1,MPHOSPH9,CEP135,NEK2,CCNB2,BRCA1,SPICE1,SGOL1,FTCD,ROCK1,TMEM63A,CEP83,CENPJ,PKN2,CDC14A,RTTN,CCDC15,IFT74,CAPRIN2,CCNB3,CCDC66,BOD1L1,MAD2L1,WDR90,SKA1,RAPGEF6,TEK,HYPK,SPAG17,KIF2C,CKAP2,TTLL3,DCDC2B,PPP1R32,NEDD1,RIF1,SPAG5
GO:CC	condensed chromosome, centromeric region	GO:0000779	0.0001401	3.8535719370308223	CENPE,CENPF,NUF2,CHMP4C,KIF18A,TTK,MTBP,CENPC,TPR,CENPK,NDC80,BUB1,HJURP,KNTC1,BUB1B,NEK2,ITGB3BP,SGOL1,SYCP2L,NCAPG,MAD2L1,SKA1,TEX14,KIF2C,SPC24,SPAG5
GO:CC	extracellular matrix	GO:0031012	0.00019211	3.716446612955899	COL4A6,COL4A5,COL7A1,FMOD,ADAMTS20,MMRN1,VWA2,COL13A1,CHAD,ZAN,ABI3BP,ANGPTL3,ADAMTS6,SPN,ANG,FREM1,COL2A1,COL11A2,IMPG1,RBP3,COL28A1,COL11A1,LRIG3,FBLN2,WNT5A,FCN3,COL20A1,CMA1,COL9A2,MUC4,MUC5B,COL9A1,NPNT,EFEMP1,COL27A1,LAMA1,ADAMTS4,EFEMP2,IL7,ANGPTL5,ADAMTS3,VCAN,HMCN1,ITIH4,GPC6,PCOLCE,SBSPON,COL21A1,COL9A3,ANGPT2,HAPLN3,COL25A1,MUC6,COL19A1
GO:CC	external encapsulating structure	GO:0030312	0.00020333	3.6918047086166883	COL4A6,COL4A5,COL7A1,FMOD,ADAMTS20,MMRN1,VWA2,COL13A1,CHAD,ZAN,ABI3BP,ANGPTL3,ADAMTS6,SPN,ANG,FREM1,COL2A1,COL11A2,IMPG1,RBP3,COL28A1,COL11A1,LRIG3,FBLN2,WNT5A,FCN3,COL20A1,CMA1,COL9A2,MUC4,MUC5B,COL9A1,NPNT,EFEMP1,COL27A1,LAMA1,ADAMTS4,EFEMP2,IL7,ANGPTL5,ADAMTS3,VCAN,HMCN1,ITIH4,GPC6,PCOLCE,SBSPON,COL21A1,COL9A3,ANGPT2,HAPLN3,COL25A1,MUC6,COL19A1
GO:CC	kinetochore	GO:0000776	0.00052838	3.277052936181424	CENPE,CENPF,NUF2,CHMP4C,KIF18A,TTK,MTBP,CENPC,TPR,CENPK,NDC80,BUB1,HJURP,KNTC1,BUB1B,NEK2,ITGB3BP,SGOL1,MAD2L1,SKA1,TEX14,KIF2C,SPC24,SPAG5
GO:CC	chromosomal region	GO:0098687	0.00053494	3.2716983581303976	CENPE,CENPF,MIS18BP1,PIF1,NUF2,CHMP4C,KIF18A,ZSCAN4,TOP2A,BRCA2,TTK,MTBP,SMC4,CENPC,TPR,RAD51AP1,CDK1,SNAI1,EID3,CENPK,NDC80,IKZF1,BUB1,HJURP,KNTC1,BUB1B,CENPQ,NEK2,ITGB3BP,SGOL1,SYCP2L,NCAPG,MAD2L1,SKA1,TEX14,KIF2C,SPC24,BLM,SMC5,RIF1,SPAG5,BAZ1A
GO:CC	centrosome	GO:0005813	0.00055121	3.258679309759524	BIRC7,CENPF,ASPM,CEP290,CEP152,PLAG1,CCDC14,KIF15,BRCA2,CCDC18,DLGAP5,HMMR,CKAP2L,KIF20B,CNTRL,XRCC2,CDK1,KIF23,CEP44,CCDC88B,CFAP58,CEP162,CEP55,TTC26,RPGR,C4ORF47,NDC80,LRRCC1,PLK4,CD2AP,SASS6,RBBP6,AKAP9,OFD1,MPHOSPH9,CEP135,NEK2,CCNB2,SPICE1,SGOL1,TMEM63A,CEP83,CENPJ,PKN2,CDC14A,RTTN,CCDC15,IFT74,CAPRIN2,CCNB3,CCDC66,WDR90,SKA1,RAPGEF6,TEK,KIF2C,CKAP2,NEDD1,SPAG5
GO:CC	complex of collagen trimers	GO:0098644	0.00121589	2.9151056044515613	COL4A6,COL4A5,COL7A1,COL2A1,COL11A2,COL28A1,COL11A1,COL27A1
GO:CC	Golgi cis cisterna	GO:0000137	0.00149365	2.8257521680498536	GOLGA8A,GOLGA6A,GOLGA8B,GOLGA6C,GOLGA6B,GOLGA8T,GOLGA8N,GOLGA8R,GOLGA8O
GO:CC	spindle	GO:0005819	0.00375454	2.425443304193521	CENPE,MAPK15,CENPF,ASPM,KIF14,CHMP4C,KIF18A,KIF15,TTK,DLGAP5,HMMR,KPNA7,CKAP2L,ECT2,KIF20B,SPAG8,CNTRL,TPR,CDK1,KIF18B,KIF23,CEP44,PTPN7,NUSAP1,CEP162,KIF11,BORA,NEK7,KIF20A,KNTC1,BUB1B,NEK2,SPICE1,SGOL1,CDC14A,MAD2L1,SKA1,KIF2C,CKAP2,NEDD1,RIF1,SPAG5
GO:CC	cilium	GO:0005929	0.00611651	2.213496322433687	ATP1A4,MAPK15,CENPF,SCNN1A,AGBL2,HAVCR1,CEP290,FAM166A,CACNA1F,CCDC39,STRC,DNAH12,SLC9C1,DNAH2,INTU,FSIP2,TTLL6,CFAP74,SPAG8,CNTRL,DNAH11,CFAP70,ARR3,CNGA4,WDR11,PDE6B,TBC1D32,IMPG1,CFAP58,CEP162,DNHD1,TTC26,RPGR,C4ORF47,DLEC1,TAS2R43,CATSPERG,LCA5,IQUB,OFD1,MNS1,TSPEAR,PDC,CFAP69,VCAN,CFAP44,CEP83,CENPJ,CDC14A,RTTN,IFT74,CFAP43,CCDC66,CATSPER4,MXRA8,PROM2,SPAG4,SPAG17,TTLL3,PPP1R32,NEDD1,SPAG5
GO:CC	Golgi cisterna membrane	GO:0032580	0.00978603	2.009393657174243	GOLGA8A,GOLGA6A,GOLGA8B,GOLGA6C,GOLGA6B,GALNT3,GOLIM4,FUT6,SAR1B,GOLGA8T,FUT3,GAL3ST4,GOLGA8N,GOLGA8R,GOLGA8O
GO:CC	deuterosome	GO:0098536	0.010009526877010663	1.9995864499492095	CEP152,CCDC78,PLK4,SASS6

GO:CC	cis-Golgi network	GO:0005801	0.01073175 7800080101	1.969329137 2743875	GOLGA8A,GOLGA6A,GOLGA8B,GOLGA6C,GOLGA6B,PMEL,GOLIM4,AKAP9,GOLGA8T,GOLGB1,GOLGA8N,GOLGA8R,GOLGA8O
GO:CC	cytoskeleton	GO:0005856	0.01296226 1163936912	1.887319232 6194785	TRIM43,BIRC7,AIF1,CENPE,MAPK15,MPZL2,CENPF,ASPM,AGBL2,LMNTD2,CEP290,FAM166A,KIF14,UACA,CEP152,PLAG1,CHMP4C,KIF18A,MYO15B,CCDC39,NPHP3-ACAD11,DDX60,CCDC14,PSTPIP1,MYO1A,DNAH12,KIF15,TOP2A,BRCA2,TTK,CCDC18,DLGAP5,PHLDB2,HMMR,KPNA7,CKAP2L,DNAH2,INTU,ECT2,TTLL6,CFAP74,NOSTRIN,KIF20B,SPAG8,CNTRL,XRCC2,PLEKHH2,DNAH11,TPR,ANG,XIRP2,CFAP70,MYL7,WDR11,CDK1,ANLN,KIF18B,KIF23,INPP5D,KRTAP19-1,ACTN3,CEP44,UTRN,PTPN7,CCDC88B,NUSAP1,KLHL14,CFAP58,CEP162,JAK3,MYO3A,ARPC4-TTLL3,RHBG,KRT12,KIF11,PTPN13,POF1B,CEP55,MLPH,BORA,NEB,DNHD1,CCDC78,TTC26,DIAPH3,RPGR,C4ORF47,FMN1,NDC80,NEIL1,LRRCC1,NEK7,PLK4,CD2AP,KIF12,SASS6,STARD9,RBBP6,AKAP9,LCA5,IQUB,KIF20A,OFD1,MVP,KNTC1,BUB1B,TENM1,MNS1,PNN,CENPQ,ARAP3,MPHOSPH9,CEP135,SLC4A1,NEK2,CCNB2,BRCA1,SPICE1,DIAPH2,SGOL1,FTCD,MYO15A,ROCK1,CFAP44,TMEM63A,CEP83,CENPJ,PKN2,CDC14A,RTTN,PRICKLE4,CCDC15,IFT74,FHOD1,CAPRIN2,CFAP43,CCNB3,CCDC66,PLS1,BOD1L1,MAD2L1,WDR90,SKA1,RAPGEF6,SPAG4,TEK,HYPK,SPAG17,KIF2C,CKAP2,TTLL3,DCDC2B,PPP1R32,NEDD1,RIF1,SPAG5
GO:CC	acrosomal vesicle	GO:0001669	0.01814227	1.741308303 6676706	SCNN1A,PRSS37,EQTN,SPAG8,CAPN11,IQCF1,SPATA1,DCST1,DCST2,ACRBP,IQUB,SPESP1,STK31,IFT74,CD46,CATSPER4,IZUMO1,SKIL,SPAG17
GO:CC	procentriole replication complex	GO:0120099	0.02889267 4444033973	1.53921226	CEP152,PLK4,SASS6,CENPJ
GO:CC	kinesin complex	GO:0005871	0.02904667 7280907388	1.536903540 4199503	KIF14,KIF18A,NPHP3-ACAD11,KIF15,KIF20B,KIF18B,KIF23,KIF11,KIF20A,KIF2C
GO:CC	Golgi cisterna	GO:0031985	0.04935551	1.306664370 3120153	GOLGA8A,GOLGA6A,GOLGA8B,GOLGA6C,GOLGA6B,GALNT3,GOLIM4,FUT6,SAR1B,GOLGA8T,FUT3,GAL3ST4,GOLGA8N,STX16,GOLGA8R,GOLGA8O

Table S5: List of Upregulated genes in Suspended and Embedded Samples at DIS 60 following differential gene expression analysis

**Upregulated Genes in Embedded Samples (360)**						
geneID	***logFC***	***AveExpr***	***t***	***P.Value***	***adj.P.Val***	***B***
COL3A1	4.787903626	6.550678743	3.90604463	0.000940173	0.005106791	-0.961788155
COL1A1	4.656603827	7.226692999	3.996888425	0.000763535	0.004424355	-0.829912004
GAL	4.638057789	0.204245819	4.801353646	0.000122065	0.001230407	1.079070451
UTS2	4.563737295	-2.107963134	6.619344653	2.39376492731144e-06	0.000100674	2.930784319
SIX1	4.559947187	-0.603999955	3.80156563	0.001194276	0.006075143	-0.87745103
CXCL8	4.292257981	-1.632069034	4.632998446	0.000178677	0.00158288	0.379014211
DYSF	4.072705514	1.280174394	4.483715637	0.000250875	0.002000528	0.605123962
COL8A1	4.023862427	-0.505130748	5.018780555	7.48633150552905e-05	0.00086863	1.366869965
CMKLR1	3.903262827	-0.45140948	3.574370833	0.002007005	0.008900692	-1.264999252
PRRG2	3.773931983	0.851789191	11.34369628	6.18630546982757e-10	.65204569766988e-0	10.71322969
RGS10	3.737918578	0.125344994	4.005066071	0.000749363	0.00436141	-0.405260529
TNFAIP6	3.718673209	-2.549085849	4.511050781	0.000235738	0.00191048	-0.051420313
ACAN	3.675800161	-1.878408492	4.872126879	0.000104063	0.001096258	0.745565015
KCNE4	3.547252129	0.135358866	5.77908876	1.40455453049154e-05	0.000293524	2.906709089
MSC	3.415840957	-0.130976593	4.757743536	0.000134701	0.00131512	1.009838215
HR	3.400208787	1.787174547	6.179509749	5.97632977510686e-06	0.000175232	4.004323178
OPRD1	3.385789777	-1.881041767	5.875503683	1.14128963795223e-05	0.00025794	2.287866035
KRT80	3.338955647	-2.03199977	6.034149064	8.13107792835627e-06	0.00021249	2.437005691
FOLR3	3.274625544	-2.060231185	3.98671066	0.000781547	0.004493709	-0.723235374
PLA2G3	3.272747184	-0.819370725	3.974854117	0.000803066	0.004564285	-0.537042136
NPR3	3.252204583	3.218499795	4.263553521	0.000414659	0.002834796	0.080262388
COL5A3	3.140941421	4.41596922	4.057243708	0.000664939	0.004015354	-0.482754179
ICAM1	3.124539475	2.037372331	4.630284327	0.000179781	0.001586647	0.923317289
TGFBI	3.080752091	1.940202274	4.217486037	0.000460735	0.003061568	0.056451829
HTR6	3.057119979	0.250756403	6.684957936	2.09294203195757e-06	.38516759032394e-0	4.52395222
MMP1	3.002678239	-2.336395362	4.672307643	0.000163439	0.001494754	0.328876067
MSC-AS1	3.000954269	-0.058244376	4.991602988	7.95635190844215e-05	0.000901046	1.478575817
TBX3	2.986318912	0.517004541	3.735436874	0.001389347	0.006787698	-0.913222639
KCNV1	2.946389787	0.499896676	3.876751136	0.001005416	0.005376442	-0.630857813
PSMB8	2.9424003	0.425961812	6.168345938	6.11874617841848e-06	0.000177133	3.719486809
TMEM171	2.882540539	-3.055840158	3.848589262	0.001072391	0.005629127	-1.153631864
GATA4	2.84003838	-3.422334375	4.501694023	0.000240813	0.00194213	-0.30622691
BHLHA15	2.788059867	-1.305460739	5.333818201	3.71469990551964e-05	0.000548556	1.765969802
PAX1	2.758444518	-0.307486238	4.756396908	0.000135112	0.001317721	1.009363524
SERPINE1	2.712570734	2.347012039	5.978026432	9.1639699515577e-06	0.000228999	3.68685068
SYNPO	2.70997527	3.177220416	7.570601952	3.61528127855707e-07	.93097280448821e-0	6.749184693
HSPB7	2.678615844	0.314745824	4.140446503	0.000549577	0.003488447	-0.109421005
BCYRN1	2.616044375	7.481166899	6.91815094	1.30466404239104e-06	.55341092069203e-0	5.45373951
IL32	2.614221088	-0.648118483	3.941027527	0.000867772	0.004818115	-0.563886496
MMP19	2.612372925	-0.682753982	5.480978579	2.68695922617465e-05	0.000441114	2.230790588
C2CD4A	2.554971636	-2.786462653	5.302225528	3.98337713035235e-05	0.000574078	1.114181499
RELB	2.515977019	3.240325547	7.739173338	2.61933404697523e-07	3.2549234718433e-05	7.058824219
TNFRSF9	2.502812133	-1.825683626	4.744849941	0.000138686	0.001340592	0.642577235
SH3TC1	2.483159498	-1.915165293	3.725050018	0.001422741	0.006909133	-1.109881624
KL	2.475599319	-2.63023036	4.550858574	0.000215327	0.001790749	0.076766536
CARTPT	2.450730344	0.638685074	6.447068201	3.41510227855899e-06	0.0001237	4.298139419
F2RL2	2.412536426	0.968025271	3.80295285	0.00119049	0.006062638	-0.781951958
ARHGDIB	2.408199869	-1.042936781	3.609463236	0.001852638	0.008399636	-1.212780231
PSMB9	2.390952718	-2.185260465	3.81543161	0.001156967	0.005950586	-0.996774995
NTSR1	2.36310679	3.622091887	5.84115837	1.22871411458466e-05	0.000267822	3.416889114
ADRA1B	2.360028756	1.726924835	7.288511317	6.25248963123689e-07	.96583600407844e-0	6.040403024
SLC24A2	2.357457783	2.96261143	4.152436885	0.000534695	0.003417523	-0.157063137
CPNE6	2.349966172	-0.357322919	3.725415196	0.001421554	0.00690653	-0.950728514
SP100	2.342717663	-1.348386563	3.758406572	0.001318239	0.00652263	-0.969690145
OOSP1	2.32957805	-2.895584005	4.735283584	0.000141719	0.001361802	0.269532225
PTH2R	2.309237294	-1.181273285	3.906936626	0.000938254	0.0050994	-0.678633579
CES5A	2.275931392	-1.794301798	5.889425326	1.10769552426376e-05	0.00025484	2.471712453

EGR2	2.222381883	1.986521734	4.430791754	0.000283032	0.002189812	0.499253135
SPHKAP	2.21905445	4.128093486	6.57121248	2.64256229221956e-06	0.000104258	4.885000658
OSMR	2.204513173	0.05579352	3.704734899	0.001490377	0.007156867	-0.977082897
PDYN	2.201831946	0.849514131	3.554664344	0.0020992	0.009202325	-1.2869714
HGF	2.195779951	-0.731958502	3.698806749	0.001510709	0.007222226	-1.020661108
HTR2B	2.187262348	-1.879017026	3.725397884	0.00142161	0.00690653	-1.094922602
LOC10013366	2.182404619	-2.151134671	4.231535151	0.000446163	0.002995242	-0.28381338
ACOT4	2.180695287	-1.275622305	4.301866438	0.000379887	0.002680746	0.027257409
VGF	2.172333364	7.974371737	6.689187237	2.07494364153878e-06	.38516759032394e-0	4.983354949
OR2W3	2.159865295	-1.076568006	5.241523415	4.55667269515194e-05	0.000627255	1.729560516
SERPINA3	2.156911096	-0.211933134	4.054284351	0.000669462	0.004030674	-0.302772131
PNPLA5	2.154492824	-2.994373593	3.838165769	0.001098292	0.005730507	-1.120861881
TICAM1	2.15099212	0.743509502	7.905453381	1.91318976314276e-07	.79585899226631e-0	6.730978156
MGLL	2.149608975	4.513722645	7.825459659	2.2242994593007e-07	.01117265661626e-0	7.278272095
RCVRN	2.14791493	-1.908875891	4.676540341	0.000161879	0.001485357	0.523700152
SLC47A1	2.146487316	0.100626062	5.754572652	1.48093939574771e-05	0.000301587	2.953400683
ARHGAP36	2.144206698	0.486954658	3.790451144	0.001225044	0.006195183	-0.80243042
KCNA1	2.139479253	2.659929603	4.427690207	0.000285041	0.002198297	0.45923159
ADAMTS14	2.135827451	0.46839595	3.838763001	0.001096791	0.005725947	-0.705147356
TG	2.126949247	0.362266893	7.966800561	1.70539813803075e-07	.64004303283117e-0	6.647292289
PLXND1	2.122947267	5.2014206	3.917557782	0.000915703	0.005010344	-0.930785032
CCKBR	2.114053698	2.716373762	7.930032951	1.82694723134074e-07	.71323943698384e-0	7.334229899
GDA	2.112228245	0.776332548	4.806926881	0.000120539	0.001220556	1.257164839
CORO2A	2.101994953	2.822423138	6.888609585	1.38460364189536e-06	.68770660380019e-0	5.478496375
SCN5A	2.094243665	1.2274032	3.651470776	0.001683245	0.00782587	-1.106125337
A2M	2.088661349	2.195593115	3.536748859	0.002186643	0.00949451	-1.416366283
SPR	2.077816032	0.036045207	5.642891765	1.88667026250071e-05	0.000352391	2.741676193
S100A10	2.054740996	4.132327102	3.956998987	0.000836596	0.004702185	-0.71464303
11-Mar	2.053335817	1.039403564	4.293390881	0.000387318	0.002710779	0.225238662
GBGT1	2.051100997	-1.092166123	4.653693722	0.000170484	0.001536371	0.699403506
PTRF	2.050646834	4.348564428	3.659488732	0.001652704	0.007737041	-1.398323392
CEMIP	2.041348734	2.881303164	5.497215561	2.59300626215632e-05	0.000432176	2.721567548
TIMP4	2.030747092	0.637269561	5.135048946	5.77381500495228e-05	0.000737039	1.892338628
TTC39A	2.017449908	1.64907739	3.802024113	0.001193023	0.006072153	-0.816540563
GPR83	1.99172712	1.813135149	4.004357101	0.000750581	0.00436652	-0.398688639
HIST1H3J	1.968897779	-1.528033338	3.672738121	0.001603434	0.007550897	-1.137472968
DAAM2	1.963432853	2.918037339	4.195270364	0.000484761	0.003178439	-0.065334945
SFRP1	1.962334155	6.333112695	3.597962045	0.001901889	0.008561313	-1.75340997
PDE2A	1.952725597	5.048519605	11.33547794	6.26191238896713e-10	.65204569766988e-0	12.93325221
DYNC1I1	1.92673749	5.21028441	7.478288591	4.32001089960039e-07	4.1533494264737e-05	6.614308451
USH1G	1.923301977	-0.270623932	4.68607569	0.000158419	0.001461536	0.912635827
DLGAP1-AS5	1.919801139	-2.401320378	4.028689415	0.000709887	0.004189825	-0.669377095
PPARG	1.909340574	-0.717839673	4.278577358	0.000400657	0.00276913	0.080791988
TRIM58	1.908304247	2.29348429	5.334500295	3.70910774059598e-05	0.000548174	2.388550981
DLC1	1.898051303	2.838734062	7.236735066	6.92192788655529e-07	.20340974089323e-0	6.127097404
WIPF3	1.896707817	4.468578314	7.802621512	2.32243108329864e-07	.03027489990116e-0	7.236587761
EMILIN3	1.896468963	3.186758466	4.079305676	0.000632172	0.003876432	-0.344067566
ITGB3	1.893514708	1.278405604	5.515583429	2.49076504788865e-05	0.000419344	2.707808176
LOC344887	1.879348761	-1.410437299	3.771477826	0.001279401	0.006397708	-0.944246762
P3H2	1.875854522	2.099532339	4.688282912	0.000157629	0.00145719	1.039353318
TRPC5	1.870233208	1.719026909	4.752593011	0.000136279	0.001326269	1.179530104
TNFAIP8L3	1.859810362	-0.086414458	3.601513237	0.001886545	0.008513222	-1.181023851
SLC2A6	1.858853957	4.229557892	8.27249111	9.68782131813752e-08	.91731949043276e-0	8.075085886
ADAMTSL3	1.848823915	-0.270999263	3.709565424	0.001474011	0.007102548	-0.973956033
SYNDIG1L	1.847613399	1.369405436	4.203831197	0.000475357	0.003130262	0.036841523
LOC284578	1.845601686	0.132609747	3.588701018	0.001942488	0.008676992	-1.205249034
LY6E	1.842720819	5.090391826	7.4026083	5.00338455103225e-07	.41953518348406e-0	6.473344305
SPRY4	1.842633174	4.653320897	6.629376138	2.34502915514293e-06	0.000100117	4.97567696
PSD4	1.826351411	1.045744217	7.174159916	7.83113298371538e-07	.53135291921259e-0	5.696895352
MMP11	1.822615835	2.483799989	6.417674617	3.62997975021258e-06	0.000128506	4.561367628
KNCN	1.819443695	-1.665161004	3.960310869	0.000830273	0.00467816	-0.640688821
DHCR24	1.81566865	7.099537327	6.602353299	2.47871136216774e-06	0.00010123	4.811566289
ESRP1	1.789783184	-1.080228036	5.383460858	3.32935748861433e-05	0.000507234	1.988648542

ADRA2C	1.789359061	2.941107921	6.318871454	4.46008755752502e-06	0.000145456	4.388262384
RIN3	1.788107314	1.591557382	4.603307487	0.000191134	0.001645369	0.868551098
LHFPL5	1.779853572	-0.862776146	3.909376041	0.000933026	0.005075517	-0.628566939
SLC6A17	1.775853519	6.310347232	5.940421851	9.93065839371985e-06	0.000240588	3.444168704
CHI3L1	1.774974621	-2.36570537	3.731712429	0.001401231	0.006838441	-1.153108045
IGSF21	1.771221461	4.623337695	5.487926161	2.64633896005734e-05	0.000436906	2.595095956
LINC01574	1.755722271	-2.430971935	3.520583235	0.002268627	0.009748533	-1.51637575
ETV4	1.750294417	3.84381648	6.271391621	4.92629184167097e-06	0.000154703	4.286941922
IL34	1.746394774	1.93147795	4.641724224	0.000175175	0.001559489	0.944494522
ZNF860	1.744383902	0.270558369	3.607919715	0.001859173	0.008425086	-1.167893906
GAS7	1.741248481	6.711046781	10.77548723	1.45585171359114e-09	.41764029565175e-0	12.19103008
LINC01018	1.740768529	-0.652241858	5.115217521	6.0348326207837e-05	0.000757651	1.641339971
GPX3	1.73254258	3.47578996	4.830162711	0.000114383	0.001168589	1.267373685
CHRM4	1.722750654	2.399713954	5.838950557	1.23456386414086e-05	0.000268474	3.418226792
TPBGL	1.720429323	2.212204427	5.174058978	5.29343796528972e-05	0.000696652	2.056217298
GABRA5	1.717050172	3.950059678	5.617799832	1.99255106066947e-05	0.000362168	2.924763729
PHYHIP	1.711167575	5.04376984	6.08089507	7.36242255463627e-06	0.00020073	3.819594289
FAM163B	1.710190399	2.880906122	4.032476514	0.000703755	0.004170504	-0.418962556
SRXN1	1.709866929	5.07564853	7.281494962	6.33913388087474e-07	.96982654943944e-0	6.239018066
CCL2	1.705472711	1.450252255	4.639703717	0.00017598	0.001563532	0.944336789
HOXC13	1.701971664	-1.834507979	3.697078128	0.00151669	0.007239449	-1.128156674
ATP10A	1.692746895	-0.052471655	4.312022676	0.000371172	0.002635136	0.219502039
OSGIN1	1.692276291	2.833714678	4.276098028	0.000402935	0.002780662	0.114607866
SHROOM1	1.688695149	0.676253329	3.635238873	0.001746799	0.00803545	-1.12081644
PHF24	1.687752076	6.190736762	9.931450128	5.52712335565014e-09	.30882913325477e-0	10.88415641
LOC285484	1.684561299	0.176344868	4.79586495	0.000123587	0.00124178	1.187574835
LYPD6B	1.681742952	0.274304633	6.326247296	4.39184941406924e-06	0.000144291	4.03524035
FAM131C	1.679114065	3.27639022	5.36497351	3.46784925159945e-05	0.000520715	2.428717317
KCNK12	1.665274024	2.919901392	5.543890144	2.34121087234078e-05	0.00040119	2.816819859
ARSJ	1.653193534	-0.151982966	3.53712138	0.002184788	0.009493228	-1.307430965
KCNJ12	1.65150012	3.354451228	4.127843548	0.000565667	0.003560659	-0.258628724
LOXL2	1.642230512	4.559305967	3.527062153	0.002235411	0.009646406	-1.733681351
L3MBTL4	1.633283012	0.577099607	4.834257642	0.000113332	0.001163706	1.303479892
CBR3	1.631940331	-0.056917101	3.880250135	0.000997391	0.005339784	-0.630542296
SPHK1	1.621728172	2.668631323	5.550780477	2.3062183251062e-05	0.000396683	2.834514638
CDHR1	1.614784444	3.350643355	3.918382907	0.000913974	0.005003165	-0.718344502
HTATIP2	1.608691498	1.06983399	4.644937542	0.000173903	0.001556435	0.949083489
ATP2A3	1.607337617	1.0072567	5.527535189	2.42646191805142e-05	0.000411552	2.708999735
PLEKHF1	1.600034231	1.192987189	4.167176552	0.000516955	0.003335645	-0.037044887
SCUBE1	1.598152459	4.485160607	5.269806449	4.27975451787856e-05	0.000601834	2.130719041
TMEM54	1.587518109	1.672642664	5.909238748	1.06162554055521e-05	0.000250229	3.517987677
ME1	1.585626017	2.584607423	3.794734074	0.001213095	0.006154369	-0.906750126
LRRC61	1.578474213	0.897357924	3.656636603	0.001663504	0.007769684	-1.085397424
NQO1	1.573615933	4.660390533	5.110117923	6.10387556500116e-05	0.000763695	1.764033507
DACT2	1.567173691	0.964658019	4.165028476	0.000519503	0.003347358	-0.039223311
TRIM14	1.566742214	1.374249317	6.011688774	8.52927665410414e-06	0.000218826	3.684971389
PARVB	1.564693223	1.79689045	3.98134729	0.000791209	0.004531674	-0.450005488
EVC2	1.558165381	0.324961639	3.654508703	0.001671608	0.007789608	-1.075530553
CAMKV	1.54078207	6.964029739	6.266383758	4.97830554010715e-06	0.000155451	4.114122101
CHST8	1.540545569	4.501153465	4.061917595	0.000657858	0.003981809	-0.546055151
RSAD2	1.537909064	-1.012019666	4.878839955	0.000102502	0.001081245	1.138314318
TMEM132E	1.537149068	5.067448439	5.01441788	7.55983320543034e-05	0.000871536	1.509873303
TLE2	1.533839675	3.70194663	6.105835625	6.98328708851956e-06	0.000193866	3.953115583
ETV5	1.531111381	5.560035647	7.268424115	6.50387442544272e-07	.01292295905326e-0	6.188017436
SPRED3	1.523975247	5.518217718	7.945912785	1.7733833891827e-07	.70452143403145e-0	7.476268457
C1orf216	1.520590127	5.894873262	7.906375588	1.90987890875934e-07	.79585899226631e-0	7.391272484
TMEM179	1.519762271	4.69906628	3.522865357	0.002256872	0.009710258	-1.768139325
LOC101926941	1.519229298	-0.213603212	6.179675302	5.97424366733238e-06	0.000175232	3.643388231
P4HA3	1.512520628	-0.968050221	3.551943152	0.002112256	0.009252995	-1.304936025
MARVELD1	1.508330523	4.237243634	4.096247313	0.000608113	0.003773232	-0.436444522
SEMA3A	1.500086703	4.005589296	3.770821079	0.001281325	0.006403822	-1.126863913
KIF26A	1.494006878	5.720587605	4.106899189	0.000593458	0.00369485	-0.585906271
THBS1	1.488278772	4.500386641	3.5829831	0.001967981	0.008761664	-1.608854868

NFAM1	1.484571749	0.414435269	6.985000257	1.14089035217842e-06	.98875333179754e-0	5.203981476
RNF112	1.480641741	3.85298932	5.026635056	7.35582826175801e-05	0.000858587	1.658579884
HRH2	1.477102914	0.790601116	6.150799015	6.34968678096642e-06	0.000181518	3.855114662
RCAN1	1.467461233	6.267405089	8.658096246	4.82990263161755e-08	1.2974681084082e-05	8.746164512
CARD10	1.466222116	2.816976847	5.958921494	9.54557656456666e-06	0.000234479	3.66797118
SHE	1.462581785	-0.895073487	3.933951083	0.000881954	0.004874631	-0.580875895
ADM2	1.462239439	2.963212436	5.25399063	4.4324202293007e-05	0.000614784	2.207106368
ADAM11	1.458347269	5.341200029	4.03660934	0.000697124	0.004146316	-0.708570582
DCHS2	1.456882478	3.010488497	4.734262193	0.000142047	0.001362086	1.092701873
ADRA2A	1.453188182	4.358990197	6.330333992	4.35450485697583e-06	0.000143847	4.379429098
EGR4	1.450600646	-0.256961209	3.676397278	0.001590086	0.007506864	-1.036208133
MPPED2	1.445637145	4.633136636	3.794534331	0.00121365	0.006154844	-1.161790847
SPTB	1.439653169	4.726559885	4.774024649	0.000129835	0.001282287	1.010147611
MMP28	1.439547384	0.722447843	3.990805908	0.000774249	0.004464393	-0.396409185
HCRTR1	1.433859376	0.948582217	3.645194258	0.001707544	0.007900633	-1.112098163
CDK18	1.426303882	2.731233455	5.478852677	2.69951570295088e-05	0.000442042	2.684746935
BEGAIN	1.415389476	5.329947963	7.283854577	6.3098573644096e-07	.96819674464096e-0	6.226836049
B3GNT2	1.41503019	3.501518937	4.128456499	0.000564873	0.003559288	-0.277299751
TEAD4	1.404637547	0.82013402	3.58178511	0.001973364	0.008779211	-1.236575267
OPCML	1.401126446	6.17946762	4.650338408	0.000171786	0.001544296	0.605437648
STAC	1.400235089	3.160282439	3.823076886	0.001136895	0.005871546	-0.90679936
ANGPTL1	1.398098774	0.798016843	3.764773228	0.001299178	0.006455953	-0.861280616
INF2	1.396590209	4.967973935	8.463943071	6.8407513488339e-08	.63517157382665e-0	8.423919849
TFCP2L1	1.395711166	-1.402098019	3.532394426	0.002208434	0.009557323	-1.370957146
FABP3	1.393294436	4.478151877	4.030056417	0.000707668	0.00418213	-0.619322662
SVOPL	1.392824877	-1.42021249	3.853430729	0.001060568	0.005589555	-0.788974584
NXPH3	1.391130279	3.789068965	3.560176675	0.002072997	0.009124685	-1.561302245
LZTS1	1.388753393	5.58549225	4.340438924	0.000347843	0.0025209	-0.052121985
ASTN2	1.380791269	5.111092266	10.39170473	2.64477775973087e-09	.84236918250117e-0	11.58177588
THY1	1.378258021	6.593931076	7.495409503	4.17929302187749e-07	.10308567566517e-0	6.594637393
NR4A3	1.356423181	4.394730947	3.590774239	0.001933325	0.008651903	-1.581898303
FAM83G	1.354788052	1.718104678	3.7730663	0.00127476	0.006379737	-0.887617142
ULBP2	1.352192848	0.906930887	5.668339344	1.78516663573219e-05	0.000340038	2.9738559
PRR19	1.349399366	2.151357549	7.423569283	4.80358597510809e-07	.32251748804431e-0	6.382688442
DOK5	1.346027859	5.19600687	6.717610312	1.95805708126338e-06	.10122867771963e-0	5.111490699
AVPR1A	1.342771437	-0.151253751	4.385473039	0.000313857	0.002351055	0.359800283
DHCR7	1.341200456	6.359495123	6.177481236	6.00195159522659e-06	0.000175701	3.938611693
LRFN2	1.335997414	3.410454625	6.502096338	3.04736735322535e-06	0.000113837	4.756282531
OAF	1.330424098	2.760903733	3.566205958	0.002044707	0.009034995	-1.418016903
PCYT2	1.329947721	5.508196961	10.29389672	3.08719785689415e-09	.96809701325713e-0	11.44730562
KCNE1	1.328373423	-1.20769459	3.876230389	0.001006615	0.005379708	-0.720563311
PTPRN	1.327415893	7.320297027	5.053106263	6.93281320544206e-05	0.00082935	1.477484044
FHL2	1.318337768	2.016810786	4.928475181	9.16742370075956e-05	0.000996794	1.544874359
CLEC2L	1.316439105	2.613362237	5.706268428	1.64415381279238e-05	0.000324087	3.153680503
PDLIM4	1.316293431	3.385638186	4.249064816	0.000428628	0.002904069	-0.000228676
HMOX1	1.311477477	4.471481383	4.649452574	0.000172131	0.00154588	0.759593367
FRMPD1	1.304438219	1.841619186	3.650156846	0.001688303	0.007835985	-1.155874121
FAR2	1.298101877	3.443607857	6.893627204	1.37067714632968e-06	.67338976514648e-0	5.520295582
SEZ6	1.296422131	7.270171746	5.421614268	3.06113477065854e-05	0.000479569	2.293097574
EPHX1	1.293417996	5.847638973	8.268577055	9.75746933589683e-08	.91731949043276e-0	8.055976847
SCN2B	1.290724281	3.843149847	5.002741536	7.76018874738128e-05	0.000887492	1.604900274
MYD88	1.285289731	2.453164534	4.942348399	8.88611358059194e-05	0.000973741	1.565163102
PPAP2C	1.284336392	1.886399262	3.950217668	0.000849694	0.004748046	-0.522978599
FABP6	1.282240817	0.0688529	3.878195972	0.001002094	0.00536025	-0.62929063
HS3ST1	1.280356975	2.879893279	5.045679398	7.04893355253876e-05	0.00083558	1.767887086
ATP2B3	1.279532725	4.931448343	7.565802721	3.64880582323247e-07	.94394887414128e-0	6.782731733
ST3GAL1	1.273481016	5.028571637	3.769347711	0.001285652	0.006414081	-1.276519034
TMTC1	1.272031656	4.23816093	5.696013376	1.68111493991764e-05	0.000328789	3.062312215
MICAL2	1.267093428	3.310480714	5.895854694	1.09252523219011e-05	0.000253703	3.534056678
IPCEF1	1.266405091	1.080148812	4.642715743	0.000174781	0.001558952	0.946365509
SNX7	1.262604791	3.744350282	3.691077908	0.001537631	0.007310751	-1.271513705
IL12RB2	1.262211301	-0.796642942	3.703211716	0.001495575	0.007174817	-1.00734131
EXTL1	1.258847262	2.659280109	5.423078317	3.05129393433469e-05	0.000479237	2.569730777

SH3RF3	1.256453724	5.30325983	6.979679553	1.15311057367095e-06	7.02129028308243e-0	5.628298849
LRRK1	1.254085366	1.313285376	4.036642504	0.000697071	0.004146316	-0.313902669
ZDHHC8P1	1.253038504	3.627267268	6.459453061	3.32853132244026e-06	0.000122093	4.669183095
OSBPL10	1.252080496	2.899136196	6.216682968	5.5262160428653e-06	0.000166032	4.185965401
EGFL7	1.244383532	3.561900983	6.100698431	7.05970435566945e-06	0.000195098	3.946222418
IRAK1	1.244231546	4.65460083	4.650477825	0.000171731	0.001544296	0.737781596
FNDC9	1.241407724	0.302974347	3.524782016	0.002247046	0.009686406	-1.336108101
GALNT9	1.239995073	4.599589618	4.343417197	0.000345485	0.002509333	0.059156004
LDLR	1.238537679	5.345240625	4.304771681	0.000377374	0.002667113	-0.114299178
UAP1L1	1.236757631	4.334198879	4.116014572	0.000581198	0.003636713	-0.413299736
DNAJA4	1.236105154	3.045546826	5.735083648	1.54469302949995e-05	0.000310076	3.209295925
ICOSLG	1.234720534	-0.903697019	3.698385313	0.001512165	0.007225404	-1.02362522
PITPNM2	1.23265989	5.186171006	4.555430706	0.0002131	0.001780152	0.464540986
SH3RF3-AS1	1.232464707	2.056965782	4.790940685	0.000124968	0.001252219	1.255280347
BDNF	1.231110552	3.242946835	4.846620036	0.000110217	0.001142643	1.317051703
AHNAK2	1.230511108	6.589491657	5.494871716	2.60635825061144e-05	0.000432821	2.464617686
KCNA6	1.222458361	6.245249484	5.168213746	5.36273653624003e-05	0.000701727	1.75607197
NLRC5	1.215772461	0.869319144	3.710360586	0.001471334	0.007093392	-0.976192437
IGFBP3	1.212248625	4.445239098	5.456804674	2.83333452069774e-05	0.000455202	2.530491625
KLF2	1.206531339	1.280453602	3.939060796	0.000871691	0.004832527	-0.517163926
PNPLA3	1.204544992	3.843927814	4.749617964	0.000137199	0.001333089	1.04962538
C10orf54	1.204067673	0.173961364	3.756326633	0.001324526	0.006544527	-0.871196553
SNORD44	1.202645186	-1.742783145	3.683526556	0.001564394	0.007407151	-1.13266465
LKAAEAR1	1.202182859	0.464271786	4.599199703	0.000192926	0.001656095	0.832070899
PEX5L	1.201318674	3.86009682	3.828367225	0.001123209	0.005823916	-0.987184926
ATP8A2	1.191019044	5.555728786	6.073560185	7.47790442358458e-06	0.00020297	3.75396114
TMEM151A	1.188088156	5.387730791	4.83809062	0.000112357	0.001158251	1.075702627
DUSP5	1.188034099	3.811064891	5.603200377	2.05693864550327e-05	0.000366577	2.89726201
SLC4A3	1.186722401	6.492371711	5.44455436	2.9106000041619e-05	0.000464755	2.356498799
NPM2	1.180335719	3.069730729	4.835948481	0.000112901	0.001161891	1.306448698
PACSIN3	1.177662322	1.981880052	4.056559415	0.000665982	0.004017668	-0.302739669
FASN	1.176368792	9.342188706	6.743333595	1.85813282100218e-06	8.9087958638443e-05	5.093433691
FAM189A2	1.176285264	1.659813016	3.509576123	0.00232618	0.009902247	-1.438548863
FLJ23867	1.173272261	2.719481624	8.023830174	1.53321036116609e-07	.43540466673226e-0	7.510090266
SSTR2	1.172087875	5.503639431	4.034490493	0.000700516	0.004157349	-0.739309803
FAM149A	1.171155074	4.191530052	8.765703965	3.99072643969959e-08	.22666735911394e-0	8.929805905
KCNQ5	1.170168068	1.774364462	3.650132517	0.001688397	0.007835985	-1.152099694
HYAL3	1.168196553	2.494068254	4.997470052	7.85240502065779e-05	0.000892011	1.680258723
CHRNA4	1.167004223	5.816340036	4.988125371	8.0186246798723e-05	0.000905292	1.376446589
PALM3	1.166959222	4.257842317	4.392780766	0.000308666	0.002327037	0.211514906
ANXA11	1.165299834	3.259668856	3.656190272	0.001665201	0.007771558	-1.28453644
GJC2	1.16025296	2.23182947	6.617022222	2.40519617846366e-06	0.000100674	4.927576207
DMTN	1.15721908	6.389210328	6.245542619	5.20091601479757e-06	0.000160706	4.078747946
SLC7A2	1.155847303	1.429982545	3.560259055	0.002072608	0.009124685	-1.316789115
TP53I11	1.153114947	7.233132216	8.938901182	2.94421853096829e-08	.07749918139914e-0	9.228401939
ATP1A3	1.15109952	9.612431912	4.831711016	0.000113985	0.001166474	0.981519891
ADAP1	1.147148119	5.839174609	5.436497241	2.96258955859396e-05	0.000469771	2.364889463
INSIG1	1.146732792	6.161255267	4.38554349	0.000313806	0.002351055	0.005315053
SNORD55	1.134223957	0.744925041	4.962841696	3.48653593602199e-05	0.00094182	1.57918814
G6PD	1.124638725	5.724854286	10.45037444	2.41163150131442e-09	.84236918250117e-0	11.69257309
SHISA8	1.123741191	1.140256813	4.76893669	0.000131337	0.001292632	1.207182634
INPP5J	1.121249734	3.825843183	4.83929958	0.000112051	0.001155751	1.247109214
NANS	1.121203475	4.967560059	5.565330923	2.23407206741214e-05	0.000387197	2.712177035
PIWIL4	1.120367712	-0.881879767	4.152587378	0.000534511	0.003417523	-0.163890902
ACAT2	1.119434761	6.094094108	6.126442393	6.68520463759197e-06	0.000187988	3.836988456
CHRNB2	1.117267408	6.019521878	8.749873397	4.10398616214075e-08	.22897565940697e-0	8.908944973
MVD	1.106723518	7.015723667	5.619562812	1.98491678082237e-05	0.000362036	2.727496894
ANKRD34A	1.105406581	3.999357146	5.033371313	7.24575025412535e-05	0.000850201	1.653555386
CCDC86	1.101524598	4.559995055	6.228174009	5.39425264695148e-06	0.000164228	4.149999556
THEM6	1.101518867	4.15324516	9.507899023	1.11249629665892e-08	5.51504604169131e-0	10.1430201
TNFAIP2	1.100115009	1.508328023	4.507373004	0.000237719	0.001924832	0.667949747
SQSTM1	1.099768971	8.297199039	5.586867992	2.13149941321489e-05	0.000375469	2.651386457
FAM212A	1.096806894	2.482026919	5.923817205	1.02898767353357e-05	0.000245065	3.59234205

SYT16	1.09566495	3.145722215	3.785407166	0.001239267	0.006243155	-0.990427282
STYK1	1.094607399	1.677701119	3.839786257	0.001094225	0.00571745	-0.745879143
PC	1.08936248	5.175327492	9.521698826	1.08706952049328e-08	.51504604169131e-0	10.21890469
IL10RA	1.089297438	2.0700197	6.054737675	7.78284791634448e-06	0.000207546	3.834546941
ADCK2	1.088756986	4.959187003	7.223930711	7.098648588965e-07	.29269443978056e-0	6.123670253
EEF1A2	1.08640534	8.329852578	5.308777123	3.92607388057118e-05	0.000568736	2.041396369
KIAA0319	1.084495406	4.319604905	7.263665634	6.56494357957974e-07	.03873211631021e-0	6.226126025
PTDSS1	1.080159966	6.458619541	10.52860486	2.13363992288041e-09	.78394289080403e-0	11.81760189
TUBB4A	1.075790716	8.083121375	4.332370056	0.000354313	0.002545276	-0.145582134
DOC2B	1.069484842	5.423532578	5.320049539	3.82944260556464e-05	0.000559619	2.136777265
TGFB1	1.068538336	3.226641031	3.54214362	0.00215994	0.009412983	-1.528293155
MKX	1.068499928	0.553941538	4.295818994	0.000385174	0.002699915	0.224890372
PLCB2	1.067310358	2.077591958	6.06347224	7.63978040074253e-06	0.000204627	3.852223673
MAFF	1.066225158	4.097421273	5.773676169	1.42106355907667e-05	0.000295372	3.235047349
KHDRBS3	1.065573346	5.324317646	5.3075866	3.93642380924978e-05	0.000569169	2.117528932
FNTB	1.065547026	1.134043356	4.389739112	0.000310816	0.002336494	0.425180967
MYLK4	1.064474649	0.332491875	3.685615162	0.001556946	0.007379536	-1.013961995
DAPK2	1.06095052	0.816838308	4.907951361	9.6002872041396e-05	0.001029158	1.474991514
F12	1.060148529	2.765817738	4.719292377	0.000146941	0.001389322	1.073974822
GAREML	1.057575612	5.78565518	6.515348533	2.96507027244143e-06	0.000111676	4.660430849
NUDT14	1.055934293	4.419926447	7.215026197	7.22429259554673e-07	.35613094958005e-0	6.129626506
KCNK3	1.055132462	5.886500511	4.319726684	0.000364696	0.002602305	-0.129923576
KIRREL3	1.054876498	5.48864296	5.068115847	6.70404514405724e-05	0.000811012	1.575493101
SLC45A1	1.047101052	4.461343849	5.129966861	5.83958792739444e-05	0.000741291	1.814175642
GRIN1	1.042199915	5.996940458	5.150077522	5.5836966328699e-05	0.000723386	1.723930867
TMEM229B	1.031998083	5.703514974	6.502822311	3.04279860962645e-06	0.000113837	4.63821952
TMEM120A	1.031601462	5.13552872	5.304697308	3.96165815225939e-05	0.000571624	2.127120873
SPRN	1.029347244	5.370207486	5.085372155	6.45051121742354e-05	0.000791346	1.622976376
ATP6V0A1	1.02871816	8.212350398	7.078008081	9.47610350623744e-07	.24909685012416e-0	5.766014245
OLFM2	1.026533937	7.824076446	7.177734289	7.77601063639775e-07	.53135291921259e-0	5.963849439
GAMT	1.026066669	5.212191208	8.776189897	3.91749917192192e-08	.22666735911394e-0	8.966375551
TKT	1.024787635	7.593714458	9.505447195	1.11707835737986e-08	.51504604169131e-0	10.18771204
PITPNM1	1.023909076	7.293415424	4.567163528	0.000207491	0.001748089	0.386641473
PEMT	1.023565953	5.214287641	7.277043733	6.3947451446656e-07	.99200040844472e-0	6.213398497
PLEKHG3	1.023298414	3.759465829	3.529097557	0.002225075	0.009613399	-1.631176624
SLCO4A1	1.022161579	2.979957101	4.292144037	0.000388423	0.002714355	0.131770394
TANC2	1.021568232	7.051416266	5.208357539	4.9048082489479e-05	0.000661714	1.823602815
CSRNP1	1.019580321	4.596661817	6.613259412	2.42383681090898e-06	0.000100678	4.93334792
SUSD5	1.018856522	2.620285702	3.690120537	0.001540999	0.007319143	-1.139787518
CYP1A1	1.018707569	1.073316348	4.192189977	0.00048819	0.00319748	0.015405611
OPLAH	1.018392115	1.276299929	4.831206461	0.000114114	0.001167148	1.338692338
SHB	1.015477158	4.635531137	4.15766248	0.000528337	0.003394215	-0.367296986
EMID1	1.014767608	4.417877325	4.328582404	0.000357392	0.002563203	0.04335928
RASGEF1C	1.014631218	4.033028052	5.198036546	5.01859147311082e-05	0.000673583	2.00732023
CABLES1	1.007942818	2.927328083	4.039342991	0.000692772	0.004130178	-0.414265315
SLC7A5	1.006523681	8.019438036	8.771332415	3.95124547077331e-08	.22666735911394e-0	8.935213779
TAGLN2	1.006243709	5.176850553	3.58506447	0.001958663	0.008732951	-1.712458098
FBXO41	1.004863851	7.002517475	4.685258749	0.000158713	0.001462766	0.65548235
MAP1A	1.004322548	8.727471307	4.71557899	0.000148181	0.001399139	0.718946214
SREBF2	1.002699761	8.831055364	6.039204689	3.04411169463475e-06	0.000212037	3.626248385
RIMBP3	1.000791552	0.687673891	3.964957425	0.000821482	0.004643812	-0.449664692

Upregulated Genes in Suspended Samples (921)

geneID	*logFC*	*AveExpr*	*t*	*P.Value*	*adj.P.Val*	*B*
KDM4E	-4.767071875	-3.409203218	-5.783539261	1.39112742995137e-05	0.000292089	1.47780142
TRIM49B	-4.581913937	-2.799986804	-4.170891634	0.000512577	0.003320551	-0.676546162
FAM90A27P	-4.553337212	-2.279874823	-4.381993538	0.000316359	0.002362605	-0.215563678
TRIM49	-4.34899393	-2.902698755	-4.267957031	0.000410505	0.002814828	-0.546373946
OTX2-AS1	-4.330458937	-1.584093808	-4.112769324	0.000585533	0.003656453	-0.456652609
LOC343052	-4.230848695	-2.796059481	-4.139186448	0.000551165	0.003495876	-0.713420366
MUC19	-3.763144047	0.251531808	-7.767853123	2.48053215772908e-07	.16321866490016e-0	6.105686433
LINC01021	-3.744551771	1.453849285	-6.273234603	4.90729099328963e-06	0.000154703	4.115009303
OR51E2	-3.615951646	-1.941153663	-5.626259448	1.95619014794779e-05	0.000359856	1.899467227

PRAME	-3.605554207	-2.440760544	-5.261833761	4.35603003593524e-05	0.000608811	1.138429297
LOC101927501	-3.385785667	-3.714127435	-3.765928067	0.00129575	0.006447688	-1.385236588
PRAMEF12	-3.355906943	-2.491881899	-4.082972192	0.000626886	0.003855464	-0.657388108
C1orf168	-3.323226276	0.009251633	-4.042500153	0.00068778	0.004112495	-0.326837737
LOC101928834	-3.219292324	-1.436553089	-5.418436375	3.08260727864757e-05	0.000481429	1.839055484
EDNRB-AS1	-3.203694419	0.079448964	-5.882952527	1.12318683014598e-05	0.000256033	3.124081828
MMRN1	-2.984334647	2.117854769	-5.135402113	5.76927243068593e-05	0.000736974	1.975113907
ETNPPL	-2.962799154	0.476847805	-3.79066668	0.00122444	0.006194665	-0.802543404
LHX5-AS1	-2.935913757	2.38323751	-5.385147223	3.31700930295572e-05	0.000505775	2.492673382
PI15	-2.910024851	5.989278886	-5.050428943	6.97444739035003e-05	0.000831479	1.548159211
LRRC74B	-2.869962176	-1.309269575	-7.497001473	4.16645213260423e-07	.10308567566517e-0	4.927759476
ROBO3	-2.839782168	6.124623452	-8.937912089	2.94930525373389e-08	.07749918139914e-0	9.24213684
LINC00989	-2.826459663	-0.997260881	-5.537892337	2.37211299580589e-05	0.000404211	2.226175668
ERBB4	-2.806411522	5.14492934	-5.622053383	1.97418281841467e-05	0.000360985	2.864996152
GOLGA6B	-2.794503588	-1.297564553	-4.725237758	0.000144977	0.001382201	0.754666616
PTF1A	-2.792592074	0.969211738	-5.108980488	6.11938464796362e-05	0.000765112	1.863936071
LINC00092	-2.78920281	-1.791739206	-8.085216519	1.36790126211848e-07	.33527592103909e-0	5.246617065
MEG3	-2.782388745	-2.408469474	-3.656084513	0.001665603	0.007771558	-1.309431377
COL6A4P2	-2.714984011	0.592224567	-7.758102585	2.52684539557507e-07	3.1833024028255e-05	6.406230849
GOLGA6A	-2.679947826	-2.26346024	-4.99000898	7.984834078524e-05	0.000902593	0.889911329
ZNF80	-2.661337493	-2.966166113	-4.764457853	0.000132673	0.001301574	0.277691355
PKHD1L1	-2.659845843	0.291223147	-4.085570692	0.000623166	0.003840544	-0.21636511
DNAH12	-2.618059151	0.194449527	-7.107554392	8.9356665922678e-07	.02316685021978e-0	5.244994719
LINC01135	-2.611531433	-2.406187405	-4.547235261	0.000217109	0.001801054	0.151037394
C12orf79	-2.579767444	0.823990428	-4.743229098	0.000139195	0.001342491	1.129346839
COL6A5	-2.548128091	-1.245148487	-4.496024006	0.000243941	0.001962165	0.37698615
GSX1	-2.547768912	0.055345421	-3.57327882	0.002012007	0.008920712	-1.235995398
PTPRVP	-2.510429468	-2.353389382	-3.720772256	0.001436725	0.006952229	-1.183298105
LOC730668	-2.509520718	-2.777514926	-4.743710245	0.000139044	0.001341739	0.330731369
PAX3	-2.466131768	4.702535878	-6.282477664	4.81312830181644e-06	0.000152907	4.274973486
ATP1A4	-2.443990018	-0.619221203	-6.704512211	2.01105319830403e-06	.18397719335495e-0	4.279391517
LOC100507661	-2.423027421	-0.950753577	-8.075455124	1.39289727794079e-07	.33885450590616e-0	5.970301002
WDR49	-2.419090255	1.552363223	-5.751376826	1.49120584961204e-05	0.000302665	3.190697867
GOLGA2P6	-2.40878039	-1.667718228	-6.244205282	5.21554574175128e-06	0.000160706	3.063580063
LCNL1	-2.406449203	2.215111279	-6.609257196	2.44382707720207e-06	0.000100771	4.900237096
EDNRB	-2.395603314	7.291065709	-5.154163186	5.5331228513298e-05	0.000718362	1.708620872
CCDC168	-2.372056378	-2.5935458	-4.071930815	0.000642942	0.003918166	-0.653035313
GLYCTK-AS1	-2.353786801	1.989837308	-4.046825031	0.000680999	0.004085326	-0.317195663
LOC399715	-2.350577187	-0.111835811	-5.056278952	6.88380313275483e-05	0.00082565	1.630749723
PRDM13	-2.339977181	2.98706506	-4.900830513	9.75527856095275e-05	0.00103846	1.457427058
LINC00894	-2.338792617	2.083564415	-6.4642087	3.29589301542153e-06	0.000121139	4.614188422
SERPINI2	-2.336852849	-0.22491002	-6.390208352	3.84336519644647e-06	0.000132967	3.954907563
FLJ16779	-2.32403376	4.345678562	-5.687074099	1.71402820381615e-05	0.000331324	3.05081792
TKTL1	-2.320686666	3.684758404	-4.85229043	0.000108818	0.001132635	1.303571823
LOC101409256	-2.312556008	-1.998597688	-4.210442736	0.00046822	0.003101151	-0.279419989
THEMIS	-2.299584343	-2.121740126	-3.671671007	0.001607348	0.007563478	-1.221945183
EN2	-2.290273007	4.86749353	-7.008444474	1.08861893132178e-06	.85717310981205e-0	5.721697816
ALOX15	-2.287456606	-0.585758818	-3.946656082	0.000856655	0.004772345	-0.536596173
LINC01573	-2.262954541	0.078202615	-6.726649572	1.92232589002085e-06	.03393200860714e-0	4.622980192
LINC00893	-2.24840039	2.5161718	-7.001633725	1.1035439860255e-06	.88001296680127e-0	5.662643784
ANKRD33	-2.244919216	-2.760289527	-4.655193534	0.000169905	0.001533424	0.216924317
NR2F2-AS1	-2.236946006	1.909778425	-5.270408005	4.27405497832337e-05	0.000601496	2.251318814
LINC00854	-2.234391491	-2.682503299	-3.937497894	0.000874817	0.004843979	-0.886021584
PRTG	-2.226070733	3.339338474	-4.47624008	0.000255184	0.002024073	0.511066235
SCART1	-2.218082266	1.510398751	-5.689891709	1.70358344949636e-05	0.000330969	3.069759881
OLIG3	-2.21583296	0.872480498	-5.657445181	1.82791752367497e-05	0.000345974	2.931764804
CRNDE	-2.215667916	4.424474132	-7.639143021	3.16984725893003e-07	.68812738082006e-0	6.93520448
COPG2IT1	-2.215615352	5.959530775	-8.909882067	3.09732654966686e-08	.10938949181891e-0	9.192455891
ALMS1-IT1	-2.209082783	0.84075265	-4.776485586	0.000129115	0.001277656	1.202198346
LOC101929679	-2.208660674	1.277080895	-6.744102222	1.85522935015872e-06	8.9087958638443e-05	5.00561259
DIO3OS	-2.208224443	-1.301114105	-4.483929668	0.000250753	0.002000528	0.354575628
VWA3A	-2.206248668	1.9597376	-5.365138784	3.46658543508486e-05	0.000520715	2.446135107
STX16-NPEPL1	-2.200228441	-0.107164548	-5.635739871	1.91624828469383e-05	0.000355372	2.699606814

ANKRD20A12I	-2.19989085	-1.711874724	-3.908994497	0.000933842	0.005078442	-0.742163019
GOLGA8B	-2.193949926	5.546816381	-9.768293022	7.21870734873986e-09	.81027864162052e-0	10.61819478
MIR647	-2.190660439	-0.350182147	-5.042278672	7.10276356037696e-05	0.000840325	1.566112777
GOLGA8A	-2.178568157	6.477761252	-6.037549356	3.07248076611383e-06	0.000212327	3.647826629
MST1L	-2.160710825	0.0289954	-3.646840484	0.001701137	0.007880972	-1.090734328
TPBG	-2.158486311	5.218596028	-4.371152429	0.000324285	0.002403129	0.073621233
LOC101927497	-2.156050947	-0.654315718	-5.462349615	2.7990540366193e-05	0.000451681	2.249603505
LOC100131564	-2.155345424	3.925770666	-7.54227011	3.81791574834029e-07	.00815327442139e-0	6.750748377
PAX7	-2.15092867	4.180569499	-3.840807448	0.00109167	0.005712268	-0.984189209
CNPY1	-2.148817111	3.264094053	-6.705876998	2.00546270869274e-06	9.1814003257369e-05	5.152437693
SLC26A7	-2.1439641	0.682403898	-3.652029645	0.001681098	0.007817878	-1.08581125
CFAP70	-2.140239356	2.209981406	-8.698577515	4.49451214884494e-08	.24395838519622e-0	8.485161342
CABP4	-2.137328237	-1.534084457	-4.379919405	0.00031786	0.00236994	0.122958311
LINC00173	-2.130695311	1.571320086	-7.061088317	9.8007010045113e-07	.41682456091068e-0	5.629472925
MIRLET7D	-2.119876418	-1.544953681	-3.928260566	0.000893526	0.004923692	-0.679925739
VGLL3	-2.118178406	4.138259541	-4.312901139	0.000370428	0.002631898	0.067174464
FKBP1AP1	-2.11398408	-2.488625412	-4.172753549	0.000510397	0.003312053	-0.453222599
AMY2B	-2.110831179	3.087645767	-8.398016129	7.70745269280098e-08	1.7169760773097e-05	8.185952335
SSPO	-2.107105443	2.986595658	-5.882576505	1.1240935690562e-05	0.000256033	3.513311551
LINC00899	-2.10671591	-1.420111301	-3.765501954	0.001297014	0.006449365	-0.955926972
BRWD1-AS1	-2.099041351	-2.753300474	-3.957559793	0.000835522	0.004697594	-0.865042332
CFAP43	-2.094550082	2.368580975	-6.355547336	4.13122352308568e-06	0.000139492	4.433794828
HESX1	-2.089971317	-0.219414567	-6.132670137	6.59771783829436e-06	0.000186276	3.542071648
LOC105747689	-2.082964147	-2.573072693	-3.81073537	0.001169471	0.005990671	-1.063538487
GDPD2	-2.076702242	4.530186756	-6.188308192	5.86649391153643e-06	0.000173935	4.08323076
PDK4	-2.069796651	0.587800355	-4.824073645	0.000115965	0.001181139	1.280736259
CSAD	-2.061986145	4.855030339	-6.013446952	3.49739508852217e-06	0.000218315	3.695430449
LOC101927391	-2.051667803	1.719742042	-8.61869021	5.18144215470525e-08	.35213434057144e-0	8.218557785
C9orf173-AS1	-2.048182067	-1.903785885	-3.954967209	0.000840499	0.004711077	-0.691272445
SLC25A21-AS1	-2.041270461	2.025378133	-8.951546973	2.87998226897275e-08	.07749918139914e-0	8.819884726
DNAH8	-2.038748039	-2.688303002	-3.852621415	0.001062535	0.005596693	-1.018557554
NRG4	-2.03822578	1.294609107	-7.617490743	3.30406010456382e-07	.76017217513928e-0	6.50657347
FER1L4	-2.036284232	2.447196689	-4.553262017	0.000214154	0.00178546	0.738552917
C22orf15	-2.034761834	-1.602606446	-4.408840894	0.000297562	0.002260106	0.161443984
SP9	-2.032562573	0.595915812	-3.812498339	0.001164762	0.005973245	-0.758794108
LINC00853	-2.027758028	-2.646992519	-3.540386433	0.002168602	0.00944086	-1.518612329
ZBBX	-2.024492934	2.265928037	-11.37479285	5.90874874310127e-10	.65204569766988e-0	12.16387761
LINC00441	-2.023397479	-0.704496057	-5.176098035	5.26947916645585e-05	0.000695001	1.736157889
CCDC140	-2.010257148	1.846565723	-5.078983329	6.54321895044009e-05	0.000797897	1.857475751
LHX5	-2.00657145	3.793182092	-4.360596681	0.000332194	0.002444881	0.207827127
FOXB1	-2.006307931	1.668281847	-3.653924099	0.001673841	0.00779405	-1.131764646
MSH5-SAPCD1	-2.003816637	-0.71014545	-3.731449588	0.001402073	0.006840725	-0.952378703
MIR8078	-2.001236155	-2.512164065	-4.660957194	0.000167699	0.001518768	0.324781351
LOC101929229	-1.998667354	-2.735588668	-4.176186591	0.000506403	0.003293149	-0.508757863
LINC00240	-1.996050478	-1.140774007	-3.523322311	0.002254525	0.009710258	-1.371074242
ZIC4	-1.983670733	5.490102196	-5.008350289	7.6632818997901e-05	0.000879304	1.463155801
GLIPR1L1	-1.978674951	-1.68822382	-5.616116489	1.99986855243441e-05	0.000363137	2.141973841
CYP4F24P	-1.977978022	0.722456259	-6.340869323	4.25973288166009e-06	0.000142514	4.172844969
PLEKHH2	-1.973793484	2.232719131	-5.320486073	3.8257497129283e-05	0.000559527	2.359455167
MIR135A2	-1.970609247	1.466114769	-4.476516844	0.000255023	0.002024073	0.604708905
CFAP54	-1.968832351	1.476881258	-4.522856377	0.000229489	0.001872293	0.701273995
SOX13	-1.968342562	4.264283499	-6.944996562	1.23615526141553e-06	7.3314442078823e-05	5.615318064
RFTN2	-1.963293175	3.758758831	-8.320261636	3.87821299129674e-08	.84293541718202e-0	8.133263826
LOC101927132	-1.962999589	-2.531721885	-3.533167318	0.00220455	0.009542778	-1.510739573
WSB1	-1.959660465	8.12179255	-8.074408696	1.39560486748657e-07	.33885450590616e-0	7.679505444
SPARCL1	-1.954420302	6.748599431	-4.901650893	9.73729343729311e-05	0.001037481	1.153498627
C9orf173	-1.950335928	-1.581902286	-5.869036609	1.15724928013989e-05	0.000258997	2.582167599
PKD1L2	-1.949051782	0.655740489	-4.15726411	0.000528819	0.003394215	-0.057046133
TSPAN19	-1.943882049	-0.031229115	-5.610282951	2.02544060144373e-05	0.000364054	2.6849066
SPAG8	-1.942375349	1.911721049	-6.394380698	3.81013559730924e-06	0.000132277	4.470595249
MIR155HG	-1.940398621	-2.04529338	-5.74221913	1.52103065247623e-05	0.000306674	2.173223766
GOLGA6C	-1.937105964	-2.13984814	-3.942809708	0.000864237	0.004807252	-0.752937562
ANGPTL3	-1.933872489	-2.268035931	-4.703200522	0.000152392	0.001427564	0.477136077

KIRREL2	-1.932231755	4.747304506	-5.631676233	1.93326544859652e-05	0.000357439	2.890821432
CCDC144CP	-1.931199182	1.249223655	-6.722294733	1.93945529562932e-06	.04002054757445e-0	4.971565087
GNG12-AS1	-1.92974457	0.485800706	-5.465851342	2.77762411934384e-05	0.000449813	2.517451869
COL7A1	-1.928346216	5.635798699	-7.693480326	2.85734520489683e-07	.45131166877267e-0	7.002578119
MIR1914	-1.926294243	-0.293248674	-5.231696314	4.65711408458329e-05	0.000635515	1.936623408
INTU	-1.922282377	4.285781833	-9.924849394	5.58682243210545e-09	.30882913325477e-0	10.79419225
ARMC2-AS1	-1.917613885	-2.594998391	-4.523315248	0.000229249	0.001871675	0.085876542
C4orf47	-1.913817546	1.532531846	-7.056868926	9.88342506862076e-07	.44787591887484e-0	5.621644194
ZNF30-AS1	-1.913725855	-1.246530546	-5.36965508	3.43223162983058e-05	0.000516872	1.916288887
LINC00106	-1.913156525	-0.570655349	-7.127902384	8.58209740872581e-07	5.8412609343474e-05	4.985299206
NAALAD2	-1.911417019	3.308978165	-9.867679416	6.13289305658422e-09	.30882913325477e-0	10.54021778
MORF4L2-AS1	-1.908270284	0.60390487	-4.609376264	0.000188519	0.00163163	0.855948768
UBE2E1-AS1	-1.902236967	-1.320488232	-3.881240667	0.000995131	0.005334027	-0.729733441
PTCHD4	-1.901924051	2.862832875	-7.172404239	7.85835613295443e-07	.53135291921259e-0	6.013595318
MIR6845	-1.898940032	-1.82158798	-3.744648343	0.001360384	0.006683737	-1.041993038
ACTG1P17	-1.89860965	-1.24985309	-4.865793502	0.000105557	0.001106899	1.051583667
SLC6A16	-1.897478634	1.715602075	-6.908865169	1.32926432385591e-06	.59340476344996e-0	5.393588316
ABCA1	-1.896767239	5.282625302	-3.971239075	0.000809745	0.004593666	-0.842087556
ANGPT1	-1.887302555	2.021427591	-4.722906428	0.000145744	0.001387339	1.11351931
HOGA1	-1.886236199	0.750970379	-4.688746368	0.000157463	0.00145719	1.024305113
ITGB8	-1.882952218	6.388960463	-5.178127441	5.24574353048261e-05	0.000692871	1.779516028
RPE65	-1.879336317	2.171205905	-4.765817093	0.000132266	0.00129945	1.200579827
C7orf34	-1.877787323	-1.744246622	-4.738106648	0.000140817	0.001354586	0.697046594
GATM	-1.874327898	5.283873132	-6.649137227	2.25199608163947e-06	.86503895043362e-0	4.973333576
HFM1	-1.874205007	2.435536759	-9.552725341	1.03208834159152e-08	.51504604169131e-0	9.84048676
IRX5	-1.870662298	4.634632986	-4.628512511	0.000180505	0.001589056	0.703697453
ADGRV1	-1.870074552	6.401555937	-4.755592202	0.000135358	0.001319416	0.836462579
ADAMTS6	-1.859622458	2.506749344	-5.639708938	1.8997752637906e-05	0.000353393	3.016878475
NPEPL1	-1.858343036	-0.065292669	-5.068871299	6.69273623737827e-05	0.000810687	1.679007176
SLC5A12	-1.857158707	-2.173902328	-5.196379961	5.03710357464883e-05	0.000675479	1.294588852
NPHS1	-1.856398398	0.374658299	-4.584033905	0.000199687	0.001704528	0.790504644
GRM1	-1.855970926	5.112156453	-5.725604928	1.57671090777657e-05	0.00031443	3.058161845
NEAT1	-1.850886213	7.421026981	-5.059578864	6.83320198193925e-05	0.000822307	1.492481248
KEL	-1.849203084	0.40920678	-5.556874312	2.27571700553657e-05	0.000393288	2.679077898
DMBX1	-1.848552533	1.687706813	-4.630512881	0.000179688	0.001586647	0.924477299
NOXA1	-1.847304136	1.845381814	-7.424028175	4.79930546952118e-07	.32251748804431e-0	6.323604497
INPP5D	-1.845916514	2.304126628	-3.702428257	0.001498256	0.007181486	-1.07935503
C14orf105	-1.845880449	1.15676597	-4.19925031	0.000480366	0.00315642	0.030115698
ACSS3	-1.845545235	2.415601937	-4.455378812	0.000267605	0.002101612	0.529071326
LRP2	-1.841696666	5.393621917	-5.172922285	5.30684223338452e-05	0.000697913	1.830123767
MIR34A	-1.834484391	-1.240446344	-4.156879349	0.000529285	0.003396013	-0.214393889
STRC	-1.834301942	0.713607699	-5.415862198	3.10011342199586e-05	0.000482777	2.457209614
CAPS2	-1.832095157	1.827653985	-7.035278609	1.03181615921625e-06	.63668513394479e-0	5.639242169
USP43	-1.831472858	1.745780991	-9.907243243	5.74936983115608e-09	.30882913325477e-0	10.07493775
TBC1D26	-1.827119854	0.404822476	-3.766049703	0.001295389	0.006447651	-0.851719187
LOC10099658	-1.823168175	-2.60889302	-4.017235584	0.00072876	0.004269489	-0.726173048
CCDC102B	-1.819337061	3.409666516	-7.048135299	1.00569558404214e-06	.53773709384263e-0	5.813525503
TFAP2B	-1.815986259	6.195291615	-4.442480239	0.00027559	0.002145945	0.140859737
C2orf73	-1.809935918	-2.116806254	-3.54943846	0.002124344	0.009292477	-1.420176307
LOC10192830	-1.809321804	3.04063316	-7.382167808	5.20651978404576e-07	.49578093055907e-0	6.413475074
LOC645434	-1.807912365	-1.254747646	-5.976800265	9.18798440309991e-06	0.000229285	2.905526127
PDE6C	-1.80636534	-0.456749276	-4.704355821	0.000151994	0.001424722	0.928568114
ILDR2	-1.804395821	6.358107454	-4.957423541	8.59036787054788e-05	0.000952186	1.288753705
ZSWIM2	-1.788195296	-1.800885367	-3.860145897	0.001044385	0.005524853	-0.833731612
KHDC1L	-1.78771759	-1.424004054	-3.612343584	0.001840502	0.008364987	-1.229491626
THRB	-1.782598917	2.644605583	-4.879243696	0.000102409	0.001081245	1.424923785
C6orf118	-1.776952691	2.420202648	-4.91623006	9.423236729657e-05	0.001017944	1.511558355
LCN12	-1.773939515	-0.879561337	-5.315130182	3.87131088521109e-05	0.000562587	1.943482685
ZIC5	-1.77374749	4.590454591	-5.050334132	6.97592642520735e-05	0.000831479	1.63873092
HERC2P7	-1.770352818	-1.892177094	-4.643247649	0.000174571	0.001558626	0.499250571
LINC00271	-1.767225206	-1.114184076	-4.57607305	0.000203332	0.001729177	0.572348388
LINC01280	-1.766304259	-1.57050207	-3.58351514	0.001965595	0.008754678	-1.295734432
CYP26B1	-1.765800745	6.442754421	-5.09695661	6.28580809919801e-05	0.000776954	1.595535775

MST1P2	-1.763179628	1.69717009	-4.998298224	7.83784396754893e-05	0.000891494	1.689379114
PDCL3P4	-1.756946752	-0.581465594	-4.69296126	0.000155967	0.001449163	0.887929674
LOC101927765	-1.755600774	-0.241075415	-3.961312198	0.00082837	0.004670322	-0.478409469
SEC31B	-1.753961206	4.538565325	-6.821266744	1.58632226533879e-06	.16263347068835e-0	5.359505705
ZIC3	-1.748619986	6.551316798	-5.766903127	1.44200283085152e-05	0.000298091	3.059951603
SOX2-OT	-1.744745965	3.975992962	-5.954485035	9.63650363647985e-06	0.000235965	3.627169644
ENPP2	-1.738008143	5.250962007	-4.022161557	0.000720583	0.0042338	-0.729959699
PHEX	-1.736817069	2.451622391	-4.378678681	0.000318762	0.002373754	0.361766299
LOC101929741	-1.733816082	-0.675943593	-4.979283247	8.17920092018409e-05	0.000919443	1.398045733
SAP25	-1.72541367	1.826185801	-5.046010572	7.04371363581261e-05	0.000835503	1.789515428
MSTN	-1.720939826	-0.121554769	-4.064715085	0.000653656	0.003965573	-0.268426297
ZNF83	-1.719878093	5.04815607	-9.705317769	3.00890483156405e-09	.81731275683239e-0	10.50950834
AQP6	-1.719399966	-1.926484262	-4.642398265	0.000174907	0.001559314	0.489464835
CCDC39	-1.71847789	3.210752257	-6.715348267	1.96710548464345e-06	.12008017461469e-0	5.170839755
KC6	-1.717982516	2.455331776	-4.58301742	0.000200149	0.001706874	0.800040541
GOLIM4	-1.717956102	5.379649236	-6.067996291	7.56674440027037e-06	0.00020357	3.760731624
UNC5CL	-1.717049323	1.110515869	-5.610005468	2.02666529477664e-05	0.000364054	2.883075724
GBX2	-1.714721871	3.489796072	-4.602238227	0.000191599	0.001648581	0.765561824
PAQR6	-1.712600508	3.769513132	-5.079517629	6.53541403061205e-05	0.000797487	1.780786147
CCNB3	-1.710002623	1.221840562	-4.39828149	0.000304817	0.002304671	0.442869927
NTRK2	-1.706106009	8.884600265	-5.608491419	2.03336103904154e-05	0.000364479	2.699210957
INO80B	-1.705810291	-0.599369621	-5.912238282	1.05482448035791e-05	0.000249592	3.06149534
SPACA6P-AS	-1.705679768	-1.833891381	-4.549316183	0.000216084	0.001793465	0.357517647
C11orf65	-1.700169717	-0.732426257	-4.912277109	9.50735782210608e-05	0.001024003	1.264245504
MPL	-1.699567481	-1.837947316	-4.300441665	0.000381126	0.002683577	-0.069834896
PTN	-1.698715671	9.808320195	-5.069168504	6.68829248428849e-05	0.000810687	1.512930492
MAP1LC3B2	-1.698233199	-0.283658392	-4.412447634	0.000295124	0.002250724	0.397147368
MIR29C	-1.691640855	-1.465873226	-4.385753423	0.000313656	0.002351055	0.15989247
LINC01197	-1.691497667	-1.022938663	-4.01421453	0.000733822	0.004293632	-0.444690374
VSTM2A-OT1	-1.688795377	1.927227838	-5.951264872	9.70305906260118e-06	0.00023696	3.620435592
MIAT	-1.686920536	9.697207127	-6.957407902	1.20574947321701e-06	.19584894824476e-0	5.52684447
PSCA	-1.686834294	-1.506211702	-3.977263177	0.000798646	0.004546237	-0.577577792
NEK5	-1.685820225	-0.5051259	-4.180092018	0.000501896	0.00327083	-0.073436506
PROX1-AS1	-1.683529906	-0.472744752	-3.870327937	0.001020314	0.00543226	-0.668241347
WNT5A	-1.682686635	4.423421524	-4.94140387	8.90498452568194e-05	0.000975224	1.413819892
HNRNPU-AS1	-1.68120006	4.068506601	-5.645270638	1.87693597725093e-05	0.000351651	2.970554166
CYP39A1	-1.679778318	0.383121538	-5.216387135	4.81810866546211e-05	0.000653396	2.032862492
MEIS1-AS3	-1.678722803	1.75596183	-3.620446561	0.001806783	0.008257321	-1.211029121
ZIC1	-1.67595968	8.172081117	-5.334538068	3.70879830606456e-05	0.000548174	2.098370149
APCDD1	-1.675012814	5.794973378	-4.380964898	0.000317103	0.002365257	0.023420219
ABCA8	-1.67396653	3.875066958	-3.849590193	0.001069936	0.005622703	-0.93799834
CFAP44	-1.673710032	3.223524891	-4.973312496	8.28947867226048e-05	0.000926969	1.593478602
GAD2	-1.666396161	4.546066655	-4.230777212	0.000446937	0.002997639	-0.179832524
MUC1	-1.665085965	1.644722093	-6.836638148	1.53775060770121e-06	.04873649022293e-0	5.256705415
MIR219A1	-1.658362624	-0.012756666	-6.187531726	5.87610285973056e-06	0.000173935	3.725128469
CXXC4	-1.652461621	5.643409704	-7.467339896	4.41256411871354e-07	.21541783534531e-0	6.567556026
MEG9	-1.65027306	-2.16055211	-3.808326579	0.001175937	0.006015264	-0.97962752
ANKRD30B	-1.645177461	0.120312477	-5.753903617	1.48308262765478e-05	0.000301687	2.991682267
FPGT-TNNI3K	-1.638587366	-2.376562927	-3.77801317	0.001260412	0.006324961	-1.06917038
MAPK15	-1.635525894	2.587004855	-3.870006821	0.001021065	0.005433697	-0.746531197
TTC14	-1.634856945	5.80978211	-7.877521881	2.01634179334863e-07	.85523376272088e-0	7.338499269
EBF1	-1.634141617	5.462964488	-5.274219183	4.23812491134161e-05	0.000598283	2.041080281
ADAM28	-1.633866197	0.622593894	-4.893037098	9.92782553136233e-05	0.001053775	1.427468483
ZNF117	-1.626465027	5.027780141	-5.847828994	1.21121205080103e-05	0.000266248	3.319744411
RGPD4	-1.621079564	-1.263722193	-5.062138006	6.79422072355151e-05	0.000819749	1.398424351
NRBP2	-1.612076443	6.365809675	-10.18317614	8.68262683592458e-09	.36352722059172e-0	11.28253592
FLJ31104	-1.609927225	-0.130727864	-4.277631354	0.000401525	0.002774075	0.150117983
FOXP2	-1.604621124	6.166883175	-4.747425662	0.00013788	0.001336869	0.823470997
FLJ37201	-1.602136647	0.979038719	-5.984752195	9.03338807182199e-06	0.000226606	3.583783856
ZNF192P1	-1.601110777	2.191207146	-4.940052185	8.93206104337229e-05	0.000977018	1.566843936
LINC00202-1	-1.601103903	0.162717406	-3.829186117	0.001121105	0.00581631	-0.725728721
RPGR	-1.59968765	3.13187499	-7.525465301	3.9436341815897e-07	.02448969805022e-0	6.682730151
LOC339539	-1.598609668	-1.320423143	-5.01282035	7.58693149287628e-05	0.000872187	1.297434239

MEIS1	-1.598120525	5.8190856	-6.081156117	7.35834653009924e-06	0.00020073	3.757961246
NDNF	-1.596135936	3.056291552	-4.05158802	0.00067361	0.004046402	-0.397857759
LOC100132215	-1.5960794	1.025060368	-3.633849541	0.001752348	0.008056918	-1.139398496
CEP290	-1.594979447	5.713189687	-11.1755163	7.94272864537736e-10	1.65204569766988e-06	12.76659973
LOC100129617	-1.594131046	-0.538010877	-5.516304434	2.48683690232679e-05	0.000419069	2.400653798
PDPN	-1.591140794	4.998399612	-6.007762566	8.60091624517115e-06	0.000220046	3.660024457
PLP1	-1.589775522	6.05859096	-4.836170924	0.000112844	0.001161891	1.027370926
LOC100507006	-1.58861891	1.552751982	-4.432634987	0.000281845	0.002182477	0.511010201
MIR3186	-1.585248551	-2.156171138	-3.810736416	0.001169469	0.005990671	-0.973782755
NLRP14	-1.579141825	-0.668570161	-5.766724171	1.44256035544118e-05	0.000298091	2.796974199
MAEL	-1.577794206	-0.344157541	-4.040684	0.000690647	0.004125589	-0.328235413
EBF2	-1.576394955	4.260181045	-5.000852274	7.79311102765564e-05	0.000888619	1.559477679
DNAH7	-1.576220886	2.550850672	-3.843915389	0.001083929	0.005678645	-0.80004354
NTN5	-1.575163695	0.311177212	-5.630302377	1.93905365188264e-05	0.000358147	2.806978443
MST1	-1.57287332	3.535693506	-7.36888686	5.34306522969791e-07	4.5186005810598e-05	6.420768014
LOC101929234	-1.571152938	-0.849682983	-4.412125431	0.00029534	0.002250724	0.323442221
NKG7	-1.568756385	-1.209552602	-4.20683948	0.000472096	0.003119056	-0.112829948
CCDC37	-1.567613891	0.449962288	-4.430475219	0.000283236	0.002190465	0.491596885
LOC101928068	-1.562123286	1.285705441	-6.678398096	2.12117775895374e-06	9.42762874034256e-05	4.911573205
AHSA2	-1.55933512	4.901501094	-6.864073185	1.45484076820165e-06	7.77640583574562e-05	5.422217939
PROX1	-1.558345732	3.45366642	-4.000464321	0.000757305	0.004394439	-0.554553335
TMEM257	-1.557002759	0.148206928	-4.119281444	0.000576866	0.003617445	-0.145868982
MUC6	-1.556698939	1.698402989	-3.84994551	0.001069066	0.005621365	-0.724208471
NPHP3-ACAD11	-1.555207138	-0.191774871	-5.084314996	6.46575868888336e-05	0.000792685	1.694019046
STK33	-1.552599681	3.581673833	-6.91966543	1.30069662976712e-06	7.55341092069203e-05	5.572261587
PRAM1	-1.552429903	0.137315228	-3.980366196	0.000792989	0.00453257	-0.423837356
IFI44L	-1.551596496	4.233390629	-5.285782583	4.13098577850399e-05	0.000588158	2.182865341
JAKMIP2-AS1	-1.548981215	0.018521691	-4.155873226	0.000530506	0.003401455	-0.079278712
LINC00472	-1.547016235	2.695073037	-5.062323542	6.79140343573029e-05	0.000819749	1.811190864
ARL4A	-1.545767853	4.842833591	-5.057727035	6.86155128782786e-05	0.000823521	1.621156613
LRRC9	-1.545500959	1.290510648	-4.036018145	0.000698069	0.004148217	-0.314251913
UACA	-1.544558746	4.217757721	-7.210248836	7.29264823526107e-07	5.35613094958005e-05	6.127143162
MAF	-1.540555085	5.084221835	-4.281398887	0.000398081	0.002757586	-0.136782933
MAN2C1	-1.539488947	5.897765454	-11.94588288	2.58556586342677e-10	1.65204569766988e-06	13.8540643
ADCY10P1	-1.537969868	1.489676608	-6.006742187	8.61963562724401e-06	0.000220217	3.69275205
LOC100507487	-1.536520379	-0.296644487	-3.897704247	0.000958306	0.005185962	-0.604254662
RSRP1	-1.534158912	4.432138471	-10.59074468	1.93675319784365e-09	2.78394289080403e-06	11.8203227
TRIM45	-1.531034085	3.77610626	-7.830587432	2.20286369289133e-07	3.01117265661626e-05	7.275495773
CNGA4	-1.530435753	-0.514848956	-4.503366048	0.000239898	0.001938174	0.545315708
MAMDC4	-1.529012878	4.401468425	-6.779928037	1.72496624906e-06	8.6328653346792e-05	5.281319834
TEX9	-1.523533472	3.259949186	-6.437742037	3.48182220574525e-06	0.000124801	4.628680175
MFSD7	-1.521307536	-1.49273157	-3.59054172	0.001934351	0.008651903	-1.272692033
BCRP3	-1.51812441	0.59701942	-5.574194419	2.19126018022349e-05	0.000382674	2.751522714
MAATS1	-1.517342507	3.400844645	-8.157156122	1.19746761431834e-07	2.14452361870128e-05	7.825198531
SPATA9	-1.514498007	-0.813504229	-4.620020772	0.000184018	0.001609891	0.716631409
PRMT5-AS1	-1.512076569	-2.272154192	-4.23410683	0.000443546	0.002983155	-0.279539966
MORN5	-1.509924418	0.742153432	-4.011264327	0.000738798	0.004319981	-0.354773744
BDH2	-1.508807122	4.395327861	-7.293948157	6.18619416241339e-07	4.94849120009145e-05	6.283026803
DNAI1	-1.507734804	1.361366765	-6.116168331	6.83215031313806e-06	0.000191122	3.887415866
LINC-PINT	-1.507635976	2.68173779	-4.27354848	0.00040529	0.002790701	0.11779933
LRP4-AS1	-1.50639996	-0.061284091	-4.532385122	0.000224567	0.001842841	0.656230564
LINC00158	-1.503636874	-1.489548173	-4.373007689	0.000322914	0.002397426	0.137032697
HIPK1-AS1	-1.502566602	-1.118280566	-3.661505891	0.001645107	0.007713338	-1.109102853
LINC01089	-1.501787431	4.801955439	-6.684193849	2.09621085939244e-06	9.38516759032394e-05	5.067108641
NPPC	-1.500452733	2.638596807	-5.640600502	1.89609495526855e-05	0.000353393	3.019307434
AKNAD1	-1.500408752	-0.396377412	-3.515402059	0.002295541	0.009824893	-1.351848225
AURKAPS1	-1.498632014	0.780650186	-4.337260935	0.000350377	0.00253118	0.31388453
MIR1247	-1.496940468	0.142966425	-3.687037559	0.001551894	0.007365145	-1.009792697
UNC13D	-1.495967458	1.791818405	-5.345049996	3.62370252171166e-05	0.000537448	2.402889738
CENPE	-1.495374075	3.386428742	-4.61107789	0.000187792	0.001630413	0.792658242
NR2F2	-1.494639661	7.613184933	-5.013108873	7.5820300972199e-05	0.000872187	1.386235524
HES5	-1.492537011	3.913750433	-4.413795225	0.000294218	0.002247986	0.301820763
LY6G6C	-1.492026894	-0.47884581	-4.38515773	0.000314083	0.002351055	0.325172349

ACRBP	-1.487516941	0.615282375	-5.896249251	1.09160123945925e-05	0.000253703	3.360127984
RFX3-AS1	-1.485577685	1.038573888	-5.494327476	2.60946862661184e-05	0.000432944	2.653925614
SEC1P	-1.483435609	-0.667294997	-4.554794012	0.000213409	0.001781691	0.620575489
MIR124-2HG	-1.483174087	5.6964302	-7.225331729	7.07908685576353e-07	.29269443978056e-05	6.093434148
HEPACAM2	-1.48308388	-1.947168993	-3.893983957	0.000966507	0.005214169	-0.792658084
ZNF663P	-1.482863375	0.28332552	-3.505975794	0.002345316	0.009963228	-1.373253566
CRTC3-AS1	-1.482767872	-0.516622909	-4.881518321	0.000101885	0.001077674	1.257148798
LOC100270804	-1.482427515	0.847045105	-4.537174886	0.000222133	0.001831924	0.72422025
IRX2	-1.48124888	5.23454595	-4.377651336	0.00031951	0.00237642	0.061071434
AP1G2	-1.481145374	3.888879669	-8.718527088	4.33822910725387e-08	.24395838519622e-05	8.827841645
LOC100133050	-1.480783106	-1.628754817	-3.628712091	0.001773018	0.008141711	-1.217761867
WDR63	-1.478157757	0.893408941	-3.80832976	0.001175929	0.006015264	-0.775231605
CCDC78	-1.476143146	2.869532247	-7.825204953	2.22536983983793e-07	.01117265661626e-05	7.188269683
OCLM	-1.473127978	-1.75297641	-3.627752344	0.001776907	0.008157516	-1.23330377
LINC00461	-1.468764369	7.942475494	-4.570898339	0.000205738	0.001742117	0.39300202
ZNF334	-1.46812475	4.765893613	-7.807198166	2.30241227205587e-07	.02576726429098e-05	7.239219278
RGN	-1.467319133	-1.16941831	-3.762628137	0.001305569	0.006477635	-0.925560139
MIR92B	-1.466406606	0.507479411	-5.184843462	5.16796845074688e-05	0.00068607	1.992300099
KIAA1407	-1.461232491	4.004550227	-11.36126074	6.02789694659733e-10	.65204569766988e-06	12.82571016
KCNH8	-1.460714066	4.753398468	-6.915263582	1.31226255372202e-06	.55341092069203e-05	5.530662496
LINC00482	-1.458073023	-1.993657878	-3.82614258	0.001128944	0.005838856	-0.91802559
WLS	-1.456488004	7.460094746	-3.725797262	0.001420313	0.006905736	-1.509298013
LRP2BP	-1.456454762	3.151078685	-8.185397891	1.13672910547227e-07	.07646305696619e-05	7.845986103
LOC101929378	-1.456405936	0.674362909	-5.624913522	1.96192939094984e-05	0.000360331	2.861138857
LAG3	-1.454056039	1.615525756	-6.688589411	2.07747804878415e-06	.38516759032394e-05	4.987374849
PARD3-AS1	-1.451729347	-2.149604732	-3.805678174	0.001183087	0.006033348	-0.97909598
WDR11-AS1	-1.45156731	0.446957577	-5.088063923	6.41185282268218e-05	0.000787662	1.796981288
TEX14	-1.44745918	1.134005567	-4.851070722	0.000109117	0.001135105	1.374363028
SNORA25	-1.447311394	-1.853683102	-3.82601003	0.001129287	0.005838856	-0.896005859
RORA	-1.444553737	6.686429808	-4.241542574	0.000436066	0.002943685	-0.33696205
DCDC1	-1.443160461	-0.769773059	-4.173925432	0.00050903	0.003306705	-0.11112299
STRIP2	-1.442262125	1.979112643	-4.366736547	0.00032757	0.002421582	0.358675824
CFAP69	-1.440257779	2.1395276	-7.175309801	7.81335600926714e-07	.53135291921259e-05	5.943033806
PLAG1	-1.43923693	3.390288521	-5.153372772	5.5428703166835e-05	0.000719117	1.967473803
MIR4712	-1.439142893	1.366060017	-4.179871231	0.00050215	0.003271316	-0.015517516
LGR4	-1.438977677	5.555068545	-4.974071894	8.27536931178325e-05	0.000926263	1.364591557
MGC32805	-1.437110909	-2.084048426	-4.147348934	0.00054096	0.003449896	-0.380769972
LOC339874	-1.435856699	-0.663207985	-4.399144753	0.000304217	0.002302042	0.328957151
FAM166A	-1.434625123	-1.2826394	-3.739973023	0.001375009	0.006735662	-0.978407309
GOLGA2P5	-1.433639214	1.238797006	-6.470683067	3.25198837039662e-06	0.000119766	4.530473749
SSPN	-1.433566086	2.890633438	-5.068591562	6.69692155146288e-05	0.000810687	1.815840285
RDH16	-1.431841733	-0.866022331	-4.003931362	0.000751314	0.004369389	-0.443187848
RFX3	-1.42857377	6.074089058	-7.705680857	2.79167817627465e-07	.42105385703572e-05	7.005800289
CDK6	-1.428499584	5.858549565	-4.648107165	0.000172657	0.001548294	0.613793111
IRX1	-1.428431249	5.356740065	-4.032402857	0.000703874	0.004170504	-0.728765941
GNRH1	-1.427435806	0.626724028	-5.258493231	4.38840036667861e-05	0.000612398	2.15029051
LOC100507053	-1.427381752	2.032732439	-4.676478235	0.000161902	0.001485357	1.014098133
CALCRL	-1.426281841	2.046506521	-3.902134577	0.000948631	0.00514203	-0.636363935
LINC01355	-1.422143356	1.106830083	-5.232987171	4.64379258846592e-05	0.000634941	2.144057471
FBXO24	-1.421828253	-0.390367656	-4.062902655	0.000656376	0.003976787	-0.28713782
MIRLET7DHG	-1.42015572	0.60099072	-3.761139111	0.001310024	0.00649219	-0.864925753
GOLGB1	-1.419343419	6.774275092	-8.049919735	1.46055403775705e-07	.38213755425964e-05	7.639051883
CLDN10	-1.418754171	-0.677551626	-3.510738762	0.002320033	0.009888019	-1.367528986
ZMAT1	-1.41723621	4.436046655	-7.433425102	4.71251503096822e-07	.30228842931158e-05	6.54689963
LOC101928812	-1.415100653	0.544525699	-7.5828134	3.53141112374283e-07	3.9095931513582e-05	6.229320206
AASS	-1.41506508	4.993643702	-3.722082421	0.001432428	0.006942468	-1.381307928
C20orf203	-1.413996109	1.904263786	-4.896186191	9.85773397218622e-05	0.001048357	1.478157756
MYO15B	-1.413320204	3.493171151	-6.418335378	3.62499936268705e-06	0.000128506	4.58914234
MIR100HG	-1.411967674	7.196297305	-5.672588113	1.76877255073826e-05	0.000338629	2.841211984
LOC100507283	-1.408772386	1.337684687	-6.632631654	2.32943390656738e-06	.99831561127921e-05	4.842345933
TPR	-1.406859278	6.933056305	-5.758175214	1.46945318278203e-05	0.000300252	3.029602796
ZIC2	-1.406654178	6.763257608	-4.560878227	0.000210477	0.00176124	0.381244828
HSD17B13	-1.406293838	-1.913749737	-4.099060262	0.000604208	0.003752826	-0.428316557

TLCD2	-1.405611503	2.319262825	-4.062024143	0.000657698	0.003981809	-0.312962375
SMOC1	-1.404180999	6.255367868	-4.272381929	0.000406373	0.002792781	-0.253661334
SEPP1	-1.403910998	2.252944164	-3.859146	0.001046779	0.005532843	-0.743507384
DICER1-AS1	-1.40380173	2.938683045	-6.042108588	7.99459200482376e-06	0.000211329	3.836698947
LOC401320	-1.403527036	3.64594734	-8.765266735	3.99381053654451e-08	.22666735911394e-0	8.882615868
GOLGA4	-1.402514758	6.561630628	-8.281982871	9.52105812818121e-08	.91731949043276e-0	8.067202033
HERC2P2	-1.39282778	6.33757526	-4.337111563	0.000350496	0.00253118	-0.111025052
DLL1	-1.392021426	5.651401825	-4.428962953	0.000284215	0.002196172	0.134889081
ITPR1-AS1	-1.391873629	0.220927876	-3.722414308	0.001431341	0.006940885	-0.939099386
YY2	-1.390155986	1.195632154	-7.002544006	1.10153701174262e-06	.88001296680127e-0	5.470940166
LINC01013	-1.389610295	-1.581945944	-4.56412156	0.000208931	0.001754059	0.455265552
PTCH1	-1.388853806	5.249933991	-5.973528211	9.25238449118178e-06	0.000230305	3.563029928
CRISPLD1	-1.387419515	5.484167594	-4.692278178	0.000156209	0.001450669	0.739967607
TARBP1	-1.387349122	5.307049764	-6.337468396	4.29009194303037e-06	0.000143134	4.322159403
HMGN5	-1.386749489	3.885338631	-4.241089188	0.000436518	0.002945651	-0.079009233
CCDC144NL	-1.386712939	1.987165115	-4.590162696	0.000196926	0.001682533	0.832607883
HHIP	-1.386600309	0.045269092	-4.985578348	8.06454793036282e-05	0.000908791	1.548779784
CCND1	-1.386380557	5.822805445	-3.722548289	0.001430903	0.006940602	-1.465321122
SEPT7-AS1	-1.385968395	0.882090346	-5.748903591	1.49920108560249e-05	0.000302941	3.128369551
LCA5L	-1.385420672	0.208291917	-4.978173169	8.19959016809306e-05	0.000920602	1.556076693
CD37	-1.384044788	1.692846261	-5.094022469	6.32711508274906e-05	0.000780928	1.887227493
PCOLCE-AS1	-1.383943581	-0.505590889	-5.175328897	5.27850347382535e-05	0.000695187	1.803239377
MKRN7P	-1.383057033	-0.54637016	-4.225964439	0.000451885	0.003021441	0.014028346
FAM227B	-1.382889651	1.674440148	-7.702394985	2.80920829121015e-07	.42105385703572e-0	6.770459404
ZRANB2-AS1	-1.381443008	1.186433628	-4.208075253	0.000470763	0.003112353	0.04703672
CRABP1	-1.379252813	6.491715546	-4.709884434	0.000150104	0.001411189	0.723371391
CCDC154	-1.37923831	2.190159853	-4.689874961	0.000157061	0.001456339	1.037738701
FANK1	-1.377777204	1.828426427	-5.217134038	4.81012386375173e-05	0.000652797	2.142659556
INTS6-AS1	-1.375459684	0.500277759	-6.321531524	4.43535271368728e-06	0.000145063	4.111174525
CDC37L1-AS1	-1.375028166	0.38984836	-7.198497242	7.46365585793046e-07	.43181679509226e-0	5.562229616
ACTG1P4	-1.373044071	0.593406349	-4.289281388	0.000390973	0.002727441	0.212438565
LINC00926	-1.371049279	0.21256466	-8.130701065	1.25742335381701e-07	2.2300342139976e-05	6.901305952
PIFO	-1.368969129	3.447805925	-3.680103263	0.001576679	0.007457584	-1.257530478
NR2F1	-1.367716754	8.249280632	-5.610596653	2.02405696668582e-05	0.000364054	2.703073257
AMT	-1.367667711	3.731408187	-6.736814488	1.88294803402866e-06	.95894667483828e-0	5.216357338
MYO5C	-1.367011597	1.812517546	-3.941427173	0.000866978	0.00481517	-0.53824814
LINC01515	-1.366794148	0.216647231	-3.583541886	0.001965475	0.008754678	-1.21726409
GLYCTK	-1.366610612	3.16908996	-3.814186519	0.001160269	0.005956897	-0.93003382
C400927-CSNK	-1.366257668	0.697728789	-4.560176906	0.000210813	0.001762436	0.766325507
CEP112	-1.364695411	3.055348547	-6.492741118	3.10687912944892e-06	0.000115118	4.73439137
LINC00928	-1.364637663	2.712637853	-3.715522357	0.001454074	0.007019442	-1.093779041
NPPA	-1.362142942	0.115746242	-3.616440423	0.001823377	0.008308213	-1.150549631
ARHGAP30	-1.360622525	0.825187302	-5.871195144	1.15189706714491e-05	0.000258816	3.352366027
ANKDD1B	-1.357326152	-1.712487831	-4.257576309	0.000420365	0.002866298	-0.109874896
SPATA4	-1.355636726	-0.322910526	-4.246487378	0.000431162	0.002915867	0.075798267
GOLGA8N	-1.35501765	1.697636012	-6.072525307	7.49434700369824e-06	0.000203115	3.841292821
SNORA11	-1.350701948	-0.670171484	-3.648790054	0.001693581	0.007849626	-1.103132966
CATSPERG	-1.350500338	3.257863576	-6.321121881	4.43915258908165e-06	0.000145063	4.39686171
ZNF483	-1.3502704	2.920170576	-4.22475545	0.000453136	0.003027593	-0.008950978
GOLGA6L10	-1.349293864	1.416354672	-4.47146172	0.000257977	0.002039142	0.593831621
PRR25	-1.346728671	-0.995120713	-3.945675574	0.000858582	0.004780161	-0.565458646
FRY-AS1	-1.346674273	0.352312274	-4.343113978	0.000345724	0.002510074	0.312940686
BRCAT54	-1.345674	1.527870111	-5.391517391	3.27078536452625e-05	0.000500397	2.487101451
BMPR1B	-1.34455327	3.221104168	-4.903142541	9.70467847412067e-05	0.001034895	1.440172663
GK5	-1.343068119	5.287253211	-8.704422809	4.44812324621438e-08	.24395838519622e-0	8.841112387
MCC	-1.343005579	5.180628356	-4.375587608	0.000321018	0.002385697	0.057461751
CCDC159	-1.342427361	2.510399728	-7.676082108	2.95377042466853e-07	.48106608693032e-0	6.881097105
FIGNL2	-1.340911929	1.655079632	-5.151855642	5.56162878476961e-05	0.000721038	2.005224102
FAM181A	-1.339145093	3.077596387	-4.71115817	0.000149671	0.001407851	1.035463057
ZNF518A	-1.338978667	5.303825962	-6.170509149	6.09087994324567e-06	0.000176887	3.973261594
LOC643923	-1.338896543	-0.712901193	-4.683681439	0.000159281	0.001467262	0.856132716
CCDC18	-1.337698556	3.050625592	-9.443269175	1.24009769030531e-08	.96128013389661e-0	9.85779026
GOLGA6L9	-1.336626975	4.373629788	-5.141411708	5.69253050627491e-05	0.000731262	1.849858929

DLGAP1-AS2	-1.334270163	-0.104733988	-4.389268018	0.000311151	0.002338045	0.374344383
MYH15	-1.332995434	1.715704927	-4.421532377	0.000289072	0.00222242	0.483460068
DCDC5	-1.332205789	1.281187516	-4.390827246	0.000310045	0.002332819	0.426762945
AGER	-1.330490018	3.213796572	-4.798176928	0.000122943	0.001237357	1.213509237
PLCG1-AS1	-1.328303775	0.084935912	-4.03013434	0.000707541	0.00418213	-0.325041899
RFX4	-1.326255641	5.218978089	-4.538812531	0.000221308	0.001825937	0.419339711
ZNF730	-1.324847667	1.64832992	-4.358123678	0.000334075	0.002453781	0.351451694
ETAA1	-1.324398336	3.789040878	-8.362555258	8.2201100529155e-08	.76655000396009e-0	8.215728133
ZNF436-AS1	-1.323550827	3.001729475	-7.013219376	1.0782797863705e-06	.82668552070031e-0	5.73125339
RGS5	-1.3234045	2.218667062	-6.72472687	1.92986934445931e-06	.03921110647135e-0	5.129017637
KLHL7-AS1	-1.322842756	0.855288841	-6.119308608	6.78688704993072e-06	0.000190439	3.82091529
COL4A3	-1.322464766	-0.304144536	-3.766419613	0.001294294	0.006443952	-0.860159901
KITLG	-1.322018801	6.745767686	-3.565866699	0.002046289	0.009037697	-1.856032272
DCST2	-1.321715396	0.63985259	-5.613880216	2.00963222391432e-05	0.000363279	2.838237421
TTPA	-1.320067748	0.523222624	-4.955598606	8.62563047797606e-05	0.000954337	1.547030639
FAM13A-AS1	-1.319449105	1.752598716	-4.719630085	0.000146829	0.001389322	1.109876519
SEMA6A-AS1	-1.319362916	0.231055267	-3.721704181	0.001433667	0.006946632	-0.940584418
TEX21P	-1.318527747	-0.195599375	-4.805502386	0.000120927	0.001223131	1.170574488
HSF4	-1.317386617	4.449564632	-4.71204287	0.000149372	0.001406313	0.894519751
QKI	-1.317185726	8.586952023	-3.904845984	0.000942758	0.005117787	-1.109580627
SERHL	-1.315224203	1.143288523	-4.630642677	0.000179635	0.001586647	0.923327514
CD72	-1.313837414	-0.015119355	-4.648743693	0.000172408	0.001547606	0.890750535
MIR3916	-1.312169742	-0.134797328	-4.732564484	0.000142594	0.001365689	1.038982911
CEP162	-1.311583317	3.492187932	-4.778285427	0.000128592	0.001274299	1.146150992
FAM132A	-1.310687268	1.258631613	-3.98007496	0.000793518	0.004532583	-0.431126349
GOLGA6L3	-1.310236625	1.05398995	-4.084774551	0.000624303	0.003844959	-0.207368766
MEGF10	-1.309502416	6.828951958	-3.806688907	0.001180353	0.006025747	-1.320868252
LOC101928063	-1.309022425	-0.462916629	-5.39371422	3.25499757974989e-05	0.000498817	2.211136134
AK7	-1.307565261	1.646679984	-4.125511059	0.000568696	0.003574799	-0.140057236
LOC101927359	-1.306520712	1.981186899	-4.734729806	0.000141897	0.001362076	1.138071259
HRSP12	-1.305872137	3.944212731	-7.369761264	5.33396219723439e-07	4.5186005810598e-05	6.431826143
PPIEL	-1.305857111	3.014874541	-5.871755057	1.15051288588345e-05	0.000258816	3.490341356
SLITRK2	-1.304633908	4.627442176	-3.596230871	0.001909414	0.008576165	-1.61360954
LOC101928020	-1.304094457	-0.708496218	-4.318244393	0.000365933	0.002609095	0.171585459
LRRIQ3	-1.303705341	-0.300769309	-4.97442572	8.26880369727492e-05	0.000926263	1.474227708
FMO4	-1.302895015	0.033676911	-4.956285436	8.61234184785924e-05	0.000953465	1.492713473
GNB3	-1.30258373	3.597925807	-5.672947515	1.76739287639785e-05	0.000338629	3.057221331
NHLH2	-1.302482956	4.436955279	-3.883681675	0.000989583	0.005316681	-0.949310017
ZFC3H1	-1.301798226	5.314360285	-8.662544033	4.79181239871859e-08	1.2974681084082e-05	8.767445808
LOC100289230	-1.300955347	1.506037236	-5.673018809	1.76711932679499e-05	0.000338629	3.046731385
LINC00467	-1.300565265	1.926909485	-6.048193395	7.89184238889291e-06	0.000208939	3.812538276
CLEC18B	-1.29892167	0.462863625	-3.581572542	0.001974321	0.008781328	-1.226522297
RIBC1	-1.298699692	1.183108708	-3.925118501	0.00089998	0.004942857	-0.543304132
URB1-AS1	-1.296832094	0.835757199	-4.406395174	0.000299226	0.002270862	0.457019908
LRRC70	-1.296280887	-0.940355249	-3.975353958	0.000802147	0.0045619	-0.503289021
LAMTOR5-AS1	-1.296071034	-0.532976264	-4.310714189	0.000372284	0.002638713	0.178913155
DHRS3	-1.292995852	3.488478906	-3.570924888	0.002022832	0.008955666	-1.501521945
CDNF	-1.292196589	-0.075188547	-5.276467452	4.2170739708870e-05	0.000597621	2.081818881
EPHA5-AS1	-1.290568968	1.3549551	-5.705630859	1.64642726112963e-05	0.000324087	3.098914401
INHBC	-1.290196674	-0.450913592	-3.775601593	0.001267387	0.006352943	-0.848159385
CCDC88B	-1.290057668	2.933942527	-4.901508822	9.7404056363847e-05	0.001037481	1.45568863
MTX3	-1.289754721	5.16742876	-4.980095297	8.16431835959885e-05	0.000918901	1.408550636
LINC01125	-1.289656785	1.110502649	-6.95592007	1.2093532748186e-06	.19584894824476e-0	5.373581879
TDRP	-1.289027863	4.797889532	-9.130884823	2.1108090858547e-08	.18051180269235e-0	9.567878458
LOC100128568	-1.288874073	-0.586403793	-5.918071602	1.04172598047868e-05	0.000247776	3.092881356
TRIM7	-1.288610516	3.385583785	-5.367683462	3.44718601954602e-05	0.000518696	2.424713365
OFD1	-1.288604393	4.770073682	-11.52485699	4.74096190081057e-10	.65204569766988e-0	13.19605567
PLCH1	-1.287483835	4.579508993	-6.80347881	1.64451317258354e-06	.38405517735481e-0	5.316063635
DUSP10	-1.287273659	2.328417931	-5.001757691	7.77731554374317e-05	0.000887926	1.693443841
TFAMP1	-1.284893396	-0.594025323	-3.762697542	0.001305362	0.006477635	-0.880334194
LOC101926913	-1.28158617	-0.822325757	-3.596400325	0.001908676	0.00857496	-1.210178761
MNS1	-1.281345729	2.670019209	-3.569872668	0.00202769	0.008974996	-1.402900113
TRPC4	-1.280752294	1.96383586	-5.646017754	1.87388942704063e-05	0.000351441	3.018120666

SULT1C4	-1.280084361	2.25974938	-3.56147187	0.002066888	0.009104373	-1.380262836
CCDC14	-1.27765776	5.95360254	-3.824992483	0.00113192	0.005849162	-1.247178693
CASC2	-1.277403096	2.382610025	-6.059880962	7.6982734999972e-06	0.000205591	3.860435124
ZNF107	-1.27718194	3.104628035	-4.790164149	0.000125188	0.001253729	1.204227714
LOC100130451	-1.277021773	-1.087282875	-4.586253096	0.000198683	0.001696749	0.606681699
FAM47E	-1.276728211	0.630394392	-5.052721602	6.93877934293341e-05	0.000829402	1.749263984
YES1	-1.275851328	5.891350893	-5.807791877	1.32023663510183e-05	0.000283061	3.165721991
ATG16L2	-1.274435996	2.841882413	-8.161915263	1.18700085398915e-07	2.14452361870128e-05	7.764510971
WNT2B	-1.273180839	2.641733423	-4.054304455	0.000669431	0.004030674	-0.355337385
VWA8-AS1	-1.27225262	-1.875984952	-3.729459022	0.00140847	0.006864369	-1.066779689
NPIPB6	-1.271688351	-0.053172976	-4.49138719	0.000246531	0.001978196	0.579873137
ARHGEF26-AS1	-1.271537025	0.743762044	-6.267977164	4.9616947306844e-06	0.000155197	4.073176244
ASIC3	-1.270971072	3.677815022	-5.715392688	1.61196875125279e-05	0.000319369	3.141432568
MANEA-AS1	-1.2707684	0.861848288	-4.511425136	0.000235537	0.00191048	0.672782872
CCDC150	-1.270198745	1.327525156	-5.76236304	1.45621570244476e-05	0.000298549	3.207421502
CRABP2	-1.2694823	5.022385357	-3.96422136	0.000822868	0.004647907	-0.84903419
AGBL5-AS1	-1.269362472	-2.180038182	-3.616092479	0.001824825	0.008312738	-1.306928725
ZNF90	-1.269234167	1.208655987	-4.168373398	0.00051554	0.003330048	-0.036407865
PTPN13	-1.26793179	5.623995392	-5.799855335	1.34301792698896e-05	0.000285598	3.163592896
SCOC-AS1	-1.266927714	0.847916326	-3.636626136	0.001741275	0.008014078	-1.128012929
TBC1D32	-1.266785998	3.861899755	-6.872295514	1.43090263221107e-06	7.77640583574562e-05	5.479392586
ERBB2IP	-1.265308284	6.008291678	-5.149556349	5.59018169784269e-05	0.000723713	1.721964952
PDXDC2P	-1.264649941	4.192838579	-5.911130841	1.05733023693245e-05	0.000249625	3.51634391
CEP83	-1.264279504	2.938076296	-5.185289592	5.16284387440718e-05	0.000685889	2.061136303
CXCR4	-1.264196765	6.311896134	-5.773067007	1.4229339910799e-05	0.000295372	3.074268753
DNAH2	-1.2631628	2.304943283	-3.837902007	0.001098956	0.005732331	-0.793939863
TNNI3	-1.262512858	2.587583521	-4.483344833	0.000251087	0.002000548	0.576454584
CETN4P	-1.262188459	0.026490868	-3.781617294	0.001250061	0.00628367	-0.821820824
DCST1	-1.261344582	-0.901356576	-4.73495566	0.000141824	0.001362076	0.915753924
SMAD6	-1.260362008	0.707481808	-4.284185271	0.000395554	0.00274737	0.20466203
AXIN2	-1.259989949	5.501942828	-5.982884699	9.06945296382524e-06	0.000226948	3.562286639
BTBD19	-1.259587907	0.569746388	-4.854271314	0.000108333	0.001128234	1.350703247
ATAD5	-1.258123032	3.376674011	-7.044470253	1.01307132485495e-06	6.56233116706576e-05	5.807402914
RORA-AS1	-1.257711715	-0.379589981	-4.748755579	0.000137466	0.001334914	1.035644398
LOC101929340	-1.25661463	-2.140309692	-3.807481861	0.001178213	0.006020257	-0.971215533
C5AR1	-1.255995365	-0.628444112	-4.435602307	0.000279945	0.002169601	0.405260807
DCDC2B	-1.255261345	1.654733548	-6.142449662	6.46270517235033e-06	0.000183599	3.972440285
C21orf62-AS1	-1.254783309	0.469551728	-5.124910103	5.90579109523386e-05	0.000746991	1.874743009
DYNLRB2	-1.253018645	0.146857307	-3.993485303	0.000769511	0.004448676	-0.396085596
MOB1B	-1.252651757	6.029922275	-5.101344044	6.22455347253757e-05	0.000773463	1.613853754
BDNF-AS	-1.25240343	2.278757185	-8.26835942	9.76135723491796e-08	1.91731949043276e-05	7.839013424
LINC00515	-1.252271615	-0.701116405	-3.850328227	0.001068129	0.005618711	-0.718686391
LOC101929709	-1.251928107	1.689155149	-6.393619158	3.81617853447775e-06	0.000132277	4.452946429
DKKL1	-1.251552549	1.66131566	-4.641488976	0.000175269	0.001559489	0.947042792
WFDC3	-1.251251561	-0.422732193	-4.292449427	0.000388152	0.002713499	0.156385834
RASSF10	-1.250051662	3.248410158	-3.672609673	0.001603905	0.007551166	-1.249177989
MTBP	-1.247545208	2.278328634	-6.563759638	2.68340769778617e-06	0.000104963	4.831721102
KIAA1875	-1.247357848	1.909486812	-5.749227206	1.49815244150761e-05	0.000302941	3.222354087
LENG8	-1.243831855	7.162363677	-7.374456967	5.2853512709104e-07	4.5186005810598e-05	6.351868548
LOC100130370	-1.243133406	0.081436223	-3.686904957	0.001552364	0.007365463	-1.010082261
PROCA1	-1.242091863	2.684543629	-6.197420622	5.75492614052999e-06	0.000171079	4.143225401
SOX9-AS1	-1.241610014	1.63711711	-3.686018848	0.00155551	0.00737656	-1.068085978
POU3F2	-1.240700074	6.629601055	-6.612605432	2.42709174274838e-06	0.000100678	4.834043261
ARR3	-1.240596886	-0.619215075	-4.567750359	0.000207215	0.001747551	0.656396644
MIR3685	-1.240368578	-0.279512649	-4.00145928	0.000755581	0.004385827	-0.398192548
SCARF1	-1.239174721	1.438672866	-6.426364104	3.5650436055095e-06	0.000127193	4.482930638
PLG	-1.238851961	-1.437271564	-3.565286837	0.002048995	0.009043273	-1.310759695
EEA1	-1.238652694	5.310714673	-8.404550489	7.61667000824564e-08	1.7169760773097e-05	8.311253785
DNHD1	-1.238183013	3.96670021	-4.363828857	0.000329752	0.002432787	0.180509262
LINC01311	-1.237351335	0.201086904	-5.126001575	5.89143708614681e-05	0.0007458	1.843290833
CCND2-AS1	-1.236316315	-0.020339353	-3.558365654	0.00208157	0.009150438	-1.265278518
ZMYND12	-1.236235274	0.276294956	-4.827772024	0.000115002	0.001173594	1.272590302
USP6	-1.234611228	0.58201507	-6.356401272	4.12387117700275e-06	0.000139492	4.197438433

KLHDC9	-1.234376648	3.256624441	-3.6984532	0.001511931	0.007225404	-1.193947572
KRT10	-1.234350976	4.528418134	-4.689659082	0.000157138	0.001456339	0.832995336
LFNG	-1.233111929	4.847888444	-5.629193839	1.9437369819565e-05	0.000358649	2.85816148
ANG	-1.232487966	0.278760809	-4.161824845	0.000523327	0.003367245	-0.054050962
NKAPP1	-1.229946006	-0.651136575	-4.232563149	0.000445115	0.002991869	0.016541939
GPM6B	-1.227994689	9.166569614	-4.576274656	0.000203239	0.001729177	0.406025202
MASP1	-1.226876141	7.210055314	-4.769464936	0.00013118	0.001291786	0.842457003
LOC102724814	-1.225383479	1.550626646	-6.308294297	4.55985380110637e-06	0.000146646	4.276681768
DPY19L2P4	-1.225331932	1.544370607	-6.164139259	6.17331225195266e-06	0.000178148	4.002998945
ROCK1P1	-1.222602008	1.782292866	-6.331996132	4.3394097764419e-06	0.000143847	4.346789409
SMC1B	-1.221510395	-1.964370946	-4.831987811	0.000113913	0.001166399	0.808264178
TMEM106A	-1.221029506	-0.21277719	-3.699123879	0.001509615	0.007218882	-0.989232254
GVINP1	-1.220855815	-0.473410098	-4.018135992	0.000727259	0.004264795	-0.378571542
ASCL1	-1.220744373	4.789225156	-5.575562512	2.18472732753884e-05	0.000381899	2.747905179
AFF1	-1.220156663	4.663359828	-5.251073994	4.461175097111e-05	0.000616898	2.055762786
CTSV	-1.219990218	1.102900743	-4.609286451	0.000188557	0.00163163	0.878829639
LOC101928222	-1.219450469	0.286492134	-4.385037478	0.000314169	0.002351055	0.394638848
CASC1	-1.219285392	1.723625692	-5.957871765	9.56701129049942e-06	0.000234578	3.622044954
COL4A5	-1.219098187	5.892506717	-3.846521279	0.001077481	0.005649351	-1.196146532
PNISR	-1.21628165	7.435520778	-8.785074122	3.85655139731414e-08	1.22666735911394e-05	8.959769107
SPATA6L	-1.216261872	1.526912038	-6.061133432	7.67782174570345e-06	0.000205345	3.804410378
USH2A	-1.215755452	-1.066973546	-3.644229352	0.00171131	0.007914049	-1.135197448
LINC01604	-1.214913031	2.064333889	-6.412955826	3.66575249788654e-06	0.000129271	4.527162614
POLI	-1.212875876	4.840870447	-7.131486633	8.52133172899271e-07	5.82993133683558e-05	5.948075876
POU2F2	-1.212540547	5.364564245	-4.908109989	9.59686318743803e-05	0.001029158	1.228889713
JAG1	-1.210088335	5.605989469	-5.179564934	5.22899680929889e-05	0.00069216	1.811374031
FSTL5	-1.210043877	6.082978806	-5.478563243	2.70122985834806e-05	0.000442042	2.442350746
ACVR2B	-1.209955784	6.547772499	-6.625821921	2.36217835788193e-06	0.000100583	4.862426481
LUZP2	-1.209605877	3.886427155	-4.08538198	0.000623435	0.003840908	-0.426635426
LRRIQ1	-1.205350208	3.956104942	-5.686189553	1.71732069035964e-05	0.00033161	3.060216559
TTC32	-1.204779566	2.910547915	-5.514109562	2.49881466876602e-05	0.000420312	2.75366426
PI4KAP1	-1.204394314	4.15236251	-3.655732165	0.001666944	0.007775808	-1.413628994
STAG3L5P	-1.203263357	-1.00458832	-4.461480415	0.00026391	0.002076163	0.395125619
EGFEM1P	-1.203259288	2.151203712	-4.948495657	8.76428564440985e-05	0.000964655	1.584747417
CDC144NL-AS	-1.202996663	3.989104349	-6.404556434	3.73032902892094e-06	0.000131042	4.545560515
UVSSA	-1.201899336	4.705520349	-5.243108073	4.54068430369942e-05	0.000625998	2.033791535
MTM1	-1.200978458	4.274762961	-5.892558195	1.10027659422316e-05	0.000254093	3.470015976
NXT2	-1.199372637	3.233973421	-4.456244993	0.000267078	0.00209837	0.466164977
IQUB	-1.199190329	0.367336476	-4.971405475	8.32501842616733e-05	0.000928608	1.564382693
DRC7	-1.198886432	0.647938737	-3.710023294	0.001472469	0.00709699	-0.971093693
CNTRL	-1.198102173	4.406285232	-3.874487486	0.001010641	0.005390185	-0.968611644
MTTP	-1.197732651	1.957458986	-4.856362074	0.000107824	0.001124855	1.394106406
RFESD	-1.197276059	-0.35469016	-4.652948902	0.000170772	0.001537451	0.857876887
STXBP3	-1.197274504	4.976166272	-7.240086363	6.87643069400784e-07	5.20340974089323e-05	6.15254399
AHCTF1P1	-1.19723488	-1.054745147	-4.1994365	0.000480161	0.00315642	-0.097614683
LINC00969	-1.197050455	1.479739605	-5.098971045	6.25760792095568e-05	0.000775493	1.8927595
SNORD115-46	-1.196358559	0.707383511	-5.396415263	3.23569290612308e-05	0.000497052	2.431508255
SAR1B	-1.196130841	5.220633078	-7.403574787	4.99398538896052e-07	4.41953518348406e-05	6.457124896
C1orf194	-1.196101491	1.721129712	-4.39974767	0.000303799	0.002301432	0.436957856
LOC155060	-1.195789718	4.318280936	-7.752956016	2.55165134496856e-07	3.19253528209182e-05	7.145148416
DNAH6	-1.195453323	2.933824237	-5.380314163	3.35252451271172e-05	0.000509065	2.472936106
NR3C2	-1.195250445	2.705649216	-4.324170749	0.000361012	0.002585237	0.223839505
TMEM9B-AS1	-1.195209958	2.134459778	-4.539363673	0.00022103	0.001824474	0.719168934
LYPLAL1-AS1	-1.194425915	-0.831358734	-3.92694071	0.000896232	0.004931163	-0.582981745
ZNF69	-1.194135722	2.161951112	-4.385269581	0.000314003	0.002351055	0.389104068
C1orf228	-1.194095173	1.786598985	-5.934461749	1.00580914214312e-05	0.00024189	3.581023335
GABPB1-AS1	-1.193413598	4.479783401	-5.043119389	7.08941705214107e-05	0.00083929	1.619740018
DNMT3B	-1.192802024	4.146128344	-6.757945766	1.8037299992586e-06	8.79880344679625e-05	5.246248747
ZNF493	-1.191378912	4.733271798	-5.033075928	7.25054184021923e-05	0.000850201	1.568936765
LOC102546294	-1.190943026	1.022172857	-4.841649404	0.000111459	0.001151302	1.352252148
MAP3K8	-1.190724193	2.527493762	-6.304463612	4.59655011827304e-06	0.000147307	4.348126303
LOC100270746	-1.188629541	-0.287422715	-4.344310862	0.000344781	0.00250621	0.271260543
LINC01004	-1.187251568	2.881986895	-4.620583361	0.000183783	0.001608607	0.853025353

TOX3	-1.186955247	5.082161054	-5.01289754	7.58561989298982e-05	0.000872187	1.487015587
TIGD4	-1.186865902	1.394146456	-6.898572622	1.3570926246439e-06	7.63821619863328e-0	5.333469171
ANP32E	-1.186230097	6.347425668	-3.71401833	0.001459082	0.007039899	-1.515733516
CAHM	-1.186076993	1.28532386	-4.614228214	0.000186453	0.001624198	0.89116909
TRHDE	-1.18602901	0.717606827	-3.59020115	0.001935854	0.008652371	-1.218146422
FLJ35934	-1.184311583	-0.375209151	-5.213972762	4.84401223345546e-05	0.000656421	1.907262248
ZNF624	-1.183193149	3.680341573	-10.31703908	2.97597425790648e-09	2.96809701325713e-0	11.30015322
MIR570	-1.18282387	-0.41896004	-3.805936605	0.001182388	0.006031465	-0.787145272
HLA-J	-1.181891966	0.692207588	-5.169452269	5.34797630784724e-05	0.000700297	1.986383423
RAD9B	-1.179412825	-0.196028655	-4.081165444	0.000629485	0.003867745	-0.236422846
MEGF6	-1.178470709	5.140915379	-5.228723236	4.6879444227969e-05	0.000638112	1.95773131
PRICKLE4	-1.178316844	3.040709498	-7.175344175	7.81282524629156e-07	5.53135291921259e-0	6.037102892
ZNF337-AS1	-1.177905166	0.341407147	-5.989812216	8.93640804948781e-06	0.000225161	3.48552283
TRHDE-AS1	-1.176377531	0.875154499	-3.964002449	0.000823281	0.004648801	-0.454465415
LCA5	-1.176002044	3.794227979	-8.760285	4.02912583055982e-08	.22666735911394e-0	8.892527653
ZNF404	-1.175662531	3.953883363	-7.881452942	2.00148211703651e-07	2.85523376272088e-0	7.375582626
WDR93	-1.175114429	0.099198409	-4.123111937	0.000571829	0.003592019	-0.13807418
NOXRED1	-1.175030843	0.39950372	-5.358651265	3.51654947325897e-05	0.0005261	2.318890634
C6orf25	-1.174150077	1.383202629	-4.08846962	0.000619042	0.003820284	-0.208731074
PARD6B	-1.174102809	2.858255183	-5.648292555	1.86464451854625e-05	0.000350787	3.033128011
C3orf58	-1.173771456	5.332078096	-5.584692353	2.14163847312801e-05	0.000376201	2.718326993
LINC00368	-1.173708013	-0.748553211	-4.386444316	0.000313162	0.002350259	0.296422076
LOC101928283	-1.172312192	-1.714279992	-4.079693546	0.000631611	0.0038746	-0.421078382
MIR99AHG	-1.171998316	4.094392546	-7.574030569	3.59152661502002e-07	3.93097280448821e-0	6.814310582
VN1R1	-1.170363323	1.665758106	-5.472694767	2.736226741562e-05	0.000444483	2.657516472
BTAF1	-1.170197801	6.534572212	-5.82055736	1.28442250092142e-05	0.000276681	3.169715877
LINC00471	-1.169602963	1.335836788	-5.973137563	9.26010410243086e-06	0.000230305	3.616320808
SERINC4	-1.169254565	2.4953128	-4.618405996	0.000184693	0.001613016	0.871765646
MYLK-AS1	-1.168984461	0.984880694	-6.402857801	3.74353039180366e-06	0.000131253	4.367858788
GUSBP2	-1.1684601	0.3299544	-3.951083844	0.00084801	0.004742987	-0.477921852
NPHP3	-1.166235448	2.053575562	-5.859422695	1.18139935196957e-05	0.000262538	3.448802903
MIR124-2	-1.165775996	1.531964012	-4.470754297	0.000258393	0.002041547	0.590653843
NSUN6	-1.165666271	4.032948763	-5.158512191	5.47980220273127e-05	0.00071296	1.920357038
CBR4	-1.165572453	4.780699381	-5.985102414	9.02664110355877e-06	0.000226606	3.621064461
PCDHA7	-1.164790188	0.158902822	-3.620165332	0.001807943	0.008258489	-1.14365978
EPHA3	-1.164503213	5.629488391	-7.14453825	8.30379591846595e-07	5.74682417171163e-0	5.93350844
SEMA3B-AS1	-1.163912065	0.063673107	-4.330289625	0.000356001	0.002556237	0.272448341
TOB1	-1.163053712	5.327255969	-8.298468401	9.23847052703133e-08	.89617012491327e-0	8.12019023
ASMTL-AS1	-1.162188919	2.307902322	-6.456336298	3.35010245311667e-06	0.000122202	4.629425878
ZNF37BP	-1.161771662	5.205799687	-5.860899428	1.17765644704436e-05	0.000262025	3.322152804
CEP44	-1.161509945	4.221918631	-6.026640756	8.26202192655378e-06	0.000215296	3.754407631
PARD3B	-1.161094178	5.234885555	-4.378046355	0.000319222	0.002376163	0.052259918
EOGT	-1.160669346	1.824999162	-4.169915035	0.000513724	0.003324195	-0.054261975
LINC00342	-1.160342187	4.48093257	-7.460831662	4.46855171531822e-07	4.21728040504825e-0	6.596335487
L3MBTL1	-1.159629459	4.493090352	-4.727218585	0.000144329	0.001376738	0.919387685
NKTR	-1.159500887	6.879527876	-5.783831198	1.39025127355182e-05	0.000292089	3.08413032
CDO1	-1.159336813	6.246501356	-7.452402982	4.54215570133938e-07	4.21728040504825e-0	6.514774305
HCG25	-1.158656937	-1.044842209	-4.105285804	0.000595654	0.003703478	-0.27080734
TMEM123	-1.156272441	5.947353501	-4.052301794	0.000672509	0.004046402	-0.738564358
WEE2	-1.152225689	-1.142987768	-3.600099009	0.001892641	0.008536414	-1.22257649
UFL1	-1.150993558	5.052803458	-6.618056863	2.40009662346208e-06	0.000100674	4.911113936
STAM-AS1	-1.150769926	0.080338526	-3.618428676	0.001815123	0.008282999	-1.146546357
NSUN5P2	-1.150411444	1.841022954	-6.231704223	5.35436617568254e-06	0.000163512	4.161597382
PIGL	-1.150180319	3.124619178	-7.683993196	2.90951365355008e-07	3.45131166877267e-0	6.969885885
FRA10AC1	-1.148825991	5.049416439	-8.200896039	1.10476619802512e-07	2.07646305696619e-0	7.950837489
KRTCAP3	-1.148353103	0.4875569	-3.980961719	0.000791908	0.004531887	-0.41631063
CARD14	-1.148313977	1.900838167	-6.834005386	1.54595947753122e-06	8.06858336458935e-0	5.296417542
LEKR1	-1.146845321	-0.200440693	-4.81263126	0.000118997	0.001208966	1.185990735
LOC101929524	-1.145871264	-0.442938264	-4.346688943	0.000342913	0.002496811	0.25989124
METTL21B	-1.1441305	2.188055837	-5.089703424	6.38842250099168e-05	0.000786242	1.88009212
KLF3-AS1	-1.143895331	3.501928427	-6.018291248	8.41018404767548e-06	0.000217297	3.778432655
RPGRIP1	-1.14312138	-0.737219441	-3.85359151	0.001060178	0.00558911	-0.714443045
LAT	-1.142951642	1.79966714	-4.569918367	0.000206196	0.001744599	0.794127957

ABCG1	-1.142381439	4.195541593	-4.005442843	0.000748717	0.004361228	-0.648432939
HCG23	-1.14176626	-1.685708356	-3.588441686	0.001943637	0.008676992	-1.292509387
LOC389705	-1.141056364	1.32569129	-4.139910187	0.000550252	0.0034913	-0.099097426
HIF3A	-1.140500683	4.374318788	-6.136616476	6.54289029740102e-06	0.000185301	3.971358712
LOC101927045	-1.140345676	1.077113824	-4.719291923	0.000146941	0.001389322	1.104494759
HEMK1	-1.140061117	4.491661486	-6.674693309	2.13729781951561e-06	9.476218269197e-05	5.062184325
UGGT2	-1.139475573	4.652295209	-6.765030122	1.7779486878936e-06	3.76046722960242e-0	5.233245562
RBBP6	-1.139103015	6.726372493	-9.734139962	7.63661688328653e-09	4.81027864162052e-0	10.5646637
ARHGAP15	-1.138321874	0.267582389	-3.950008266	0.000850102	0.004748872	-0.480786657
STK31	-1.137252923	-0.340099783	-3.944092374	0.000861701	0.004794607	-0.5134468
SPATA6	-1.134912615	4.366921177	-5.051194738	6.96251282247408e-05	0.000831269	1.648964971
CAPN10-AS1	-1.134431492	1.917140792	-5.191707081	5.08969854951346e-05	0.000678133	2.091885291
LOC388849	-1.133510228	0.87227803	-5.165002336	5.40120263231493e-05	0.000704741	1.993997751
LUC7L3	-1.133246236	8.214827942	-6.499063707	3.06652913729451e-06	0.000114319	4.590885445
C17orf75	-1.131783177	4.853821646	-7.126743173	8.6018459043053e-07	5.8412609343474e-05	5.93731574
LOC100996447	-1.131661625	1.427508167	-4.808940788	0.000119992	0.001217722	1.295620875
ITGB7	-1.131037895	-0.923811217	-4.007805188	0.000744676	0.004343581	-0.438830774
YPEL4	-1.131029191	3.547379355	-4.665474823	0.00016599	0.001508528	0.892703824
LOC100129027	-1.129232861	-0.305495784	-3.766663391	0.001293572	0.006442115	-0.859059839
SNX20	-1.129208039	-0.95901198	-3.769374049	0.001285574	0.006414081	-0.891448411
EAF2	-1.127504398	1.383010521	-3.779663941	0.001255661	0.006304879	-0.857128581
WNT3	-1.127148554	4.366864036	-5.88038667	1.12938907523907e-05	0.000256599	3.435755282
LOC101929095	-1.126700903	-1.217142435	-3.805396801	0.001183849	0.006035182	-0.848027967
PON2	-1.126479258	5.964723556	-4.768668059	0.000131416	0.00129272	0.872808251
NPIPB9	-1.125720861	-0.568145983	-3.914057099	0.000923075	0.005035931	-0.585941391
SDHAF3	-1.125629513	3.124335469	-7.183989269	7.68051306145672e-07	5.52361937376496e-0	6.058632086
TRIM22	-1.125115689	3.372517877	-3.949362104	0.000851361	0.004752509	-0.6612322
ZNF846	-1.124366982	2.667418619	-4.542333598	0.000219542	0.001815793	0.698015443
SUCLG2	-1.123929909	3.929124346	-5.802560676	1.3352074744413e-05	0.0002846	3.306706228
ST8SIA4	-1.123334874	5.01110611	-9.948303645	5.37769337148919e-09	4.30882913325477e-0	10.89909855
ZFYVE16	-1.122778777	5.746387854	-5.959336196	9.5371222310453e-06	0.000234479	3.495109638
MAK	-1.120533903	1.43005265	-6.041457565	3.00566647183956e-06	0.000211329	3.757703658
IFT81	-1.118482244	5.377004792	-6.632312758	2.33095680502203e-06	.99831561127921e-0	4.919254484
MYH7B	-1.117378909	2.176413579	-3.855195613	0.001056291	0.005573195	-0.747580678
SLC35A3	-1.1165165	4.242894541	-7.209701723	7.30051905746216e-07	5.35613094958005e-0	6.123828583
PUS10	-1.114462225	2.163784797	-6.866530468	1.44764370080486e-06	7.77640583574562e-0	5.387968474
PILRA	-1.114224173	1.471498933	-3.602877115	0.001880685	0.008497271	-1.232307318
LOC441204	-1.113835457	3.311446997	-4.688515698	0.000157546	0.00145719	0.965590616
RND3	-1.109538613	7.105247472	-6.632161099	2.33168142026876e-06	.99831561127921e-0	4.867544476
ACSL6	-1.109448304	5.712821021	-7.194559001	7.52188852211602e-07	5.45247371561482e-0	6.027805852
RHD	-1.108710665	-0.067394608	-5.639952444	1.89876936001512e-05	0.000353393	2.757978792
ALDH8A1	-1.107340995	0.479746511	-3.599995813	0.001893087	0.008536414	-1.190491151
CRYBG3	-1.106555906	3.572590163	-3.821451994	0.001141132	0.005888433	-0.96794171
PNN	-1.105192748	7.346976907	-5.26541745	4.32157431666387e-05	0.00060585	1.946894486
NUP62CL	-1.104209908	-0.049785819	-4.335684234	0.000351641	0.002532897	0.275598087
B3GNT5	-1.103891894	4.75292762	-4.230248182	0.000447478	0.002998562	-0.224812161
ZNF460	-1.103782031	2.896939514	-3.855271773	0.001056106	0.005573195	-0.812307042
LOC101927056	-1.102090174	-0.679692489	-3.861081713	0.001042149	0.005514755	-0.695692228
CORO6	-1.101949141	1.907254369	-4.232509991	0.000445169	0.002991869	0.074887881
TRIM5	-1.101582429	3.406960249	-4.781577181	0.000127639	0.001268543	1.159599053
FILIP1	-1.100939063	2.673243186	-4.075251231	0.00063807	0.00390212	-0.313590946
DDX11L2	-1.099139115	1.401956586	-4.268662098	0.000409844	0.002812402	0.168956335
PITPNA-AS1	-1.099006638	2.363302815	-6.018830826	3.40052721835412e-06	0.000217297	3.778718252
CDK5RAP3	-1.098117417	6.708833899	-6.897888176	1.35896439730433e-06	7.63821619863328e-0	5.411684042
XPNPEP3	-1.097895887	4.253058743	-4.815448901	0.000118243	0.001202203	1.142702201
TAS2R19	-1.097717826	-0.389724768	-4.322214179	0.000362629	0.002593637	0.218894521
CENPC	-1.097116107	3.521560783	-6.707755492	1.99779417899361e-06	9.1814003257369e-05	5.160726329
LOC440600	-1.096901844	-1.379173077	-3.67811844	0.001583845	0.007485666	-1.098379447
ZNF121	-1.096533397	3.569939111	-4.155566288	0.000530878	0.003401458	-0.232340194
SCAF11	-1.09634491	6.073484575	-7.527228355	3.93024552892681e-07	4.02448969805022e-0	6.66252406
OCLN	-1.096198641	1.618132143	-3.533863247	0.00220106	0.009536706	-1.386843484
ZNF780B	-1.093802883	5.014608114	-5.877926889	1.13536792152608e-05	0.000256998	3.372639632
NMU	-1.093171386	2.328808655	-4.223157397	0.000454796	0.00303757	0.0316473

LOC10013331	-1.092589246	0.280645655	-4.808182738	0.000120198	0.001218453	1.236171866
FAM89A	-1.092304543	3.298234599	-3.976205174	0.000800584	0.004555849	-0.593748177
PDCD4	-1.092249704	5.766570583	-6.875167431	1.42263765508413e-06	7.77640583574562e-0	5.390768017
STARD6	-1.091358008	-1.648133968	-3.996809967	0.000763672	0.004424355	-0.556260067
SPICE1	-1.091129262	3.903314974	-6.973528571	1.16740649453967e-06	7.07341730256971e-0	5.675248908
SMC5-AS1	-1.090686403	0.284998419	-4.444526429	0.000274307	0.002140287	0.514496532
PDLIM5	-1.090306881	5.53498336	-4.3559333	0.00033575	0.002464102	-0.026888549
DSG2	-1.087564707	3.607798943	-4.918705669	9.37093995104076e-05	0.001014093	1.439550881
LOC10272462	-1.087397721	1.327554679	-3.593052231	0.001923307	0.008622402	-1.24398103
SH3BP5-AS1	-1.08656775	3.704216137	-5.145807627	5.63705459310602e-05	0.000728631	1.923499044
SKIDA1	-1.086303896	3.822839397	-5.291003985	4.08351763027826e-05	0.000583675	2.226273717
GRIK1-AS1	-1.086047338	-1.922849013	-4.099157254	0.000604073	0.003752826	-0.424441365
CA14	-1.085237832	4.46294774	-3.852971918	0.001061683	0.005593816	-1.027434237
SNORD52	-1.08517761	-1.490875522	-3.534622651	0.002197256	0.009524747	-1.37010325
USP45	-1.085111528	3.243606314	-6.869713512	1.43837561922792e-06	7.77640583574562e-0	5.470461323
TNFRSF25	-1.084373498	3.843469129	-4.039851961	0.000691965	0.004129408	-0.523639093
NKAP	-1.084267237	4.369032012	-7.093915204	9.18106840928894e-07	6.10105768363804e-0	5.895862468
NRXN3	-1.084083936	5.575151048	-4.487657376	0.000248633	0.001990266	0.26634997
HLTF	-1.084009756	6.626484394	-5.995062361	8.83691436402718e-06	0.000222961	3.540757343
LOC643072	-1.083456389	2.156718811	-6.334347859	4.3181440840715e-06	0.000143679	4.384576106
LINC00910	-1.082800449	-0.556632936	-3.749052597	0.001346749	0.006629227	-0.903643978
TSPAN12	-1.079430551	2.376772036	-5.511188316	2.51484799655336e-05	0.000422231	2.753201998
UNC80	-1.079157021	4.886928481	-5.756056212	1.4761981825678e-05	0.000301293	3.123615802
ANKRD31	-1.079146625	0.086088819	-4.201187791	0.000478241	0.003146985	0.017594091
LRRCC1	-1.078906567	4.140060539	-6.629224028	2.34576045282266e-06	0.000100117	4.989667831
SLC24A1	-1.078220913	3.064205657	-7.500187933	4.14087265365154e-07	4.10308567566517e-0	6.634674256
NPIPB15	-1.076669568	1.090714023	-3.699800997	0.00150728	0.007213812	-1.009434723
RTCA-AS1	-1.073926256	0.540892824	-3.987131827	0.000780793	0.004492203	-0.40366168
MPHOSPH10	-1.072974823	5.334178799	-6.905837632	1.3373882219702e-06	7.60515146250772e-0	5.473162787
EDA2R	-1.072766663	4.509543069	-4.594994922	0.000194777	0.001667397	0.621002941
MIR181A2HG	-1.070831277	1.07248458	-5.107115303	6.14490364807235e-05	0.000766204	1.892367569
BL5P-PVRIG2P	-1.070259291	4.262235405	-4.878764391	0.000102519	0.001081245	1.28089561
DDX12P	-1.069831475	2.340160888	-4.281673879	0.000397831	0.002757586	0.156668435
TYMSOS	-1.069614135	0.852882751	-5.112044449	6.07769843002512e-05	0.000761463	1.887759119
NBR2	-1.069287428	2.351118961	-4.62547945	0.000181752	0.001596953	0.893560032
TTC26	-1.068484695	3.303685242	-10.39159809	2.64522231907373e-09	1.84236918250117e-0	11.31603093
RMDN2	-1.067809731	1.240042129	-5.419761541	3.07363468810913e-05	0.000481115	2.528203583
C7orf31	-1.067109938	2.835427766	-6.651097989	2.24297512454483e-06	9.84914100962992e-0	5.034956336
OPN1SW	-1.06576523	-1.409516233	-3.83046338	0.001117832	0.005804273	-0.822997207
TAS2R31	-1.065445251	-0.272891653	-3.912174341	0.000927065	0.005049103	-0.572070636
ZNF138	-1.065323015	4.641123125	-6.957796456	1.20481015275048e-06	7.19584894824476e-0	5.616148007
ZC3H12C	-1.065124957	5.365050991	-6.9710205	1.17328813854416e-06	7.07341730256971e-0	5.60131335
RAB11B-AS1	-1.064814349	1.703426356	-6.327908008	4.37663407215051e-06	0.000144291	4.332160113
SNHG21	-1.06481374	1.923354057	-5.060166007	6.82423843412251e-05	0.000822285	1.819688454
PABPC1L	-1.064502933	2.532001575	-4.393584598	0.000308101	0.002323919	0.385580814
C8orf37	-1.064428423	3.357596613	-7.687694328	2.88904512245881e-07	3.45131166877267e-0	6.996309194
CKMT2-AS1	-1.064165439	2.657258544	-4.485622474	0.000249788	0.001995139	0.576073551
PRDM5	-1.062623293	2.510748429	-5.832630786	1.25146702111438e-05	0.000271181	3.409679423
SASS6	-1.062398246	3.070301121	-5.687809912	1.71129426200693e-05	0.000331146	3.110244701
SCX	-1.062392528	0.606721319	-3.952953116	0.000844386	0.004724166	-0.473760405
GLIPR1L2	-1.062292709	1.200594935	-4.785516036	0.000126509	0.00126332	1.243573518
MTR	-1.061482121	5.510915204	-4.975926922	8.24100615831253e-05	0.000924116	1.364120269
INSIG2	-1.061016417	5.555938966	-5.972805816	9.26666495403855e-06	0.000230305	3.534061877
RNF139-AS1	-1.060521257	1.739206541	-5.444037934	2.91390415188218e-05	0.000464876	2.603092477
COL11A2	-1.059451848	4.661831206	-3.527967634	0.002230807	0.009631329	-1.776318818
FAM83H-AS1	-1.0594342	0.482419113	-3.754624092	0.001329694	0.006561191	-0.876918312
C1orf54	-1.057932009	3.490738543	-5.477360323	2.70836602035473e-05	0.000442042	2.649811124
ADAM9	-1.057028304	6.16061292	-4.135552099	0.00055577	0.00351654	-0.563170453
TMF1	-1.055562305	6.025080154	-7.815994645	2.26443649717163e-07	3.01930375867402e-0	7.212401197
SLFN5	-1.055130352	4.114477378	-3.633913702	0.001752091	0.008056918	-1.459431476
CCDC108	-1.052324448	1.625847393	-3.889612209	0.000976233	0.005260426	-0.638932245
SPEF2	-1.051277097	3.074581869	-4.514481245	0.000233904	0.001901527	0.606850894
LOC285847	-1.051008467	0.569939014	-3.729146537	0.001409477	0.006865843	-0.930531815

TMEM47	-1.049257729	6.629470777	-6.66243059	2.19156230901299e-06	.66987166636239e-0	4.934771784
SRRM2-AS1	-1.048176445	-0.532886425	-4.378680374	0.00031876	0.002373754	0.311735057
REST	-1.047934426	4.238802019	-5.01506137	7.54894578684979e-05	0.000871536	1.582885946
SENP7	-1.046815347	5.878600817	-5.07560813	6.59274289943554e-05	0.000802864	1.56132639
LOC101927027	-1.045668206	3.303907426	-6.000305562	8.73868779191229e-06	0.000221708	3.747543414
TYW5	-1.045365672	3.929248967	-7.330294862	5.76128207225981e-07	.71934258358609e-0	6.357508195
SUGCT	-1.045212434	1.407638749	-4.240533014	0.000437074	0.002948311	0.109642247
EVI5	-1.044987815	4.377057613	-5.750066061	1.49543770447346e-05	0.000302941	3.157542949
FAM35DP	-1.043382434	-0.360030642	-3.771828161	0.001278376	0.006394332	-0.850595804
GLIPR1	-1.042631345	1.802508749	-5.496495479	2.59710082884264e-05	0.000432262	2.711464427
ZNF680	-1.042252948	3.849651445	-3.544082749	0.00215042	0.009383859	-1.615626193
CBX3P2	-1.042241167	1.936609647	-7.402116179	5.00817749483331e-07	.41953518348406e-0	6.316490985
C19orf81	-1.041588774	2.735842249	-4.372149319	0.000323548	0.002398639	0.324885164
SMC5	-1.041455309	5.601728567	-5.01552968	7.54103231717015e-05	0.000871536	1.44517654
LOC202181	-1.040585076	2.807427106	-6.685200563	2.09190515385682e-06	.38516759032394e-0	5.099126959
TET1	-1.038995597	5.023644233	-4.842167474	0.000111329	0.001151302	1.110458384
RNPC3	-1.038653537	4.251388891	-10.54700874	2.07323966842852e-09	.78394289080403e-0	11.74355645
TMEM147-AS1	-1.038518744	2.615259589	-4.796363697	0.000123447	0.001241748	1.246604571
TAS2R5	-1.038494565	0.063892995	-4.400642276	0.000303179	0.002297998	0.412931282
ZSCAN16-AS1	-1.038419044	2.695441428	-6.956297739	1.20843743702156e-06	.19584894824476e-0	5.604962263
SACS-AS1	-1.038260755	-0.855619488	-4.05571381	0.000667274	0.004023321	-0.340260915
KLRAP1	-1.038151282	2.077227892	-3.942171305	0.000865502	0.004809893	-0.554822475
SPACA6P	-1.037036442	2.237904429	-5.701548206	1.66106179385777e-05	0.000326469	3.139176077
CCDC146	-1.036672951	2.903818114	-5.378620908	3.36505892946815e-05	0.000510002	2.470077218
EFHC2	-1.035725612	1.553376624	-3.593422117	0.001921685	0.008618568	-1.257979755
BTG2	-1.033960891	5.726540203	-6.682384194	2.1039737443895e-06	.39689202659244e-0	5.001904843
RAP2C	-1.033868592	5.194522877	-4.948396924	8.7662288891486e-05	0.000964655	1.329662775
ARGLU1	-1.033202277	7.220380459	-3.519109764	0.002276249	0.009769795	-1.968947477
NADK2	-1.031940774	6.083140351	-4.486165189	0.00024948	0.001993546	0.230623552
BAZ2B	-1.031797313	6.337363415	-11.30965638	6.50581191240413e-10	.65204569766988e-0	12.97565707
FAM181A-AS1	-1.031756916	0.786860254	-4.424988808	0.000286802	0.00220869	0.494834168
ABCC2	-1.03139423	-0.519840405	-3.542103488	0.002160137	0.009412983	-1.301792447
C5	-1.031321793	3.071688568	-5.312702164	3.89214757655439e-05	0.000565166	2.324288274
FBXO36	-1.029515604	3.35547482	-7.934192731	1.81275569217617e-07	.71323943698384e-0	7.435378311
EXPH5	-1.029512326	0.333784461	-4.010095926	0.000740778	0.004328548	-0.358344158
LOC100507547	-1.029459814	0.480841073	-4.222275151	0.000455715	0.003039262	0.07458562
TGDS	-1.029255054	2.530058547	-4.183003341	0.000498563	0.003253751	-0.069191872
P4HA1	-1.028992218	6.976630053	-4.997568423	7.85067400681403e-05	0.000892011	1.354613385
WDR90	-1.028963068	4.471395415	-5.539030953	2.36621463662677e-05	0.000403582	2.696890348
REEP3	-1.028937438	4.473736301	-4.018779325	0.000726188	0.00426125	-0.661409772
IZUMO4	-1.027983559	2.000299465	-6.508581842	3.00680164410563e-06	0.000112783	4.703842856
ZNF217	-1.027038311	4.069538222	-4.73006521	0.000143402	0.001370763	0.975230133
TTC8	-1.026904378	4.451034584	-6.942064769	1.24345270035585e-06	.35085775967649e-0	5.59473333
RIN2	-1.026824809	3.519358765	-4.036502301	0.000697295	0.004146316	-0.488927685
JRKL	-1.026320658	4.19597012	-4.942905198	8.87500824130979e-05	0.000973108	1.429016726
FGF2	-1.026306626	4.376087349	-6.398923968	3.77428833966247e-06	0.000132078	4.509988846
PLAGL1	-1.025907941	4.943314981	-6.824139564	1.57712557455294e-06	.13823527411256e-0	5.332043974
RHBDL1	-1.025788177	3.02076734	-4.435943765	0.000279727	0.002168833	0.440388003
ENOSF1	-1.025611	3.121211065	-5.320726777	3.8237150115117e-05	0.000559527	2.338854183
GFPT2	-1.02543806	4.112108975	-7.167589817	7.93351105728197e-07	.53135291921259e-0	6.045787941
LOC283710	-1.024995214	-0.669595838	-3.775186697	0.00126859	0.006355826	-0.859371154
PDIA2	-1.024638251	3.980096141	-4.143867014	0.00054529	0.003468416	-0.312527544
TAS2R20	-1.023385933	-0.135492785	-3.526969332	0.002235883	0.009646406	-1.326943236
RRM2B	-1.023286812	5.896214621	-3.911287539	0.00092895	0.005057861	-1.054630412
C6orf52	-1.022650687	1.217958588	-3.658926219	0.001654828	0.007742749	-1.100913935
AMH	-1.022609009	4.794773625	-5.142391589	5.68011653719088e-05	0.000731262	1.799310043
NSUN5P1	-1.022418191	3.869295363	-7.388536019	5.14233302698724e-07	4.4922487146361e-05	6.466815232
LOC100129203	-1.022198904	-0.607284655	-3.996646356	0.000763958	0.004424611	-0.429162707
PBLD	-1.021787061	3.10621847	-5.613522315	2.01119936115326e-05	0.000363279	2.954703188
ACKR3	-1.021649153	3.677606005	-5.840634418	1.23009978332505e-05	0.000267822	3.402165203
MRPS31P5	-1.021235629	4.256372686	-6.371552362	3.99563859887716e-06	0.000136682	4.462082466
MIR186	-1.020311276	0.63672615	-5.902896646	1.07615408281315e-05	0.00025223	3.387859257
NPHP1	-1.019594395	2.488857736	-5.56886239	2.21691289965008e-05	0.000385313	2.872015236

INTS2	-1.019497265	4.247085304	-5.63742276	1.90924591041784e-05	0.000354433	2.929520919
EPX	-1.019345859	1.857038707	-4.03061223	0.000706767	0.00418213	-0.352654337
GCC2	-1.019181858	5.172256182	-11.15915002	3.13949268025889e-10	.65204569766988e-06	12.7256674
BROX	-1.019013759	5.131383802	-4.786580706	0.000126205	0.00126115	0.974901704
CHD7	-1.018461438	7.331659235	-5.897298629	1.08914765391133e-05	0.000253703	3.323732511
COG6	-1.017947088	4.939402339	-7.39065079	5.12119943475235e-07	4.4922487146361e-05	6.443270486
ZNF273	-1.016346742	3.219216063	-5.143702871	5.66354724220739e-05	0.000730622	1.956263645
CHD1	-1.016049915	5.552707386	-5.232535135	4.64845311934436e-05	0.000635103	1.928803092
ESCO1	-1.015611167	4.47816842	-7.533341518	3.88418574940453e-07	.00861136069901e-05	6.732651492
ACADVL	-1.015548615	6.775337825	-8.592365024	5.43102903699958e-08	1.3778973252621e-05	8.621775354
SKIL	-1.015441488	5.384877942	-5.118137905	5.9956526827166e-05	0.000753769	1.688895507
ARMC4	-1.015310434	1.522546672	-3.95976809	0.000831306	0.004682536	-0.485778348
C5orf34	-1.015076951	2.355458821	-4.453048531	0.00026903	0.00210827	0.523853307
PAXBP1	-1.013630421	5.739610915	-3.814434383	0.001159611	0.00595545	-1.261414974
FANCM	-1.013553358	2.617247654	-4.1139683	0.000583928	0.003649199	-0.225469343
TGM1	-1.013031697	1.840471975	-4.143693725	0.000545506	0.003468416	-0.111198369
PPP1R3B	-1.011646672	2.834713874	-4.600259028	0.000192462	0.00165367	0.811561137
IFT80	-1.008785941	5.141989319	-5.5542052	2.28902549815687e-05	0.000394468	2.664687387
ARMCX4	-1.008360112	5.563565846	-7.84358018	2.14950375082652e-07	.98286668115106e-05	7.276953137
CAPRIN2	-1.007996525	5.557475319	-6.851666125	1.49174807960807e-06	.87565380641637e-05	5.35214526
ZNF805	-1.007961313	4.22359658	-5.853361742	1.19688969195982e-05	0.000264375	3.389432771
ZNF267	-1.006849139	2.590811864	-5.348647531	3.59504016364304e-05	0.000534777	2.415268765
DFNB59	-1.005960599	1.394401649	-5.604054726	2.05311267872566e-05	0.000366252	2.904542788
DONSON	-1.005406072	4.711668412	-6.22460608	5.43487604282977e-06	0.000165003	4.125441582
JMJD1C	-1.004804596	6.123254187	-5.849627003	1.20653843952349e-05	0.00026554	3.242501739
LMLN	-1.003621603	3.979175294	-8.403913771	7.62546693752972e-08	1.7169760773097e-05	8.301876889
TMTC2	-1.003549842	6.007648497	-4.397202799	0.000305568	0.002308791	0.033374204
CLEC2D	-1.003515977	2.607553047	-6.709584532	1.9903566104773e-06	.18127378878506e-05	5.134064505
C4orf33	-1.001648658	3.560673659	-5.579310085	2.16693402407645e-05	0.000379515	2.860776952
TRIM52	-1.00144106	4.033790703	-6.891045551	1.37782440628896e-06	.67338976514648e-05	5.511419764

Table S6: GO Terms based on the upregulated genes in the embedded and suspended samples at DIS 60

Embedded GO Terms based on Upregulated Genes					
**source**	**term_name**	**term_id**	**adj_p_value**	**neg_log10_adj_p_value**	**intersections**
GO:MF	protein binding	GO:0005515	1E-05	4.948566570734577	COL3A1,COL1A1,GAL,UTS2,SIX1,CXCL8,DYSF,COL8A1,CMKLR1,PRRG2,RGS10,TNFAIP6,ACAN,KCNE4,MSC,HR,OPRD1,KRT80,FOLR3,NPR3,COL5A3,ICAM1,TGFBI,HTR6,TBX3,KCNV1,PSMB8,TMEM171,GATA4,BHLHA15,SERPINE1,SYNPO,HSPB7,IL32,RELB,TNFRSF9,SH3TC1,KL,CARTPT,F2RL2,ARHGDIB,PSMB9,NTSR1,ADRA1B,CPNE6,SP100,PTH2R,EGR2,SPHKAP,OSMR,PDYN,HGF,HTR2B,VGF,SERPINA3,PNPLA5,TICAM1,MGLL,SLC47A1,KCNA1,TG,PLXND1,CCKBR,CORO2A,SCN5A,A2M,S100A10,CEMIP,TIMP4,TTC39A,DAAM2,SFRP1,PDE2A,DYNC1I1,USH1G,PPARG,TRIM58,DLC1,WIPF3,EMILIN3,ITGB3,P3H2,TRPC5,TNFAIP8L3,SLC2A6,ADAMTSL3,LY6E,SPRY4,PSD4,DHCR24,ESRP1,ADRA2C,RIN3,LHFPL5,SLC6A17,CHI3L1,ETV4,IL34,ZNF860,GAS7,GPX3,TPBGL,GABRA5,PHYHIP,FAM163B,SRXN1,CCL2,HOXC13,ATP10A,OSGIN1,SHROOM1,PHF24,FAM131C,KCNJ12,LOXL2,L3MBTL4,CBR3,SPHK1,HTATIP2,ATP2A3,PLEKHF1,SCUBE1,TMEM54,ME1,LRRC61,NQO1,DACT2,TRIM14,PARVB,CAMKV,CHST8,RSAD2,TLE2,ETV5,SPRED3,C1ORF216,P4HA3,SEMA3A,KIF26A,THBS1,NFAM1,RNF112,RCAN1,CARD10,SHE,ADM2,ADAM11,ADRA2A,EGR4,MPPED2,SPTB,MMP28,CDK18,BEGAIN,B3GNT2,TEAD4,OPCML,STAC,ANGPTL1,INF2,FABP3,SVOPL,NXPH3,LZTS1,ASTN2,THY1,NR4A3,FAM83G,ULBP2,PRR19,DOK5,AVPR1A,DHCR7,LRFN2,PCYT2,KCNE1,PTPRN,FHL2,PDLIM4,HMOX1,FRMPD1,SEZ6,EPHX1,MYD88,ATP2B3,TMTC1,MICAL2,IPCEF1,SNX7,IL12RB2,EXTL1,SH3RF3,LRRK1,OSBPL10,EGFL7,IRAK1,FNDC9,LDLR,DNAJA4,ICOSLG,PITPNM2,BDNF,AHNAK2,KCNA6,NLRC5,IGFBP3,KLF2,LKAAEAR1,PEX5L,ATP8A2,TMEM151A,DUSP5,SLC4A3,NPM2,PACSIN3,FASN,SSTR2,KCNQ5,HYAL3,CHRNA4,PALM3,ANXA11,DMTN,ATP1A3,ADAP1,INSIG1,G6PD,INPP5J,PIWIL4,ACAT2,CHRNB2,MVD,ANKRD34A,CCDC86,TNFAIP2,SQSTM1,SYT16,STYK1,PC,IL10RA,ADCK2,EEF1A2,KIAA0319,TUBB4A,DOC2B,TGFB1,PLCB2,MAFF,KHDRBS3,FNTB,MYLK4,DAPK2,F12,NUDT14,KCNK3,KIRREL3,GRIN1,TMEM229B,TMEM120A,ATP6V0A1,OLFM2,GAMT,TKT,PITPNM1,PEMT,PLEKHG3,SLCO4A1,TANC2,CSRNP1,CYP1A1,OPLAH,SHB,EMID1,CABLES1,SLC7A5,TAGLN2,FBXO41,MAP1A,SREBF2,RIMBP3
GO:MF	G protein-coupled amine receptor activity	GO:0008227	0.001	2.8968239237422453	HTR6,ADRA1B,HTR2B,ADRA2C,CHRM4,HRH2,ADRA2A
GO:MF	G protein-coupled peptide receptor activity	GO:0008528	0.004	2.3547663870524294	GAL,OPRD1,NPR3,F2RL2,NTSR1,CCKBR,GPR83,HCRTR1,AVPR1A,SSTR2
GO:MF	potassium ion transmembrane transporter activity	GO:0015079	0.006	2.252180878606022	KCNE4,KCNV1,SLC24A2,KCNA1,KCNK12,KCNJ12,KCNE1,SCN2B,KCNA6,KCNQ5,ATP1A3,KCNK3
GO:MF	signaling receptor binding	GO:0005102	0.007	2.1469859346185136	COL3A1,GAL,UTS2,CXCL8,OPRD1,ICAM1,TGFBI,SERPINE1,IL32,KL,CARTPT,F2RL2,OSMR,PDYN,HGF,VGF,TG,CCKBR,A2M,SFRP1,ITGB3,ADRA2C,IL34,GABRA5,CCL2,OSGIN1,SEMA3A,THBS1,ADM2,ADAM11,ADRA2A,ANGPTL1,NXPH3,THY1,ULBP2,AVPR1A,MYD88,EGFL7,ICOSLG,PITPNM2,BDNF,DMTN,SQSTM1,TGFB1,PITPNM1,RIMBP3
GO:MF	peptide receptor activity	GO:0001653	0.007	2.1445276557210806	GAL,OPRD1,NPR3,F2RL2,NTSR1,CCKBR,GPR83,HCRTR1,AVPR1A,SSTR2

GO:MF	voltage-gated potassium channel activity	GO:0005249	0.009	2.0668985359093117	KCNE4,KCNV1,KCNA1,KCNJ12,KCNE1,SCN2B,KCNA6,KCNQ5,KCNK3
GO:MF	potassium channel activity	GO:0005267	0.016	1.7926560082097938	KCNE4,KCNV1,KCNA1,KCNK12,KCNJ12,KCNE1,SCN2B,KCNA6,KCNQ5,KCNK3
GO:MF	amide binding	GO:0033218	0.02103288627963 9285	1.6771011263003672	CMKLR1,FOLR3,NPR3,PTH2R,CCKBR,CEMIP,PPARG,DHCR24,HCRTR1,AVPR1A,LDLR,PNPLA3,PEX5L,FASN,ATP1A3,PC,GRIN1,SLC7A5
GO:MF	growth factor binding	GO:0019838	0.031	1.5049948089017045	COL3A1,COL1A1,KL,OSMR,SCN5A,A2M,ITGB3,THBS1,IGFBP3,IL10RA
GO:MF	alpha-adrenergic receptor activity	GO:0004936	0.039	1.4131597903402557	ADRA1B,ADRA2C,ADRA2A
GO:BP	multicellular organismal process	GO:0032501	1.067036829635 4323e-15	14.971820590293058	COL3A1,COL1A1,GAL,UTS2,SIX1,CXCL8,DYSF,COL8A1,CMKLR1,TNFAIP6,ACAN,KCNE4,MSC,OPRD1,KRT80,FOLR3,PLA2G3,NPR3,ICAM1,TGFBI,HTR6,TBX3,GATA4,BHLHA15,PAX1,SERPINE1,HSPB7,IL32,MMP19,C2CD4A,RELB,TNFRSF9,KL,CARTPT,F2RL2,ARHGDIB,NTSR1,ADRA1B,SLC24A2,CPNE6,SP100,PTH2R,EGR2,PDYN,HGF,HTR2B,VGF,OR2W3,SERPINA3,TICAM1,MGLL,RCVRN,KCNA1,TG,PLXND1,CCKBR,GDA,SCN5A,A2M,S100A10,CEMIP,GPR83,DAAM2,SFRP1,PDE2A,USH1G,PPARG,TRIM58,DLC1,WIPF3,ITGB3,TRPC5,SPRY4,DHCR24,ESRP1,ADRA2C,LHFPL5,SLC6A17,CHI3L1,IGSF21,ETV4,IL34,GAS7,GABRA5,CCL2,HOXC13,PHF24,KCNJ12,LOXL2,CBR3,SPHK1,CDHR1,HTATIP2,ATP2A3,SCUBE1,DACT2,CHST8,RSAD2,TMEM132E,ETV5,SPRED3,MARVELD1,SEMA3A,KIF26A,THBS1,NFAM1,RNF112,HRH2,RCAN1,CARD10,ADM2,ADAM11,DCHS2,ADRA2A,HCRTR1,BEGAIN,B3GNT2,TEAD4,OPCML,STAC,TFCP2L1,LZTS1,ASTN2,THY1,NR4A3,ULBP2,DOK5,AVPR1A,KCNE1,PTPRN,FHL2,PDLIM4,HMOX1,SEZ6,SCN2B,MYD88,ATP2B3,ST3GAL1,MICAL2,IL12RB2,EXTL1,LRRK1,EGFL7,IRAK1,LDLR,DNAJA4,ICOSLG,BDNF,IGFBP3,KLF2,ATP8A2,DUSP5,SLC4A3,NPM2,FASN,SSTR2,HYAL3,CHRNA4,GJC2,DMTN,SLC7A2,ATP1A3,INSIG1,G6PD,INPP5J,PIWIL4,CHRNB2,TNFAIP2,SQSTM1,IL10RA,KIAA0319,TGFB1,PLCB2,MAFF,F12,KIRREL3,GRIN1,TMEM229B,TMEM120A,GAMT,PITPNM1,PEMT,TANC2,CSRNP1,CYP1A1,SHB,CABLES1,SLC7A5,MAP1A,RIMBP3
GO:BP	developmental process	GO:0032502	1.091387524742 7753e-12	11.96202101478811	COL3A1,COL1A1,GAL,SIX1,CXCL8,COL8A1,CMKLR1,TNFAIP6,ACAN,MSC,KRT80,PLA2G3,NPR3,COL5A3,ICAM1,TGFBI,HTR6,TBX3,PSMB8,GATA4,BHLHA15,PAX1,SERPINE1,HSPB7,MMP19,RELB,TNFRSF9,KL,CARTPT,ARHGDIB,CPNE6,SP100,EGR2,HGF,HTR2B,VGF,KCNA1,TG,PLXND1,CCKBR,GDA,SCN5A,A2M,S100A10,DAAM2,SFRP1,PDE2A,USH1G,PPARG,TRIM58,DLC1,WIPF3,ITGB3,TRPC5,SPRY4,MMP11,DHCR24,ESRP1,ADRA2C,LHFPL5,SLC6A17,CHI3L1,IGSF21,ETV4,IL34,GAS7,GABRA5,CCL2,HOXC13,ATP10A,OSGIN1,SHROOM1,LOXL2,SPHK1,CDHR1,HTATIP2,SCUBE1,DACT2,PARVB,CHST8,RSAD2,TMEM132E,TLE2,ETV5,SPRED3,MARVELD1,SEMA3A,KIF26A,THBS1,NFAM1,RNF112,RCAN1,CARD10,ADM2,DCHS2,B3GNT2,TEAD4,OPCML,INF2,TFCP2L1,FABP3,LZTS1,ASTN2,THY1,NR4A3,DOK5,AVPR1A,KCNE1,PTPRN,FHL2,PDLIM4,HMOX1,SEZ6,SCN2B,MYD88,ST3GAL1,MICAL2,EXTL1,LRRK1,EGFL7,LDLR,BDNF,IGFBP3,KLF2,PNPLA3,ATP8A2,DUSP5,NPM2,FASN,SSTR2,HYAL3,PALM3,GJC2,DMTN,INSIG1,G6PD,INPP5J,PIWIL4,CHRNB2,TNFAIP2,SQSTM1,KIAA0319,TGFB1,MKX,MAFF,KCNK3,KIRREL3,GRIN1,TMEM120A,ATP6V0A1,OLFM2,GAMT,PITPNM1,PEMT,TANC2,CSRNP1,CYP1A1,SHB,CABLES1,SLC7A5,TAGLN2,MAP1A,RIMBP3

GO:BP	anatomical structure development	GO:0048856	2.0724173227403876e-12	11.683522786227071	COL3A1,COL1A1,GAL,SIX1,CXCL8,COL8A1,CMKLR1,TNFAIP6,ACAN,MSC,KRT80,PLA2G3,NPR3,COL5A3,ICAM1,TGFBI,HTR6,TBX3,GATA4,BHLHA15,PAX1,SERPINE1,HSPB7,MMP19,RELB,TNFRSF9,KL,CARTPT,ARHGDIB,CPNE6,SP100,EGR2,HGF,HTR2B,VGF,KCNA1,TG,PLXND1,CCKBR,GDA,SCN5A,A2M,S100A10,DAAM2,SFRP1,PDE2A,USH1G,PPARG,TRIM58,DLC1,ITGB3,TRPC5,SPRY4,DHCR24,ESRP1,ADRA2C,LHFPL5,SLC6A17,CHI3L1,IGSF21,ETV4,IL34,GAS7,GABRA5,CCL2,HOXC13,ATP10A,SHROOM1,LOXL2,SPHK1,CDHR1,HTATIP2,SCUBE1,DACT2,PARVB,CHST8,RSAD2,TMEM132E,TLE2,ETV5,SPRED3,MARVELD1,SEMA3A,KIF26A,THBS1,NFAM1,RNF112,RCAN1,CARD10,ADM2,DCHS2,B3GNT2,TEAD4,OPCML,INF2,TFCP2L1,LZTS1,ASTN2,THY1,NR4A3,DOK5,AVPR1A,KCNE1,PTPRN,FHL2,PDLIM4,HMOX1,SEZ6,SCN2B,MYD88,ST3GAL1,MICAL2,EXTL1,LRRK1,EGFL7,LDLR,BDNF,IGFBP3,KLF2,ATP8A2,DUSP5,NPM2,FASN,SSTR2,HYAL3,PALM3,GJC2,DMTN,INSIG1,G6PD,INPP5J,CHRNB2,TNFAIP2,KIAA0319,TGFB1,MKX,MAFF,KCNK3,KIRREL3,GRIN1,OLFM2,GAMT,PITPNM1,PEMT,TANC2,CSRNP1,CYP1A1,SHB,CABLES1,SLC7A5,TAGLN2,MAP1A,RIMBP3
GO:BP	multicellular organism development	GO:0007275	1.2061723587359998e-11	10.918590628265976	COL3A1,COL1A1,GAL,SIX1,CXCL8,COL8A1,CMKLR1,TNFAIP6,ACAN,MSC,PLA2G3,NPR3,TGFBI,HTR6,TBX3,GATA4,BHLHA15,PAX1,SERPINE1,HSPB7,MMP19,KL,CARTPT,ARHGDIB,CPNE6,SP100,EGR2,HGF,HTR2B,VGF,KCNA1,TG,PLXND1,CCKBR,GDA,SCN5A,A2M,S100A10,DAAM2,SFRP1,PDE2A,USH1G,PPARG,DLC1,ITGB3,TRPC5,SPRY4,DHCR24,ESRP1,ADRA2C,LHFPL5,SLC6A17,CHI3L1,IGSF21,ETV4,IL34,GAS7,GABRA5,CCL2,HOXC13,LOXL2,SPHK1,CDHR1,HTATIP2,SCUBE1,DACT2,CHST8,TMEM132E,ETV5,SPRED3,MARVELD1,SEMA3A,KIF26A,THBS1,NFAM1,RNF112,CARD10,ADM2,DCHS2,B3GNT2,TEAD4,OPCML,TFCP2L1,LZTS1,ASTN2,THY1,NR4A3,DOK5,AVPR1A,KCNE1,PTPRN,FHL2,PDLIM4,HMOX1,SEZ6,SCN2B,MYD88,MICAL2,EXTL1,LRRK1,EGFL7,LDLR,BDNF,KLF2,ATP8A2,DUSP5,NPM2,SSTR2,HYAL3,GJC2,INSIG1,G6PD,INPP5J,CHRNB2,TNFAIP2,KIAA0319,TGFB1,MAFF,KIRREL3,GRIN1,PITPNM1,PEMT,TANC2,CSRNP1,CYP1A1,SHB,CABLES1,SLC7A5,MAP1A
GO:BP	system development	GO:0048731	2.3894056863481453e-11	10.621710107100473	COL3A1,COL1A1,SIX1,CXCL8,COL8A1,CMKLR1,TNFAIP6,ACAN,MSC,PLA2G3,NPR3,TGFBI,HTR6,TBX3,GATA4,BHLHA15,PAX1,SERPINE1,HSPB7,MMP19,CPNE6,SP100,EGR2,HGF,HTR2B,VGF,KCNA1,TG,PLXND1,CCKBR,GDA,SCN5A,A2M,S100A10,DAAM2,SFRP1,PDE2A,USH1G,PPARG,DLC1,ITGB3,TRPC5,DHCR24,ESRP1,ADRA2C,LHFPL5,SLC6A17,CHI3L1,IGSF21,IL34,GAS7,GABRA5,CCL2,LOXL2,SPHK1,CDHR1,HTATIP2,SCUBE1,DACT2,CHST8,TMEM132E,ETV5,SPRED3,MARVELD1,SEMA3A,KIF26A,THBS1,RNF112,CARD10,ADM2,DCHS2,B3GNT2,TEAD4,OPCML,TFCP2L1,LZTS1,ASTN2,THY1,DOK5,AVPR1A,KCNE1,PTPRN,FHL2,PDLIM4,HMOX1,SEZ6,SCN2B,MYD88,MICAL2,EXTL1,LRRK1,EGFL7,LDLR,BDNF,KLF2,ATP8A2,SSTR2,HYAL3,GJC2,INSIG1,G6PD,INPP5J,CHRNB2,TNFAIP2,KIAA0319,TGFB1,KIRREL3,GRIN1,PITPNM1,TANC2,CSRNP1,CYP1A1,SHB,CABLES1,SLC7A5,MAP1A
GO:BP	regulation of multicellular organismal process	GO:0051239	3.378617851109056e-10	9.471260927651043	COL1A1,GAL,SIX1,CXCL8,CMKLR1,TNFAIP6,ACAN,KCNE4,PLA2G3,NPR3,TBX3,GATA4,SERPINE1,HSPB7,IL32,RELB,TNFRSF9,KL,CARTPT,ARHGDIB,NTSR1,ADRA1B,SP100,PTH2R,EGR2,HGF,HTR2B,TICAM1,MGLL,KCNA1,TG,PLXND1,SCN5A,S100A10,DAAM2,SFRP1,PPARG,ITGB3,TRPC5,ESRP1,ADRA2C,CHI3L1,ETV4,IL34,CCL2,KCNJ12,LOXL2,SPHK1,HTATIP2,ATP2A3,RSAD2,ETV5,SPRED3,SEMA3A,THBS1,NFAM1,RNF112,HRH2,CARD10,ADM2,ADRA2A,BEGAIN,TEAD4,THY1,NR4A3,AVPR1A,KCNE1,HMOX1,SCN2B,MYD88,ATP2B3,IL12RB2,IRAK1,LDLR,DNAJA4,ICOSLG,BDNF,KLF2,ATP8A2,SLC4A3,GJC2,DMTN,ATP1A3,G6PD,CHRNB2,KIAA0319,TGFB1,MAFF,F12,GRIN1,GAMT,PEMT,SHB,SLC7A5

GO:BP	signaling	GO:0023052	2.437155535101260 5e-9	8.61311675	COL3A1,COL1A1,GAL,UTS2,SIX1,CXCL8,DYSF,CMKLR1,RGS10,TNFAIP6,OPRD1,PLA2G3,NPR3,ICAM1,HTR6,TBX3,GATA4,BHLHA15,SERPINE1,IL32,RELB,KL,CARTPT,F2RL2,ARHGDIB,NTSR1,ADRA1B,SLC24A2,SP100,PTH2R,EGR2,OSMR,PDYN,HGF,HTR2B,VGF,OR2W3,TICAM1,MGLL,RCVRN,ARHGAP36,KCNA1,TG,PLXND1,CCKBR,CORO2A,SCN5A,TIMP4,GPR83,DAAM2,SFRP1,PDE2A,PPARG,TRIM58,DLC1,ITGB3,TNFAIP8L3,LY6E,SPRY4,PSD4,DHCR24,ADRA2C,RIN3,CHI3L1,IL34,CHRM4,TPBGL,GABRA5,CCL2,OSGIN1,PHF24,SPHK1,ATP2A3,PLEKHF1,SCUBE1,NQO1,DACT2,EVC2,CAMKV,RSAD2,TLE2,SPRED3,SEMA3A,KIF26A,THBS1,NFAM1,HRH2,RCAN1,CARD10,ADM2,ADAM11,ADRA2A,HCRTR1,BEGAIN,TEAD4,STAC,ANGPTL1,NXPH3,LZTS1,THY1,NR4A3,FAM83G,DOK5,AVPR1A,LRFN2,PTPRN,FHL2,PDLIM4,HMOX1,FRMPD1,SEZ6,SCN2B,MYD88,IL12RB2,SH3RF3,LRRK1,EGFL7,IRAK1,LDLR,ICOSLG,PITPNM2,BDNF,NLRC5,IGFBP3,KLF2,PEX5L,DUSP5,SSTR2,CHRNA4,PALM3,GJC2,DMTN,ADAP1,INSIG1,SHISA8,CHRNB2,SQSTM1,IL10RA,KIAA0319,DOC2B,TGFB1,PLCB2,DAPK2,KCNK3,GRIN1,OLFM2,PITPNM1,PLEKHG3,CSRNP1,SUSD5,SHB,RASGEF1C,MAP1A,SREBF2
GO:BP	regulation of biological process	GO:0050789	3.331384298762951e-9	8.477375265475949	COL3A1,COL1A1,GAL,UTS2,SIX1,CXCL8,DYSF,COL8A1,CMKLR1,RGS10,TNFAIP6,ACAN,KCNE4,MSC,HR,OPRD1,PLA2G3,NPR3,ICAM1,TGFBI,HTR6,MMP1,TBX3,KCNV1,PSMB8,GATA4,BHLHA15,PAX1,SERPINE1,SYNPO,HSPB7,IL32,C2CD4A,RELB,TNFRSF9,KL,CARTPT,F2RL2,ARHGDIB,PSMB9,NTSR1,ADRA1B,SLC24A2,CPNE6,SP100,PTH2R,EGR2,OSMR,PDYN,HGF,HTR2B,VGF,OR2W3,SERPINA3,TICAM1,MGLL,RCVRN,ARHGAP36,KCNA1,TG,PLXND1,CCKBR,CORO2A,SCN5A,A2M,S100A10,CEMIP,TIMP4,GPR83,DAAM2,SFRP1,PDE2A,USH1G,PPARG,TRIM58,DLC1,ITGB3,P3H2,TRPC5,TNFAIP8L3,SLC2A6,LY6E,SPRY4,PSD4,MMP11,DHCR24,ESRP1,ADRA2C,RIN3,CHI3L1,ETV4,IL34,ZNF860,CHRM4,TPBGL,GABRA5,CCL2,HOXC13,ATP10A,OSGIN1,PHF24,KCNK12,KCNJ12,LOXL2,L3MBTL4,SPHK1,HTATIP2,ATP2A3,PLEKHF1,SCUBE1,ME1,NQO1,DACT2,TRIM14,PARVB,EVC2,CAMKV,RSAD2,TLE2,ETV5,SPRED3,SEMA3A,KIF26A,THBS1,NFAM1,RNF112,HRH2,RCAN1,CARD10,ADM2,ADAM11,ADRA2A,EGR4,SPTB,MMP28,HCRTR1,CDK18,BEGAIN,TEAD4,STAC,ANGPTL1,INF2,TFCP2L1,FABP3,NXPH3,LZTS1,ASTN2,THY1,NR4A3,FAM83G,ULBP2,DOK5,AVPR1A,LRFN2,KCNE1,PTPRN,FHL2,PDLIM4,HMOX1,FRMPD1,SEZ6,SCN2B,MYD88,FABP6,ATP2B3,ST3GAL1,MICAL2,SNX7,IL12RB2,SH3RF3,LRRK1,EGFL7,IRAK1,LDLR,DNAJA4,ICOSLG,PITPNM2,BDNF,AHNAK2,KCNA6,NLRC5,IGFBP3,KLF2,PEX5L,ATP8A2,DUSP5,SLC4A3,NPM2,PACSIN3,SSTR2,KCNQ5,HYAL3,CHRNA4,PALM3,GJC2,DMTN,TP53I11,ATP1A3,ADAP1,INSIG1,G6PD,SHISA8,INPP5J,PIWIL4,CHRNB2,MVD,ANKRD34A,SQSTM1,STYK1,PC,IL10RA,EEF1A2,KIAA0319,TUBB4A,DOC2B,TGFB1,MKX,PLCB2,MAFF,KHDRBS3,DAPK2,F12,KCNK3,GRIN1,ATP6V0A1,OLFM2,GAMT,TKT,PITPNM1,PEMT,PLEKHG3,TANC2,CSRNP1,SUSD5,CYP1A1,SHB,RASGEF1C,CABLES1,SLC7A5,MAP1A,SREBF2
GO:BP	cell communication	GO:0007154	4.114660689619586 6e-9	8.385665972529878	COL3A1,COL1A1,GAL,UTS2,SIX1,CXCL8,DYSF,CMKLR1,RGS10,TNFAIP6,OPRD1,PLA2G3,NPR3,ICAM1,HTR6,TBX3,GATA4,BHLHA15,SERPINE1,IL32,RELB,KL,CARTPT,F2RL2,ARHGDIB,NTSR1,ADRA1B,SLC24A2,SP100,PTH2R,EGR2,OSMR,PDYN,HGF,HTR2B,VGF,OR2W3,TICAM1,MGLL,RCVRN,ARHGAP36,KCNA1,TG,PLXND1,CCKBR,CORO2A,SCN5A,TIMP4,GPR83,DAAM2,SFRP1,PDE2A,PPARG,TRIM58,DLC1,ITGB3,TNFAIP8L3,LY6E,SPRY4,PSD4,DHCR24,ADRA2C,RIN3,CHI3L1,IL34,CHRM4,TPBGL,GABRA5,CCL2,OSGIN1,PHF24,SPHK1,ATP2A3,PLEKHF1,SCUBE1,NQO1,DACT2,EVC2,CAMKV,RSAD2,TLE2,SPRED3,SEMA3A,KIF26A,THBS1,NFAM1,HRH2,RCAN1,CARD10,ADM2,ADAM11,ADRA2A,HCRTR1,BEGAIN,TEAD4,STAC,ANGPTL1,NXPH3,LZTS1,THY1,NR4A3,FAM83G,DOK5,AVPR1A,LRFN2,PTPRN,FHL2,PDLIM4,HMOX1,FRMPD1,SEZ6,SCN2B,MYD88,IL12RB2,SH3RF3,LRRK1,EGFL7,IRAK1,ICOSLG,PITPNM2,BDNF,NLRC5,IGFBP3,KLF2,PEX5L,DUSP5,SSTR2,CHRNA4,PALM3,GJC2,DMTN,ATP1A3,ADAP1,INSIG1,SHISA8,CHRNB2,SQSTM1,IL10RA,KIAA0319,DOC2B,TGFB1,PLCB2,DAPK2,KCNK3,GRIN1,OLFM2,PITPNM1,PLEKHG3,CSRNP1,SUSD5,SHB,RASGEF1C,SLC7A5,MAP1A,SREBF2

GO:BP	animal organ development	GO:0048513	1.61093326643835 8e-8	7.792922450032592	COL3A1,COL1A1,GAL,SIX1,CXCL8,COL8A1,TNFAIP6,ACAN,MSC,KRT80,COL5A3,TGFBI,HTR6,TBX3,GATA4,BHLHA15,PAX1,SERPINE1,HSPB7,MMP19,KL,EGR2,HGF,HTR2B,VGF,KCNA1,TG,PLXND1,CCKBR,SCN5A,A2M,DAAM2,SFRP1,PDE2A,USH1G,PPARG,DLC1,ITGB3,SPRY4,DHCR24,ESRP1,LHFPL5,SLC6A17,CHI3L1,ETV4,GABRA5,CCL2,HOXC13,LOXL2,SPHK1,SCUBE1,DACT2,TMEM132E,TLE2,SPRED3,SEMA3A,KIF26A,RNF112,RCAN1,DCHS2,TEAD4,TFCP2L1,THY1,NR4A3,AVPR1A,KCNE1,PTPRN,FHL2,PDLIM4,SEZ6,MYD88,MICAL2,LRRK1,KLF2,ATP8A2,FASN,SSTR2,HYAL3,INSIG1,G6PD,CHRNB2,TGFB1,MKX,MAFF,KCNK3,KIRREL3,GRIN1,GAMT,PITPNM1,CSRNP1,CYP1A1,SLC7A5
GO:BP	biological regulation	GO:0065007	2.93246744774504 5e-8	7.532766800130819	COL3A1,COL1A1,GAL,UTS2,SIX1,CXCL8,DYSF,COL8A1,CMKLR1,RGS10,TNFAIP6,ACAN,KCNE4,MSC,HR,OPRD1,PLA2G3,NPR3,ICAM1,TGFBI,HTR6,MMP1,TBX3,KCNV1,PSMB8,GATA4,BHLHA15,PAX1,SERPINE1,SYNPO,HSPB7,IL32,C2CD4A,RELB,TNFRSF9,KL,CARTPT,F2RL2,ARHGDIB,PSMB9,NTSR1,ADRA1B,SLC24A2,CPNE6,SP100,PTH2R,EGR2,OSMR,PDYN,HGF,HTR2B,VGF,OR2W3,SERPINA3,TICAM1,MGLL,RCVRN,ARHGAP36,KCNA1,TG,PLXND1,CCKBR,CORO2A,SCN5A,A2M,S100A10,CEMIP,TIMP4,GPR83,DAAM2,SFRP1,PDE2A,USH1G,PPARG,TRIM58,DLC1,ITGB3,P3H2,TRPC5,TNFAIP8L3,SLC2A6,LY6E,SPRY4,PSD4,MMP11,DHCR24,ESRP1,ADRA2C,RIN3,CHI3L1,ETV4,IL34,ZNF860,CHRM4,TPBGL,GABRA5,CCL2,HOXC13,ATP10A,OSGIN1,PHF24,KCNK12,KCNJ12,LOXL2,L3MBTL4,SPHK1,HTATIP2,ATP2A3,PLEKHF1,SCUBE1,ME1,NQO1,DACT2,TRIM14,PARVB,EVC2,CAMKV,CHST8,RSAD2,TLE2,ETV5,SPRED3,SEMA3A,KIF26A,THBS1,NFAM1,RNF112,HRH2,RCAN1,CARD10,ADM2,ADAM11,ADRA2A,EGR4,SPTB,MMP28,HCRTR1,CDK18,BEGAIN,TEAD4,STAC,ANGPTL1,INF2,TFCP2L1,FABP3,NXPH3,LZTS1,ASTN2,THY1,NR4A3,FAM83G,ULBP2,DOK5,AVPR1A,DHCR7,LRFN2,KCNE1,PTPRN,FHL2,PDLIM4,HMOX1,FRMPD1,SEZ6,SCN2B,MYD88,FABP6,ATP2B3,ST3GAL1,MICAL2,SNX7,IL12RB2,SH3RF3,LRRK1,EGFL7,IRAK1,LDLR,DNAJA4,ICOSLG,PITPNM2,BDNF,AHNAK2,KCNA6,NLRC5,IGFBP3,KLF2,PEX5L,ATP8A2,DUSP5,SLC4A3,NPM2,PACSIN3,SSTR2,KCNQ5,HYAL3,CHRNA4,PALM3,GJC2,DMTN,TP53I11,ATP1A3,ADAP1,INSIG1,G6PD,SHISA8,INPP5J,PIWIL4,CHRNB2,MVD,ANKRD34A,SQSTM1,STYK1,PC,IL10RA,EEF1A2,KIAA0319,TUBB4A,DOC2B,TGFB1,MKX,PLCB2,MAFF,KHDRBS3,DAPK2,F12,KCNK3,GRIN1,ATP6V0A1,OLFM2,GAMT,TKT,PITPNM1,PEMT,PLEKHG3,SLCO4A1,TANC2,CSRNP1,SUSD5,CYP1A1,SHB,RASGEF1C,CABLES1,SLC7A5,MAP1A,SREBF2
GO:BP	response to stimulus	GO:0050896	3.14369418886292 64e-7	6.502559707718174	COL3A1,COL1A1,GAL,UTS2,SIX1,CXCL8,DYSF,CMKLR1,RGS10,TNFAIP6,MSC,OPRD1,PLA2G3,NPR3,ICAM1,TGFBI,HTR6,MMP1,TBX3,GATA4,BHLHA15,SERPINE1,HSPB7,IL32,MMP19,C2CD4A,RELB,KL,CARTPT,F2RL2,ARHGDIB,NTSR1,ADRA1B,SLC24A2,CPNE6,SP100,PTH2R,EGR2,OSMR,PDYN,HGF,HTR2B,VGF,OR2W3,SERPINA3,TICAM1,MGLL,RCVRN,SLC47A1,ARHGAP36,KCNA1,TG,PLXND1,CCKBR,CORO2A,SCN5A,A2M,S100A10,TIMP4,GPR83,DAAM2,SFRP1,PDE2A,PPARG,TRIM58,DLC1,ITGB3,TNFAIP8L3,LY6E,SPRY4,PSD4,DHCR24,ADRA2C,RIN3,LHFPL5,CHI3L1,IL34,GPX3,CHRM4,TPBGL,GABRA5,SRXN1,CCL2,OSGIN1,PHF24,LOXL2,CBR3,SPHK1,ATP2A3,PLEKHF1,SCUBE1,ME1,NQO1,DACT2,TRIM14,EVC2,RSAD2,TLE2,ETV5,SPRED3,SEMA3A,KIF26A,THBS1,NFAM1,RNF112,HRH2,RCAN1,CARD10,ADM2,ADAM11,ADRA2A,MMP28,HCRTR1,BEGAIN,B3GNT2,TEAD4,STAC,ANGPTL1,NXPH3,THY1,NR4A3,FAM83G,ULBP2,DOK5,AVPR1A,KCNE1,PTPRN,FHL2,HMOX1,FRMPD1,SEZ6,EPHX1,SCN2B,MYD88,ST3GAL1,IPCEF1,IL12RB2,SH3RF3,LRRK1,EGFL7,IRAK1,LDLR,DNAJA4,ICOSLG,PITPNM2,BDNF,NLRC5,IGFBP3,KLF2,PNPLA3,PEX5L,ATP8A2,DUSP5,FASN,SSTR2,HYAL3,CHRNA4,PALM3,ANXA11,GJC2,DMTN,ATP1A3,ADAP1,INSIG1,G6PD,SHISA8,CHRNB2,SQSTM1,IL10RA,KIAA0319,TGFB1,PLCB2,DAPK2,F12,KCNK3,GRIN1,TMEM229B,TMEM120A,OLFM2,PITPNM1,PLEKHG3,CSRNP1,SUSD5,CYP1A1,SHB,RASGEF1C,SLC7A5,SREBF2

GO:BP	regulation of cellular process	GO:0050794	3.155233058584645e-7	6.5009685564455015	COL3A1,COL1A1,GAL,UTS2,SIX1,CXCL8,DYSF,COL8A1,CMKLR1,RGS10,TNFAIP6,ACAN,KCNE4,MSC,HR,OPRD1,PLA2G3,NPR3,ICAM1,TGFBI,HTR6,MMP1,TBX3,KCNV1,GATA4,BHLHA15,PAX1,SERPINE1,SYNPO,IL32,C2CD4A,RELB,TNFRSF9,KL,CARTPT,F2RL2,ARHGDIB,NTSR1,ADRA1B,SLC24A2,CPNE6,SP100,PTH2R,EGR2,OSMR,PDYN,HGF,HTR2B,VGF,OR2W3,TICAM1,MGLL,RCVRN,ARHGAP36,KCNA1,TG,PLXND1,CCKBR,CORO2A,SCN5A,S100A10,CEMIP,TIMP4,GPR83,DAAM2,SFRP1,PDE2A,USH1G,PPARG,TRIM58,DLC1,ITGB3,P3H2,TRPC5,TNFAIP8L3,SLC2A6,LY6E,SPRY4,PSD4,MMP11,DHCR24,ESRP1,ADRA2C,RIN3,CHI3L1,ETV4,IL34,ZNF860,CHRM4,TPBGL,GABRA5,CCL2,HOXC13,ATP10A,OSGIN1,PHF24,KCNK12,KCNJ12,LOXL2,L3MBTL4,SPHK1,HTATIP2,ATP2A3,PLEKHF1,SCUBE1,ME1,NQO1,DACT2,TRIM14,EVC2,CAMKV,RSAD2,TLE2,ETV5,SPRED3,SEMA3A,KIF26A,THBS1,NFAM1,RNF112,HRH2,RCAN1,CARD10,ADM2,ADAM11,ADRA2A,EGR4,SPTB,MMP28,HCRTR1,CDK18,BEGAIN,TEAD4,STAC,ANGPTL1,INF2,TFCP2L1,FABP3,NXPH3,LZTS1,ASTN2,THY1,NR4A3,FAM83G,ULBP2,DOK5,AVPR1A,LRFN2,KCNE1,PTPRN,FHL2,HMOX1,FRMPD1,SEZ6,SCN2B,MYD88,FABP6,ST3GAL1,MICAL2,SNX7,IL12RB2,SH3RF3,LRRK1,EGFL7,IRAK1,LDLR,DNAJA4,ICOSLG,PITPNM2,BDNF,AHNAK2,KCNA6,NLRC5,IGFBP3,KLF2,PEX5L,ATP8A2,DUSP5,NPM2,PACSIN3,SSTR2,KCNQ5,HYAL3,CHRNA4,PALM3,GJC2,DMTN,TP53I11,ADAP1,INSIG1,G6PD,SHISA8,INPP5J,PIWIL4,CHRNB2,MVD,ANKRD34A,SQSTM1,STYK1,PC,IL10RA,EEF1A2,KIAA0319,TUBB4A,DOC2B,TGFB1,MKX,PLCB2,MAFF,KHDRBS3,DAPK2,F12,KCNK3,GRIN1,ATP6V0A1,OLFM2,PITPNM1,PLEKHG3,TANC2,CSRNP1,SUSD5,CYP1A1,SHB,RASGEF1C,CABLES1,SLC7A5,MAP1A,SREBF2
GO:BP	system process	GO:0003008	5.280881500996858e-7	6.277293577643217	COL1A1,GAL,UTS2,SIX1,KCNE4,NPR3,TGFBI,TBX3,GATA4,HSPB7,C2CD4A,KL,CARTPT,NTSR1,ADRA1B,SLC24A2,EGR2,PDYN,HTR2B,OR2W3,SERPINA3,MGLL,RCVRN,KCNA1,CCKBR,SCN5A,CEMIP,PDE2A,USH1G,PPARG,ADRA2C,LHFPL5,SLC6A17,GABRA5,CCL2,PHF24,KCNJ12,CBR3,SPHK1,ATP2A3,HRH2,RCAN1,ADM2,ADRA2A,BEGAIN,B3GNT2,STAC,NR4A3,AVPR1A,KCNE1,HMOX1,SEZ6,SCN2B,ATP2B3,LDLR,BDNF,KLF2,ATP8A2,SLC4A3,SSTR2,CHRNA4,SLC7A2,ATP1A3,G6PD,CHRNB2,IL10RA,PLCB2,GRIN1,TMEM120A,GAMT,SLC7A5,MAP1A
GO:BP	cellular response to stimulus	GO:0051716	6.735350251295721e-7	6.171639815197138	COL3A1,COL1A1,GAL,UTS2,SIX1,CXCL8,CMKLR1,RGS10,TNFAIP6,MSC,OPRD1,NPR3,ICAM1,HTR6,MMP1,TBX3,GATA4,BHLHA15,SERPINE1,IL32,RELB,KL,CARTPT,F2RL2,ARHGDIB,NTSR1,ADRA1B,SLC24A2,CPNE6,SP100,PTH2R,EGR2,OSMR,PDYN,HGF,HTR2B,VGF,OR2W3,TICAM1,MGLL,RCVRN,ARHGAP36,KCNA1,TG,PLXND1,CCKBR,CORO2A,SCN5A,TIMP4,GPR83,DAAM2,SFRP1,PDE2A,PPARG,TRIM58,DLC1,ITGB3,TNFAIP8L3,LY6E,SPRY4,PSD4,DHCR24,ADRA2C,RIN3,CHI3L1,IL34,GPX3,CHRM4,TPBGL,GABRA5,SRXN1,CCL2,OSGIN1,PHF24,CBR3,SPHK1,ATP2A3,PLEKHF1,SCUBE1,NQO1,DACT2,EVC2,RSAD2,TLE2,ETV5,SPRED3,SEMA3A,KIF26A,THBS1,NFAM1,HRH2,RCAN1,CARD10,ADM2,ADAM11,ADRA2A,MMP28,HCRTR1,BEGAIN,B3GNT2,TEAD4,STAC,ANGPTL1,NXPH3,THY1,NR4A3,FAM83G,DOK5,AVPR1A,KCNE1,PTPRN,FHL2,HMOX1,FRMPD1,SEZ6,EPHX1,MYD88,IPCEF1,IL12RB2,SH3RF3,LRRK1,EGFL7,IRAK1,LDLR,ICOSLG,PITPNM2,BDNF,NLRC5,IGFBP3,KLF2,PNPLA3,PEX5L,DUSP5,FASN,SSTR2,HYAL3,CHRNA4,PALM3,DMTN,ATP1A3,ADAP1,INSIG1,G6PD,SHISA8,CHRNB2,SQSTM1,IL10RA,KIAA0319,TGFB1,PLCB2,DAPK2,KCNK3,GRIN1,OLFM2,PITPNM1,PLEKHG3,CSRNP1,SUSD5,CYP1A1,SHB,RASGEF1C,SLC7A5,SREBF2
GO:BP	cell differentiation	GO:0030154	6.899151354686686e-7	6.161204327327038	COL3A1,COL1A1,SIX1,COL8A1,CMKLR1,TNFAIP6,ACAN,KRT80,PLA2G3,ICAM1,TGFBI,TBX3,PSMB8,GATA4,BHLHA15,PAX1,SERPINE1,MMP19,RELB,TNFRSF9,CARTPT,CPNE6,EGR2,HGF,HTR2B,KCNA1,PLXND1,A2M,S100A10,DAAM2,SFRP1,PDE2A,USH1G,PPARG,TRIM58,WIPF3,ITGB3,TRPC5,MMP11,ESRP1,ADRA2C,LHFPL5,ETV4,IL34,GAS7,GABRA5,CCL2,OSGIN1,LOXL2,CDHR1,HTATIP2,SCUBE1,DACT2,RSAD2,TMEM132E,ETV5,SPRED3,SEMA3A,KIF26A,NFAM1,RNF112,RCAN1,B3GNT2,TEAD4,OPCML,TFCP2L1,FABP3,LZTS1,ASTN2,THY1,NR4A3,DOK5,AVPR1A,KCNE1,FHL2,SEZ6,MYD88,ST3GAL1,LRRK1,EGFL7,LDLR,BDNF,IGFBP3,KLF2,PNPLA3,ATP8A2,NPM2,FASN,GJC2,DMTN,INSIG1,G6PD,INPP5J,PIWIL4,CHRNB2,TNFAIP2,SQSTM1,KIAA0319,TGFB1,MKX,MAFF,KIRREL3,TMEM120A,OLFM2,TANC2,CYP1A1,SHB,SLC7A5,TAGLN2,MAP1A,RIMBP3

GO:BP	cellular developmental process	GO:0048869	6.998351445770831e-7	6.155004251746445	COL3A1,COL1A1,SIX1,COL8A1,CMKLR1,TNFAIP6,ACAN,KRT80,PLA2G3,ICAM1,TGFBI,TBX3,PSMB8,GATA4,BHLHA15,PAX1,SERPINE1,MMP19,RELB,TNFRSF9,CARTPT,CPNE6,EGR2,HGF,HTR2B,KCNA1,PLXND1,A2M,S100A10,DAAM2,SFRP1,PDE2A,USH1G,PPARG,TRIM58,WIPF3,ITGB3,TRPC5,MMP11,ESRP1,ADRA2C,LHFPL5,ETV4,IL34,GAS7,GABRA5,CCL2,OSGIN1,LOXL2,CDHR1,HTATIP2,SCUBE1,DACT2,RSAD2,TMEM132E,ETV5,SPRED3,SEMA3A,KIF26A,NFAM1,RNF112,RCAN1,B3GNT2,TEAD4,OPCML,TFCP2L1,FABP3,LZTS1,ASTN2,THY1,NR4A3,DOK5,AVPR1A,KCNE1,FHL2,SEZ6,MYD88,ST3GAL1,LRRK1,EGFL7,LDLR,BDNF,IGFBP3,KLF2,PNPLA3,ATP8A2,NPM2,FASN,GJC2,DMTN,INSIG1,G6PD,INPP5J,PIWIL4,CHRNB2,TNFAIP2,SQSTM1,KIAA0319,TGFB1,MKX,MAFF,KIRREL3,TMEM120A,OLFM2,TANC2,CYP1A1,SHB,SLC7A5,TAGLN2,MAP1A,RIMBP3
GO:BP	circulatory system development	GO:0072359	2E-06	5.652504153021299	COL3A1,COL1A1,SIX1,CXCL8,COL8A1,ACAN,MSC,NPR3,TGFBI,TBX3,GATA4,SERPINE1,HSPB7,MMP19,SP100,EGR2,HTR2B,PLXND1,SCN5A,SFRP1,PDE2A,PPARG,DLC1,ITGB3,CHI3L1,CCL2,LOXL2,SPHK1,HTATIP2,SCUBE1,THBS1,CARD10,ADM2,THY1,FHL2,PDLIM4,HMOX1,MICAL2,EGFL7,LDLR,KLF2,G6PD,TNFAIP2,TGFB1,SHB
GO:BP	response to oxygen-containing compound	GO:1901700	2E-06	5.637791305698842	COL3A1,COL1A1,GAL,SIX1,CXCL8,RGS10,ICAM1,HTR6,GATA4,SERPINE1,MMP19,KL,CPNE6,SP100,EGR2,HGF,HTR2B,VGF,TICAM1,SCN5A,A2M,SFRP1,PDE2A,PPARG,ITGB3,GPX3,CHRM4,CCL2,SPHK1,ME1,NQO1,THBS1,RNF112,HRH2,ADRA2A,NR4A3,AVPR1A,KCNE1,PTPRN,HMOX1,MYD88,IL12RB2,IRAK1,LDLR,KLF2,PNPLA3,SSTR2,PALM3,ANXA11,DMTN,ATP1A3,INSIG1,G6PD,CHRNB2,SQSTM1,IL10RA,TGFB1,GRIN1,CYP1A1,SLC7A5
GO:BP	signal transduction	GO:0007165	3E-06	5.521120914764597	COL3A1,COL1A1,GAL,UTS2,SIX1,CXCL8,CMKLR1,RGS10,TNFAIP6,OPRD1,NPR3,ICAM1,HTR6,GATA4,BHLHA15,SERPINE1,IL32,RELB,KL,CARTPT,F2RL2,ARHGDIB,NTSR1,ADRA1B,SLC24A2,SP100,PTH2R,OSMR,PDYN,HGF,HTR2B,VGF,OR2W3,TICAM1,MGLL,RCVRN,ARHGAP36,KCNA1,TG,PLXND1,CCKBR,CORO2A,TIMP4,GPR83,DAAM2,SFRP1,PDE2A,PPARG,TRIM58,DLC1,ITGB3,TNFAIP8L3,LY6E,SPRY4,PSD4,DHCR24,ADRA2C,RIN3,CHI3L1,IL34,CHRM4,TPBGL,GABRA5,CCL2,OSGIN1,PHF24,SPHK1,ATP2A3,PLEKHF1,SCUBE1,DACT2,EVC2,RSAD2,TLE2,SPRED3,SEMA3A,KIF26A,THBS1,NFAM1,HRH2,RCAN1,CARD10,ADM2,ADAM11,ADRA2A,HCRTR1,BEGAIN,TEAD4,STAC,ANGPTL1,NXPH3,THY1,NR4A3,FAM83G,DOK5,AVPR1A,FHL2,HMOX1,FRMPD1,SEZ6,MYD88,IL12RB2,SH3RF3,LRRK1,EGFL7,IRAK1,ICOSLG,PITPNM2,BDNF,NLRC5,IGFBP3,KLF2,PEX5L,DUSP5,SSTR2,CHRNA4,PALM3,DMTN,ADAP1,INSIG1,SHISA8,CHRNB2,SQSTM1,IL10RA,KIAA0319,TGFB1,PLCB2,DAPK2,GRIN1,OLFM2,PITPNM1,PLEKHG3,CSRNP1,SUSD5,SHB,RASGEF1C,SREBF2
GO:BP	response to chemical	GO:0042221	3E-06	5.501061847797059	COL3A1,COL1A1,GAL,SIX1,CXCL8,CMKLR1,RGS10,TNFAIP6,MSC,OPRD1,ICAM1,HTR6,GATA4,BHLHA15,SERPINE1,HSPB7,MMP19,RELB,KL,CARTPT,NTSR1,CPNE6,SP100,EGR2,OSMR,HGF,HTR2B,VGF,OR2W3,TICAM1,SLC47A1,KCNA1,SCN5A,A2M,TIMP4,GPR83,SFRP1,PDE2A,PPARG,ITGB3,SPRY4,DHCR24,RIN3,CHI3L1,IL34,GPX3,CHRM4,SRXN1,CCL2,LOXL2,CBR3,SPHK1,ME1,NQO1,ETV5,SEMA3A,THBS1,RNF112,HRH2,ADAM11,ADRA2A,MMP28,HCRTR1,B3GNT2,NR4A3,AVPR1A,KCNE1,PTPRN,FHL2,HMOX1,EPHX1,SCN2B,MYD88,IPCEF1,IL12RB2,IRAK1,LDLR,NLRC5,KLF2,PNPLA3,FASN,SSTR2,HYAL3,CHRNA4,PALM3,ANXA11,GJC2,DMTN,ATP1A3,INSIG1,G6PD,CHRNB2,SQSTM1,IL10RA,TGFB1,PLCB2,DAPK2,F12,KCNK3,GRIN1,CYP1A1,SLC7A5,SREBF2
GO:BP	blood vessel development	GO:0001568	4E-06	5.430004111051351	COL3A1,COL1A1,SIX1,CXCL8,COL8A1,NPR3,TGFBI,TBX3,GATA4,SERPINE1,MMP19,SP100,EGR2,PLXND1,SFRP1,PDE2A,PPARG,ITGB3,CHI3L1,CCL2,LOXL2,SPHK1,HTATIP2,THBS1,CARD10,ADM2,THY1,HMOX1,EGFL7,LDLR,KLF2,TNFAIP2,TGFB1,SHB

GO:BP	regulation of signaling	GO:0023051	4E-06	5.40436385 98344375	COL3A1,COL1A1,GAL,CXCL8,DYSF,CMKLR1,RGS10,TNFAIP6,PLA2G3,ICAM1,HTR6,GATA4,SERPINE1,KL,CARTPT,F2RL2,ARHGDIB,NTSR1,ADRA1B,SLC24A2,SP100,EGR2,HGF,HTR2B,VGF,TICAM1,MGLL,DAAM2,SFRP1,PDE2A,PPARG,TRIM58,DLC1,ITGB3,TNFAIP8L3,SPRY4,PSD4,ADRA2C,CHI3L1,IL34,TPBGL,CCL2,PHF24,SPHK1,PLEKHF1,SCUBE1,DACT2,CAMKV,RSAD2,TLE2,SPRED3,SEMA3A,KIF26A,THBS1,NFAM1,RCAN1,ADRA2A,HCRTR1,BEGAIN,LZTS1,THY1,DOK5,LRFN2,FHL2,HMOX1,FRMPD1,SEZ6,MYD88,SH3RF3,LRRK1,EGFL7,IRAK1,LDLR,BDNF,NLRC5,IGFBP3,KLF2,PEX5L,DUSP5,PALM3,INSIG1,SHISA8,CHRNB2,SQSTM1,IL10RA,KIAA0319,DOC2B,DAPK2,GRIN1,PLEKHG3,MAP1A,SREBF2
GO:BP	regulation of cell communicat ion	GO:0010646	5E-06	5.34499386	COL3A1,COL1A1,GAL,CXCL8,DYSF,CMKLR1,RGS10,TNFAIP6,PLA2G3,ICAM1,HTR6,GATA4,SERPINE1,KL,CARTPT,F2RL2,ARHGDIB,NTSR1,ADRA1B,SLC24A2,SP100,EGR2,HGF,HTR2B,VGF,TICAM1,MGLL,DAAM2,SFRP1,PDE2A,PPARG,TRIM58,DLC1,ITGB3,TNFAIP8L3,SPRY4,PSD4,ADRA2C,CHI3L1,IL34,TPBGL,CCL2,PHF24,SPHK1,PLEKHF1,SCUBE1,DACT2,CAMKV,RSAD2,TLE2,SPRED3,SEMA3A,KIF26A,THBS1,NFAM1,RCAN1,ADRA2A,HCRTR1,BEGAIN,LZTS1,THY1,DOK5,AVPR1A,LRFN2,FHL2,HMOX1,FRMPD1,SEZ6,MYD88,SH3RF3,LRRK1,EGFL7,IRAK1,BDNF,NLRC5,IGFBP3,KLF2,PEX5L,DUSP5,PALM3,INSIG1,SHISA8,CHRNB2,SQSTM1,IL10RA,KIAA0319,DOC2B,DAPK2,GRIN1,PLEKHG3,MAP1A,SREBF2
GO:BP	positive regulation of multicellula r organismal process	GO:0051240	5E-06	5.28390596 6216413	COL1A1,GAL,SIX1,CXCL8,CMKLR1,ACAN,PLA2G3,GATA4,SERPINE1,IL32,KL,CARTPT,NTSR1,ADRA1B,PTH2R,EGR2,HGF,HTR2B,TICAM1,PLXND1,PPARG,ITGB3,TRPC5,CHI3L1,ETV4,IL34,CCL2,LOXL2,SPHK1,RSAD2,ETV5,THBS1,NFAM1,RNF112,HRH2,ADM2,ADRA2A,TEAD4,THY1,NR4A3,AVPR1A,HMOX1,MYD88,IL12RB2,IRAK1,ICOSLG,BDNF,ATP8A2,GJC2,DMTN,CHRNB2,TGFB1,F12,PEMT,SHB,SLC7A5
GO:BP	regulation of biological quality	GO:0065008	8E-06	5.09316116 8482887	COL3A1,GAL,UTS2,SIX1,DYSF,CMKLR1,KCNE4,OPRD1,PLA2G3,NPR3,TBX3,SERPINE1,C2CD4A,KL,CARTPT,F2RL2,NTSR1,ADRA1B,SLC24A2,EGR2,HTR2B,VGF,KCNA1,TG,PLXND1,CCKBR,SCN5A,DAAM2,SFRP1,PDE2A,PPARG,DLC1,ITGB3,TRPC5,ADRA2C,RIN3,GABRA5,CCL2,ATP10A,KCNK12,SCUBE1,PARVB,CAMKV,CHST8,SEMA3A,THBS1,HRH2,ADM2,ADRA2A,SPTB,LZTS1,AVPR1A,DHCR7,LRFN2,KCNE1,PTPRN,SEZ6,SCN2B,MYD88,BDNF,KLF2,ATP8A2,SLC4A3,CHRNA4,PALM3,DMTN,ATP1A3,SHISA8,CHRNB2,SQSTM1,IL10RA,KIAA0319,DOC2B,F12,KCNK3,GRIN1,ATP6V0A1,SLCO4A1,TANC2,CYP1A1,SLC7A5,MAP1A
GO:BP	regulation of system process	GO:0044057	9E-06	5.06589172 9401017	GAL,KCNE4,GATA4,HSPB7,CARTPT,NTSR1,ADRA1B,EGR2,MGLL,KCNA1,SCN5A,PPARG,ADRA2C,KCNJ12,SPHK1,ATP2A3,HRH2,ADM2,ADRA2A,BEGAIN,NR4A3,AVPR1A,KCNE1,SCN2B,ATP2B3,SLC4A3,ATP1A3,G6PD,GRIN1
GO:BP	regulation of developmen tal process	GO:0050793	9E-06	5.06589014 7687895	COL1A1,GAL,SIX1,CXCL8,CMKLR1,TNFAIP6,ACAN,PLA2G3,TBX3,GATA4,BHLHA15,SERPINE1,KL,CARTPT,CPNE6,SP100,EGR2,HGF,TG,PLXND1,S100A10,DAAM2,SFRP1,PPARG,TRIM58,DLC1,ITGB3,TRPC5,MMP11,ESRP1,ADRA2C,CHI3L1,ETV4,IL34,CCL2,ATP10A,LOXL2,SPHK1,HTATIP2,PARVB,ETV5,SPRED3,SEMA3A,THBS1,NFAM1,RNF112,ADM2,TEAD4,INF2,LZTS1,THY1,HMOX1,SEZ6,LDLR,BDNF,IGFBP3,KLF2,ATP8A2,HYAL3,PALM3,GJC2,DMTN,INSIG1,G6PD,CHRNB2,KIAA0319,TGFB1,MAFF,OLFM2,GAMT,TANC2,SHB,SLC7A5
GO:BP	cell-cell signaling	GO:0007267	1E-05	5.01730765 6696154	COL1A1,GAL,UTS2,DYSF,RGS10,TNFAIP6,PLA2G3,HTR6,TBX3,GATA4,BHLHA15,CARTPT,F2RL2,NTSR1,ADRA1B,SLC24A2,EGR2,PDYN,HGF,HTR2B,VGF,KCNA1,SCN5A,DAAM2,SFRP1,PPARG,ADRA2C,CHRM4,TPBGL,GABRA5,CCL2,PHF24,NQO1,CAMKV,TLE2,HRH2,ADRA2A,HCRTR1,BEGAIN,LZTS1,THY1,LRFN2,PTPRN,PDLIM4,SEZ6,SCN2B,LRRK1,CHRNA4,GJC2,SHISA8,CHRNB2,SQSTM1,DOC2B,TGFB1,KCNK3,GRIN1,MAP1A
GO:BP	response to organic substance	GO:0010033	1E-05	5.0144041	COL3A1,COL1A1,GAL,SIX1,CXCL8,CMKLR1,RGS10,MSC,ICAM1,HTR6,GATA4,BHLHA15,SERPINE1,HSPB7,MMP19,RELB,KL,NTSR1,CPNE6,SP100,EGR2,OSMR,HTR2B,VGF,TICAM1,SCN5A,A2M,TIMP4,GPR83,SFRP1,PDE2A,PPARG,ITGB3,SPRY4,DHCR24,CHI3L1,IL34,GPX3,CHRM4,CCL2,SPHK1,ME1,NQO1,THBS1,HRH2,ADRA2A,HCRTR1,B3GNT2,NR4A3,AVPR1A,KCNE1,PTPRN,FHL2,HMOX1,EPHX1,MYD88,IL12RB2,IRAK1,LDLR,NLRC5,KLF2,PNPLA3,FASN,SSTR2,HYAL3,PALM3,ANXA11,DMTN,ATP1A3,INSIG1,G6PD,CHRNB2,IL10RA,TGFB1,F12,GRIN1,CYP1A1,SLC7A5,SREBF2

GO:BP	circulatory system process	GO:0003013	1E-05	5.0118403928705755	UTS2,KCNE4,NPR3,GATA4,HSPB7,C2CD4A,KL,CARTPT,NTSR1,ADRA1B,HTR2B,SCN5A,PDE2A,PPARG,ADRA2C,SLC6A17,KCNJ12,ATP2A3,HRH2,ADM2,ADRA2A,AVPR1A,KCNE1,SCN2B,ATP2B3,KLF2,SLC4A3,SLC7A2,ATP1A3,SLC7A5
GO:BP	vasculature development	GO:0001944	1E-05	4.988678740982692	COL3A1,COL1A1,SIX1,CXCL8,COL8A1,NPR3,TGFBI,TBX3,GATA4,SERPINE1,MMP19,SP100,EGR2,PLXND1,SFRP1,PDE2A,PPARG,ITGB3,CHI3L1,CCL2,LOXL2,SPHK1,HTATIP2,THBS1,CARD10,ADM2,THY1,HMOX1,EGFL7,LDLR,KLF2,TNFAIP2,TGFB1,SHB
GO:BP	synaptic signaling	GO:0099536	1E-05	4.927578421158535	UTS2,DYSF,RGS10,HTR6,CARTPT,NTSR1,SLC24A2,EGR2,PDYN,HTR2B,VGF,KCNA1,CHRM4,GABRA5,CCL2,PHF24,NQO1,CAMKV,HRH2,ADRA2A,HCRTR1,BEGAIN,LZTS1,LRFN2,PDLIM4,SEZ6,SCN2B,CHRNA4,SHISA8,CHRNB2,SQSTM1,DOC2B,KCNK3,GRIN1,MAP1A
GO:BP	cellular response to chemical stimulus	GO:0070887	1E-05	4.885459912978159	COL3A1,COL1A1,SIX1,CXCL8,CMKLR1,RGS10,TNFAIP6,MSC,OPRD1,ICAM1,HTR6,GATA4,BHLHA15,SERPINE1,RELB,CPNE6,SP100,EGR2,OSMR,HGF,HTR2B,TICAM1,KCNA1,SCN5A,SFRP1,PDE2A,PPARG,ITGB3,SPRY4,RIN3,CHI3L1,IL34,GPX3,CHRM4,SRXN1,CCL2,CBR3,SPHK1,NQO1,ETV5,THBS1,HRH2,ADRA2A,MMP28,HCRTR1,B3GNT2,NR4A3,AVPR1A,KCNE1,PTPRN,HMOX1,EPHX1,MYD88,IPCEF1,IL12RB2,IRAK1,LDLR,NLRC5,KLF2,PNPLA3,FASN,SSTR2,HYAL3,PALM3,DMTN,ATP1A3,INSIG1,G6PD,CHRNB2,SQSTM1,IL10RA,TGFB1,DAPK2,KCNK3,GRIN1,CYP1A1,SLC7A5
GO:BP	anterograde trans-synaptic signaling	GO:0098916	1E-05	4.827701392001445	UTS2,DYSF,HTR6,CARTPT,NTSR1,SLC24A2,EGR2,PDYN,HTR2B,VGF,KCNA1,CHRM4,GABRA5,CCL2,PHF24,NQO1,CAMKV,HRH2,ADRA2A,HCRTR1,BEGAIN,LZTS1,LRFN2,PDLIM4,SEZ6,SCN2B,CHRNA4,SHISA8,CHRNB2,SQSTM1,DOC2B,KCNK3,GRIN1,MAP1A
GO:BP	chemical synaptic transmission	GO:0007268	1E-05	4.827701392001445	UTS2,DYSF,HTR6,CARTPT,NTSR1,SLC24A2,EGR2,PDYN,HTR2B,VGF,KCNA1,CHRM4,GABRA5,CCL2,PHF24,NQO1,CAMKV,HRH2,ADRA2A,HCRTR1,BEGAIN,LZTS1,LRFN2,PDLIM4,SEZ6,SCN2B,CHRNA4,SHISA8,CHRNB2,SQSTM1,DOC2B,KCNK3,GRIN1,MAP1A
GO:BP	response to abiotic stimulus	GO:0009628	1E-05	4.82585642	COL3A1,COL1A1,OPRD1,MMP1,GATA4,RELB,NTSR1,SLC24A2,HTR2B,VGF,RCVRN,KCNA1,SFRP1,PDE2A,PPARG,ITGB3,LHFPL5,CHI3L1,PHF24,LOXL2,NQO1,THBS1,STAC,AVPR1A,KCNE1,HMOX1,SCN2B,MYD88,IRAK1,DNAJA4,ICOSLG,ATP8A2,HYAL3,CHRNA4,CHRNB2,KIAA0319,TGFB1,KCNK3,GRIN1,TMEM120A,PITPNM1,CYP1A1,SLC7A5
GO:BP	trans-synaptic signaling	GO:0099537	2E-05	4.741322016312722	UTS2,DYSF,HTR6,CARTPT,NTSR1,SLC24A2,EGR2,PDYN,HTR2B,VGF,KCNA1,CHRM4,GABRA5,CCL2,PHF24,NQO1,CAMKV,HRH2,ADRA2A,HCRTR1,BEGAIN,LZTS1,LRFN2,PDLIM4,SEZ6,SCN2B,CHRNA4,SHISA8,CHRNB2,SQSTM1,DOC2B,KCNK3,GRIN1,MAP1A
GO:BP	blood circulation	GO:0008015	2E-05	4.697174617830845	UTS2,KCNE4,NPR3,GATA4,HSPB7,C2CD4A,KL,CARTPT,NTSR1,ADRA1B,HTR2B,SCN5A,PDE2A,PPARG,ADRA2C,KCNJ12,ATP2A3,HRH2,ADM2,ADRA2A,AVPR1A,KCNE1,SCN2B,ATP2B3,KLF2,SLC4A3,ATP1A3
GO:BP	regulation of localization	GO:0032879	2E-05	4.663643650322335	GAL,SIX1,DYSF,TNFAIP6,KCNE4,OPRD1,PLA2G3,KCNV1,SERPINE1,CARTPT,F2RL2,NTSR1,SP100,HTR2B,RCVRN,KCNA1,SCN5A,S100A10,CEMIP,SFRP1,USH1G,PPARG,TRIM58,ITGB3,ADRA2C,RIN3,CCL2,KCNK12,KCNJ12,SPHK1,RSAD2,THBS1,CARD10,ADRA2A,STAC,FABP3,ASTN2,THY1,NR4A3,AVPR1A,KCNE1,PTPRN,SCN2B,BDNF,KCNA6,ATP8A2,PACSIN3,KCNQ5,HYAL3,CHRNA4,GJC2,DMTN,INSIG1,G6PD,SHISA8,CHRNB2,SQSTM1,DOC2B,TGFB1,PLCB2,KCNK3,GRIN1,SLC7A5,MAP1A,SREBF2

GO:BP	response to endogenous stimulus	GO:0009719	2E-05	4.661692611289628	COL3A1,COL1A1,GAL,SIX1,CXCL8,RGS10,TNFAIP6,OPRD1,ICAM1,HTR6,GATA4,MMP19,KL,EGR2,HGF,HTR2B,VGF,TICAM1,A2M,TIMP4,GPR83,SFRP1,PDE2A,PPARG,ITGB3,SPRY4,DHCR24,CHRM4,CCL2,SPHK1,ME1,NQO1,SPRED3,THBS1,HRH2,ADRA2A,HCRTR1,NR4A3,FAM83G,DOK5,AVPR1A,KCNE1,FHL2,MYD88,LDLR,BDNF,KLF2,PNPLA3,SSTR2,DMTN,ATP1A3,INSIG1,CHRNB2,TGFB1,SLC7A5,SREBF2
GO:BP	blood vessel morphogenesis	GO:0048514	3E-05	4.521276821721081	COL3A1,SIX1,CXCL8,COL8A1,NPR3,TGFBI,GATA4,SERPINE1,MMP19,SP100,PLXND1,SFRP1,PPARG,ITGB3,CHI3L1,CCL2,LOXL2,SPHK1,HTATIP2,THBS1,CARD10,ADM2,THY1,HMOX1,EGFL7,LDLR,KLF2,TNFAIP2,TGFB1,SHB
GO:BP	regulation of cell migration	GO:0030334	3E-05	4.498784616146531	COL3A1,COL1A1,CXCL8,CMKLR1,TNFAIP6,ICAM1,SERPINE1,ARHGDIB,SP100,HGF,PLXND1,CEMIP,DAAM2,SFRP1,PPARG,DLC1,ITGB3,RIN3,IL34,CCL2,OSGIN1,SPHK1,SEMA3A,KIF26A,THBS1,CARD10,ADRA2A,MMP28,THY1,NR4A3,HMOX1,MYD88,DNAJA4,IGFBP3,DMTN,TGFB1,DAPK2,PLEKHG3
GO:BP	calcium ion transport	GO:0006816	3E-05	4.468691621979311	OPRD1,BHLHA15,NTSR1,SLC24A2,HTR2B,RCVRN,SCN5A,CEMIP,ITGB3,TRPC5,CCL2,ATP2A3,ADRA2A,STAC,THY1,KCNE1,ATP2B3,PACSIN3,CHRNA4,GJC2,G6PD,CHRNB2,TGFB1,PLCB2,GRIN1
GO:BP	metal ion transport	GO:0030001	5E-05	4.308244208096113	GAL,KCNE4,OPRD1,KCNV1,BHLHA15,NTSR1,SLC24A2,HTR2B,RCVRN,KCNA1,SCN5A,CEMIP,ITGB3,TRPC5,SLC6A17,CCL2,KCNK12,KCNJ12,ATP2A3,ADRA2A,STAC,THY1,KCNE1,SCN2B,ATP2B3,KCNA6,PACSIN3,KCNQ5,CHRNA4,GJC2,ATP1A3,G6PD,CHRNB2,TGFB1,PLCB2,KCNK3,GRIN1
GO:BP	monoatomic ion transport	GO:0006811	6E-05	4.259359479039088	GAL,KCNE4,OPRD1,KCNV1,BHLHA15,NTSR1,SLC24A2,HTR2B,RCVRN,SLC47A1,KCNA1,TG,SCN5A,CEMIP,ITGB3,TRPC5,LHFPL5,SLC6A17,GABRA5,CCL2,ATP10A,KCNK12,KCNJ12,ATP2A3,ADRA2A,STAC,THY1,KCNE1,SCN2B,ATP2B3,KCNA6,SLC4A3,PACSIN3,KCNQ5,CHRNA4,GJC2,ATP1A3,G6PD,CHRNB2,TGFB1,PLCB2,KCNK3,GRIN1,TMEM120A,ATP6V0A1,SLCO4A1
GO:BP	anatomical structure morphogenesis	GO:0009653	6E-05	4.225580800850762	COL3A1,COL1A1,SIX1,CXCL8,COL8A1,ACAN,PLA2G3,NPR3,TGFBI,TBX3,GATA4,PAX1,SERPINE1,MMP19,CPNE6,SP100,EGR2,HGF,HTR2B,PLXND1,SCN5A,SFRP1,USH1G,PPARG,DLC1,ITGB3,TRPC5,LHFPL5,CHI3L1,GAS7,CCL2,HOXC13,ATP10A,SHROOM1,LOXL2,SPHK1,CDHR1,HTATIP2,DACT2,PARVB,TLE2,SEMA3A,THBS1,CARD10,ADM2,B3GNT2,TEAD4,INF2,TFCP2L1,LZTS1,ASTN2,THY1,NR4A3,FHL2,HMOX1,MICAL2,EGFL7,LDLR,BDNF,KLF2,ATP8A2,DUSP5,PALM3,DMTN,INSIG1,CHRNB2,TNFAIP2,KIAA0319,TGFB1,KIRREL3,GAMT,TANC2,CSRNP1,SHB,MAP1A

GO:BP	positive regulation of cellular process	GO:0048522	6E-05	4.192504647060179	COL3A1,COL1A1,GAL,SIX1,CXCL8,COL8A1,CMKLR1,TNFAIP6,ACAN,OPRD1,PLA2G3,ICAM1,HTR6,MMP1,TBX3,GATA4,BHLHA15,PAX1,SERPINE1,SYNPO,IL32,RELB,KL,CARTPT,F2RL2,NTSR1,ADRA1B,SLC24A2,CPNE6,SP100,EGR2,OSMR,HGF,HTR2B,TICAM1,KCNA1,PLXND1,CCKBR,SCN5A,S100A10,CEMIP,DAAM2,SFRP1,PDE2A,PPARG,TRIM58,DLC1,ITGB3,TRPC5,TNFAIP8L3,ADRA2C,CHI3L1,ETV4,IL34,CCL2,HOXC13,ATP10A,LOXL2,SPHK1,HTATIP2,PLEKHF1,SCUBE1,NQO1,TRIM14,RSAD2,ETV5,SEMA3A,THBS1,NFAM1,RNF112,CARD10,ADM2,ADRA2A,EGR4,HCRTR1,TEAD4,STAC,TFCP2L1,FABP3,THY1,NR4A3,ULBP2,DOK5,AVPR1A,KCNE1,PTPRN,HMOX1,MYD88,MICAL2,SNX7,IL12RB2,SH3RF3,LRRK1,EGFL7,IRAK1,LDLR,DNAJA4,ICOSLG,BDNF,NLRC5,IGFBP3,KLF2,ATP8A2,NPM2,PACSIN3,HYAL3,GJC2,DMTN,G6PD,CHRNB2,MVD,SQSTM1,IL10RA,EEF1A2,KIAA0319,DOC2B,TGFB1,MAFF,DAPK2,F12,KCNK3,GRIN1,OLFM2,CSRNP1,CYP1A1,SHB,SLC7A5,MAP1A,SREBF2
GO:BP	homeostatic process	GO:0042592	7E-05	4.18158927	COL3A1,CMKLR1,NPR3,ICAM1,GATA4,BHLHA15,KL,CARTPT,NTSR1,SLC24A2,PTH2R,HTR2B,VGF,SERPINA3,PNPLA5,KCNA1,CCKBR,CEMIP,USH1G,PPARG,TRIM58,ITGB3,TRPC5,CCL2,CDHR1,ATP2A3,NQO1,ADRA2A,HCRTR1,FABP3,THY1,NR4A3,AVPR1A,PTPRN,HMOX1,ATP2B3,LRRK1,LDLR,KLF2,PNPLA3,SLC4A3,DMTN,ATP1A3,INSIG1,G6PD,PIWIL4,SQSTM1,IL10RA,TGFB1,PLCB2,GRIN1,ATP6V0A1,PEMT,MAP1A,SREBF2
GO:BP	regulation of transport	GO:0051049	8E-05	4.088134003067105	GAL,DYSF,KCNE4,OPRD1,PLA2G3,KCNV1,SERPINE1,CARTPT,F2RL2,NTSR1,SP100,RCVRN,KCNA1,SCN5A,S100A10,CEMIP,SFRP1,USH1G,PPARG,TRIM58,ITGB3,ADRA2C,RIN3,CCL2,KCNK12,KCNJ12,SPHK1,RSAD2,THBS1,ADRA2A,STAC,FABP3,THY1,NR4A3,AVPR1A,KCNE1,PTPRN,SCN2B,KCNA6,ATP8A2,PACSIN3,KCNQ5,HYAL3,CHRNA4,GJC2,DMTN,INSIG1,G6PD,SHISA8,CHRNB2,DOC2B,TGFB1,KCNK3,GRIN1,SLC7A5,SREBF2
GO:BP	monoatomic ion transmembrane transport	GO:0034220	1E-04	3.989691379587269	GAL,KCNE4,KCNV1,BHLHA15,NTSR1,SLC24A2,HTR2B,SLC47A1,KCNA1,SCN5A,CEMIP,ITGB3,TRPC5,SLC6A17,GABRA5,CCL2,ATP10A,KCNK12,KCNJ12,ATP2A3,ADRA2A,STAC,THY1,KCNE1,SCN2B,ATP2B3,KCNA6,SLC4A3,KCNQ5,CHRNA4,GJC2,ATP1A3,G6PD,CHRNB2,TGFB1,PLCB2,KCNK3,GRIN1,TMEM120A,ATP6V0A1
GO:BP	regulation of locomotion	GO:0040012	2E-04	3.7991843331832147	COL3A1,COL1A1,CXCL8,CMKLR1,TNFAIP6,ICAM1,SERPINE1,ARHGDIB,SP100,HGF,PLXND1,CEMIP,DAAM2,SFRP1,PPARG,DLC1,ITGB3,RIN3,IL34,CHRM4,CCL2,OSGIN1,SPHK1,SEMA3A,KIF26A,THBS1,CARD10,ADRA2A,MMP28,THY1,NR4A3,HMOX1,MYD88,DNAJA4,IGFBP3,DMTN,TGFB1,DAPK2,PLEKHG3
GO:BP	regulation of cell motility	GO:2000145	2E-04	3.787752517235493	COL3A1,COL1A1,CXCL8,CMKLR1,TNFAIP6,ICAM1,SERPINE1,ARHGDIB,SP100,HGF,PLXND1,CEMIP,DAAM2,SFRP1,PPARG,DLC1,ITGB3,RIN3,IL34,CCL2,OSGIN1,SPHK1,SEMA3A,KIF26A,THBS1,CARD10,ADRA2A,MMP28,THY1,NR4A3,HMOX1,MYD88,DNAJA4,IGFBP3,DMTN,TGFB1,DAPK2,PLEKHG3
GO:BP	muscle system process	GO:0003012	2E-04	3.699934768113463	UTS2,KCNE4,TBX3,GATA4,ADRA1B,HTR2B,KCNA1,SCN5A,PPARG,ADRA2C,KCNJ12,SPHK1,ADRA2A,STAC,NR4A3,KCNE1,HMOX1,SCN2B,ATP8A2,SSTR2,G6PD,CHRNB2,GAMT

GO:BP	regulation of signal transduction	GO:0009966	2E-04	3.696064592189217	COL3A1,COL1A1,CXCL8,CMKLR1,RGS10,TNFAIP6,ICAM1,HTR6,GATA4,SERPINE1,KL,CARTPT,F2RL2,ARHGDIB,NTSR1,ADRA1B,SP100,HGF,HTR2B,TICAM1,MGLL,DAAM2,SFRP1,PDE2A,PPARG,TRIM58,DLC1,ITGB3,TNFAIP8L3,SPRY4,PSD4,ADRA2C,CHI3L1,IL34,TPBGL,CCL2,PHF24,SPHK1,PLEKHF1,SCUBE1,DACT2,RSAD2,TLE2,SPRED3,SEMA3A,KIF26A,THBS1,NFAM1,RCAN1,ADRA2A,HCRTR1,BEGAIN,THY1,DOK5,FHL2,HMOX1,FRMPD1,SEZ6,MYD88,SH3RF3,LRRK1,EGFL7,IRAK1,BDNF,NLRC5,IGFBP3,KLF2,PEX5L,DUSP5,PALM3,INSIG1,SHISA8,SQSTM1,IL10RA,KIAA0319,DAPK2,GRIN1,PLEKHG3,SREBF2
GO:BP	angiogenesis	GO:0001525	2E-04	3.654619127403878	CXCL8,COL8A1,NPR3,TGFBI,GATA4,SERPINE1,MMP19,SP100,PLXND1,SFRP1,PPARG,ITGB3,CHI3L1,CCL2,LOXL2,SPHK1,HTATIP2,THBS1,CARD10,ADM2,THY1,HMOX1,EGFL7,KLF2,TNFAIP2,SHB
GO:BP	regulation of multicellular organismal developmen	GO:2000026	2E-04	3.638824764599945	GAL,SIX1,CXCL8,TNFAIP6,ACAN,PLA2G3,TBX3,GATA4,SERPINE1,KL,CARTPT,SP100,EGR2,TG,PLXND1,S100A10,DAAM2,SFRP1,PPARG,ITGB3,TRPC5,ESRP1,CHI3L1,ETV4,IL34,LOXL2,SPHK1,HTATIP2,ETV5,SPRED3,SEMA3A,THBS1,NFAM1,RNF112,ADM2,THY1,HMOX1,LDLR,BDNF,KLF2,GJC2,G6PD,KIAA0319,TGFB1,MAFF,SHB,SLC7A5
GO:BP	transport	GO:0006810	3E-04	3.5814351623317835	COL1A1,GAL,CXCL8,DYSF,KCNE4,OPRD1,FOLR3,PLA2G3,TBX3,KCNV1,BHLHA15,SERPINE1,CARTPT,F2RL2,NTSR1,SLC24A2,SP100,EGR2,HTR2B,VGF,RCVRN,SLC47A1,KCNA1,TG,CCKBR,SCN5A,S100A10,CEMIP,SFRP1,DYNC1I1,USH1G,PPARG,TRIM58,WIPF3,ITGB3,TRPC5,TNFAIP8L3,SLC2A6,ADRA2C,RIN3,LHFPL5,SLC6A17,GABRA5,CCL2,ATP10A,KCNK12,KCNJ12,LOXL2,SPHK1,HTATIP2,ATP2A3,PLEKHF1,RSAD2,THBS1,HRH2,ADRA2A,STAC,FABP3,SVOPL,ASTN2,THY1,NR4A3,AVPR1A,KCNE1,PTPRN,SCN2B,MYD88,FABP6,ATP2B3,IPCEF1,SNX7,OSBPL10,LDLR,PITPNM2,KCNA6,PEX5L,ATP8A2,SLC4A3,PACSIN3,KCNQ5,HYAL3,CHRNA4,ANXA11,GJC2,DMTN,SLC7A2,ATP1A3,INSIG1,G6PD,SHISA8,CHRNB2,TNFAIP2,SQSTM1,IL10RA,DOC2B,TGFB1,PLCB2,KCNK3,SLC45A1,GRIN1,TMEM120A,SPRN,ATP6V0A1,OLFM2,PITPNM1,SLCO4A1,TANC2,SLC7A5,MAP1A,SREBF2
GO:BP	cell population proliferation	GO:0008283	3E-04	3.506548614117777	COL3A1,GAL,SIX1,CXCL8,COL8A1,ACAN,PLA2G3,NPR3,ICAM1,TGFBI,TBX3,BHLHA15,PAX1,TNFRSF9,CPNE6,EGR2,OSMR,HGF,HTR2B,TICAM1,KCNA1,PLXND1,CCKBR,SCN5A,DAAM2,SFRP1,USH1G,PPARG,DLC1,ITGB3,P3H2,TRPC5,DHCR24,LHFPL5,IL34,GAS7,CCL2,OSGIN1,LOXL2,SPHK1,LRRC61,PARVB,ETV5,SEMA3A,KIF26A,THBS1,DCHS2,ADRA2A,EGR4,B3GNT2,FABP3,LZTS1,THY1,NR4A3,AVPR1A,PTPRN,HMOX1,SEZ6,MYD88,FABP6,IL12RB2,EGFL7,IRAK1,ICOSLG,BDNF,IGFBP3,ATP8A2,SSTR2,GJC2,DMTN,TP53I11,ATP1A3,INPP5J,CHRNB2,MVD,SQSTM1,KIAA0319,TGFB1,KIRREL3,TANC2,SHB,SLC7A5,MAP1A
GO:BP	positive regulation of biological process	GO:0048518	4E-04	3.373232372586933	COL3A1,COL1A1,GAL,SIX1,CXCL8,COL8A1,CMKLR1,TNFAIP6,ACAN,OPRD1,PLA2G3,ICAM1,HTR6,MMP1,TBX3,GATA4,BHLHA15,PAX1,SERPINE1,SYNPO,IL32,C2CD4A,RELB,KL,CARTPT,F2RL2,NTSR1,ADRA1B,SLC24A2,CPNE6,SP100,PTH2R,EGR2,OSMR,HGF,HTR2B,TICAM1,KCNA1,PLXND1,CCKBR,SCN5A,A2M,S100A10,CEMIP,DAAM2,SFRP1,PDE2A,PPARG,TRIM58,DLC1,ITGB3,TRPC5,TNFAIP8L3,ADRA2C,CHI3L1,ETV4,IL34,CCL2,HOXC13,ATP10A,LOXL2,SPHK1,HTATIP2,ATP2A3,PLEKHF1,SCUBE1,NQO1,TRIM14,RSAD2,ETV5,SEMA3A,THBS1,NFAM1,RNF112,HRH2,CARD10,ADM2,ADRA2A,EGR4,HCRTR1,TEAD4,STAC,TFCP2L1,FABP3,THY1,NR4A3,ULBP2,DOK5,AVPR1A,KCNE1,PTPRN,HMOX1,MYD88,MICAL2,SNX7,IL12RB2,SH3RF3,LRRK1,EGFL7,IRAK1,LDLR,DNAJA4,ICOSLG,BDNF,NLRC5,IGFBP3,KLF2,ATP8A2,NPM2,PACSIN3,HYAL3,GJC2,DMTN,G6PD,CHRNB2,MVD,SQSTM1,IL10RA,EEF1A2,KIAA0319,DOC2B,TGFB1,MAFF,DAPK2,F12,KCNK3,GRIN1,OLFM2,PEMT,CSRNP1,CYP1A1,SHB,SLC7A5,MAP1A,SREBF2
GO:BP	monoatomic cation transport	GO:0006812	4E-04	3.3495443091205983	GAL,KCNE4,OPRD1,KCNV1,BHLHA15,NTSR1,SLC24A2,HTR2B,RCVRN,SLC47A1,KCNA1,SCN5A,CEMIP,ITGB3,TRPC5,SLC6A17,CCL2,KCNK12,KCNJ12,ATP2A3,ADRA2A,STAC,THY1,KCNE1,SCN2B,ATP2B3,KCNA6,PACSIN3,KCNQ5,CHRNA4,GJC2,ATP1A3,G6PD,CHRNB2,TGFB1,PLCB2,KCNK3,GRIN1,ATP6V0A1

GO:BP	regulation of blood circulation	GO:1903522	5E-04	3.3324530272396733	KCNE4,GATA4,HSPB7,ADRA1B,SCN5A,ADRA2C,KCNJ12,ATP2A3,HRH2,ADM2,ADRA2A,AVPR1A,KCNE1,SCN2B,ATP2B3,SLC4A3,ATP1A3
GO:BP	regulation of anatomical structure morphogenesis	GO:0022603	5E-04	3.2827219810876884	SIX1,CXCL8,GATA4,SERPINE1,CPNE6,SP100,HGF,PLXND1,SFRP1,PPARG,DLC1,ITGB3,TRPC5,CHI3L1,CCL2,ATP10A,SPHK1,HTATIP2,PARVB,SEMA3A,THBS1,ADM2,INF2,LZTS1,THY1,HMOX1,BDNF,KLF2,PALM3,DMTN,CHRNB2,KIAA0319,TGFB1,TANC2
GO:BP	regulation of transmembrane transport	GO:0034762	5E-04	3.268347369533009	GAL,KCNE4,KCNV1,NTSR1,KCNA1,SCN5A,CEMIP,ITGB3,CCL2,KCNK12,KCNJ12,THBS1,ADRA2A,STAC,THY1,NR4A3,KCNE1,SCN2B,KCNA6,KCNQ5,GJC2,G6PD,SHISA8,TGFB1,KCNK3,GRIN1,SLC7A5
GO:BP	transmembrane transport	GO:0055085	6E-04	3.2529880604441765	GAL,KCNE4,KCNV1,BHLHA15,NTSR1,SLC24A2,HTR2B,SLC47A1,KCNA1,SCN5A,CEMIP,ITGB3,TRPC5,SLC2A6,SLC6A17,GABRA5,CCL2,ATP10A,KCNK12,KCNJ12,ATP2A3,THBS1,ADRA2A,STAC,SVOPL,THY1,NR4A3,KCNE1,SCN2B,ATP2B3,KCNA6,PEX5L,SLC4A3,KCNQ5,CHRNA4,GJC2,SLC7A2,ATP1A3,G6PD,SHISA8,CHRNB2,TGFB1,PLCB2,KCNK3,SLC45A1,GRIN1,TMEM120A,ATP6V0A1,SLCO4A1,SLC7A5
GO:BP	establishment of localization	GO:0051234	6E-04	3.228662972483989	COL1A1,GAL,CXCL8,DYSF,KCNE4,OPRD1,FOLR3,PLA2G3,TBX3,KCNV1,BHLHA15,SERPINE1,CARTPT,F2RL2,NTSR1,SLC24A2,SP100,EGR2,HTR2B,VGF,RCVRN,SLC47A1,KCNA1,TG,CCKBR,SCN5A,S100A10,CEMIP,SFRP1,DYNC1I1,USH1G,PPARG,TRIM58,WIPF3,ITGB3,TRPC5,TNFAIP8L3,SLC2A6,ADRA2C,RIN3,LHFPL5,SLC6A17,GABRA5,CCL2,ATP10A,KCNK12,KCNJ12,LOXL2,SPHK1,HTATIP2,ATP2A3,PLEKHF1,RSAD2,THBS1,HRH2,ADAM11,ADRA2A,STAC,FABP3,SVOPL,ASTN2,THY1,NR4A3,AVPR1A,KCNE1,PTPRN,FRMPD1,SCN2B,MYD88,FABP6,ATP2B3,IPCEF1,SNX7,OSBPL10,LDLR,PITPNM2,KCNA6,PEX5L,ATP8A2,SLC4A3,PACSIN3,KCNQ5,HYAL3,CHRNA4,ANXA11,GJC2,DMTN,SLC7A2,ATP1A3,INSIG1,G6PD,SHISA8,CHRNB2,TNFAIP2,SQSTM1,IL10RA,DOC2B,TGFB1,PLCB2,KCNK3,SLC45A1,GRIN1,TMEM120A,SPRN,ATP6V0A1,OLFM2,PITPNM1,SLCO4A1,TANC2,SLC7A5,MAP1A,SREBF2
GO:BP	regulation of leukocyte migration	GO:0002685	7E-04	3.1836355697463397	CXCL8,CMKLR1,TNFAIP6,ICAM1,SERPINE1,ITGB3,RIN3,IL34,CCL2,THBS1,MMP28,THY1,HMOX1,MYD88,TGFB1,DAPK2
GO:BP	regulation of response to stimulus	GO:0048583	9E-04	3.040204540640019	COL3A1,COL1A1,CXCL8,CMKLR1,RGS10,TNFAIP6,PLA2G3,ICAM1,HTR6,GATA4,SERPINE1,C2CD4A,KL,CARTPT,F2RL2,ARHGDIB,NTSR1,ADRA1B,SP100,OSMR,HGF,HTR2B,TICAM1,MGLL,SCN5A,A2M,DAAM2,SFRP1,PDE2A,PPARG,TRIM58,DLC1,ITGB3,TNFAIP8L3,SPRY4,PSD4,ADRA2C,RIN3,CHI3L1,IL34,TPBGL,CCL2,PHF24,SPHK1,PLEKHF1,SCUBE1,DACT2,RSAD2,TLE2,SPRED3,SEMA3A,KIF26A,THBS1,NFAM1,RCAN1,ADRA2A,MMP28,HCRTR1,BEGAIN,THY1,NR4A3,ULBP2,DOK5,FHL2,HMOX1,FRMPD1,SEZ6,MYD88,SH3RF3,LRRK1,EGFL7,IRAK1,LDLR,ICOSLG,BDNF,NLRC5,IGFBP3,KLF2,PEX5L,DUSP5,PALM3,DMTN,INSIG1,G6PD,SHISA8,SQSTM1,IL10RA,KIAA0319,DAPK2,F12,GRIN1,PLEKHG3,SHB,SREBF2
GO:BP	phosphorus metabolic process	GO:0006793	0.001	2.983569346024512	OPRD1,PLA2G3,CARTPT,NTSR1,HGF,HTR2B,ACOT4,GDA,CEMIP,SFRP1,PDE2A,PPARG,DLC1,ITGB3,TRPC5,TNFAIP8L3,SPRY4,ADRA2C,CHI3L1,IL34,CCL2,SPHK1,HTATIP2,ME1,NQO1,CAMKV,THBS1,RCAN1,CARD10,ADM2,ADRA2A,CDK18,BEGAIN,FABP3,THY1,PCYT2,PTPRN,FAR2,ST3GAL1,IL12RB2,LRRK1,OSBPL10,IRAK1,LDLR,UAP1L1,PITPNM2,BDNF,NLRC5,IGFBP3,PNPLA3,DUSP5,GJC2,DMTN,G6PD,INPP5J,NANS,MVD,SQSTM1,STYK1,PC,ADCK2,EEF1A2,PTDSS1,TGFB1,PLCB2,MYLK4,DAPK2,NUDT14,TKT,PITPNM1,PEMT,SHB

GO:BP	regulation of monoatomic ion transmembrane transport	GO:0034765	0.001	2.9223100632651935	GAL,KCNE4,KCNV1,NTSR1,KCNA1,SCN5A,CEMIP,ITGB3,CCL2,KCNK12,KCNJ12,ADRA2A,STAC,THY1,KCNE1,SCN2B,KCNA6,KCNQ5,GJC2,G6PD,TGFB1,KCNK3,GRIN1
GO:BP	inorganic cation transmembrane transport	GO:0098662	0.001	2.9114343007119445	GAL,KCNE4,KCNV1,BHLHA15,NTSR1,SLC24A2,HTR2B,SLC47A1,KCNA1,SCN5A,CEMIP,ITGB3,TRPC5,SLC6A17,KCNK12,KCNJ12,ATP2A3,ADRA2A,STAC,THY1,KCNE1,SCN2B,ATP2B3,KCNA6,KCNQ5,GJC2,ATP1A3,G6PD,TGFB1,PLCB2,KCNK3,GRIN1,ATP6V0A1
GO:BP	regulation of metal ion transport	GO:0010959	0.001	2.869073382788957	GAL,KCNE4,OPRD1,NTSR1,RCVRN,KCNA1,SCN5A,CEMIP,ITGB3,CCL2,ADRA2A,STAC,THY1,KCNE1,SCN2B,PACSIN3,GJC2,G6PD,TGFB1,KCNK3,GRIN1
GO:BP	regulation of monoatomic ion transport	GO:0043269	0.001	2.8438890568816726	GAL,KCNE4,OPRD1,KCNV1,NTSR1,RCVRN,KCNA1,SCN5A,CEMIP,ITGB3,CCL2,KCNK12,KCNJ12,ADRA2A,STAC,THY1,KCNE1,SCN2B,KCNA6,PACSIN3,KCNQ5,GJC2,G6PD,TGFB1,KCNK3,GRIN1
GO:BP	inorganic ion transmembrane transport	GO:0098660	0.001	2.8282609169865824	GAL,KCNE4,KCNV1,BHLHA15,NTSR1,SLC24A2,HTR2B,SLC47A1,KCNA1,SCN5A,CEMIP,ITGB3,TRPC5,SLC6A17,GABRA5,KCNK12,KCNJ12,ATP2A3,ADRA2A,STAC,THY1,KCNE1,SCN2B,ATP2B3,KCNA6,SLC4A3,KCNQ5,GJC2,ATP1A3,G6PD,TGFB1,PLCB2,KCNK3,GRIN1,ATP6V0A1
GO:BP	tube morphogenesis	GO:0035239	0.002	2.7996484666280583	COL3A1,SIX1,CXCL8,COL8A1,NPR3,TGFBI,TBX3,GATA4,SERPINE1,MMP19,SP100,PLXND1,SFRP1,PPARG,DLC1,ITGB3,CHI3L1,CCL2,LOXL2,SPHK1,HTATIP2,THBS1,CARD10,ADM2,THY1,HMOX1,MICAL2,EGFL7,LDLR,KLF2,TNFAIP2,TGFB1,SHB
GO:BP	regulation of cell population proliferation	GO:0042127	0.002	2.764726570838976	GAL,SIX1,CXCL8,ACAN,PLA2G3,NPR3,ICAM1,TBX3,TNFRSF9,OSMR,HTR2B,TICAM1,PLXND1,CCKBR,SCN5A,DAAM2,SFRP1,PPARG,DLC1,ITGB3,P3H2,TRPC5,DHCR24,IL34,CCL2,OSGIN1,SPHK1,ETV5,SEMA3A,KIF26A,THBS1,ADRA2A,EGR4,FABP3,LZTS1,THY1,NR4A3,AVPR1A,PTPRN,HMOX1,SEZ6,MYD88,FABP6,IL12RB2,EGFL7,IRAK1,ICOSLG,BDNF,IGFBP3,ATP8A2,SSTR2,GJC2,DMTN,TP53I11,INPP5J,CHRNB2,MVD,KIAA0319,TGFB1,TANC2,SLC7A5
GO:BP	muscle contraction	GO:0006936	0.002	2.7517231813388463	UTS2,KCNE4,TBX3,GATA4,ADRA1B,HTR2B,KCNA1,SCN5A,ADRA2C,KCNJ12,SPHK1,ADRA2A,STAC,KCNE1,SCN2B,ATP8A2,SSTR2,CHRNB2,GAMT
GO:BP	regulation of MAPK cascade	GO:0043408	0.002	2.7439655638864084	ICAM1,GATA4,KL,CARTPT,ADRA1B,HGF,HTR2B,SFRP1,PPARG,ITGB3,TNFAIP8L3,SPRY4,ADRA2C,CHI3L1,IL34,CCL2,SPHK1,SPRED3,SEMA3A,THBS1,ADRA2A,HCRTR1,DOK5,MYD88,SH3RF3,IRAK1,IGFBP3,DUSP5
GO:BP	response to mechanical stimulus	GO:0009612	0.002	2.730099969317399	COL1A1,GATA4,KCNA1,PDE2A,PPARG,ITGB3,LHFPL5,CHI3L1,PHF24,THBS1,MYD88,ATP8A2,KIAA0319,TGFB1,TMEM120A
GO:BP	positive regulation of MAPK cascade	GO:0043410	0.002	2.7167232094935776	ICAM1,GATA4,KL,CARTPT,ADRA1B,HGF,HTR2B,ITGB3,TNFAIP8L3,ADRA2C,CHI3L1,IL34,CCL2,SPHK1,SEMA3A,THBS1,ADRA2A,HCRTR1,DOK5,MYD88,SH3RF3,IRAK1,IGFBP3

GO:BP	monoatomic cation transmembrane transport	GO:0098655	0.002	2.646485538732597	GAL,KCNE4,KCNV1,BHLHA15,NTSR1,SLC24A2,HTR2B,SLC47A1,KCNA1,SCN5A,CEMIP,ITGB3,TRPC5,SLC6A17,KCNK12,KCNJ12,ATP2A3,ADRA2A,STAC,THY1,KCNE1,SCN2B,ATP2B3,KCNA6,KCNQ5,GJC2,ATP1A3,G6PD,TGFB1,PLCB2,KCNK3,GRIN1,ATP6V0A1
GO:BP	nervous system development	GO:0007399	0.002	2.638363319088319	COL3A1,SIX1,ACAN,PLA2G3,HTR6,TBX3,BHLHA15,CPNE6,EGR2,KCNA1,TG,PLXND1,GDA,SCN5A,S100A10,DAAM2,SFRP1,USH1G,PPARG,DLC1,TRPC5,ESRP1,ADRA2C,LHFPL5,SLC6A17,IGSF21,IL34,GAS7,GABRA5,CCL2,SPHK1,CDHR1,CHST8,TMEM132E,ETV5,MARVELD1,SEMA3A,KIF26A,RNF112,B3GNT2,OPCML,LZTS1,ASTN2,THY1,DOK5,AVPR1A,KCNE1,SEZ6,SCN2B,MYD88,LDLR,BDNF,ATP8A2,SSTR2,GJC2,G6PD,INPP5J,CHRNB2,KIAA0319,TGFB1,KIRREL3,GRIN1,PITPNM1,TANC2,CABLES1,SLC7A5,MAP1A
GO:BP	positive regulation of response to stimulus	GO:0048584	0.003	2.5800464368770193	COL3A1,COL1A1,CXCL8,CMKLR1,PLA2G3,ICAM1,HTR6,GATA4,SERPINE1,C2CD4A,KL,CARTPT,F2RL2,ADRA1B,OSMR,HGF,HTR2B,TICAM1,A2M,DAAM2,SFRP1,PDE2A,PPARG,TRIM58,ITGB3,TNFAIP8L3,ADRA2C,CHI3L1,IL34,CCL2,SPHK1,PLEKHF1,SCUBE1,RSAD2,SEMA3A,THBS1,NFAM1,ADRA2A,HCRTR1,THY1,NR4A3,ULBP2,DOK5,HMOX1,MYD88,SH3RF3,LRRK1,IRAK1,LDLR,ICOSLG,BDNF,NLRC5,IGFBP3,KLF2,DMTN,IL10RA,KIAA0319,DAPK2,F12,GRIN1,SHB
GO:BP	negative regulation of cell migration	GO:0030336	0.003	2.557865143774407	COL3A1,TNFAIP6,SERPINE1,ARHGDIB,SP100,SFRP1,PPARG,DLC1,RIN3,CCL2,THBS1,CARD10,MMP28,THY1,HMOX1,DNAJA4,IGFBP3
GO:BP	phosphate-containing compound metabolic process	GO:0006796	0.003	2.500726965692756	OPRD1,PLA2G3,CARTPT,NTSR1,HGF,HTR2B,ACOT4,GDA,CEMIP,SFRP1,PDE2A,PPARG,DLC1,ITGB3,TRPC5,TNFAIP8L3,SPRY4,ADRA2C,CHI3L1,IL34,CCL2,SPHK1,HTATIP2,ME1,NQO1,CAMKV,THBS1,RCAN1,CARD10,ADM2,ADRA2A,CDK18,BEGAIN,FABP3,THY1,PCYT2,PTPRN,FAR2,ST3GAL1,IL12RB2,LRRK1,OSBPL10,IRAK1,LDLR,PITPNM2,BDNF,NLRC5,IGFBP3,PNPLA3,DUSP5,GJC2,DMTN,G6PD,INPP5J,MVD,SQSTM1,STYK1,PC,ADCK2,EEF1A2,PTDSS1,TGFB1,PLCB2,MYLK4,DAPK2,NUDT14,TKT,PITPNM1,PEMT,SHB
GO:BP	calcium ion transmembrane transport	GO:0070588	0.004	2.4354337931010734	BHLHA15,NTSR1,SLC24A2,HTR2B,SCN5A,CEMIP,ITGB3,TRPC5,ATP2A3,ADRA2A,STAC,THY1,KCNE1,ATP2B3,GJC2,G6PD,TGFB1,PLCB2,GRIN1
GO:BP	regulation of heart contraction	GO:0008016	0.004	2.379143583099905	KCNE4,GATA4,HSPB7,ADRA1B,SCN5A,KCNJ12,ATP2A3,ADM2,AVPR1A,KCNE1,SCN2B,ATP2B3,SLC4A3,ATP1A3
GO:BP	anatomical structure formation involved in morphogenesis	GO:0048646	0.004	2.376229756002723	COL3A1,COL1A1,SIX1,CXCL8,COL8A1,PLA2G3,NPR3,TGFBI,TBX3,GATA4,PAX1,SERPINE1,MMP19,SP100,EGR2,PLXND1,SFRP1,PPARG,DLC1,ITGB3,CHI3L1,CCL2,LOXL2,SPHK1,HTATIP2,THBS1,CARD10,ADM2,TEAD4,THY1,FHL2,HMOX1,EGFL7,KLF2,ATP8A2,DUSP5,TNFAIP2,TGFB1,SHB
GO:BP	positive regulation of molecular function	GO:0044093	0.004	2.361952264326575	GAL,RGS10,OPRD1,NPR3,IL32,CARTPT,NTSR1,SP100,HTR2B,TICAM1,KCNA1,PLXND1,S100A10,CEMIP,SFRP1,PPARG,TRIM58,DLC1,ITGB3,TNFAIP8L3,ADRA2C,CHI3L1,IL34,CCL2,SPHK1,ATP2A3,TRIM14,THBS1,NFAM1,CARD10,ADRA2A,STAC,THY1,AVPR1A,KCNE1,MYD88,IRAK1,BDNF,NPM2,ADAP1,EEF1A2,TGFB1,PLCB2,KCNK3,GRIN1,RASGEF1C

GO:BP	response to organic cyclic compound	GO:0014070	0.005	2.33085739	COL1A1,SIX1,HTR6,MMP19,KL,HTR2B,VGF,TICAM1,A2M,GPR83,SFRP1,PDE2A,PPARG,CHRM4,CCL2,NQO1,THBS1,HRH2,NR4A3,AVPR1A,KCNE1,EPHX1,MYD88,KLF2,PNPLA3,SSTR2,DMTN,ATP1A3,INSIG1,G6PD,CHRNB2,TGFB1,CYP1A1
GO:BP	cellular response to oxygen-containing compound	GO:1901701	0.005	2.3294095864678592	COL3A1,COL1A1,SIX1,CXCL8,RGS10,ICAM1,HTR6,GATA4,SERPINE1,CPNE6,HGF,HTR2B,TICAM1,SCN5A,SFRP1,PDE2A,PPARG,ITGB3,CHRM4,CCL2,SPHK1,NQO1,HRH2,ADRA2A,NR4A3,AVPR1A,KCNE1,PTPRN,MYD88,IRAK1,LDLR,KLF2,PNPLA3,SSTR2,DMTN,ATP1A3,INSIG1,CHRNB2,SQSTM1,TGFB1,SLC7A5
GO:BP	locomotion	GO:0040011	0.005	2.311622346616013	COL3A1,COL1A1,CXCL8,CMKLR1,TNFAIP6,ICAM1,SERPINE1,ARHGDIB,SP100,HGF,PLXND1,CEMIP,DAAM2,SFRP1,PPARG,DLC1,ITGB3,RIN3,IL34,CHRM4,CCL2,OSGIN1,SPHK1,SEMA3A,KIF26A,THBS1,RCAN1,CARD10,ADRA2A,MMP28,THY1,NR4A3,HMOX1,MYD88,DNAJA4,IGFBP3,DMTN,TGFB1,DAPK2,PLEKHG3
GO:BP	localization	GO:0051179	0.006	2.2520584810978663	COL1A1,GAL,SIX1,CXCL8,DYSF,TNFAIP6,KCNE4,OPRD1,FOLR3,PLA2G3,TGFBI,TBX3,KCNV1,BHLHA15,SERPINE1,CARTPT,F2RL2,NTSR1,SLC24A2,SP100,EGR2,HTR2B,VGF,RCVRN,SLC47A1,KCNA1,TG,CCKBR,SCN5A,S100A10,CEMIP,SFRP1,DYNC1I1,USH1G,PPARG,TRIM58,WIPF3,ITGB3,TRPC5,TNFAIP8L3,SLC2A6,DHCR24,ADRA2C,RIN3,LHFPL5,SLC6A17,GABRA5,PHYHIP,CCL2,ATP10A,KCNK12,KCNJ12,LOXL2,SPHK1,HTATIP2,ATP2A3,PLEKHF1,RSAD2,THBS1,HRH2,CARD10,ADAM11,ADRA2A,STAC,FABP3,SVOPL,ASTN2,THY1,NR4A3,AVPR1A,KCNE1,PTPRN,FRMPD1,SCN2B,MYD88,FABP6,ATP2B3,IPCEF1,SNX7,OSBPL10,LDLR,PITPNM2,BDNF,KCNA6,PEX5L,ATP8A2,SLC4A3,PACSIN3,KCNQ5,HYAL3,CHRNA4,ANXA11,GJC2,DMTN,SLC7A2,ATP1A3,INSIG1,G6PD,SHISA8,CHRNB2,TNFAIP2,SQSTM1,IL10RA,DOC2B,TGFB1,PLCB2,KCNK3,SLC45A1,GRIN1,TMEM120A,SPRN,ATP6V0A1,OLFM2,PITPNM1,SLCO4A1,TANC2,SLC7A5,MAP1A,SREBF2
GO:BP	chemical homeostasis	GO:0048878	0.006	2.235013538509496	ICAM1,GATA4,BHLHA15,KL,CARTPT,NTSR1,SLC24A2,HTR2B,VGF,PNPLA5,KCNA1,CCKBR,CEMIP,PPARG,ITGB3,TRPC5,ATP2A3,ADRA2A,HCRTR1,FABP3,THY1,AVPR1A,PTPRN,HMOX1,ATP2B3,LDLR,PNPLA3,SLC4A3,ATP1A3,INSIG1,TGFB1,PLCB2,GRIN1,ATP6V0A1,SREBF2
GO:BP	negative regulation of cell motility	GO:2000146	0.006	2.2340511613127005	COL3A1,TNFAIP6,SERPINE1,ARHGDIB,SP100,SFRP1,PPARG,DLC1,RIN3,CCL2,THBS1,CARD10,MMP28,THY1,HMOX1,DNAJA4,IGFBP3
GO:BP	regulation of gliogenesis	GO:0014013	0.006	2.2158611600059093	EGR2,DAAM2,PPARG,IL34,ETV5,RNF112,LDLR,GJC2,TGFB1,SLC7A5
GO:BP	negative regulation of locomotion	GO:0040013	0.006	2.2018925764222335	COL3A1,TNFAIP6,SERPINE1,ARHGDIB,SP100,SFRP1,PPARG,DLC1,RIN3,CCL2,SEMA3A,THBS1,CARD10,MMP28,THY1,HMOX1,DNAJA4,IGFBP3
GO:BP	cell migration	GO:0016477	0.007	2.1849271466922597	COL3A1,COL1A1,SIX1,CXCL8,CMKLR1,TNFAIP6,ICAM1,HTR6,SERPINE1,ARHGDIB,SP100,HGF,HTR2B,PLXND1,CEMIP,DAAM2,SFRP1,PPARG,DLC1,ITGB3,RIN3,IL34,CCL2,OSGIN1,LOXL2,SPHK1,SEMA3A,KIF26A,THBS1,CARD10,ADRA2A,MMP28,ASTN2,THY1,NR4A3,HMOX1,MYD88,DNAJA4,IGFBP3,DMTN,KIAA0319,TGFB1,DAPK2,KIRREL3,PLEKHG3

GO:BP	cell development	GO:0048468	0.008	2.1130754526907527	SIX1,TNFAIP6,ACAN,PLA2G3,ICAM1,TBX3,GATA4,BHLHA15,PAX1,RELB,TNFRSF9,CARTPT,CPNE6,EGR2,HTR2B,KCNA1,PLXND1,S100A10,DAAM2,SFRP1,PDE2A,USH1G,PPARG,TRIM58,TRPC5,LHFPL5,IL34,GAS7,GABRA5,CDHR1,DACT2,RSAD2,TMEM132E,ETV5,SEMA3A,KIF26A,NFAM1,RNF112,RCAN1,B3GNT2,OPCML,TFCP2L1,LZTS1,THY1,KCNE1,FHL2,SEZ6,MYD88,ST3GAL1,LRRK1,LDLR,BDNF,KLF2,ATP8A2,NPM2,FASN,GJC2,DMTN,G6PD,INPP5J,CHRNB2,KIAA0319,TGFB1,MKX,KIRREL3,TANC2,SHB,SLC7A5,MAP1A,RIMBP3
GO:BP	intracellular signal transduction	GO:0035556	0.008	2.071011056694599	COL3A1,GAL,CXCL8,CMKLR1,ICAM1,HTR6,GATA4,BHLHA15,RELB,KL,CARTPT,F2RL2,ARHGDIB,ADRA1B,SP100,HGF,HTR2B,TICAM1,CORO2A,SFRP1,PDE2A,PPARG,TRIM58,DLC1,ITGB3,TNFAIP8L3,SPRY4,PSD4,DHCR24,ADRA2C,CHI3L1,IL34,CCL2,SPHK1,ATP2A3,PLEKHF1,SPRED3,SEMA3A,THBS1,NFAM1,RCAN1,CARD10,ADRA2A,HCRTR1,TEAD4,NR4A3,DOK5,AVPR1A,FHL2,HMOX1,SEZ6,MYD88,SH3RF3,LRRK1,IRAK1,PITPNM2,IGFBP3,PEX5L,DUSP5,DMTN,SQSTM1,KIAA0319,PLCB2,DAPK2,GRIN1,PLEKHG3,RASGEF1C
GO:BP	regulation of intracellular signal transduction	GO:1902531	0.009	2.0253191781430697	COL3A1,CMKLR1,ICAM1,HTR6,GATA4,KL,CARTPT,F2RL2,ARHGDIB,ADRA1B,HGF,HTR2B,TICAM1,SFRP1,PDE2A,PPARG,TRIM58,DLC1,ITGB3,TNFAIP8L3,SPRY4,PSD4,ADRA2C,CHI3L1,IL34,CCL2,SPHK1,PLEKHF1,SPRED3,SEMA3A,THBS1,RCAN1,ADRA2A,HCRTR1,DOK5,FHL2,HMOX1,SEZ6,MYD88,SH3RF3,LRRK1,IRAK1,IGFBP3,PEX5L,DUSP5,SQSTM1,KIAA0319,DAPK2,PLEKHG3
GO:BP	cellular response to organic substance	GO:0071310	0.010855100284011944	1.9643661599947448	COL3A1,COL1A1,SIX1,CXCL8,CMKLR1,RGS10,MSC,ICAM1,HTR6,GATA4,BHLHA15,SERPINE1,SP100,EGR2,OSMR,HTR2B,TICAM1,SFRP1,PDE2A,PPARG,ITGB3,SPRY4,CHI3L1,IL34,CHRM4,CCL2,SPHK1,THBS1,HRH2,ADRA2A,HCRTR1,B3GNT2,NR4A3,AVPR1A,KCNE1,PTPRN,MYD88,IL12RB2,IRAK1,LDLR,NLRC5,KLF2,PNPLA3,FASN,SSTR2,HYAL3,PALM3,DMTN,ATP1A3,INSIG1,CHRNB2,IL10RA,TGFB1,CYP1A1,SLC7A5
GO:BP	tube development	GO:0035295	0.011	1.9469116161531084	COL3A1,SIX1,CXCL8,COL8A1,NPR3,TGFBI,TBX3,GATA4,SERPINE1,MMP19,SP100,PLXND1,CCKBR,SFRP1,PPARG,DLC1,ITGB3,CHI3L1,CCL2,LOXL2,SPHK1,HTATIP2,DACT2,THBS1,CARD10,ADM2,THY1,HMOX1,MICAL2,EGFL7,LDLR,KLF2,TNFAIP2,TGFB1,CYP1A1,SHB
GO:BP	regulation of molecular function	GO:0065009	0.011530891767218016	1.938137104274634	GAL,CMKLR1,RGS10,KCNE4,OPRD1,NPR3,PSMB8,SERPINE1,IL32,CARTPT,PSMB9,NTSR1,SP100,HGF,HTR2B,SERPINA3,TICAM1,KCNA1,PLXND1,A2M,S100A10,CEMIP,TIMP4,SFRP1,PPARG,TRIM58,DLC1,ITGB3,TNFAIP8L3,SPRY4,DHCR24,ADRA2C,CHI3L1,IL34,CCL2,SPHK1,ATP2A3,NQO1,TRIM14,THBS1,NFAM1,RCAN1,CARD10,ADRA2A,BEGAIN,STAC,THY1,AVPR1A,KCNE1,HMOX1,SCN2B,MYD88,IRAK1,BDNF,NLRC5,NPM2,ADAP1,SHISA8,EEF1A2,TGFB1,PLCB2,KCNK3,GRIN1,SHB,RASGEF1C
GO:BP	inflammatory response	GO:0006954	0.013	1.899489756112697	CXCL8,CMKLR1,TNFAIP6,PLA2G3,SERPINE1,C2CD4A,RELB,OSMR,HGF,SERPINA3,TICAM1,MGLL,A2M,PDE2A,PPARG,CHI3L1,IL34,CCL2,SPHK1,SCUBE1,THBS1,NFAM1,HMOX1,MYD88,LDLR,FASN,HYAL3,IL10RA,TGFB1,F12
GO:BP	regulation of extrinsic apoptotic signaling pathway via death domain receptors	GO:1902041	0.014	1.8586505196015009	ICAM1,SERPINE1,SP100,HGF,SFRP1,THBS1,HMOX1
GO:BP	positive regulation of gliogenesis	GO:0014015	0.013995319039695158	1.854017196833416	EGR2,PPARG,IL34,ETV5,RNF112,GJC2,TGFB1,SLC7A5

GO:BP	regulation of calcium ion transport	GO:0051924	0.015	1.8190921253716548	OPRD1,NTSR1,RCVRN,CEMIP,ITGB3,CCL2,ADRA2A,STAC,THY1,KCNE1,PACSIN3,GJC2,G6PD,TGFB1,GRIN1
GO:BP	positive regulation of signaling	GO:0023056	0.01622560768631936 2	1.7897990288959718	COL3A1,COL1A1,GAL,PLA2G3,ICAM1,HTR6,GATA4,KL,CARTPT,F2RL2,ADRA1B,SLC24A2,HGF,HTR2B,TICAM1,DAAM2,SFRP1,PPARG,TRIM58,ITGB3,TNFAIP8L3,ADRA2C,CHI3L1,IL34,CCL2,SPHK1,PLEKHF1,SCUBE1,RSAD2,SEMA3A,THBS1,NFAM1,ADRA2A,HCRTR1,DOK5,HMOX1,MYD88,SH3RF3,LRRK1,IRAK1,BDNF,NLRC5,IGFBP3,KLF2,SQSTM1,IL10RA,KIAA0319,DOC2B,GRIN1
GO:BP	positive regulation of cell communication	GO:0010647	0.016225607686319362	1.7897990288959718	COL3A1,COL1A1,GAL,PLA2G3,ICAM1,HTR6,GATA4,KL,CARTPT,F2RL2,ADRA1B,SLC24A2,HGF,HTR2B,TICAM1,DAAM2,SFRP1,PPARG,TRIM58,ITGB3,TNFAIP8L3,ADRA2C,CHI3L1,IL34,CCL2,SPHK1,PLEKHF1,SCUBE1,RSAD2,SEMA3A,THBS1,NFAM1,ADRA2A,HCRTR1,DOK5,HMOX1,MYD88,SH3RF3,LRRK1,IRAK1,BDNF,NLRC5,IGFBP3,KLF2,SQSTM1,IL10RA,KIAA0319,DOC2B,GRIN1
GO:BP	positive regulation of intracellular signal transduction	GO:1902533	0.018280168004776682	1.7380198171792027	COL3A1,ICAM1,HTR6,GATA4,KL,CARTPT,F2RL2,ADRA1B,HGF,HTR2B,TICAM1,PPARG,TRIM58,ITGB3,TNFAIP8L3,ADRA2C,CHI3L1,IL34,CCL2,SPHK1,PLEKHF1,SEMA3A,THBS1,ADRA2A,HCRTR1,DOK5,HMOX1,MYD88,SH3RF3,LRRK1,IRAK1,IGFBP3,KIAA0319
GO:BP	positive regulation of monoatomic ion transport	GO:0043270	0.020946487500379238	1.678888793084194	GAL,NTSR1,KCNA1,SCN5A,CEMIP,CCL2,ADRA2A,STAC,THY1,KCNE1,GJC2,G6PD,KCNK3,GRIN1
GO:BP	response to stress	GO:0006950	0.021	1.6689447712113954	COL3A1,COL1A1,GAL,CXCL8,CMKLR1,TNFAIP6,OPRD1,PLA2G3,NPR3,TBX3,GATA4,BHLHA15,SERPINE1,HSPB7,IL32,C2CD4A,RELB,CARTPT,F2RL2,SP100,OSMR,HGF,VGF,SERPINA3,TICAM1,MGLL,A2M,S100A10,SFRP1,PDE2A,PPARG,TRIM58,ITGB3,DHCR24,ADRA2C,CHI3L1,IL34,GPX3,GABRA5,SRXN1,CCL2,LOXL2,SPHK1,ATP2A3,SCUBE1,NQO1,TRIM14,RSAD2,ETV5,SEMA3A,THBS1,NFAM1,RNF112,RCAN1,ADAM11,ADRA2A,STAC,THY1,ULBP2,AVPR1A,PTPRN,HMOX1,SCN2B,MYD88,IPCEF1,SH3RF3,IRAK1,LDLR,DNAJA4,ICOSLG,NLRC5,KLF2,FASN,SSTR2,HYAL3,CHRNA4,DMTN,INSIG1,G6PD,CHRNB2,SQSTM1,IL10RA,KIAA0319,TGFB1,F12,KCNK3,CYP1A1,SLC7A5,SREBF2
GO:BP	vascular process in circulatory system	GO:0003018	0.023	1.6394733717935501	UTS2,C2CD4A,ADRA1B,HTR2B,PDE2A,ADRA2C,SLC6A17,ATP2A3,HRH2,ADRA2A,AVPR1A,KLF2,SLC4A3,SLC7A2,SLC7A5
GO:BP	positive regulation of transmembrane transport	GO:0034764	0.023098318770282636	1.6364196294308275	GAL,NTSR1,KCNA1,CEMIP,CCL2,STAC,THY1,NR4A3,KCNE1,GJC2,G6PD,KCNK3,GRIN1,SLC7A5
GO:BP	tissue development	GO:0009888	0.024	1.6233902313023374	COL3A1,COL1A1,GAL,SIX1,COL8A1,ACAN,MSC,KRT80,ICAM1,TGFBI,TBX3,GATA4,BHLHA15,PAX1,SERPINE1,KL,EGR2,HGF,HTR2B,PLXND1,SCN5A,SFRP1,PDE2A,PPARG,DLC1,ITGB3,DHCR24,ESRP1,LHFPL5,CHI3L1,ETV4,HOXC13,LOXL2,SCUBE1,DACT2,TMEM132E,SPRED3,SEMA3A,RCAN1,TFCP2L1,ASTN2,KCNE1,FHL2,MYD88,MICAL2,KLF2,DUSP5,FASN,HYAL3,G6PD,TGFB1,MAFF,CYP1A1,TAGLN2

GO:BP	regulation of monoatomic cation transmembrane transport	GO:1904062	0.02416389591986 3714	1.6168330435569649	GAL,KCNE4,NTSR1,KCNA1,SCN5A,CEMIP,ITGB3,ADRA2A,STAC,THY1,KCNE1,SCN2B,GJC2,G6PD,TGFB1,KCNK3,GRIN1
GO:BP	MAPK cascade	GO:0000165	0.02432124067280 1916	1.6140142746456663	ICAM1,GATA4,KL,CARTPT,ADRA1B,HGF,HTR2B,SFRP1,PPARG,ITGB3,TNFAIP8L3,SPRY4,ADRA2C,CHI3L1,IL34,CCL2,SPHK1,SPRED3,SEMA3A,THBS1,ADRA2A,HCRTR1,DOK5,MYD88,SH3RF3,IRAK1,IGFBP3,DUSP5
GO:BP	response to organonitrogen compound	GO:0010243	0.02438905215193 7204	1.612805077635522	COL3A1,COL1A1,GAL,SIX1,RGS10,ICAM1,HTR6,MMP19,KL,EGR2,HTR2B,VGF,TICAM1,SFRP1,PDE2A,PPARG,ITGB3,CHRM4,NQO1,HRH2,NR4A3,KCNE1,MYD88,LDLR,KLF2,PNPLA3,DMTN,ATP1A3,INSIG1,CHRNB2,TGFB1,GRIN1,SLC7A5,SREBF2
GO:BP	heart contraction	GO:0060047	0.025	1.5944240118937119	KCNE4,GATA4,HSPB7,ADRA1B,SCN5A,KCNJ12,ATP2A3,ADM2,AVPR1A,KCNE1,SCN2B,ATP2B3,SLC4A3,ATP1A3
GO:BP	positive regulation of monoatomic ion transmembrane transport	GO:0034767	0.02658310370081 9503	1.5753943145351845	GAL,NTSR1,KCNA1,CEMIP,CCL2,STAC,THY1,KCNE1,GJC2,G6PD,KCNK3,GRIN1
GO:BP	neurogenesis	GO:0022008	0.02703093653646 5197	1.56813891	COL3A1,SIX1,ACAN,PLA2G3,BHLHA15,CPNE6,EGR2,KCNA1,PLXND1,S100A10,DAAM2,SFRP1,USH1G,PPARG,TRPC5,ESRP1,ADRA2C,LHFPL5,IL34,GAS7,GABRA5,CCL2,CDHR1,TMEM132E,ETV5,SEMA3A,KIF26A,RNF112,B3GNT2,OPCML,LZTS1,ASTN2,THY1,DOK5,SEZ6,MYD88,LDLR,BDNF,ATP8A2,GJC2,INPP5J,CHRNB2,KIAA0319,TGFB1,KIRREL3,TANC2,SLC7A5,MAP1A
GO:BP	positive regulation of signal transduction	GO:0009967	0.02710714576540 0145	1.566916208852377	COL3A1,COL1A1,ICAM1,HTR6,GATA4,KL,CARTPT,F2RL2,ADRA1B,HGF,HTR2B,TICAM1,DAAM2,SFRP1,PPARG,TRIM58,ITGB3,TNFAIP8L3,ADRA2C,CHI3L1,IL34,CCL2,SPHK1,PLEKHF1,SCUBE1,RSAD2,SEMA3A,THBS1,NFAM1,ADRA2A,HCRTR1,DOK5,HMOX1,MYD88,SH3RF3,LRRK1,IRAK1,BDNF,NLRC5,IGFBP3,KLF2,IL10RA,KIAA0319,GRIN1
GO:BP	behavior	GO:0007610	0.033	1.481073327945488	GAL,OPRD1,CARTPT,NTSR1,SLC24A2,EGR2,HTR2B,GPR83,GABRA5,THBS1,RCAN1,ADM2,ADAM11,HCRTR1,NR4A3,AVPR1A,SEZ6,LDLR,BDNF,ATP8A2,CHRNA4,CHRNB2,KIRREL3,GRIN1,MAP1A
GO:BP	positive regulation of cation transmembrane transport	GO:1904064	0.037	1.4313347912831202	GAL,NTSR1,KCNA1,CEMIP,STAC,THY1,KCNE1,GJC2,G6PD,KCNK3,GRIN1
GO:BP	regulation of leukocyte chemotaxis	GO:0002688	0.03755552856415 9966	1.4253261212917452	CXCL8,CMKLR1,TNFAIP6,SERPINE1,RIN3,IL34,CCL2,THBS1,MMP28,DAPK2

GO:BP	regulation of neurogenesis	GO:0050767	0.039	1.4066413050907096	ACAN,EGR2,PLXND1,S100A10,DAAM2,PPARG,TRPC5,IL34,ETV5,SEMA3A,RNF112,THY1,LDLR,BDNF,GJC2,KIAA0319,TGFB1,SLC7A5
GO:BP	G protein-coupled receptor signaling pathway, coupled to cyclic nucleotide second messenger	GO:0007187	0.039417784743498704	1.40430779	OPRD1,HTR6,HTR2B,CHRM4,CCL2,HRH2,SSTR2
GO:BP	collagen metabolic process	GO:0032963	0.041871071031726444	1.3780859300278125	COL1A1,MMP1,MMP19,ADAMTS14,PPARG,P3H2,MMP11,MMP28,TGFB1
GO:BP	response to external stimulus	GO:0009605	0.043	1.3625218927669651	COL1A1,CXCL8,CMKLR1,TNFAIP6,PLA2G3,GATA4,BHLHA15,SERPINE1,C2CD4A,RELB,KL,CARTPT,NTSR1,SLC24A2,SP100,OSMR,HGF,VGF,TICAM1,MGLL,RCVRN,KCNA1,A2M,SFRP1,PDE2A,PPARG,TRIM58,ITGB3,RIN3,LHFPL5,CHI3L1,IL34,CCL2,PHF24,SPHK1,NQO1,TRIM14,RSAD2,SEMA3A,KIF26A,THBS1,MMP28,ULBP2,AVPR1A,MYD88,IL12RB2,IRAK1,LDLR,NLRC5,ATP8A2,SSTR2,HYAL3,PALM3,DMTN,G6PD,IL10RA,KIAA0319,TGFB1,DAPK2,F12,TMEM229B,TMEM120A,PITPNM1,CYP1A1,SLC7A5,SREBF2
GO:BP	heart process	GO:0003015	0.044468717501550174	1.3519453956248577	KCNE4,GATA4,HSPB7,ADRA1B,SCN5A,KCNJ12,ATP2A3,ADM2,AVPR1A,KCNE1,SCN2B,ATP2B3,SLC4A3,ATP1A3
GO:BP	regulation of angiogenesis	GO:0045765	0.048	1.3213814471206322	CXCL8,GATA4,SERPINE1,SP100,PLXND1,SFRP1,PPARG,ITGB3,CHI3L1,SPHK1,HTATIP2,THBS1,ADM2,HMOX1,KLF2
GO:CC	cell periphery	GO:0071944	4.537794387920346e-12	11.343155186330023	COL3A1,COL1A1,DYSF,COL8A1,CMKLR1,PRRG2,RGS10,ACAN,KCNE4,OPRD1,PLA2G3,NPR3,COL5A3,ICAM1,TGFBI,HTR6,MMP1,KCNV1,SERPINE1,SYNPO,MMP19,TNFRSF9,KL,F2RL2,NTSR1,ADRA1B,SLC24A2,CPNE6,PTH2R,OSMR,PDYN,HTR2B,OR2W3,SERPINA3,MGLL,SLC47A1,KCNA1,ADAMTS14,PLXND1,CCKBR,SCN5A,A2M,S100A10,CEMIP,TIMP4,GPR83,SFRP1,PDE2A,USH1G,DLC1,EMILIN3,ITGB3,P3H2,TRPC5,TNFAIP8L3,SLC2A6,ADAMTSL3,LY6E,SPRY4,PSD4,MMP11,KNCN,ADRA2C,LHFPL5,SLC6A17,CHI3L1,IGSF21,GAS7,CHRM4,TPBGL,GABRA5,ATP10A,SHROOM1,LYPD6B,KCNJ12,LOXL2,SPHK1,CDHR1,SCUBE1,PARVB,EVC2,CAMKV,SPRED3,MARVELD1,THBS1,NFAM1,HRH2,ADAM11,DCHS2,ADRA2A,SPTB,MMP28,HCRTR1,OPCML,STAC,ANGPTL1,LZTS1,ASTN2,THY1,ULBP2,AVPR1A,LRFN2,KCNE1,PTPRN,PDLIM4,FRMPD1,SEZ6,SCN2B,MYD88,ATP2B3,IPCEF1,IL12RB2,EGFL7,IRAK1,LDLR,ICOSLG,AHNAK2,KCNA6,ATP8A2,SLC4A3,PACSIN3,FASN,SSTR2,KCNQ5,HYAL3,CHRNA4,PALM3,ANXA11,GJC2,DMTN,SLC7A2,ATP1A3,ADAP1,G6PD,SHISA8,INPP5J,CHRNB2,TNFAIP2,STYK1,IL10RA,KIAA0319,DOC2B,TGFB1,F12,KCNK3,KIRREL3,GRIN1,TMEM120A,SPRN,ATP6V0A1,OLFM2,PITPNM1,SLCO4A1,SHB,EMID1,RASGEF1C,SLC7A5
GO:CC	somatodendritic compartment	GO:0036477	3.447511315830519e-8	7.462494299656917	GAL,OPRD1,HTR6,SYNPO,NTSR1,CPNE6,PDYN,HTR2B,VGF,RCVRN,KCNA1,TRPC5,KNCN,RIN3,CHRM4,GABRA5,NQO1,SEMA3A,RNF112,HRH2,ADAM11,ADRA2A,LZTS1,ASTN2,THY1,PTPRN,PDLIM4,SEZ6,LDLR,CHRNA4,GJC2,ATP1A3,SHISA8,INPP5J,TUBB4A,TGFB1,KIRREL3,GRIN1,TANC2,MAP1A

GO:CC	synapse	GO:0045202	3.566091815320672e-8	7.447807479339634	UTS2,DYSF,RGS10,ACAN,OPRD1,HTR6,SYNPO,RELB,CARTPT,NTSR1,PDYN,HTR2B,VGF,KCNA1,PLXND1,PDE2A,ITGB3,SLC6A17,IGSF21,CHRM4,GABRA5,SPHK1,NQO1,CAMKV,RNF112,HRH2,ADAM11,ADRA2A,SPTB,HCRTR1,BEGAIN,LZTS1,LRFN2,PTPRN,PDLIM4,SEZ6,SCN2B,ATP2B3,BDNF,KCNA6,KCNQ5,CHRNA4,DMTN,ATP1A3,SHISA8,CHRNB2,EEF1A2,DOC2B,KCNK3,KIRREL3,GRIN1,ATP6V0A1,OLFM2,TANC2,MAP1A
GO:CC	extracellular matrix	GO:0031012	7.893266264283403e-8	7.102743246851471	COL3A1,COL1A1,COL8A1,ACAN,COL5A3,ICAM1,TGFBI,MMP1,SERPINE1,MMP19,SERPINA3,ADAMTS14,A2M,S100A10,TIMP4,SFRP1,EMILIN3,P3H2,ADAMTSL3,MMP11,CHI3L1,LOXL2,THBS1,ADAM11,MMP28,ANGPTL1,EGFL7,ANXA11,TGFB1,F12,EMID1
GO:CC	external encapsulating structure	GO:0030312	8.250491426216195e-8	7.083520182681866	COL3A1,COL1A1,COL8A1,ACAN,COL5A3,ICAM1,TGFBI,MMP1,SERPINE1,MMP19,SERPINA3,ADAMTS14,A2M,S100A10,TIMP4,SFRP1,EMILIN3,P3H2,ADAMTSL3,MMP11,CHI3L1,LOXL2,THBS1,ADAM11,MMP28,ANGPTL1,EGFL7,ANXA11,TGFB1,F12,EMID1
GO:CC	membrane	GO:0016020	2E-06	5.79203043652266665	DYSF,CMKLR1,PRRG2,RGS10,KCNE4,OPRD1,FOLR3,PLA2G3,NPR3,ICAM1,TGFBI,HTR6,KCNV1,TMEM171,SERPINE1,SYNPO,IL32,TNFRSF9,KL,F2RL2,ARHGDIB,NTSR1,ADRA1B,SLC24A2,CPNE6,PTH2R,OSMR,PDYN,HGF,HTR2B,OR2W3,PNPLA5,TICAM1,MGLL,RCVRN,SLC47A1,KCNA1,PLXND1,CCKBR,SCN5A,S100A10,GBGT1,CEMIP,GPR83,SFRP1,PDE2A,USH1G,DLC1,ITGB3,TRPC5,TNFAIP8L3,SLC2A6,SYNDIG1L,LY6E,SPRY4,PSD4,KNCN,DHCR24,ADRA2C,LHFPL5,SLC6A17,IGSF21,GAS7,CHRM4,TPBGL,GABRA5,FAM163B,SRXN1,ATP10A,SHROOM1,LYPD6B,KCNK12,ARSJ,KCNJ12,LOXL2,SPHK1,CDHR1,HTATIP2,ATP2A3,PLEKHF1,SCUBE1,TMEM54,TRIM14,PARVB,EVC2,CAMKV,CHST8,RSAD2,TMEM132E,SPRED3,TMEM179,MARVELD1,THBS1,NFAM1,RNF112,HRH2,ADAM11,DCHS2,ADRA2A,SPTB,HCRTR1,BEGAIN,B3GNT2,OPCML,STAC,TFCP2L1,SVOPL,LZTS1,ASTN2,THY1,ULBP2,AVPR1A,DHCR7,LRFN2,PCYT2,KCNE1,PTPRN,CLEC2L,PDLIM4,HMOX1,FRMPD1,FAR2,SEZ6,EPHX1,SCN2B,MYD88,FABP6,ATP2B3,ST3GAL1,TMTC1,IPCEF1,SNX7,IL12RB2,EXTL1,OSBPL10,IRAK1,FNDC9,GALNT9,LDLR,DNAJA4,ICOSLG,PITPNM2,AHNAK2,KCNA6,PNPLA3,PEX5L,ATP8A2,TMEM151A,SLC4A3,PACSIN3,FASN,SSTR2,KCNQ5,HYAL3,CHRNA4,PALM3,ANXA11,GJC2,DMTN,SLC7A2,TP53I11,ATP1A3,ADAP1,INSIG1,G6PD,SHISA8,INPP5J,CHRNB2,SYT16,STYK1,IL10RA,ADCK2,EEF1A2,KIAA0319,PTDSS1,DOC2B,MYLK4,DAPK2,F12,KCNK3,KIRREL3,SLC45A1,GRIN1,TMEM229B,TMEM120A,SPRN,ATP6V0A1,OLFM2,TKT,PITPNM1,PEMT,SLCO4A1,SUSD5,CYP1A1,SHB,RASGEF1C,SLC7A5,SREBF2
GO:CC	plasma membrane	GO:0005886	2E-06	5.664734922000383	DYSF,CMKLR1,PRRG2,RGS10,KCNE4,OPRD1,PLA2G3,NPR3,ICAM1,TGFBI,HTR6,KCNV1,SERPINE1,SYNPO,TNFRSF9,KL,F2RL2,NTSR1,ADRA1B,SLC24A2,CPNE6,PTH2R,OSMR,PDYN,HTR2B,OR2W3,MGLL,SLC47A1,KCNA1,PLXND1,CCKBR,SCN5A,S100A10,CEMIP,GPR83,SFRP1,PDE2A,USH1G,DLC1,ITGB3,TRPC5,TNFAIP8L3,SLC2A6,LY6E,SPRY4,PSD4,KNCN,ADRA2C,LHFPL5,SLC6A17,IGSF21,GAS7,CHRM4,TPBGL,GABRA5,ATP10A,SHROOM1,LYPD6B,KCNJ12,SPHK1,CDHR1,SCUBE1,PARVB,EVC2,CAMKV,SPRED3,MARVELD1,THBS1,NFAM1,HRH2,ADAM11,DCHS2,ADRA2A,SPTB,HCRTR1,OPCML,STAC,LZTS1,THY1,ULBP2,AVPR1A,LRFN2,KCNE1,PTPRN,PDLIM4,FRMPD1,SEZ6,SCN2B,MYD88,ATP2B3,IPCEF1,IL12RB2,IRAK1,LDLR,ICOSLG,AHNAK2,KCNA6,ATP8A2,SLC4A3,PACSIN3,FASN,SSTR2,KCNQ5,HYAL3,CHRNA4,PALM3,GJC2,DMTN,SLC7A2,ATP1A3,ADAP1,G6PD,SHISA8,INPP5J,CHRNB2,STYK1,IL10RA,KIAA0319,DOC2B,F12,KCNK3,KIRREL3,GRIN1,TMEM120A,SPRN,ATP6V0A1,OLFM2,PITPNM1,SLCO4A1,SHB,RASGEF1C,SLC7A5
GO:CC	cell body	GO:0044297	2E-06	5.616127149970755	GAL,SYNPO,NTSR1,CPNE6,PDYN,VGF,RCVRN,KCNA1,PLXND1,TRPC5,KNCN,RIN3,GABRA5,NQO1,TMEM132E,RNF112,ADAM11,ADRA2A,ASTN2,THY1,PTPRN,SEZ6,CHRNA4,GJC2,ATP1A3,TUBB4A,TGFB1,PITPNM1,MAP1A

GO:CC	cell junction	GO:0030054	8E-06	5.105001020545977	UTS2,DYSF,RGS10,ACAN,OPRD1,ICAM1,HTR6,SYNPO,RELB,CARTPT,NTSR1,PDYN,HTR2B,VGF,KCNA1,PLXND1,SCN5A,PDE2A,DLC1,ITGB3,SPRY4,SLC6A17,IGSF21,CHRM4,GABRA5,SHROOM1,SPHK1,NQO1,PARVB,CAMKV,TLE2,RNF112,HRH2,ADAM11,ADRA2A,SPTB,HCRTR1,BEGAIN,LZTS1,THY1,LRFN2,PTPRN,FHL2,PDLIM4,SEZ6,SCN2B,ATP2B3,BDNF,KCNA6,KCNQ5,CHRNA4,GJC2,DMTN,SLC7A2,ATP1A3,SHISA8,CHRNB2,EEF1A2,DOC2B,KCNK3,KIRREL3,GRIN1,ATP6V0A1,OLFM2,TANC2,MAP1A
GO:CC	neuronal cell body	GO:0043025	1E-05	4.9737162688102865	GAL,SYNPO,NTSR1,CPNE6,PDYN,VGF,RCVRN,KCNA1,TRPC5,KNCN,RIN3,GABRA5,NQO1,RNF112,ADAM11,ADRA2A,ASTN2,THY1,PTPRN,SEZ6,CHRNA4,GJC2,ATP1A3,TUBB4A,TGFB1,MAP1A
GO:CC	collagen-containing extracellular matrix	GO:0062023	3E-05	4.595131194622257	COL3A1,COL1A1,COL8A1,ACAN,COL5A3,ICAM1,TGFBI,SERPINE1,SERPINA3,A2M,S100A10,SFRP1,EMILIN3,P3H2,LOXL2,THBS1,ADAM11,MMP28,ANGPTL1,EGFL7,ANXA11,TGFB1,F12
GO:CC	neuron projection	GO:0043005	8E-05	4.091135458430855	OPRD1,HTR6,SYNPO,NTSR1,CPNE6,PDYN,HTR2B,RCVRN,KCNA1,PLXND1,USH1G,TRPC5,KNCN,RIN3,LHFPL5,CHRM4,GABRA5,CDHR1,NQO1,SEMA3A,RNF112,HRH2,ADAM11,ADRA2A,LZTS1,THY1,PTPRN,PDLIM4,SEZ6,ATP2B3,KCNA6,SSTR2,KCNQ5,CHRNA4,GJC2,ATP1A3,SHISA8,INPP5J,TUBB4A,TGFB1,KIRREL3,GRIN1,TANC2,MAP1A
GO:CC	dendrite	GO:0030425	2E-04	3.6842265443065263	OPRD1,HTR6,SYNPO,NTSR1,CPNE6,PDYN,HTR2B,RCVRN,KCNA1,TRPC5,RIN3,CHRM4,GABRA5,NQO1,SEMA3A,HRH2,LZTS1,THY1,PDLIM4,SEZ6,CHRNA4,SHISA8,INPP5J,KIRREL3,GRIN1,TANC2,MAP1A
GO:CC	dendritic tree	GO:0097447	2E-04	3.657353619512339	OPRD1,HTR6,SYNPO,NTSR1,CPNE6,PDYN,HTR2B,RCVRN,KCNA1,TRPC5,RIN3,CHRM4,GABRA5,NQO1,SEMA3A,HRH2,LZTS1,THY1,PDLIM4,SEZ6,CHRNA4,SHISA8,INPP5J,KIRREL3,GRIN1,TANC2,MAP1A
GO:CC	plasma membrane region	GO:0098590	2E-04	3.6384578459295085	KCNE4,OPRD1,KL,F2RL2,ADRA1B,OSMR,SLC47A1,KCNA1,PLXND1,SCN5A,CEMIP,PDE2A,DLC1,ITGB3,SPRY4,PSD4,KNCN,LHFPL5,IGSF21,CHRM4,GABRA5,SHROOM1,SPHK1,CDHR1,EVC2,ADAM11,ADRA2A,THY1,LRFN2,KCNE1,PDLIM4,ATP2B3,LDLR,KCNQ5,CHRNA4,DMTN,ATP1A3,SHISA8,CHRNB2,IL10RA,GRIN1,PITPNM1,SLC7A5
GO:CC	extracellular region	GO:0005576	4E-04	3.4231662635583846	COL3A1,COL1A1,GAL,UTS2,CXCL8,DYSF,COL8A1,PRRG2,TNFAIP6,ACAN,FOLR3,PLA2G3,NPR3,COL5A3,ICAM1,TGFBI,MMP1,PSMB8,SERPINE1,IL32,MMP19,KL,CARTPT,F2RL2,ARHGDIB,PSMB9,CPNE6,OOSP1,CES5A,PDYN,HGF,VGF,SERPINA3,ADAMTS14,TG,A2M,SPR,S100A10,CEMIP,TIMP4,DAAM2,SFRP1,EMILIN3,ITGB3,ADAMTSL3,LY6E,MMP11,CHI3L1,IL34,GPX3,CCL2,LYPD6B,ARSJ,LOXL2,CBR3,SCUBE1,TLE2,SEMA3A,THBS1,ADM2,MMP28,OPCML,ANGPTL1,FABP3,NXPH3,THY1,ULBP2,HMOX1,ATP2B3,ST3GAL1,EGFL7,LDLR,ICOSLG,BDNF,IGFBP3,PACSIN3,FASN,HYAL3,ANXA11,ATP1A3,G6PD,NANS,ACAT2,THEM6,TNFAIP2,SQSTM1,TUBB4A,TGFB1,F12,KIRREL3,GRIN1,SPRN,ATP6V0A1,OLFM2,TKT,EMID1,SLC7A5,TAGLN2

GO:CC	endomembrane system	GO:0012505	8E-04	3.082514969103893	COL3A1,COL1A1,GAL,DYSF,COL8A1,TNFAIP6,ACAN,OPRD1,FOLR3,PLA2G3,COL5A3,TGFBI,SERPINE1,SYNPO,CARTPT,NTSR1,ADRA1B,CPNE6,PDYN,HGF,VGF,SERPINA3,TICAM1,MGLL,KCNA1,SCN5A,A2M,GBGT1,CEMIP,PDE2A,DYNC1I1,DLC1,WIPF3,ITGB3,P3H2,SYNDIG1L,MMP11,DHCR24,ADRA2C,RIN3,SLC6A17,CHI3L1,SRXN1,ATP10A,ARSJ,LOXL2,SPHK1,HTATIP2,ATP2A3,PLEKHF1,CHST8,RSAD2,P4HA3,THBS1,NFAM1,RNF112,B3GNT2,ASTN2,THY1,ULBP2,AVPR1A,DHCR7,PCYT2,PTPRN,PDLIM4,HMOX1,SEZ6,EPHX1,MYD88,HS3ST1,ST3GAL1,TMTC1,SNX7,EXTL1,IRAK1,GALNT9,LDLR,PITPNM2,BDNF,IGFBP3,PNPLA3,ATP8A2,PACSIN3,FASN,HYAL3,ANXA11,DMTN,ATP1A3,INSIG1,SQSTM1,KIAA0319,PTDSS1,TGFB1,PLCB2,DAPK2,F12,KIRREL3,GRIN1,TMEM120A,ATP6V0A1,TKT,PITPNM1,PEMT,CYP1A1,EMID1,SREBF2
GO:CC	presynapse	GO:0098793	0.001	2.938972317243995	DYSF,OPRD1,NTSR1,PDYN,KCNA1,PDE2A,SLC6A17,IGSF21,GABRA5,SPHK1,RNF112,ADAM11,ADRA2A,LRFN2,PTPRN,ATP2B3,BDNF,KCNA6,KCNQ5,CHRNB2,DOC2B,KIRREL3,GRIN1,ATP6V0A1
GO:CC	axon	GO:0030424	0.004	2.4476152484826827	OPRD1,NTSR1,CPNE6,PDYN,KCNA1,PLXND1,TRPC5,RIN3,SEMA3A,ADAM11,ADRA2A,THY1,PTPRN,ATP2B3,KCNA6,KCNQ5,GJC2,ATP1A3,INPP5J,TUBB4A,TGFB1,KIRREL3,GRIN1,TANC2,MAP1A
GO:CC	cell projection	GO:0042995	0.009	2.063013925185654	OPRD1,HTR6,TBX3,SYNPO,NTSR1,CPNE6,PDYN,HTR2B,RCVRN,KCNA1,PLXND1,GPR83,USH1G,DLC1,ITGB3,TRPC5,SPRY4,PSD4,KNCN,RIN3,LHFPL5,SLC6A17,CHRM4,GABRA5,CDHR1,NQO1,PARVB,EVC2,SEMA3A,RNF112,HRH2,ADAM11,ADRA2A,SPTB,LZTS1,THY1,PTPRN,PDLIM4,SEZ6,ATP2B3,KCNA6,ATP8A2,SSTR2,KCNQ5,HYAL3,CHRNA4,GJC2,DMTN,ATP1A3,SHISA8,INPP5J,SQSTM1,TUBB4A,TGFB1,KIRREL3,GRIN1,TANC2,SLC7A5,MAP1A
GO:CC	axon terminus	GO:0043679	0.013	1.8812561039769735	OPRD1,NTSR1,PDYN,KCNA1,ADRA2A,PTPRN,KCNA6,KCNQ5,GRIN1
GO:CC	plasma membrane bounded cell projection	GO:0120025	0.015502353402398647	1.8096023668560548	OPRD1,HTR6,TBX3,SYNPO,NTSR1,CPNE6,PDYN,HTR2B,RCVRN,KCNA1,PLXND1,GPR83,USH1G,DLC1,ITGB3,TRPC5,SPRY4,PSD4,KNCN,RIN3,LHFPL5,CHRM4,GABRA5,CDHR1,NQO1,PARVB,EVC2,SEMA3A,RNF112,HRH2,ADAM11,ADRA2A,LZTS1,THY1,PTPRN,PDLIM4,SEZ6,ATP2B3,KCNA6,SSTR2,KCNQ5,HYAL3,CHRNA4,GJC2,DMTN,ATP1A3,SHISA8,INPP5J,SQSTM1,TUBB4A,TGFB1,KIRREL3,GRIN1,TANC2,SLC7A5,MAP1A
GO:CC	synaptic membrane	GO:0097060	0.025407020877605307	1.5950462555426157	OPRD1,KCNA1,PDE2A,ITGB3,IGSF21,CHRM4,GABRA5,ADAM11,ADRA2A,LRFN2,PDLIM4,ATP2B3,KCNQ5,CHRNA4,SHISA8,CHRNB2,GRIN1
GO:CC	perikaryon	GO:0043204	0.032	1.4893284969622345	SYNPO,NTSR1,CPNE6,RCVRN,KCNA1,RNF112,ADAM11,ASTN2,PTPRN,GJC2
GO:CC	neuron projection terminus	GO:0044306	0.035	1.4568952847001433	OPRD1,NTSR1,PDYN,KCNA1,ADRA2A,PTPRN,KCNA6,KCNQ5,GRIN1

Suspended GO Terms Based on Uprgeulated Genes

source	***term_name***	***term_id***	***adj_p_value***	***neg_log10_adj_p_value***	***intersections***
GO:MF	DNA-binding transcription factor activity, RNA polymerase II-specific	GO:0000981	2E-05	4.675023471790667	PTF1A,GSX1,PAX3,EN2,OLIG3,PAX7,HESX1,SP9,LHX5,FOXB1,ZIC4,SOX13,IRX5,DMBX1,TFAP2B,THRB,ZIC5,ZIC3,ZNF83,GBX2,ZIC1,EBF1,ZNF117,FOXP2,MEIS1,EBF2,PROX1,MAF,NR2F2,HES5,IRX2,RORA,PLAG1,RFX3,IRX1,ZIC2,YY2,NR2F1,ZNF518A,RFX4,ZNF730,HSF4,NHLH2,ZNF107,ZNF90,POU3F2,ASCL1,POU2F2,NR3C2,ZNF69,ZNF493,ZNF404,HIF3A,ZNF846,ZNF460,ZNF780B,ZNF138,PRDM5,SCX,REST,ZNF680,ZNF217,PLAGL1,ZNF273,SKIL,ZNF267

GO:MF	RNA polymerase II cis-regulatory region sequence-specific DNA binding	GO:0000978	2E-05	4.609802320305243	PTF1A,GSX1,PAX3,EN2,OLIG3,PAX7,HESX1,SP9,FOXB1,ZIC4,SOX13,IRX5,TFAP2B,THRB,ZIC5,ZIC3,ZNF83,ZIC1,MUC1,EBF1,ZNF117,FOXP2,MEIS1,EBF2,PROX1,MAF,NR2F2,HES5,IRX2,ZNF334,RORA,PLAG1,RFX3,IRX1,ZIC2,YY2,NR2F1,ZNF483,RFX4,ZNF730,HSF4,NHLH2,ZNF107,ZNF90,POU3F2,ASCL1,POU2F2,NR3C2,ZNF493,ZNF624,HIF3A,ZNF460,ZNF121,ZNF780B,ZNF138,PRDM5,SCX,REST,ZNF680,ZNF217,CHD7,ZNF273,SKIL,ZNF805,ZNF267
GO:MF	RNA polymerase II transcription regulatory region sequence-specific DNA binding	GO:0000977	3E-05	4.563869382911939	PTF1A,GSX1,PAX3,EN2,OLIG3,PAX7,HESX1,SP9,LHX5,FOXB1,ZIC4,SOX13,IRX5,DMBX1,TFAP2B,THRB,ZIC5,ZIC3,ZNF83,GBX2,ZIC1,MUC1,EBF1,ZNF117,FOXP2,MEIS1,EBF2,PROX1,MAF,NR2F2,HES5,IRX2,ZNF334,RORA,PLAG1,RFX3,IRX1,ZIC2,YY2,NR2F1,ZNF483,ZNF518A,RFX4,ZNF730,HSF4,NHLH2,ZNF107,ZNF90,POU3F2,ASCL1,POU2F2,NR3C2,ZNF493,ZNF624,HIF3A,ZNF460,ZNF121,ZNF780B,ZNF138,PRDM5,SCX,REST,ZNF680,ZNF217,PLAGL1,CHD7,ZNF273,SKIL,ZNF805,ZNF267
GO:MF	cis-regulatory region sequence-specific DNA binding	GO:0000987	5E-05	4.320964161162461	PTF1A,GSX1,PAX3,EN2,OLIG3,PAX7,HESX1,SP9,FOXB1,ZIC4,SOX13,IRX5,TFAP2B,THRB,ZIC5,ZIC3,ZNF83,ZIC1,MUC1,EBF1,ZNF117,FOXP2,MEIS1,EBF2,PROX1,MAF,NR2F2,HES5,IRX2,ZNF334,RORA,PLAG1,RFX3,IRX1,ZIC2,YY2,NR2F1,ZNF483,RFX4,ZNF730,HSF4,NHLH2,ZNF107,ZNF90,POU3F2,ASCL1,POU2F2,NR3C2,ZNF493,ZNF624,HIF3A,ZNF460,ZNF121,ZNF780B,ZNF138,PRDM5,SCX,REST,ZNF680,ZNF217,CHD7,ZNF273,SKIL,ZNF805,ZNF267
GO:MF	transcription cis-regulatory region binding	GO:0000976	1E-04	3.998158116013218	ERBB4,PTF1A,GSX1,PAX3,EN2,OLIG3,PAX7,HESX1,SP9,LHX5,FOXB1,ZIC4,SOX13,IRX5,DMBX1,TFAP2B,THRB,ZIC5,ZIC3,ZNF83,GBX2,ZIC1,MUC1,EBF1,ZNF117,FOXP2,MEIS1,EBF2,PROX1,MAF,NR2F2,HES5,IRX2,ZNF334,RORA,PLAG1,RFX3,IRX1,ZIC2,YY2,NR2F1,ZNF483,ZNF518A,RFX4,ZNF730,HSF4,NHLH2,ZNF107,ZNF90,SMAD6,POU3F2,ASCL1,POU2F2,NR3C2,ZNF493,ZNF624,HIF3A,ZNF460,ZNF121,ZNF780B,ZNF138,PRDM5,SCX,REST,ZNF680,ZNF217,PLAGL1,CHD7,ZNF273,SKIL,ZNF805,ZNF267
GO:MF	transcription regulatory region nucleic acid binding	GO:0001067	1E-04	3.974101336264201	ERBB4,PTF1A,GSX1,PAX3,EN2,OLIG3,PAX7,HESX1,SP9,LHX5,FOXB1,ZIC4,SOX13,IRX5,DMBX1,TFAP2B,THRB,ZIC5,ZIC3,ZNF83,GBX2,ZIC1,MUC1,EBF1,ZNF117,FOXP2,MEIS1,EBF2,PROX1,MAF,NR2F2,HES5,IRX2,ZNF334,RORA,PLAG1,RFX3,IRX1,ZIC2,YY2,NR2F1,ZNF483,ZNF518A,RFX4,ZNF730,HSF4,NHLH2,ZNF107,ZNF90,SMAD6,POU3F2,ASCL1,POU2F2,NR3C2,ZNF493,ZNF624,HIF3A,ZNF460,ZNF121,ZNF780B,ZNF138,PRDM5,SCX,REST,ZNF680,ZNF217,PLAGL1,CHD7,ZNF273,SKIL,ZNF805,ZNF267
GO:MF	DNA-binding transcription factor activity	GO:0003700	1E-04	3.852642295562024	PTF1A,GSX1,PAX3,EN2,OLIG3,PAX7,HESX1,SP9,LHX5,FOXB1,ZIC4,SOX13,IRX5,DMBX1,TFAP2B,THRB,ZIC5,ZIC3,ZNF83,GBX2,ZIC1,EBF1,ZNF117,FOXP2,MEIS1,EBF2,PROX1,MAF,NR2F2,HES5,IRX2,RORA,PLAG1,RFX3,IRX1,ZIC2,YY2,NR2F1,ZNF518A,RFX4,ZNF730,HSF4,NHLH2,ZNF107,ZNF90,POU3F2,ASCL1,POU2F2,NR3C2,ZNF69,ZNF493,ZNF404,HIF3A,ZNF846,ZNF460,ZNF780B,ZNF138,PRDM5,SCX,REST,ZNF680,ZNF217,PLAGL1,ZNF273,SKIL,ZNF267
GO:MF	transcription regulator activity	GO:0140110	3E-04	3.464420433863733	PTF1A,GSX1,PAX3,EN2,OLIG3,PAX7,HESX1,SP9,LHX5,FOXB1,ZIC4,SOX13,IRX5,DMBX1,TFAP2B,THRB,ZIC5,ZIC3,ZNF83,GBX2,ZIC1,MUC1,EBF1,ZNF117,FOXP2,MEIS1,EBF2,PROX1,MAF,NR2F2,HES5,IRX2,RORA,PLAG1,RFX3,IRX1,AASS,ZIC2,YY2,CCND1,NR2F1,ZNF518A,RFX4,ZNF730,HSF4,NHLH2,ZNF107,ZNF90,POU3F2,ASCL1,POU2F2,NR3C2,ZNF69,DNMT3B,ZNF493,TOX3,ZNF404,BTAF1,TOB1,HIF3A,EAF2,TRIM22,ZNF846,MAK,ZNF460,TRIM5,ZNF780B,ZNF138,PRDM5,SCX,TMF1,REST,ZNF680,BTG2,RAP2C,ZNF217,FGF2,PLAGL1,ZNF273,SKIL,ZNF267,JMJD1C,TRIM52
GO:MF	sequence-specific double-stranded DNA binding	GO:1990837	4E-04	3.4437054121478043	ERBB4,PTF1A,GSX1,PAX3,EN2,OLIG3,PAX7,HESX1,SP9,LHX5,FOXB1,ZIC4,SOX13,IRX5,DMBX1,TFAP2B,THRB,ZIC5,ZIC3,ZNF83,GBX2,ZIC1,MUC1,EBF1,ZNF117,FOXP2,MEIS1,EBF2,PROX1,MAF,NR2F2,HES5,IRX2,ZNF334,RORA,PLAG1,RFX3,IRX1,ZIC2,YY2,NR2F1,ZNF483,ZNF518A,RFX4,ZNF730,HSF4,NHLH2,ZNF107,ZNF90,SMAD6,POU3F2,ASCL1,POU2F2,NR3C2,ZNF493,ZNF624,HIF3A,ZNF460,CENPC,ZNF121,ZNF780B,ZNF138,PRDM5,SCX,REST,ZNF680,ZNF217,PLAGL1,CHD7,ZNF273,SKIL,ZNF805,ZNF267

GO:MF	sequence-specific DNA binding	GO:0043565	8E-04	3.100827060525939	ERBB4,PTF1A,GSX1,PAX3,EN2,OLIG3,PAX7,HESX1,SP9,LHX5,FOXB1,ZIC4,SOX13,IRX5,DMBX1,TFAP2B,THRB,ZIC5,ZIC3,ZNF83,GBX2,ZIC1,MUC1,EBF1,ZNF117,FOXP2,MEIS1,MAEL,EBF2,PROX1,MAF,NR2F2,HES5,IRX2,ZNF334,RORA,PLAG1,RFX3,IRX1,GOLGB1,ZIC2,YY2,NR2F1,ZNF483,ZNF518A,RFX4,ZNF730,HSF4,NHLH2,ZNF107,ZNF90,SMAD6,POU3F2,ASCL1,POU2F2,NR3C2,ZNF493,ZNF624,HIF3A,ZNF460,CENPC,ZNF121,ZNF780B,ZNF138,PRDM5,SCX,REST,ZNF680,TET1,ZNF217,PLAGL1,CHD7,ZNF273,SKIL,ZNF805,ZNF267
GO:MF	double-stranded DNA binding	GO:0003690	0.002	2.723289844177103	ERBB4,PTF1A,GSX1,PAX3,EN2,OLIG3,PAX7,HESX1,SP9,LHX5,FOXB1,MSH5-SAPCD1,ZIC4,SOX13,IRX5,DMBX1,TFAP2B,THRB,ZIC5,ZIC3,ZNF83,GBX2,ZIC1,MUC1,EBF1,ZNF117,FOXP2,MEIS1,EBF2,PROX1,MAF,NR2F2,HES5,IRX2,ZNF334,RORA,PLAG1,RFX3,IRX1,ZIC2,YY2,NR2F1,ZNF483,ZNF518A,RFX4,ZNF730,HSF4,NHLH2,ZNF107,ZNF90,SMAD6,POU3F2,ASCL1,POU2F2,NR3C2,ZNF493,ZNF624,HIF3A,ZNF460,CENPC,ZNF121,ZNF780B,ZNF138,PRDM5,SCX,REST,ZNF680,ZNF217,PLAGL1,CHD7,ZNF273,SKIL,ZNF805,ZNF267
GO:MF	DNA binding	GO:0003677	0.002	2.6930833257901803	ERBB4,PTF1A,ZNF80,GSX1,PAX3,PRDM13,EN2,OLIG3,PAX7,HESX1,SP9,LHX5,FOXB1,MSH5-SAPCD1,ZIC4,SOX13,IRX5,DMBX1,TFAP2B,THRB,ZIC5,ZIC3,ZNF83,GBX2,ZIC1,MUC1,CXXC4,EBF1,ZNF117,FOXP2,MEIS1,MAEL,EBF2,PROX1,MAF,NR2F2,HES5,IRX2,ZNF334,RORA,PLAG1,RFX3,IRX1,GOLGB1,ZMAT1,ZIC2,YY2,HMGN5,NR2F1,ZNF483,ZNF518A,RFX4,ZNF730,HSF4,NHLH2,ZNF107,ZNF90,SMAD6,ATAD5,POU3F2,ANG,SMC1B,ASCL1,POLI,POU2F2,NR3C2,ZNF69,DNMT3B,ZNF493,TOX3,TIGD4,ZNF624,ZNF404,BTAF1,HIF3A,HEMK1,LUC7L3,ZNF846,PNN,ZNF460,CENPC,ZNF121,ZNF780B,LOC100133315,NKAP,HLTF,ZNF138,PRDM5,SCX,TMF1,REST,ZNF680,SMC5,TET1,BAZ2B,ZNF217,JRKL,PLAGL1,CHD7,ZNF273,CHD1,SKIL,PAXBP1,FANCM,ZNF805,ZNF267,JMJD1C
GO:MF	minus-end-directed microtubule motor activity	GO:0008569	0.002	2.6049066360132325	DNAH12,DNAH8,DNAH7,DNAH2,DNHD1,DNAH6
GO:MF	dynein light intermediate chain binding	GO:0051959	0.004	2.409345297656179	DNAH12,DNAH8,DNAH7,CCDC88B,DNAH2,DNHD1,DNAH6

GO:MF	protein binding	GO:0005515	0.014987243278322487	1.8242782430098805	TRIM49B,TRIM49,PRAME,MMRN1,LRRC74B,ROBO3,ERBB4,PTF1A,GOLGA6A,ZNF80,DNAH12,COL6A5,GSX1,PAX3,ATP1A4,WDR49,EDNRB,PRDM13,SERPINI2,THEMIS,ALOX15,ANKRD33,PRTG,OLIG3,GOLGA8B,GOLGA8A,TPBG,PAX7,CFAP70,CABP4,VGLL3,CFAP43,HESX1,PDK4,DNAH8,NRG4,C22ORF15,LHX5,FOXB1,MSH5-SAPCD1,PLEKHH2,SOX13,RFTN2,WSB1,SPARCL1,SPAG8,ANGPTL3,KIRREL2,COL7A1,INTU,NAALAD2,ABCA1,ANGPT1,HOGA1,ITGB8,GATM,ADGRV1,NPEPL1,NPHS1,GRM1,NEAT1,KEL,DMBX1,NOXA1,INPP5D,ACSS3,LRP2,CCDC102B,TFAP2B,PDE6C,ZSWIM2,THRB,CYP26B1,SEC31B,ZIC3,PHEX,MSTN,ZNF83,AQP6,UNC5CL,GBX2,PAQR6,CCNB3,NTRK2,INO80B,C11ORF65,MPL,PTN,MAP1LC3B2,PSCA,WNT5A,ZIC1,APCDD1,CFAP44,GAD2,MUC1,ANKRD30B,FPGT-TNNI3K,MAPK15,TTC14,EBF1,ADAM28,RGPD4,FOXP2,RPGR,MEIS1,NDNF,CEP290,PDPN,PLP1,NLRP14,EBF2,DNAH7,NTN5,MST1,NKG7,PROX1,MUC6,NPHP3-ACAD11,PRAM1,ARL4A,LRRC9,UACA,MAF,RSRP1,TRIM45,MAMDC4,TEX9,MORN5,BDH2,DNAI1,NPPC,AKNAD1,UNC13D,CENPE,NR2F2,HES5,LY6G6C,ACRBP,HEPACAM2,IRX2,AP1G2,ZNF334,WLS,LRP2BP,LAG3,TEX14,RORA,DCDC1,LGR4,FAM166A,RDH16,RFX3,CDK6,GNRH1,CALCRL,FBXO24,GOLGB1,CLDN10,AASS,MYO15B,TPR,HSD17B13,SMOC1,GOLGA4,DLL1,PTCH1,HHIP,CCND1,LCA5L,CD37,CRABP1,FANK1,PIFO,NR2F1,AMT,MYO5C,GLYCTK,NPPA,ARHGAP30,ANKDD1B,SPATA4,GOLGA8N,ZNF483,BMPR1B,MCC,GOLGA6L9,MYH15,AGER,RFX4,ETAA1,RGS5,COL4A3,KITLG,TTPA,HSF4,QKI,CD72,CEP162,MEGF10,HRSP12,SLITRK2,LRRIQ3,FMO4,GNB3,NHLH2,ZFC3H1,RIBC1,LRRC70,CDNF,INHBC,CCDC88B,TRIM7,OFD1,DUSP10,MNS1,TRPC4,CCDC14,ZNF107,FAM47E,YES1,ATG16L2,WNT2B,CCDC150,CRABP2,PTPN13,TBC1D32,CEP83,CXCR4,DNAH2,TNNI3,DCST1,SMAD6,AXIN2,BTBD19,ATAD5,DCDC2B,DYNLRB2,MOB1B,DKKL1,WFDC3,RASSF10,LENG8,PROCA1,POU3F2,ARR3,SCARF1,PLG,EEA1,DNHD1,ZMYND12,USP6,KLHDC9,KRT10,ANG,GPM6B,MASP1,SMC1B,TMEM106A,ASCL1,AFF1,CTSV,COL4A5,SPATA6L,USH2A,POLI,POU2F2,JAG1,FSTL5,ACVR2B,LRRIQ1,TTC32,UVSSA,MTM1,NXT2,IQUB,DRC7,CNTRL,MTTP,RFESD,STXBP3,SAR1B,DNAH6,NR3C2,ZNF69,DNMT3B,ZNF493,MAP3K8,TOX3,TIGD4,ANP32E,ZNF624,RAD9B,MEGF6,PRICKLE4,LCA5,ZNF404,WDR93,C6ORF25,PARD6B,BTAF1,NPHP3,CBR4,PCDHA7,EPHA3,TOB1,CEP44,PARD3B,EOGT,L3MBTL1,TMEM123,UFL1,FRA10AC1,KRTCAP3,CARD14,RPGRIP1,LAT,ABCG1,HIF3A,HEMK1,UGGT2,RBBP6,SPATA6,LUC7L3,C17ORF75,ITGB7,SNX20,EAF2,WNT3,PON2,TRIM22,ZNF846,SUCLG2,ZFYVE16,MAK,IFT81,MYH7B,SLC35A3,PUS10,PILRA,RND3,ACSL6,RHD,CRYBG3,PNN,NUP62CL,B3GNT5,ZNF460,CORO6,TRIM5,CDK5RAP3,XPNPEP3,TAS2R19,CENPC,ZNF121,SCAF11,OCLN,NMU,PDCD4,SPICE1,PDLIM5,DSG2,USP45,TNFRSF25,NKAP,NRXN3,HLTF,TSPAN12,ANKRD31,LRRCC1,SLC24A1,MPHOSPH10,EDA2R,TTC26,RMDN2,C7ORF31,OPN1SW,ZNF138,ZC3H12C,PRDM5,SASS6,SCX,GLIPR1L2,MTR,INSIG2,COL11A2,ADAM9,TMF1,SPEF2,TMEM47,REST,SENP7,TYW5,EVI5,GLIPR1,SMC5,T
GO:MF	cytoskeletal motor activity	GO:0003774	0.016621914190929933	1.77931896	DNAH12,DNAH8,DNAH7,DNAI1,CENPE,MYO15B,MYO5C,MYH15,DNAH2,DYNLRB2,DNHD1,DNAH6,MYH7B

GO:MF	ion binding	GO:0043167	0.035	1.457695613823444	KDM4E,TRIM49B,TRIM49,MMRN1,ETNPPL,ERBB4,ZNF80,DNAH12,ATP1A4,WDR49,PRDM13,TKTL1,ALOX15,CABP4,AMY2B,GDPD2,PDK4,CSAD,DNAH8,SP9,ZBBX,LHX5,MSH5-SAPCD1,ZIC4,SPARCL1,PKD1L2,NAALAD2,ABCA1,RPE65,HFM1,ADGRV1,ADAMTS6,NPEPL1,KEL,ACSS3,LRP2,CAPS2,PDE6C,ZSWIM2,THRB,LCN12,ZIC5,CYP26B1,ZIC3,ENPP2,PHEX,ZNF83,NTRK2,INO80B,NEK5,CYP39A1,ZIC1,ABCA8,GAD2,CXXC4,FPGT-TNNI3K,MAPK15,EBF1,ADAM28,ZNF117,NRBP2,FOXP2,NLRP14,EBF2,DNAH7,STK33,IFI44L,ARL4A,MAN2C1,TRIM45,CNGA4,UNC13D,CENPE,NR2F2,ZNF334,RGN,TEX14,RORA,PLAG1,CDK6,ZMAT1,MYO15B,ZIC2,SMOC1,DLL1,YY2,HHIP,CRABP1,NR2F1,MYO5C,GLYCTK,ZNF483,BMPR1B,GK5,MCC,FIGNL2,ZNF518A,MYH15,ZNF730,TTPA,AK7,FMO4,ZFC3H1,TRIM7,PLCH1,TRPC4,ZNF107,YES1,CRABP2,ZNF90,DNAH2,DCST1,SMAD6,ATAD5,MOB1B,EEA1,DNHD1,ZMYND12,LFNG,ANG,MASP1,SMC1B,POLI,JAG1,FSTL5,ACVR2B,RFESD,SAR1B,DNAH6,NR3C2,ZNF69,DNMT3B,ZNF493,MAP3K8,TRHDE,ZNF624,MEGF6,PRICKLE4,ZNF404,BTAF1,CBR4,PCDHA7,EPHA3,L3MBTL1,CDO1,WEE2,ABCG1,RBBP6,STK31,ITGB7,YPEL4,SNX20,PON2,TRIM22,ZNF846,SUCLG2,ST8SIA4,ZFYVE16,MAK,MYH7B,PUS10,RND3,ACSL6,ZNF460,TRIM5,XPNPEP3,ZNF121,SCAF11,ZNF780B,PDLIM5,DSG2,CA14,USP45,NRXN3,HLTF,ZNF138,ZC3H12C,PRDM5,MTR,COL11A2,ADAM9,SLFN5,REST,TYW5,ZNF680,SMC5,TET1,EFHC2,RAP2C,NADK2,BAZ2B,ABCC2,P4HA1,ZNF217,PLAGL1,ENOSF1,RRM2B,EPX,CHD7,ZNF273,CHD1,ESCO1,ACADVL,FANCM,TGM1,CAPRIN2,ZNF805,ZNF267,JMJD1C,LMLN,TRIM52
GO:MF	dynein intermediate chain binding	GO:0045505	0.03557360043235 3115	1.4488721773002549	DNAH12,DNAH8,DNAH7,DNAH2,DYNLRB2,DNHD1,DNAH6
GO:BP	animal organ development	GO:0048513	1.0227806540650 43e-9	8.9902175	ERBB4,PTF1A,GSX1,PAX3,EDNRB,PRDM13,EN2,ALOX15,ANKRD33,OLIG3,PAX7,CABP4,CFAP43,HESX1,LHX5,FOXB1,SOX13,INTU,ANGPT1,ITGB8,RPE65,IRX5,ADGRV1,ADAMTS6,NPHS1,KEL,DMBX1,LRP2,STRC,TFAP2B,PDE6C,ILDR2,THRB,CYP26B1,ZIC3,PHEX,MSTN,AQP6,CCDC39,GBX2,NTRK2,PTN,WNT5A,ZIC1,APCDD1,FOXP2,MEIS1,NDNF,CEP290,PDPN,PLP1,EBF2,MST1,PROX1,MAF,TRIM45,DNAI1,NPPC,NR2F2,HES5,IRX2,RGN,WLS,RORA,PLAG1,LGR4,RFX3,CDK6,IRX1,GNRH1,CALCRL,ZIC2,SMOC1,DLL1,PTCH1,HHIP,CCND1,CCDC154,PIFO,NPPA,BMPR1B,MYH15,RFX4,COL4A3,KITLG,TTPA,HSF4,MEGF10,NHLH2,DHRS3,CCDC14,WNT2B,TBC1D32,CXCR4,TNNI3,SMAD6,AXIN2,POU3F2,KRT10,LFNG,ANG,GPM6B,ASCL1,USH2A,JAG1,ACVR2B,MTM1,CNTRL,STXBP3,PRICKLE4,NPHP3,EPHA3,CDO1,RPGRIP1,RBBP6,EAF2,WNT3,CRYBG3,B3GNT5,CDK5RAP3,PDCD4,PDLIM5,DSG2,USP45,TSPAN12,SCX,INSIG2,COL11A2,ADAM9,TMF1,SPEF2,REST,BTG2,EXPH5,TTC8,FGF2,RRM2B,AMH,NPHP1,CHD7,SKIL,PAXBP1,TGM1,IFT80

GO:BP	developmental process	GO:0032502	1.008046189440 7839e-8	7.996519567732091	OR51E2,PRAME,PRAMEF12,ROBO3,ERBB4,PTF1A,GSX1,PAX3,ATP1A4,EDNRB,PRDM13,THEMIS,EN2,ALOX15,ANKRD33,PRTG,OLIG3,TPBG,PAX7,CABP4,CFAP43,HESX1,GDPD2,NRG4,SP9,LHX5,FOXB1,ZIC4,CFAP54,SOX13,SPARCL1,SPAG8,ANGPTL3,COL7A1,INTU,ABCA1,ANGPT1,ITGB8,RPE65,IRX5,ADGRV1,ADAMTS6,NPHS1,KEL,DMBX1,INPP5D,LRP2,STRC,TFAP2B,PDE6C,ILDR2,THRB,ZIC5,CYP26B1,ZIC3,ENPP2,PHEX,MSTN,AQP6,CCDC39,GBX2,NTRK2,INO80B,C11ORF65,MPL,PTN,MIAT,WNT5A,ZIC1,APCDD1,CFAP44,ADAM28,NRBP2,FOXP2,MEIS1,NDNF,CEP290,PDPN,PLP1,NLRP14,MAEL,EBF2,NTN5,MST1,PROX1,ARL4A,MAF,TRIM45,SPATA9,BDH2,DNAI1,NPPC,UNC13D,NR2F2,HES5,ACRBP,IRX2,CCDC78,RGN,WLS,LAG3,RORA,STRIP2,CFAP69,PLAG1,LGR4,RFX3,CDK6,IRX1,GNRH1,CALCRL,ZIC2,SMOC1,GOLGA4,DLL1,PTCH1,CRISPLD1,HHIP,CCND1,CCDC154,PIFO,NR2F1,NPPA,CATSPERG,BMPR1B,MYH15,AGER,RFX4,COL4A3,KITLG,TTPA,HSF4,QKI,MEGF10,SLITRK2,GNB3,NHLH2,DHRS3,CDNF,TDRP,OFD1,DUSP10,MNS1,TRPC4,CCDC14,YES1,WNT2B,CRABP2,PTPN13,TBC1D32,CXCR4,TNNI3,SMAD6,AXIN2,ATAD5,C5AR1,DKKL1,RASSF10,POU3F2,SCARF1,DNHD1,KRT10,LFNG,ANG,GPM6B,ASCL1,SPATA6L,USH2A,POU2F2,JAG1,FSTL5,ACVR2B,MTM1,DRC7,CNTRL,STXBP3,PRICKLE4,C6ORF25,PARD6B,NPHP3,PCDHA7,EPHA3,TOB1,L3MBTL1,CDO1,WEE2,UFL1,RPGRIP1,ABCG1,HIF3A,RBBP6,ARHGAP15,SPATA6,EAF2,WNT3,ST8SIA4,MAK,IFT81,ACSL6,CRYBG3,B3GNT5,CDK5RAP3,PDCD4,PDLIM5,DSG2,USP45,NKAP,NRXN3,HLTF,TSPAN12,EDA2R,TTC26,PABPC1L,SCX,MTR,INSIG2,COL11A2,ADAM9,TMF1,SLFN5,SPEF2,REST,SMC5,TET1,EFHC2,BTG2,RAP2C,EXPH5,TTC8,RIN2,JRKL,FGF2,RRM2B,AMH,ACKR3,NPHP1,CHD7,ACADVL,SKIL,PAXBP1,TGM1,IFT80,CAPRIN2
GO:BP	cell differentiation	GO:0030154	2.873963526719 6034e-8	7.541518747769269	OR51E2,PRAME,PRAMEF12,ROBO3,ERBB4,PTF1A,GSX1,EDNRB,PRDM13,THEMIS,EN2,ANKRD33,PRTG,OLIG3,TPBG,CABP4,CFAP43,HESX1,GDPD2,LHX5,FOXB1,CFAP54,SOX13,SPAG8,COL7A1,INTU,ABCA1,ANGPT1,ITGB8,IRX5,ADGRV1,NPHS1,KEL,INPP5D,LRP2,STRC,TFAP2B,PDE6C,ILDR2,THRB,ZIC5,CYP26B1,ZIC3,MSTN,GBX2,NTRK2,MPL,PTN,MIAT,WNT5A,ZIC1,APCDD1,CFAP44,NRBP2,MEIS1,NDNF,CEP290,PDPN,PLP1,NLRP14,MAEL,EBF2,NTN5,PROX1,ARL4A,MAF,SPATA9,BDH2,NPPC,NR2F2,HES5,ACRBP,IRX2,CCDC78,LAG3,RORA,CFAP69,PLAG1,LGR4,RFX3,CDK6,IRX1,GNRH1,ZIC2,SMOC1,GOLGA4,DLL1,PTCH1,HHIP,CCND1,NR2F1,CATSPERG,BMPR1B,AGER,KITLG,TTPA,HSF4,QKI,MEGF10,SLITRK2,GNB3,NHLH2,CDNF,DUSP10,MNS1,TRPC4,YES1,WNT2B,CRABP2,CXCR4,TNNI3,SMAD6,AXIN2,C5AR1,DKKL1,RASSF10,POU3F2,SCARF1,DNHD1,KRT10,LFNG,ANG,GPM6B,ASCL1,USH2A,POU2F2,JAG1,FSTL5,ACVR2B,DRC7,C6ORF25,PARD6B,NPHP3,EPHA3,TOB1,L3MBTL1,WEE2,UFL1,RPGRIP1,ABCG1,SPATA6,WNT3,MAK,IFT81,ACSL6,CDK5RAP3,PDCD4,PDLIM5,NKAP,NRXN3,HLTF,EDA2R,TTC26,PABPC1L,SCX,MTR,COL11A2,ADAM9,TMF1,SLFN5,SPEF2,REST,TET1,EFHC2,BTG2,RAP2C,EXPH5,TTC8,RIN2,FGF2,AMH,ACKR3,NPHP1,CHD7,ACADVL,SKIL,TGM1,IFT80,CAPRIN2
GO:BP	cellular developmental process	GO:0048869	2.933136164330606e-8	7.532667775394907	OR51E2,PRAME,PRAMEF12,ROBO3,ERBB4,PTF1A,GSX1,EDNRB,PRDM13,THEMIS,EN2,ANKRD33,PRTG,OLIG3,TPBG,CABP4,CFAP43,HESX1,GDPD2,LHX5,FOXB1,CFAP54,SOX13,SPAG8,COL7A1,INTU,ABCA1,ANGPT1,ITGB8,IRX5,ADGRV1,NPHS1,KEL,INPP5D,LRP2,STRC,TFAP2B,PDE6C,ILDR2,THRB,ZIC5,CYP26B1,ZIC3,MSTN,GBX2,NTRK2,MPL,PTN,MIAT,WNT5A,ZIC1,APCDD1,CFAP44,NRBP2,MEIS1,NDNF,CEP290,PDPN,PLP1,NLRP14,MAEL,EBF2,NTN5,PROX1,ARL4A,MAF,SPATA9,BDH2,NPPC,NR2F2,HES5,ACRBP,IRX2,CCDC78,LAG3,RORA,CFAP69,PLAG1,LGR4,RFX3,CDK6,IRX1,GNRH1,ZIC2,SMOC1,GOLGA4,DLL1,PTCH1,HHIP,CCND1,NR2F1,CATSPERG,BMPR1B,AGER,KITLG,TTPA,HSF4,QKI,MEGF10,SLITRK2,GNB3,NHLH2,CDNF,DUSP10,MNS1,TRPC4,YES1,WNT2B,CRABP2,CXCR4,TNNI3,SMAD6,AXIN2,C5AR1,DKKL1,RASSF10,POU3F2,SCARF1,DNHD1,KRT10,LFNG,ANG,GPM6B,ASCL1,USH2A,POU2F2,JAG1,FSTL5,ACVR2B,DRC7,C6ORF25,PARD6B,NPHP3,EPHA3,TOB1,L3MBTL1,WEE2,UFL1,RPGRIP1,ABCG1,SPATA6,WNT3,MAK,IFT81,ACSL6,CDK5RAP3,PDCD4,PDLIM5,NKAP,NRXN3,HLTF,EDA2R,TTC26,PABPC1L,SCX,MTR,COL11A2,ADAM9,TMF1,SLFN5,SPEF2,REST,TET1,EFHC2,BTG2,RAP2C,EXPH5,TTC8,RIN2,FGF2,AMH,ACKR3,NPHP1,CHD7,ACADVL,SKIL,TGM1,IFT80,CAPRIN2
GO:BP	cilium organization	GO:0044782	1.166827931776 0472e-7	6.932993183191877	CFAP70,CFAP43,DNAH8,CFAP54,INTU,CCDC39,CFAP44,MAPK15,RPGR,CEP290,DNAH7,DNAI1,CFAP69,RFX3,LCA5L,PIFO,RFX4,CEP162,OFD1,MNS1,TBC1D32,CEP83,DNAH2,DNHD1,IQUB,DRC7,LCA5,NPHP3,RPGRIP1,SPATA6,MAK,IFT81,TTC26,SPEF2,WDR90,TTC8,IFT80

GO:BP	system development	GO:0048731	3.631460519286932 6e-7	6.439918673063658	ROBO3,ERBB4,PTF1A,GSX1,PAX3,EDNRB,PRDM13,EN2,PRTG,OLIG3,TPBG,CABP4,CFAP43,HESX1,NRG4,LHX5,FOXB1,ZIC4,SOX13,ANGPTL3,INTU,ANGPT1,ITGB8,RPE65,IRX5,ADGRV1,ADAMTS6,NPHS1,KEL,DMBX1,LRP2,STRC,TFAP2B,PDE6C,THRB,ZIC5,CYP26B1,ZIC3,PHEX,CCDC39,GBX2,NTRK2,PTN,WNT5A,ZIC1,APCDD1,NRBP2,FOXP2,MEIS1,NDNF,CEP290,PDPN,PLP1,NTN5,PROX1,MAF,TRIM45,DNAI1,NPPC,NR2F2,HES5,IRX2,RGN,WLS,RORA,PLAG1,LGR4,RFX3,CDK6,IRX1,GNRH1,CALCRL,ZIC2,SMOC1,GOLGA4,DLL1,PTCH1,HHIP,CCND1,CCDC154,PIFO,NR2F1,NPPA,BMPR1B,MYH15,AGER,RFX4,COL4A3,KITLG,TTPA,HSF4,QKI,SLITRK2,NHLH2,DHRS3,CDNF,DUSP10,TRPC4,CCDC14,WNT2B,CRABP2,PTPN13,TBC1D32,CXCR4,TNNI3,SMAD6,AXIN2,ATAD5,C5AR1,RASSF10,POU3F2,SCARF1,LFNG,ANG,GPM6B,ASCL1,USH2A,JAG1,ACVR2B,CNTRL,STXBP3,PRICKLE4,PARD6B,NPHP3,PCDHA7,EPHA3,UFL1,RPGRIP1,HIF3A,EAF2,WNT3,ST8SIA4,ACSL6,CRYBG3,B3GNT5,CDK5RAP3,PDCD4,PDLIM5,DSG2,USP45,NRXN3,HLTF,TSPAN12,SCX,MTR,INSIG2,COL11A2,TMF1,SPEF2,REST,EFHC2,BTG2,TTC8,RIN2,JRKL,FGF2,RRM2B,AMH,ACKR3,NPHP1,CHD7,SKIL,IFT80,CAPRIN2
GO:BP	multicellular organismal process	GO:0032501	8.387385369351313e-7	6.0763734022981675	OR51E2,MMRN1,ROBO3,ERBB4,PTF1A,PKHD1L1,GSX1,PAX3,ATP1A4,EDNRB,PRDM13,THEMIS,EN2,ALOX15,PRTG,OLIG3,TPBG,SLC26A7,CABP4,CFAP43,HESX1,GDPD2,PDK4,NRG4,SP9,LHX5,FOXB1,ZIC4,GLIPR1L1,CFAP54,SOX13,SPAG8,ANGPTL3,COL7A1,INTU,ABCA1,ANGPT1,ITGB8,RPE65,GATM,IRX5,ADGRV1,ADAMTS6,NPHS1,GRM1,KEL,DMBX1,INPP5D,LRP2,STRC,TFAP2B,PDE6C,ILDR2,THRB,ZIC5,CYP26B1,ZIC3,ENPP2,PHEX,MSTN,AQP6,CCDC39,GBX2,NTRK2,INO80B,MPL,PTN,WNT5A,CYP39A1,ZIC1,APCDD1,CFAP44,ADAM28,NRBP2,FOXP2,RPGR,MEIS1,NDNF,CEP290,PDPN,PLP1,NLRP14,MAEL,EBF2,NTN5,MST1,NKG7,PROX1,MUC6,PRAM1,MAF,TRIM45,CNGA4,SPATA9,DNAI1,NPPC,UNC13D,NR2F2,HES5,ACRBP,IRX2,CCDC78,RGN,WLS,LAG3,TEX14,RORA,CFAP69,PLAG1,LGR4,SSPN,RFX3,CDK6,IRX1,GNRH1,CALCRL,ZIC2,SMOC1,GOLGA4,DLL1,PTCH1,CRISPLD1,HHIP,CCND1,CCDC154,PIFO,NR2F1,NPPA,CATSPERG,BMPR1B,MCC,MYH15,AGER,RFX4,COL4A3,KITLG,DCST2,TTPA,HSF4,QKI,MEGF10,SLITRK2,FMO4,GNB3,NHLH2,DHRS3,CDNF,CCDC88B,TDRP,OFD1,DUSP10,MNS1,TRPC4,CCDC14,YES1,WNT2B,ASIC3,CRABP2,PTPN13,TBC1D32,CXCR4,TNNI3,DCST1,SMAD6,AXIN2,ATAD5,C5AR1,DKKL1,RASSF10,POU3F2,ARR3,SCARF1,PLG,EEA1,DNHD1,LFNG,ANG,GPM6B,TMEM106A,ASCL1,SPATA6L,USH2A,POU2F2,JAG1,ACVR2B,DRC7,CNTRL,MTTP,STXBP3,MAP3K8,PRICKLE4,LCA5,C6ORF25,PARD6B,VN1R1,NPHP3,PCDHA7,EPHA3,TOB1,L3MBTL1,WEE2,UFL1,RPGRIP1,LAT,ABCG1,HIF3A,RBBP6,SPATA6,EAF2,WNT3,ST8SIA4,MAK,IFT81,MYH7B,ACSL6,CRYBG3,B3GNT5,CDK5RAP3,XPNPEP3,TAS2R19,OCLN,NMU,PDCD4,PDLIM5,DSG2,USP45,NKAP,NRXN3,HLTF,TSPAN12,SLC24A1,TTC26,OPN1SW,TAS2R31,PABPC1L,SCX,MTR,INSIG2,COL11A2,ADAM9,TMF1,SPEF2,REST,SMC5,TET1,TAS2R5,EFHC2,BTG2,ABCC2,C5,LOC100507547,TTC8,RIN2,JRKL,FGF2,PDIA2,TAS2R20,RRM2B,AMH,PBLD,ACKR3,NPHP1,EPX,CHD7,ACADVL,SKIL,TGM1,IFT80,CAPRIN2,JMJD1C
GO:BP	cilium assembly	GO:0060271	9.619455640264954e-7	6.016849503754099	CFAP70,CFAP43,DNAH8,CFAP54,INTU,CCDC39,CFAP44,MAPK15,RPGR,CEP290,DNAH7,DNAI1,CFAP69,RFX3,RFX4,CEP162,OFD1,MNS1,TBC1D32,CEP83,DNAH2,DNHD1,IQUB,DRC7,NPHP3,RPGRIP1,SPATA6,MAK,IFT81,TTC26,SPEF2,WDR90,TTC8,IFT80
GO:BP	cell population proliferation	GO:0008283	1E-06	5.90748689	OR51E2,PRAME,PRAMEF12,ROBO3,ERBB4,EDNRB,PRTG,OLIG3,TPBG,CFAP70,CFAP43,DNAH8,LHX5,FOXB1,CFAP54,INTU,ANGPT1,ADGRV1,NEAT1,KEL,INPP5D,LRP2,STRC,TFAP2B,ENPP2,MSTN,CCDC39,GBX2,NTRK2,MPL,PTN,WNT5A,CFAP44,MAPK15,RPGR,MEIS1,NDNF,CEP290,PDPN,PLP1,DNAH7,MST1,PROX1,DNAI1,NPPC,NR2F2,HES5,CCDC78,RGN,RORA,CFAP69,PLAG1,LGR4,RFX3,CDK6,CALCRL,GOLGB1,GOLGA4,DLL1,PTCH1,HMGN5,HHIP,CCND1,LCA5L,PIFO,NR2F1,BMPR1B,MCC,AGER,RFX4,COL4A3,KITLG,CEP162,MEGF10,AK7,SLITRK2,CDNF,CCDC88B,OFD1,DUSP10,MNS1,WNT2B,CRABP2,TBC1D32,CEP83,CXCR4,DNAH2,SMAD6,AXIN2,ATAD5,C5AR1,RASSF10,MTBP,POU3F2,SCARF1,PLG,DNHD1,ANG,GPM6B,ASCL1,JAG1,IQUB,DRC7,LCA5,PARD6B,NPHP3,EPHA3,TOB1,UFL1,RPGRIP1,SPATA6,EAF2,WNT3,MAK,IFT81,ACSL6,CDK5RAP3,OCLN,PDCD4,PDLIM5,NKAP,NRXN3,TTC26,SCX,MTR,SPEF2,REST,EFHC2,BTG2,WDR90,TTC8,FGF2,ACKR3,NPHP1,SKIL,PAXBP1,TGM1,IFT80,CAPRIN2

GO:BP	anatomical structure development	GO:0048856	1E-06	5.891928967513984	ROBO3,ERBB4,PTF1A,GSX1,PAX3,EDNRB,PRDM13,THEMIS,EN2,ALOX15,ANKRD33,PRTG,OLIG3,TPBG,PAX7,CABP4,CFAP43,HESX1,NRG4,SP9,LHX5,FOXB1,ZIC4,CFAP54,SOX13,SPARCL1,ANGPTL3,COL7A1,INTU,ANGPT1,ITGB8,RPE65,IRX5,ADGRV1,ADAMTS6,NPHS1,KEL,DMBX1,INPP5D,LRP2,STRC,TFAP2B,PDE6C,ILDR2,THRB,ZIC5,CYP26B1,ZIC3,ENPP2,PHEX,MSTN,AQP6,CCDC39,GBX2,NTRK2,INO80B,C11ORF65,MPL,PTN,WNT5A,ZIC1,APCDD1,CFAP44,NRBP2,FOXP2,MEIS1,NDNF,CEP290,PDPN,PLP1,MAEL,EBF2,NTN5,MST1,PROX1,MAF,TRIM45,BDH2,DNAI1,NPPC,UNC13D,NR2F2,HES5,ACRBP,IRX2,CCDC78,RGN,WLS,LAG3,RORA,STRIP2,CFAP69,PLAG1,LGR4,RFX3,CDK6,IRX1,GNRH1,CALCRL,ZIC2,SMOC1,GOLGA4,DLL1,PTCH1,CRISPLD1,HHIP,CCND1,CCDC154,PIFO,NR2F1,NPPA,BMPR1B,MYH15,AGER,RFX4,COL4A3,KITLG,TTPA,HSF4,QKI,MEGF10,SLITRK2,NHLH2,DHRS3,CDNF,OFD1,DUSP10,MNS1,TRPC4,CCDC14,WNT2B,CRABP2,PTPN13,TBC1D32,CXCR4,TNNI3,SMAD6,AXIN2,ATAD5,C5AR1,DKKL1,RASSF10,POU3F2,SCARF1,DNHD1,KRT10,LFNG,ANG,GPM6B,ASCL1,USH2A,POU2F2,JAG1,ACVR2B,MTM1,DRC7,CNTRL,STXBP3,PRICKLE4,C6ORF25,PARD6B,NPHP3,PCDHA7,EPHA3,L3MBTL1,CDO1,WEE2,UFL1,RPGRIP1,HIF3A,RBBP6,ARHGAP15,EAF2,WNT3,ST8SIA4,IFT81,ACSL6,CRYBG3,B3GNT5,CDK5RAP3,PDCD4,PDLIM5,DSG2,USP45,NKAP,NRXN3,HLTF,TSPAN12,EDA2R,TTC26,PABPC1L,SCX,MTR,INSIG2,COL11A2,ADAM9,TMF1,SPEF2,REST,TET1,EFHC2,BTG2,RAP2C,EXPH5,TTC8,RIN2,JRKL,FGF2,RRM2B,AMH,ACKR3,NPHP1,CHD7,ACADVL,SKIL,PAXBP1,TGM1,IFT80,CAPRIN2
GO:BP	pattern specification process	GO:0007389	2E-06	5.636521318350934	ERBB4,FOXB1,INTU,LRP2,CYP26B1,ZIC3,CCDC39,GBX2,WNT5A,ZIC1,MEIS1,DNAI1,NR2F2,HES5,IRX2,WLS,RFX3,IRX1,DLL1,PTCH1,HHIP,PIFO,BMPR1B,RFX4,OFD1,MNS1,WNT2B,TBC1D32,SMAD6,AXIN2,LFNG,ASCL1,ACVR2B,NPHP3,WNT3,BTG2,TTC8,FGF2
GO:BP	central nervous system development	GO:0007417	3E-06	5.593987286247123	ERBB4,PTF1A,GSX1,PRDM13,EN2,OLIG3,CFAP43,HESX1,LHX5,FOXB1,ZIC4,SOX13,INTU,DMBX1,LRP2,ZIC5,ZIC3,CCDC39,GBX2,NTRK2,PTN,WNT5A,ZIC1,FOXP2,MEIS1,NDNF,CEP290,PLP1,PROX1,NR2F2,HES5,WLS,RORA,CDK6,ZIC2,DLL1,PTCH1,BMPR1B,AGER,RFX4,TTPA,NHLH2,DUSP10,TRPC4,CCDC14,WNT2B,CXCR4,C5AR1,POU3F2,GPM6B,ASCL1,STXBP3,WNT3,B3GNT5,CDK5RAP3,SPEF2,BTG2,TTC8,JRKL,FGF2,ACKR3,CHD7
GO:BP	cell projection organization	GO:0030030	4E-06	5.378995232759171	ROBO3,PRTG,OLIG3,TPBG,CFAP70,CFAP43,DNAH8,FOXB1,CFAP54,INTU,ADGRV1,KEL,LRP2,STRC,ENPP2,MSTN,CCDC39,GBX2,NTRK2,PTN,WNT5A,CFAP44,MAPK15,RPGR,NDNF,CEP290,PDPN,PLP1,DNAH7,DNAI1,HES5,CCDC78,CFAP69,RFX3,GOLGA4,PTCH1,LCA5L,PIFO,NR2F1,BMPR1B,AGER,RFX4,CEP162,AK7,SLITRK2,CDNF,OFD1,MNS1,CRABP2,TBC1D32,CEP83,CXCR4,DNAH2,POU3F2,SCARF1,DNHD1,GPM6B,IQUB,DRC7,LCA5,PARD6B,NPHP3,EPHA3,RPGRIP1,SPATA6,WNT3,MAK,IFT81,OCLN,PDLIM5,NRXN3,TTC26,MTR,SPEF2,EFHC2,BTG2,WDR90,TTC8,NPHP1,SKIL,IFT80,CAPRIN2
GO:BP	tissue development	GO:0009888	9E-06	5.070460213983439	ERBB4,PTF1A,EDNRB,ALOX15,ANKRD33,HESX1,FOXB1,COL7A1,INTU,ITGB8,ADGRV1,NPHS1,KEL,LRP2,STRC,TFAP2B,THRB,CYP26B1,ZIC3,PHEX,MSTN,CCDC39,GBX2,PTN,WNT5A,APCDD1,MEIS1,CEP290,PDPN,EBF2,MST1,PROX1,MAF,BDH2,NPPC,NR2F2,HES5,IRX2,CCDC78,WLS,LGR4,RFX3,CDK6,IRX1,DLL1,PTCH1,HHIP,CCND1,CCDC154,PIFO,NPPA,BMPR1B,MYH15,KITLG,HSF4,MEGF10,DUSP10,WNT2B,CRABP2,TBC1D32,CXCR4,TNNI3,SMAD6,AXIN2,POU3F2,KRT10,LFNG,GPM6B,ASCL1,USH2A,JAG1,ACVR2B,MTM1,NPHP3,EPHA3,RBBP6,WNT3,PDCD4,PDLIM5,DSG2,EDA2R,SCX,COL11A2,ADAM9,BTG2,RAP2C,EXPH5,TTC8,FGF2,AMH,NPHP1,CHD7,ACADVL,SKIL,TGM1,IFT80
GO:BP	sensory organ development	GO:0007423	9E-06	5.042402724162064	PTF1A,OLIG3,CABP4,HESX1,INTU,RPE65,ADGRV1,STRC,TFAP2B,PDE6C,THRB,CYP26B1,ZIC3,GBX2,NTRK2,WNT5A,ZIC1,MEIS1,CEP290,PROX1,MAF,HES5,SMOC1,DLL1,BMPR1B,MYH15,HSF4,WNT2B,TBC1D32,ASCL1,USH2A,JAG1,ACVR2B,RPGRIP1,CRYBG3,USP45,TSPAN12,INSIG2,TTC8,FGF2,NPHP1,CHD7,SKIL
GO:BP	nervous system development	GO:0007399	1E-05	5.016300073230232	ROBO3,ERBB4,PTF1A,GSX1,PAX3,EDNRB,PRDM13,EN2,PRTG,OLIG3,TPBG,CABP4,CFAP43,HESX1,NRG4,LHX5,FOXB1,ZIC4,SOX13,INTU,IRX5,ADGRV1,KEL,DMBX1,LRP2,STRC,TFAP2B,PDE6C,THRB,ZIC5,ZIC3,CCDC39,GBX2,NTRK2,PTN,WNT5A,ZIC1,APCDD1,NRBP2,FOXP2,MEIS1,NDNF,CEP290,PLP1,NTN5,PROX1,NR2F2,HES5,IRX2,WLS,RORA,PLAG1,CDK6,IRX1,GNRH1,ZIC2,GOLGA4,DLL1,PTCH1,HHIP,CCND1,NR2F1,BMPR1B,AGER,RFX4,TTPA,QKI,SLITRK2,NHLH2,CDNF,DUSP10,TRPC4,CCDC14,WNT2B,CRABP2,PTPN13,TBC1D32,CXCR4,C5AR1,RASSF10,POU3F2,SCARF1,GPM6B,ASCL1,USH2A,JAG1,STXBP3,PARD6B,NPHP3,PCDHA7,EPHA3,UFL1,RPGRIP1,WNT3,ST8SIA4,ACSL6,B3GNT5,CDK5RAP3,PDLIM5,NRXN3,HLTF,MTR,SPEF2,REST,EFHC2,BTG2,TTC8,JRKL,FGF2,ACKR3,CHD7,SKIL,CAPRIN2

GO:BP	camera-type eye development	GO:0043010	1E-05	4.986161417975029	PTF1A,CABP4,HESX1,RPE65,TFAP2B,PDE6C,THRB,NTRK2,WNT5A,MEIS1,CEP290,PROX1,MAF,HES5,DLL1,BMPR1B,MYH15,HSF4,WNT2B,TBC1D32,JAG1,ACVR2B,RPGRIP1,CRYBG3,USP45,TSPAN12,TTC8,FGF2,NPHP1,CHD7,SKIL
GO:BP	neurogenesis	GO:0022008	1E-05	4.848244154638219	ROBO3,ERBB4,PTF1A,GSX1,EDNRB,PRDM13,EN2,PRTG,OLIG3,TPBG,CABP4,LHX5,FOXB1,SOX13,IRX5,ADGRV1,KEL,LRP2,STRC,PDE6C,THRB,ZIC3,GBX2,NTRK2,PTN,WNT5A,APCDD1,NRBP2,MEIS1,NDNF,CEP290,PLP1,NTN5,PROX1,NR2F2,HES5,IRX2,RORA,PLAG1,CDK6,IRX1,GNRH1,GOLGA4,DLL1,PTCH1,HHIP,CCND1,NR2F1,BMPR1B,AGER,SLITRK2,NHLH2,CDNF,DUSP10,TRPC4,WNT2B,CRABP2,CXCR4,C5AR1,RASSF10,POU3F2,SCARF1,GPM6B,ASCL1,USH2A,JAG1,PARD6B,EPHA3,UFL1,RPGRIP1,WNT3,ACSL6,CDK5RAP3,PDLIM5,NRXN3,HLTF,MTR,REST,EFHC2,BTG2,TTC8,FGF2,CHD7,SKIL,CAPRIN2
GO:BP	plasma membrane bounded cell projection organization	GO:0120036	1E-05	4.832352411304042	ROBO3,PRTG,OLIG3,TPBG,CFAP70,CFAP43,DNAH8,FOXB1,CFAP54,INTU,ADGRV1,KEL,LRP2,STRC,ENPP2,MSTN,CCDC39,GBX2,NTRK2,PTN,WNT5A,CFAP44,MAPK15,RPGR,NDNF,CEP290,PDPN,PLP1,DNAH7,DNAI1,HES5,CFAP69,RFX3,GOLGA4,PTCH1,LCA5L,PIFO,NR2F1,BMPR1B,AGER,RFX4,CEP162,SLITRK2,CDNF,OFD1,MNS1,CRABP2,TBC1D32,CEP83,CXCR4,DNAH2,POU3F2,SCARF1,DNHD1,GPM6B,IQUB,DRC7,LCA5,PARD6B,NPHP3,EPHA3,RPGRIP1,SPATA6,WNT3,MAK,IFT81,OCLN,PDLIM5,NRXN3,TTC26,MTR,SPEF2,EFHC2,BTG2,WDR90,TTC8,SKIL,IFT80,CAPRIN2
GO:BP	multicellular organism development	GO:0007275	2E-05	4.605350901187922	ROBO3,ERBB4,PTF1A,GSX1,PAX3,EDNRB,PRDM13,EN2,PRTG,OLIG3,TPBG,CABP4,CFAP43,HESX1,NRG4,SP9,LHX5,FOXB1,ZIC4,SOX13,ANGPTL3,COL7A1,INTU,ANGPT1,ITGB8,RPE65,IRX5,ADGRV1,ADAMTS6,NPHS1,KEL,DMBX1,INPP5D,LRP2,STRC,TFAP2B,PDE6C,THRB,ZIC5,CYP26B1,ZIC3,PHEX,CCDC39,GBX2,NTRK2,INO80B,MPL,PTN,WNT5A,ZIC1,APCDD1,NRBP2,FOXP2,MEIS1,NDNF,CEP290,PDPN,PLP1,NTN5,MST1,PROX1,MAF,TRIM45,DNAI1,NPPC,NR2F2,HES5,IRX2,RGN,WLS,LAG3,RORA,PLAG1,LGR4,RFX3,CDK6,IRX1,GNRH1,CALCRL,ZIC2,SMOC1,GOLGA4,DLL1,PTCH1,HHIP,CCND1,CCDC154,PIFO,NR2F1,NPPA,BMPR1B,MYH15,AGER,RFX4,COL4A3,KITLG,TTPA,HSF4,QKI,SLITRK2,NHLH2,DHRS3,CDNF,OFD1,DUSP10,MNS1,TRPC4,CCDC14,WNT2B,CRABP2,PTPN13,TBC1D32,CXCR4,TNNI3,SMAD6,AXIN2,ATAD5,C5AR1,RASSF10,POU3F2,SCARF1,LFNG,ANG,GPM6B,ASCL1,USH2A,JAG1,ACVR2B,CNTRL,STXBP3,PRICKLE4,PARD6B,NPHP3,PCDHA7,EPHA3,L3MBTL1,UFL1,RPGRIP1,HIF3A,RBBP6,EAF2,WNT3,ST8SIA4,ACSL6,CRYBG3,B3GNT5,CDK5RAP3,PDCD4,PDLIM5,DSG2,USP45,NKAP,NRXN3,HLTF,TSPAN12,SCX,MTR,INSIG2,COL11A2,TMF1,SPEF2,REST,TET1,EFHC2,BTG2,TTC8,RIN2,JRKL,FGF2,RRM2B,AMH,ACKR3,NPHP1,CHD7,SKIL,IFT80,CAPRIN2
GO:BP	regionalization	GO:0003002	3E-05	4.594214081118448	FOXB1,INTU,LRP2,CYP26B1,ZIC3,CCDC39,GBX2,WNT5A,DNAI1,NR2F2,HES5,IRX2,WLS,RFX3,IRX1,DLL1,PTCH1,HHIP,PIFO,BMPR1B,RFX4,OFD1,MNS1,WNT2B,TBC1D32,SMAD6,AXIN2,LFNG,ASCL1,ACVR2B,NPHP3,WNT3,BTG2,FGF2
GO:BP	anatomical structure morphogenesis	GO:0009653	3E-05	4.557788593524345	ROBO3,ERBB4,PTF1A,PAX3,PRTG,TPBG,PAX7,CABP4,HESX1,SP9,LHX5,FOXB1,SOX13,ANGPTL3,COL7A1,INTU,ANGPT1,ITGB8,RPE65,IRX5,NPHS1,KEL,LRP2,STRC,TFAP2B,PDE6C,THRB,CYP26B1,ZIC3,ENPP2,PHEX,AQP6,CCDC39,GBX2,NTRK2,C11ORF65,MPL,PTN,WNT5A,ZIC1,APCDD1,MEIS1,NDNF,CEP290,PDPN,MAEL,MST1,PROX1,NPPC,UNC13D,NR2F2,HES5,ACRBP,IRX2,WLS,RORA,STRIP2,PLAG1,LGR4,IRX1,CALCRL,GOLGA4,DLL1,PTCH1,CRISPLD1,HHIP,CCDC154,NPPA,BMPR1B,COL4A3,QKI,SLITRK2,DHRS3,WNT2B,CRABP2,TBC1D32,CXCR4,TNNI3,SMAD6,AXIN2,C5AR1,DKKL1,POU3F2,LFNG,ANG,JAG1,ACVR2B,MTM1,C6ORF25,PARD6B,NPHP3,EPHA3,RPGRIP1,HIF3A,ARHGAP15,WNT3,PDCD4,PDLIM5,NRXN3,TSPAN12,SCX,INSIG2,COL11A2,ADAM9,TMF1,SPEF2,TET1,TTC8,RIN2,FGF2,AMH,ACKR3,NPHP1,CHD7,SKIL,IFT80,CAPRIN2
GO:BP	sensory organ morphogenesis	GO:0090596	3E-05	4.552420055407963	PTF1A,CABP4,HESX1,INTU,RPE65,STRC,TFAP2B,PDE6C,THRB,CYP26B1,ZIC3,GBX2,NTRK2,WNT5A,ZIC1,MEIS1,CEP290,PROX1,DLL1,WNT2B,JAG1,RPGRIP1,TSPAN12,INSIG2,TTC8,FGF2,CHD7
GO:BP	animal organ morphogenesis	GO:0009887	3E-05	4.550566931060515	ERBB4,PTF1A,PAX3,CABP4,HESX1,INTU,RPE65,IRX5,LRP2,STRC,TFAP2B,PDE6C,THRB,CYP26B1,ZIC3,PHEX,AQP6,CCDC39,GBX2,NTRK2,PTN,WNT5A,ZIC1,APCDD1,MEIS1,CEP290,MST1,PROX1,NPPC,HES5,IRX2,WLS,PLAG1,LGR4,IRX1,DLL1,PTCH1,HHIP,CCDC154,BMPR1B,DHRS3,WNT2B,TNNI3,SMAD6,AXIN2,LFNG,JAG1,ACVR2B,NPHP3,RPGRIP1,PDCD4,TSPAN12,SCX,INSIG2,COL11A2,SPEF2,TTC8,FGF2,CHD7,IFT80

GO:BP	renal system development	GO:0072001	3E-05	4.520088	ERBB4,EDNRB,ANGPT1,ADAMTS6,NPHS1,LRP2,TFAP2B,CYP26B1,WNT5A,CEP290,PROX1,HES5,IRX2,RGN,LGR4,IRX1,DLL1,PTCH1,COL4A3,WNT2B,TBC1D32,SMAD6,JAG1,ACVR2B,CNTRL,NPHP3,TTC8,FGF2,RRM2B
GO:BP	kidney development	GO:0001822	6E-05	4.2280838333408175	ERBB4,EDNRB,ANGPT1,ADAMTS6,NPHS1,LRP2,TFAP2B,CYP26B1,WNT5A,CEP290,PROX1,HES5,IRX2,RGN,LGR4,IRX1,DLL1,PTCH1,COL4A3,WNT2B,TBC1D32,SMAD6,JAG1,ACVR2B,CNTRL,NPHP3,FGF2,RRM2B
GO:BP	head development	GO:0060322	6E-05	4.195139709396897	ERBB4,PTF1A,GSX1,PRDM13,EN2,CFAP43,HESX1,LHX5,FOXB1,DMBX1,LRP2,ZIC3,CCDC39,GBX2,NTRK2,WNT5A,ZIC1,FOXP2,MEIS1,NDNF,CEP290,PLP1,PROX1,NR2F2,HES5,WLS,RORA,CDK6,ZIC2,DLL1,PTCH1,CRISPLD1,RFX4,NHLH2,CCDC14,WNT2B,CXCR4,POU3F2,ASCL1,STXBP3,WNT3,B3GNT5,CDK5RAP3,SCX,SPEF2,BTG2,TTC8,FGF2,CHD7
GO:BP	brain development	GO:0007420	6E-05	4.192684845339291	ERBB4,PTF1A,GSX1,PRDM13,EN2,CFAP43,HESX1,LHX5,FOXB1,DMBX1,LRP2,ZIC3,CCDC39,GBX2,NTRK2,WNT5A,ZIC1,FOXP2,MEIS1,NDNF,CEP290,PLP1,PROX1,NR2F2,HES5,WLS,RORA,CDK6,ZIC2,DLL1,PTCH1,RFX4,NHLH2,CCDC14,WNT2B,CXCR4,POU3F2,ASCL1,STXBP3,WNT3,B3GNT5,CDK5RAP3,SPEF2,BTG2,TTC8,FGF2,CHD7
GO:BP	microtubule-based movement	GO:0007018	7E-05	4.1845462579188535	DNAH12,ATP1A4,CFAP70,CFAP43,DNAH8,CFAP54,INTU,CCDC39,CFAP44,RPGR,DNAH7,MST1,DNAI1,CENPE,RGN,CFAP69,RFX3,LCA5L,OFD1,MNS1,DNAH2,DYNLRB2,DNHD1,DRC7,DNAH6,LCA5,NPHP3,MAK,IFT81,TTC26,TMF1,SPEF2,IFT80
GO:BP	eye development	GO:0001654	7E-05	4.126291962772317	PTF1A,CABP4,HESX1,RPE65,TFAP2B,PDE6C,THRB,NTRK2,WNT5A,MEIS1,CEP290,PROX1,MAF,HES5,SMOC1,DLL1,BMPR1B,MYH15,HSF4,WNT2B,TBC1D32,JAG1,ACVR2B,RPGRIP1,CRYBG3,USP45,TSPAN12,TTC8,FGF2,NPHP1,CHD7,SKIL
GO:BP	visual system development	GO:0150063	9E-05	4.025672600035307	PTF1A,CABP4,HESX1,RPE65,TFAP2B,PDE6C,THRB,NTRK2,WNT5A,MEIS1,CEP290,PROX1,MAF,HES5,SMOC1,DLL1,BMPR1B,MYH15,HSF4,WNT2B,TBC1D32,JAG1,ACVR2B,RPGRIP1,CRYBG3,USP45,TSPAN12,TTC8,FGF2,NPHP1,CHD7,SKIL
GO:BP	cilium-dependent cell motility	GO:0060285	1E-04	3.99490324	ATP1A4,CFAP43,DNAH8,CFAP54,CCDC39,CFAP44,DNAH7,MST1,DNAI1,RGN,CFAP69,RFX3,MNS1,DNAH2,DNHD1,DRC7,IFT81,TMF1,SPEF2
GO:BP	cilium or flagellum-dependent cell motility	GO:0001539	1E-04	3.99490324	ATP1A4,CFAP43,DNAH8,CFAP54,CCDC39,CFAP44,DNAH7,MST1,DNAI1,RGN,CFAP69,RFX3,MNS1,DNAH2,DNHD1,DRC7,IFT81,TMF1,SPEF2
GO:BP	sensory system development	GO:0048880	1E-04	3.8774605228615493	PTF1A,CABP4,HESX1,RPE65,TFAP2B,PDE6C,THRB,NTRK2,WNT5A,MEIS1,CEP290,PROX1,MAF,HES5,SMOC1,DLL1,BMPR1B,MYH15,HSF4,WNT2B,TBC1D32,JAG1,ACVR2B,RPGRIP1,CRYBG3,USP45,TSPAN12,TTC8,FGF2,NPHP1,CHD7,SKIL
GO:BP	generation of neurons	GO:0048699	2E-04	3.813536798043159	ROBO3,ERBB4,PTF1A,GSX1,EDNRB,PRDM13,EN2,PRTG,OLIG3,TPBG,CABP4,LHX5,FOXB1,IRX5,ADGRV1,KEL,LRP2,STRC,PDE6C,THRB,ZIC3,GBX2,NTRK2,PTN,WNT5A,NRBP2,MEIS1,NDNF,CEP290,PLP1,PROX1,NR2F2,HES5,IRX2,RORA,CDK6,IRX1,GNRH1,GOLGA4,DLL1,PTCH1,HHIP,CCND1,NR2F1,BMPR1B,AGER,SLITRK2,NHLH2,CDNF,WNT2B,CRABP2,CXCR4,POU3F2,SCARF1,GPM6B,ASCL1,USH2A,JAG1,PARD6B,EPHA3,RPGRIP1,WNT3,ACSL6,CDK5RAP3,PDLIM5,NRXN3,MTR,REST,EFHC2,BTG2,TTC8,FGF2,SKIL,CAPRIN2
GO:BP	regulation of developmental process	GO:0050793	2E-04	3.7453574195633297	PRAME,PRAMEF12,ERBB4,PTF1A,EDNRB,PRTG,TPBG,HESX1,GDPD2,SOX13,ANGPTL3,ABCA1,ITGB8,ADGRV1,KEL,INPP5D,LRP2,TFAP2B,CYP26B1,ENPP2,MSTN,GBX2,NTRK2,INO80B,C11ORF65,MPL,PTN,WNT5A,APCDD1,MEIS1,PDPN,MAEL,MST1,PROX1,MAF,NPPC,HES5,RGN,LAG3,RORA,STRIP2,PLAG1,LGR4,RFX3,CDK6,GNRH1,SMOC1,GOLGA4,DLL1,PTCH1,CCND1,BMPR1B,AGER,COL4A3,KITLG,TTPA,MEGF10,SLITRK2,GNB3,DUSP10,WNT2B,CRABP2,PTPN13,CXCR4,SMAD6,AXIN2,ATAD5,C5AR1,DKKL1,RASSF10,POU3F2,SCARF1,KRT10,LFNG,GPM6B,ASCL1,USH2A,JAG1,ACVR2B,MTM1,NPHP3,EPHA3,TOB1,L3MBTL1,WEE2,UFL1,ABCG1,ARHGAP15,EAF2,WNT3,CDK5RAP3,PDCD4,PDLIM5,NKAP,HLTF,TSPAN12,SCX,ADAM9,REST,TET1,BTG2,RIN2,FGF2,AMH,CHD7,SKIL,CAPRIN2
GO:BP	retina morphogenesis in camera-type eye	GO:0060042	3E-04	3.5535844364918057	PTF1A,CABP4,RPE65,TFAP2B,PDE6C,THRB,NTRK2,PROX1,DLL1,RPGRIP1,TSPAN12,TTC8

GO:BP	epithelium development	GO:0060429	3E-04	3.4967530159544316	ERBB4,EDNRB,HESX1,FOXB1,INTU,NPHS1,LRP2,STRC,TFAP2B,THRB,CYP26B1,ZIC3,CCDC39,GBX2,WNT5A,APCDD1,CEP290,PDPN,MST1,PROX1,MAF,BDH2,NPPC,NR2F2,HES5,IRX2,CCDC78,LGR4,RFX3,CDK6,IRX1,DLL1,PTCH1,HHIP,CCND1,PIFO,HSF4,DUSP10,WNT2B,TBC1D32,CXCR4,SMAD6,AXIN2,POU3F2,KRT10,LFNG,ASCL1,USH2A,JAG1,ACVR2B,NPHP3,RBBP6,WNT3,SCX,ADAM9,RAP2C,EXPH5,TTC8,FGF2,NPHP1,CHD7,ACADVL,SKIL,TGM1
GO:BP	motile cilium assembly	GO:0044458	3E-04	3.4767276036912014	CFAP43,CFAP54,INTU,CCDC39,CFAP44,CFAP69,MNS1,DNHD1,DRC7,SPATA6,IFT81,SPEF2
GO:BP	reproductive process	GO:0022414	4E-04	3.4326800551014545	ATP1A4,EDNRB,CFAP43,HESX1,MSH5-SAPCD1,GLIPR1L1,CFAP54,SPAG8,HFM1,IRX5,LRP2,THRB,CYP26B1,MSTN,CCDC39,CCNB3,PTN,WNT5A,CFAP44,MAPK15,ADAM28,NLRP14,MAEL,MST1,SPATA9,DNAI1,NPPC,NR2F2,ACRBP,RGN,TEX14,CFAP69,PLAG1,LGR4,GNRH1,PTCH1,NPPA,CATSPERG,BMPR1B,KITLG,DCST2,QKI,NHLH2,TDRP,MNS1,WNT2B,DCST1,DKKL1,DNHD1,LFNG,ANG,SMC1B,SPATA6L,JAG1,DRC7,WEE2,SPATA6,EAF2,WNT3,MAK,IFT81,CENPC,DSG2,ANKRD31,TTC26,PABPC1L,SCX,TMF1,SPEF2,AMH,NPHP1,CHD7,SKIL,FANCM
GO:BP	reproduction	GO:0000003	5E-04	3.3205294757189763	ATP1A4,EDNRB,CFAP43,HESX1,MSH5-SAPCD1,GLIPR1L1,CFAP54,SPAG8,HFM1,IRX5,LRP2,THRB,CYP26B1,MSTN,CCDC39,CCNB3,PTN,WNT5A,CFAP44,MAPK15,ADAM28,NLRP14,MAEL,MST1,SPATA9,DNAI1,NPPC,NR2F2,ACRBP,RGN,TEX14,CFAP69,PLAG1,LGR4,GNRH1,PTCH1,NPPA,CATSPERG,BMPR1B,KITLG,DCST2,QKI,NHLH2,TDRP,MNS1,WNT2B,DCST1,DKKL1,DNHD1,LFNG,ANG,SMC1B,SPATA6L,JAG1,DRC7,WEE2,SPATA6,EAF2,WNT3,MAK,IFT81,CENPC,DSG2,ANKRD31,TTC26,PABPC1L,SCX,TMF1,SPEF2,AMH,NPHP1,CHD7,SKIL,FANCM
GO:BP	cilium movement	GO:0003341	7E-04	3.1330308597344745	ATP1A4,CFAP70,CFAP43,CFAP54,CCDC39,CFAP44,DNAH7,MST1,DNAI1,RGN,CFAP69,RFX3,OFD1,MNS1,DNHD1,DRC7,NPHP3,IFT81,TMF1,SPEF2
GO:BP	neuron differentiation	GO:0030182	8E-04	3.115345522937571	ROBO3,ERBB4,PTF1A,GSX1,EDNRB,PRDM13,EN2,PRTG,OLIG3,TPBG,CABP4,LHX5,FOXB1,IRX5,ADGRV1,KEL,LRP2,STRC,PDE6C,THRB,ZIC3,GBX2,NTRK2,PTN,WNT5A,NRBP2,MEIS1,NDNF,CEP290,PLP1,PROX1,HES5,IRX2,RORA,IRX1,GOLGA4,DLL1,PTCH1,CCND1,NR2F1,BMPR1B,AGER,SLITRK2,NHLH2,CDNF,WNT2B,CRABP2,CXCR4,POU3F2,SCARF1,GPM6B,ASCL1,USH2A,JAG1,PARD6B,EPHA3,RPGRIP1,WNT3,CDK5RAP3,PDLIM5,NRXN3,MTR,REST,EFHC2,BTG2,TTC8,FGF2,SKIL,CAPRIN2
GO:BP	retina development in camera-type eye	GO:0060041	8E-04	3.0723569042991485	PTF1A,CABP4,RPE65,TFAP2B,PDE6C,THRB,NTRK2,PROX1,DLL1,BMPR1B,TBC1D32,ACVR2B,RPGRIP1,USP45,TSPAN12,TTC8,NPHP1,CHD7
GO:BP	camera-type eye morphogenesis	GO:0048593	0.001	2.9581812059968464	PTF1A,CABP4,RPE65,TFAP2B,PDE6C,THRB,NTRK2,WNT5A,MEIS1,PROX1,DLL1,WNT2B,JAG1,RPGRIP1,TSPAN12,TTC8
GO:BP	eye morphogenesis	GO:0048592	0.001	2.876964779608331	PTF1A,CABP4,RPE65,TFAP2B,PDE6C,THRB,NTRK2,WNT5A,MEIS1,CEP290,PROX1,DLL1,WNT2B,JAG1,RPGRIP1,TSPAN12,TTC8,FGF2
GO:BP	sperm flagellum assembly	GO:0120316	0.002	2.7138413481782386	CFAP43,CFAP54,CFAP44,CFAP69,MNS1,DNHD1,DRC7,IFT81,SPEF2
GO:BP	regulation of transcription by RNA polymerase II	GO:0006357	0.002	2.6336160139460616	PTF1A,GSX1,PAX3,EDNRB,PRDM13,EN2,OLIG3,PAX7,VGLL3,HESX1,SP9,LHX5,FOXB1,ZIC4,SOX13,SPAG8,IRX5,DMBX1,TFAP2B,THRB,ZIC5,ZIC3,ZNF83,GBX2,WNT5A,ZIC1,MUC1,EBF1,ZNF117,FOXP2,MEIS1,MAEL,EBF2,PROX1,MAF,NR2F2,HES5,IRX2,RORA,PLAG1,RFX3,CDK6,IRX1,AASS,TPR,ZIC2,DLL1,YY2,PTCH1,TARBP1,CCND1,NR2F1,BMPR1B,ZNF518A,RFX4,ZNF730,HSF4,NHLH2,ZNF107,YES1,ZNF90,SMAD6,POU3F2,ASCL1,POU2F2,JAG1,ACVR2B,NR3C2,ZNF69,DNMT3B,ZNF493,TOX3,ZNF404,HIF3A,EAF2,ZNF846,ZNF460,CDK5RAP3,ZNF780B,NKAP,HLTF,ZNF138,PRDM5,SCX,TMF1,REST,ZNF680,TET1,BTG2,BAZ2B,ZNF217,FGF2,PLAGL1,CHD7,ZNF273,CHD1,SKIL,PAXBP1,CAPRIN2,ZNF267,JMJD1C
GO:BP	pancreas development	GO:0031016	0.003	2.5329791931844587	PTF1A,ILDR2,ZIC3,CCDC39,WNT5A,PROX1,WLS,RFX3,CDK6,DLL1,ACVR2B,NPHP3

GO:BP	sexual reproduction	GO:0019953	0.003	2.4812103210233483	ATP1A4,CFAP43,MSH5-SAPCD1,GLIPR1L1,CFAP54,SPAG8,HFM1,CYP26B1,CCNB3,PTN,WNT5A,CFAP44,MAPK15,ADAM28,NLRP14,MAEL,MST1,SPATA9,NPPC,NR2F2,ACRBP,RGN,TEX14,CFAP69,LGR4,PTCH1,CATSPERG,BMPR1B,DCST2,QKI,TDRP,MNS1,DCST1,DKKL1,DNHD1,LFNG,ANG,SMC1B,SPATA6L,DRC7,WEE2,SPATA6,WNT3,MAK,IFT81,CENPC,ANKRD31,TTC26,PABPC1L,TMF1,SPEF2,AMH,NPHP1,SKIL,FANCM
GO:BP	regulation of RNA biosynthetic process	GO:2001141	0.004	2.428353183501484	PRAME,PRAMEF12,ERBB4,PTF1A,GSX1,PAX3,EDNRB,PRDM13,EN2,OLIG3,PAX7,VGLL3,HESX1,SP9,LHX5,FOXB1,ZIC4,SOX13,SPAG8,IRX5,DMBX1,TFAP2B,THRB,ZIC5,ZIC3,SAP25,MSTN,ZNF83,GBX2,INO80B,WNT5A,ZIC1,MUC1,EBF1,ZNF117,FOXP2,MEIS1,CEP290,MAEL,EBF2,PROX1,MAF,NR2F2,HES5,IRX2,ZNF334,RGN,RORA,PLAG1,LGR4,RFX3,CDK6,IRX1,GOLGB1,AASS,TPR,ZIC2,DLL1,YY2,PTCH1,TARBP1,HMGN5,CCND1,FANK1,NR2F1,ZNF483,BMPR1B,ZNF518A,AGER,RFX4,ZNF730,HSF4,NHLH2,ZNF107,YES1,CRABP2,ZNF90,SMAD6,POU3F2,ASCL1,POU2F2,JAG1,ACVR2B,NR3C2,ZNF69,DNMT3B,ZNF493,TOX3,ZNF624,ZNF404,BTAF1,TOB1,L3MBTL1,UFL1,CARD14,HIF3A,EAF2,TRIM22,ZNF846,MAK,ZNF460,TRIM5,CDK5RAP3,ZNF780B,PDCD4,NKAP,HLTF,EDA2R,ZNF138,PRDM5,SCX,TMF1,REST,ZNF680,TET1,BTG2,RAP2C,BAZ2B,ZNF217,FGF2,PLAGL1,AMH,INTS2,CHD7,ZNF273,CHD1,SKIL,PAXBP1,CAPRIN2,ZNF805,ZNF267,JMJD1C,TRIM52
GO:BP	plasma membrane bounded cell projection assembly	GO:0120031	0.004	2.418156816287245	CFAP70,CFAP43,DNAH8,CFAP54,INTU,MSTN,CCDC39,CFAP44,MAPK15,RPGR,CEP290,DNAH7,DNAI1,CFAP69,RFX3,RFX4,CEP162,OFD1,MNS1,TBC1D32,CEP83,DNAH2,DNHD1,IQUB,DRC7,NPHP3,RPGRIP1,SPATA6,MAK,IFT81,OCLN,TTC26,SPEF2,WDR90,TTC8,IFT80
GO:BP	developmental process involved in reproduction	GO:0003006	0.004	2.394667589167053	ATP1A4,CFAP43,HESX1,CFAP54,SPAG8,IRX5,LRP2,CYP26B1,PTN,WNT5A,CFAP44,ADAM28,NLRP14,MAEL,MST1,SPATA9,NPPC,NR2F2,ACRBP,RGN,CFAP69,PLAG1,LGR4,GNRH1,PTCH1,CATSPERG,BMPR1B,KITLG,QKI,NHLH2,TDRP,MNS1,WNT2B,DNHD1,LFNG,ANG,SPATA6L,DRC7,WEE2,SPATA6,EAF2,MAK,IFT81,TTC26,PABPC1L,SCX,TMF1,SPEF2,AMH,NPHP1,CHD7,SKIL
GO:BP	regulation of DNA-templated transcription	GO:0006355	0.004	2.3610635570273923	PRAME,PRAMEF12,ERBB4,PTF1A,GSX1,PAX3,EDNRB,PRDM13,EN2,OLIG3,PAX7,VGLL3,HESX1,SP9,LHX5,FOXB1,ZIC4,SOX13,SPAG8,IRX5,DMBX1,TFAP2B,THRB,ZIC5,ZIC3,SAP25,MSTN,ZNF83,GBX2,INO80B,WNT5A,ZIC1,MUC1,EBF1,ZNF117,FOXP2,MEIS1,CEP290,MAEL,EBF2,PROX1,MAF,NR2F2,HES5,IRX2,ZNF334,RORA,PLAG1,LGR4,RFX3,CDK6,IRX1,GOLGB1,AASS,TPR,ZIC2,DLL1,YY2,PTCH1,TARBP1,HMGN5,CCND1,FANK1,NR2F1,ZNF483,BMPR1B,ZNF518A,AGER,RFX4,ZNF730,HSF4,NHLH2,ZNF107,YES1,CRABP2,ZNF90,SMAD6,POU3F2,ASCL1,POU2F2,JAG1,ACVR2B,NR3C2,ZNF69,DNMT3B,ZNF493,TOX3,ZNF624,ZNF404,BTAF1,TOB1,L3MBTL1,UFL1,CARD14,HIF3A,EAF2,TRIM22,ZNF846,MAK,ZNF460,TRIM5,CDK5RAP3,ZNF780B,PDCD4,NKAP,HLTF,EDA2R,ZNF138,PRDM5,SCX,TMF1,REST,ZNF680,TET1,BTG2,RAP2C,BAZ2B,ZNF217,FGF2,PLAGL1,AMH,INTS2,CHD7,ZNF273,CHD1,SKIL,PAXBP1,CAPRIN2,ZNF805,ZNF267,JMJD1C,TRIM52
GO:BP	cell development	GO:0048468	0.005	2.3257697860376667	ROBO3,ERBB4,EDNRB,THEMIS,EN2,PRTG,OLIG3,TPBG,CABP4,CFAP43,FOXB1,CFAP54,SOX13,ANGPT1,ITGB8,IRX5,ADGRV1,NPHS1,KEL,INPP5D,LRP2,STRC,PDE6C,THRB,CYP26B1,GBX2,NTRK2,MPL,PTN,WNT5A,CFAP44,MEIS1,NDNF,CEP290,PLP1,MAEL,PROX1,MAF,NPPC,HES5,ACRBP,IRX2,LAG3,RORA,CFAP69,PLAG1,RFX3,CDK6,IRX1,GOLGA4,DLL1,PTCH1,NR2F1,BMPR1B,AGER,KITLG,TTPA,QKI,MEGF10,SLITRK2,NHLH2,CDNF,DUSP10,MNS1,WNT2B,CRABP2,CXCR4,AXIN2,C5AR1,RASSF10,POU3F2,SCARF1,DNHD1,LFNG,ANG,GPM6B,ASCL1,POU2F2,JAG1,DRC7,C6ORF25,PARD6B,EPHA3,L3MBTL1,WEE2,UFL1,RPGRIP1,WNT3,IFT81,CDK5RAP3,PDCD4,PDLIM5,NKAP,NRXN3,HLTF,TTC26,PABPC1L,MTR,TMF1,SPEF2,REST,EFHC2,BTG2,RAP2C,EXPH5,TTC8,FGF2,AMH,CHD7,SKIL,CAPRIN2
GO:BP	tube development	GO:0035295	0.005	2.272535529345526	EDNRB,HESX1,FOXB1,ANGPTL3,INTU,ANGPT1,ITGB8,LRP2,TFAP2B,THRB,ZIC3,PHEX,CCDC39,GBX2,NTRK2,WNT5A,MEIS1,NDNF,CEP290,PDPN,MST1,PROX1,NPPC,NR2F2,HES5,IRX2,RORA,LGR4,IRX1,CALCRL,DLL1,PTCH1,HHIP,PIFO,COL4A3,QKI,WNT2B,TBC1D32,CXCR4,TNNI3,SMAD6,C5AR1,ANG,ASCL1,JAG1,ACVR2B,NPHP3,HIF3A,NRXN3,TSPAN12,TTC8,RIN2,FGF2,ACKR3,CHD7

GO:BP	transcription by RNA polymerase II	GO:0006366	0.006	2.228817089539503	PTF1A,GSX1,PAX3,EDNRB,PRDM13,EN2,OLIG3,PAX7,VGLL3,HESX1,SP9,LHX5,FOXB1,ZIC4,SOX13,SPAG8,IRX5,DMBX1,TFAP2B,THRB,ZIC5,ZIC3,ZNF83,GBX2,WNT5A,ZIC1,MUC1,EBF1,ZNF117,FOXP2,MEIS1,MAEL,EBF2,PROX1,MAF,NR2F2,HES5,IRX2,RORA,PLAG1,RFX3,CDK6,IRX1,AASS,TPR,ZIC2,DLL1,YY2,PTCH1,TARBP1,CCND1,NR2F1,BMPR1B,ZNF518A,RFX4,ZNF730,HSF4,NHLH2,ZNF107,YES1,ZNF90,SMAD6,C5AR1,POU3F2,ASCL1,POU2F2,JAG1,ACVR2B,NR3C2,ZNF69,DNMT3B,ZNF493,TOX3,ZNF404,HIF3A,EAF2,ZNF846,ZNF460,CDK5RAP3,ZNF780B,NKAP,HLTF,ZNF138,PRDM5,SCX,TMF1,REST,ZNF680,TET1,BTG2,BAZ2B,ZNF217,FGF2,PLAGL1,INTS2,CHD7,ZNF273,CHD1,SKIL,PAXBP1,CAPRIN2,ZNF267,JMJD1C
GO:BP	cell projection assembly	GO:0030031	0.006	2.212266660187827	CFAP70,CFAP43,DNAH8,CFAP54,INTU,MSTN,CCDC39,CFAP44,MAPK15,RPGR,CEP290,DNAH7,DNAI1,CFAP69,RFX3,RFX4,CEP162,OFD1,MNS1,TBC1D32,CEP83,DNAH2,DNHD1,IQUB,DRC7,NPHP3,RPGRIP1,SPATA6,MAK,IFT81,OCLN,TTC26,SPEF2,WDR90,TTC8,IFT80
GO:BP	multicellular organism reproduction	GO:0032504	0.007	2.1858755185775443	ATP1A4,EDNRB,CFAP43,GLIPR1L1,CFAP54,SPAG8,THRB,CYP26B1,MSTN,PTN,CFAP44,ADAM28,NLRP14,MAEL,MST1,SPATA9,NPPC,NR2F2,ACRBP,RGN,TEX14,CFAP69,LGR4,PTCH1,CATSPERG,BMPR1B,DCST2,QKI,NHLH2,TDRP,MNS1,DCST1,DKKL1,DNHD1,ANG,SPATA6L,DRC7,WEE2,SPATA6,WNT3,MAK,IFT81,DSG2,TTC26,PABPC1L,TMF1,SPEF2,AMH,NPHP1,SKIL
GO:BP	RNA biosynthetic process	GO:0032774	0.007	2.1537682855775246	PRAME,PRAMEF12,ERBB4,PTF1A,GSX1,PAX3,EDNRB,PRDM13,EN2,OLIG3,PAX7,VGLL3,HESX1,SP9,LHX5,FOXB1,ZIC4,SOX13,SPAG8,IRX5,DMBX1,TFAP2B,THRB,ZIC5,ZIC3,SAP25,MSTN,ZNF83,GBX2,INO80B,WNT5A,ZIC1,MUC1,EBF1,ZNF117,FOXP2,MEIS1,CEP290,MAEL,EBF2,PROX1,MAF,NR2F2,HES5,IRX2,ZNF334,RGN,RORA,PLAG1,LGR4,RFX3,CDK6,IRX1,GOLGB1,AASS,TPR,ZIC2,DLL1,YY2,PTCH1,TARBP1,HMGN5,CCND1,FANK1,NR2F1,ZNF483,BMPR1B,ZNF518A,AGER,RFX4,ZNF730,HSF4,NHLH2,ZNF107,YES1,ATG16L2,CRABP2,ZNF90,SMAD6,C5AR1,POU3F2,ANG,ASCL1,POU2F2,JAG1,ACVR2B,NR3C2,ZNF69,DNMT3B,ZNF493,TOX3,ZNF624,ZNF404,BTAF1,TOB1,L3MBTL1,UFL1,CARD14,HIF3A,EAF2,TRIM22,ZNF846,MAK,ZNF460,TRIM5,CDK5RAP3,ZNF780B,PDCD4,NKAP,HLTF,EDA2R,ZNF138,PRDM5,SCX,TMF1,REST,ZNF680,TET1,BTG2,RAP2C,BAZ2B,ZNF217,FGF2,PLAGL1,AMH,INTS2,CHD7,ZNF273,CHD1,SKIL,PAXBP1,CAPRIN2,ZNF805,ZNF267,JMJD1C,TRIM52
GO:BP	flagellated sperm motility	GO:0030317	0.0100857000333335896	1.9962939526754224	ATP1A4,CFAP43,CFAP54,CCDC39,CFAP44,MST1,DNAI1,RGN,CFAP69,MNS1,DNHD1,DRC7,IFT81,TMF1,SPEF2
GO:BP	sperm motility	GO:0097722	0.0100857000333335896	1.9962939526754224	ATP1A4,CFAP43,CFAP54,CCDC39,CFAP44,MST1,DNAI1,RGN,CFAP69,MNS1,DNHD1,DRC7,IFT81,TMF1,SPEF2
GO:BP	microtubule-based process	GO:0007017	0.0107821167634453 78	1.9672959693497645	DNAH12,ATP1A4,GOLGA8B,CFAP70,CFAP43,DNAH8,CFAP54,INTU,CCDC102B,CCDC39,CFAP44,MAPK15,RPGR,DNAH7,MST1,DNAI1,CENPE,HEPACAM2,CCDC78,RGN,CFAP69,RFX3,TPR,LCA5L,FIGNL2,CCDC88B,OFD1,MNS1,DNAH2,DYNLRB2,DNHD1,DRC7,DNAH6,LCA5,PARD6B,NPHP3,EPHA3,CEP44,PARD3B,MAK,IFT81,OCLN,SPICE1,TTC26,SASS6,TMF1,SPEF2,GCC2,IFT80
GO:BP	DNA-templated transcription	GO:0006351	0.0108811804002039 64	1.9633239894275338	PRAME,PRAMEF12,ERBB4,PTF1A,GSX1,PAX3,EDNRB,PRDM13,EN2,OLIG3,PAX7,VGLL3,HESX1,SP9,LHX5,FOXB1,ZIC4,SOX13,SPAG8,IRX5,DMBX1,TFAP2B,THRB,ZIC5,ZIC3,SAP25,MSTN,ZNF83,GBX2,INO80B,WNT5A,ZIC1,MUC1,EBF1,ZNF117,FOXP2,MEIS1,CEP290,MAEL,EBF2,PROX1,MAF,NR2F2,HES5,IRX2,ZNF334,RORA,PLAG1,LGR4,RFX3,CDK6,IRX1,GOLGB1,AASS,TPR,ZIC2,DLL1,YY2,PTCH1,TARBP1,HMGN5,CCND1,FANK1,NR2F1,ZNF483,BMPR1B,ZNF518A,AGER,RFX4,ZNF730,HSF4,NHLH2,ZNF107,YES1,CRABP2,ZNF90,SMAD6,C5AR1,POU3F2,ANG,ASCL1,POU2F2,JAG1,ACVR2B,NR3C2,ZNF69,DNMT3B,ZNF493,TOX3,ZNF624,ZNF404,BTAF1,TOB1,L3MBTL1,UFL1,CARD14,HIF3A,EAF2,TRIM22,ZNF846,MAK,ZNF460,TRIM5,CDK5RAP3,ZNF780B,PDCD4,NKAP,HLTF,EDA2R,ZNF138,PRDM5,SCX,TMF1,REST,ZNF680,TET1,BTG2,RAP2C,BAZ2B,ZNF217,FGF2,PLAGL1,AMH,INTS2,CHD7,ZNF273,CHD1,SKIL,PAXBP1,CAPRIN2,ZNF805,ZNF267,JMJD1C,TRIM52
GO:BP	organelle assembly	GO:0070925	0.0113116438442013 21	1.9464742774273998	GOLGA8B,CFAP70,CFAP43,DNAH8,CFAP54,INTU,CCDC39,MAP1LC3B2,CFAP44,MAPK15,RPGR,CEP290,DNAH7,PROX1,DNAI1,CENPE,ACRBP,CCDC78,CFAP69,RFX3,TPR,RFX4,CEP162,OFD1,MNS1,ATG16L2,TBC1D32,CEP83,DNAH2,DNHD1,MTM1,IQUB,DRC7,NPHP3,CEP44,WEE2,RPGRIP1,SPATA6,MAK,IFT81,CENPC,SPICE1,NRXN3,TTC26,SASS6,TMF1,SPEF2,WDR90,TTC8,IFT80

GO:BP	forebrain development	GO:0030900	0.0139222805422620 36	1.856289619194051	ERBB4,GSX1,PRDM13,HESX1,LHX5,FOXB1,LRP2,ZIC3,GBX2,NTRK2,WNT5A,ZIC1,FOXP2,NDNF,PROX1,NR2F2,HES5,CDK6,RFX4,NHLH2,WNT2B,CXCR4,POU3F2,ASCL1,BTG2,TTC8,FGF2,CHD7
GO:BP	ossification	GO:0001503	0.0145693106143909 34	1.8365609975401798	ALOX15,GDPD2,INTU,ADGRV1,PHEX,PTN,WNT5A,NPPC,LGR4,CDK6,SMOC1,PTCH1,CCDC154,BMPR1B,DHRS3,SMAD6,AXIN2,GPM6B,JAG1,ACVR2B,TOB1,UFL1,WNT3,SCX,COL11A2,REST,FGF2,IFT80
GO:BP	nucleobase-containing compound biosynthetic process	GO:0034654	0.0161527685055202 24	1.7917530310030643	PRAME,PRAMEF12,ERBB4,PTF1A,GSX1,PAX3,EDNRB,PRDM13,EN2,OLIG3,PAX7,VGLL3,HESX1,PDK4,SP9,LHX5,FOXB1,ZIC4,SOX13,SPAG8,IRX5,DMBX1,USP43,TFAP2B,THRB,ZIC5,ZIC3,SAP25,MSTN,ZNF83,GBX2,INO80B,WNT5A,ZIC1,MUC1,MAPK15,EBF1,ZNF117,FOXP2,MEIS1,CEP290,MAEL,EBF2,PROX1,MAF,NPPC,NR2F2,HES5,IRX2,ZNF334,RGN,RORA,PLAG1,LGR4,RFX3,CDK6,IRX1,GOLGB1,AASS,TPR,ZIC2,DLL1,YY2,PTCH1,TARBP1,HMGN5,CCND1,FANK1,NR2F1,NPPA,ZNF483,BMPR1B,ZNF518A,AGER,RFX4,ZNF730,HSF4,AK7,NHLH2,ZNF107,YES1,ATG16L2,CRABP2,ZNF90,SMAD6,C5AR1,POU3F2,ANG,ASCL1,POLI,POU2F2,JAG1,ACVR2B,NR3C2,ZNF69,DNMT3B,ZNF493,TOX3,ZNF624,ZNF404,BTAF1,TOB1,L3MBTL1,UFL1,CARD14,HIF3A,EAF2,TRIM22,ZNF846,MAK,ACSL6,ZNF460,TRIM5,CDK5RAP3,ZNF780B,PDCD4,NKAP,HLTF,EDA2R,ZNF138,PRDM5,SCX,TMF1,REST,ZNF680,TET1,BTG2,RAP2C,NADK2,BAZ2B,ZNF217,FGF2,PLAGL1,GFPT2,RRM2B,AMH,INTS2,CHD7,ZNF273,CHD1,SKIL,PAXBP1,CAPRIN2,ZNF805,ZNF267,JMJD1C,TRIM52
GO:BP	regulation of cell differentiation	GO:0045595	0.017	1.7740277658243695	PRAME,PRAMEF12,EDNRB,PRTG,GDPD2,SOX13,ABCA1,ADGRV1,INPP5D,LRP2,TFAP2B,CYP26B1,MSTN,NTRK2,MPL,PTN,WNT5A,MEIS1,PDPN,PROX1,MAF,NPPC,HES5,LAG3,RORA,PLAG1,RFX3,CDK6,SMOC1,GOLGA4,DLL1,PTCH1,CCND1,BMPR1B,AGER,KITLG,TTPA,MEGF10,GNB3,DUSP10,CRABP2,CXCR4,SMAD6,AXIN2,DKKL1,RASSF10,POU3F2,ASCL1,USH2A,JAG1,ACVR2B,NPHP3,EPHA3,TOB1,L3MBTL1,WEE2,UFL1,ABCG1,WNT3,CDK5RAP3,PDCD4,NKAP,HLTF,REST,BTG2,RIN2,FGF2,CHD7,SKIL,CAPRIN2
GO:BP	neural retina development	GO:0003407	0.0222257470383135 92	1.6531436328700118	PTF1A,CABP4,RPE65,TFAP2B,PDE6C,THRB,NTRK2,RPGRIP1,USP45,TSPAN12,TTC8
GO:BP	loop of Henle development	GO:0072070	0.0224061886421181 96	1.649632011937505	HES5,IRX2,IRX1,DLL1,JAG1
GO:BP	cilium movement involved in cell motility	GO:0060294	0.024	1.623758893760665	ATP1A4,CFAP43,CFAP54,CCDC39,CFAP44,MST1,DNAI1,RGN,CFAP69,MNS1,DNHD1,DRC7,IFT81,TMF1,SPEF2
GO:BP	axoneme assembly	GO:0035082	0.0258301867693953 37	1.5878724535779591	CFAP43,DNAH8,CCDC39,CFAP44,DNAH7,DNAI1,CFAP69,MNS1,DNAH2,DRC7,TTC26,SPEF2
GO:BP	heterocycle biosynthetic process	GO:0018130	0.027	1.5610313333404995	PRAME,PRAMEF12,ERBB4,PTF1A,GSX1,PAX3,EDNRB,PRDM13,EN2,OLIG3,PAX7,VGLL3,HESX1,PDK4,SP9,LHX5,FOXB1,ZIC4,SOX13,SPAG8,IRX5,DMBX1,USP43,TFAP2B,THRB,ZIC5,ZIC3,SAP25,MSTN,ZNF83,GBX2,INO80B,WNT5A,ZIC1,MUC1,MAPK15,EBF1,ZNF117,FOXP2,MEIS1,CEP290,MAEL,EBF2,PROX1,MAF,NPPC,NR2F2,HES5,IRX2,ZNF334,RGN,RORA,PLAG1,LGR4,RFX3,CDK6,IRX1,GOLGB1,AASS,TPR,ZIC2,DLL1,YY2,PTCH1,TARBP1,HMGN5,CCND1,FANK1,NR2F1,NPPA,ZNF483,BMPR1B,ZNF518A,AGER,RFX4,ZNF730,HSF4,AK7,NHLH2,ZNF107,YES1,ATG16L2,CRABP2,ZNF90,SMAD6,C5AR1,POU3F2,ANG,ASCL1,POLI,POU2F2,JAG1,ACVR2B,NR3C2,ZNF69,DNMT3B,ZNF493,TOX3,ZNF624,ZNF404,NOXRED1,BTAF1,TOB1,L3MBTL1,UFL1,CARD14,HIF3A,EAF2,TRIM22,ZNF846,MAK,ACSL6,ZNF460,TRIM5,CDK5RAP3,ZNF780B,PDCD4,NKAP,HLTF,EDA2R,ZNF138,PRDM5,SCX,TMF1,REST,ZNF680,TET1,BTG2,RAP2C,NADK2,BAZ2B,ZNF217,FGF2,PLAGL1,GFPT2,RRM2B,AMH,INTS2,CHD7,ZNF273,CHD1,SKIL,PAXBP1,CAPRIN2,ZNF805,ZNF267,JMJD1C,TRIM52

GO:BP	organic cyclic compound biosynthetic process	GO:1901362	0.036	1.4482752977042528	PRAME,PRAMEF12,ERBB4,PTF1A,GSX1,PAX3,EDNRB,PRDM13,EN2,OLIG3,PAX7,VGLL3,HESX1,PDK4,SP9,LHX5,FOXB1,ZIC4,SOX13,SPAG8,IRX5,DMBX1,USP43,TFAP2B,THRB,ZIC5,ZIC3,SAP25,MSTN,ZNF83,GBX2,INO80B,WNT5A,CYP39A1,ZIC1,MUC1,MAPK15,EBF1,ZNF117,FOXP2,MEIS1,CEP290,MAEL,EBF2,PROX1,MAF,NPPC,NR2F2,HES5,IRX2,ZNF334,RGN,RORA,PLAG1,LGR4,RFX3,CDK6,IRX1,GOLGB1,AASS,TPR,ZIC2,DLL1,YY2,PTCH1,TARBP1,HMGN5,CCND1,FANK1,NR2F1,NPPA,ZNF483,BMPR1B,ZNF518A,AGER,RFX4,ZNF730,HSF4,AK7,NHLH2,ZNF107,YES1,ATG16L2,CRABP2,ZNF90,SMAD6,C5AR1,DKKL1,POU3F2,ANG,ASCL1,POLI,POU2F2,JAG1,ACVR2B,NR3C2,ZNF69,DNMT3B,ZNF493,TOX3,ZNF624,ZNF404,NOXRED1,BTAF1,TOB1,L3MBTL1,UFL1,CARD14,ABCG1,HIF3A,EAF2,TRIM22,ZNF846,MAK,ACSL6,ZNF460,TRIM5,CDK5RAP3,ZNF780B,PDCD4,NKAP,HLTF,EDA2R,ZNF138,PRDM5,SCX,INSIG2,TMF1,REST,ZNF680,TET1,BTG2,RAP2C,NADK2,BAZ2B,ZNF217,FGF2,PLAGL1,GFPT2,RRM2B,AMH,INTS2,CHD7,ZNF273,CHD1,SKIL,PAXBP1,CAPRIN2,ZNF805,ZNF267,JMJD1C,TRIM52
GO:BP	multicellular organismal reproductive process	GO:0048609	0.035788845592901526	1.4462523100123004	ATP1A4,EDNRB,CFAP43,CFAP54,SPAG8,CYP26B1,MSTN,PTN,CFAP44,ADAM28,NLRP14,MAEL,MST1,SPATA9,NPPC,NR2F2,ACRBP,RGN,TEX14,CFAP69,LGR4,PTCH1,CATSPERG,BMPR1B,QKI,NHLH2,TDRP,MNS1,DKKL1,DNHD1,ANG,SPATA6L,DRC7,WEE2,SPATA6,WNT3,MAK,IFT81,DSG2,TTC26,PABPC1L,TMF1,SPEF2,AMH,NPHP1,SKIL
GO:BP	regulation of nervous system development	GO:0051960	0.036	1.4421193056965145	PRTG,TPBG,LRP2,GBX2,NTRK2,PTN,WNT5A,PROX1,HES5,PLAG1,GOLGA4,DLL1,SLITRK2,DUSP10,CRABP2,PTPN13,CXCR4,RASSF10,ASCL1,NPHP3,UFL1,WNT3,HLTF,REST,BTG2,FGF2,CHD7,SKIL,CAPRIN2
GO:BP	epithelial cilium movement involved in extracellular fluid movement	GO:0003351	0.036674786447034355	1.4356324063229782	CFAP43,CFAP54,CCDC39,DNAI1,RFX3,OFD1,NPHP3,SPEF2
GO:BP	muscle tissue development	GO:0060537	0.038	1.4221042398259318	ERBB4,ANKRD33,NPHS1,KEL,LRP2,TFAP2B,CYP26B1,ZIC3,MSTN,WNT5A,MEIS1,PROX1,NR2F2,DLL1,PTCH1,NPPA,MYH15,MEGF10,TNNI3,MTM1,PDLIM5,DSG2,SCX,BTG2,FGF2,CHD7,SKIL
GO:BP	regulation of RNA metabolic process	GO:0051252	0.039	1.4090367258408325	PRAME,PRAMEF12,ERBB4,PTF1A,GSX1,PAX3,EDNRB,PRDM13,EN2,OLIG3,PAX7,VGLL3,HESX1,SP9,LHX5,FOXB1,ZIC4,SOX13,SPAG8,IRX5,DMBX1,TFAP2B,ILDR2,THRB,ZIC5,ZIC3,SAP25,MSTN,ZNF83,GBX2,INO80B,WNT5A,ZIC1,MUC1,EBF1,ZNF117,FOXP2,MEIS1,CEP290,MAEL,EBF2,PROX1,MAF,NR2F2,HES5,IRX2,ZNF334,RGN,RORA,PLAG1,LGR4,RFX3,CDK6,IRX1,GOLGB1,AASS,TPR,ZIC2,DLL1,YY2,PTCH1,TARBP1,HMGN5,CCND1,FANK1,NR2F1,ZNF483,BMPR1B,ZNF518A,AGER,RFX4,ZNF730,HSF4,QKI,HRSP12,NHLH2,ZNF107,YES1,CRABP2,ZNF90,SMAD6,AXIN2,POU3F2,ASCL1,POU2F2,JAG1,ACVR2B,NR3C2,ZNF69,DNMT3B,ZNF493,TOX3,ZNF624,ZNF404,BTAF1,TOB1,L3MBTL1,UFL1,CARD14,HIF3A,EAF2,TRIM22,ZNF846,MAK,ZNF460,TRIM5,CDK5RAP3,ZNF780B,PDCD4,NKAP,HLTF,EDA2R,ZNF138,PRDM5,SCX,TMF1,REST,ZNF680,TET1,BTG2,RAP2C,BAZ2B,ZNF217,FGF2,PLAGL1,AMH,INTS2,CHD7,ZNF273,CHD1,SKIL,PAXBP1,CAPRIN2,ZNF805,ZNF267,JMJD1C,TRIM52
GO:BP	spinal cord development	GO:0021510	0.039	1.4079268870048622	GSX1,OLIG3,LHX5,FOXB1,SOX13,INTU,ZIC1,PROX1,DLL1,PTCH1,RFX4,ASCL1
GO:BP	nephron development	GO:0072006	0.044745138831049316	1.3492541400916962	ERBB4,EDNRB,ANGPT1,NPHS1,TFAP2B,HES5,IRX2,LGR4,IRX1,DLL1,PTCH1,COL4A3,WNT2B,JAG1,FGF2

GO:BP	regulation of nucleobase-containing compound metabolic process	GO:0019219	0.0472014104216 69275	1.32604502 40524286	PRAME,PRAMEF12,ERBB4,PTF1A,GSX1,PAX3,EDNRB,PRDM13,EN2,OLIG3,PAX7,VGLL3,HESX1,PDK4,SP9,LHX5,FOXB1,ZIC4,SOX13,SPAG8,IRX5,DMBX1,TFAP2B,ILDR2,THRB,ZIC5,ZIC3,SAP25,MSTN,ZNF83,GBX2,INO80B,WNT5A,ZIC1,MUC1,MAPK15,EBF1,ZNF117,FOXP2,MEIS1,CEP290,MAEL,EBF2,PROX1,MAF,NPPC,NR2F2,HES5,IRX2,ZNF334,RGN,RORA,PLAG1,LGR4,RFX3,CDK6,IRX1,GOLGB1,AASS,TPR,ZIC2,DLL1,YY2,PTCH1,TARBP1,HMGN5,CCND1,FANK1,NR2F1,ZNF483,BMPR1B,ZNF518A,AGER,RFX4,ZNF730,HSF4,QKI,HRSP12,NHLH2,ZNF107,YES1,CRABP2,ZNF90,SMAD6,AXIN2,ATAD5,POU3F2,ASCL1,POU2F2,JAG1,ACVR2B,NR3C2,ZNF69,DNMT3B,ZNF493,TOX3,ZNF624,ZNF404,BTAF1,TOB1,L3MBTL1,UFL1,CARD14,HIF3A,RBBP6,EAF2,TRIM22,ZNF846,MAK,ZNF460,TRIM5,CDK5RAP3,ZNF780B,PDCD4,NKAP,HLTF,ANKRD31,EDA2R,ZNF138,PRDM5,SCX,TMF1,REST,ZNF680,SMC5,TET1,BTG2,RAP2C,BAZ2B,ZNF217,FGF2,PLAGL1,AMH,INTS2,CHD7,ZNF273,CHD1,ESCO1,SKIL,PAXBP1,CAPRIN2,ZNF805,ZNF267,JMJD1C,TRIM52
GO:BP	epithelial cell differentiation	GO:0030855	0.047	1.32446439 28606024	ERBB4,EDNRB,FOXB1,INTU,NPHS1,STRC,THRB,CYP26B1,WNT5A,PDPN,PROX1,MAF,BDH2,NPPC,NR2F2,HES5,CCDC78,RFX3,CDK6,DLL1,PTCH1,CCND1,HSF4,CXCR4,POU3F2,KRT10,ASCL1,USH2A,JAG1,ACVR2B,SCX,ADAM9,RAP2C,EXPH5,TTC8,FGF2,ACADVL,SKIL,TGM1
GO:BP	lymphatic endothelial cell fate commitment	GO:0060838	0.049	1.30955811 54637606	PDPN,PROX1,NR2F2
GO:BP	aromatic compound biosynthetic process	GO:0019438	0.05	1.30397399 53345135	PRAME,PRAMEF12,ERBB4,PTF1A,GSX1,PAX3,EDNRB,PRDM13,EN2,OLIG3,PAX7,VGLL3,HESX1,PDK4,SP9,LHX5,FOXB1,ZIC4,SOX13,SPAG8,IRX5,DMBX1,USP43,TFAP2B,THRB,ZIC5,ZIC3,SAP25,MSTN,ZNF83,GBX2,INO80B,WNT5A,ZIC1,MUC1,MAPK15,EBF1,ZNF117,FOXP2,MEIS1,CEP290,MAEL,EBF2,PROX1,MAF,NPPC,NR2F2,HES5,IRX2,ZNF334,RGN,RORA,PLAG1,LGR4,RFX3,CDK6,IRX1,GOLGB1,AASS,TPR,ZIC2,DLL1,YY2,PTCH1,TARBP1,HMGN5,CCND1,FANK1,NR2F1,NPPA,ZNF483,BMPR1B,ZNF518A,AGER,RFX4,ZNF730,HSF4,AK7,NHLH2,ZNF107,YES1,ATG16L2,CRABP2,ZNF90,SMAD6,C5AR1,POU3F2,ANG,ASCL1,POLI,POU2F2,JAG1,ACVR2B,NR3C2,ZNF69,DNMT3B,ZNF493,TOX3,ZNF624,ZNF404,BTAF1,TOB1,L3MBTL1,UFL1,CARD14,HIF3A,EAF2,TRIM22,ZNF846,MAK,ACSL6,ZNF460,TRIM5,CDK5RAP3,ZNF780B,PDCD4,NKAP,HLTF,EDA2R,ZNF138,PRDM5,SCX,TMF1,REST,ZNF680,TET1,BTG2,RAP2C,NADK2,BAZ2B,ZNF217,FGF2,PLAGL1,GFPT2,RRM2B,AMH,INTS2,CHD7,ZNF273,CHD1,SKIL,PAXBP1,CAPRIN2,ZNF805,ZNF267,JMJD1C,TRIM52
GO:CC	cilium	GO:0005929	1.6504661433817 03e-10	9.78239338	DNAH12,ATP1A4,CFAP70,CFAP43,DNAH8,CFAP54,SPAG8,INTU,C4ORF47,STRC,CCDC39,CFAP44,MAPK15,RPGR,CEP290,DNAH7,CNGA4,DNAI1,CFAP69,FAM166A,PTCH1,HHIP,LCA5L,FANK1,PIFO,SPATA4,CATSPERG,CEP162,AK7,DHRS3,OFD1,MNS1,TBC1D32,CEP83,DNAH2,DYNLRB2,ARR3,DNHD1,SPATA6L,USH2A,IQUB,DRC7,CNTRL,DNAH6,LCA5,NPHP3,RPGRIP1,SPATA6,MAK,IFT81,TTC26,OPN1SW,SPEF2,EFHC2,TTC8,NPHP1,IFT80
GO:CC	axoneme	GO:0005930	4E-06	5.45486369 5108642	DNAH12,CFAP70,CFAP43,DNAH8,CFAP54,SPAG8,CCDC39,DNAH7,DNAI1,LCA5L,SPATA4,CEP162,MNS1,DNAH2,DNHD1,DNAH6,LCA5,RPGRIP1,MAK,EFHC2
GO:CC	ciliary plasm	GO:0097014	4E-06	5.40895125 9382764	DNAH12,CFAP70,CFAP43,DNAH8,CFAP54,SPAG8,CCDC39,DNAH7,DNAI1,LCA5L,SPATA4,CEP162,MNS1,DNAH2,DNHD1,DNAH6,LCA5,RPGRIP1,MAK,EFHC2
GO:CC	motile cilium	GO:0031514	8E-05	4.08982722 3160957	ATP1A4,CFAP70,CFAP43,DNAH8,INTU,CFAP44,RPGR,DNAI1,CFAP69,CATSPERG,AK7,OFD1,MNS1,DNAH2,DNHD1,SPATA6L,IQUB,DRC7,SPATA6,MAK,IFT81,SPEF2,NPHP1
GO:CC	microtubule organizing center	GO:0005815	9E-05	4.06033010 6510933	CFAP70,INTU,C4ORF47,CCDC102B,CCNB3,MAPK15,RPGR,CEP290,TEX9,DNAI1,HEPACAM2,CCDC78,PLAG1,FAM166A,CDK6,FANK1,PIFO,CEP112,CCDC18,CEP162,CCDC88B,OFD1,CCDC14,YES1,CEP83,AXIN2,DCDC2B,DYNLRB2,RASSF10,USH2A,CNTRL,LCA5,CEP44,RBBP6,MAK,IFT81,CDK5RAP3,SPICE1,LRRCC1,TTC26,C7ORF31,SASS6,EVI5,CCDC146,EFHC2,WDR90,TTC8,IFT80,CAPRIN2
GO:CC	axonemal dynein complex	GO:0005858	3E-04	3.50285958 47979935	DNAH12,CFAP70,DNAH8,DNAH7,DNAI1,DNAH2,DNHD1
GO:CC	dynein complex	GO:0030286	4E-04	3.42029083 61987836	DNAH12,CFAP70,DNAH8,DNAH7,DNAI1,TPR,DNAH2,DYNLRB2,DNHD1,DNAH6

GO:CC	microtubule cytoskeleton	GO:0015630	0.001	2.8957929202423083	DNAH12,CFAP70,DNAH8,SPAG8,INTU,C4ORF47,CCDC102B,CCNB3,MAP1LC3B2,MAPK15,RPGR,CEP290,DNAH7,NPHP3-ACAD11,TEX9,DNAI1,CENPE,HEPACAM2,CCDC78,DCDC1,PLAG1,FAM166A,CDK6,TPR,FANK1,PIFO,CEP112,CCDC18,CEP162,CCDC88B,OFD1,MNS1,CCDC14,YES1,CEP83,DNAH2,AXIN2,DCDC2B,DYNLRB2,RASSF10,DNHD1,USH2A,CNTRL,DNAH6,LCA5,CEP44,RBBP6,MAK,IFT81,CDK5RAP3,SPICE1,LRRCC1,TTC26,RMDN2,C7ORF31,SASS6,SPEF2,EVI5,CCDC146,EFHC2,WDR90,REEP3,TTC8,IFT80,CAPRIN2
GO:CC	non-motile cilium	GO:0097730	0.003	2.5584789674317445	ATP1A4,C4ORF47,STRC,RPGR,CEP290,CFAP69,DHRS3,ARR3,USH2A,LCA5,RPGRIP1,MAK,OPN1SW,TTC8,NPHP1,IFT80
GO:CC	9+0 non-motile cilium	GO:0097731	0.003	2.465651298782679	ATP1A4,C4ORF47,RPGR,CEP290,DHRS3,ARR3,USH2A,LCA5,RPGRIP1,MAK,OPN1SW,TTC8,NPHP1,IFT80
GO:CC	ciliary basal body	GO:0036064	0.004	2.428024062837976	CFAP70,INTU,MAPK15,RPGR,CEP290,FAM166A,FANK1,PIFO,OFD1,USH2A,LCA5,IFT81,TTC26,EFHC2,TTC8,IFT80
GO:CC	plasma membrane bounded cell projection cytoplasm	GO:0032838	0.004	2.418010610867108	DNAH12,CFAP70,CFAP43,DNAH8,CFAP54,SPAG8,CCDC39,DNAH7,DNAI1,LCA5L,SPATA4,CEP162,MNS1,DNAH2,DNHD1,DNAH6,LCA5,RPGRIP1,MAK,EFHC2
GO:CC	centriole	GO:0005814	0.005	2.2987334338686374	INTU,CCDC102B,MAPK15,CEP290,CCDC78,CEP162,OFD1,CEP83,CEP44,IFT81,SPICE1,LRRCC1,SASS6,CCDC146,WDR90
GO:CC	9+2 motile cilium	GO:0097729	0.0138363919566322707	1.8589771438106357	ATP1A4,CFAP70,CFAP43,DNAH8,RPGR,DNAI1,CFAP69,CATSPERG,MNS1,DNAH2,DNHD1,SPATA6L,SPATA6,IFT81,SPEF2
GO:CC	cytoskeleton	GO:0005856	0.0158189540704256 96	1.8008222349002379	DNAH12,CFAP70,CFAP43,GDPD2,DNAH8,PLEKHH2,CFAP54,SPAG8,INTU,C4ORF47,INPP5D,CCDC102B,CCDC39,CCNB3,MAP1LC3B2,CFAP44,MAPK15,RPGR,CEP290,DNAH7,NPHP3-ACAD11,UACA,TEX9,DNAI1,CENPE,HEPACAM2,CCDC78,DCDC1,PLAG1,FAM166A,CDK6,MYO15B,TPR,LCA5L,FANK1,PIFO,MYO5C,CEP112,SPATA4,CCDC18,MYH15,KITLG,CEP162,CCDC88B,OFD1,MNS1,TRPC4,CCDC14,YES1,PTPN13,CEP83,DNAH2,TNNI3,AXIN2,DCDC2B,DYNLRB2,RASSF10,DNHD1,KRT10,ANG,USH2A,IQUB,DRC7,CNTRL,DNAH6,PRICKLE4,LCA5,EPHA3,CEP44,RPGRIP1,RBBP6,MAK,IFT81,MYH7B,PNN,FILIP1,CDK5RAP3,SPICE1,PDLIM5,LRRCC1,TTC26,RMDN2,C7ORF31,SASS6,SPEF2,EVI5,CCDC146,EFHC2,WDR90,REEP3,TTC8,NPHP1,IFT80,CAPRIN2
GO:CC	cell projection	GO:0042995	0.021	1.6799366905313298	ROBO3,PKHD1L1,DNAH12,ATP1A4,TPBG,CFAP70,CABP4,CFAP43,GDPD2,DNAH8,PLEKHH2,CFAP54,SPAG8,ANGPTL3,INTU,C4ORF47,ANGPT1,ADGRV1,NPHS1,GRM1,LRP2,STRC,CCDC39,NTRK2,CFAP44,GAD2,MAPK15,RPGR,CEP290,PDPN,DNAH7,CNGA4,DNAI1,CFAP69,FAM166A,CDK6,MYO15B,PTCH1,HHIP,LCA5L,FANK1,PIFO,NPPA,SPATA4,CATSPERG,BMPR1B,MCC,KITLG,CEP162,MEGF10,AK7,GNB3,DHRS3,OFD1,MNS1,PTPN13,TBC1D32,CEP83,DNAH2,DYNLRB2,ARR3,EEA1,DNHD1,ANG,SPATA6L,USH2A,MTM1,IQUB,DRC7,CNTRL,MTTP,DNAH6,LCA5,NPHP3,EPHA3,UFL1,RPGRIP1,SPATA6,MAK,IFT81,NMU,PDLIM5,NRXN3,UNC80,TTC26,OPN1SW,SPEF2,EFHC2,TTC8,NPHP1,IFT80
GO:CC	Golgi cis cisterna	GO:0000137	0.023	1.637604564854809	GOLGA6B,GOLGA6A,GOLGA8B,GOLGA8A,GOLGA6C,GOLGA8N
GO:CC	photoreceptor cell cilium	GO:0097733	0.0259215616152547 08	1.5863388384318902	ATP1A4,RPGR,CEP290,DHRS3,ARR3,USH2A,LCA5,RPGRIP1,MAK,OPN1SW,TTC8,NPHP1
GO:CC	photoreceptor connecting cilium	GO:0032391	0.0268882321858088 25	1.5704377502938538	CEP290,USH2A,LCA5,RPGRIP1,MAK,TTC8,NPHP1
GO:CC	cytoplasmic region	GO:0099568	0.0280207559413165 02	1.5525201525365426	DNAH12,CFAP70,CFAP43,DNAH8,CFAP54,SPAG8,CCDC39,DNAH7,DNAI1,LCA5L,SPATA4,CEP162,MNS1,DNAH2,DNHD1,DNAH6,LCA5,RPGRIP1,MAK,EFHC2

GO:CC	plasma membrane bounded cell projection	GO:0120025	0.044	1.360160721960412	ROBO3,PKHD1L1,DNAH12,ATP1A4,TPBG,CFAP70,CABP4,CFAP43,GDPD2,DNAH8,PLEKHH2,CFAP54,SPAG8,ANGPTL3,INTU,C4ORF47,ANGPT1,ADGRV1,GRM1,LRP2,STRC,CCDC39,NTRK2,CFAP44,GAD2,MAPK15,RPGR,CEP290,PDPN,DNAH7,CNGA4,DNAI1,CFAP69,FAM166A,CDK6,MYO15B,PTCH1,HHIP,LCA5L,FANK1,PIFO,SPATA4,CATSPERG,BMPR1B,MCC,KITLG,CEP162,AK7,GNB3,DHRS3,OFD1,MNS1,PTPN13,TBC1D32,CEP83,DNAH2,DYNLRB2,ARR3,EEA1,DNHD1,ANG,SPATA6L,USH2A,MTM1,IQUB,DRC7,CNTRL,MTTP,DNAH6,LCA5,NPHP3,EPHA3,UFL1,RPGRIP1,SPATA6,MAK,IFT81,NMU,UNC80,TTC26,OPN1SW,SPEF2,EFHC2,TTC8,NPHP1,IFT80

Table S7: Upregulated genes identified from single cell sequencing in HUVECs cultured with cortical organoids and monocultured HUVECs

Upregulated Genes Identified from HUVECs cultured in scaffold containing a cortical organoid (22)

	p_val	*avg_log2FC*	*pct.1*	*pct.2*	*p_val_adj*
CLEC2B	1.22E-20	0.100918582	0.837	0.961	2.43E-17
NQO1	7.59E-70	0.100946745	0.815	0.971	1.52E-66
TM4SF1	1.59E-153	0.103208987	0.882	0.994	3.17E-150
MT2A	2.29E-158	0.10417551	0.948	1	4.58E-155
CRIP2	1.52E-34	0.105902761	0.746	0.789	3.04E-31
ARHGDIB	5.90E-39	0.106169515	0.807	0.926	1.18E-35
DOCK4	1.31E-13	0.106855267	0.93	0.972	2.61E-10
AKR1C3	1.42E-13	0.108056527	0.771	0.905	2.84E-10
ADD3	0.005018823	0.108669093	0.771	0.961	1
STC1	0.000576808	0.1096926	0.791	1	1
S100A6	4.81E-283	0.110543574	0.953	1	9.61E-280
IGFBP4	5.70E-33	0.110805083	0.809	0.927	1.14E-29
HOPX	1.48E-28	0.113211927	0.75	0.85	2.95E-25
KRT18	6.38E-78	0.113328684	0.84	0.974	1.28E-74
KLHDC8B	1.15E-10	0.115302165	0.714	0.911	2.31E-07
MT1X	0.000562956	0.122793471	0.742	0.884	1
DUT	2.24E-57	0.123754511	0.802	0.907	4.48E-54
ECSCR	6.24E-58	0.128371906	0.795	0.931	1.25E-54
TANC2	1.06E-07	0.133207303	0.881	0.966	0.000211617
GNG11	6.07E-79	0.138967576	0.843	0.977	1.21E-75
EFEMP1	2.24E-146	0.163950409	0.932	0.979	4.48E-143
CLEC14A	4.99E-55	0.165654543	0.821	0.98	9.98E-52

Upregulated Genes Identified from HUVEC Monocultures (27)

	p_val	*avg_log2FC*	*pct.1*	*pct.2*	*p_val_adj*
IGFBP2	0	-0.312145007	0.456	1	0
COL1A1	0	-0.226254794	0.445	1	0
COL6A1	0	-0.201535795	0.332	1	0
SNAPC1	1.97E-70	-0.183487827	0.711	0.797	3.94E-67
COL6A2	0	-0.177369206	0.34	0.997	0
AC025419.1	0.050038094	-0.165086592	0.504	0.56	1
ANKRD1	1.07E-78	-0.164932561	0.6	0.755	2.14E-75
CRIM1	1.27E-135	-0.153589563	0.749	0.886	2.54E-132
COL6A3	0	-0.152958101	0.27	0.967	0
CDH2	5.42E-103	-0.145584301	0.542	0.738	1.08E-99
HMGCS1	1.12E-141	-0.144625997	0.567	0.779	2.23E-138
IGFBP5	0	-0.142507459	0.43	1	0
ADAMTS6	4.95E-33	-0.140905986	0.567	0.69	9.90E-30
HIF1A-AS3	2.42E-96	-0.137935907	0.683	0.857	4.84E-93
CNTNAP2	0	-0.130243858	0.32	0.775	0
COL4A1	1.25E-160	-0.127047777	0.814	0.999	2.49E-157
DDIT3	4.55E-56	-0.121810872	0.56	0.736	9.10E-53
ITM2C	0	-0.120427571	0.3	0.975	0
PRKG1	1.19E-58	-0.115208247	0.288	0.427	2.38E-55
RPS4Y1	0	-0.113407747	0.28	0.974	0
PTX3	1.63E-60	-0.112349416	0.701	0.863	3.26E-57
HSPA5	3.68E-49	-0.107570252	0.842	0.992	7.36E-46
AUTS2	0	-0.104751581	0.287	0.973	0
PAPPA	0	-0.102981513	0.375	0.883	0
TUBB2B	0	-0.101991637	0.287	0.998	0
NTM	0	-0.101932918	0.368	0.915	0
TENM3	0	-0.101283872	0.193	0.934	0
